# Ligand Type Guided Keto‐Arylation Enables Modular Total Synthesis of Polycyclic CBS Xanthones

**DOI:** 10.1002/anie.202513532

**Published:** 2025-07-22

**Authors:** Jonas W. Meringdal, Vivienne Prangenberg, Tim Treiber, Andreas J. Schneider, Leon Honsdorf, Dirk Menche

**Affiliations:** ^1^ Kekulé‐Institute for Organic Chemistry and Biochemistry University of Bonn Gerhard‐Domagk‐Str. 1 53121 Bonn Germany

**Keywords:** Davis oxidation, Keto‐arylation, Ligand types, Polycyclic xanthones, Total synthesis

## Abstract

The first total synthesis of the potent polycyclic xanthone antibiotics CBS72, CBS87 and CBS100 was accomplished by a modular strategy featuring a very demanding intermolecular aromatic keto‐arylation. Central to the solution was a recently‐developed ligand type approach, rather than brute force screening, demonstrating the usefulness of this novel concept in complex target synthesis. Additional key features include an asymmetric Davis hydroxylation proceeding with only catalytic amounts of base, thus enabling the conversion of a highly sensitive, elaborate substrate. Furthermore, a late‐stage aminolysis completed the polycyclic framework, circumventing laborious protective group chemistry. Together, this strategy provides a concise, high‐yielding access, confirming the full architecture of this most potent class of polyaromatic xanthones, and establishes ligand types as a powerful design tool for sophisticated cross‐couplings.

## Graphical abstract

A ligand type concept guided a central aromatic keto‐arylation in the first total syntheses of CBS72, CBS87 and CBS100. Davis oxidation with catalytic base and late‐stage aminolysis completed the route. This strategy provides modular, concise and high‐yielding access to uncharted polycyclic xanthones. Meanwhile, ligand types emerge as a powerful design tool in sophisticated cross couplings.

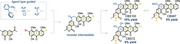

Densely functionalized polycyclic angular xanthones have attracted considerable synthetic interest due to their potent antimicrobial activity and structural complexity (Figure 1).^[^ ^1^ ^]^ Recent accomplishments include the total synthesis of FD594 (**1**) and kigamicin A precursor **2** (top).^[^ ^2^, ^3^, ^4^ ^]^ However, despite considerable efforts,^[^ ^5^, ^6^, ^7^ ^]^ a synthetic route to the most potent class of these polycyclic heterocycles,^[^ ^1^ ^]^ the CBS family isolated from *Streptomyces albus*,^[^ ^8^, ^9^, ^10^ ^]^ remains elusive (bottom). As shown for three particularly efficacious members, CBS72 (**3**), CBS87 (**4**) and CBS100 (**5**), they may demonstrate antibacterial potencies in low nanomolar concentrations, which exceed all other polycyclic angular xanthones by one to two orders of magnitude.^[^ ^9^, ^11^, ^12^ ^]^ Characteristic features of their hexacyclic structure include an unsubstituted C18‐lactam (orange) in combination with distinct C25‐C26‐oxygenation patterns (blue), which were assigned by reported NMR data and an x‐ray structure of a natural analog.^[^ ^8^, ^9^, ^13^, ^14^ ^]^ These structural elements are integral to their bioactivities and pose unsolved synthetic challenges.^[^ ^1^, ^9^ ^]^

**Figure 1:**
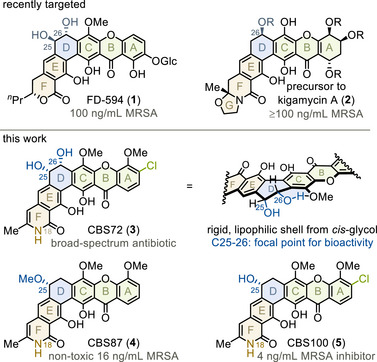
Antimicrobial polycyclic xanthone natural products. Minimal inhibitory concentrations in grey. Glc, glucoside, see ref. [2].

In particular, the versatile oxygenation pattern of **3‐5** deserves special attention. CBS72 (**3**) contains a C25‐C26‐glycol in a unique *cis*‐configuration. This *cis*‐glycol is crucial for broad‐spectrum activity against multidrug‐resistant bacteria.^[^ ^1^, ^9^ ^]^ Possibly, the resulting lipophilic shell^[^ ^9^, ^13^ ^]^ facilitates binding to C_55_‐lipid bactoprenol, a murein carrier for cell wall synthesis.^[^ ^14^, ^15^, ^16^ ^]^ In contrast, a *trans‐*glycol orients perpendicular to the D‐ring, disturbing widespan coordination (not shown).^[^ ^11^, ^17^ ^]^ Moreover, CBS87 (**4**) and CBS100 (**5**) are distinctly oxygenated solely at C25. These members specifically act against methicillin‐resistant *Staphylococcus aureus* (MRSA), achieving 30‐fold selectivity over human liver cells and expressing single digit nanomolar activity.^[^ ^9^ ^]^

As shown in Figure 2 (top), established fragment couplings may efficiently access alkyne **6** or stilbene **8**.^[^ ^2^, ^3^ ^]^ However, electronic factors limit mono‐oxygenation to C26 (**7**).^[^ ^3^ ^]^ Furthermore, asymmetric dihydroxylation of (*E*)‐stilbenes selectively furnishes *trans‐*diols such as **9**, but the mirror symmetry of (*Z*)‐stilbenes would result in a mostly racemic *cis‐*diol.^[^ ^18^, ^19^ ^]^ In contrast, to access desired oxygenation patterns **11** and **12** for **35**, our approach relied on late redox manipulation of deoxybenzoin **10**, envisioned from a very demanding aromatic keto‐arylation (bottom).

**Figure 2:**
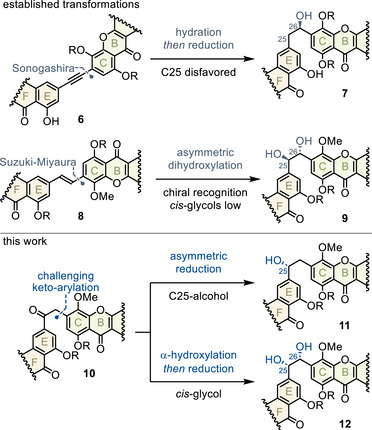
Fragment coupling and oxygenation strategies.

Despite the high potential, ketone cross‐couplings are largely underdeveloped and reported intermolecular cases have been restricted to simplified starting materials, presumably due to limited functional group tolerance and challenging selectivity.^[^ ^20^ ^]^ A rare counterexample is Nicolaou's late‐stage fragment union in gukulenin B, which achieved 43% yield but required 91 mol‐% catalyst (2022).^[^ ^21^ ^]^ An intermolecular keto‐arylation of advanced aromatic intermediates has yet to be reported,^[^ ^22^ ^]^ most likely due to strong, substrate‐specific sensitivity of the employed ligand.^[^ ^23^ ^]^

Specifically for these cases, we recently proposed a ligand type approach.^[^ ^24^ ^]^ Building on Doyle and Sigman's seminal work (2021–2023),^[^ ^25^, ^26^, ^27^ ^]^ we concluded that examining a small set of archetypical ligands, characterized by their binding mode, suffices to identify promising catalysts, arguing that ligands of the same type exhibit similar reactivity and selectivity.

As shown in Figure 3, late introduction of the characteristic unsubstituted C18‐lactam, a strong catalyst poison, completes our retrosynthesis (orange).^[^ ^1^, ^28^ ^]^ This strategy circumvents protective group chemistry and permits the use of reactive catalysts for modular oxygenation (blue) and keto‐arylation (red). Fragmentation of unified precursor **10** leads to isocoumarin **13** and xanthone **14**, envisioned to stem from a series of coupling and cyclization reactions.^[^ ^29^, ^30^, ^31^ ^]^

**Figure 3:**
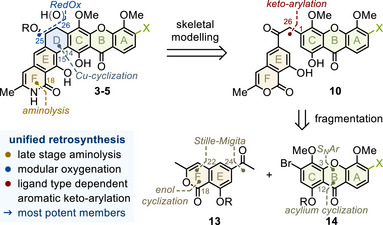
Unified retrosynthetic analysis.

For synthesis of isocoumarin **13**, we adopted the sequential crosscoupling strategy of Hosokawa and Tatsuta to introduce the F‐ring on readily available bistriflate **15** (Figure 4 and Supporting Information).^[^ ^29^ ^]^

**Figure 4:**
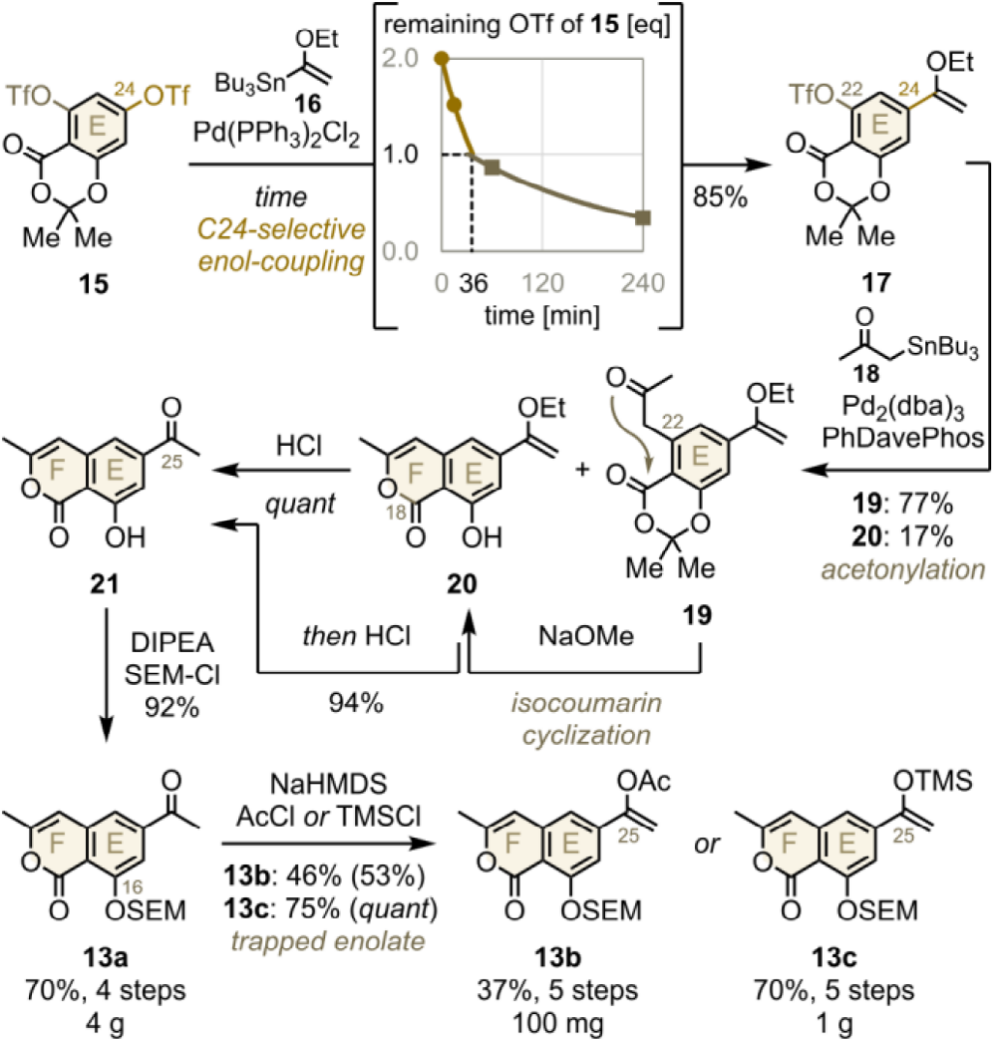
Isocoumarin fragment preparation. dba, dibenzylideneacetone; DIPEA, diisopropylethylamin; HMDS, hexamethyldisilazide; PhDavePhos, 2‐diphenylphosphino‐2′‐(*N*,*N*‐dimethylamino)‐biphenyl; SEM, 2‐(trimethylsilyl)ethoxymethyl; TMS, trimethylsilyl.

Since cross‐coupling initially occurs at less hindered C24, the acetophenone had to be introduced first.^[^ ^29^ ^]^ To facilitate future basic F‐ring cyclization, we delayed the release of acidic acetophenone from enol **17** until the isocoumarin skeleton was complete. We then determined the optimal time to form mono‐enol ether **17** (orange) without overreacting to the C22‐enol ether (brown) by using two equivalents **16** at low catalyst loading (chart).^[^ ^32^ ^]^ Extrapolation of the resulting exponential decay gave an optimal reaction time of 36 minutes at 45 °C (see Supporting Information for a detailed derivation).

For scale‐up, reduced catalyst loading and temperature extended the optimal time window, yielding 85% of **17** (Table S2). Then, a second cross‐coupling at C22 with acetone‐donor **18** installed the open F‐ring in **19** (77%), which partially cyclized during the reaction to **20** through *O‐*nucleophilic attack on C18 (17%).^[^ ^29^, ^33^ ^]^ As planned, sodium methanolate completed isocoumarin cyclization,^[^ ^34^ ^]^ before acidic hydrolysis released acetophenone **21** (89% from **17**). Ensuing SEM protection of the C16‐phenol yielded four grams of isocoumarin **13** in 70% over 4 steps.^[^ ^35^ ^]^ Notably, only SEM groups provided downstream solubility and stability.^[^ ^36^ ^]^ As alternative carbon nucleophiles for subsequent keto‐arylation (see below), the exceedingly labile^[^ ^37^ ^]^ enolate of **13a** was trapped at 90 °C and purified via HPLC or cryogenic column chromatography, furnishing acetyl enol‐ether **13b** (46%) and silyl enol‐ether **13c** (75%).

With isocoumarin **13** in hand, the next target was xanthone **14** (Figure 5). Previously, poor regioselectivity and side reactions impeded effective chlorine introduction.^[^ ^35^, ^36^ ^]^ Therefore, we started from commercially available 3‐chlorosalicylaldehyde (**22**), using the C5‐aldehyde as masked phenol (dark green) to construct the xanthone skeleton.

**Figure 5:**
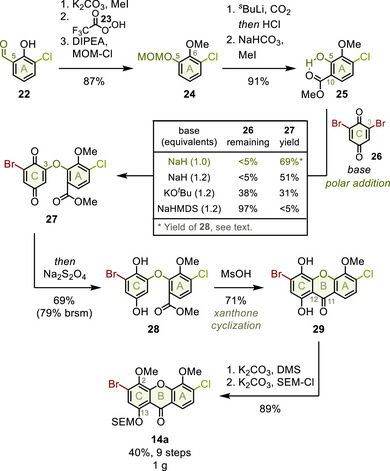
Xanthone fragment preparation. DMS, dimethyl sulfate; MOM, methoxymethyl.

First, etherification produced the C6anisole. Next, Dakin oxidation with trifluoroperacetic acid (**23**) unmasked the C5phenol,^[^ ^38^ ^]^ which was converted to directing MOM ether **24** (87%). *ortho*‐Lithiation with carbon dioxide introduced the C10‐carboxylate, completing the A‐ring oxygenation pattern. For subsequent coupling, the MOM group was hydrolyzed and the carboxylic acid was selectively methylated using sodium bicarbonate as weak base, providing three grams of methyl salicylate **25** (91%).^[^ ^39^ ^]^

Previously, polar addition to **27** proved challenging as unreacted quinone **26** limited scaleup.^[^ ^31^, ^40^ ^]^ Suspecting that an intramolecular hydrogen bond (grey) necessitated an excess of weak base and base‐labile **26**, we now focused on a slight excess of a strong base and **26** (table).^[^ ^41^ ^]^ Sodium hydride proved ideal, since substitution of the base drastically reduced conversion (further parameters in Table S3). Furthermore, using exactly one equivalent suppressed side product formation, yielding 69% **28** after in situ reduction with sodium dithionite.

Next, strongly acidic conditions fused the B‐ring between C11 and C12, completing xanthone skeleton **29** (71%).^[^ ^31^ ^]^ Then, methylation of the more acidic phenol selectively introduced the C2‐anisole. Finally, SEM protection of the C13‐phenol yielded one gram of xanthone 14a in 40% over nine steps.^[^ ^35^ ^]^

With fragment syntheses completed, we focused on the central keto‐arylation to **10a** (Figure 6). To efficiently explore a wide reaction space, we combined possible reaction conditions (**A‐D**)^[^ ^42^, ^43^, ^44^, ^45^ ^]^ with suitable ligand types (**I‐III**) following our recently developed concept (top part of Figure).^[^ ^24^, ^25^ ^]^ Then, a focused set of prevalent ligands was selected,^[^ ^20^, ^46^ ^]^ for example, PhDavePhos was chosen as type **I** ligand for couplings **C** and **D** (see also **17**, Figure 4).^[^ ^3^, ^29^, ^33^ ^]^

**Figure 6:**
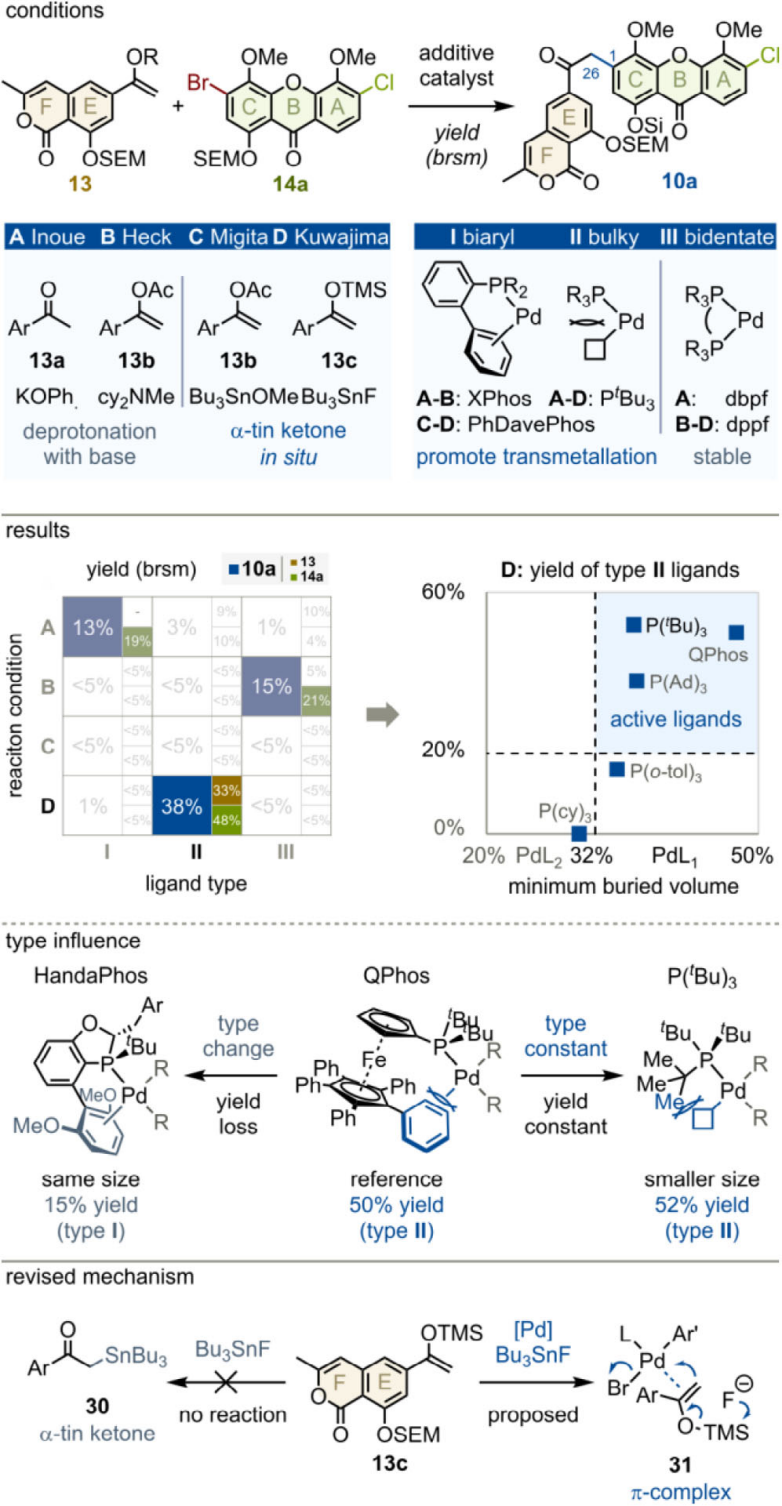
Ligand type dependent aromatic keto‐arylation. Ad, adamantyl; cy, cyclohexyl; dbpf, 1,1′‐bis(di‐*tert*‐butylphosphino)ferrocene. dppf, 1,1′‐bis(diphenylphosphino)ferrocene; tol, tolyl; XPhos, 2‐dicyclohexylphosphino‐2′,4′,6′‐triisopropylbiphenyl.

Reaction monitoring revealed three promising combinations: **A‐I**, **B‐III** and **D‐II** (left chart, detailed results in Tables S4–S8). Crucially, each condition required a completely different catalyst, efficiently identified using ligand types. Due to severe base‐lability of **13**, conditions **A** and **B** were discarded (Table S9). For condition **D**, a high temperature without additives^[^ ^47^, ^48^, ^49^ ^]^ proved optimal (Tables S10, S11). Exploring further type **II** ligands showed that mono‐ligating variants (PdL_1_) were ideal, likely promoting the challenging transmetallation as previously proposed (right chart, blue quadrant, Tables S12, S13).^[^ ^25^ ^]^

To verify our approach, two ligands with identical steric and electronic profiles but different ligand types were evaluated (lower part).^[^ ^24^, ^50^ ^]^ As expected, QPhos^[^ ^51^ ^]^ was three times more active than HandaPhos.^[^ ^52^ ^]^ The difference lies in coordination: HandaPhos’ lower biaryl ring can facially bind to the metal center (type **I**), whereas QPhos’ phenyl groups block this interaction (type **II**). By contrast, tri‐*tert*‐butylphosphine,^[^ ^49^, ^53^ ^]^ though smaller, yielded similar results as QPhos, consistent with their shared type **II**. Overall, these results confirm the central importance of the ligand type for complex cross‐couplings, promoting their application for sophisticated bond formation.^[^ ^24^ ^]^

Finally, contrary to the current understanding,^[^ ^45^, ^54^ ^]^ α‐stannyl ketone **30** was never observed from **13c** and tributyltin fluoride alone (Figure S6). Since **13c** was fully recovered in unsuccessful couplings, we propose that palladium coordination activates **13c**, enabling transmetallation upon desilylation with tributyltin fluoride as mild, sparingly dissolved^[^ ^55^ ^]^ fluoride donor (**31**). Extending this concept, even non‐fluoride additives might activate silyl enol‐ethers, broadening the narrow scope of this valuable coupling.^[^ ^20^, ^23^ ^]^

During scale‐up, we observed that the yield of **10a** improved when a marginal excess of ligand was employed, likely preventing the undesirable formation of palladium black (Table S14).^[^ ^46^ ^]^ These conditions furnished 62% of α‐arylated product **10a**, a twofold improvement over complementary, thoroughly optimized multistep protocols, highlighting the power of this challenging bond formation (Figure 7).^[^ ^3^ ^]^ Now, the remaining task was shaping the conjoint skeleton for ultimate D‐ring cyclization, targeting CBS100 (**5**).

**Figure 7:**
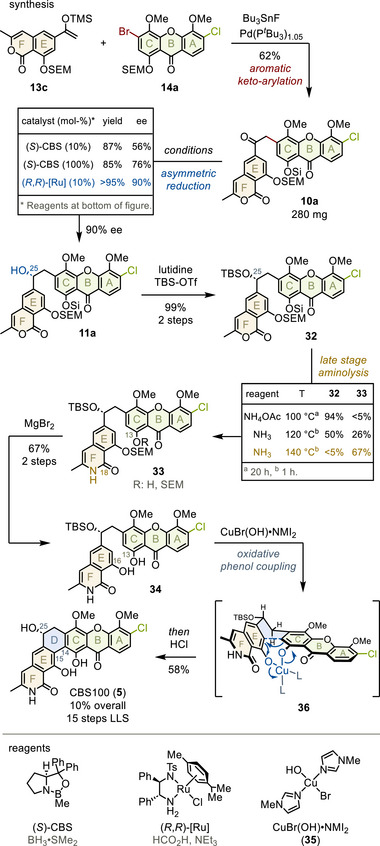
Total synthesis of CBS100. Si = SEM. NMI, *N*‐methylimidazole; TBS, *tert*‐butyldimethylsilyl.

First, asymmetric reduction installed the C25‐stereocenter (top table). While CBS reduction furnished only modestly enriched **11a**,^[^ ^56^ ^]^ Noyori transfer hydrogenation provided the desired enantiomer in 90% excess.^[^ ^3^, ^57^ ^]^ Ensuing TBS‐protection yielded 99% of benzyl ether **32** over two steps.^[^ ^58^ ^]^ Next, the C18‐lactam was introduced. Importantly, advanced intermediate **32** tolerated the forceful introduction of ammonia under high temperatures, furnishing lactam **33** and completing the skeleton of CBS100 (bottom table).^[^ ^59^, ^60^ ^]^ During aminolysis, the C13‐phenol in **33** was partially deprotected. Treating mixture **33** with magnesium bromide provided 67% **34** over two steps, liberating phenols C13 and C16 as anchor points for subsequent D‐ring formation.^[^ ^61^ ^]^

In the final step, **34** was subjected to readily available copper oxidant **35** obtained by an improved protocol (see Supporting Information for details). Presumably, ligand exchange forms oxidative addition complex **36**, a copper(II)‐bisphenolate preorganized for D‐ring cyclization between C14 and C15.^[^ ^2^, ^3^, ^29^ ^]^ Reductive elimination (blue arrows) would close the D‐ring, release a copper(0)‐complex and – after keto‐enol tautomerization at C13 and C16 (not shown) – provide the coupled bisphenol. Indeed, in situ deprotection of the C25‐benzylic alcohol furnished CBS100 (**5**) as the single product.

The very low solubility of CBS xanthones in a broad of solvents (including MeOH, THF, MeCN, acetone, DCM),^[^ ^9^, ^14^ ^]^ probably due to π‐stacking of the extended lipophilic shell (Figure 1), posed major challenges for isolation and purification. In detail, only neat alcohols could elute traces with low purity from silica gel, and considerable precipitation during high‐pressure liquid chromatography diminished separation and yield.^[^ ^62^ ^]^ Ultimately, extensive substrate‐specific reverse phase selection was required to provide high purity (94–98%), furnishing 58% CBS100 (**5**) in 10% yield over 15 steps. The purity may be further improved by crystallization from methanolic mixtures giving all natural products in >98% (see Figures S1, S3, S5). The analytical data of all natural products (see below) were in full agreement with reported values, confirming their assignments (Tables S18–S20).^[^ ^9^ ^]^

After establishing access to chlorinated member CBS100 (**5**), we aimed to expand our methodology to include hydrogen‐bearing CBS87 (**4**). Often, complex substrate effects hinder methodology transfer between substitution patterns, reducing the yield of individual transformations by a factor of two to three.^[^ ^63^ ^]^ Despite the narrow reaction window (see above), our optimized conditions demonstrated remarkable robustness (Figure 8).

**Figure 8:**
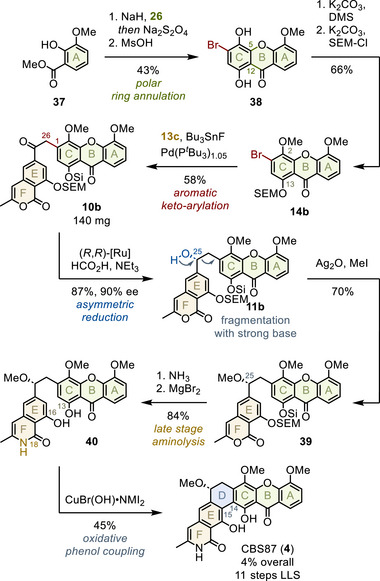
Total synthesis of CBS87.

In detail, ring annulation of commercially available methyl salicylate **37** with bromoquinone **26** provided 43% of xanthone skeleton **38** (top part).^[^ ^30^, ^31^ ^]^ After derivatization of phenols C2 and C13 (66%),^[^ ^35^ ^]^ **14b** was coupled with **13c**, providing α‐arylated **10b** in a near identical yield of 58%.^[^ ^45^, ^49^, ^53^ ^]^ Subsequent Noyori transfer hydrogenation of the C25‐ketone furnished 87% of benzyl alcohol **11b**, maintaining 90% enantioselectivity.^[^ ^3^, ^57^ ^]^

In contrast to CBS100 (**5**), CBS87 (**4**) additionally requires introduction of a C25‐methyl ether. As before, coupled skeleton **11b** exhibited severe base‐lability, resulting in Grob fragmentation under Williamson ether conditions (dark blue).^[^ ^64^ ^]^ In contrast, silver‐mediated cationic methylation provided 70% of desired benzyl ether **39**, underlining a reasonable stability of these substrates against (Lewis) acidic conditions.^[^ ^65^ ^]^

In the last steps, C18‐ammonia incorporation followed by liberation of phenols C13 and C16 completed CBS87 skeleton **40** in 84% yield.^[^ ^59^, ^61^ ^]^ Finally, copper‐mediated oxidative D‐ring cyclization furnished 45% CBS87 (**4**) in 4% yield over 11 steps.^[^ ^2^, ^3^, ^29^ ^]^

With transferability between xanthone fragments confirmed, the final target was *cis*‐glycol bearing CBS72 (**3**) (Figure 9).

**Figure 9:**
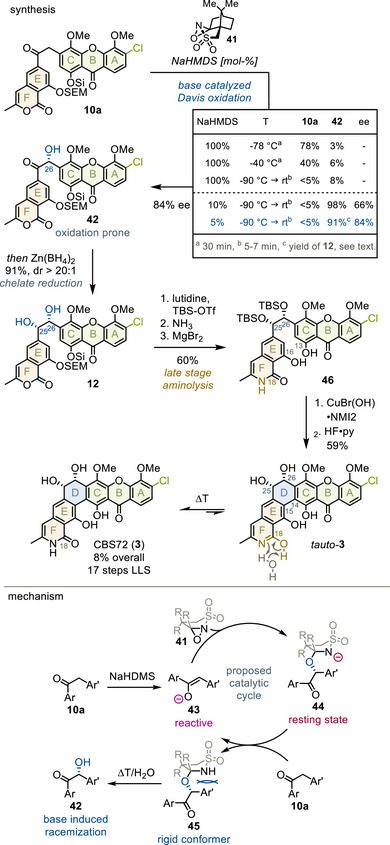
Total synthesis of CBS72. py, pyridine.

Initially, severe base‐lability of **10a** obstructed α‐hydroxylation under Davis conditions with one equivalent of base (table).^[^ ^66^, ^67^, ^68^ ^]^ However, we serendipitously found that a catalytic amount of base selectively formed **42**, when the initial red, cryogenic solution began to bleach (5 min). In line with previous observations,^[^ ^66^, ^67^ ^]^ this short reaction time was essential, since the C26‐alcohol slowly racemized under basic conditions (Table S17). Consequently, less base improved the enantiopurity of benzoin **42** to 84%.

To the best of our knowledge, this constitutes the first Davis oxidation with only catalytic amounts of base. Based on our observations, we believe that violet^[^ ^68^ ^]^ enolate **43** is rapidly converted to red **44** (bottom part). **44** must deprotonate **10a** for turnover, forming **45**. The persistence of a red color suggests that hemiaminals **44** and **45** are kinetically trapped at low temperatures, thereby stabilizing the C26‐stereocenter as originally proposed.^[^ ^67^ ^]^ Upon warming, both hemiaminals collapse, releasing racemizable **42**.

Air oxidation of delicate benzoin **42** inhibited isolation. However, prompt chelate‐controlled reduction with zinc borohydride furnished pivotal *cis‐*glycol **12** as single diastereomer in 91% yield.^[^ ^69^ ^]^ Subsequent glycol protection, aminolysis and phenol deprotection provided 60% of skeleton **46**, underlining the usefulness of this sequence. Finally, copper oxidation closed the D‐ring between C14 and C15. Surprisingly, both TBS ethers survived strongly acidic hydrolysis, suggesting severe steric shielding of the C25‐C26‐*cis*‐glycol. Astonishingly, fluoride deprotection furnished C18‐lactim *tauto*‐**3** as major product – an unprecedented tautomeric form among polycyclic xanthones (orange).^[^ ^1^, ^9^ ^]^ Although *tauto*‐**3** is thermodynamically disfavored, it possesses a considerable halflife (∼1 h at 100 °C) and persists after equilibration (9:1 ratio). A water‐assisted tautomerization mechanism appears plausible (brown), but the factors influencing tautomeric preference and their relevance to bioactivity remain unclear. In total, 59% CBS72 (**3**) was isolated in 8% yield over 17 steps.

In conclusion, we accomplished the first total synthesis of CBS100 (**5**), CBS87 (**4**) and CBS72 (**3**), uniquely oxygenated polycyclic xanthones, in concise modular sequences (15, 11 and 17 steps), high overall yields (10%, 4% and 8%) and good idealities^[^ ^70^, ^71^ ^]^ (61%, 69% and 59%; Figures S7, S8). These results compare favorably to previous routes to polycyclic xanthones,^[^ ^1^ ^]^ demonstrating the general usefulness of the strategy and procedures developed herein. In detail, site‐selective, sequential StilleMigita coupling and polar ring annulation set the stage for a series of unprecedented transformations: 1) a ligand type dependent, aromatic keto‐arylation that linked sensitive isocoumarin **13** to xanthone **14**, while also carrying the elusive C25‐oxygen for modular synthesis, validating our recently developed ligand type approach, 2) a catalytic, asymmetric Davis oxidation that selectively introduced the C26‐alcohol for subsequent chelate‐controlled reduction to the critical *cis*‐glycol, 3) a late stage aminolysis that enabled these challenging transformations and circumvented laborious protective group chemistry. These total syntheses also confirm the full architecture of this most potent class of polycyclic xanthone antibiotics.

Taken together, these methods may find broad application in the transformation of sensitive, advanced intermediates. In particular, this work highlights the power of ligand types as a rational design tool for complex cross‐couplings, thereby enabling powerful new strategies for sophisticated bond formation.

Ongoing studies focus on alternative amines for the divergent synthesis of uncharted polycyclic xanthones and novel activators for mild keto‐arylation of silyl enol‐ethers.

## Conflict of Interests

The authors declare no conflict of interest.

## Supporting information



Supporting Information

## Data Availability

The data that support the findings of this study are available in the Supporting Information of this article.
