# Supplementary material for: Ligand Type Guided Keto‐Arylation Enables Modular Total Synthesis of Polycyclic CBS Xanthones
Source: Angew Chem Int Ed Engl. 2025 Jul 22;64(37):e202513532. doi: 10.1002/anie.202513532 (PMC12416469; doi:10.1002/anie.202513532)
Supplement: Supplementary file 1 — Supporting Information [file ANIE-64-e202513532-s001.pdf]

Ligand Type Guided Keto-Arylation Enables Modular Total Synthesis of Polycyclic CBS Xanthones

Jonas W. Meringdal, Vivienne Prangenberg, Tim Treiber, Andreas J. Schneider, Leon Honsdorf, Dirk Menche*

Kekulé-Institut für Organische Chemie und Biochemie, Universität Bonn

Gerhard-Domagk-Str. 1, 53121 Bonn, Germany

E-mail: dirk.menche@uni-bonn.de

Contents

1 Materials and Methods **4**

2 Synthetic Procedures **6**

2.1 Synthesis of Isocoumarin **13** 6

2.2 Synthesis of Xanthone **14** 14

2.3 Synthesis of CBS100 (**5**) 24

2.4 Synthesis of CBS87 (**4**) 36

2.5 Synthesis of CBS72 (**3**) 48

2.6 Stereochemical Determination of Diol **12** 59

2.7 Synthesis of Reagents 71

3 Reaction Optimizations **72**

3.1 Mono-Enol Ether Stille (**17**) 72

3.2 Polar Addition (**27**) 76

3.3 Fragment Coupling (**10a**) 78

3.4 Enantioselective Ketone Reduction (**11a**) 90

3.5 Late Stage Aminolysis (**33**) 91

3.6 Catalytic Davis Oxidation (**42**) 92

4 NMR Comparisons **93**

4.1 Comparison of CBS100 (**5**) 93

4.2 Comparison of CBS87 (**4**) 95

4.3 Comparison of CBS72 (**3**) 97

5 Idealities **99**

6 Chromatograms **101**

6.1 Leading to Isocoumarin **13** 101

6.2 Leading to CBS100 (**5**) 102

6.3 Leading to CBS87 (**4**) 105

6.4 Leading to CBS72 (**3**) 108

6.5 Additional Chromatograms 111

6.6 Enantiomeric Excess 112

7 Experimental Spectra 118

7.1 Leading to Isocoumarin **13** 118

7.2 Leading to Xanthone **14**...149

7.3 Leading to CBS100 (**5**)...172

7.4 Leading to CBS87 (**4**)...212

7.5 Leading to SCB72 (**3**)...256

7.6 Additional Spectra 290

1 Materials and Methods

Reagents were purchased from commercial suppliers (Sigma-Aldrich, TCI, Acros, abcr, Carbolution, BLDPharm) in the highest purity grade available and used without further purification.

All reactions were performed using anhydrous solvents under an atmosphere of argon using standard Schlenk techniques unless stated otherwise. Solutions for cross-couplings were degassed by freeze-pump-thaw (3-4 times). The fragment coupling was carried out with degassed, anhydrous toluene from Sigma-Aldrich (900522). For filtering over pre-packed solid phase extraction (SPE), silica gel columns from Machery-Nagel were used (730071).

Temperatures above room temperature (rt) refer to the temperature of the oil bath pre-heated to the stated temperature. The following ice baths were used: ice/water (0 °C), freezer (-30 °C), MeCN/dry ice (-40 °C), acetone/dry ice (-78 °C), acetone/liquid nitrogen (-90 °C).

TLC monitoring was performed with silica gel 60 F254 precoated polyester sheets from Macherey-Nagel and visualized using UV light and staining with a solution of $KMnO_4$ (1.5 g $KMnO_4$, 10 g K_2CO_3, 1.25 mL 10% NaOH in 200 mL H_2O) and subsequent heating.

For column chromatography Silica Gel 60 (40-63 µm) from Merck was used. For preparative TLC 20×20 cm aluminum silica gel 60 F254 sheets from Merck were used. For preparative HPLC a Nucleodur 100-5 Phenyl-Hexyl 5 µm 250×16 mm, Nucleodur 100-5 Sphinx 250×8 mm or Nucleodur 100-5 C18 5 µm 250×8 mm from Machery-Nagel or a ChiralART Cellulose-SC 5 µm (250×20 mm) from YMC was used. For determination of the enantiomeric excess (ee), a Daicel Chiralpak IC-U 1.6 µm (3.0×100 mm) at 0.85 mL/min was used.

Solvents for chromatography were obtained as follows. Cyclohexane (cy) and ethyl acetate (EA) were distilled. Stabilizer free THF was obtained from a solvent purification system from MBraun. MeCN, *n*-hexane (H) and ethanol were bought in HPLC grade. H_2O was obtained from a PureLab® flex 3.

Nuclear magnetic resonance (NMR) spectra were recorded on Bruker spectrometers in deuterated solvents obtained from Deutero. Spectra were measured at 298 K and chemical shifts are reported in ppm relative to $(Me)_4Si$ (δ = 0.00 ppm) and were calibrated to the residual undeuterated solvent signal. qNMRs were measured with a d_1 delay of 30 s. The following

abbreviations were used: s (singlet), d (doublet), t (triplet), q (quartet), m (multiplet), b (broad). High-resolution mass spectra (HRMS) were recorded on a Daltonik micrOTOF-Q from Bruker, Orbitrap XL from Thermo Fisher Scientific or Apex IV FT-ICR Bruker Daltonics.

2 Synthetic Procedures

2.1 Synthesis of Isocoumarin **13**

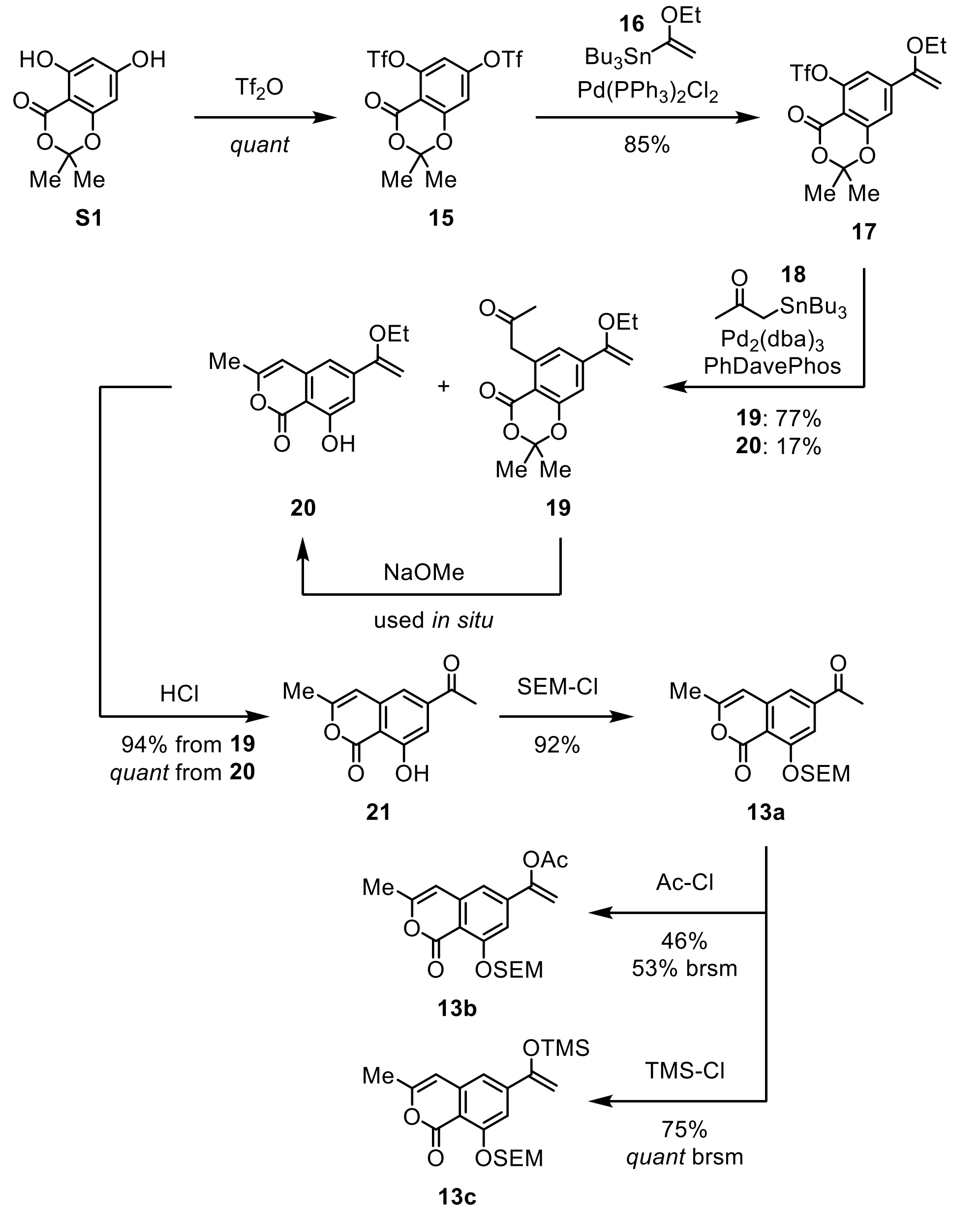

Triflate **15**

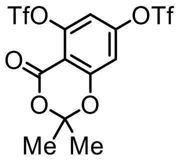

Procedure: To a solution of phenol **S1** (4.00 g, 19.0 mmol, 1.00 eq.) and anhydrous pyridine (10.0 mL, 124 mmol, 6.53 eq.) in DCM (100 mL) at -40 °C was added dropwise triflate anhydride (10.0 mL, 59.4 mmol, 3.13 eq.). The ice bath was removed and the reaction was stirred at room temperature for 20 min. Then, the reaction was cooled to -40 °C for 5 min and 2 M HCl (10.0 mL, 200 mmol, 10.5 eq.) was slowly added. The phases were separated, the water layer was extracted with DCM (4×100 mL) and each organic layer was filtered through the same silica plug (6×5 cm). The combined filtrates were concentrated under reduced pressure to yield **15** as a white solid in quantitative yield.

R*f* (cy/EA 2:1) 0.62; **^{1}H NMR** (400 MHz, CD_2Cl_2) δ 7.08 (d, *J* = 2.3 Hz, 1H), 6.96 (d, *J* = 2.4 Hz, 1H), 1.78 (s, 6H); **^{13}C NMR** (126 MHz, CD_2Cl_2) δ 158.8, 156.3, 153.7, 150.0, 119.1 (q, *J* = 321 Hz), 119.0 (q, *J* = 321 Hz), 111.8, 111.1, 108.7, 108.4, 25.7; **HRMS** (ESI+) 491.9852, calc. 491.9852 $[M+NH_4]^+$. Analytical data were in agreement with those previously reported.[24]

Enol ether **17**

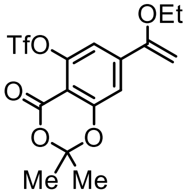

Procedure: To a mixture of triflate **15** (7.53 g, 19.0 mmol, 1.00 eq.) and **16** (13.5 mL, 40.0 mmol, 2.11 eq.) in DMF (50 mL) was added $Pd(PPh_3)_2Cl_2$ (333 mg, 474 µmol, 2.5 mol-%). The reaction was stirred at 42 °C for 70 min. Then, H_2O (200 mL) was added, the water layer was extracted with DCM (4×200 mL), each organic layer was filtered through the same silica plug (6×5 cm) and the combined filtrates were concentrated under reduced pressure. Column chromatography (SiO_2/K_2CO_3 10:1, cy/EA 10:1) yielded **17** as a white solid (6.43 g, 16.2 mmol, 85%). **17** was used directly in the next step to avoid decomposition.

R_f (cy/EA 4:1) 0.42; **^{1}H NMR** (400 MHz, CD_2Cl_2) δ 7.33 (d, *J* = 1.6 Hz, 1H), 7.24 (dd, *J* = 1.6, 0.7 Hz, 1H), 4.83 (d, *J* = 3.4 Hz, 1H), 4.46 (d, *J* = 3.4 Hz, 1H), 3.93 (q, *J* = 7.0 Hz, 2H), 1.75 (s, 6H), 1.41 (t, *J* = 7.0 Hz, 3H); **^{13}C NMR** (101 MHz, CD_2Cl_2) δ 157.7, 157.4, 156.8, 148.9, 145.4, 119.2 (q, *J* = 321 Hz), 114.6, 113.8, 107.7, 107.4, 87.0, 64.5, 25.7, 14.5; **HRMS** (ESI+) 397.0562, calc. 397.0563 $[M+H]^+$.

Keton **19** and Isocumarin **20**

Procedure: A solution of isopropenyl acetate (5.50 mL, 50.7 mmol, 3.13 eq.) and Bu_3SnOMe (12.0 mL, 41.6 mmol, 2.57 eq.) in toluene (20 mL) was stirred at 100 °C for 30 min, resulting in a solution of **18**. The solution was cooled to room temperature for 2 min with a water bath. PhDavePhos (618 mg, 1.62 mmol, 10 mol-%) and $Pd_2(dba)_3$ (443 mg, 484 µmol, 3.0 mol-%) were added, the solution was stirred for 5 min and added to a solution of enol ether **17** (6.43 g, 16.2 mmol, 1.00 eq.) in DMF (30 mL). The reaction was stirred at 110 °C for 30 min. Then, H_2O (200 mL) was added, the water layer was extracted with DCM (4×200 mL), each organic layer was filtered over the same silica plug (6×5 cm) and the combined filtrates were concentrated under reduced pressure. Column chromatography (SiO_2/K_2CO_3 10:1, cy/EA 9:1 → 3:1) yielded **19** as yellow solid (3.78 g, 12.4 mmol, 77%) and **20** as slightly yellow solid (682 mg, 2.78 mmol, 17%).

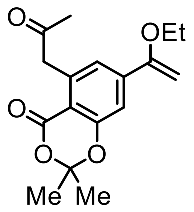

Ketone 19: $\mathbf{R}_f$ (cy/EA 3:1) 0.37; **^{1}H NMR** (400 MHz, CD_2Cl_2) δ 7.18 (d, *J* = 1.7 Hz, 1H), 7.11 (d, *J* = 1.7 Hz, 1H), 4.79 (d, *J* = 3.0 Hz, 1H), 4.36 (d, *J* = 3.0 Hz, 1H), 4.15 (s, 2H), 3.91 (q, *J* = 7.0 Hz, 2H), 2.25 (s, 3H), 1.70 (s, 6H), 1.41 (t, *J* = 7.1 Hz, 6H); **^{13}C NMR** (176 MHz, CD_2Cl_2) δ 205.1, 161.0, 158.3, 157.4, 143.7, 139.3, 123.5, 113.5, 112.8, 106.1, 85.5, 64.2, 49.6, 30.2, 25.8, 14.6; **HRMS** (ESI+) 305.1382, calc. 305.1384 $[M+H]^+$.

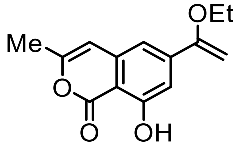

Isocumarin 20: $\mathbf{R}_f$ (cy/EA 3:1) 0.60; **^{1}H NMR** (400 MHz, CD_2Cl_2) δ 10.88 (s, 1H), 7.17 (d, *J* = 1.5 Hz, 1H), 7.11 (d, *J* = 1.5 Hz, 1H), 6.31 (d, *J* = 1.1 Hz, 1H), 4.82 (d, *J* = 3.0 Hz, 1H), 4.37 (d, *J* = 3.0 Hz, 1H), 3.92 (q, *J* = 7.0 Hz, 2H), 2.27 (d, *J* = 1.0 Hz, 3H), 1.42 (t, *J* = 7.0 Hz, 3H); **^{13}C NMR** (176 MHz, CD_2Cl_2) δ 166.9, 161.7, 158.7, 154.7, 145.7, 138.4, 112.5, 111.5, 105.7, 105.2, 85.7, 64.2, 19.6, 14.6; **HRMS** (ESI+) 247.0961, calc. 247.0965 $[M+H]^+$.

Acetophenone 21

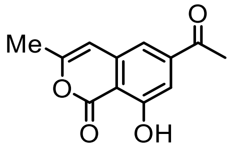

Procedure from 19: To a solution of ketone **19** (3.78 g, 12.4 mmol, 1.00 eq.) in MeOH (30 mL) was added NaOMe (5.4 M in MeOH, 2.50 mL, 13.5 mmol, 1.09 eq.). The reaction was stirred at 50 °C for 15 min. The oil bath was removed, 3 M HCl (40.0 mL, 120.0 mmol, 9.68 eq.) and DCM (100 mL) were added and the reaction was stirred at room temperature for 5 min. Then, H_2O (200 mL) was added and the phases were separated. The water layer was extracted with DCM (3×100 mL) and each organic layer was filtered through the same silica plug (6×5 cm). The initial colorless filtrate (50 mL) was discarded and product was eluted with DCM (500 mL) and Et_2O (200 mL). The combined filtrates were concentrated under reduced pressure to yield **21** as a yellow solid (2.53 g, 11.6 mmol, 94%).

Procedure from 20: Under air, to a solution of enol ether **20** (682 mg, 2.78 mmol, 1.00 eq.) in MeOH (10 mL) and DCM (10 mL) was added 3 M HCl (10.0 mL, 30.0 mmol, 10.8 eq.). The reaction was stirred at room temperature for 5 min. Then, H_2O (50 mL) was added, the water layer was extracted with DCM (4×20 mL) and each organic layer was filtered through the same silica plug (3×5 cm). The initial colorless filtrate (50 mL) was discarded and product was eluted with Et_2O (80 mL). The combined filtrates were concentrated under reduced pressure to yield **21** as a yellow solid in quantitative yield.

R_f (cy/EA 2:1) 0.45; **^{1}H NMR** (400 MHz, CD_2Cl_2) δ 10.98 (d, *J* = 0.6 Hz, 1H), 7.39 (d, *J* = 1.5 Hz, 1H), 7.36 (d, *J* = 1.5 Hz, 1H), 6.41 – 6.36 (m, 1H), 2.61 (d, *J* = 0.6 Hz, 4H), 2.38 – 2.25 (m, 4H); **^{13}C NMR** (126 MHz, CD_2Cl_2) δ 197.5, 166.7, 162.2, 155.5, 144.4, 138.9, 114.9, 114.0, 108.6, 105.1, 27.3, 19.6; **HRMS** (ESI-) 217.0511, calc. 217.0506 $[M-H]^-$.

SEM Ether **13a**

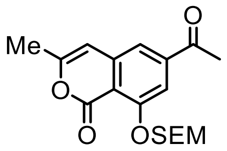

Procedure: To a mixture of acetophenone **21** (2.53 g, 11.6 mmol, 1.00 eq.) and DIPEA (10.0 mL, 57.4 mmol, 4.95 eq.) in DCE (40 mL) and DMF (10 mL) at -40 °C was added SEM-Cl (4.50 mL, 25.4 mmol, 2.19 eq.). The reaction was stirred at 90 °C for 45 min. Then, H_2O (100 mL) was added, the phases were separated and the water layer was extracted with DCM (3×100 mL). The combined organic layers were dried over $MgSO_4$, filtered and concentrated under reduced pressure. Column chromatography (SiO_2, cy/EA 4:1 → 1:1) yielded **13a** as a yellow solid (3.71 g, 10.7 mmol, 92%).

R*f* (cy/EA 2:1) 0.37; **^{1}H NMR** (500 MHz, CD_2Cl_2) δ 7.62 (d, *J* = 1.5 Hz, 1H), 7.46 (d, *J* = 1.5 Hz, 1H), 6.26 (d, *J* = 1.2 Hz, 1H), 5.43 (s, 2H), 3.92 – 3.75 (m, 2H), 2.62 (s, 3H), 2.24 (d, *J* = 0.9 Hz, 3H), 1.13 – 0.80 (m, 2H), -0.01 (s, 9H); **^{13}C NMR** (126 MHz, CD_2Cl_2) δ 197.4, 160.0, 158.8, 156.3, 142.6, 141.2, 118.2, 112.7, 112.3, 103.8, 94.1, 67.4, 27.2, 19.7, 18.3, -1.4; **HRMS** (ESI+) 371.1281, calc. 371.1285 $[M+Na]^+$.

Acetate Enol Ether **13b**

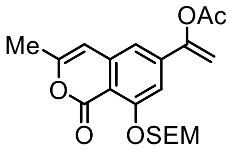

Procedure: To a mixture of acetophenone **13a** (190 mg, 545 µmol, 1.00 eq.) in THF (5 mL) at -90 °C was quickly added dropwise NaHMDS (1 M in THF, 550 µL, 550 µmol, 1.01 eq.) and Ac-Cl (1 M in DCM, 660 µL, 660 µmol, 1.21 eq.). The ice bath was removed and the reaction was stirred at room temperature for 60 min. Then, the reaction was added onto pH 7 buffer (10 mL), H_2O (5 mL) was added and the water layer was extracted with DCM (3×15 mL). The combined organic layers were dried over Na_2SO_4, filtered and concentrated under reduced pressure. Preparative HPLC (Phenyl-Hexyl, 15 mL/min, MeCN/H_2O 11:9) yielded **13b** as a slightly yellow solid (97.4 mg, 249 µmol, 46%, 53% brsm).

R_t (Phenyl-Hexyl, 15 mL/min, MeCN/H_2O 11:9) 21.23 min; **^{1}H NMR** (700 MHz, CD_2Cl_2) δ 7.25 (d, *J* = 1.7 Hz, 1H), 7.01 (d, *J* = 1.7 Hz, 1H), 6.17 (d, *J* = 1.2 Hz, 1H), 5.63 (d, *J* = 2.5 Hz, 1H), 5.39 (s, 2H), 5.20 (d, *J* = 2.5 Hz, 1H), 3.90 – 3.72 (m, 2H), 2.28 (s, 3H), 2.22 (d, *J* = 1.0 Hz, 3H), 0.97 (dd, *J* = 9.0, 7.7 Hz, 2H), 0.01 (s, 9H); **^{13}C NMR** (176 MHz, CD_2Cl_2) δ 169.2, 160.0, 158.9, 156.0, 152.0, 141.4, 141.1, 114.5, 110.5, 110.1, 105.7, 103.7, 94.3, 67.3, 21.1, 19.7, 18.4, -1.4. **13b** decomposed during HRMS.

TMS Enol Ether **13c**

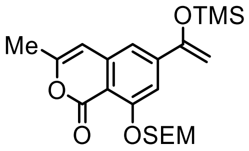

Procedure: To a mixture of acetophenone **13a** (1.39 g, 3.99 mmol, 1.00 eq.) in THF (40 mL) at -90 °C was quickly added dropwise NaHMDS (1 M in THF, 4.20 mL, 4.20 mmol, 1.05 eq.) and TMS-Cl (560 µL, 4.40 mmol, 1.10 eq.). The ice bath was removed and the reaction was stirred at room temperature for 30 min. Then, the reaction was added onto pH 7 buffer (100 mL), H_2O (100 mL) was added and the water layer was extracted with MTBE (3×200 mL). The combined organic layers were dried over Na_2SO_4, filtered and concentrated under reduced pressure. The crude product was quickly filtered over a silica plug (4×5 cm, P/EA cooled to -30 °C) as follows: the initial colorless filtrate was discarded (P/EA 19:1, 200 mL), the product was collected (P/EA 4:1, 400 mL) and finally the starting material was eluted (MTBE, 200 mL). Concentration under reduced pressure yielded **13c** as a slightly yellow solid (1.26 g, 3.00 mmol, 75%, quantitative brsm).

$\mathbf{R}_f$ (cy/EA 4:1) 0.41; $\mathbf{R}_t$ (Phenyl-Hexyl, MeCN/H_2O 7:3) 11.58 min; **^{1}H NMR** (700 MHz, CD_2Cl_2) δ 7.37 (d, *J* = 1.6 Hz, 1H), 7.15 (d, *J* = 1.6 Hz, 1H), 6.18 (d, *J* = 1.2 Hz, 1H), 5.38 (s, 2H), 5.08 (d, *J* = 2.2 Hz, 1H), 4.61 (d, *J* = 2.1 Hz, 1H), 3.95 – 3.67 (m, 2H), 2.22 (d, *J* = 1.0 Hz, 3H), 1.06 – 0.88 (m, 2H), 0.29 (s, 9H), 0.00 (s, 9H); **^{13}C NMR** (126 MHz, CD_2Cl_2) δ 159.6, 159.2, 155.4, 154.6, 144.7, 140.6, 115.0, 111.2, 109.6, 103.9, 94.6, 94.3, 67.2, 19.6, 18.4, 0.0, -1.4. **13c** decomposed during HRMS.

2.2 Synthesis of Xanthone **14**

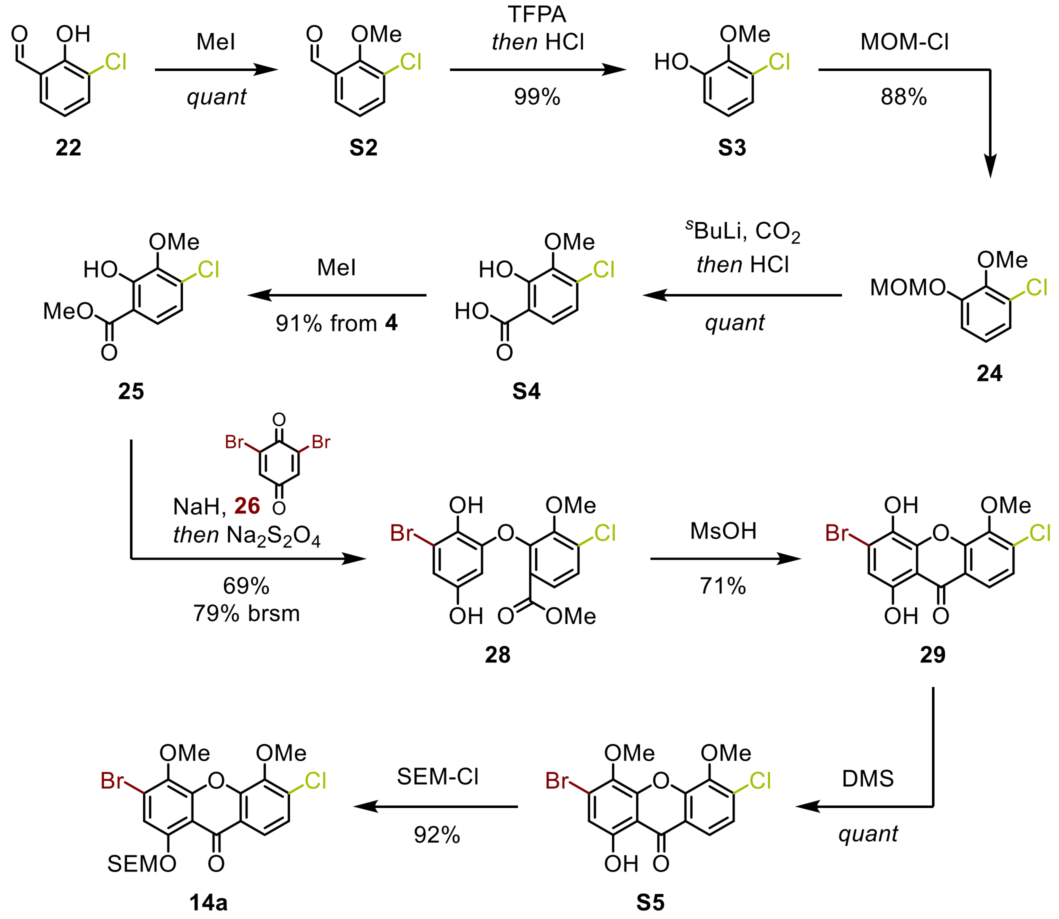

Anisole **S2**

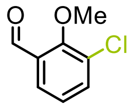

2

Procedure: To a mixture of phenol **22** (5.00 g, 31.9 mmol, 1.00 eq.) and K_2CO_3 (6.62 g, 47.9 mmol, 1.50 eq.) in acetone (60 mL) was added methyl iodide (10.0 mL, 160 mmol, 5.02 eq.) and the reaction was stirred at 50 °C for 3 h, after which the reaction turned from yellow into a colorless suspension. The crude product was filtered over a silica plug (3×5 cm) and the solvent was removed under reduced pressure to yield **S2** as a yellow oil in quantitative yield.

R*f* (cy/EA 9:1) 0.44; **^{1}H NMR** (500 MHz, CD_2Cl_2) δ 10.34 (s, 1H), 7.71 (d, *J* = 7.8 Hz, 1H), 7.63 (dd, *J* = 7.9, 1.8 Hz, 1H), 7.18 (t, *J* = 7.9 Hz, 1H), 3.98 (s, 3H); **^{13}C NMR** (126 MHz, CD_2Cl_2) δ 189.1, 159.3, 136.6, 131.3, 129.1, 127.3, 125.5, 63.4; **HRMS** (APCI) 171.0213, calc. 171.0207 $[M+H]^+$. Analytical data were in agreement with those previously reported.[33]

Phenol **S3**

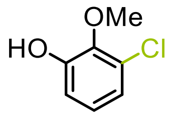

Procedure: To a solution of H_2O_2 (35% in H_2O, 3.00 mL, 34.8 mmol, 1.48 eq.) in DCM (4 mL) at 0 °C was added dropwise trifluoracetic anhydride (24.5 mL, 176 mmol, 7.49 eq.) over 30 min with a syringe pump and the solution was stirred at 0 °C for 30 min, resulting in a solution of trifluorperacetic acid (**23**). This solution was added dropwise at 0 °C over 30 min with a syringe pump to a vigorously stirred mixture of aldehyde **S2** (4.00 g, 23.5 mmol, 1.00 eq.) and KH_2PO_4 (63.7 g, 468 mmol, 19.9 eq.) in DCM (100 mL) and the reaction was stirred at 0 °C for 2 h. Then, saturated $NaHSO_3$ (100 mL) was added and the mixture was stirred at room temperature for 30 min. H_2O (200 mL) was added, the phases were separated and the aqueous layer was extracted with DCM (3×200 mL). The combined organic layers were dried over $MgSO_4$, filtered and most of the DCM was carefully removed under reduced pressure (40 °C, 600 mbar) to yield the crude formate as a yellow oil in quantitative yield. Under air, to a solution of the crude formate in MeOH (47 mL) was added concentrated HCl (2.00 mL, 24.0 mmol, 1.02 eq.) and the reaction was stirred at room temperature for 1 h. Then, the reaction was concentrated under reduced pressure, filtered over a silica plug (3×5 cm) and concentrated under reduced pressure to yield **S3** as a yellow oil (3.68 g, 23.2 mmol, 99%).

$\mathbf{R}_{\boldsymbol{f}}$ (cy/EA 9:1) 0.26; **^{1}H NMR** (499 MHz, CD_2Cl_2) δ 6.95 (t, *J* = 8.0 Hz, 1H), 6.90 (dd, *J* = 8.2, 1.8 Hz, 1H), 6.87 (dd, *J* = 7.9, 1.8 Hz, 1H), 5.89 (bs, 1H), 3.91 (s, 3H); **^{13}C NMR** (126 MHz, CD_2Cl_2) δ 150.7, 143.9, 127.3, 125.5, 122.0, 114.6, 61.5; **HRMS** (ESI-) 157.0058, calc. 157.0062 $[M-H]^-$. Analytical data were in agreement with those previously reported.[33]

Formate of Phenol **S3**

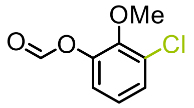

$\mathbf{R}_{\boldsymbol{f}}$ (cy/EA 9:1) 0.38; **^{1}H NMR** (500 MHz, CD_2Cl_2) δ 8.29 (d, *J* = 1.2 Hz, 1H), 7.33 (dt, *J* = 7.6, 1.7 Hz, 1H), 7.12 – 7.05 (m, 2H), 3.85 (d, *J* = 1.3 Hz, 3H); **^{13}C NMR** (126 MHz, CD_2Cl_2) δ 159.1, 148.7, 144.5, 129.3, 128.8, 125.1, 122.1, 61.4; **HRMS** (ESI-) 185.0010, calc. 185.0011 $[M]^-$.

MOM Ether **24**

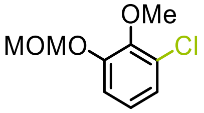

Procedure: To a solution of phenol **S3** (2.74 g, 17.3 mmol, 1.00 eq.) and DIPEA (31.0 mL, 177 mmol, 10.2 eq.) in DCM (100 mL) was added MOM-Cl (4.00 mL, 52.7 mmol, 3.05 eq.) and the reaction was stirred at 40 °C for 20 h. The reaction was quenched with AcOH (25.0 mL, 437 mmol, 25.3 eq.) and H_2O (100 mL) was added. The phases were separated and the aqueous layer was extracted with DCM (3×100 mL). The combined organic layers were dried over $MgSO_4$ and the solvent was removed under reduced pressure. Column chromatography (cy/EA 9:1) yielded **24** as a yellow oil (3.07 g, 15.2 mmol, 88%). For the next step, a solution of **24** in THF (50 mL) was stored over 3 Å mole sieve for at least 16 h.

R_f (cy/EA 9:1) 0.40; **^{1}H NMR** (500 MHz, CD_2Cl_2) δ 7.09 – 7.01 (m, 2H), 6.97 (td, *J* = 8.1, 1.1 Hz, 1H), 5.21 (d, *J* = 1.2 Hz, 2H), 3.86 (d, *J* = 1.2 Hz, 3H), 3.50 (d, *J* = 1.2 Hz, 3H); **^{13}C NMR** (126 MHz, CD_2Cl_2) δ 152.0, 146.9, 128.7, 124.8, 123.6, 115.8, 95.8, 61.0, 56.6; **HRMS** (ESI+) 203.0470, calc. 203.0469 $[M+H]^+$.

Acid **S4**

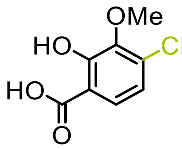

Procedure: To a solution of MOM ether **24** (2.87 g, 14.2 mmol, 1.00 eq.) in THF (50 mL) at -78 °C was added dropwise ^{s}BuLi (1.60 M, 13.5 mL, 17.6 mmol, 1.24 eq.) with a syringe pump over 15 min and the solution was stirred for 30 min. CO_2 (g) passed through Drierite™ was added for 30 min. The ice bath was removed and the reaction was stirred at room temperature for 30 min. Then, half saturated $NaHCO_3$ solution (140 mL) was added and the aqueous layer was washed with DCM (2×150 mL). Concentrated HCl (150 mL) and Et_2O (100 mL) were added and the emulsion was stirred at room temperature for 30 min. The phases were separated and the aqueous layer was extracted with Et_2O (3×250 mL). The combined Et_2O layers were dried over Na_2SO_4, filtered and the solvent was removed under reduced pressure to yield **S4** as a white solid in quantitative yield. **R_f** (cy/EA 4:1) 0.33; **^{1}H NMR** (499 MHz, Acetone) δ 11.47 (bs, 1H), 7.64 (d, J = 8.7 Hz, 1H), 7.32 (bs, 1H), 7.00 (d, J = 8.7 Hz, 1H), 3.89 (s, 3H); **^{13}C NMR** (126 MHz, Acetone) δ 172.3, 157.5, 145.7, 134.8, 126.3, 120.6, 113.4, 60.6; **HRMS** (ESI-) 200.9960, calc. 200.9960 [M-H]$^{-}$. Analytical data were in agreement with those previously reported.[25]

Ester **25**

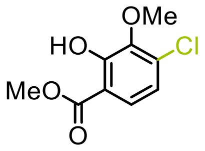

Procedure: To a solution of acid **S4** (3.32 g, 16.4 mmol, 1.00 eq.) and $NaHCO_3$ (1.51 g, 18.0 mmol, 1.10 eq.) in DMF (30 mL) was added methyl iodide (1.40 mL, 22.5 mmol, 1.37 eq.) and the reaction was stirred at room temperature for 24 h. Then, saturated $NaHCO_3$ solution (20 mL), brine (150 mL) and H_2O (150 mL) were added and the water layer was extracted with Et_2O (3×200 mL). The combined organic layers were dried over $MgSO_4$, filtered and concentrated under reduced pressure to yield **25** as a yellow oil (3.22 g, 14.9 mmol, 91%).

R_f (cy/EA 4:1) 0.54; **^{1}H NMR** (700 MHz, CD_2Cl_2) δ 11.04 (s, 1H), 7.54 (d, *J* = 8.7 Hz, 1H), 6.91 (d, *J* = 8.7 Hz, 1H), 3.95 (s, 3H), 3.90 (s, 3H); **^{13}C NMR** (176 MHz, CD_2Cl_2) δ 170.9, 156.8, 145.4, 134.9, 125.4, 120.5, 113.0, 60.9, 53.1; **HRMS** (ESI+) 239.0079, calc. 239.0082 $[M+Na]^+$. Analytical data were in agreement with those previously reported.[26]

Hydroquinone **28**

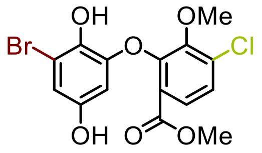

Procedure: To a solution of ester **25** (670 mg, 3.09 mmol, 1.00 eq.) in DMF (8 mL) at 0 °C was added NaH (60% in mineral oil, 124 mg, 3.10 mmol, 1.00 eq.) and the mixture was stirred at 0 °C for 15 min. **26** (986 mg, 3.71 mmol, 1.20 eq.) was added and the reaction was stirred at 40 °C for 90 min. Then, Et_2O (60 mL), $Na_2S_2O_4$ (10.8 g, 62.0 mmol, 20.1 eq.) and H_2O (62 mL) were added and the reduction was stirred at room temperature for 15 min. The phases were separated and the aqueous layer was extracted with EA (3×60 mL). The combined organic layers were dried over Na_2SO_4, filtered and concentrated under reduced pressure. Column chromatography (SiO_2, cy/EA 4:1 → 2:1) yielded **28** as a white solid (862 mg, 2.14 mmol, 69%, 79% brsm).

R_f (cy/EA 2:1) 0.38; **^{1}H NMR** (499 MHz, Acetone) δ 8.05 (bs, 1H), 8.05 (bs, 1H), 7.68 (d, *J* = 8.6 Hz, 1H), 7.51 (d, *J* = 8.6 Hz, 1H), 6.70 (d, *J* = 2.8 Hz, 1H), 5.99 (d, *J* = 2.7 Hz, 1H), 3.83 (s, 3H), 3.72 (s, 3H); **^{13}C NMR** (126 MHz, Acetone) δ 165.2, 151.2, 151.2, 149.3, 148.2, 138.4, 133.8, 127.8, 127.6, 126.2, 113.4, 110.4, 102.9, 61.8, 52.7; **HRMS** (ESI+) 424.9404, calc. 424.9398 $[M+Na]^+$. Analytical data were in agreement with those previously reported.[26]

Xanthone **29**

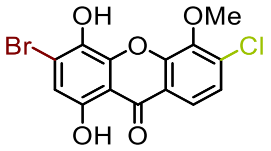

Procedure: A mixture of hydroquinone **28** (464 mg, 1.15 mmol, 1.00 eq.) and MsOH (10.0 mL, 154 mmol, 134 eq.) was stirred at 70 °C for 7 h. The reaction was quenched by dropwise addition on to sodium citrate dibasic sesquihydrate (50.0 g, 190 mmol, 165 eq.). The solid was taken up in H_2O (100 mL) and the aqueous phase was extracted with EA (3×50 mL). The combined organic layers were dried over Na_2SO_4, filtered and concentrated under reduced pressure. Recrystallization from EtOH and column chromatography of the concentrated mother liquid (SiO_2, cy/EA 13:2) yielded **29** as a yellow solid (305 mg, 821 µmol, 71%).

R_f (cy/EA 4:1) 0.38; **^{1}H NMR** (700 MHz, Acetone) δ 11.92 (s, 1H), 7.97 (d, *J* = 8.7 Hz, 1H), 7.56 (d, *J* = 8.7 Hz, 1H), 7.04 (s, 1H), 4.11 (s, 3H); **^{13}C NMR** (176 MHz, Acetone) δ 182.3, 154.7, 151.3, 146.3, 145.8, 136.3, 135.4, 126.6, 121.7, 121.7, 121.1, 114.2, 109.3, 62.1; **HRMS** (EI) 369.9243, calc. 369.9244 $[M]^+$. Analytical data were in agreement with those previously reported.[26]

Anisole **S5**

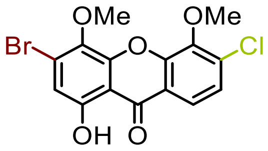

Procedure: To a mixture of xanthone **29** (743 mg, 2.00 mmol, 1.00 eq.) and K_2CO_3 (829 mg, 6.00 mmol, 3.00 eq.) in acetone (15 mL) and DMF (3 mL) was added DMS (220 µL, 2.31 mmol, 1.16 eq.) and the reaction was stirred at 55 °C for 5 h. Then, the reaction was concentrated under reduced pressure. H_2O (40 mL) and 1 M HCl (20.0 mL, 20.0 mmol, 10.0 eq.) were added, the water layer was extracted with 2-Me-THF (3×60 mL) and each organic layer was filtered over the same silica plug (3×5 cm). The combined filtrates were concentrated under reduced pressure to yield **S5** as a yellow solid in quantitative yield.

R*f* (cy/EA 4:1) 0.61; **^{1}H NMR** (500 MHz, Acetone) δ 12.25 (s, 1H), 7.98 (d, *J* = 8.7 Hz, 1H), 7.58 (d, *J* = 8.7 Hz, 1H), 7.07 (s, 1H), 4.19 (s, 3H), 4.05 (s, 3H); **^{13}C NMR** (126 MHz, Acetone) δ 182.3, 158.0, 151.3, 150.1, 146.3, 139.0, 135.5, 127.0, 126.7, 121.7, 121.6, 114.5, 109.6, 62.2, 61.9; **HRMS** (EI) 383.9399, calc. 383.9400 $[M]^+$.

SEM Protected **S5**

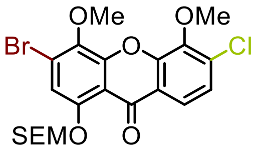

Procedure: To a mixture of anisole **S5** (771 mg, 2.00 mmol, 1.00 eq.) and K_2CO_3 (2.76 g, 20.0 mmol, 10.0 eq.) in acetone (50 mL) was added a SEM-Cl (1.77 mL, 10.0 mmol, 5.00 eq.) and the reaction was heated to reflux for 16 h. Then, the reaction was filtered over a silica plug (3×5 cm), the product was eluted with 2-Me-THF (100 mL) and the combined organic layers were concentrated under reduced pressure. Column chromatography (SiO_2, cy/THF 9:1 → 4:1) yielded **14a** as an off-white solid (914 mg, 1.77 mmol, 89%).

R_f (cy/EA 9:1) 0.26; **R_t** (Phenyl-Hexyl, MeCN/H_2O 7:3) 13.08 min; **^{1}H NMR** (400 MHz, CD_2Cl_2) δ 7.89 (d, *J* = 8.7 Hz, 1H), 7.37 (d, *J* = 8.7 Hz, 1H), 7.30 (s, 1H), 5.35 (s, 2H), 4.13 (s, 3H), 4.04 (s, 3H), 3.90 – 3.80 (m, 2H), 1.02 – 0.93 (m, 2H), 0.01 (s, 9H); **^{13}C NMR** (176 MHz, CD_2Cl_2) δ 174.9, 154.1, 151.4, 149.4, 145.3, 141.3, 133.8, 125.7, 124.0, 123.3, 121.9, 115.1, 113.8, 94.9, 67.4, 62.0, 61.8, 18.4, -1.4; **HRMS** (ESI+) 537.0105, calc. 537.0106 $[M+Na]^+$.

2.3 Synthesis of CBS100 (5)

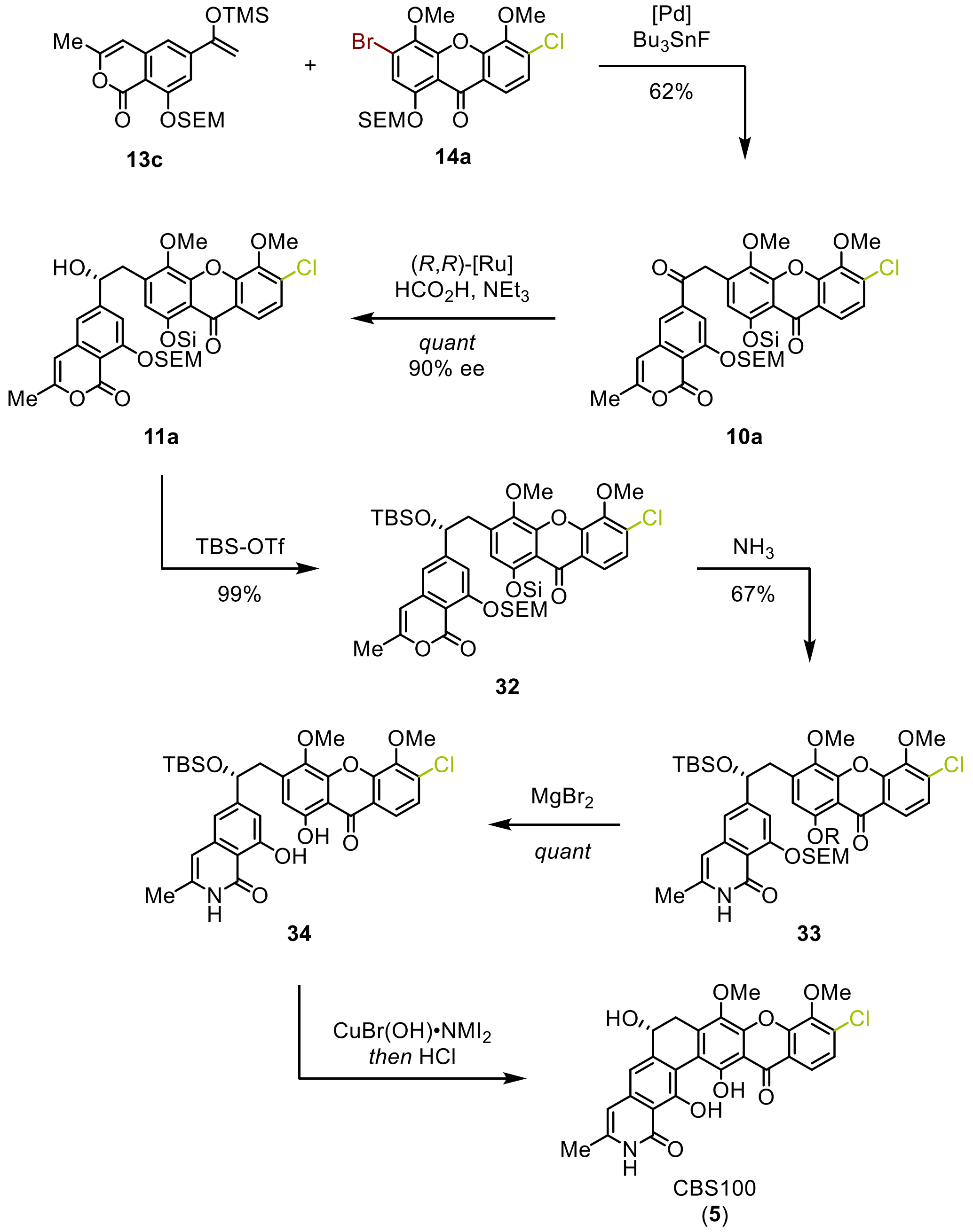

Coupled **10a**

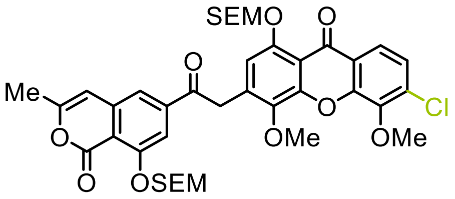

Procedure: In a glove box, a suspension of Pd(P^{t}Bu$_3$)$_2$ (19.0 mg, 37.2 µmol, 6.5 mol-%) and Pd$_2$(dba)$_3$ (15.5 mg, 16.9 µmol, 3.0 mol-%) in toluene (6 mL) was vigorously stirred at room temperature for 30 min and the supernatant (5.5 mL) was added to a mixture of bromide **14a** (294 mg, 572 µmol, 1.00 eq.), enol **13c** (600 mg, 1.43 mmol, 2.50 eq.) and Bu$_3$SnF (442 mg, 1.43 mmol, 2.50 eq.) in toluene (2 mL). The reaction was tightly sealed and stirred outside the glovebox at 103 °C for 30 min. Then, the reaction was cooled to -40 °C, quickly filtered over a silica plug (3×5 cm, MTBE cooled to -30 °C) and the filtrate was concentrated under reduced pressure. Preparative HPLC (Phenyl-Hexyl, 15 mL/min, MeCN/H$_2$O 7:3 20 min → MeCN 5 min) yielded **10a** as a colourless solid (278 mg, 355 µmol, 62%, 42% brsm **13c**).

R*f* (cy/EA 2:1) 0.28; **R*t*** (Phenyl-Hexyl, MeCN/H$_2$O 7:3) 15.95 min; **^{1}H NMR** (700 MHz, CD$_2$Cl$_2$) δ 7.91 (d, *J* = 8.7 Hz, 1H), 7.72 (d, *J* = 1.5 Hz, 1H), 7.58 (d, *J* = 1.5 Hz, 1H), 7.36 (d, *J* = 8.7 Hz, 1H), 6.94 (s, 1H), 6.29 (d, *J* = 1.3 Hz, 1H), 5.46 (s, 2H), 5.34 (s, 2H), 4.45 (s, 2H), 4.09 (s, 3H), 3.99 (s, 3H), 3.88 – 3.80 (m, 4H), 2.26 (d, *J* = 1.0 Hz, 3H), 0.98 – 0.92 (m, 4H), -0.01 (s, 9H), -0.01 (s, 9H); **^{13}C NMR** (176 MHz, CD$_2$Cl$_2$) δ 195.9, 175.1, 160.1, 158.7, 156.5, 153.6, 150.9, 149.5, 145.3, 142.0, 141.7, 141.3, 135.2, 133.7, 125.4, 123.3, 122.0, 118.1, 113.8, 113.3, 112.9, 112.4, 103.8, 94.9, 94.1, 67.5, 67.2, 61.8, 61.8, 41.5, 19.7, 18.3, 18.3, -1.4, -1.4; **HRMS** (ESI+) 805.2240, calc. 805.2238 [M+Na]$^+$.

Side Product of Coupled **10a**

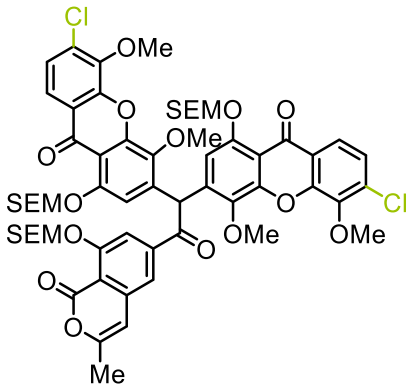

R_t (Phenyl-Hexyl, MeCN/H_2O 7:3) 20.25 min; **^{1}H NMR** (700 MHz, CD_2Cl_2) δ 7.91 (d, *J* = 8.7 Hz, 2H), 7.76 (d, *J* = 1.5 Hz, 1H), 7.58 (d, *J* = 1.5 Hz, 1H), 7.37 (d, *J* = 8.7 Hz, 2H), 6.89 (s, 1H), 6.83 (s, 2H), 6.24 (d, *J* = 1.3 Hz, 1H), 5.41 (s, 2H), 5.23 (s, 4H), 4.09 (s, 6H), 3.99 (s, 6H), 3.81 – 3.74 (m, 2H), 3.74 – 3.65 (m, 4H), 2.21 (s, 3H), 0.89 – 0.80 (m, 2H), 0.80 – 0.70 (m, 4H), -0.06 (s, 9H), -0.11 (s, 18H); **^{13}C NMR** (176 MHz, CD_2Cl_2) δ 196.4, 175.0, 160.0, 158.6, 156.4, 153.8, 151.0, 149.5, 145.3, 141.6, 141.2, 141.1, 137.2, 133.9, 125.6, 123.3, 122.1, 118.2, 114.1, 112.9, 112.6, 112.3, 103.8, 95.0, 93.9, 67.4, 67.0, 61.9, 61.9, 48.8, 19.7, 18.3, 18.3, -1.5, -1.5, -1.5; **HRMS** (ESI+) 1239.3191, calc. 1239.3190 [M+Na].

Alcohol **11a**

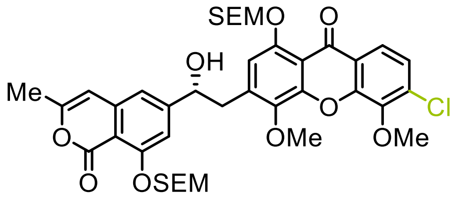

Procedure: To a solution of ketone **10a** (56.0 mg, 71.5 µmol, 1.00 eq.), HCO_2H (16.2 µL, 427 µmol, 5.97 eq.) and NEt_3 (99.7 µL, 715 µmol, 10.0 eq.) in DCM (0.7 mL) was added RuCl(*p*-cymene)[(*R*,*R*-Ts-DPEN) (9.75 mg, 15.3 µmol, 21 mol-%) and the reaction was stirred at room temperature for 1 h. Then, the reaction was filtered through a SPE silica plug (MTBE). Concentration under reduced pressure yielded sufficiently pure **11a** as a brown solid in quantitative yield (90% ee determined by chiral HPLC).

R*f* (cy/EA 1:1) 0.54; **R*t*** (Chiralpak, H/EtOH 9:1) 6.75 min; $[\boldsymbol{\alpha}]_{\mathbf{D}}^{\mathbf{20}}$ (c=1.00, DCM) -2.0°; **^{1}H NMR** (700 MHz, CD_2Cl_2) δ 7.90 (d, *J* = 8.6 Hz, 1H), 7.35 (d, *J* = 8.6 Hz, 1H), 7.19 (d, *J* = 1.5 Hz, 1H), 6.97 (d, *J* = 1.6 Hz, 1H), 6.90 (s, 1H), 6.15 (d, *J* = 1.2 Hz, 1H), 5.37 (s, 2H), 5.28 (s, 2H), 5.06 (dt, *J* = 8.2, 3.9 Hz, 1H), 4.11 (s, 3H), 4.03 (s, 3H), 3.83 (dt, *J* = 9.3, 7.4 Hz, 4H), 3.20 (dd, *J* = 13.7, 4.3 Hz, 1H), 3.12 (dd, *J* = 13.7, 8.7 Hz, 1H), 2.90 (s, 1H), 2.21 (d, *J* = 1.0 Hz, 3H), 1.01 – 0.88 (m, 4H), 0.01 (s, 9H), -0.00 (s, 9H); **^{13}C NMR** (176 MHz, CD_2Cl_2) δ 175.1, 159.9, 159.2, 155.6, 153.5, 152.6, 150.9, 149.5, 145.3, 141.7, 140.9, 138.6, 133.6, 125.4, 123.3, 122.0, 115.3, 113.4, 111.4, 109.1, 103.8, 94.8, 94.1, 74.1, 67.3, 67.2, 61.9, 61.9, 40.9, 19.7, 18.3, 18.3, -1.3, -1.4; **HRMS** (ESI+) 805.2240, calc. 805.2238 $[M+Na]^{+}$.

Mosher of Alcohol **11a**

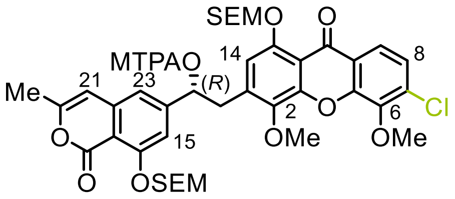

Procedure: To a solution of alcohol **11a** (1.60 mg, 2.04 µmol, 1.00 eq.) and pyridine (3 drops) in DCM (0.2 mL) was added MTPA-Cl (2 drops) and the reaction was stirred at room temperature for 16 h. Then, H_2O (1 mL) was added and the water layer was extracted with EA (3×1 mL). The combined organic layers were dried over Na_2SO_4, filtered and concentrated under reduced pressure. Preparative TLC (cy/EA 2:1) yielded sufficiently pure major diastereomer as a white solid in quantitative yield. Mosher ester analysis assigned the alcohol to be (*R*)-**11a**.

(*S*)-Ester: $[\alpha]_D^{20}$ (c=0.38, DCM) -18.7°; **^{1}H NMR** (700 MHz, CD_2Cl_2) δ 7.93 (d, *J* = 8.6 Hz, 1H), 7.37 (d, *J* = 8.7 Hz, 1H), 7.35 – 7.30 (m, 1H), 7.26 – 7.17 (m, 4H), 7.08 (d, *J* = 1.5 Hz, 1H), 6.94 (s, 1H), 6.76 (d, *J* = 1.5 Hz, 1H), 6.24 (dd, *J* = 9.7, 4.7 Hz, 1H), 6.06 (s, 1H), 5.28 – 5.21 (m, 4H), 4.12 (s, 3H), 4.00 (s, 3H), 3.86 – 3.77 (m, 4H), 3.35 (dd, *J* = 14.2, 9.7 Hz, 1H), 3.31 (s, 3H), 3.28 (dd, *J* = 14.2, 4.7 Hz, 1H), 2.23 (s, 3H), 0.97 – 0.92 (m, 4H), 0.01 (s, 9H), -0.00 (s, 9H); **^{13}C NMR** (176 MHz, CD_2Cl_2) δ 175.1, 165.9, 160.1, 158.9, 156.1, 153.6, 151.0, 149.6, 147.0, 145.3, 141.9, 141.2, 136.6, 133.8, 132.2, 130.1, 128.7, 127.5, 125.5, 123.3, 122.1, 115.7, 113.7, 112.8, 111.5, 109.9, 103.6, 94.8, 94.1, 84.7, 77.8, 67.4, 67.3, 61.9, 61.9, 55.8, 37.9, 19.7, 18.3, -1.3, -1.4; **HRMS** (ESI+) 1023.2797, calc. 1023.2792 $[M+Na]^+$. **^{13}C**F_3 not observed due to low resolution after quadrupole splitting.

(*R*)-Ester: $[\alpha]_D^{20}$ (c=0.03, DCM) 0.0°; **^{1}H NMR** (700 MHz, CD_2Cl_2) δ 7.94 (d, *J* = 8.6 Hz, 1H), 7.38 (d, *J* = 8.7 Hz, 1H), 7.25 (d, *J* = 1.5 Hz, 1H), 7.20 (d, *J* = 7.7 Hz, 2H), 7.15 – 7.07 (m, 3H), 7.00 (d, *J* = 1.5 Hz, 1H), 6.84 (s, 1H), 6.32 (dd, *J* = 10.0, 4.7 Hz, 1H), 6.18 (s, 1H), 5.41 (d, *J* = 7.1 Hz, 1H), 5.35 (d, *J* = 7.1 Hz, 1H), 5.24 (d, *J* = 7.1 Hz, 1H), 5.22 (d, *J* = 7.1 Hz, 1H), 4.10 (s, 3H), 3.91 (s, 3H), 3.88 – 3.79 (m, 4H), 3.45 – 3.37 (m, 4H), 3.17 (dd, *J* = 14.1, 4.7 Hz, 1H), 2.23 (s, 3H), 1.02 – 0.92 (m, 4H), 0.01 (s, 9H), 0.01 (s, 9H); **^{13}C NMR** (176 MHz, CD_2Cl_2) δ 175.1, 166.1, 160.3, 158.9, 156.2, 153.5, 150.8, 149.5, 146.8, 145.3, 141.7, 141.3, 136.4, 133.7, 132.3, 129.7, 128.5, 127.3, 125.5, 123.3, 122.0, 116.3, 113.6, 112.3, 111.9, 110.1, 103.6, 94.7, 94.2, 85.0, 77.3, 67.4, 67.2, 61.9, 61.7, 56.1, 37.3, 19.7, 18.4, 18.4, -1.4, -1.4; **HRMS** (ESI+) 1023.2797, calc. 1023.2792 $[M+Na]^+$. **^{13}C**F_3 not observed due to low resolution after quadrupole splitting.

hydrogen	(*S*)-ester [ppm]	(*R*)-ester [ppm]	$\Delta\delta^{SR}$ [ppm]
H-14	6.94	6.84	0.10
C2-OMe	4.00	3.91	0.09
C6-OMe	4.12	4.10	0.02
H-8	7.37	7.38	-0.01
H-21	6.06	6.18	-0.12
H-15	7.08	7.25	-0.17
H-23	6.76	7.00	-0.24

TBS Ether **32**

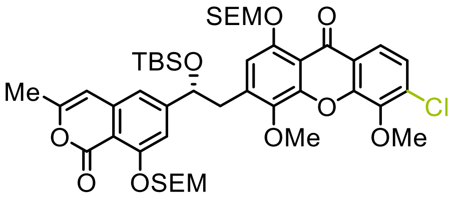

Procedure: To a solution of alcohol **aa1** (76.1 mg, 96.9 µmol, 1.00 eq.) and lutidine (56.3 µL, 485 µmol, 5.00 eq.) in DCM (1 mL) was added dropwise TBSOTf (44.5 µL, 194 µmol, 2.03 eq.) and the reaction was stirred at room temperature for 1 h. Then, H_2O (1.5 mL) was added, the phases were separated and the water layer was extracted with DCM (3×1.5 mL). The combined organic layers were dried over Na_2SO_4, filtered and concentrated under reduced pressure. Column chromatography (cy/EA 4:1) yielded **32** as a yellow oil (86.6 mg, 96.2 µmol, 99%).

$\mathbf{R}_f$ (cy/EA 2:1) 0.60; $[\boldsymbol{\alpha}]_{\mathbf{D}}^{\mathbf{20}}$ (c=1.00, DCM) -26.0°; ^{1}**H NMR** (700 MHz, CD_2Cl_2) δ 7.91 (d, *J* = 8.6 Hz, 1H), 7.35 (d, *J* = 8.6 Hz, 1H), 7.21 (d, *J* = 1.4 Hz, 1H), 6.90 (d, *J* = 1.4 Hz, 1H), 6.88 (s, 1H), 6.15 (d, *J* = 1.2 Hz, 1H), 5.37 (d, *J* = 7.0 Hz, 1H), 5.34 (d, *J* = 7.0 Hz, 1H), 5.31 (d, *J* = 7.4 Hz, 1H), 5.25 (d, *J* = 6.9 Hz, 1H), 5.04 (dd, *J* = 7.7, 5.3 Hz, 1H), 4.12 (s, 3H), 4.01 (s, 3H), 3.86 – 3.79 (m, 4H), 3.08 – 3.05 (m, 2H), 2.21 (d, *J* = 0.9 Hz, 3H), 0.99 – 0.92 (m, 4H), 0.80 (s, 9H), 0.01 (s, 9H), 0.01 (s, 9H), -0.19 (s, 3H), -0.22 (s, 3H); ^{13}**C NMR** (176 MHz, CD_2Cl_2) δ 175.2, 159.9, 159.3, 155.5, 153.6, 153.2, 150.9, 149.6, 145.3, 141.9, 140.7, 139.3, 133.5, 125.3, 123.3, 122.0, 115.4, 114.1, 113.2, 111.6, 109.0, 103.8, 94.9, 94.1, 75.0, 67.2, 67.1, 61.8, 61.7, 42.5, 25.9, 19.6, 18.4, 18.4, -1.3, -1.4, -4.9, -5.2; **HRMS** (ESI+) 921.3252, calc. 921.3259 $[M+Na]^{+}$.

Amide **33**

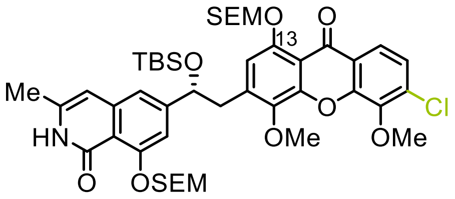

Procedure: A solution of lactone **32** (73.2 mg, 81.4 µmol, 1.00 eq.) in methoxyethanol (0.8 mL) at 0 °C was purged with NH_3 gas for 15 min. The reaction was tightly sealed and heated to 140 °C for 1 h. Then, saturated citric acid (5 mL) was added and the water layer was extracted with EA (3×5 mL). The combined organic layers were dried over Na_2SO_4, filtered and concentrated under reduced pressure. Column chromatography (cy/EA 1:3) yielded a sufficiently pure but inseparable 7:3 mixture of **33** and C13-OH-**33** as a yellow solid (46.8 mg, 54.4 µmol, 67%). As reference for analytics, a screening sample was purified yielding a small amount of semi-pure **33**.

R*f* (cy/EA 1:3) 0.28; $[\alpha]_D^{20}$ (c=1.00, DCM) -22.0°; **^{1}H NMR** (400 MHz, Acetone) δ 9.83 (bs, 1H), 7.90 (d, *J* = 8.7 Hz, 1H), 7.46 (d, *J* = 8.7 Hz, 1H), 7.13 (d, *J* = 1.7 Hz, 1H), 7.11 (d, *J* = 1.7 Hz, 1H), 6.98 (s, 1H), 6.17 (bs, 1H), 5.33 – 5.23 (m, 3H), 5.18 (dd, *J* = 7.9, 5.1 Hz, 1H), 4.16 (s, 3H), 4.07 (s, 3H), 3.91 – 3.81 (m, 4H), 3.18 (dd, *J* = 13.1, 7.9 Hz, 1H), 3.13 (dd, *J* = 13.0, 5.2 Hz, 1H), 2.25 (d, *J* = 1.0 Hz, 3H), 1.04 – 0.91 (m, 4H), 0.84 (s, 9H), 0.03 (s, 9H), 0.02 (s, 9H), -0.13 (s, 3H), -0.14 (s, 3H); **HRMS** (ESI+) 898.3603, calc. 898.3599[M+H]$^+$.

Bisphenol **34**

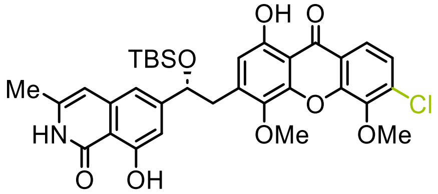

Procedure: To a solution of amide mixture **33** (32.8 mg, 38.2 µmol, 1.00 eq.) in Et_2O (0.5 mL) was added $MgBr_2 \cdot Et_2O$ (94.3 mg, 365 µmol, 9.55 eq.) and the reaction was stirred at room temperature for 30 min. Then, the reaction was filtered through a SPE silica plug (EA). Concentration under reduced pressure yielded **34** as a yellow solid in quantitative yield, which could be used directly and may be purified by preparative HPLC (Phenyl-Hexyl, 15 mL/min, MeCN/H_2O 8:2).

$\mathbf{R}_f$ (cy/EA 1:1) 0.35; $\mathbf{R}_t$ (Phenyl-Hexyl, MeCN/H_2O 8:2) 18.25 min; $[\alpha]_D^{20}$ (c=1.00, DCM) -19.0°; **^{1}H NMR** (700 MHz, Acetone) δ 12.85 (s, 1H), 12.04 (s, 1H), 10.41 (bs, 1H), 7.98 (d, *J* = 8.6 Hz, 1H), 7.55 (d, *J* = 8.6 Hz, 1H), 7.01 (d, *J* = 1.5 Hz, 1H), 6.83 (d, *J* = 1.6 Hz, 1H), 6.77 (s, 1H), 6.39 (s, 1H), 5.13 (dd, *J* = 8.6, 4.6 Hz, 1H), 4.19 (s, 3H), 4.09 (s, 3H), 3.15 (dd, *J* = 13.2, 4.6 Hz, 1H), 3.11 (dd, *J* = 13.2, 8.6 Hz, 1H), 2.32 (s, 3H), 0.82 (s, 9H), -0.18 (s, 3H), -0.18 (s, 3H); **^{13}C NMR** (176 MHz, Acetone) δ 182.2, 167.6, 162.7, 157.1, 153.4, 151.4, 149.3, 146.2, 143.6, 140.7, 139.5, 139.1, 135.1, 126.2, 121.8, 121.6, 113.5, 113.0, 110.5, 109.6, 108.7, 106.2, 75.8, 62.1, 62.1, 42.8, 26.2, 19.0, 18.7, -4.7, -5.1; **HRMS** (ESI+) 638.1973, calc. 638.1971 $[M+H]^+$.

CBS100 (**5**)

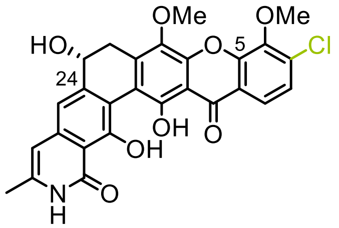

Procedure: To a suspension of bisphenol **34** (6.61 mg, 10.4 µmol, 1.00 eq.) in MeCN (1 mL) was added CuBr(OH)·NMI_2 (10.1 mg, 31.1 µmol, 2.99 eq.) and the reaction was stirred at 75 °C for 75 min. MeCN was removed under reduced pressure, MeOH (1 mL) and 3 M HCl (1.00 mL, 3.00 mmol, 288 eq.) were added and the reaction was stirred at room temperature for 1 h. Then, saturated NH_4Cl (5 mL) was added and the water layer was extracted with 2-Me-THF (4×5 mL). The combined organic layers were dried over Na_2SO_4, filtered and concentrated under reduced pressure to furnish crude **5**. Crude **5** was triturated with MeCN (600 µL), THF (600 µL) and acetone (600 µL) under ultrasound. Purification of the collected supernatant in 4 portions using preparative HPLC (Sphinx, 15 mL/min, MeCN/H_2O 6:4) yielded CBS100 (**5**) as a yellow solid containing aliphatic trace impurities (98% purity, 3.24 mg, 6.06 µmol, 58%), which may be removed by re-crystallization from hot MeOH/DCM (Figure S1, full scale spectra in Chapter 7.3).
R*t* (Sphinx, MeCN/H_2O 6:4) 5.10 min; $[\boldsymbol{\alpha}]_{\mathbf{D}}^{\mathbf{20}}$ (c=0.65, DMSO) -175.9°; **^{1}H NMR 298 K** (700 MHz, DMSO) δ 13.85 (s, 1H), 12.93 (s, 1H), 11.77 (bs, 1H), 7.95 (d, *J* = 8.6 Hz, 1H), 7.60 (d, *J* = 8.6 Hz, 1H), 7.18 (bs, 1H), 6.52 (s, 1H), 5.94 (bs, 1H), 4.49 (bs, 1H), 4.13 (s, 3H), 3.95 (s, 3H), 3.41 (bs, 1H), 2.46 (bs, 1H), 2.26 (d, *J* = 1.0 Hz, 3H); **^{13}C NMR 298 K** (176 MHz, DMSO, 298 K) δ 181.2, 166.9, 157.5, 153.2, 149.8*, 147.3, 144.6, 140.0, 138.8, 138.6, 135.7, 133.3, 125.3, 120.8, 120.5, 114.1, 112.0, 109.0, 108.7, 107.5, 105.4, 67.5, 61.6, 61.6, 33.0, 18.6; **^{1}H NMR 384 K** (700 MHz, DMSO, 348 K) δ 13.79 (s, 1H), 12.84 (bs, 1H), 11.56 (bs, 1H), 7.96 (dd, *J* = 8.7, 2.0 Hz, 1H), 7.58 (dd, *J* = 8.7, 2.0 Hz, 1H), 7.15 (bs, 1H), 6.48 (s, 1H), 5.57 (bs, 1H), 4.58 (bs, 1H), 4.15 (d, *J* = 1.9 Hz, 3H), 3.97 (d, *J* = 1.8 Hz, 3H), 3.24 (bs, 1H), 2.72 (bs, 1H), 2.28 (s, 3H); **^{13}C NMR 384 K** (176 MHz, DMSO) δ 180.8, 166.6, 157.4, 153.0, 149.4*, 147.0, 144.4, 139.7, 138.5, 138.3, 136.0, 132.9, 125.0, 120.4, 120.3, 114.1, 112.0, 109.0, 108.2, 107.2, 105.0, 67.0, 61.3, 61.2, 32.5, 18.2; **^{1}H NMR re-crystallized** (700 MHz, DMSO) δ 13.84 (s, 1H), 12.93 (s, 1H), 11.75 (bs, 1H), 7.96 (d, *J* = 8.7 Hz, 1H), 7.61 (d, *J* = 8.7 Hz, 1H), 7.18 (bs, 1H), 6.51 (s, 1H), 5.91 (bs, 1H), 4.50 (bs, 1H), 4.13 (s, 3H), 3.95 (s, 3H), 3.40

(s, 1H), 2.45 (bs, 1H), 2.27 (d, *J* = 1.0 Hz, 3H); **HRMS** (ESI+) 522.0952, calc. 522.0950 $[M+H]^+$.
* Carbons C5 and C24 overlap. Analytical data were in agreement with those previously reported.[7]

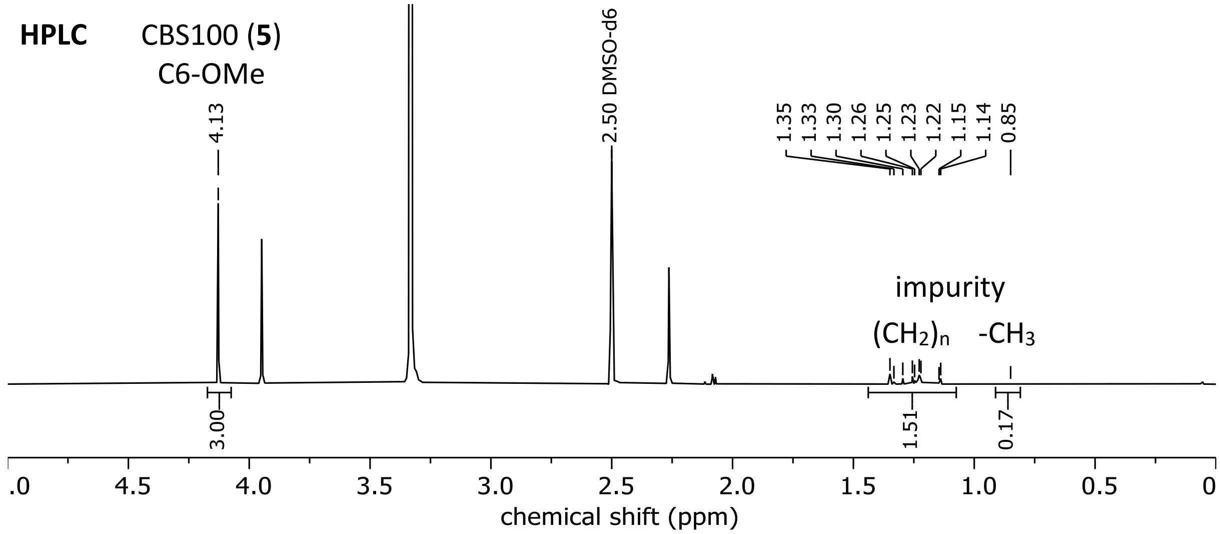

compound	MW [g/mol]	integral	Σ(H)	$\frac{MW \times integral}{\Sigma(H)}$	weight-%
CBS100 (**5**)	521.9	3.00	3	521.9	97.8%
$(CH_2)_n$	14.0	1.51	2	10.6	2.0%
$-CH_3$	15.0	0.17	3	0.9	0.2%
total		-		533.4	100%

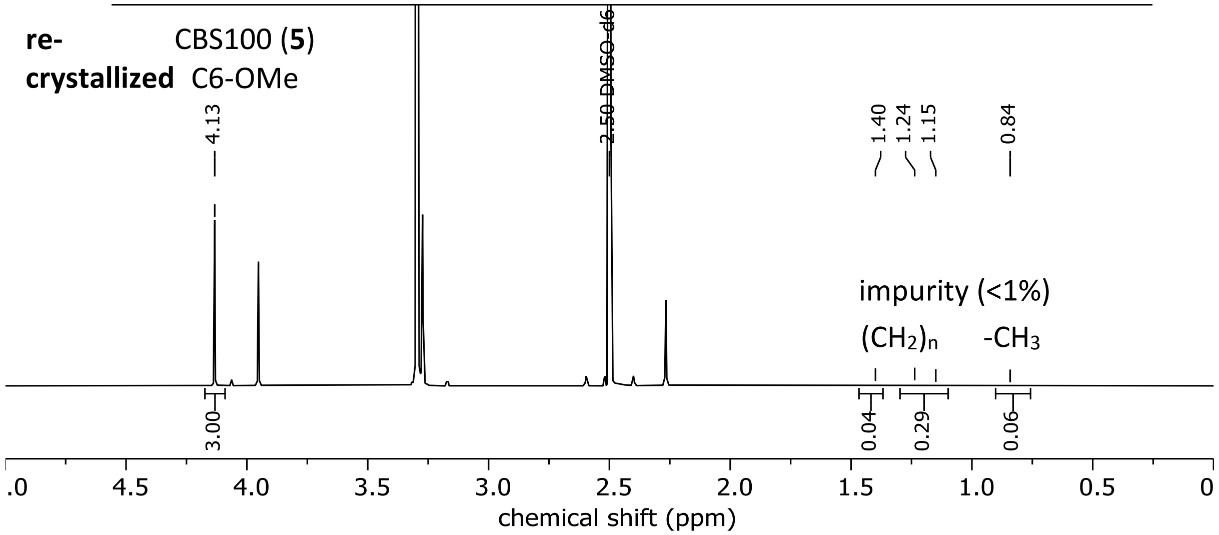

Figure S1. ^{1}H NMR purity of CBS100 (**5**).

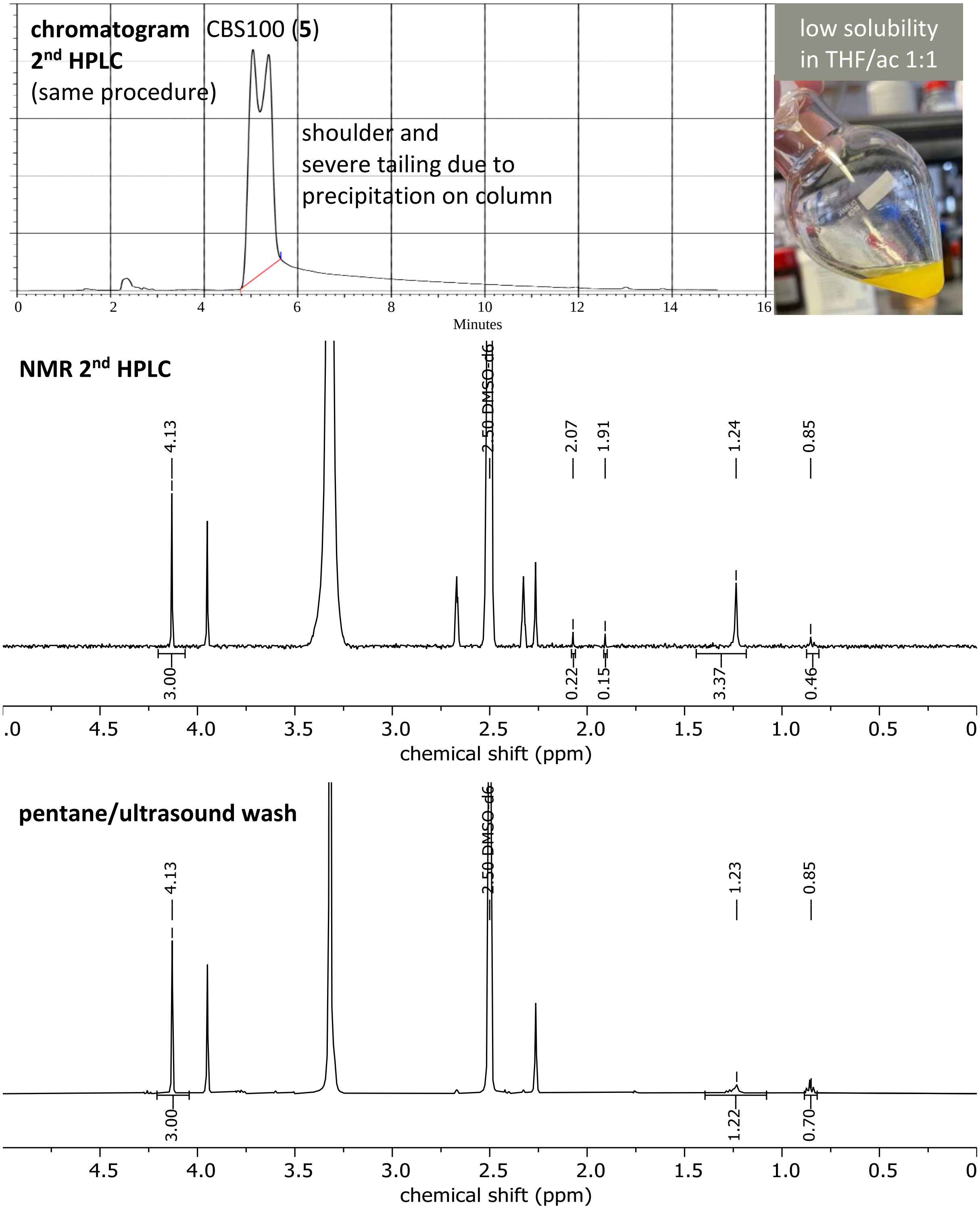

Figure S2. Unsuccessful purification attempts after first HPLC.

2.4 Synthesis of CBS87 (**4**)

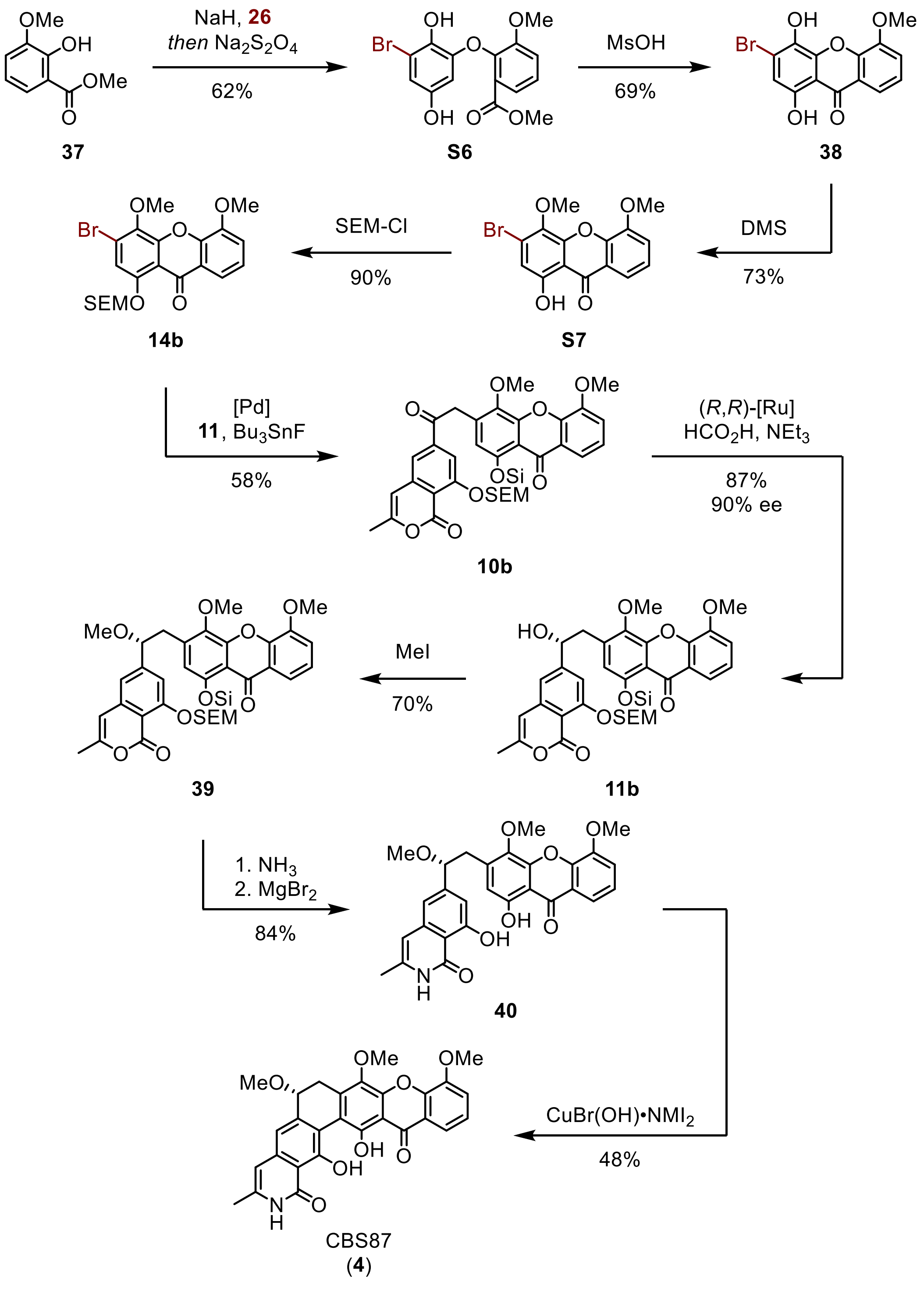

Hydroquinone **S6**

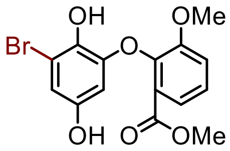

Procedure: To a solution of ester **37** (2.06 g, 11.3 mmol, 1.00 eq.) in DMF (25 mL) at 0 °C was added NaH (60% in mineral oil, 453 mg, 11.3 mmol, 1.00 eq.) and the mixture was stirred at 0 °C for 15 min, after which NaH was fully dissolved. **26** (3.60 mg, 13.5 mmol, 1.19 eq.) was added and the reaction was stirred at 40 °C for 90 min. The reaction was allowed to cool to room temperature for 10 min. Then, Et_2O (200 mL), $Na_2S_2O_4$ (33.4 g, 192 mmol, 17.0 eq.) and H_2O (200 mL) were added and the reduction was stirred at room temperature for 15 min. The phases were separated and the aqueous layer was extracted with EA (3×100 mL). The combined organic layers were dried over Na_2SO_4, filtered and concentrated under reduced pressure. Column chromatography (SiO_2, cy/EA 4:1 → 2:1) yielded **S6** as a white solid (2.58 g, 6.99 mmol, 62%).

R_f (cy/EA 2:1) 0.19; **^{1}H NMR** (500 MHz, Acetone) δ 8.09 (bs, 1H), 7.87 (bs, 1H), 7.46 (dd, *J* = 7.5, 1.9 Hz, 1H), 7.41 (dd, *J* = 8.3, 1.9 Hz, 1H), 7.38 – 7.34 (m, 1H), 6.64 (d, *J* = 2.7 Hz, 1H), 5.95 (d, *J* = 2.7 Hz, 1H), 3.82 (s, 3H), 3.72 (s, 3H); **^{13}C NMR** (126 MHz, Acetone) δ 166.2, 154.1, 151.1, 148.5, 143.5, 138.2, 127.0, 126.9, 123.2, 117.7, 112.5, 109.7, 102.6, 56.7, 52.5; **HRMS** (EI) 367.9888, calc. 367.9896 $[M]^+$. Analytical data were in agreement with those previously reported.[26]

Xanthone **38**

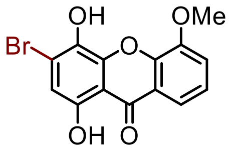

Procedure: A mixture of hydroquinone **S6** (2.56 g, 6.93 mmol, 1.00 eq.) in MsOH (100 mL, 1.04 mol, 150 eq.) was stirred at 70 °C for 4 h. The reaction was quenched by dropwise addition on to sodium citrate dibasic sesquihydrate (350 g, 1.33 mol, 192 eq.). The solid was taken up in H_2O (700 mL) and the aqueous phase was extracted with 2-Me-THF (4×200 mL). The combined organic layers were dried over Na_2SO_4, filtered and concentrated under reduced pressure. Recrystallization from MeOH yielded **38** as a yellow solid (1.61 g, 4.78 mmol, 69%).

R*f* (cy/EA 1:1) 0.32; **^{1}H NMR** (400 MHz, Acetone) δ 12.08 (s, 1H), 8.85 (bs, 1H), 7.78 (dd, *J* = 8.0, 1.5 Hz, 1H), 7.55 (dd, *J* = 8.0, 1.5 Hz, 1H), 7.44 (t, *J* = 8.0 Hz, 1H), 7.00 (s, 1H), 4.04 (s, 3H); **^{13}C NMR** (176 MHz, Acetone) δ 182.9, 154.6, 149.8, 147.1, 145.8, 136.4, 125.4, 122.1, 120.2, 118.1, 117.0, 113.7, 109.3, 57.0; **HRMS** (EI) 335.9635, calc. 335.9633 $[M]^+$. Analytical data were in agreement with those previously reported.[26]

Anisole **S7**

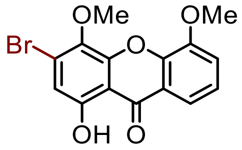

Procedure: To a mixture of xanthone **38** (169 mg, 501 µmol, 1.00 eq.) and K_2CO_3 (208 mg, 1.50 mmol, 2.99 eq.) in acetone (4 mL) and DMF (0.8 mL) was added DMS (55.0 µL, 578 µmol, 1.15 eq.) and the reaction was stirred at 55 °C for 3 h. Then, H_2O (20 mL), 1 M HCl (5.00 mL, 5.00 mmol, 9.98 eq.) and brine (10 mL) were added, the water layer was extracted with 2-Me-THF (3×15 mL) and each organic layer was filtered over the same silica plug (1×3 cm). The combined filtrates were concentrated under reduced pressure to yield crude **S7** as a slightly yellow, poorly soluble solid (71% w/w, 182 mg, 367 µmol, 73%). Purification of a small sample of crude **S7** (2.80 mg) by column chromatography (cy/THF 5:1) yielded **S7** (2.00 mg, 71% w/w).

R*f* (cy/EA 1:1) 0.73; **^{1}H NMR** (700 MHz, Acetone) δ 12.41 (bs, 1H), 7.79 (dd, *J* = 8.0, 1.4 Hz, 1H), 7.58 (dd, *J* = 7.9, 1.4 Hz, 1H), 7.47 (t, *J* = 8.0 Hz, 1H), 7.02 (s, 1H), 4.10 (s, 3H), 4.04 (s, 3H); **^{13}C NMR** (176 MHz, Acetone) δ 182.9, 157.9, 150.1, 150.0, 147.1, 139.0, 126.2, 125.6, 122.0, 118.2, 116.9, 113.9, 109.7, 61.6, 57.2; **HRMS** (EI) 349.9786, calc. 349.9790 $[M]^+$. Analytical data were in agreement with those previously reported.[26]

SEM Protected **14b**

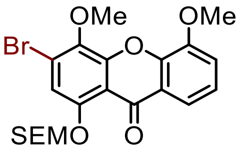

Procedure: To a mixture of crude anisole **S7** (71% w/w, 173 mg, 350 µmol, 1.00 eq.) and K_2CO_3 (484 mg, 3.50 mmol, 10.0 eq.) in acetone (15 mL) was added a SEM-Cl (310 µL, 1.75 mmol, 5.00 eq.) and the reaction was heated to reflux for 16 h. Then, the reaction was filtered over a silica plug (1×3 cm), the product was eluted with 2-Me-THF (20 mL) and the combined organic layers were concentrated under reduced pressure. Column chromatography (SiO_2, cy/EA 4:1) yielded **14b** as a white solid (151 mg, 314 µmol, 90%).

R_f (cy/EA 4:1) 0.41; **^{1}H NMR** (500 MHz, Acetone) δ 7.72 (dd, *J* = 8.0, 1.5 Hz, 1H), 7.44 (dd, *J* = 8.0, 1.5 Hz, 1H), 7.36 (t, *J* = 8.0 Hz, 1H), 7.30 (s, 1H), 5.38 (s, 2H), 4.07 (s, 3H), 4.07 (s, 3H), 3.92 – 3.87 (m, 2H), 1.02 – 0.95 (m, 2H), 0.02 (s, 9H); **^{13}C NMR** (126 MHz, Acetone) δ 175.3, 154.3, 152.1, 149.8, 145.9, 142.0, 125.0, 124.5, 123.0, 117.6, 116.8, 115.3, 114.7, 95.3, 67.4, 61.6, 57.1, 18.6, -1.2; **HRMS** (ESI+) 503.0496, calc. 503.0496 $[M+Na]^+$.

Coupled **10b**

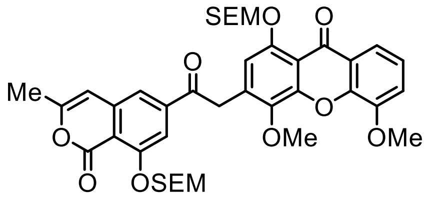

Procedure: In a glove box, a suspension of Pd(P^{t}Bu$_3$)$_2$ (12.4 mg, 24.3 µmol, 7.8 mol-%) and Pd$_2$(dba)$_3$ (10.4 g, 11.4 µmol, 3.6 mol-%) in toluene (4 mL) was vigorously stirred at room temperature for 30 min and the supernatant (3.0 mL) was added to a mixture of bromide **14b** (150 mg, 312 µmol, 1.00 eq.), enol **13c** (328 mg, 780 µmol, 2.50 eq.) and Bu$_3$SnF (245 mg, 793 µmol, 2.54 eq.) in toluene (2 mL). The reaction was tightly sealed and stirred outside the glovebox at 103 °C for 30 min. Then, the reaction was cooled to -40 °C, quickly filtered over a silica plug (3×5 cm, MTBE cooled to -30 °C) and the filtrate was concentrated under reduced pressure. Preparative HPLC (Phenyl-Hexyl, 15 mL/min, MeCN/H$_2$O 7:3 20 min) yielded **10b** as a colourless solid (136 mg, 182 µmol, 58%, 39% brsm **13c**).

R$_t$ (Phenyl-Hexyl, MeCN/H$_2$O 7:3) 14.14 min; **^{1}H NMR** (500 MHz, Acetone) δ 7.79 (d, *J* = 1.5 Hz, 1H), 7.77 (d, *J* = 1.5 Hz, 1H), 7.74 (dd, *J* = 8.0, 1.5 Hz, 1H), 7.42 (dd, *J* = 8.1, 1.5 Hz, 1H), 7.34 (t, *J* = 8.0 Hz, 1H), 7.03 (s, 1H), 6.50 (d, *J* = 1.2 Hz, 1H), 5.48 (s, 2H), 5.32 (s, 2H), 4.58 (s, 2H), 4.05 (s, 3H), 4.02 (s, 3H), 3.91 – 3.84 (m, 4H), 2.24 (d, *J* = 1.0 Hz, 3H), 0.97 – 0.92 (m, 4H), 0.00 (s, 9H), -0.01 (s, 9H); **^{13}C NMR** (126 MHz, Acetone) δ 196.5, 175.6, 160.5, 158.3, 156.9, 153.6, 151.4, 149.8, 146.0, 143.0, 142.6, 141.9, 135.6, 124.6, 124.5, 118.9, 117.7, 116.4, 114.6, 114.4, 113.3, 113.2, 104.0, 95.5, 94.4, 67.4, 67.1, 61.5, 57.0, 41.9, 19.4, 18.6, 18.5, -1.2, -1.3; **HRMS** (ESI+) 771.2625, calc. 771.2627 [M+Na]$^+$.

Side Product of Coupled **10b**

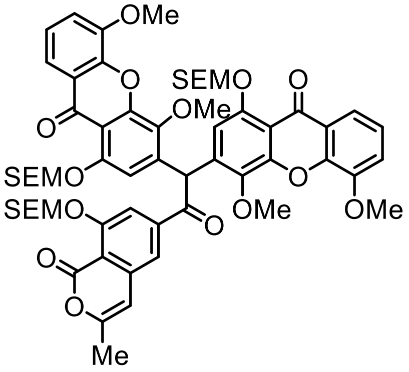

R_t (Phenyl-Hexyl, MeCN/H_2O 7:3) 17.44 min; **^{1}H NMR** (500 MHz, Acetone) δ 7.79 (d, *J* = 1.5 Hz, 1H), 7.73 (dd, *J* = 8.2, 1.6 Hz, 2H), 7.71 (d, *J* = 1.9 Hz, 1H), 7.44 (dd, *J* = 8.1, 1.5 Hz, 2H), 7.34 (t, *J* = 8.0 Hz, 2H), 6.94 (s, 1H), 6.85 (s, 2H), 6.47 (d, *J* = 1.2 Hz, 1H), 5.42 (s, 2H), 5.23 (d, *J* = 1.1 Hz, 4H), 4.07 (s, 6H), 4.05 (s, 6H), 3.82 – 3.76 (m, 2H), 3.75 – 3.69 (m, 4H), 2.21 (d, *J* = 0.9 Hz, 3H), 0.88 – 0.82 (m, 2H), 0.77 – 0.72 (m, 4H), -0.07 (s, 9H), -0.12 (s, 18H); **^{13}C NMR** (126 MHz, Acetone) δ 196.9, 175.5, 160.6, 158.1, 157.0, 153.9, 151.5, 149.8, 146.0, 142.7, 142.2, 141.9, 137.4, 124.7, 124.5, 118.7, 117.7, 116.5, 115.0, 113.6, 113.5, 113.3, 104.1, 95.7, 94.4, 67.4, 66.9, 61.8, 57.0, 49.9, 19.4, 18.5*, -1.4, -1.4; **HRMS** (ESI+) 1171.3969, calc. 1171.3970 $[M+Na]^+$. *Carbons -**C**H_2TMS of all three SEM groups overlap.

Alcohol **11b**

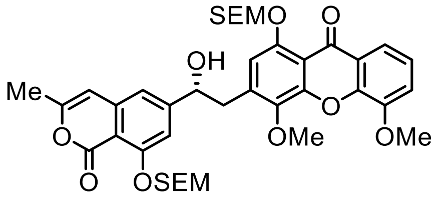

Procedure: To a solution of ketone **10b** (95.1 mg, 127 µmol, 1.00 eq.), HCO_2H (29.0 µL, 769 µmol, 6.05 eq.) and NEt_3 (180 µL, 1.30 mmol, 10.2 eq.) in DCM (3 mL) was added RuCl(*p*-cymene)[(*R*,*R*-Ts-DPEN) (8.10 mg, 12.7 µmol, 10 mol-%) and the reaction was stirred at room temperature for 2 h. Then, the reaction was filtered through a silica plug (1×5 cm, MTBE). Concentration under reduced pressure yielded sufficiently pure **11b** as a brown solid (84.5 mg, 111 µmol, 87%, 90% ee determined by chiral HPLC).

R*f* (cy/EA 1:1) 0.46; **R*t*** (Chiralpak, H/EtOH 7:3) 3.16 min; $[\boldsymbol{\alpha}]_{\mathbf{D}}^{\mathbf{20}}$ (c=1.00, DCM) -8.0°; **^{1}H NMR** (700 MHz, Acetone) δ 7.72 (dd, *J* = 8.0, 1.4 Hz, 1H), 7.42 (dd, *J* = 8.0, 1.4 Hz, 1H), 7.33 (t, *J* = 7.9 Hz, 1H), 7.17 (d, *J* = 1.5 Hz, 1H), 7.08 (d, *J* = 1.4 Hz, 1H), 6.93 (s, 1H), 6.32 (d, *J* = 1.1 Hz, 1H), 5.31 (d, *J* = 6.9 Hz, 1H), 5.29 (d, *J* = 6.9 Hz, 1H), 5.26 (d, *J* = 6.8 Hz, 1H), 5.23 (d, *J* = 6.8 Hz, 1H), 5.13 – 5.08 (m, 1H), 4.80 (d, *J* = 4.2 Hz, 1H), 4.05 (s, 3H), 4.01 (s, 3H), 3.85 (dd, *J* = 8.8, 7.6 Hz, 2H), 3.82 – 3.78 (m, 2H), 3.20 (dd, *J* = 13.3, 7.6 Hz, 1H), 3.17 (dd, *J* = 13.3, 5.9 Hz, 1H), 2.18 (d, *J* = 1.0 Hz, 3H), 0.97 – 0.93 (m, 2H), 0.91 (dd, *J* = 6.5, 2.1 Hz, 1H), 0.89 (dd, *J* = 6.3, 2.1 Hz, 1H), 0.02 (s, 8H), -0.01 (s, 9H); ^{13}C NMR (176 MHz, Acetone) δ 175.5, 160.1, 158.7, 155.8, 154.4, 153.2, 151.4, 149.7, 145.9, 142.7, 141.2, 138.8, 124.5, 124.4, 117.6, 116.2, 116.1, 114.6, 114.0, 112.7, 109.4, 104.1, 95.4, 94.3, 73.9, 67.1, 67.0, 61.5, 56.9, 41.2, 19.4, 18.5, 18.4, -1.2, -1.3; **HRMS** (ESI+) 773.2785, calc. 773.2784 $[M+Na]^{+}$.

Methyl Ether **39**

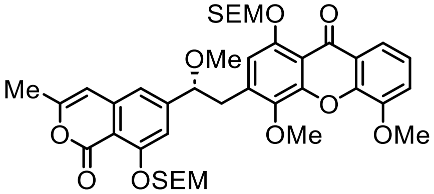

Procedure: To a mixture of alcohol **11b** (36.4 mg, 48.5 µmol, 1.00 eq.) and Ag_2O (68.0 mg, 293 µmol, 6.04 eq.) in MeCN (1.5 mL) was added MeI (300 µL, 4.83 mmol, 99.6 eq.), the reaction was tightly sealed and stirred at 90 °C for 4.5 h. Then, the reaction was filtered through a silica plug (1×3 cm, EA) and concentrated under reduced pressure. Column chromatography (cy/EA 4:1 → 1:1) yielded sufficiently pure **39** as a yellow oil (26.0 mg, 34.0 µmol, 70%).

$[\boldsymbol{\alpha}]_{\mathbf{D}}^{\mathbf{20}}$ (c=1.00, DCM) -26.0°; ^{1}**H NMR** (500 MHz, Acetone) δ 7.72 (dd, *J* = 8.0, 1.5 Hz, 1H), 7.40 (dd, *J* = 8.0, 1.5 Hz, 1H), 7.31 (t, *J* = 8.0 Hz, 1H), 7.12 (d, *J* = 1.4 Hz, 1H), 7.00 (d, *J* = 1.4 Hz, 1H), 6.90 (s, 1H), 6.32 (d, *J* = 1.1 Hz, 1H), 5.33 (d, *J* = 6.9 Hz, 1H), 5.30 (d, *J* = 6.8 Hz, 1H), 5.26 (d, *J* = 6.8 Hz, 1H), 5.22 (d, *J* = 6.8 Hz, 1H), 4.61 (dd, *J* = 7.5, 5.9 Hz, 1H), 4.05 (s, 3H), 3.99 (s, 3H), 3.89 – 3.82 (m, 2H), 3.81 (ddd, *J* = 8.8, 7.3, 1.4 Hz, 2H), 3.27 (s, 3H), 3.25 (dd, *J* = 12.1, 5.6 Hz, 1H), 3.08 (dd, *J* = 13.5, 5.9 Hz, 1H), 2.18 (d, *J* = 0.9 Hz, 3H), 0.96 (dd, *J* = 8.7, 7.5 Hz, 2H), 0.93 – 0.88 (m, 2H), 0.03 (s, 9H), -0.01 (s, 9H); ^{13}**C NMR** (126 MHz, Acetone) δ 175.5, 160.4, 158.6, 156.1, 153.3, 151.4, 151.0, 149.8, 146.0, 142.7, 141.5, 138.3, 124.5, 124.5, 117.7, 116.9, 116.3, 114.4, 114.1, 113.2, 109.9, 104.0, 95.5, 94.4, 83.9, 67.2, 67.0, 61.6, 57.4, 57.0, 39.2, 19.4, 18.6, 18.5, -1.2, -1.2; **HRMS** (ESI+) 787.2939, calc. 787.2940 $[M+Na]^{+}$.

Bisphenol **40**

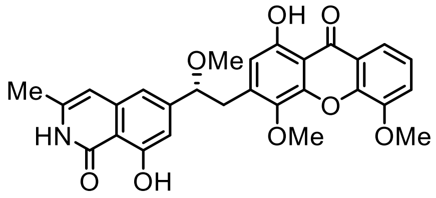

Procedure: A solution of ether **39** (17.1 mg, 22.9 µmol, 1.00 eq.) in methoxyethanol (1 mL) at 0 °C was purged with NH_3 gas for 15 min. The reaction was tightly sealed and heated to 138 °C for 1 h. Then, 1 M HCl (3 mL) was added. The water layer was first extracted with DCM (3×4 mL), filtered over a silica plug (3×3 cm) and the eluent was discarded. Then the water layer was extracted with EA (3×4 mL), filtered over the same silica plug and the eluent was collected. Concentration under reduced pressure gave the partially SEM-deprotected amide.

To a solution of the partially deprotected amide in Et_2O (1 mL) was added $MgBr_2 \cdot Et_2O$ (57.3 mg, 222 µmol, 9.69 eq.) and the reaction was stirred at room temperature for 30 min. Then, the reaction was filtered through a SPE silica plug (EA) and washed with saturated NH_4Cl (5 mL). Concentration under reduced pressure yielded **40** as a yellow solid (9.70 mg, 19.3 µmol, 84%), which could be used directly and may be purified by preparative HPLC (C18, 4 mL/min, MeCN/H_2O 6:4 + 0.05% TFA).

R*t* (C18, MeCN/H_2O 6:4 + 0.05% TFA) 11.93 min; $[\alpha]_D^{20}$ (c=0.20, Aceton) -30.0°; **^{1}H NMR** (700 MHz, Acetone) δ 12.85 (s, 1H), 12.19 (s, 1H), 10.42 (bs, 1H), 7.78 (d, *J* = 8.0 Hz, 1H), 7.54 (d, *J* = 7.8 Hz, 1H), 7.42 (t, *J* = 7.9 Hz, 1H), 6.94 (d, *J* = 1.6 Hz, 1H), 6.76 (d, *J* = 1.5 Hz, 1H), 6.70 (s, 1H), 6.38 (s, 1H), 4.57 (dd, *J* = 8.4, 5.2 Hz, 1H), 4.09 (s, 3H), 4.01 (s, 3H), 3.23 (dd, *J* = 13.7, 8.5 Hz, 1H), 3.21 (s, 3H), 3.08 (dd, *J* = 13.7, 5.2 Hz, 1H), 2.32 (s, 3H); **^{13}C NMR** (176 MHz, Acetone) δ 182.8, 167.6, 162.8, 156.9, 150.3, 150.0, 149.3, 147.2, 142.5, 140.8, 139.4, 139.1, 125.0, 122.0, 117.7, 116.9, 113.9, 112.0, 110.8, 110.1, 108.9, 106.1, 84.1, 61.5, 57.2, 57.1, 39.6, 19.0; **HRMS** (ESI+) 542.1212, calc. 542.1212 $[M+K]^+$.

CBS87 (**4**)

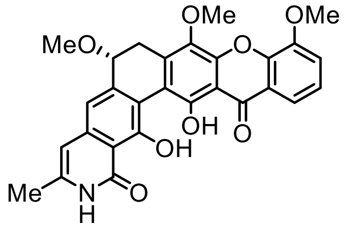

Procedure: To a suspension of bisphenol **40** (1.48 mg, 2.94 µmol, 1.00 eq.) in DMF (1 mL) was added CuBr(OH)·NMI_2 (2.86 mg, 8.82 µmol, 3.00 eq.) and the reaction was stirred at 135 °C for 5 min. MeOH (1 mL) and 3 M HCl (1 mL, 3.00 mmol, 1020 eq.) were added and the reaction was stirred at room temperature for 10 min. Then, saturated NH_4Cl (3 mL) was added and the water layer was extracted with 2-Me-THF (4×3 mL). The combined organic layers were dried over Na_2SO_4, filtered and concentrated under reduced pressure to furnish crude **4**. Crude **4** was triturated with MeCN (400 µL), THF (400 µL) and again MeCN (400 µL) under ultrasound. Purification of the collected supernatant in 3 portions using preparative HPLC (C18, 4 mL/min, MeCN/H_2O 6:4) yielded CBS87 (**4**) as a yellow solid containing trace impurities (94% purity, 700 µg, 1.31 µmol, 45%), which may be removed by re-crystallization from hot MeOH (**Figure S3**, full scale spectra in Chapter 7.4).

R_t (C18, MeCN/H_2O 6:4) 11.87 min; $[\alpha]_D^{20}$ (c=0.14, DMSO) -178.6°; **^{1}H NMR 298 K** (700 MHz, DMSO) δ 13.87 (s, 1H), 13.11 (s, 1H), 11.83 (bs, 1H), 7.77 (dd, *J* = 8.0, 1.4 Hz, 1H), 7.59 (dd, *J* = 8.1, 1.4 Hz, 1H), 7.46 (t, *J* = 8.0 Hz, 1H), 7.07 (bs, 1H), 6.51 (s, 1H), 4.45 (bs, 1H), 4.04 (s, 3H), 3.95 (bs, 3H), 3.63 (bs, 1H), 3.15 (bs, 3H), 2.65 (bs, 1H), 2.27 (s, 3H); **^{13}C NMR 298 K** (176 MHz, DMSO) δ 181.8, 166.9, 158.2, 153.1, 148.4, 147.5, 145.5, 138.9, 136.9*, 124.5, 120.7, 117.3, 115.9, 114.6, 113.4, 112.8*, 110.8, 107.5, 105.2, 75.5, 61.3, 56.7, 55.8, 29.6, 18.6; **^{1}H NMR 384 K** (700 MHz, DMSO) δ 13.80 (bs, 1H), 13.00 (s, 1H), 11.57 (bs, 1H), 7.79 (dd, *J* = 8.0, 1.4 Hz, 1H), 7.58 (dd, *J* = 8.0, 1.4 Hz, 1H), 7.45 (t, *J* = 8.0 Hz, 1H), 7.07 (s, 1H), 6.48 (s, 1H), 4.40 (bs, 1H), 4.06 (s, 3H), 3.99 (s, 3H), 3.34 (bs, 3H), 2.28 (d, *J* = 1.0 Hz, 3H); **^{13}C NMR 384 K** (176 MHz, DMSO) δ 181.4, 166.6, 157.8, 152.8, 148.3, 147.1, 146.7, 145.5, 138.4, 138.2, 124.0, 120.5, 117.4, 115.8, 113.5, 112.6, 110.5, 107.3, 104.9, 76.0, 60.9, 56.6, 55.6, 28.7, 18.1; **^{1}H NMR re-crystallized** (700 MHz, DMSO) δ 13.87 (s, 1H), 13.10 (s, 1H), 11.81 (s, 1H), 7.78 (dd, *J* = 8.0, 1.4 Hz, 1H), 7.59 (dd, *J* = 8.1, 1.4 Hz,

1H), 7.46 (t, J = 8.0 Hz, 1H), 7.08 (s, 1H), 6.51 (s, 1H), 4.44 (s, 1H), 4.04 (s, 3H), 3.96 (s, 3H), 3.65 (s, 1H), 3.17 (s, 3H), 2.66 (s, 1H), 2.27 (s, 3H); **HRMS** (ESI+) 524.1312, calc. 524.1316 $[M+Na]^+$. * HMBC correlation. Analytical data were in agreement with those previously reported.[7]

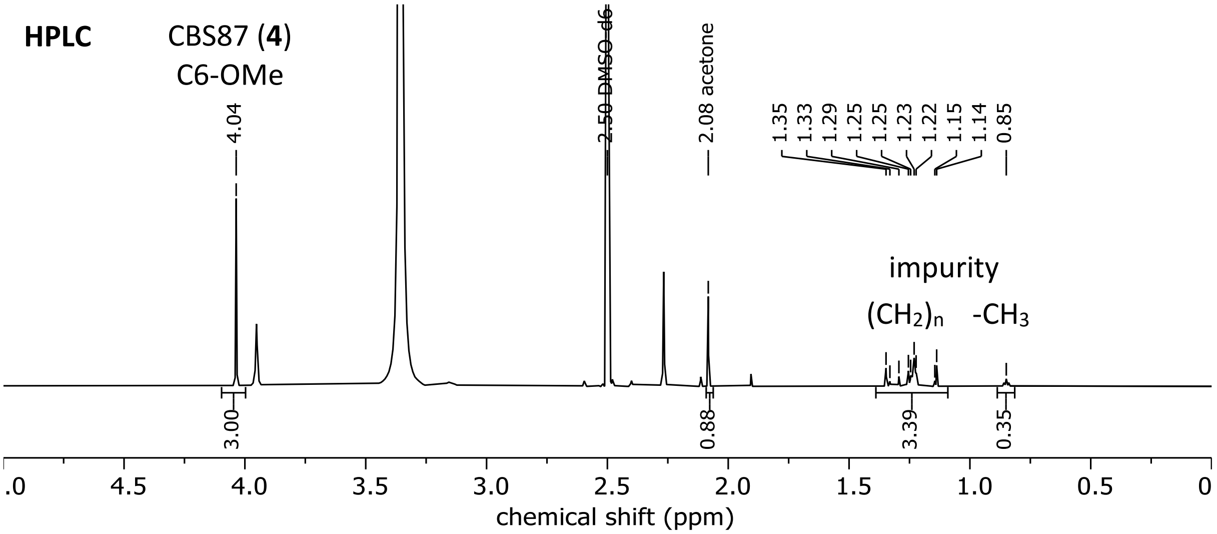

compound	MW [g/mol]	integral	Σ(H)	$\frac{MW \times integral}{\Sigma(H)}$	weight-%
CBS87 (**4**)	501.5	3.00	3	501.5	93.7%
acetone	58.1	0.88	6	8.5	1.6%
$(CH_2)_n$	14.0	3.39	2	23.7	4.4%
$-CH_3$	15.0	0.35	3	1.7	0.3%
total		-		535.4	100%

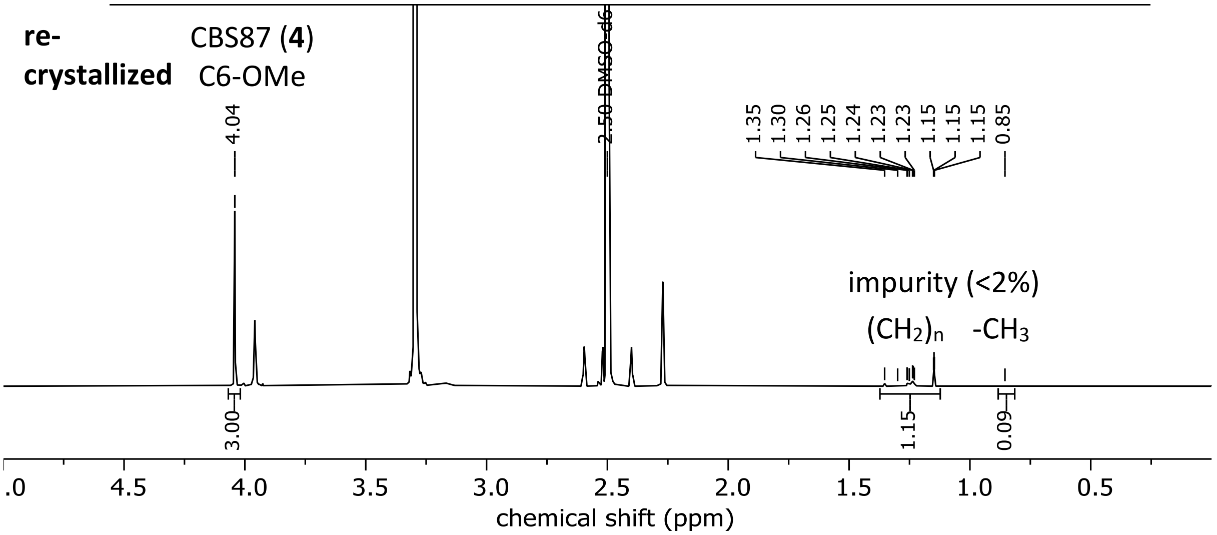

Figure S3. ^{1}H NMR purity of CBS87 (**4**).

2.5 Synthesis of CBS72 (**3**)

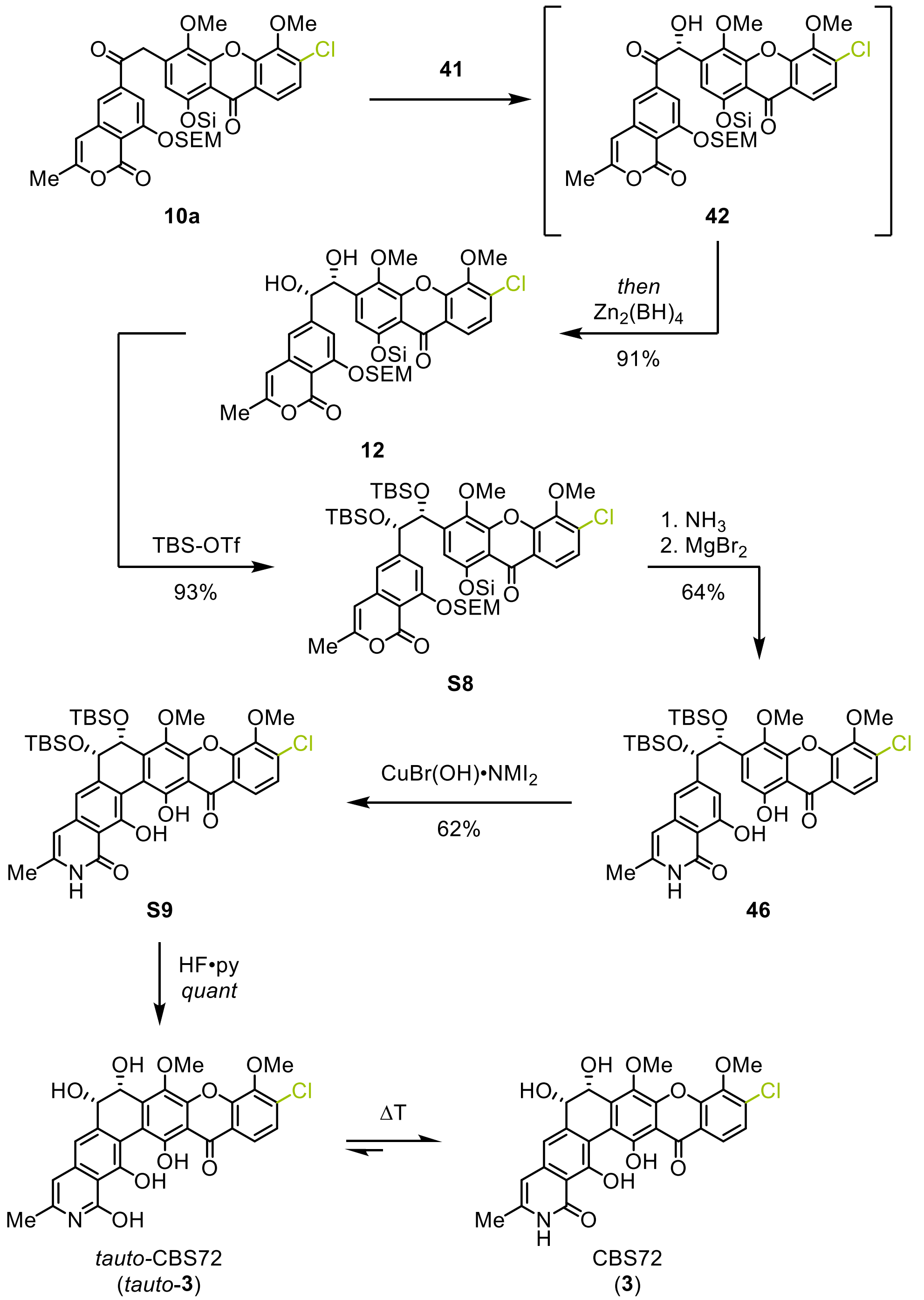

Diol **12**

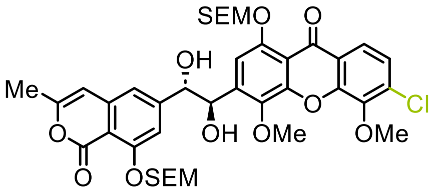

Procedure: To a solution of ketone **10a** (31.3 mg, 40.0 µmol, 1.00 eq.) and **41** (11.9 mg, 51.9 µmol, 1.30 eq.) in THF (1.6 mL) at -90 °C was added dropwise NaHMDS (10.0 mM in THF, 200 µL, 2.00 µmol, 5.0 mol-%, cooled to -90 °C), resulting in a red solution. The ice bath was removed and the reaction was stirred for 6:30 min at room temperature, at which point the solution began to bleach. pH 7 buffer (1 mL) and brine (2 mL) were added. Quickly, the water layer was extracted with MTBE (4×1 mL) and each organic layer was filtered through the same silica plug (1×3 cm topped with Na_2SO_4). Concentration under reduced pressure gave very labile crude hydroxyketone **42**.

Crude hydroxyketone **42** was dissolved in THF (1 mL), $Zn(BH_4)_2$ (500 mM in THF, 200 µL, 100 µmol, 2.50 eq.) was slowly added dropwise and the reaction was stirred at room temperature for 5 min, at which point gas evolution had ceased. pH 7 buffer (1 mL) was slowly added dropwise, followed by brine (2 mL). The water layer was extracted with MTBE (4×2 mL) and the combined organic layers were concentrated under reduced pressure. Column chromatography (cy/EA 1:1) yielded **12** as a white solid (29.0 mg, 36.2 µmol, 91%, 84% ee determined by *dia*-**S11** (Chapter 2.6), dr > 20:1 determined by conversion to the corresponding acetonide).

R*f* (cy/EA 1:1) 0.27; $[\alpha]_D^{20}$ (c=1.00, DCM) -38.0°; **^{1}H NMR** (700 MHz, Acetone) δ 7.90 (d, *J* = 8.6 Hz, 1H), 7.46 (d, *J* = 8.7 Hz, 1H), 7.08 (s, 1H), 7.05 (d, *J* = 1.4 Hz, 1H), 7.02 (d, *J* = 1.4 Hz, 1H), 6.27 (d, *J* = 1.3 Hz, 1H), 5.43 (t, *J* = 5.0 Hz, 1H), 5.20 (d, *J* = 6.8 Hz, 1H), 5.20 (d, *J* = 6.8 Hz, 1H), 5.18 (d, *J* = 6.8 Hz, 1H), 5.15 (d, *J* = 6.8 Hz, 1H), 5.03 (t, *J* = 5.0 Hz, 1H), 4.96 (d, *J* = 4.5 Hz, 1H), 4.80 (d, *J* = 4.6 Hz, 1H), 4.15 (s, 3H), 4.07 (s, 3H), 3.84 – 3.69 (m, 4H), 2.16 (d, *J* = 0.9 Hz, 3H), 0.92 (t, *J* = 8.3 Hz, 2H), 0.84 (ddd, *J* = 9.7, 6.7, 2.9 Hz, 2H), 0.02 (s, 9H), -0.03 (s, 9H); **^{13}C NMR** (176 MHz, Acetone) δ 175.0, 159.6, 158.8, 155.6, 153.8, 151.0, 150.9, 150.2, 145.9, 142.9, 141.4, 140.5, 133.5, 125.7, 124.1, 122.4, 118.2, 114.6, 114.1, 111.6, 109.6, 104.1, 95.4, 94.5, 77.0, 72.3, 67.0, 67.0, 62.1, 61.9, 19.4, 18.4, 18.4, -1.2, -1.3; **HRMS** (ESI+) 823.2345, calc. 823.2343 $[M+Na]^+$.

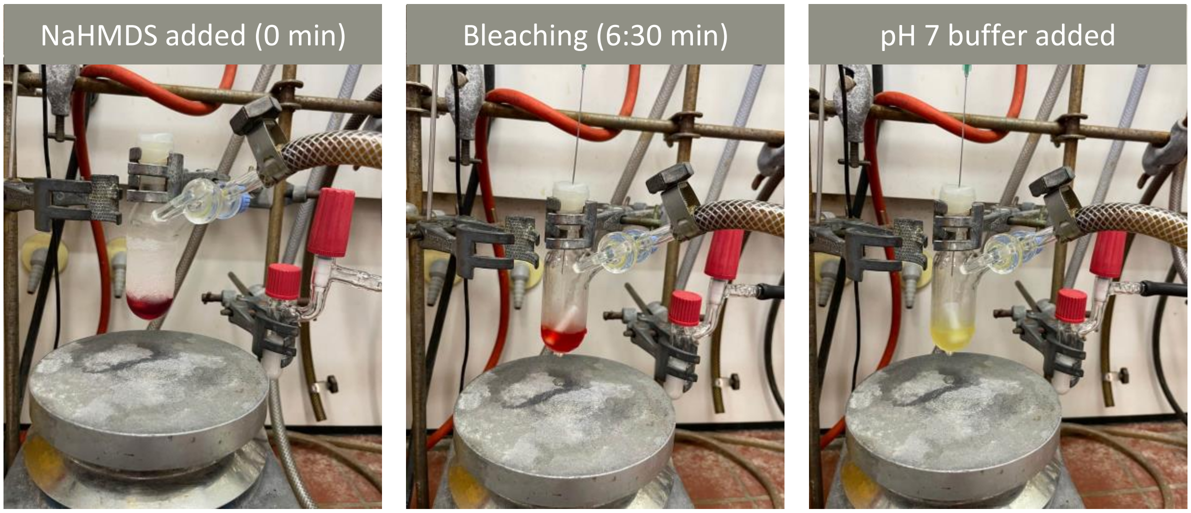
NaHMDS added (0 min)
Bleaching (6:30 min)
pH 7 buffer added

Acetonide of Diol **12**

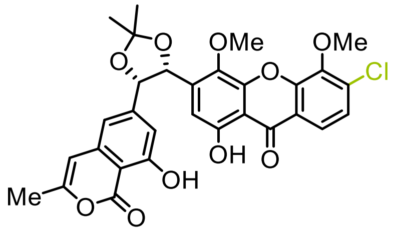

Procedure: A solution of diol **12** (2.38 mg, 2.97 µmol, 1.00 eq.), 2,2-dimethoxypropane (9.80 µL, 80.0 µmol, 26.9 eq.) and *p*-TsOH·H_2O (150 µg, 789 nmol, 0.27 eq.) in acetone (0.5 mL) was stirred at 50 °C for 1 h. The reaction filtered through SiO_2 (0.5×2 cm). Column chromatography (cy/EA 4:1) yielded sufficiently pure acetonide as a slightly yellow solid in quantitative yield (dr > 20:1). $\mathbf{R}_f$ (cy/EA 2:1) 0.48; $[\boldsymbol{\alpha}]_{\mathbf{D}}^{\mathbf{20}}$ (c=0.35, Acetone) -68.6°; ^{1}**H NMR** (400 MHz, Acetone) δ 11.89 (s, 1H), 10.68 (s, 1H), 7.88 (d, *J* = 8.7 Hz, 1H), 7.50 (d, *J* = 8.7 Hz, 1H), 6.83 (s, 1H), 6.79 – 6.74 (m, 1H), 6.71 (s, 1H), 6.37 (s, 1H), 6.05 (d, *J* = 7.8 Hz, 1H), 5.72 (d, *J* = 7.9 Hz, 1H), 4.14 (s, 3H), 4.13 (s, 3H), 2.13 (d, *J* = 1.0 Hz, 3H), 1.83 (s, 3H), 1.63 (s, 3H); ^{13}**C NMR** (176 MHz, Acetone) δ 182.0, 167.1, 161.5, 157.1, 155.1, 151.3, 149.7, 148.5, 146.1, 142.1, 138.3, 137.4, 135.3, 126.3, 121.7, 121.5, 115.0, 113.6, 110.3, 108.8, 108.7, 105.3, 105.2, 81.0, 77.0, 62.0, 62.0, 26.7, 24.4; **HRMS** (APCI) 581.1212, calc. 581.1209 $[M+H]^+$.

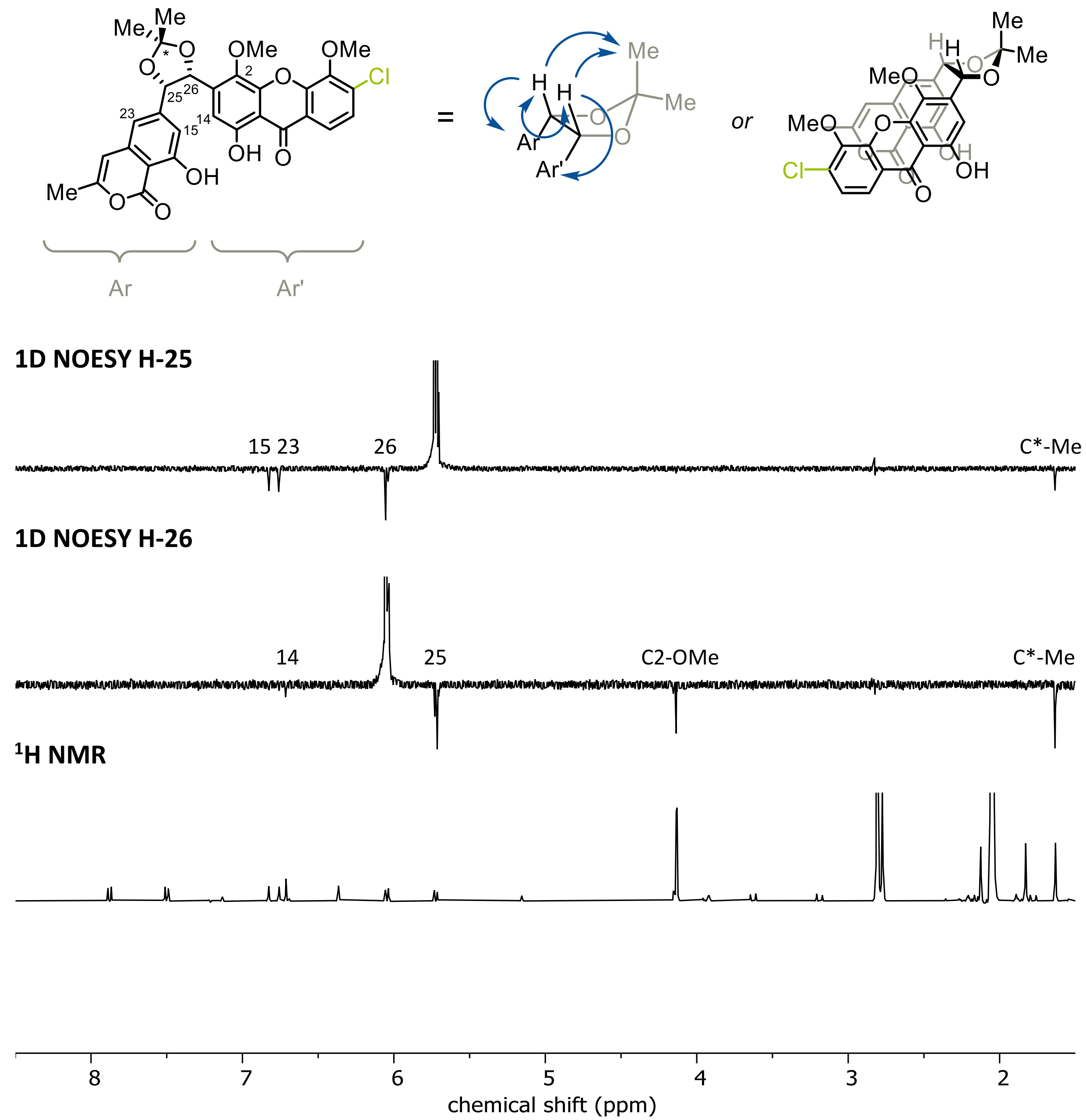

1D NOESY H-25		1D NOESY H-26	
correlation	integral	correlation	integral
H-26	-1.00	H-25	-1.00
H-23	-0.47	C*-Me	-0.70
H-15	-0.44	C2-OMe	-0.35
C*-Me	-0.43	H-14	-0.13

Figure S4. 1D NOESY correlations of α-acetonide hydrogens in acetone.

TBS Ether **S8**

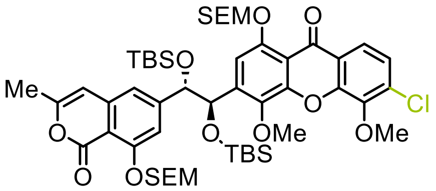

Procedure: To a solution of diol **12** (40.1 mg, 50.0 µmol, 1.00 eq.) and lutidine (40.0 µL, 345 µmol, 7.08 eq.) in DCM (0.5 mL) at room temperature was added dropwise TBS-OTf (40.0 µL, 174 µmol, 3.48 eq.) and the reaction was stirred at room temperature for 2 h. pH 7 buffer (1 mL) and brine (2 mL) were added, the water layer was extracted with MTBE (4×2 mL) and the combined organic layers were concentrated under reduced pressure. Column chromatography (cy/EA 19:1 → 6:1) yielded **S8** as a colorless oil (47.7 mg, 46.3 µmol, 93%).

R*f* (cy/EA 4:1) 0.43; $[\boldsymbol{\alpha}]_{\mathbf{D}}^{\mathbf{20}}$ (c=1.00, DCM) -5.0°; **^{1}H NMR** (499 MHz, Acetone) δ 7.92 (d, *J* = 8.7 Hz, 1H), 7.48 (d, *J* = 8.7 Hz, 1H), 7.21 (bs, 1H), 7.12 (s, 1H), 7.01 (bs, 1H), 6.31 (s, 1H), 5.42 (bs, 1H), 5.31 (d, *J* = 6.9 Hz, 1H), 5.29 (d, *J* = 7.0 Hz, 1H), 5.27 (d, *J* = 6.9 Hz, 1H), 5.23 (d, *J* = 6.9 Hz, 1H), 4.88 (bs, 1H), 4.20 (s, 3H), 4.17 (s, 3H), 3.82 (ddd, *J* = 10.1, 7.9, 2.9 Hz, 4H), 2.19 (s, 3H), 1.00 – 0.92 (m, 4H), 0.80 (s, 9H), 0.77 (s, 9H), 0.04 (s, 9H), 0.02 (s, 9H), -0.10 (s, 3H), -0.13 (s, 3H), -0.14 (s, 3H), -0.14 (s, 3H); **^{13}C NMR** (126 MHz, Acetone) δ 174.9, 160.0, 158.7, 155.9, 154.2, 151.2, 151.0, 150.2, 145.9, 143.0, 141.5, 140.6, 133.6, 125.8, 124.1, 122.4, 118.9, 114.5, 114.2, 110.8, 109.8, 104.0, 95.3, 94.5, 79.8, 73.8, 67.0*, 62.3, 62.0, 26.2, 26.2, 19.4, 18.8, 18.7, 18.7, 18.6, -1.2, -1.2, -4.5, -4.7, -4.7, -4.9; **HRMS** (ESI+) 1051.4077, calc. 1051.4073 $[M+Na]^{+}$. *Carbons -O**C**H_2CH_2TMS of both SEM groups overlap.

Bisphenol **46**

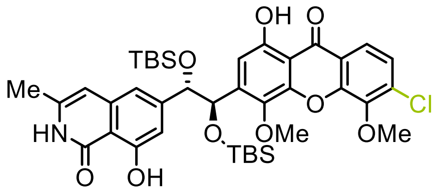

Procedure: A solution of ether **S8** (44.5 mg, 43.2 µmol, 1.00 eq.) in methoxyethanol (0.5 mL) at 0 °C was purged with NH_3 gas for 15 min. The reaction was tightly sealed and heated to 138 °C for 1 h. Then, saturated citric acid (3 mL) was added. The phases were separated, the water layer was extracted with EA (3×3 mL) and each layer was filtered through the same SPE silica plug. Concentration under reduced pressure gave the partially deprotected amide.

To a solution of the partially deprotected amide in Et_2O (2 mL) was added $MgBr_2{\cdot}Et_2O$ (112 mg, 432 µmol, 10.0 eq.) and the reaction was stirred at room temperature for 30 min. Then, saturated NH_4Cl (4 mL) was added. The phases were separated, the water layer was extracted with EA (3×4 mL), each layer was filtered through the same SPE silica plug and the combined organic layers were concentrated under reduced pressure. Preparative HPLC (Phenyl-Hexyl, 16 mL/min, MeCN/H_2O 9:1) yielded **46** as a yellow solid (21.3 mg, 42.3 µmol, 64%).

$\mathbf{R_t}$ (Phenyl-Hexyl, MeCN/H_2O 9:1) 13.75 min; $[\alpha]_D^{20}$ (c=1.00, DCM) -26.0°; **^{1}H NMR** (700 MHz, Acetone) δ 12.80 (s, 1H), 12.05 (s, 1H), 10.44 (bs, 1H), 7.99 (d, *J* = 8.7 Hz, 1H), 7.57 (d, *J* = 8.6 Hz, 1H), 6.98 (bs, 1H), 6.89 (s, 1H), 6.83 (bs, 1H), 6.37 (bs, 1H), 5.34 (bs, 1H), 4.83 (bs, 1H), 4.19 (s, 3H), 4.18 (bs, 3H), 2.33 (d, *J* = 1.0 Hz, 3H), 0.77 (s, 9H), 0.71 (s, 9H), -0.15 (bs, 3H), -0.21 (bs, 3H), -0.22 (bs, 3H), -0.23 (bs, 3H); **^{13}C NMR** (176 MHz, Acetone) δ 182.2, 167.7, 162.2, 157.5, 151.5, 150.5, 149.1, 147.1, 146.2, 140.0, 138.9, 138.7, 135.2, 126.3, 121.8, 121.7, 115.6, 111.7, 110.6, 109.4, 109.1, 106.1, 80.3, 73.5, 62.4, 62.1, 26.1, 26.0, 19.0, 18.7, 18.6, -4.6, -4.9, -4.9, -5.1; **HRMS** (ESI+) 768.2776, calc. 768.2785 $[M+H]^+$.

Cyclized **S9**

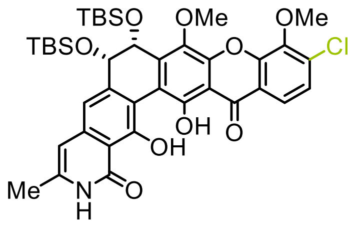

Procedure: To a suspension of bisphenol **46** (7.56 mg, 9.84 µmol, 1.00 eq.) in MeCN (1 mL) was added CuBr(OH)·NMI_2 (9.58 mg, 29.5 µmol, 3.00 eq.) and the reaction was stirred at 75 °C for 75 min. MeCN was removed under reduced pressure, MeOH (1 mL), DMSO (0.5 mL) and 6 M HCl (1 mL, 6.00 mmol, 610 eq.) were added and the reaction was stirred at room temperature for 1 h. Then, half saturated NH_4Cl (5 mL) was added and the water layer was extracted with 2-Me-THF (4×5 mL). The combined organic layers were dried over Na_2SO_4, filtered and concentrated under reduced pressure to furnish crude **S9** as a brown solid (17.0 mg). Crude **S9** was triturated with MeCN (200 µL), THF (200 µL) and again MeCN (500 µL) under ultrasound and the supernatant was collected. Purification of the supernatant in 3 portions using preparative HPLC (Phenyl-Hexyl, 16 mL/min, MeCN/H_2O 9:1) yielded cyclized **S9** as a yellow solid (4.66 mg, 6.08 µmol, 62%).

$\mathbf{R}_t$ (Phenyl-Hexyl, MeCN/H_2O 9:1) 14.26 min; $[\alpha]_D^{20}$ (c=1.00, DCM) -312.0°; **^{1}H NMR** (500 MHz, DMSO) δ 13.78 (s, 1H), 12.90 (s, 1H), 11.76 (d, *J* = 1.6 Hz, 1H), 7.97 (d, *J* = 8.7 Hz, 1H), 7.62 (d, *J* = 8.7 Hz, 1H), 7.06 (d, *J* = 1.2 Hz, 1H), 6.48 (t, *J* = 1.5 Hz, 1H), 5.05 (d, *J* = 2.2 Hz, 1H), 4.78 (dd, *J* = 2.3, 1.2 Hz, 1H), 4.11 (s, 3H), 4.06 (s, 3H), 2.26 (d, *J* = 1.0 Hz, 3H), 1.03 (s, 9H), 0.51 (s, 9H), 0.20 (s, 3H), 0.19 (s, 3H), 0.07 (s, 3H), -0.12 (s, 3H); **^{13}C NMR** (126 MHz, DMSO) δ 181.4, 166.9, 157.2, 153.5, 149.9, 147.5, 144.6, 144.6, 139.3, 138.6, 138.6, 135.2, 133.6, 125.5, 121.0, 120.5, 113.1, 111.9, 110.6, 109.0, 108.9, 105.5, 73.6, 67.0, 62.2, 61.7, 25.9, 25.2, 18.4, 18.2, 17.6, -4.8, -4.8, -4.8, -5.2; **HRMS** (APCI) 766.2631 calc. 766.2629 $[M+H]^+$.

CBS72 (**3**)

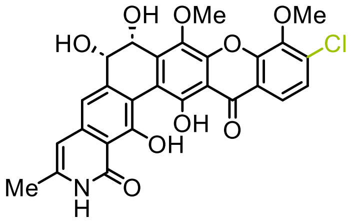

Procedure: To a solution of cyclized **S9** (3.66 mg, 4.78 µmol, 1.00 eq.) in THF (0.9 mL) was added HF·py (70%, 3 drops) and the reaction was stirred at room temperature for 16 h. Then, saturated $NaHCO_3$ (2 mL) was added and the water layer was extracted with 2-Me-THF (5×2 mL). The combined organic layers were concentrated under reduced pressure to furnish crude **3**. Crude **3** was triturated with MeCN (200 µL), MeOH (400 µL) and again MeCN (300 µL) under ultrasound. Purification of the collected supernatant in 3 portions using preparative HPLC (Phenyl-Hexyl, 15 mL/min, MeCN/H_2O 9:11) separated CBS72 (**3**) and its lactim tautomer (1:3 HPLC area). Heating *tauto*-**3** in DMSO (0.5 mL) at 100 °C for 2.5 h yielded CBS72 (**3**) as a yellow solid containing trace impurities (95% purity, 2.57 mg, 4.56 µmol, 95%, **3**:*tauto*-**3** 9:1), which together with *tauto*-**3** may be removed by re-crystallization from hot MeOH (Figure S5, full scale spectra in Chapter 7.5).

R*t* (Phenyl-Hexyl, MeCN/H_2O 9:11) 14.94 min; $[\boldsymbol{\alpha}]_{\mathbf{D}}^{\mathbf{20}}$ (c=1.00, 0.38) -157.1°; **^{1}H NMR** (700 MHz, DMSO) δ 13.82 (bs, 1H), 12.87 (bs, 1H), 11.72 (d, *J* = 1.7 Hz, 1H), 7.96 (d, *J* = 8.6 Hz, 1H), 7.62 (d, *J* = 8.6 Hz, 1H), 7.20 (d, *J* = 1.3 Hz, 1H), 6.50 (dd, *J* = 1.1 Hz, 1H), 5.05 (d, *J* = 2.9 Hz, 1H), 4.52 (dd, *J* = 2.9, 1.3 Hz, 1H), 4.13 (s, 3H), 4.04 (s, 3H), 2.27 (d, *J* = 0.9 Hz, 3H); **^{13}C NMR** (176 MHz, DMSO) δ 181.4, 166.9, 157.2, 153.3, 149.9, 147.5, 145.6, 144.6, 140.3, 138.7, 138.4, 136.1, 133.4, 125.3, 120.9, 120.5, 113.4, 112.0, 111.1, 109.0, 108.6, 105.4, 71.1, 64.9, 62.7, 61.6, 18.6; **^{1}H NMR re-crystallized** (700 MHz, DMSO) δ 13.82 (s, 1H), 12.86 (s, 1H), 11.70 (d, *J* = 1.8 Hz, 1H), 7.97 (d, *J* = 8.6 Hz, 1H), 7.62 (d, *J* = 8.7 Hz, 1H), 7.20 (d, *J* = 1.2 Hz, 1H), 6.49 (t, *J* = 1.4 Hz, 1H), 5.70 (d, *J* = 6.3 Hz, 1H), 5.19 (d, *J* = 4.5 Hz, 1H), 5.05 (dd, *J* = 4.6, 2.9 Hz, 1H), 4.52 (ddd, *J* = 6.3, 3.0, 1.3 Hz, 1H), 4.14 (s, 3H), 4.04 (s, 3H), 2.27 (d, *J* = 1.0 Hz, 3H); **HRMS** (ESI+) 560.0723 calc. 560.0719 $[M+H]^+$. Analytical data were in agreement with those previously reported.[7]

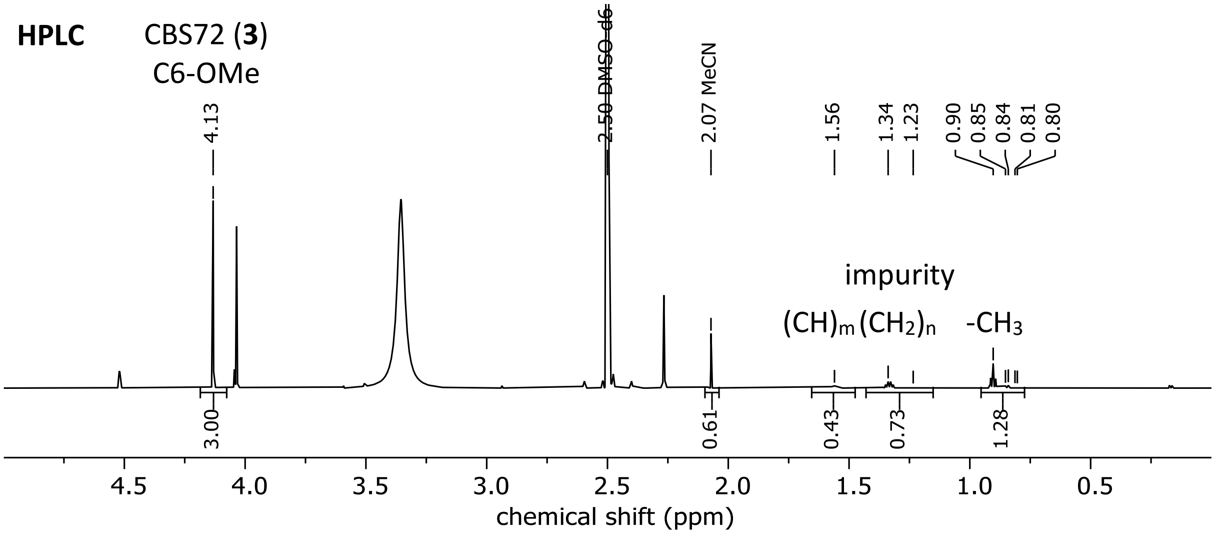

compound	MW [g/mol]	integral	Σ(H)	$\frac{MW \times integral}{\Sigma(H)}$	weight-%
CBS72 (**3**)	537.9	3.00	3	537.9	95.5%
MeCN	41.1	0.61	3	8.4	1.5%
$(CH)_m$	13.0	0.43	1	5.6	1.0%
$(CH_2)_n$	14.0	0.73	2	5.1	0.9%
$-CH_3$	15.0	1.28	3	6.4	1.1%
total		-		563.4	100%

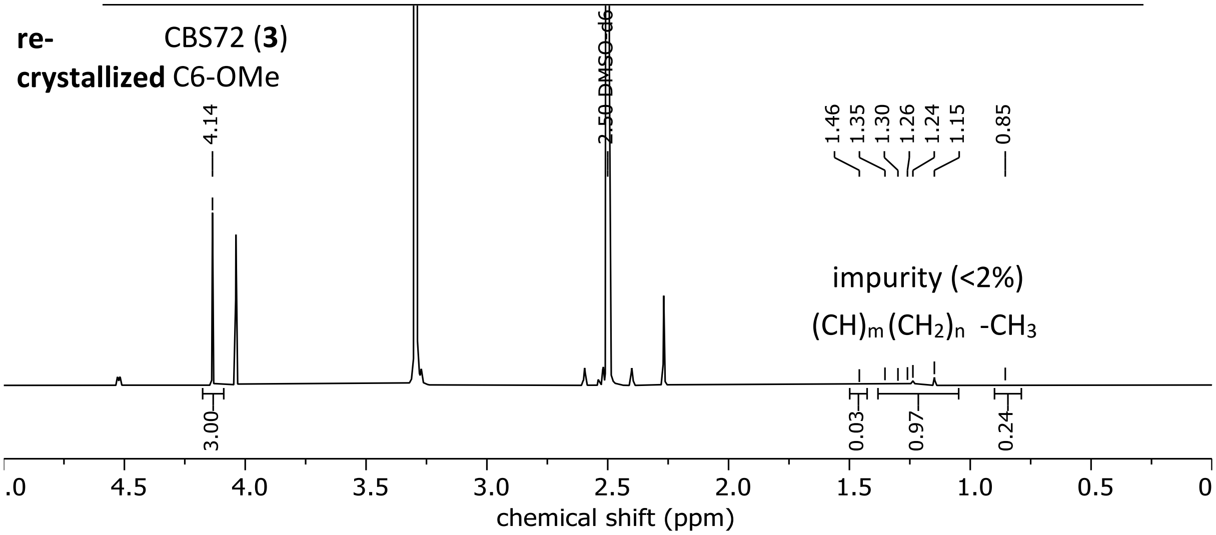

Figure S5. ^{1}H NMR purity of CBS72 (**3**).

Tautomer of CBS72 (*tauto*-**3**)

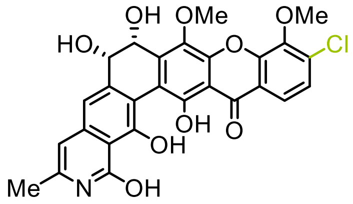

R_t (Phenyl-Hexyl, MeCN/H_2O 9:11) 18.02 min; **^{1}H NMR** (700 MHz, DMSO) δ 13.88 (bs, 1H), 12.82 (s, 1H), 7.97 (dd, *J* = 8.6, 1.7 Hz, 1H), 7.63 (dd, *J* = 8.5, 2.0 Hz, 1H), 7.49 (s, 1H), 7.23 (s, 1H), 6.51 (bs, 1H), 5.93 (bs, 1H), 5.36 (bs, 1H), 5.09 (t, *J* = 2.4 Hz, 1H), 4.62 (s, 1H), 4.13 (s, 3H), 4.05 (d, *J* = 1.7 Hz, 3H), 2.48 (s, 4H); **^{13}C NMR** (176 MHz, DMSO) δ 181.4, 161.0, 153.9, 153.0, 150.0, 149.9, 148.0, 144.6, 140.3, 138.2, 136.0, 133.4, 125.4, 120.9, 120.6, 114.0, 112.5, 111.5, 111.3, 108.7, 105.9, 71.4, 64.8, 62.8, 61.6, 18.5.

2.6 Stereochemical Determination of Diol **12**

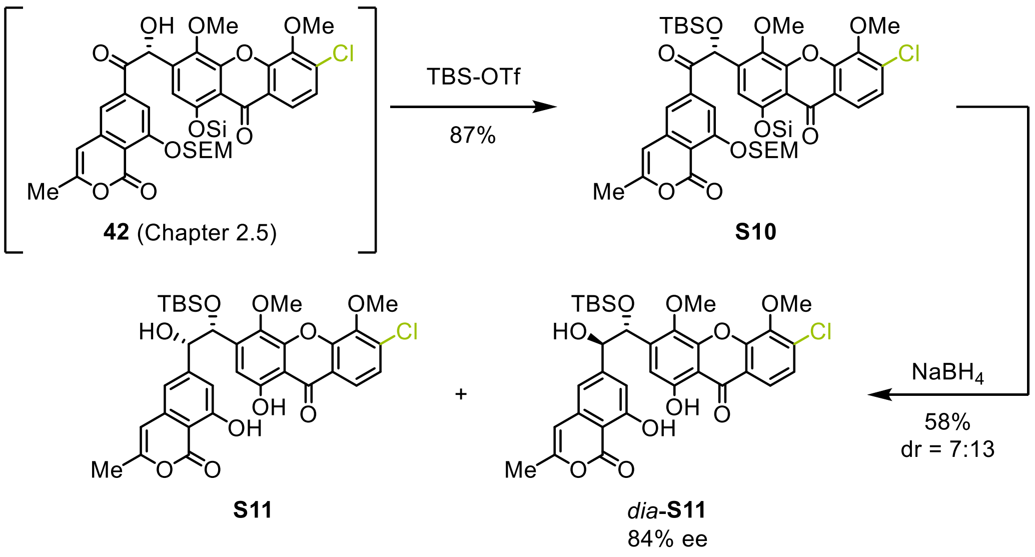

Hydroxyketone **42**

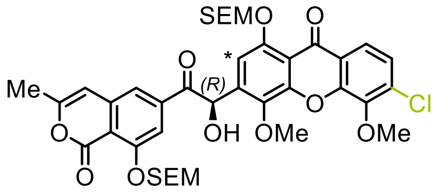

Procedure: Column chromatography of crude hydroxyketone **42** (Chapter 2.5) (cy/EA 4:1 → 2:1) yielded labile **42** (25.1 mg, 31.4 µmol, 63%) alongside the corresponding diketone (9.00 mg, 11.2 µmol, 22%).

R*f* (cy/EA 2:1) 0.28; **^{1}H NMR** (400 MHz, CD_2Cl_2) δ 7.87 (d, *J* = 8.7 Hz, 1H), 7.63 (d, *J* = 1.5 Hz, 1H), 7.49 (d, *J* = 1.5 Hz, 1H), 7.35 (d, *J* = 8.7 Hz, 1H), 6.92 (s, 1H), 6.18 (d, *J* = 5.3 Hz, 1H), 6.16 (d, *J* = 1.1 Hz, 1H), 5.36 (d, *J* = 1.4 Hz, 2H), 5.32 (s, 2H), 4.52 (d, *J* = 5.3 Hz, 1H), 4.11 (s, 3H), 4.09 (s, 3H), 3.85 – 3.78 (m, 2H), 3.74 (ddd, *J* = 9.6, 7.0, 2.5 Hz, 2H), 2.18 (d, *J* = 0.9 Hz, 3H), 0.93 (ddd, *J* = 8.6, 7.5, 3.3 Hz, 2H), 0.89 – 0.79 (m, 2H), 0.00 (s, 9H), -0.06 (s, 9H); **HRMS** (MALDI) 821.2221, calc. 821.2187 $[M+Na]^+$. Compound **42** decomposes during ^{13}C NMR.

Side Product of Intermediate Hydroxyketone **42**

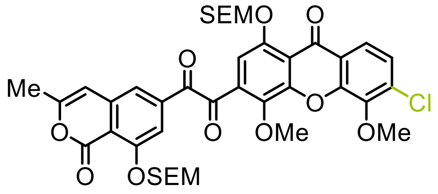

R*f* (cy/EA 2:1) 0.54; **^{1}H NMR** (500 MHz, Acetone) δ 7.92 (d, *J* = 8.7 Hz, 1H), 7.79 (d, *J* = 1.5 Hz, 1H), 7.61 (d, *J* = 1.5 Hz, 1H), 7.57 (s, 1H), 7.50 (d, *J* = 8.7 Hz, 1H), 6.52 (d, *J* = 1.2 Hz, 1H), 5.51 (s, 2H), 5.49 (s, 2H), 4.04 (s, 3H), 3.96 – 3.92 (m, 2H), 3.91 (s, 3H), 3.91 – 3.85 (m, 2H), 2.24 (d, *J* = 0.9 Hz, 3H), 1.06 – 1.00 (m, 2H), 1.00 – 0.92 (m, 2H), 0.04 (s, 9H), -0.01 (s, 9H); **^{13}C NMR** (126 MHz, Acetone) δ 193.5, 192.0, 174.8, 160.7, 158.1, 157.4, 154.4, 152.0, 150.2, 146.0, 145.7, 142.2, 138.2, 134.3, 131.7, 126.5, 124.2, 122.6, 120.3, 119.1, 114.2, 113.1, 109.4, 103.8, 95.2, 94.6, 67.5*, 62.4, 62.1, 18.6, 18.6*, -1.2, -1.3; **HRMS** (ESI+) 819.2032, calc. 819.2030 $[M+Na]^+$. *Carbons -O**C**H$_2$**C**H$_2$TMS of both SEM groups overlap. Impurity in NMR is reduced Davis reagent.

Mosher of Intermediate Hydroxyketone **42**

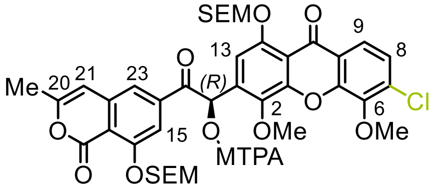

Procedure: To a solution of crude hydroxyketone **42** (Chapter 2.5) and pyridine (3 drops) in DCM (0.2 mL) was added MTPA-Cl (2 drops) and the reaction was stirred at room temperature for 16 h. Then, pH 7 buffer (1 mL) was added and the water layer was extracted with MTBE (3×1 mL). The combined organic layers were dried over Na_2SO_4, filtered and concentrated under reduced pressure. Column chromatography (cy/EA 2:1) yielded the major diastereomer as a labile yellow oil. Mosher ester analysis assigned the alcohol to be (*R*)-**42**.

(*S*)-Ester: 4.80 mg, 4.30 µmol, 43%; **R*f*** (cy/EA 3:1) 0.30; $[\alpha]_D^{20}$ (c=0.29, DCM) -47.6°; **^{1}H NMR** (700 MHz, CD_2Cl_2) δ 7.88 (d, *J* = 8.6 Hz, 1H), 7.64 (d, *J* = 1.6 Hz, 1H), 7.63 (dd, *J* = 7.2, 2.6 Hz, 2H), 7.52 (d, *J* = 1.5 Hz, 1H), 7.47 – 7.45 (m, 2H), 7.48 – 7.43 (m, 3H), 7.41 (s, 1H), 7.36 (d, *J* = 8.7 Hz, 1H), 7.02 (s, 1H), 6.19 (d, *J* = 1.2 Hz, 1H), 5.40 (d, *J* = 6.9 Hz, 1H), 5.38 (d, *J* = 6.9 Hz, 1H), 5.27 (d, *J* = 7.0 Hz, 1H), 5.25 (d, *J* = 7.0 Hz, 1H), 4.08 (s, 6H), 3.80 – 3.74 (m, 4H), 3.57 (s, 3H), 2.21 (d, *J* = 0.9 Hz, 3H), 0.91 – 0.83 (m, 4H), -0.02 (s, 9H), -0.06 (s, 9H); **^{13}C NMR** (176 MHz, CD_2Cl_2) δ 190.9, 174.8, 166.2, 159.9, 158.4, 156.6, 154.4, 151.4, 149.5, 145.3, 141.4, 141.2, 139.2, 134.2, 131.9, 131.7, 130.3, 128.9, 128.2, 125.9, 123.3, 122.1, 118.2, 115.5, 113.2, 112.3, 110.8, 103.6, 94.9, 93.9, 85.3*, 74.4, 67.5, 67.3, 62.8, 61.9, 56.0, 19.7, 18.3, 18.3, -1.4, -1.5; **HRMS** (ESI+) 1037.2593, calc. 1037.2585 $[M+Na]^+$. ***C**-CF_3 assigned through HMBC. **^{13}C**F_3 not observed due to low resolution after quadrupole splitting.

(*R*)-Ester: 6.10 mg, 5.47 µmol, 55%; **R*f*** (cy/EA 3:1) 0.30; $[\alpha]_D^{20}$ (c=0.39, DCM) -48.2°; **^{1}H NMR** (700 MHz, CD_2Cl_2) δ 7.87 (d, *J* = 8.7 Hz, 1H), 7.66 (d, *J* = 1.7 Hz, 1H), 7.65 (d, *J* = 3.8 Hz, 2H), 7.54 (d, *J* = 1.5 Hz, 1H), 7.43 – 7.40 (m, 1H), 7.42 – 7.39 (m, 2H), 7.40 (s, 1H), 7.35 (d, *J* = 8.7 Hz, 1H), 6.98 (s, 1H), 6.20 (d, *J* = 1.1 Hz, 1H), 5.40 (d, *J* = 6.9 Hz, 1H), 5.39 (d, *J* = 6.9 Hz, 1H), 5.24 (d, *J* = 6.9 Hz, 1H), 5.23 (d, *J* = 6.9 Hz, 1H), 4.07 (s, 3H), 3.98 (s, 3H), 3.78 – 3.73 (m, 4H), 3.76 (s, 3H), 2.21 (d, *J* = 0.9 Hz, 3H), 0.91 – 0.84 (m, 4H), -0.02 (s, 9H), -0.05 (s, 9H); **^{13}C NMR** (176 MHz, CD_2Cl_2) δ 191.2, 174.8, 166.3, 160.0, 158.4, 156.7, 154.3, 151.3, 149.4, 145.3, 141.4, 141.2, 139.2, 134.1, 132.3, 131.5, 130.3, 128.9, 127.8, 125.9, 123.3, 122.1, 118.2, 115.5, 113.2, 112.3, 110.7, 103.6, 94.9,

93.9, 85.1*, 74.3, 67.5, 67.2, 62.7, 61.9, 56.3, 19.7, 18.3, 18.3, -1.4, -1.5; **HRMS** (ESI+) 1037.2593, calc. 1037.2585 $[M+Na]^+$. ***C**-CF_3 assigned through HMBC. **^{13}C**F_3 not observed due to low resolution after quadrupole splitting.

hydrogen	(*S*)-ester [ppm]	(*R*)-ester [ppm]	$\Delta\delta^{SR}$ [ppm]
H-15	7.62	7.66	-0.04
H-23	7.52	7.54	-0.02
H-21	6.19	7.20	-0.01
C20-Me	2.21	2.21	0.00
C6-OMe	4.08	4.07	0.01
H-8	7.36	7.35	0.01
H-9	7.88	7.87	0.01
H-14	7.02	6.98	0.04
C2-OMe	4.08	3.98	0.10

TBS Ether **S10**

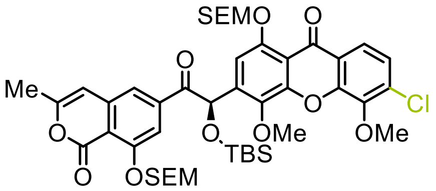

Procedure: Crude hydroxyketone **42** (Chapter 2.5) was dissolved in DCM (2 mL), lutidine (70.0 µL, 604 µmol, 6.04 eq.) and TBS-OTf (70.0 µL, 305 µmol, 3.05 eq.) were added dropwise and the reaction was stirred at room temperature for 2 h. pH 7 buffer (1 mL) and brine (2 mL) were added, the water layer was extracted with MTBE (4×2 mL) and the combined organic layers were concentrated under reduced pressure. Column chromatography (cy/EA 10:1 → 6:1) yielded **S10** as a yellow oil (79.7 mg, 87.2 µmol, 87%, 84% ee determined by chiral HPLC of *dia*-**S11**).

R_f (cy/EA 4:1) 0.33; $[\alpha]_D^{20}$ (c=1.00, DCM) -21.0°; **^{1}H NMR** (500 MHz, Acetone) δ 7.88 (d, *J* = 8.7 Hz, 1H), 7.65 (d, *J* = 1.6 Hz, 1H), 7.64 (d, *J* = 1.6 Hz, 1H), 7.44 (d, *J* = 8.7 Hz, 1H), 7.39 (s, 1H), 6.40 (d, *J* = 1.1 Hz, 1H), 6.30 (s, 1H), 5.38 (d, *J* = 0.9 Hz, 2H), 5.38 (s, 2H), 4.10 (s, 3H), 4.07 (s, 3H), 3.91 – 3.86 (m, 2H), 3.80 – 3.75 (m, 2H), 2.19 (d, *J* = 0.9 Hz, 3H), 1.03 – 0.98 (m, 2H), 0.96 (s, 9H), 0.89 – 0.84 (m, 2H), 0.20 (s, 3H), 0.18 (s, 3H), 0.04 (s, 9H), -0.06 (s, 9H); **^{13}C NMR** (126 MHz, Acetone) δ 196.2, 174.8, 160.1, 158.1, 156.8, 154.6, 151.4, 150.2, 145.9, 141.6, 141.5, 140.9, 139.9, 133.7, 125.9, 124.1, 122.4, 119.0, 114.8, 113.7, 112.9, 111.0, 103.9, 95.3, 94.1, 75.3, 67.4, 67.2, 62.2, 62.0, 26.3, 19.4, 18.9, 18.7, 18.6, -1.2, -1.4, -4.5, -4.6; **HRMS** (ESI+) 935.3063, calc. 935.3052 $[M+Na]^+$.

Alcohol **S11**

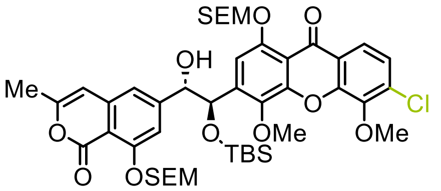

Procedure: To a solution of ether **S10** (36.5 mg, 40.0 µmol, 1.00 eq.) in MeOH (1.5 mL) at room temperature was added $NaBH_4$ (3.02 mg, 79.9 µmol, 2.00 eq.) and the reaction was stirred at room temperature for 5 min. Then, concentrated citric acid (3 mL) was added and the water layer was extracted with MTBE (3×3 mL). The combined organic layers were concentrated under reduced pressure and pre-purified by column chromatography (cy/EA 2:1). Preparative HPLC (ChiarlART, 18 mL/min, H/EtOH 92:8) yielded **S11** as a white solid (7.30 mg, 7.97 µmol, 20%, diastereomer confirmed by conversion to the corresponding TBS ether), alongside *dia*-**S11** (14.0 mg, 15.3 µmol, 38%, 84% ee determined by chiral HPLC, regioisomer confirmed by oxidation to corresponding ketone) and side product (2.60 mg, 2.84 µmol, 7%) both as colourless oil.

$\mathbf{R}_{\boldsymbol{f}}$ (cy/EA 3:1) 0.28; $\mathbf{R}_{\boldsymbol{t}}$ (ChiralART, H/EtOH 92:8) 13.66 min; $\mathbf{R}_{\boldsymbol{t}}$ (Chiralpak, H/EtOH 92:8) 3.45 min; $[\boldsymbol{\alpha}]_{\mathbf{D}}^{\mathbf{20}}$ (c=1.00, DCM) -15.0°; **^{1}H NMR** (500 MHz, Acetone) δ 7.91 (d, *J* = 8.7 Hz, 1H), 7.47 (d, *J* = 8.7 Hz, 1H), 7.14 (d, *J* = 1.4 Hz, 1H), 7.09 (s, 1H), 7.05 (d, *J* = 1.4 Hz, 1H), 6.29 (d, *J* = 1.2 Hz, 1H), 5.45 (d, *J* = 6.0 Hz, 1H), 5.27 (s, 2H), 5.22 (d, *J* = 6.8 Hz, 1H), 5.21 (d, *J* = 6.8 Hz, 1H), 4.90 – 4.84 (m, 1H), 4.82 (d, *J* = 4.1 Hz, 1H), 4.19 (s, 3H), 4.17 (s, 3H), 3.88 – 3.73 (m, 4H), 2.19 (d, *J* = 1.0 Hz, 3H), 0.97 – 0.90 (m, 4H), 0.81 (s, 9H), 0.04 (s, 9H), 0.01 (s, 9H), -0.08 (s, 3H), -0.13 (s, 3H); **^{13}C NMR** (126 MHz, Acetone) δ 175.0, 159.8, 158.8, 155.7, 154.1, 151.3, 151.1, 150.3, 146.0, 143.3, 141.5, 140.5, 133.6, 125.8, 124.1, 122.4, 118.8, 114.7, 114.2, 111.2, 109.7, 104.0, 95.4, 94.6, 78.0, 73.3, 67.0, 67.0, 62.3, 62.0, 26.3, 19.4, 18.7, 18.6, 18.5, -1.2, -1.3, -4.8, -4.8; **HRMS** (ESI+) 937.3211, calc. 937.3208 $[M+Na]^{+}$.

Alcohol *dia*-**S11**

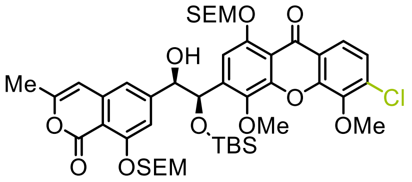

R_f (cy/EA 3:1) 0.33; **R_t** (ChiralART, H/EtOH 92:8) 19.47 min; **R_t** (Chiralpak, H/EtOH 92:8) 5.23 min; $[\alpha]_D^{20}$ (c=1.00, DCM) 19.0°; **^{1}H NMR** (700 MHz, Acetone) δ 7.91 (d, *J* = 8.6 Hz, 1H), 7.46 (d, *J* = 8.7 Hz, 1H), 7.25 (s, 1H), 7.17 (d, *J* = 1.4 Hz, 1H), 7.14 (d, *J* = 1.4 Hz, 1H), 6.33 (d, *J* = 1.1 Hz, 1H), 5.43 (d, *J* = 3.8 Hz, 1H), 5.34 (d, *J* = 6.8 Hz, 1H), 5.33 (d, *J* = 6.9 Hz, 1H), 5.29 (d, *J* = 6.8 Hz, 1H), 5.29 (d, *J* = 6.9 Hz, 1H), 4.96 (dd, *J* = 6.6, 3.8 Hz, 1H), 4.53 (d, *J* = 6.4 Hz, 1H), 4.13 (s, 3H), 4.05 (s, 3H), 3.89 (td, *J* = 9.9, 6.7 Hz, 1H), 3.85 (td, *J* = 9.9, 6.8 Hz, 1H), 3.80 (ddd, *J* = 8.9, 7.1, 1.1 Hz, 2H), 2.18 (d, *J* = 1.0 Hz, 3H), 1.00 (ddd, *J* = 9.8, 6.6, 4.6 Hz, 2H), 0.92 (ddd, *J* = 12.5, 8.9, 7.7 Hz, 2H), 0.87 (s, 9H), 0.04 (s, 8H), -0.00 (s, 9H), -0.14 (s, 3H), -0.16 (s, 3H); **^{13}C NMR** (176 MHz, Acetone) δ 174.9, 159.9, 158.7, 155.9, 153.7, 151.5, 151.0, 150.2, 145.9, 142.6, 140.9, 140.5, 133.6, 125.8, 124.1, 122.5, 117.2, 114.2, 113.6, 111.7, 109.5, 104.1, 95.2, 94.3, 77.5, 74.3, 67.0*, 61.9, 61.9, 26.3, 19.4, 18.8, 18.7, 18.5, -1.2, -1.3, -4.7, -5.1; **HRMS** (ESI+) 937.3202, calc. 937.3208 $[M+Na]^+$. *Carbons -O**C**H_2CH_2TMS of both SEM groups overlap.

Side Product of Alcohol **S11**

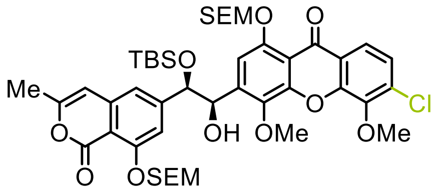

R$_f$ (cy/EA 3:1) 0.42; **R$_t$** (ChiralART, H/EtOH 92:8) 17.85 min; **R$_t$** (Chiralpak, H/EtOH 92:8) 4.72 min; $[\alpha]_D^{20}$ (c=1.00, DCM) +17.0°; **^{1}H NMR** (500 MHz, Acetone) δ 7.91 (d, *J* = 8.7 Hz, 1H), 7.46 (d, *J* = 8.7 Hz, 1H), 7.28 (s, 1H), 7.22 (d, *J* = 1.4 Hz, 1H), 7.11 – 7.05 (m, 1H), 6.32 (d, *J* = 1.2 Hz, 1H), 5.36 (d, *J* = 6.7 Hz, 1H), 5.33 (d, *J* = 5.7 Hz, 1H), 5.32 (d, *J* = 5.5 Hz, 1H), 5.29 (d, *J* = 6.9 Hz, 1H), 5.20 (dd, *J* = 6.7, 4.1 Hz, 1H), 5.15 (d, *J* = 4.1 Hz, 1H), 4.50 (d, *J* = 6.7 Hz, 1H), 4.13 (s, 3H), 4.01 (s, 3H), 3.94 – 3.88 (m, 2H), 3.80 (t, *J* = 8.3 Hz, 2H), 2.17 (d, *J* = 1.0 Hz, 3H), 1.04 – 0.98 (m, 2H), 0.91 (td, *J* = 8.0, 6.0 Hz, 2H), 0.87 (s, 9H), 0.05 (s, 9H), 0.00 (s, 9H), -0.12 (s, 3H), -0.15 (s, 3H); **^{13}C NMR** (126 MHz, Acetone) δ 174.9, 160.0, 158.7, 156.0, 153.9, 151.4, 151.1, 150.3, 145.9, 143.1, 141.0, 141.0, 133.6, 125.8, 124.1, 122.4, 117.2, 114.3, 113.7, 112.0, 109.7, 104.1, 95.5, 94.4, 78.7, 74.0, 67.2, 67.1, 61.9, 61.8, 26.3, 19.4, 18.9, 18.7, 18.6, -1.2, -1.3, -4.7, -5.0; **HRMS** (ESI+) 937.3204, calc. 937.3208 [M+Na]$^+$.

TBS Ether of Alcohol **S11**

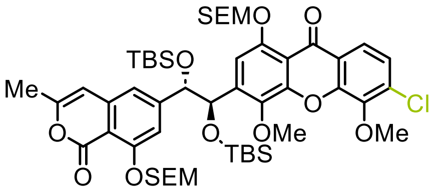

Procedure: To a solution of alcohol **S11** (5.49 mg, 6.00 µmol, 1.00 eq.) and lutidine (5 drops) in DCM (0.3 mL) at room temperature was added dropwise TBS-OTf (3 drops) and the reaction was stirred at room temperature for 1 h. pH 7 buffer (0.5 mL) and brine (2 mL) were added, the water layer was extracted with MTBE (4×2 mL) and the combined organic layers were concentrated under reduced pressure. Column chromatography (cy/EA 19:1 → 6:1) yielded the corresponding TBS ether **S8** as a colorless oil with identical spectroscopic data (5.40 mg, 5.24 µmol, 87%).

R*f* (cy/EA 4:1) 0.43; $[\alpha]_D^{20}$ (c=1.00, DCM) -5.0°; **^{1}H NMR** (499 MHz, Acetone) δ 7.92 (d, *J* = 8.7 Hz, 1H), 7.48 (d, *J* = 8.7 Hz, 1H), 7.21 (bs, 1H), 7.12 (s, 1H), 7.01 (bs, 1H), 6.31 (s, 1H), 5.42 (bs, 1H), 5.31 (d, *J* = 6.9 Hz, 1H), 5.29 (d, *J* = 7.0 Hz, 1H), 5.27 (d, *J* = 6.9 Hz, 1H), 5.23 (d, *J* = 6.9 Hz, 1H), 4.88 (bs, 1H), 4.20 (s, 3H), 4.17 (s, 3H), 3.82 (ddd, *J* = 10.1, 7.9, 2.9 Hz, 4H), 2.19 (s, 3H), 1.00 – 0.92 (m, 4H), 0.80 (s, 9H), 0.77 (s, 9H), 0.04 (s, 9H), 0.02 (s, 9H), -0.10 (s, 3H), -0.13 (s, 3H), -0.14 (s, 3H), -0.14 (s, 3H); **^{13}C NMR** (126 MHz, Acetone) δ 174.9, 160.0, 158.7, 155.9, 154.2, 151.2, 151.0, 150.2, 145.9, 143.0, 141.5, 140.6, 133.6, 125.8, 124.1, 122.4, 118.9, 114.5, 114.2, 110.8, 109.8, 104.0, 95.3, 94.5, 79.8, 73.8, 67.0*, 62.3, 62.0, 26.2, 26.2, 19.4, 18.8, 18.7, 18.7, 18.6, -1.2, -1.2, -4.5, -4.7, -4.7, -4.9; **HRMS** (ESI+) 1051.4077, calc. 1051.4073 $[M+Na]^+$.
*Carbons -O**C**H_2CH_2TMS of both SEM groups overlap.

Ketone of Alcohol *dia*-S11

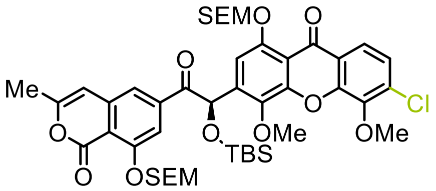

Procedure: To a solution of alcohol *dia*-**S11** (11.9 mg, 13.0 µmol, 1.00 eq.) in DCM (0.4 mL) at room temperature was added DMP (8.27 mg, 19.5 µmol, 1.50 eq.) and the reaction was stirred at room temperature for 45 min. Then, pH 7 buffer (0.5 mL) and saturated $NaHSO_3$ (1 mL) were added. The water layer was extracted with MTBE (4×1 mL), each organic layer was filtered through the same silica plug (0.5×3 cm) and the combined organic layers were concentrated under reduced pressure. Column chromatography (cy/EA 6:1) yielded ketone **S10** as a yellow oil with identical spectroscopic data (11.0 mg, 12.0 µmol, 92%).

R_f (cy/EA 4:1) 0.33; $[\alpha]_D^{20}$ (c=1.00, DCM) -21.0°; **^{1}H NMR** (500 MHz, Acetone) δ 7.88 (d, *J* = 8.7 Hz, 1H), 7.65 (d, *J* = 1.6 Hz, 1H), 7.64 (d, *J* = 1.6 Hz, 1H), 7.44 (d, *J* = 8.7 Hz, 1H), 7.39 (s, 1H), 6.40 (d, *J* = 1.1 Hz, 1H), 6.30 (s, 1H), 5.38 (d, *J* = 0.9 Hz, 2H), 5.38 (s, 2H), 4.10 (s, 3H), 4.07 (s, 3H), 3.91 – 3.86 (m, 2H), 3.80 – 3.75 (m, 2H), 2.19 (d, *J* = 0.9 Hz, 3H), 1.03 – 0.98 (m, 2H), 0.96 (s, 9H), 0.89 – 0.84 (m, 2H), 0.20 (s, 3H), 0.18 (s, 3H), 0.04 (s, 9H), -0.06 (s, 9H); **^{13}C NMR** (126 MHz, Acetone) δ 196.2, 174.8, 160.1, 158.1, 156.8, 154.6, 151.4, 150.2, 145.9, 141.6, 141.5, 140.9, 139.9, 133.7, 125.9, 124.1, 122.4, 119.0, 114.8, 113.7, 112.9, 111.0, 103.9, 95.3, 94.1, 75.3, 67.4, 67.2, 62.2, 62.0, 26.3, 19.4, 18.9, 18.7, 18.6, -1.2, -1.4, -4.5, -4.6; **HRMS** (ESI+) 935.3063, calc. 935.3052 $[M+Na]^+$.

Mosher of Alcohol *dia*-**S11**

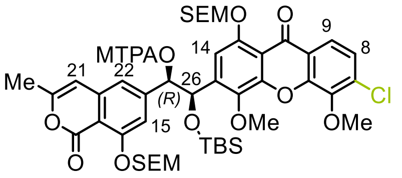

Procedure: To a solution of MTPA (23.4 mg, 99.9 µmol, 20.0 eq.) and DMAP (12.2 mg, 99.9 µmol, 20.0 eq.) in toluene (0.1 mL) at 0 °C was added a solution of TCBC (15.7 µL, 100 µmol, 20.0 eq.) and NEt_3 (13.9 µL, 100 µmol, 20.0 eq.) in toluene (0.1 mL) cooled to 0 °C. The mixture was stirred for 10 min at 0 °C and a solution of alcohol *dia*-**4** (4.58 mg, 5.00 µmol, 1.00 eq.) in toluene (0.1 mL) was added. The ice bath was removed and the reaction was stirred at room temperature for 2 h. Then, pH 7 buffer (1 mL) was added and the water layer was extracted with MTBE (4×1 mL). The combined organic layers were concentrated under reduced pressure. Column chromatography (cy/EA 4:1 → 3:1) yielded the corresponding MTPA-ester as a yellow oil. Mosher ester analysis assigned the alcohol to be (*R*)-**S11**.

(*S*)-Ester: 2.60 mg, 2.30 µmol, 46%; **R*f*** (cy/EA 4:1) 0.29; $[\alpha]_D^{20}$ (c=0.50, DCM) 12.0°; **^{1}H NMR** (700 MHz, Acetone) δ 7.95 (d, *J* = 8.6 Hz, 1H), 7.49 (d, *J* = 8.6 Hz, 1H), 7.34 (d, *J* = 1.5 Hz, 1H), 7.33 – 7.29 (m, 1H), 7.28 – 7.25 (m, 2H), 7.22 (d, *J* = 7.4 Hz, 2H), 7.18 (d, *J* = 1.5 Hz, 1H), 7.13 (s, 1H), 6.41 (d, *J* = 1.2 Hz, 1H), 6.35 (d, *J* = 2.7 Hz, 1H), 5.61 (d, *J* = 2.8 Hz, 1H), 5.48 (d, *J* = 7.0 Hz, 1H), 5.40 (d, *J* = 7.1 Hz, 1H), 5.09 (d, *J* = 7.0 Hz, 1H), 4.97 (d, *J* = 7.0 Hz, 1H), 4.25 (s, 3H), 4.16 (s, 3H), 3.90 – 3.85 (m, 2H), 3.85 – 3.79 (m, 1H), 3.76 – 3.71 (m, 1H), 3.60 (s, 3H), 2.23 (d, *J* = 1.0 Hz, 3H), 1.03 – 0.94 (m, 4H), 0.85 (s, 9H), 0.03 (s, 9H), 0.03 (s, 9H), -0.21 (s, 3H), -0.35 (s, 3H); **^{13}C NMR** (176 MHz, Acetone) δ 174.8, 166.6, 160.5, 158.4, 156.7, 154.1, 150.9, 150.2, 145.9, 145.9, 141.5, 140.5, 139.8, 133.7, 131.7, 130.7, 129.4, 128.3, 125.9, 124.2, 122.5, 117.0, 114.3, 113.0, 110.2, 110.0, 103.8, 94.7, 94.5, 85.7, 80.5, 67.3, 67.1, 62.1, 62.0, 56.3, 26.3, 19.4, 18.7, 18.6, 18.6, -1.3, -1.3, -4.8, -5.6; **HRMS** (ESI+) 1153.3605, calc. 1153.3606 $[M+Na]^+$. **^{13}C**F_3 not observed due to low resolution after quadrupole splitting.

(*R*)-Ester: 2.52 mg, 2.23 µmol, 45%; $[\alpha]_D^{20}$ (c=0.38, DCM) 39.8°; **R*f*** (cy/EA 4:1) 0.18; **^{1}H NMR** (700 MHz, Acetone) δ 7.94 (d, *J* = 8.6 Hz, 1H), 7.49 (d, *J* = 8.6 Hz, 1H), 7.44 – 7.39 (m, 1H), 7.35 (s, 1H), 7.37 – 7.30 (m, 2H), 7.27 (d, *J* = 7.9 Hz, 2H), 7.23 (d, *J* = 1.5 Hz, 1H), 6.96 – 6.93 (m, 1H), 6.27 (d, *J* = 2.9 Hz, 1H), 6.27 (d, *J* = 1.1 Hz, 1H), 5.59 (d, *J* = 2.8 Hz, 1H), 5.42 (d, *J* = 7.0 Hz, 1H), 5.32 (d, *J* =

7.0 Hz, 1H), 5.31 (d, *J* = 6.9 Hz, 1H), 5.24 (d, *J* = 6.9 Hz, 1H), 4.23 (s, 3H), 4.15 (s, 3H), 3.90 – 3.76 (m, 4H), 3.47 (s, 3H), 2.22 (d, *J* = 0.9 Hz, 3H), 1.02 – 0.91 (m, 4H), 0.87 (s, 9H), 0.03 (s, 9H), 0.02 (s, 9H), -0.19 (s, 3H), -0.36 (s, 3H); **^{13}C NMR** (176 MHz, Acetone) δ 174.9, 166.4, 160.4, 158.4, 156.7, 154.3, 151.1, 150.2, 145.9, 145.9, 141.4, 140.8, 140.1, 133.7, 132.5, 130.8, 129.4, 128.3, 125.9, 124.1, 122.5, 116.9, 114.4, 112.8, 110.4, 110.2, 103.8, 94.8, 94.4, 85.5*, 81.0, 72.4, 67.2, 67.2, 62.1, 62.0, 56.1, 26.3, 19.4, 18.7, 18.6, 18.6, -1.3, -1.3, -4.8, -5.6; **HRMS** (ESI+) 1153.3605, calc. 1153.3606 $[M+Na]^+$. ***C**-CF_3 assigned through HMBC. **^{13}C**F_3 not observed due to low resolution after quadrupole splitting.

hydrogen	(*S*)-ester [ppm]	(*R*)-ester [ppm]	$\Delta\delta^{SR}$ [ppm]
H-23	7.18	6.94	0.24
H-21	6.41	6.27	0.14
H-15	7.34	7.23	0.11
H-26	5.61	5.59	0.02
H-8	7.49	7.49	0.00
H-9	7.95	7.94	-0.01
H-14	7.13	7.35	-0.22

2.7 Synthesis of Reagents

CuBr(OH)·NMI$_2$ (**35**)

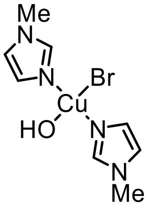

Procedure: Under oxygen, to a suspension of *N*-methylimidazole (350 µL, 4.39 mmol, 2.10 eq.) in MeOH (9.5 mL) and H_2O (1.5 mL) was added CuBr (300 mg, 2.09 mmol, 1.00 eq.) and the reaction was stirred at room temperature for 1 h. Then, the reaction was centrifuged (3000 RPM, 1 min) and the supernatant was concentrated under reduced pressure. Two times, the resulting solid was triturated with acetone (20 mL) under ultrasound for 5 min, centrifuged (3000 RPM, 1 min) and the supernatant was discarded. Drying the precipitant under reduced pressure yielded CuBr(OH)·NMI$_2$ (**35**) as a green solid (505 mg, 1.56 mmol, 75%).

Elemental Analysis (calculated) C 30.05 (29.60), H 4.08 (4.04), N 17.38 (17.26); **HRMS** (ESI+) 227.0349, calc. 227.0352 $[Cu(MeCN)_4]^+$.

Zn(BH$_4$)$_2$

Procedure: In the glove box, a suspension of $ZnCl_2$ (681 mg, 5.00 mmol, 1.00 eq.) and $NaBH_4$ (435 mg, 11.5 mmol, 2.30 eq.) in THF (10 mL) was stirred at room temperature for 72 h. Then, the reaction was centrifuged (1000 RPM, 6 min), the supernatant was transferred into a Schlenk tube under argon, tightly sealed and stored at -30 °C.

3 Reaction Optimizations

3.1 Mono-Enol Ether Stille (**17**)

Conversion of the Triflates in **15** over Time

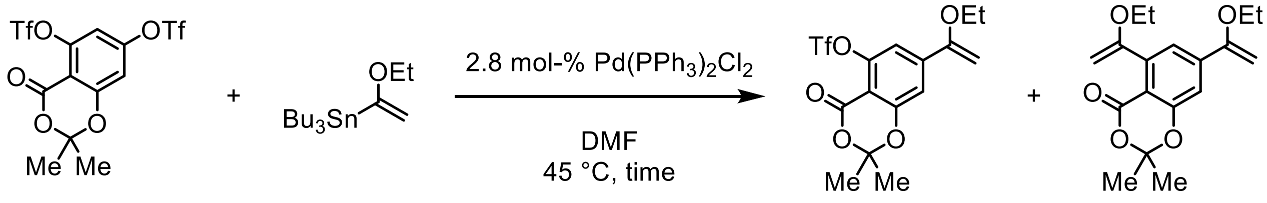

15 1.0 eq | **16** 2.1 eq | **17** desired | **S12**

Procedure: To a solution of **15** (36.1 mg, 76.1 µmol, 1.00 eq.), **16** (55.0 µL, 163 µmol, 2.14 eq.) and triphenylmethane as internal standard (20.5 mg, 83.9 µmol, 1.00 eq.) in DMF (0.6 mL) at room temperature was added $Pd(PPh_3)_2Cl_2$ (1.50 mg, 2.14 µmol, 2.8 mol-%) and the deprotonation was stirred at 45 °C. Repeatedly, aliquots of the reaction solution were diluted with Et_2O, filtered over SiO_2, concentrated under reduced pressure and analyzed by qNMR (Table S1).

Conclusion: Because of the lower step yield to **17** compared to **S12** (entry 1), the reaction temperature was slightly lowered.

Table S1. Results of mono-enol ether Stille.

entry	time	**15**	**17**	**S12**	step yield	OTf of **15**
1	15 min	52%	35%	<5%	73% **17**	1.52 eq
2	1 h	<5%	60%	10%	-	0.87 eq
3	4 h	<5%	22%	44%	89% **S12**	0.35 eq

Rate Equation: Conversion of the Triflates in **15**

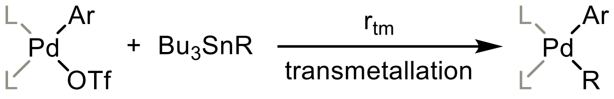

Most likely, the transmetallation of **16** is the rate determining step, resulting in the following rate equation.

$$r_{tm} = -k_{tm} \cdot [\boldsymbol{S13}][\mathbf{16}]$$

Since all other steps in the catalytic cycle are fast, it can be assumed that the resting state of the catalyst is **S13**.

$$[\boldsymbol{S13}] \approx [Pd]_{t=0}$$

By choosing a low catalyst loading, the first term of the rate equation can be considered constant.

$$[\mathbf{16}] \gg [Pd]_{t=0}$$

$$r_{tm} = -k_{tm} \cdot [Pd]_{t=0}[\mathbf{16}] \approx -k'_{tm}[\mathbf{16}]$$

This is a first order differential equation, meaning that the concentration of **c11** follows exponential decay.

$$r_{tm} = \frac{d[\mathbf{16}]}{dt} \approx -k'_{tm}[\mathbf{16}]$$

$$[\mathbf{16}] = c \cdot e^{-k \cdot t}$$

Since **16** cannot be observed directly, the remaining challenge is finding an observable expression. Based on a typical equimolar conversion of **16** and the (pseudo-)halide substrate, the concentration of **16** can be expressed through the conversion of the triflates in **15**.

$$[\mathbf{16}] = [\mathbf{16}]_{t=0} - \Delta[\mathbf{16}]$$

$$\Delta[\mathbf{16}] \approx \Delta[\mathbf{15}]^{OTf}$$

$$[\mathbf{16}] \approx [\mathbf{16}]_{t=0} - \Delta[\mathbf{15}]^{OTf}$$

Keeping in mind that reduction of the palladium(II) pre-catalyst and residual trace oxygen consumes added **16**, we arrive at our final expression.

$$[\mathbf{16}]_{t=0} = [\mathbf{16}]_{added} - 2 \cdot [Pd]_{added} - [O_2] \approx 2$$

$$[\mathbf{16}] \approx 2 - \Delta[\mathbf{15}]^{OTf} = 2 - \left(\frac{[\mathbf{17}]}{yield\ \mathbf{17}} + \frac{[\boldsymbol{S12}]}{yield\ \mathbf{17} \times yield\ \boldsymbol{S12}}\right)$$

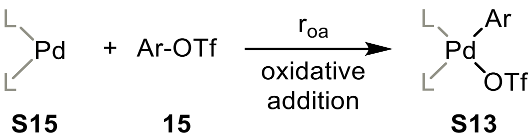

S15 **15** **S13**

In the unlikely case that the oxidative addition of the triflates in **15** is the rate determining step, the following rate equation is obtained.

$$r_{oa} = -k_{oa} \cdot [\boldsymbol{S15}][\mathbf{15}]^{OTf}$$

Under the same assumptions as before, the rate equation can be simplified to a first order differential equation, resulting in exponential decay of the triflates in **15**.

$$[\boldsymbol{S15}] \approx [Pd]_{t=0}$$

$$[\mathbf{15}]^{OTf} \gg [Pd]_{t=0}$$

$$r_{oa} = -k_{oa} \cdot [Pd]_{t=0}[\mathbf{15}]^{OTf} \approx -k'_{oa}[\mathbf{15}]^{OTf}$$

$$r_{oa} = \frac{d[\mathbf{15}]^{OTf}}{dt} \approx -k'_{oa}[\mathbf{15}]^{OTf}$$

$$[\mathbf{15}]^{OTf} = c \cdot e^{-k \cdot t}$$

Because an approximately equimolar initial concentration of the triflates in **15** and **16** was chosen, this ultimately results in the same conversion expression, making the rate expression independent of the rate determining step.

$$[\mathbf{15}]^{OTf} = [\mathbf{15}]^{OTf}_{t=0} - \Delta[\mathbf{15}]^{OTf} = 2 - \Delta[\mathbf{15}]^{OTf}$$

$$[\mathbf{15}]^{OTf} = 2 - \left(\frac{[\mathbf{17}]}{yield\ \mathbf{17}} + \frac{[\boldsymbol{S12}]}{yield\ \mathbf{17} \times yield\ \boldsymbol{S12}}\right)$$

Scale-up: Optimal Reaction Time for Isolation of **17**

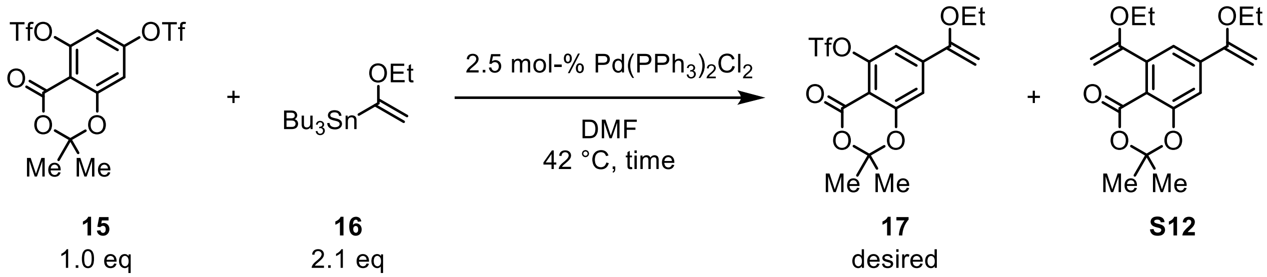

Procedure: See synthesis of enol ether **c3**, results in Table S2.

Conclusion: Reducing the catalyst loading and reaction temperature extended the optimal reaction time to 70 min (entry 2).

Table S2. Influence of reaction time on isolated yield **17**.

entry	time	**17**	notes
1	75 min	79%	slight overreaction to **S12**
2	70 min	85%	-

3.2 Polar Addition (**27**)

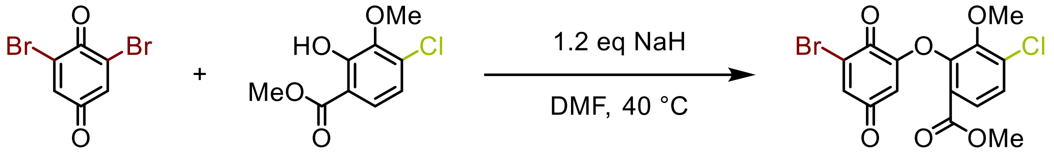

26 1.2 eq   **25** 1.0 eq   **27**

Procedure: To a solution of **25** (20.0 mg, 92.3 µmol, 1.00 eq.) and tecnazene as internal standard (24.1 mg, 92.3 µmol, 1.00 eq.) in DMF (1 mL) at 0 °C was added NaH (60% in mineral oil, 4.43 mg, 111 µmol, 1.20 eq.) and the deprotonation was stirred at 0 °C for 15 min. Then, **26** (29.5 mg, 111 µmol, 1.20 eq.) was added and the reaction was stirred at 40 °C. Repeatedly, aliquots of the reaction solution were filtered over Celite®, concentrated under reduced pressure and analyzed by qNMR.

Conclusion: For good conversion in the following step, complete conversion of **26** was required while maintaining a usable yield of **27**. This was the case for entry 2 (Table S3). Because the formation of an unknown side product correlated with the excess of base used, the amount of sodium hydride was reduced to 1.0 eq for scale-up.

Table S3. Results of polar addition.

entry	variation	time	**26**	**25**	**27**	side product
1		1 h	<5%	16%	45%	14%
2	none	2 h	<5%	17%	51%	15%
3		4 h	<5%	16%	46%	15%
5		1 h	<5%	16%	46%	13%
6	1.5 eq **26**	2 h	<5%	18%	41%	13%
7		4 h	<5%	23%	30%	10%
8		1 h	40%	<5%	48%	21%
9	2.0 eq **26**	2 h	40%	<5%	50%	21%
10		4 h	38%	<5%	49%	20%
11	KO^{t}Bu	1 h	38%	47%	32%	12%
12	instead of	2 h	38%	44%	31%	11%
13	NaH	4 h	38%	45%	34%	12%
14	NaHMDS	1 h	>95%	>95%	<5%	<5%
15	instead of	2 h	97%	>95%	<5%	<5%
16	NaH	4 h	25%	>95%	<5%	<5%
17	THF	1 h	>95%	>95%	<5%	<5%
18	instead of	2 h	>95%	>95%	<5%	<5%
19	DMF	4 h	>95%	>95%	<5%	<5%
20	DCM	1 h	>95%	>95%	<5%	<5%
21	instead of	2 h	>95%	>95%	<5%	<5%
22	DMF	4 h	>95%	>95%	<5%	<5%
23	rt	1 h	44%	8%	40%	19%
24	and	2 h	43%	7%	42%	19%
25	1.7 eq **26**	4 h	43%	6%	36%	16%
26	50 °C	1 h	43%	12%	41%	17%
27	and	2 h	35%	10%	41%	18%
28	1.7 eq **26**	4 h	49%	12%	39%	17%

3.3 Fragment Coupling (10a)

Reaction Condition A: Initial Screening with a Strong Base

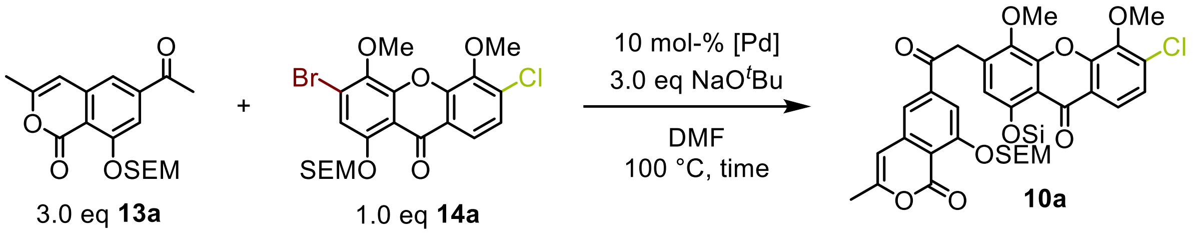

Procedure: To a solution of **14a** (5.16 mg, 10.0 µmol, 1.00 eq.), **13a** (10.5 mg, 30.1 µmol, 3.00 eq.), NaO^{t}Bu (1.00 M in THF, 30.0 µL, 30.0 µmol, 3.00 eq.) and triphenylmethane as internal standard (2.44 mg, 10.0 µmol, 1.00 eq.) in DMF (0.5 mL) at room temperature was added catalyst (1.00 µmol, 10 mol-%) and the reaction was stirred at 100 °C. Repeatedly, aliquots of the reaction solution were diluted with EA, filtered through SiO_2 (EA), concentrated under reduced pressure and analyzed by qNMR (Table S4).

Conclusion: Rapid consumption of acetophenone **13a** together with limited formation of **10a** suggests that **13a** decomposes with a strong base (all entries). Therefore, cross-couplings with a mild base were evaluated next.

Table S4. Results for reaction condition A with strong base.

entry	catalyst	time	**13a**	**14a**	**10a**
1		15 min	4%	43%	<5%
2	Pd XPhos G3	3 h	2%	36%	<5%
3		5 h	<5%	2%	<5%
4		15 min	7%	48%	<5%
5	Pd P^{t}Bu$_3$ G4	3 h	4%	8%	<5%
6		5 h	<5%	6%	<5%
7		15 min	5%	46%	1%
8	Pd dbpf G3	3 h	2%	20%	<5%
9		5 h	<5%	18%	<5%

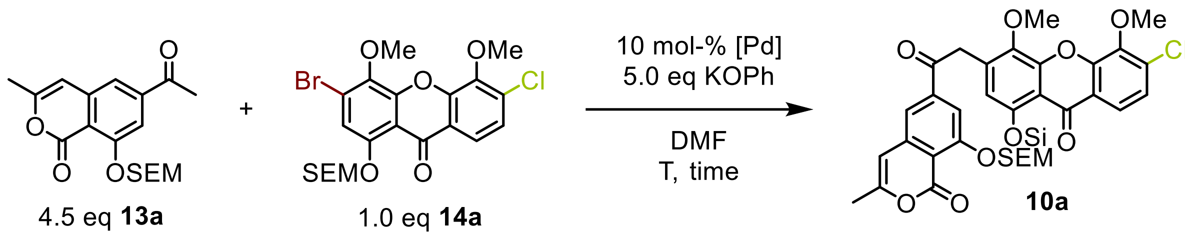

Procedure: To a solution of **14a** (5.16 mg, 10.0 µmol, 1.00 eq.), **13a** (15.7 mg, 45.1 µmol, 4.50 eq.), KOPh (6.60 mg, 49.9 µmol, 4.99 eq.) and triphenylmethane as internal standard (2.44 mg, 10.0 µmol, 1.00 eq.) in DMF (0.5 mL) at room temperature was added catalyst (1.00 µmol, 10 mol-%) and the reaction was stirred with a stepwise increase in temperature. At the end of each temperature step, an aliquot of the reaction solution was diluted with EA, filtered through SiO_2 (EA), concentrated under reduced pressure and analyzed by qNMR (Table S5).

Conclusion: To reduce the influence of decomposition on the yield of **10a**, the equivalents of **13a** and base were increased. In combination with XPhos 10-13% of **10a** was formed (entry 1-3). However, decomposition of bromide **14a** and product **10a** also limited the overall yield. Therefore, further activation conditions were evaluated.

Table S5. Results for reaction condition A with a mild base.

entry	catalyst	additive	time	**13a**	**14a**	**10a**
1	Pd XPhos G3	rt	45 min	-	33%	13%
2		60 °C	45 min	78%	22%	13%
3		100 °C	30 min	65%	<5%	10%
4		100 °C	16 h	30%	<5%	<5%
5	Pd P^tBu_3 G4	rt	45 min	93%	70%	3%
6		60 °C	45 min	74%	57%	3%
7		100 °C	30 min	55%	36%	3%
8		100 °C	16 h	18%	<5%	2%
9	Pd dbpf G3	rt	45 min	98%	76%	1%
10		60 °C	45 min	68%	42%	2%
11		100 °C	30 min	56%	41%	<5%
12		100 °C	16 h	15%	<5%	<5%

Reaction Condition B: Initial Screening with a Weak Base

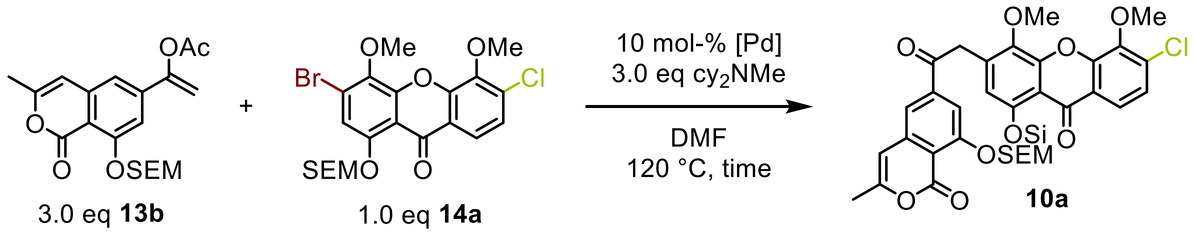

Procedure: To a solution of **14a** (5.16 mg, 10.0 µmol, 1.00 eq.), **13b** (11.7 mg, 30.0 µmol, 3.00 eq.), cy_2NMe (6.42 µL, 30.0 µmol, 3.00 eq.) and triphenylmethane as internal standard (2.44 mg, 10.0 µmol, 1.00 eq.) in DMF (0.5 mL) at room temperature was added catalyst (1.00 µmol, 10 mol-%) and the reaction was stirred at 120 °C. Repeatedly, aliquots of the reaction solution were diluted with EA, filtered through SiO_2 (EA), concentrated under reduced pressure and analyzed by qNMR (Table S6).

Conclusion: Since enol ethers are typically challenging reaction partners in Heck reactions, the reaction temperature was increased to 120 °C. In combination with dppf, 15% **10a** was formed (entry 3). However, rapid consumption of enol ether **13b** suggests that **13b** decomposes under mild basic conditions (all entry). Therefore, tin mediated cross-couplings were evaluated next.

Table S6. Results for reaction condition B.

entry	catalyst	time	**13b**	**14a**	**10a**
1		1 h	54%	74%	<5%
2	Pd XPhos G3	4 h	39%	72%	<5%
3		16 h	5%	50%	<5%
4		1 h	50%	74%	<5%
5	Pd P^tBu_3 G4	4 h	45%	66%	<5%
6		16 h	29%	55%	<5%
7		1 h	51%	70%	<5%
8	Pd dppf G3	4 h	33%	63%	<5%
9		16 h	5%	30%	15%

Reaction Condition C: Initial Screening

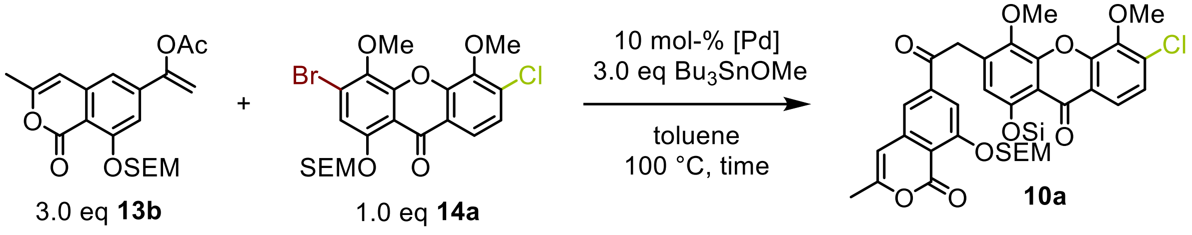

Procedure: To a solution of **14a** (5.16 mg, 10.0 µmol, 1.00 eq.), **13b** (11.7 mg, 30.0 µmol, 3.00 eq.), Bu_3SnOMe (8.98 µL, 30.0 µmol, 3.00 eq.) and triphenylmethane as internal standard (2.44 mg, 10.0 µmol, 1.00 eq.) in toluene (0.5 mL) at room temperature was added catalyst (1.00 µmol, 10 mol-%) and the reaction was stirred at 100 °C. Repeatedly, aliquots of the reaction solution were diluted with EA, filtered through SiO_2 (EA), concentrated under reduced pressure and analyzed by qNMR (Table S7).

Conclusion: Since essentially no conversion occurred under all conditions, the more reactive TMS enol ether **13c** was evaluated next.

Table S7. Results for reaction condition C.

entry	catalyst	time	**13b**	**14a**	**10a**
1	$Pd_2(dba)_3$ + PdDavePhos	1 h	83%	87%	<5%
2		4 h	82%	86%	<5%
3		16 h	78%	65%	<5%
4	$Pd(P^tBu_3)_2$	1 h	84%	85%	<5%
5		4 h	79%	79%	<5%
6		16 h	66%	69%	<5%
7	$Pd(dppf)Cl_2$	1 h	84%	91%	<5%
8		4 h	79%	91%	<5%
9		16 h	-	-	<5%

Reaction Condition D: Initial Screening

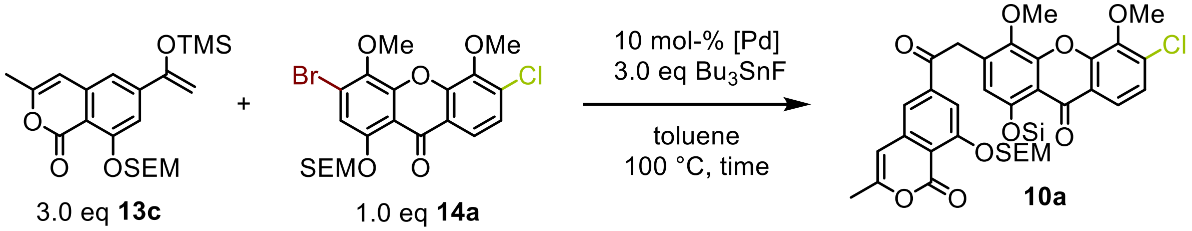

Procedure: To a mixture of **14a** (5.16 mg, 10.0 µmol, 1.00 eq.), **13c** (12.6 mg, 30.0 µmol, 3.00 eq.), Bu_3SnF (9.27 mg, 30.0 µmol, 3.00 eq.) and triphenylmethane as internal standard (2.44 mg, 10.0 µmol, 1.00 eq.) in toluene (0.5 mL) at room temperature was added catalyst (1.00 µmol, 10 mol-%) and the reaction was stirred at 100 °C. Repeatedly, aliquots of the reaction solution were diluted with EA, filtered through SiO_2 (EA), concentrated under reduced pressure and analyzed by qNMR (Table S8).

Conclusion: 38% **10a** (48% brsm **14a**) was obtained after 1 h using P^tBu_3 and Bu_3SnF (entry 4). Since this was the best result so far, variations of these conditions were evaluated next.

Table S8. Results for reaction condition D.

entry	catalyst	time	**13c**	**14a**	**10a**
1	$Pd_2(dba)_3$ +	1 h	98%	>95%	<5%
2	PdDavePhos	4 h	82%	86%	<5%
4	$Pd(P^tBu_3)_2$	1 h	62%	21%	38%
5		4 h	36%	14%	32%
7	$Pd(dppf)Cl_2$	1 h	77%	85%	<5%
8		4 h	76%	49%	<5%

Control Experiment: Base Stability of **13a**

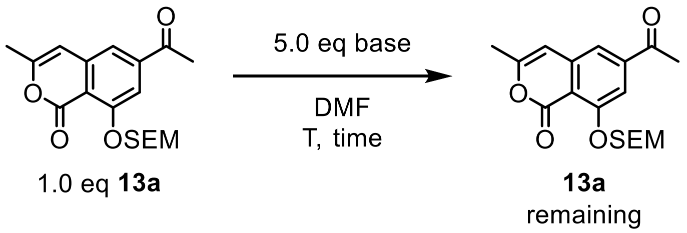

Procedure: A mixture of **13a** (3.49 mg, 10.0 µmol, 1.00 eq.) and base (50.0 µmol, 5.00 eq.) in DMF (0.5 mL) was stirred with a stepwise increase in temperature. At the end of each temperature step, an aliquot of the reaction solution was diluted with EA, filtered through SiO_2 (EA), concentrated under reduced pressure and analyzed by qNMR (Table S9).

Conclusion: Under basic conditions, **13a** decomposes even faster without catalyst and **14a** (compare to Table S5), resulting in severe decomposition within minutes (entry 1 and 4).

Table S9. Stability of **13a** against bases.

entry	base	T	time	**13a**
1		rt	45 min	30%
2	KOPh	60 °C	45 min	16%
3		100 °C	45 min	<5%
4		rt	45 min	<5%
5	KOH	60 °C	45 min	<5%
6		100 °C	45 min	<5%

Reaction Condition D-II: Temperature

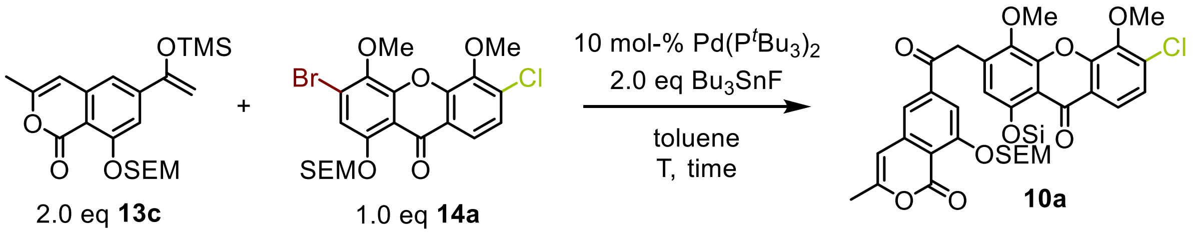

Procedure: To a mixture of **14a** (5.16 mg, 10.0 µmol, 1.00 eq.), **13c** (8.41 mg, 20.0 µmol, 2.00 eq.), Bu_3SnF (6.18 mg, 20.0 µmol, 2.00 eq.) and 1,3,5-trimethoxybenzene as internal standard (1.68 mg, 10.0 µmol, 1.00 eq.) in solvent (0.5 mL) at room temperature was added catalyst (1.00 µmol, 10 mol-%) and the reaction was stirred at 100 °C. Repeatedly, aliquots of the reaction solution were diluted with EA, filtered through SiO_2 (EA), concentrated under reduced pressure and analyzed by qNMR (Table S10).

Conclusion: A higher temperature with a shorter reaction time, improves conversion of **14a** to **10a** (compare entry 1-2 with entry 5 and 9). Decreasing the amount of **13c** and Bu_3SnF from three to two equivalents, decreases the yield of **10a** slightly (compare entry 2 with Table S8, entry 4). To increase comparability, further screenings were performed at 100 °C and 1 h, but the final coupling was performed at 103 °C and 30 min.

Table S10. Influence of temperature and time on result D-II.

entry	T	time	**13c**	**14a**	**10a**
1		15 min	60%	36%	28%
2	100 °C	1 h	55%	33%	33%
3		4 h	48%	26%	25%
4		15 min	82%	77%	6%
5	80 °C	1 h	77%	24%	27%
6		4 h	39%	21%	15%
7		15 min	72%	86%	<5%
8	60 °C	1 h	69%	81%	9%
9		4 h	64%	70%	11%

Reaction Condition D-II: Solvent and Additives

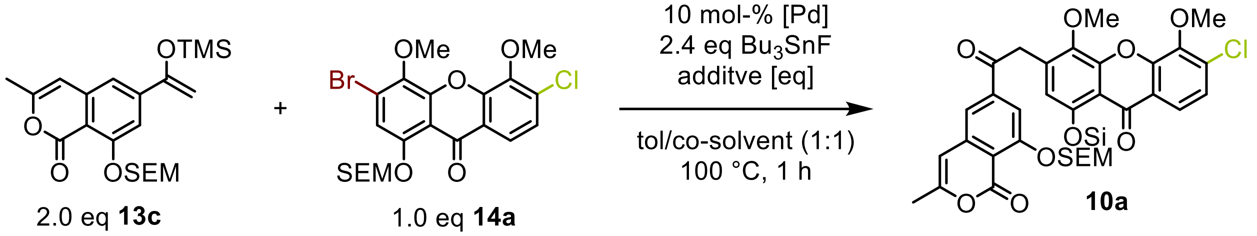

Procedure: To a mixture of **14a** (2.57 mg, 4.99 µmol, 1.00 eq.), **13c** (4.21 mg, 10.0 µmol, 2.00 eq.), Bu_3SnF (6.18 mg, 12.0 µmol, 2.40 eq.) and triphenylmethane as internal standard (2.44 mg, 10.0 µmol, 2.00 eq.) in co-solvent (0.25 mL) at room temperature was added catalyst (256 µg, 501 nmol, 10 mol-% in 0.25 mL toluene) and the reaction was stirred at 100 °C for 1 h. Then, the reaction solution was filtered through SiO_2 (EA), concentrated under reduced pressure and analyzed by qNMR (Table S11).

Conclusion: $Pd_2(dba)_3$ in toluene was optimal, providing 56% (65% brsm **14a**) **10a** (entry 10). This suggest that mono-ligation is critical for conversion. To test this hypothesis, both mono-ligated (PdL_1) and bis-ligated catalyst were investigated (PdL_2).

Table S11. Influence of solvent and additives on result D-II.

entry	additive	co-solvent	**13c**	**14a**	**10a**
1	CsF 5.00 eq	toluene	<5%	<5%	<5%
2		dioxane	<5%	<5%	<5%
3		DMF	<5%	<5%	<5%
4	LiCl 10.0 eq	toluene	52%	34%	24%
5		dioxane	-	22%	1%
6		DMF	20%	<5%	13%
7	CuI 30 mol-%	toluene	82%	80%	3%
8		dioxane	92%	82%	2%
9		DMF	<5%	13%	37%
10	$Pd_2(dba)_3$ 5.0 mol-%	toluene	50%	14%	56%
11		dioxane	55%	22%	50%
12		DMF	<5%	<5%	34%

Reaction Condition D-II: Catalyst

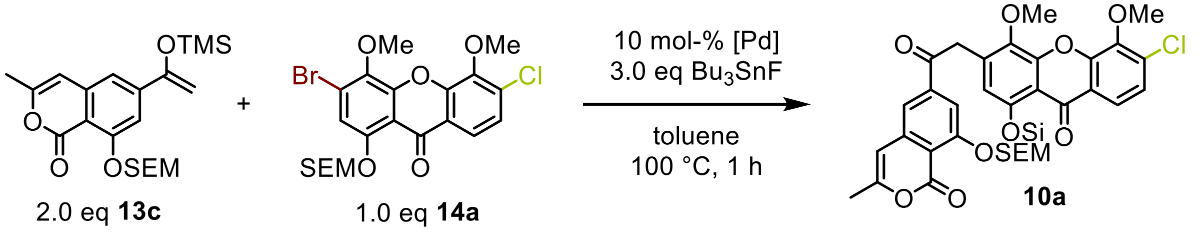

Procedure: To a mixture of **14a** (2.57 mg, 4.99 µmol, 1.00 eq.), **13c** (4.21 mg, 10.0 µmol, 2.00 eq.), Bu_3SnF (4.64 mg, 15.0 µmol, 3.00 eq.) and triphenylmethane as internal standard (2.44 mg, 10.0 µmol, 2.00 eq.) in toluene (0.4 mL) at room temperature was added catalyst (1.00 µmol, 10 mol-% Pd in 100 µL toluene) and the reaction was stirred at 100 °C for 1 h. Then, the reaction solution was filtered through SiO_2 (EA), concentrated under reduced pressure and analyzed by qNMR (Table S12).

Conclusion: A minimum buried volume over 34% ensures good conversion (compare entry 1-2 to entry 3-6). An equimolar catalyst improves conversion further (entry 3-4). A type **II** ligand performs better than a type **I** ligand (entry 6-7). Overall, P^tBu_3 and QPhos performed the best (entry 4 and 6). In the next step, the catalyst loading was investigated.

Table S12. Influence of catalyst and stoichiometry on reaction condition D.

entry	Type	Pd:L	ligand	V_{bur}(min)	catalyst*	**13c**	**14a**	**10a**
1			Pcy_3	30%	A	>95%	93%	<5%
2	**II**	PdL_2	P(*o*-tol$_3$)	34%	B	91%	80%	16%
3			P^tBu_3	36%	B	52%	-	31%
4			P^tBu_3	36%	C	53%	26%	48%
5	**II**	PdL_1	PAd_3	37%	D	40%	-	34%
6			QPhos	48%	D	32%	20%	50%
7	**I**	PdL_1	HandaPhos	44%	D	72%	49%	13%

* A: PdL_2Cl_2, B: PdL_2, C: PdL_2 + $Pd_2(dba)_3$, D: $Pd_2(dba)_3$ + L.

Reaction Condition D-II: Catalyst Loading

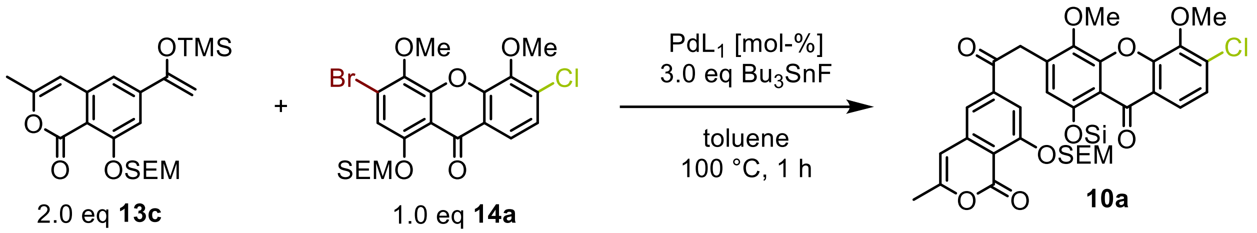

Procedure: To a mixture of **14a** (2.57 mg, 4.99 µmol, 1.00 eq.), **13c** (4.21 mg, 10.0 µmol, 2.00 eq.), Bu_3SnF (4.64 mg, 15.0 µmol, 3.00 eq.) and triphenylmethane as internal standard (2.44 mg, 10.0 µmol, 2.00 eq.) in toluene (0.5 mL total) at room temperature was added PdL_1 catalyst (500 nmol, 10 mol-% Pd per 100 µL toluene) and the reaction was stirred at 100 °C for 1 h. Then, the reaction solution was filtered through SiO_2 (EA), concentrated under reduced pressure and analyzed by qNMR (Table S13).

Conclusion: 10 to 20 mol-% of either P^tBu_3 or QPhos were optimal, providing 48-52% **10a** (entry 2-3 and 5-6). In the end, P^tBu_3 was used because of its lower cost and 11 mol-% palladium were employed.

Table S13. Influence of catalyst loading on reaction condition D-II.

entry	catalyst	[Pd]	**13c**	**14a**	**10a**
1	$Pd(P^tBu_3)_2$ + $Pd_2(dba)_3$	5 mol-%	68%	53%	21%
2		10 mol-%	53%	26%	48%
3		20 mol-%	38%	8%	52%
4	QPhos + $Pd_2(dba)_3$	5 mol-%	82%	61%	16%
5		10 mol-%	32%	20%	50%
6		20 mol-%	49%	13%	50%

Control Experiment: Fluoride Salt Stability of **13c**

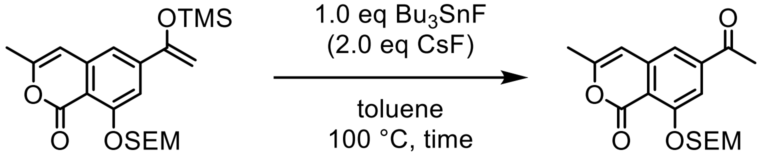

1.0 eq **13c** **13a**

Procedure: A mixture of **13c** (2.10 mg, 5.00 µmol, 1.00 eq.), Bu_3SnF (1.54 mg, 4.98 µmol, 1.00 eq.) either with our without cesium fluoride (1.52 mg, 10.0 µmol, 2.00 eq.) in toluene (0.5 mL) was heated to 100 °C. Repeatedly, aliquots of the reaction solution were diluted with EA, filtered through SiO_2 (EA), concentrated under reduced pressure and analyzed by NMR (Figure S6).

Conclusion: Without cesium fluoride, **13c** is inert against Bu_3SnF on the reaction time scale (entry 1-2).

entry	additive	time	**13c**	**13a**
1	Bu_3SnF	15 min	>95%	<5%
2		1 h	>95%	<5%
3	Bu_3SnF	15 min	93%	7%
4	CsF	1 h	39%	61%

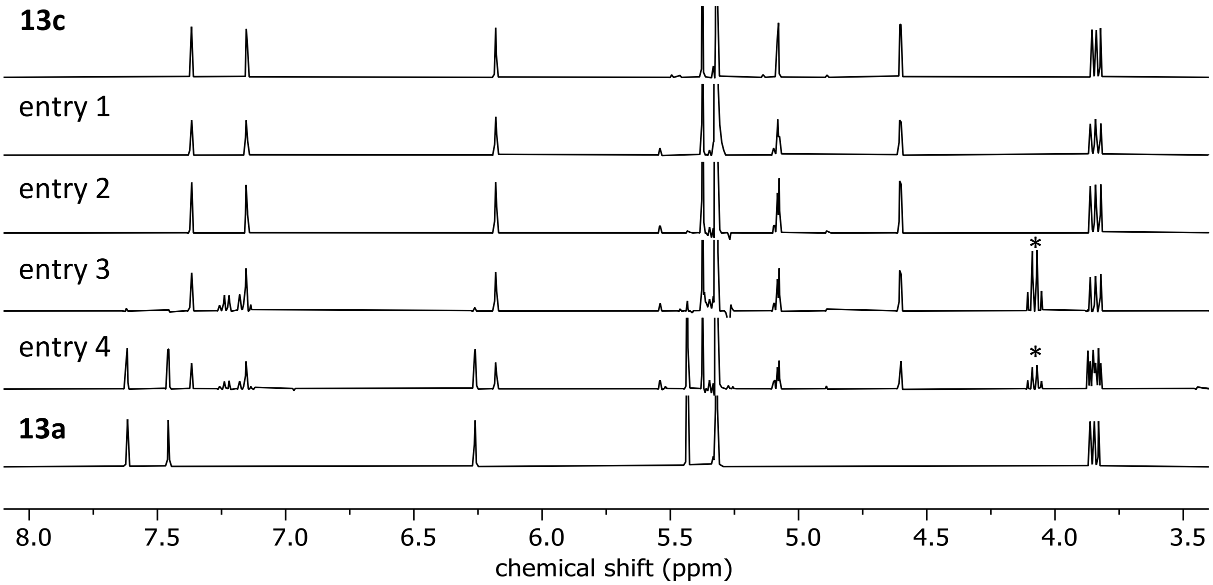

Figure S6. Stability of **13c** against fluoride salts measured in CD_2Cl_2. * Ethyl acetate.

Scale-up: Palladium to Ligand Ratio

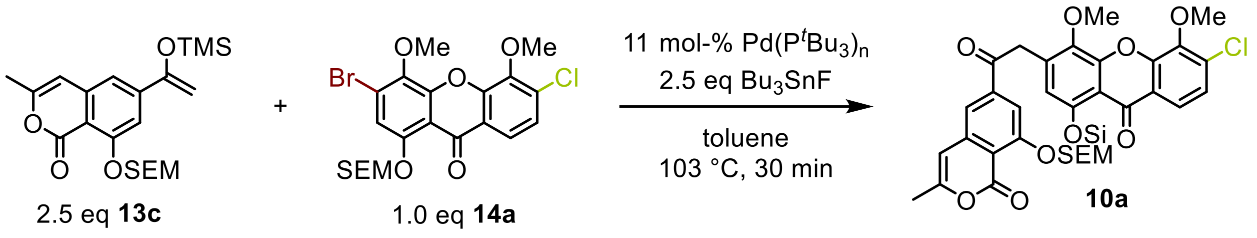

Procedure: See synthesis of coupled **3**, results in Table S14.

Conclusion: A P^{t}Bu$_3$ to palladium ratio of 1.05 was optimal (entry 2).

Table S14. Influence of palladium to ligand ratio on isolated yields.

entry	P^{t}Bu$_3$: Pd	**13c**	**14a**	**10a**
1	1.03	46%	17%	46%
2	1.05	41%	<5%	62%
3	1.09	48%	13%	53%

3.4 Enantioselective Ketone Reduction (**11a**)

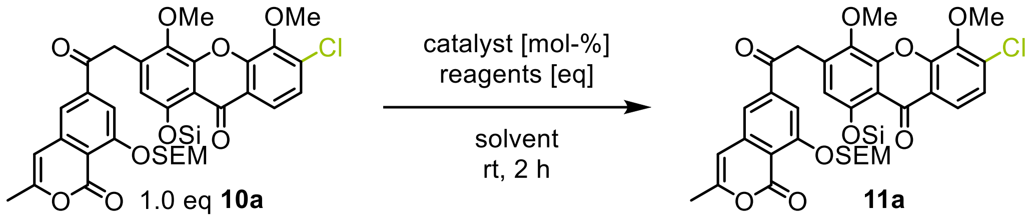

Procedure CBS: To a solution of ketone **10a** (2.00 mg, 2.55 µmol, 1.00 eq.) and 1,3,5-trimethoxybenzene as internal standard (429 µg, 2.55 µmol, 1.00 eq.) in THF (0.1 mL) was added (*S*)-2-methyl-CBS-oxazaborolidin (1.00 M in THF) and $BH_3 \cdot Me_2S$ (100 mM in THF, 38.3 µL, 3.83 µmol, 1.50 eq.) and the reaction was stirred at room temperature for 2 h. Then, the reaction was filtered through a SPE silica plug (MTBE) and concentrated under reduced pressure. The yield was determined by qNMR and after preparative TLC (SiO_2, cy/EA 1:1) the enantiopurity was analyzed by chiral HPLC (Table S15, entry 1-2).

Procedure Noyori: See synthesis of alcohol **11a** (Chapter 2.3).

Conclusion: Noyori reduction is both higher yielding and more selective, making it the preferred method (entry 3).

Table S15. Results of enantioselective ketone reduction.

entry	catalyst	loading	reagents	**10a**	**11a**	ee
1	(*S*)-CBS	10 mol-%	1.50 eq $BH_3 \cdot SMe_2$	<5%	87%	56%
2		100 mol-%		<5%	85%	76%
3	(*R,R*)-[Ru]	10 mol-%	6.00 eq HCO_2H, 10.0 eq NEt_3	<5%	>95%	90%

3.5 Late Stage Aminolysis (**33**)

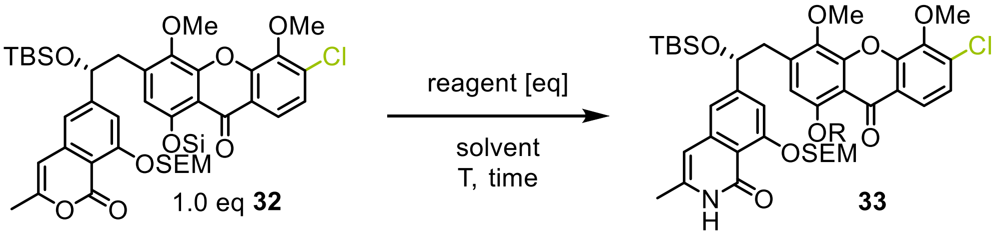

Procedure Ammonium Acetate: To a solution of **32** (4.50 mg, 5.00 µmol, 1.00 eq.) and 1,3,5-trimethoxybenzene as internal standard (841 µg, 5.00 µmol, 1.00 eq.) in DMSO (0.6 mL) was added NH_4OAc (1.16 mg, 15.0 µmol, 3.00 eq.) and the reaction was stirred at 100 °C for 20 h. Then, saturated citric acid (2 mL) was added and the water layer was extracted with MTBE (5×2 mL). The combined organic layers were concentrated under reduced pressure and analyzed by qNMR (Table S16, entry 1).

Procedure Ammonia: A solution of **32** (4.40 mg, 4.89 µmol, 1.00 eq.) in methoxyethanol (0.6 mL) was purged with NH_3 gas for 15 min. The reaction was tightly sealed and heated for 1 h. Then, saturated citric acid (2 mL) was added, the water layer was extracted with EA (5×2 mL) and each organic layer was filtered over the same SPE silica plug. The combined organic layers were concentrated under reduced pressure, 1,3,5-trimethoxybenzene as internal standard (1.62 µg, 9.63 µmol, 2.00 eq.) was added and the product analyzed by qNMR (Table S16, entry 2-3).

Conclusion: NH_3 gas and high temperature ensures good conversion (entry 3). Later, the reaction temperature was slightly lowered to 138 °C to improve selectivity.

Table S16. Results of late stage aminolysis.

entry	reagents	time	T	**32**	**33**
1	3.00 eq NH_4OAc	20 h	100 °C	94%	<5%
2	excess NH_3	1 h	120 °C	50%	26%
3			140 °C	<5%	67%

3.6 Catalytic Davis Oxidation (42)

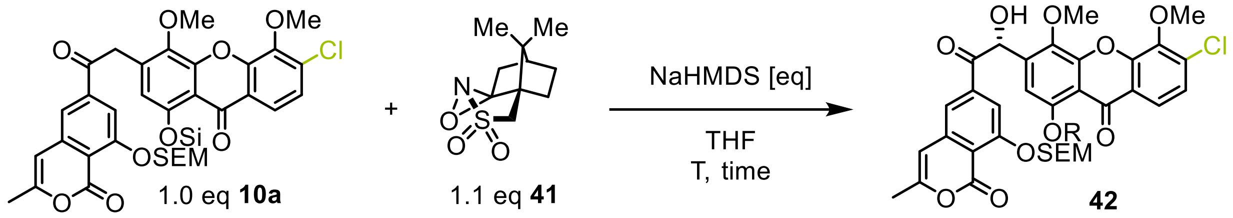

Procedure: To a solution of **10a** (4.30 mg, 5.49 µmol, 1.00 eq.) and 1,3,5-trimethoxybenzene as internal standard (302 µg, 1.80 µmol, 0.33 eq.) in THF (0.2 mL) at cryogenic temperature was added NaHMDS (cryogenic stock solution in THF, 100 µL). The deprotonation was stirred at cryogenic temperature for 2 min, **41** (1.41 mg, 6.15 µmol, 1.12 eq.) was added and the reaction was stirred as stated. Then, pH 7 buffer (0.5 mL) was added, the water layer was extracted with EA (3×0.5 mL) and each organic layer was filtered over the same SPE silica plug. The combined organic layers were concentrated under reduced pressure and analyzed by qNMR. (Table S17)

To a solution of crude **42** and pyridine (3 drops) in DCM (0.5 mL) was added MTPA-Cl (1 drop) and the reaction was stirred at room temperature for 16 h. Then, pH 7 buffer (0.5 mL) was added, the water layer was extracted with EA (3×0.5 mL) and each organic layer was filtered over the same SPE silica plug. The combined organic layers were concentrated under reduced pressure and the enantiomeric excess was determined by the NMR ratio of the Mosher esters.

Conclusion: A catalytic amount of NaHMDS and short reaction time is essential for product formation (entry 4). Later, the amount of NaHMDS was halved to improve selectivity.

Table S17. Results of α-hydroxylation.

entry	NaHMDS	T	time	**10a**	**42**	ee
1	100 mol-%	-78 °C	30 min	78%	3%	-
2		-40 °C	30 min	40%	6%	-
3		-90 °C → rt	5 min	<5%	8%	-
4	10 mol-%	-90 °C → rt	5 min	<5%	98%	66%
5			15 min	<5%	93%	52%

4 NMR Comparisons

4.1 Comparison of CBS100 (**5**)

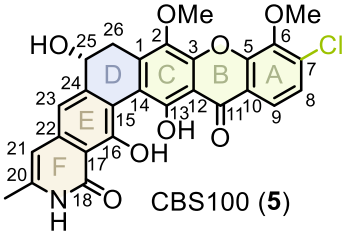

CBS100 (**5**)

Table S18. NMR comparison natural and synthetic CBS100 (**5**).

#	^{1}H				^{13}C		
	Ref. 8	298 K	348 K	re-crystallized	Ref. 8	298 K	348 K
1	-				140.0	140.0	139.7
2	-				135.8	135.7	136.0
2-OMe	3.95	3.95	3.97	3.95	61.6	61.6	61.3
3	-				147.3	147.3	147.0
5	-				149.7	149.8	149.4
6	-				144.6	144.6	144.4
6-OMe	4.12	4.13	4.15	4.13	61.6	61.6	61.2
7	-				133.3	133.3	132.9
8	7.60	7.60	7.58	7.61	125.3	125.3	125.0
9	7.94	7.95	7.96	7.96	120.8	120.5	120.4
10	-				120.5	120.8	120.3
11	-				181.2	181.2	180.8
12	-				107.5	107.5	107.2
13	-				153.3	153.2	153.0
13-OH	12.94	12.93	12.84	12.93	-		
14	-				114.0	114.1	114.1
15	-				112.0	112.0	112.0
16	-				157.5	157.5	157.4

16-OH	13.85	13.85	13.79	13.84		-	
17			-		108.7	109.0	108.2
18			-		166.9	166.9	166.6
18-NH	11.79	11.77	11.56	11.75		-	
20			-		138.8	138.8	138.5
20-Me	2.26	2.26	2.28	2.27	18.6	18.6	18.2
21	6.52	6.52	6.48	6.51	105.5	105.4	105.0
22			-		138.6	138.6	138.3
23	7.19	7.18	7.15	7.18	108.7	108.7	109.0
24			-		149.8	149.8	149.4
25	4.48	4.49	4.58	4.50	67.4	67.5	67.0
25-OH	5.95	5.94	5.57	5.91		-	
26	2.45/3.34	2.46/3.41	2.72/3.24	2.45/3.40	33.1	33.0	32.5

4.2 Comparison of CBS87 (**4**)

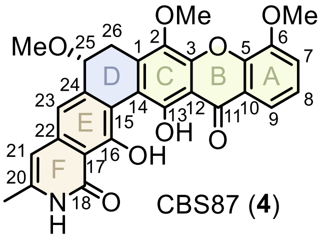

CBS87 (**4**)

Table S19. NMR comparison natural and synthetic CBS87 (**4**).

#	^{1}H				^{13}C		
	Ref. 8	298 K	348 K	re-crystallized	Ref. 8	298 K	348 K
1		-				not observed	
2		-			137.1	136.6	138.2
2-OMe	3.95	3.95	3.99	3.96	61.3	61.3	60.9
3		-			147.5	147.5	147.1
5		-			145.5	145.5	145.5
6		-			148.4	148.4	148.3
6-OMe	4.04	4.04	4.06	4.04	56.6	56.7	56.6
7	7.59	7.59	7.58	7.59	117.2	117.3	117.4
8	7.46	7.46	7.45	7.46	124.4	124.5	124.0
9	7.77	7.77	7.79	7.78	115.9	115.9	115.8
10		-			120.7	120.7	120.5
11		-			181.8	181.8	181.4
12		-			107.5	107.5	107.3
13		-			153.1	153.1	152.8
13-OH	13.12	13.11	13.00	13.10		-	
14		-			113.6	113.4	113.5
15		-			not observed	112.8	112.6
16		-			158.3	158.2	157.8
16-OH	13.88	13.87	13.80	13.87		-	

17	-				110.3	110.8	110.5
18	-				166.9	166.9	166.6
18-NH	11.85	11.83	11.57	11.81	-		
20	-				138.7	138.9	138.4
20-Me	2.27	2.27	2.28	2.27	18.6	18.6	18.1
21	6.52	6.51	6.48	6.51	105.2	105.2	104.9
22	-				not observed		146.7
23	7.07	7.07	7.07	7.08	113.2	114.6	144.6
24	-				not observed		
25	4.45	4.45	4.40	4.44	76.5	75.5	76.0
25-OMe	3.15	3.15	3.34	3.17	55.7	55.8	55.6
26	2.66/3.62	2.65/3.63	overlaps	2.66/3.65	29.7	29.6	28.7

4.3 Comparison of CBS72 (**3**)

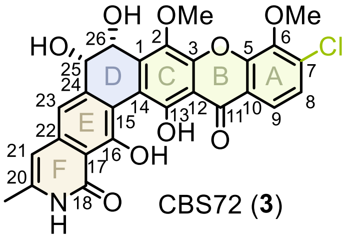

CBS72 (**3**)

Table S20. NMR comparison natural and synthetic CBS72 (**3**).

#	^{1}H			^{13}C	
	Ref. 8	**3**	re-crystallized	Ref. 8	**3**
1		-		145.6	145.6
2		-		136.1	136.1
2-OMe	4.03	4.04	4.04	62.7	62.7
3		-		147.5	147.5
5		-		149.9	149.9
6		-		144.6	144.6
6-OMe	4.13	4.13	4.14	61.6	61.6
7		-		133.4	133.4
8	7.61	7.62	7.62	125.3	125.3
9	7.96	7.96	7.97	120.9	120.9
10		-		120.5	120.5
11		-		181.4	181.4
12		-		108.6	108.6
13		-		153.3	153.3
13-OH	12.88	12.87	12.86	-	
14		-		113.4	113.4
15		-		112.0	112.0
16		-		157.2	157.2
16-OH	13.83	13.82	13.82	-	

17		-		109.0	109.0
18		-		166.9	166.9
18-NH	11.74	11.72	11.70	-	
20		-		138.4	138.4
20-Me	2.26	2.27	2.27	18.6	18.6
21	6.50	6.50	6.49	105.4	105.4
22		-		138.7	138.7
23	7.20	7.20	7.20	111.1	111.1
24		-		140.3	140.3
25	4.52	4.52	4.52	71.1	71.1
25-OH	5.73	-	5.70	-	
26	5.05	5.05	5.05	64.9	64.9
26-OH	5.24	-	5.19	-	

5 Idealities

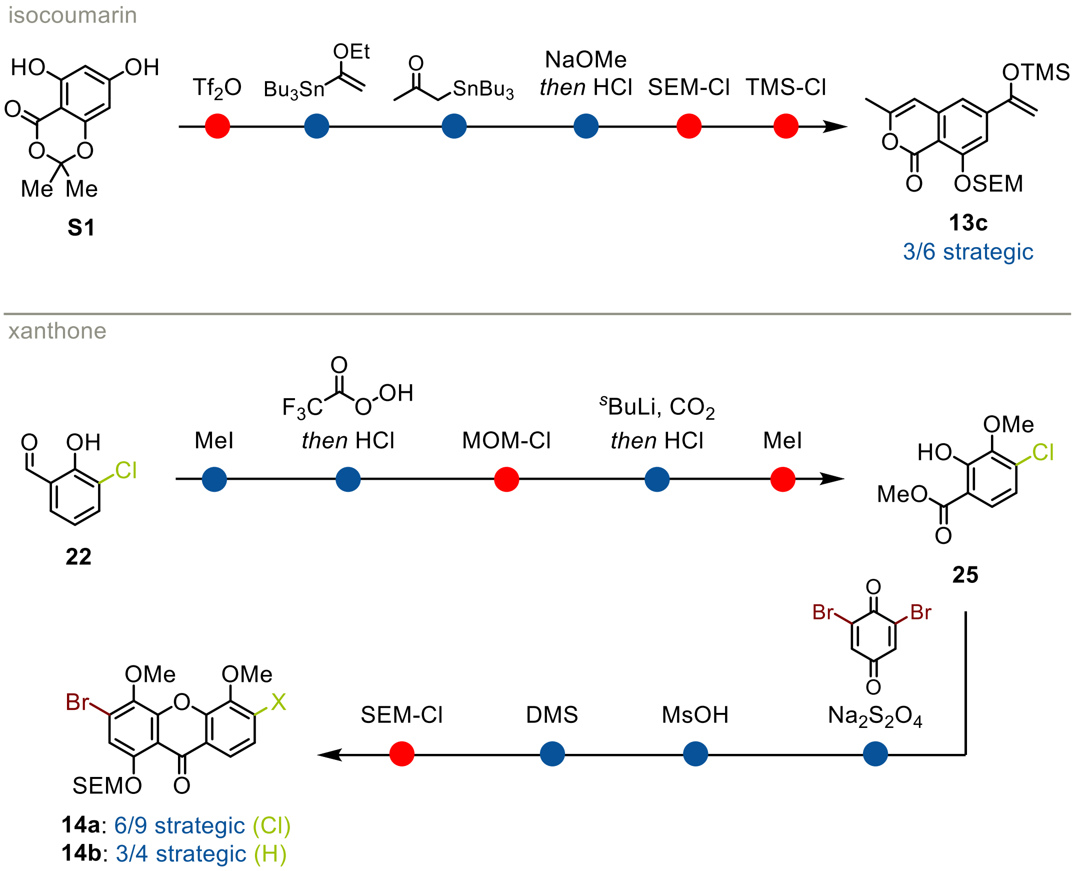

Figure S7. Ideality of fragment syntheses.

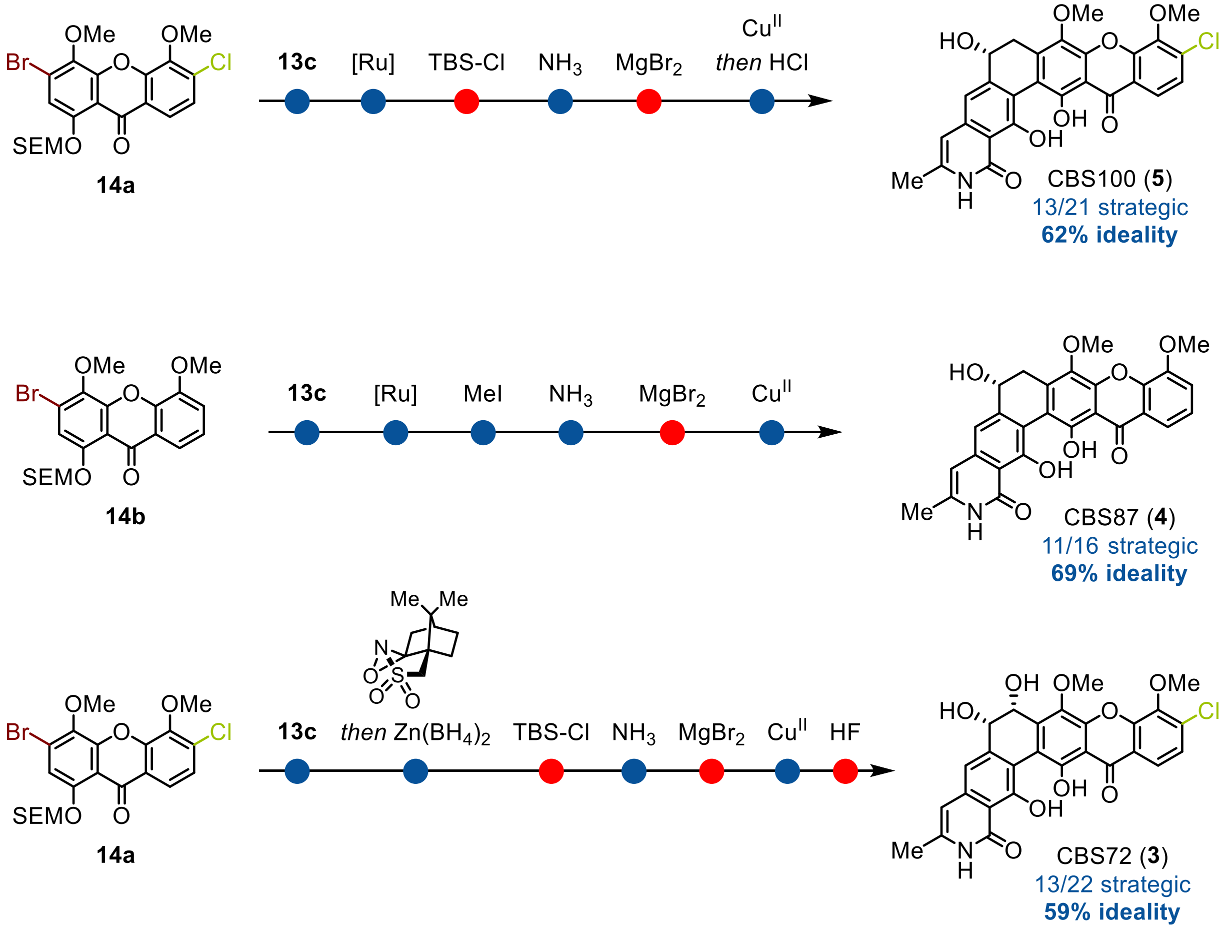

Figure S8. Ideality of complete total syntheses.

6 Chromatograms

6.1 Leading to Isocoumarin **13**

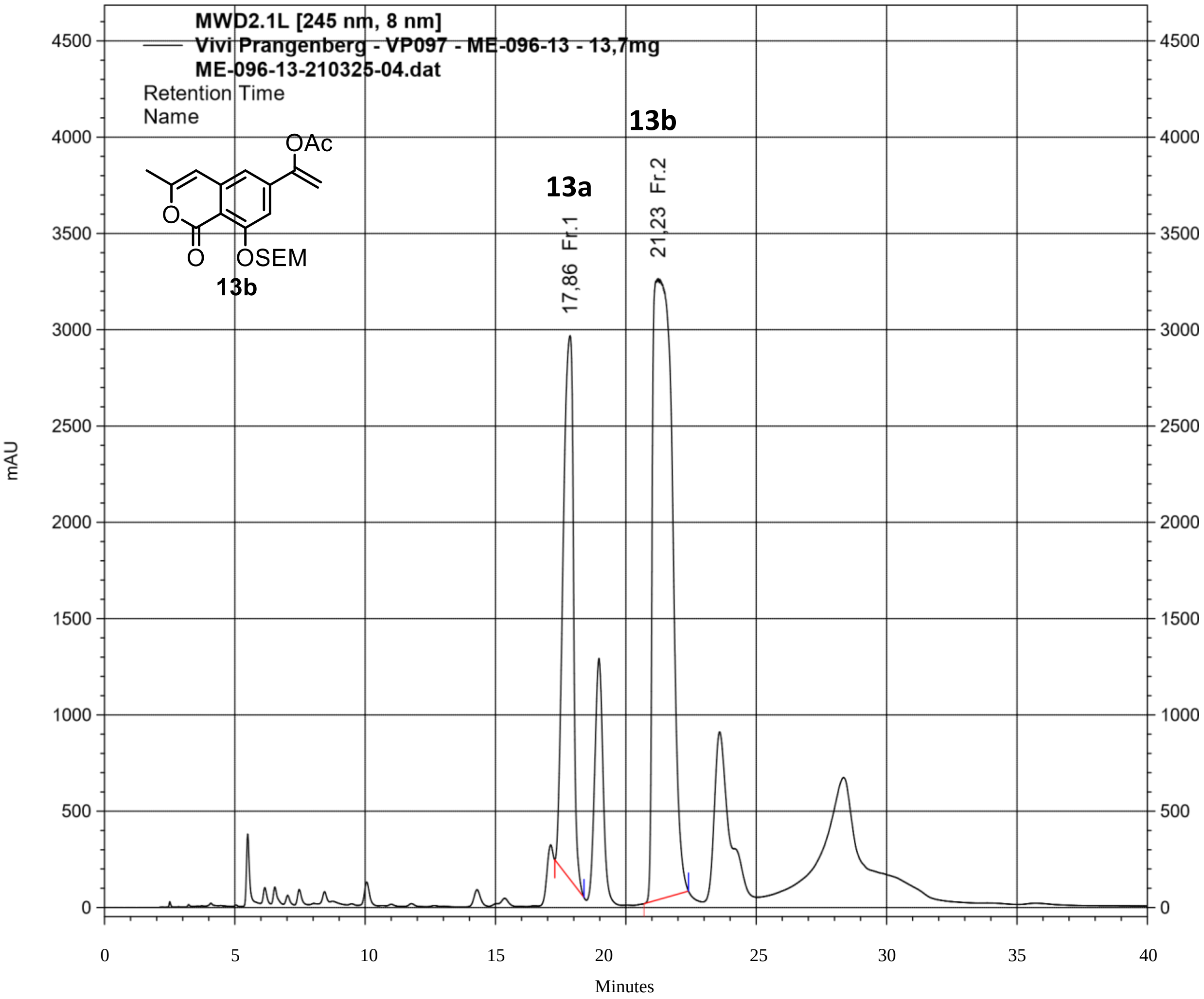

6.2 Leading to CBS100 (**5**)

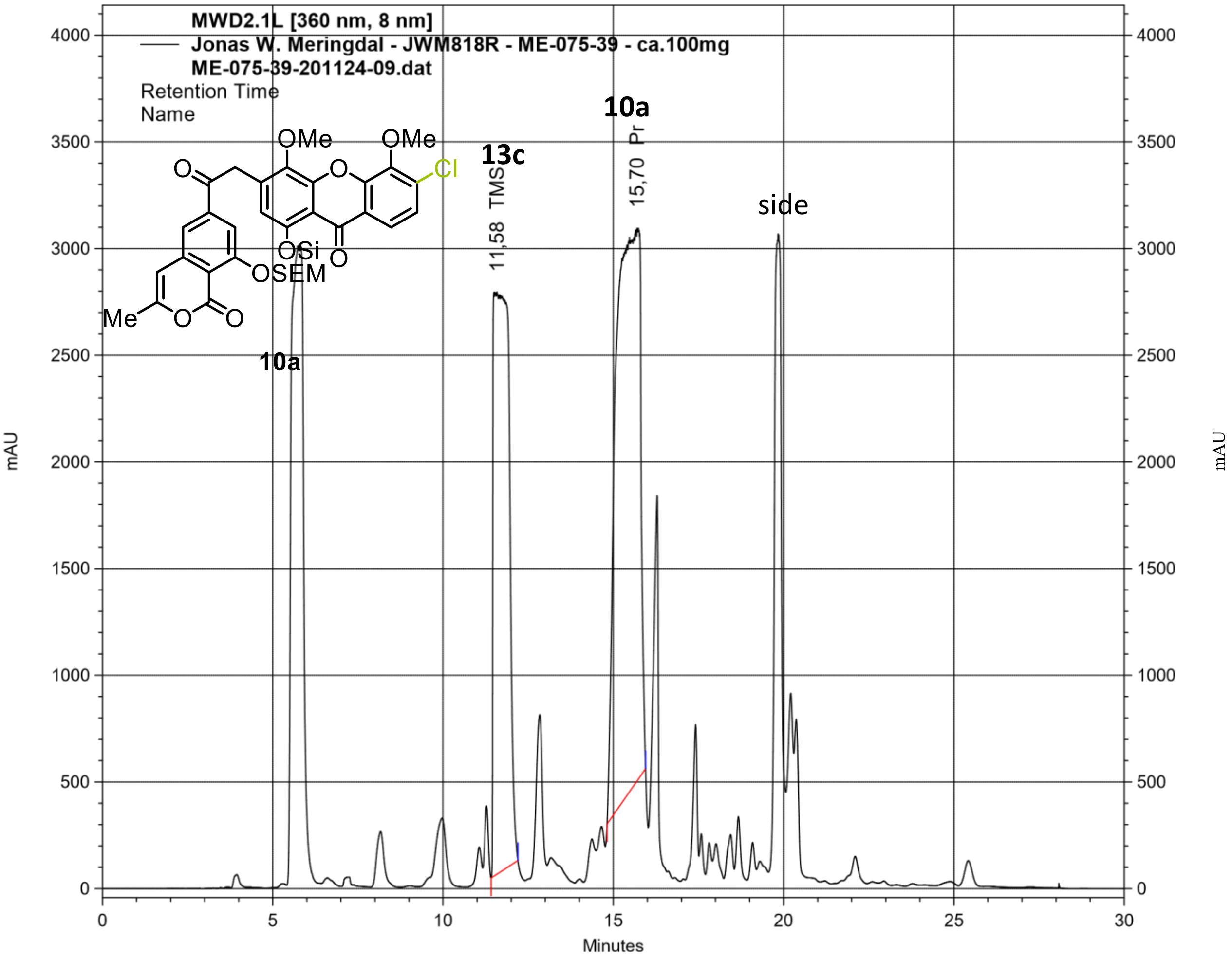

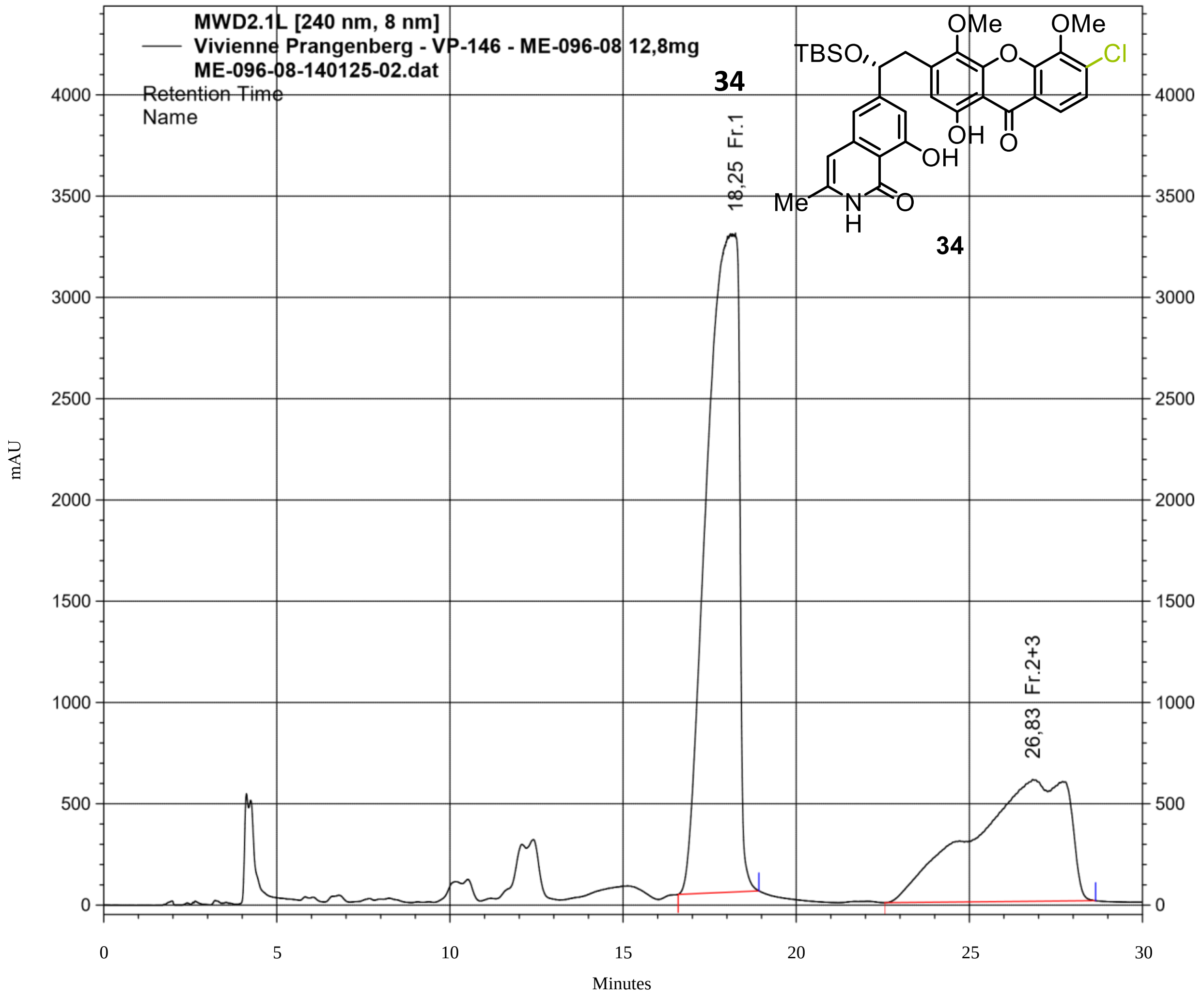
MWD2.1L [240 nm, 8 nm]
Vivienne Prangenberg - VP-146 - ME-096-08 12,8mg
ME-096-08-140125-02.dat
Retention Time
Name
34
18,25 Fr.1
26,83 Fr.2+3
TBSO
OMe
OMe
Cl
OH
OH
O
Me
N
H
O
34
mAU
Minutes

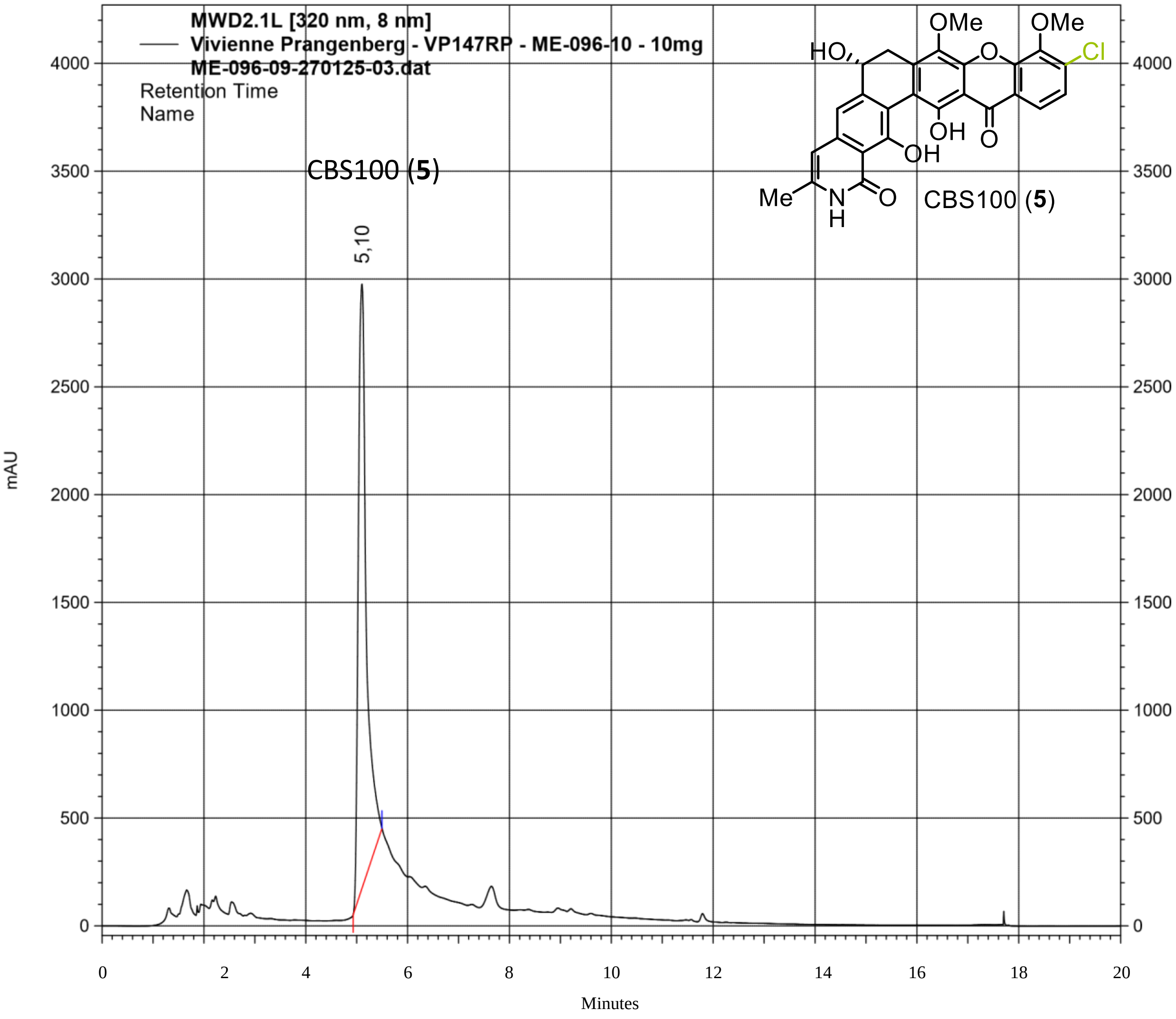
MWD2.1L [320 nm, 8 nm]
Vivienne Prangenberg - VP147RP - ME-096-10 - 10mg
ME-096-09-270125-03.dat
Retention Time
Name
CBS100 (5)
5,10
OMe
OMe
HO
O
Cl
OH
OH
O
Me
N
H
O
CBS100 (5)
mAU
Minutes

6.3 Leading to CBS87 (**4**)

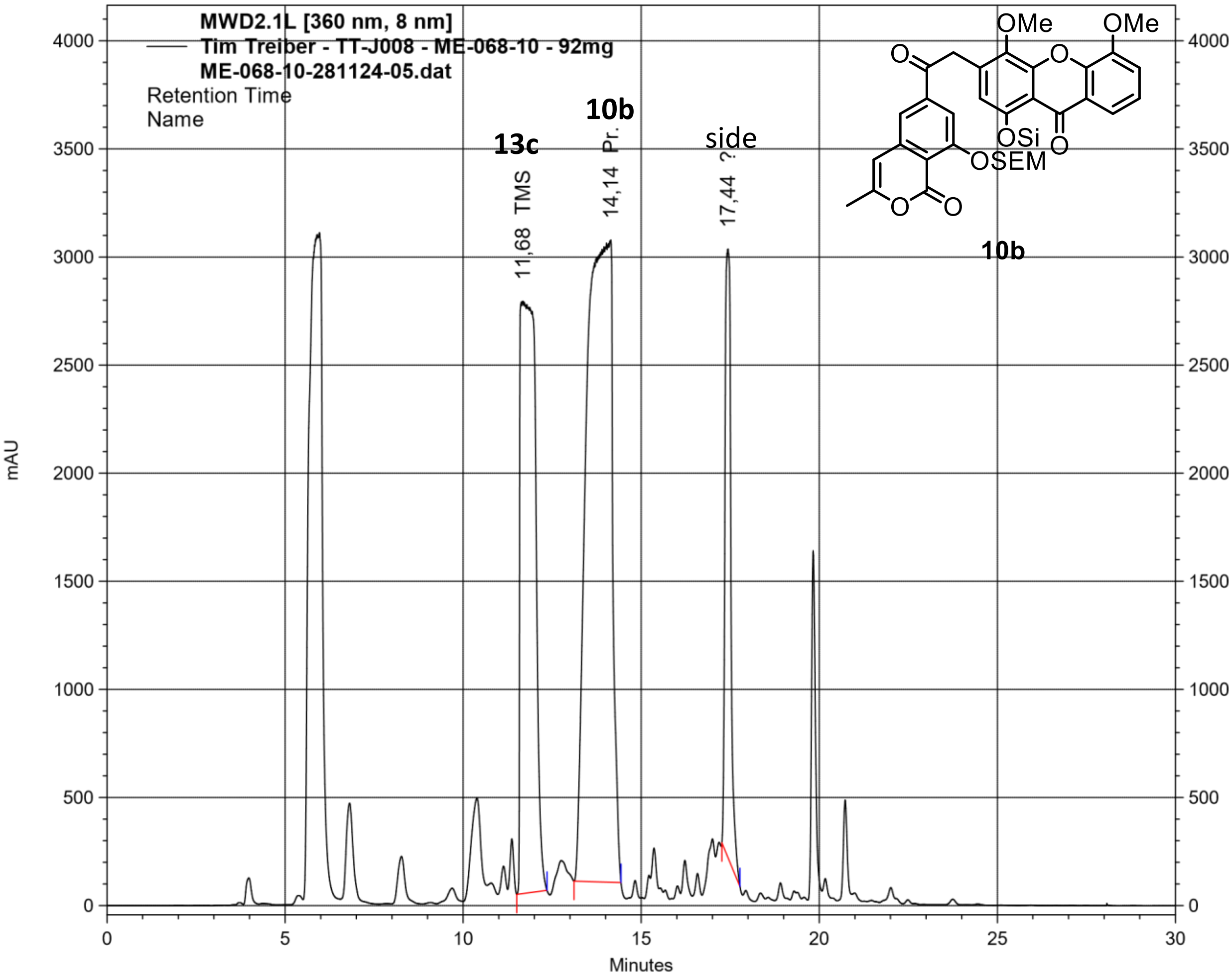

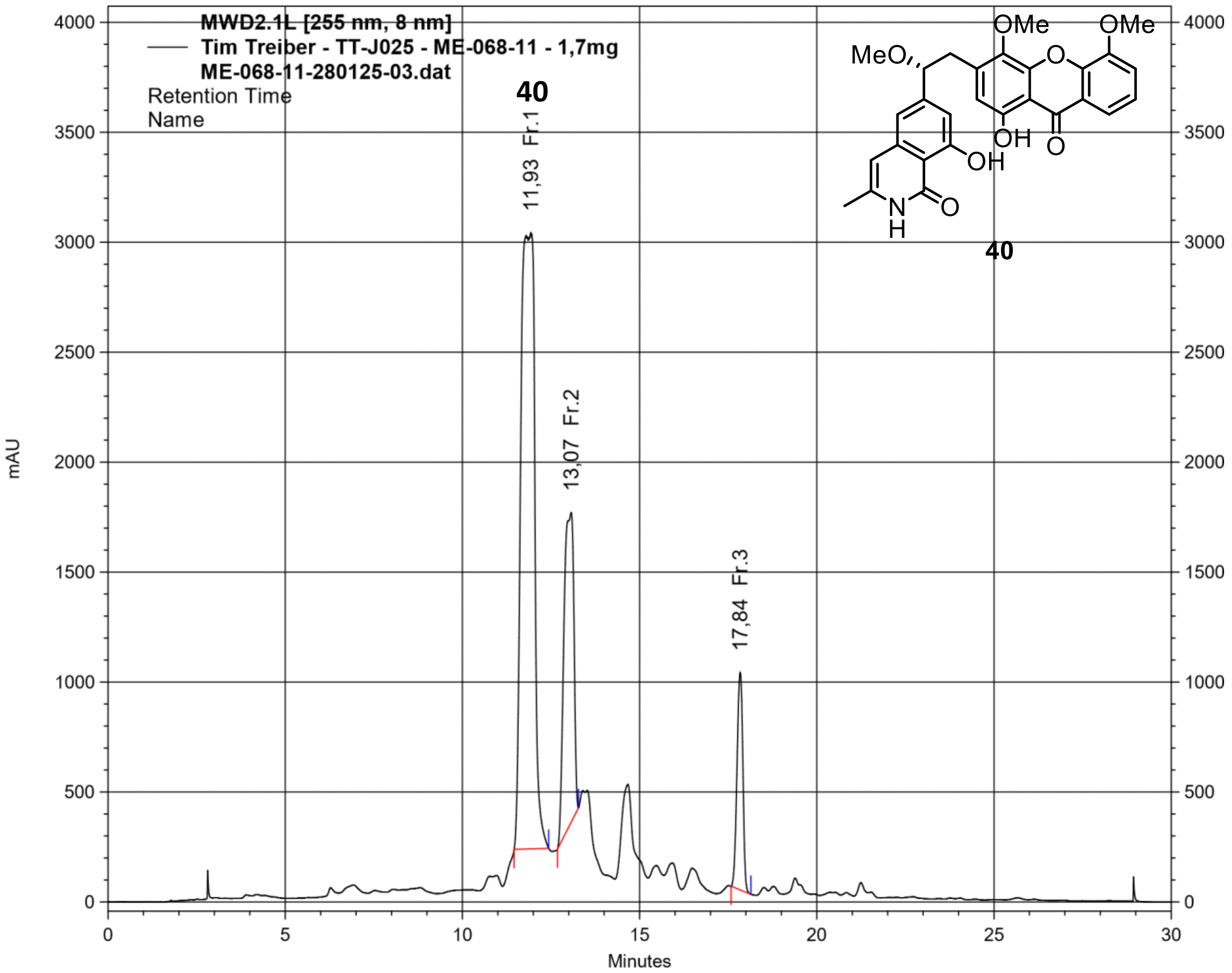
MWD2.1L [255 nm, 8 nm]
Tim Treiber - TT-J025 - ME-068-11 - 1,7mg
ME-068-11-280125-03.dat
Retention Time
Name
40
11,93 Fr.1
13,07 Fr.2
17,84 Fr.3
mAU
Minutes
40

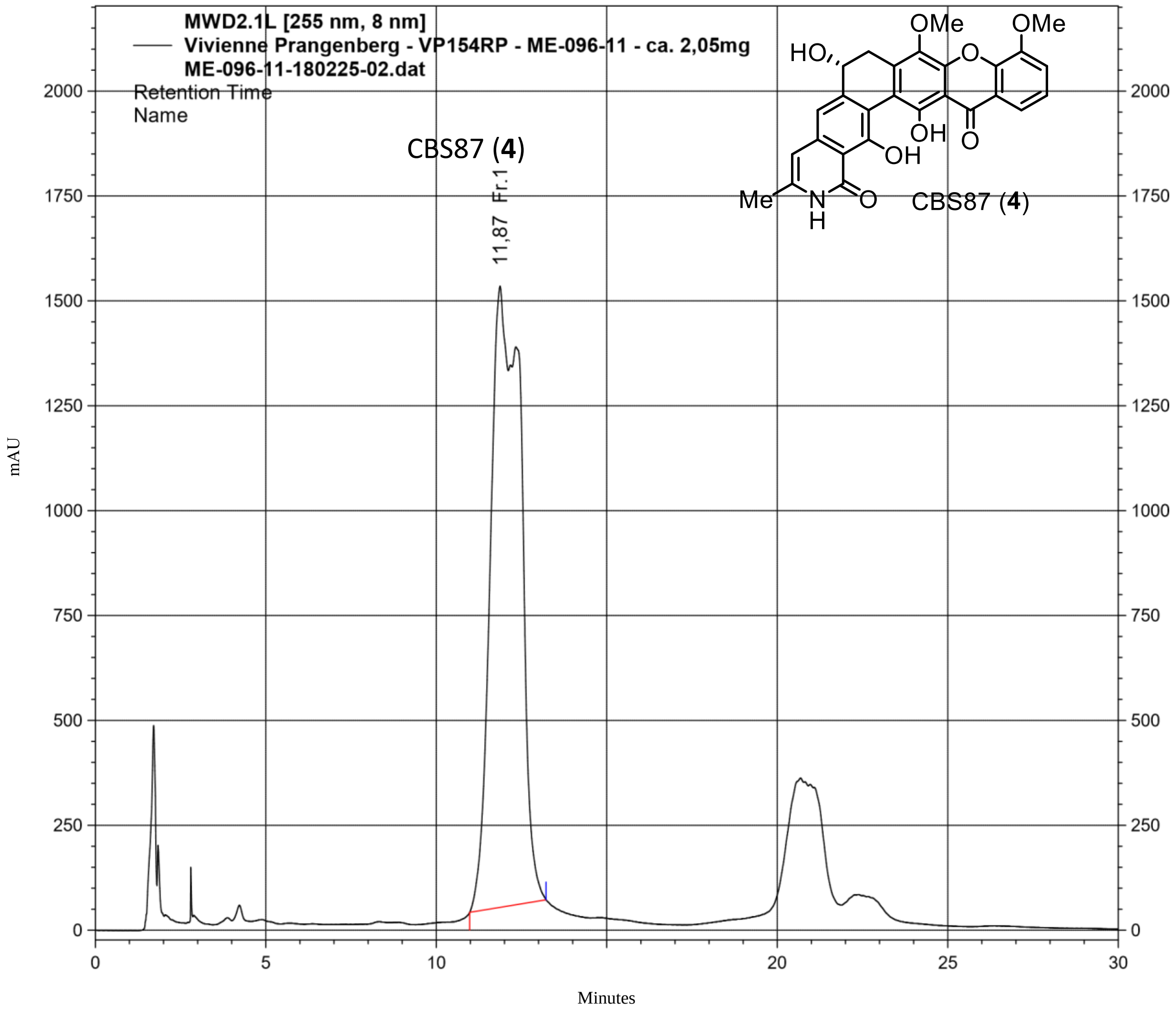
MWD2.1L [255 nm, 8 nm]
Vivienne Prangenberg - VP154RP - ME-096-11 - ca. 2,05mg
ME-096-11-180225-02.dat
Retention Time
Name
CBS87 (**4**)
11,87 Fr.1
HO
OMe
OMe
O
OH
O
OH
Me
N
H
O
CBS87 (**4**)
mAU
Minutes
0
250
500
750
1000
1250
1500
1750
2000
5
10
20
25
30

6.4 Leading to CBS72 (**3**)

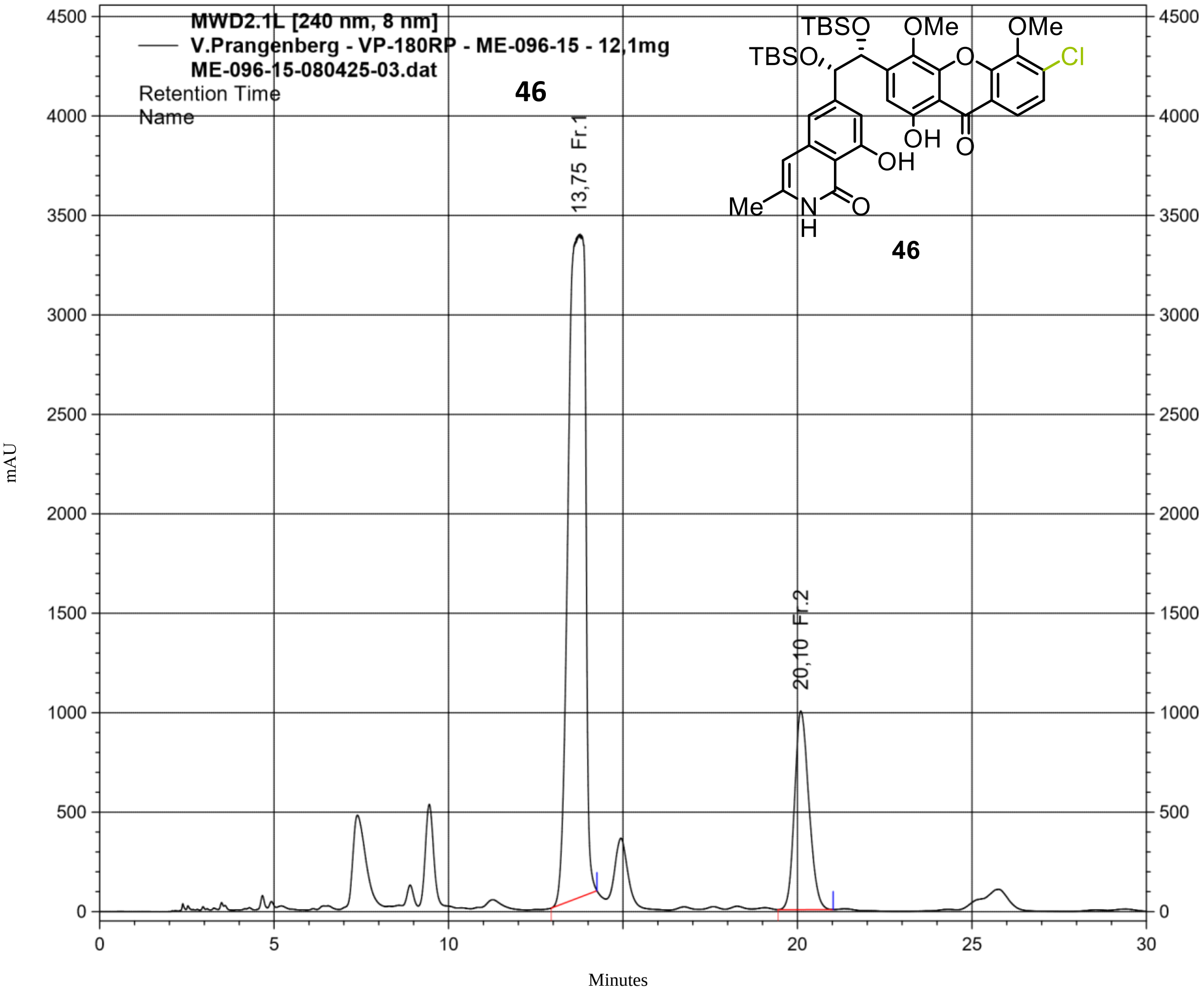

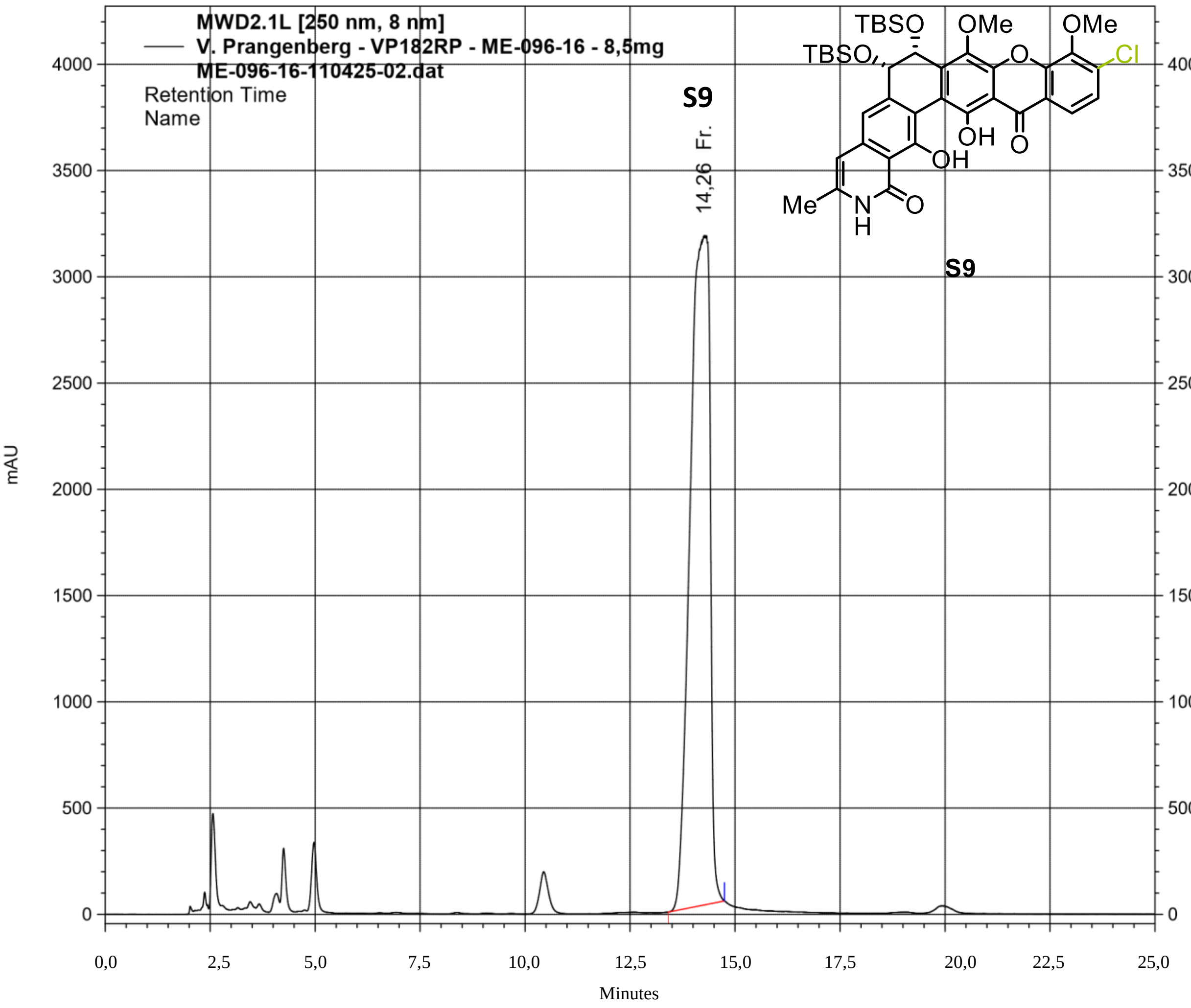

MWD2.1L [250 nm, 8 nm]
V. Prangenberg - VP182RP - ME-096-16 - 8,5mg
ME-096-16-110425-02.dat
Retention Time
Name
S9
14,26 Fr.
TBSO
TBSO
OMe
OMe
Cl
O
OH
OH
O
Me
N
H
O
S9
mAU
Minutes

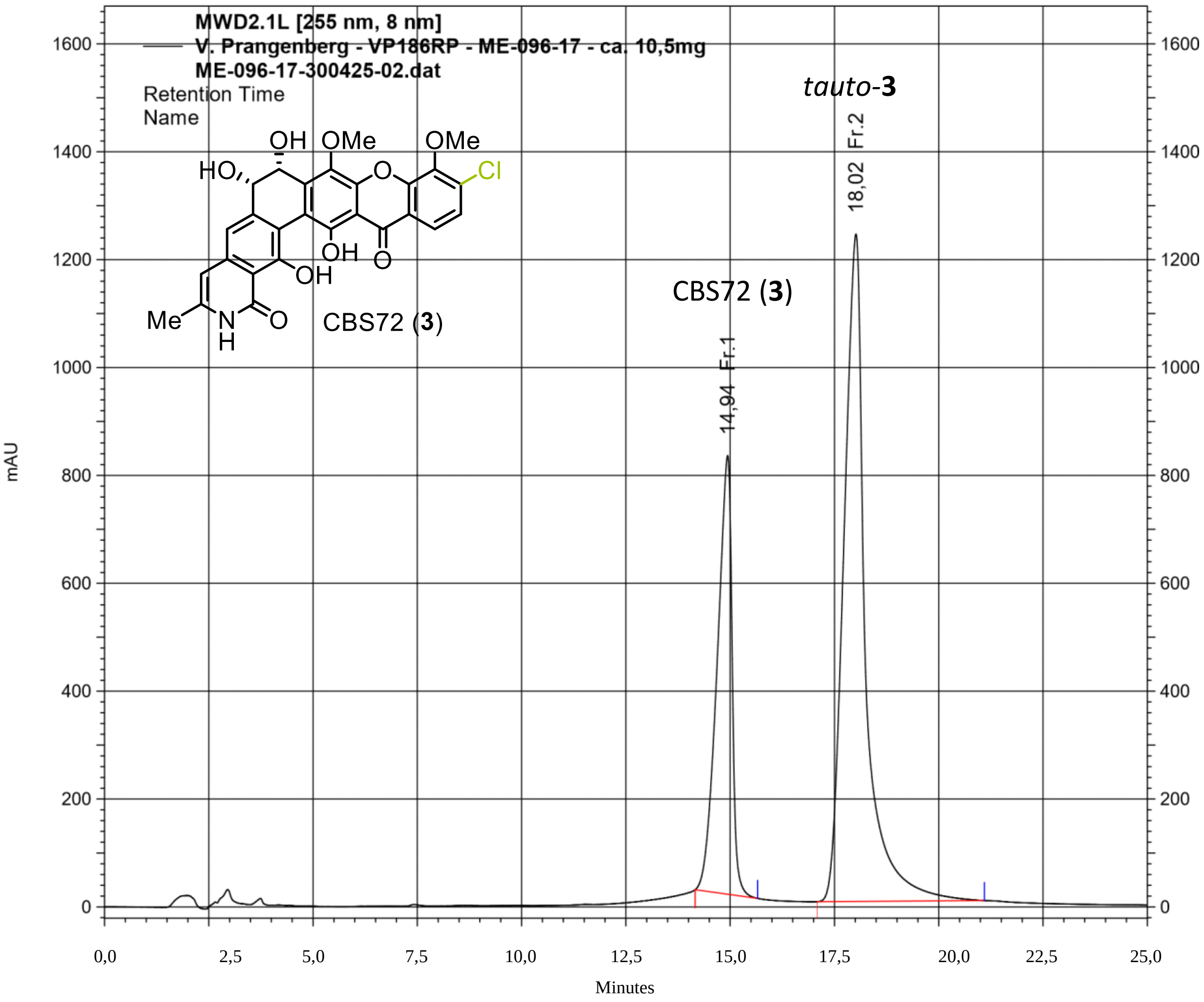
MWD2.1L [255 nm, 8 nm]
V. Prangenberg - VP186RP - ME-096-17 - ca. 10,5mg
ME-096-17-300425-02.dat
Retention Time
Name
CBS72 (3)
tauto-3
14,94 Fr.1
18,02 Fr.2
CBS72 (3)
mAU
Minutes

6.5 Additional Chromatograms

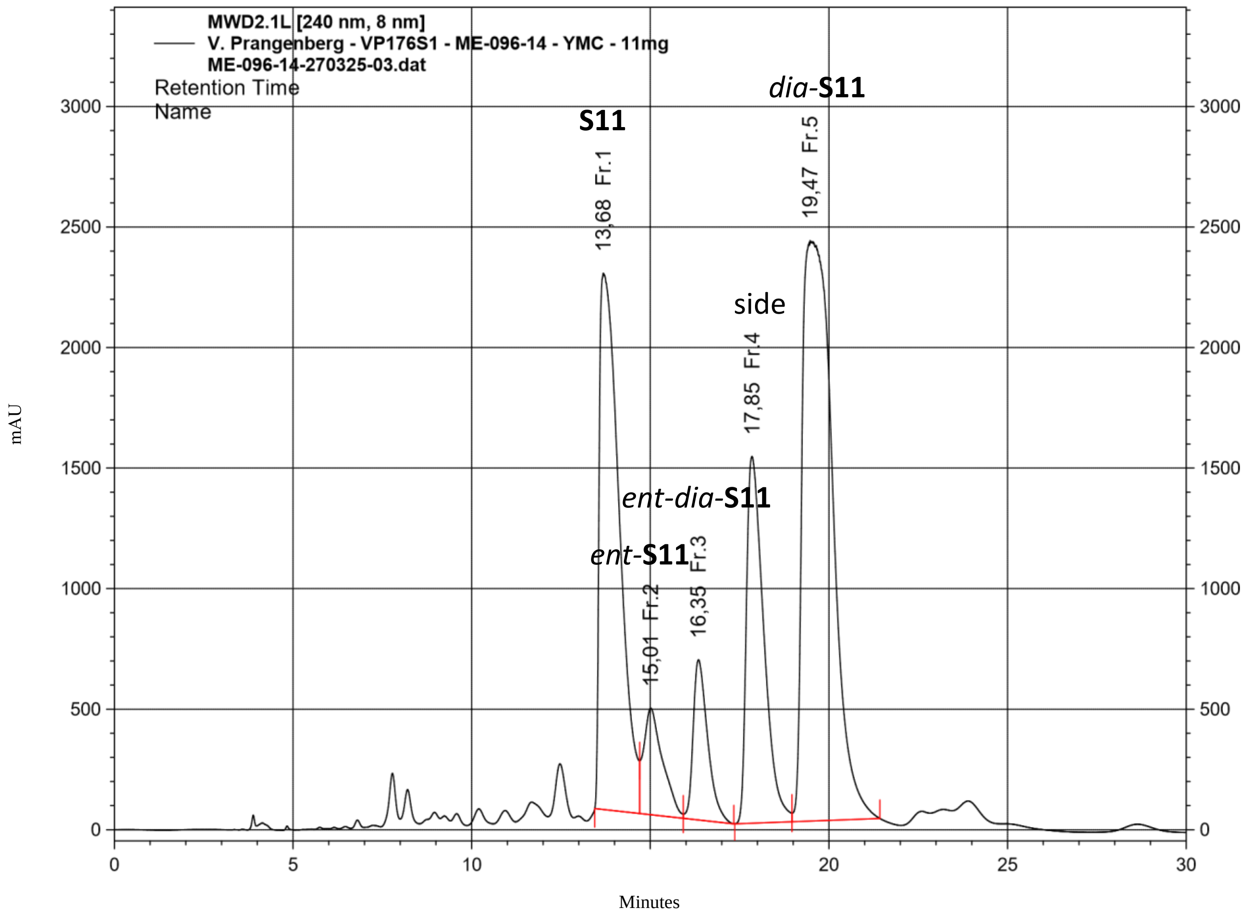

6.6 Enantiomeric Excess

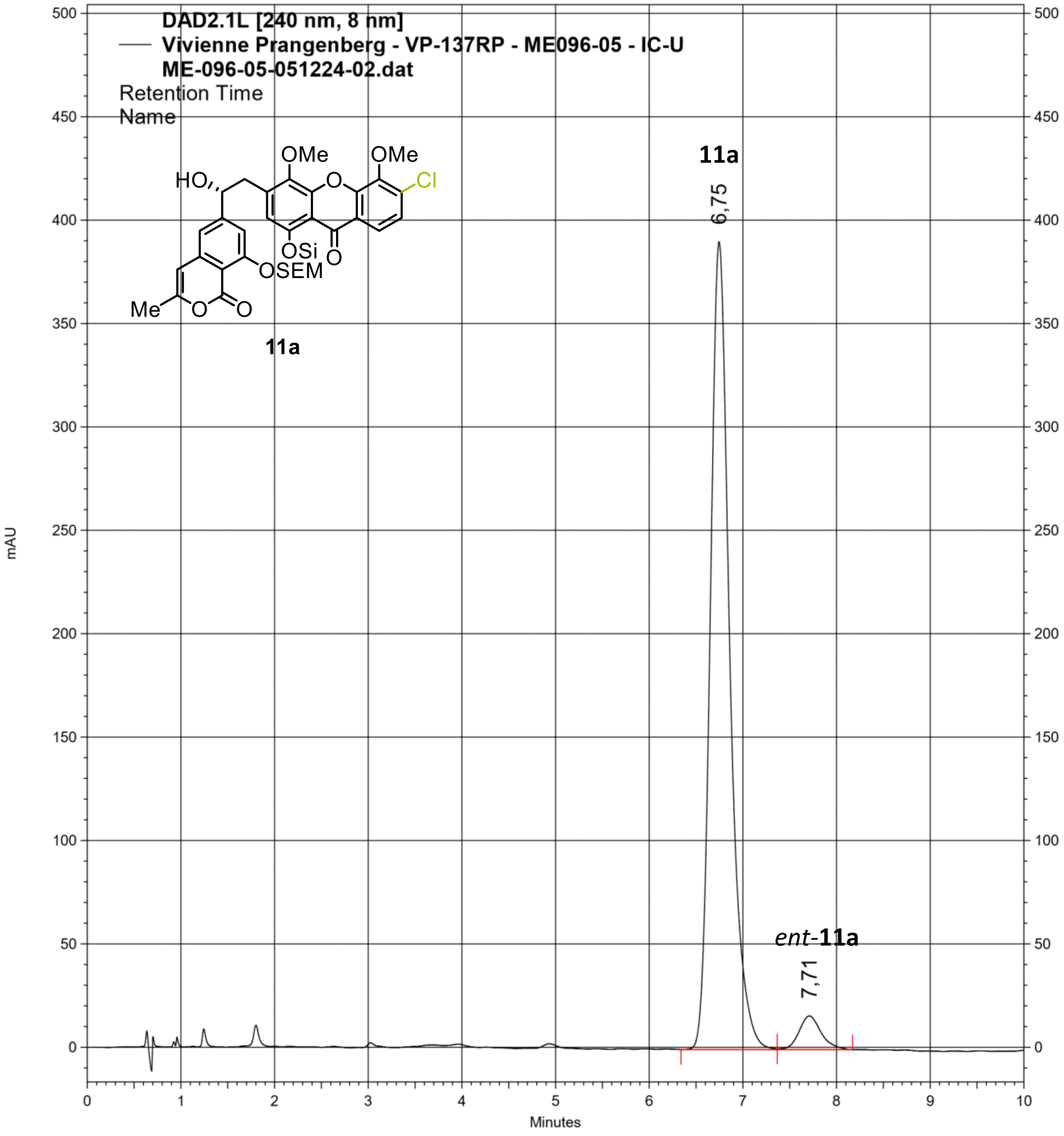

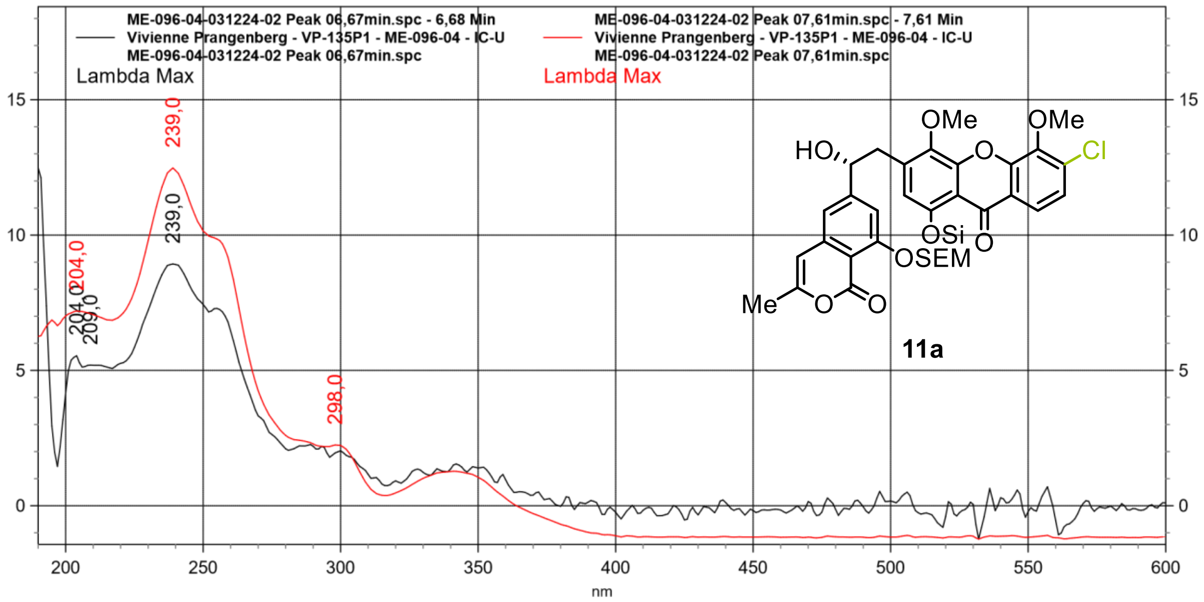

nm, 8 nm] Results

Retention Time	Area	Area %	Height	Height %
6,746	560753236	95,44	39069004	96,00
7,711	26782335	4,56	1629144	4,00
Totals	587535571	100,00	40698148	100,00

DAD2.1L [295 nm, 8 nm] Results

Retention Time	Area	Area %	Height	Height %
6,746	142545733	95,46	9928622	96,04
7,707	6781580	4,54	409724	3,96
Totals	149327313	100,00	10338346	100,00

DAD2.1L [340 nm, 8 nm] Results

Retention Time	Area	Area %	Height	Height %
6,745	102187199	95,69	7128220	96,10
7,699	4597275	4,31	289252	3,90
Totals	106784474	100,00	7417472	100,00

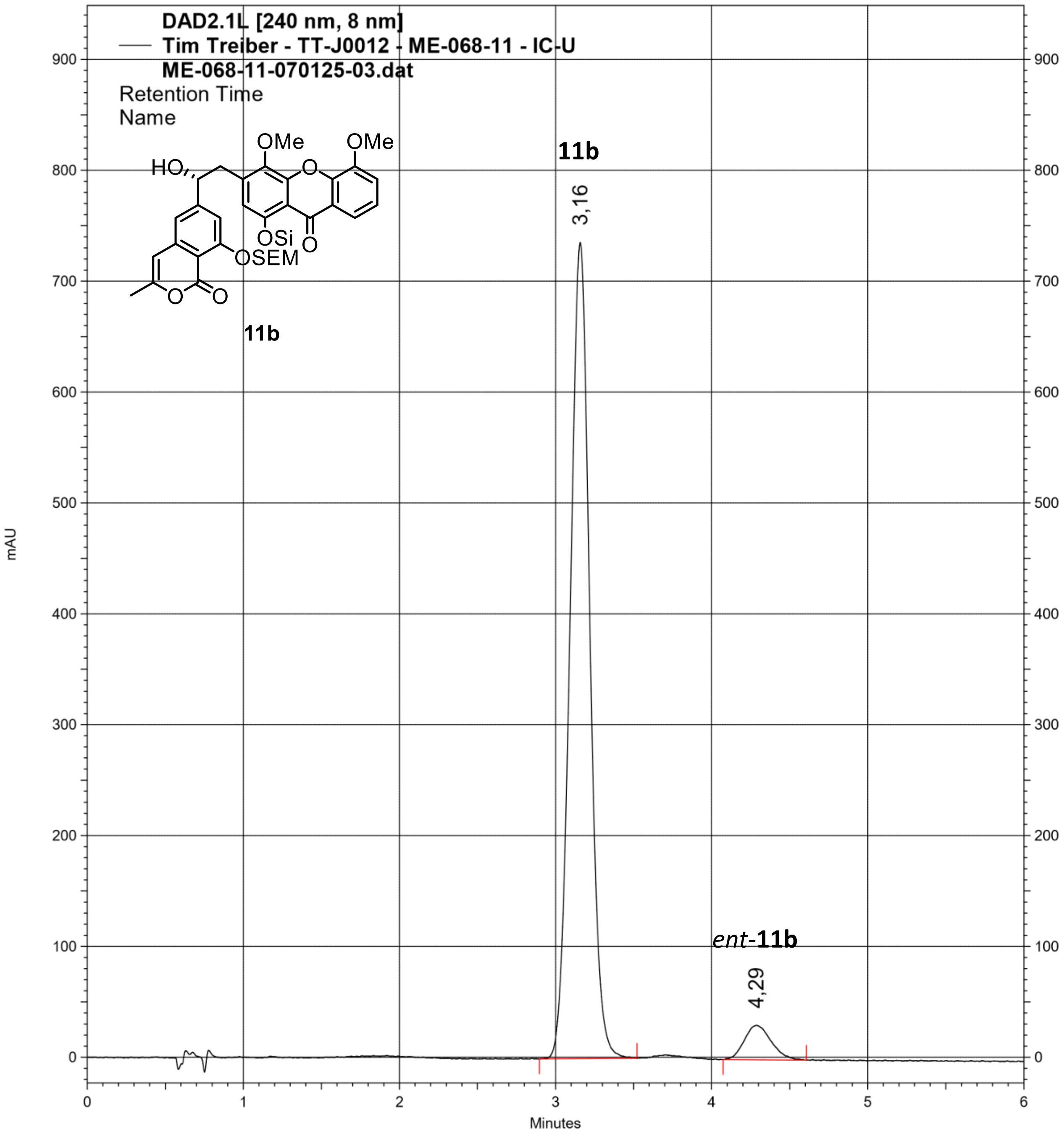
DAD2.1L [240 nm, 8 nm]
Tim Treiber - TT-J0012 - ME-068-11 - IC-U
ME-068-11-070125-03.dat
Retention Time
Name
HO
OMe
OMe
OSi
OSEM
11b
11b
3,16
ent-11b
4,29
mAU
Minutes

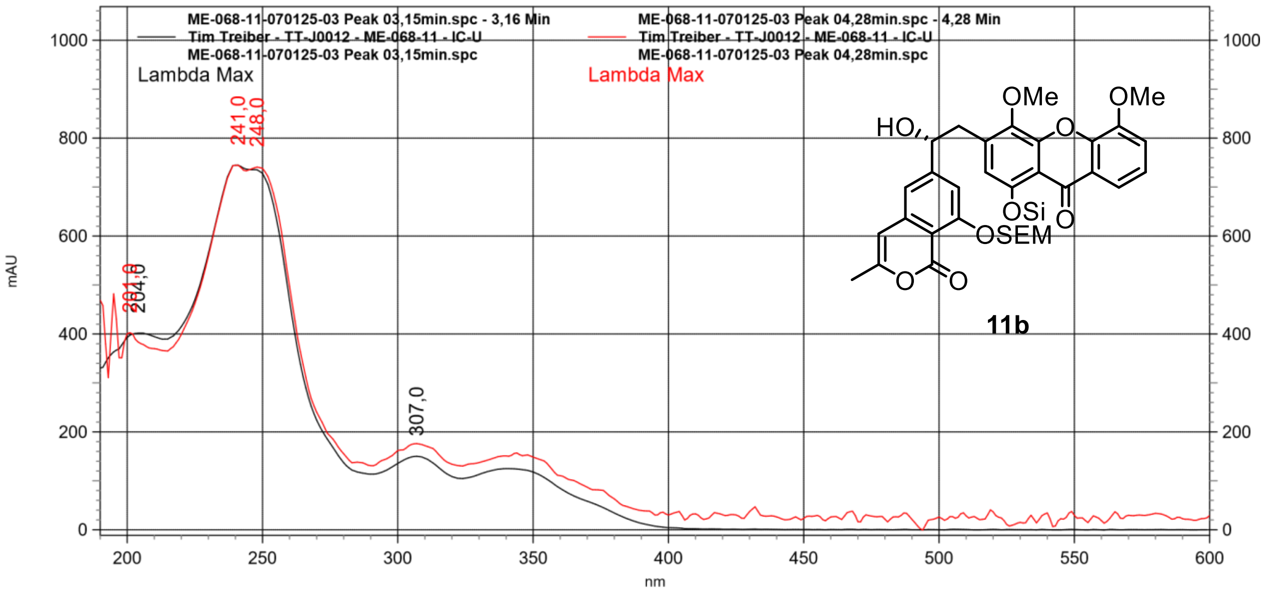

nm, 8 nm] Results

Retention Time	Area	Area %	Height	Height %
3,158	642801599	94,47	73614440	95,94
4,286	37621655	5,53	3116035	4,06
Totals	680423254	100,00	76730475	100,00

DAD2.1L [305 nm, 8 nm] Results

Retention Time	Area	Area %	Height	Height %
3,158	128096822	94,64	14686728	95,98
4,285	7250215	5,36	615382	4,02
Totals	135347037	100,00	15302110	100,00

DAD2.1L [340 nm, 8 nm] Results

Retention Time	Area	Area %	Height	Height %
3,159	108468123	94,60	12437722	95,96
4,284	6191588	5,40	523106	4,04
Totals	114659711	100,00	12960828	100,00

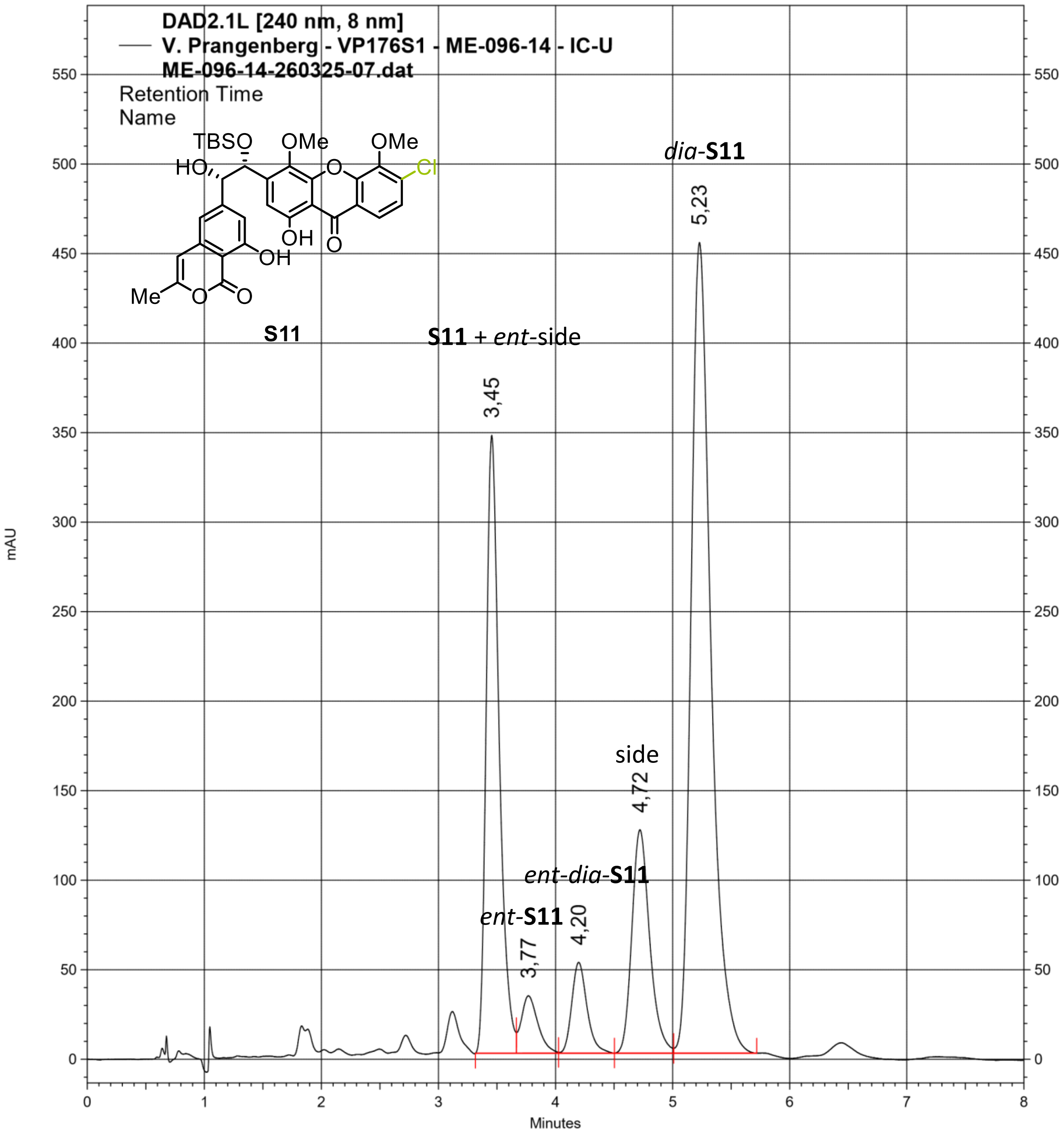
DAD2.1L [240 nm, 8 nm]
V. Prangenberg - VP176S1 - ME-096-14 - IC-U
ME-096-14-260325-07.dat
Retention Time
Name
TBSO
OMe
OMe
HO
O
Cl
OH
O
OH
Me
O
O
S11
S11 + ent-side
3,45
dia-S11
5,23
side
4,72
ent-dia-S11
ent-S11
4,20
3,77
mAU
Minutes

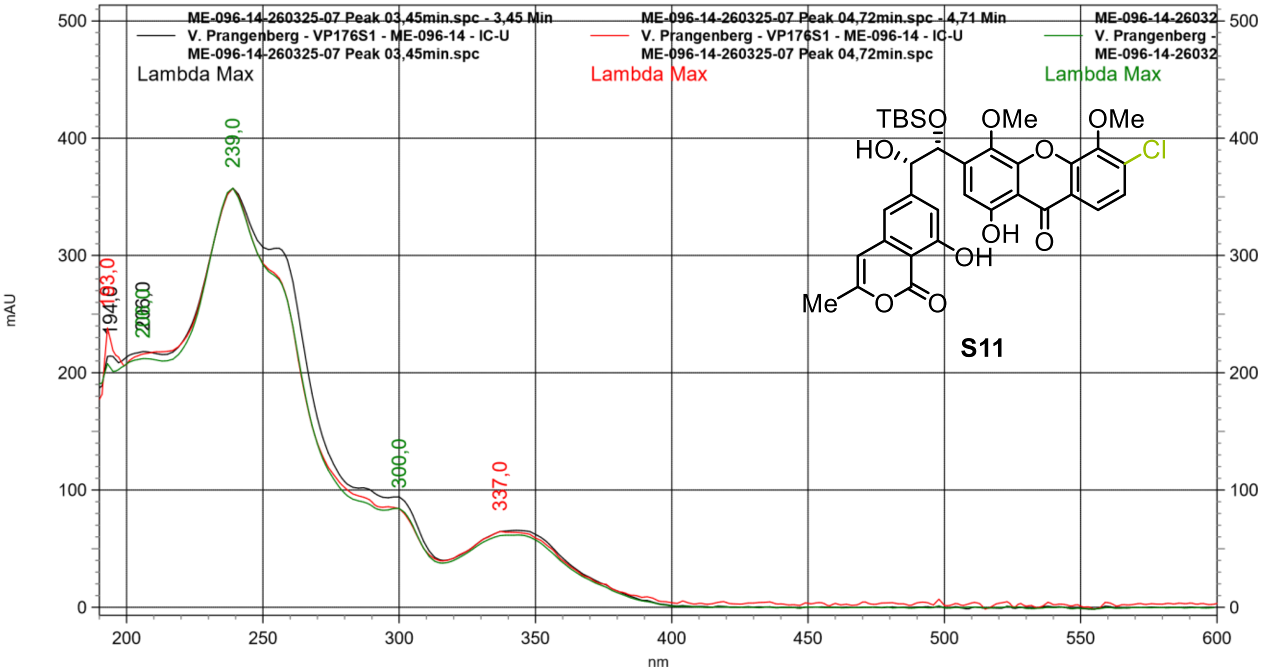

nm, 8 nm] Results

Retention Time	Area	Area %	Height	Height %
3,455	268158458	26,50	34515726	34,32
3,768	31817882	3,14	3208391	3,19
4,198	45962962	4,54	5076996	5,05
4,721	131915659	13,04	12480436	12,41
5,229	534140127	52,78	45278321	45,03
Totals	1011995088	100,00	100559870	100,00

DAD2.1L [300 nm, 8 nm] Results

Retention Time	Area	Area %	Height	Height %
3,455	71216648	28,08	9098158	36,55
3,769	9269005	3,65	895369	3,60
4,197	11905753	4,69	1237594	4,97
4,723	31855016	12,56	2915603	11,71
5,230	129379237	51,01	10743607	43,16
Totals	253625659	100,00	24890331	100,00

7 Experimental Spectra

7.1 Leading to Isocoumarin **13**

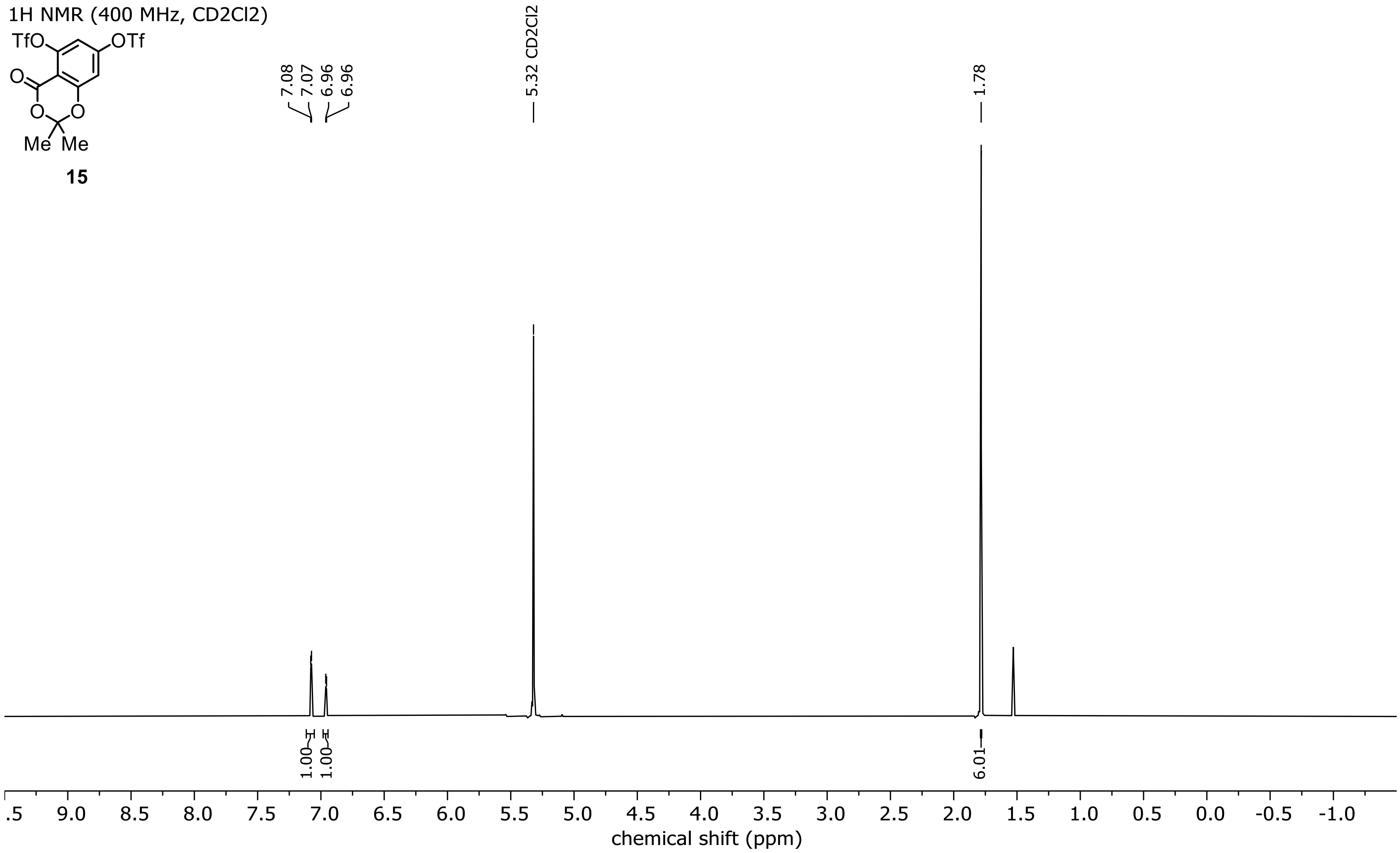

13C NMR (125 MHz, CD2Cl2)

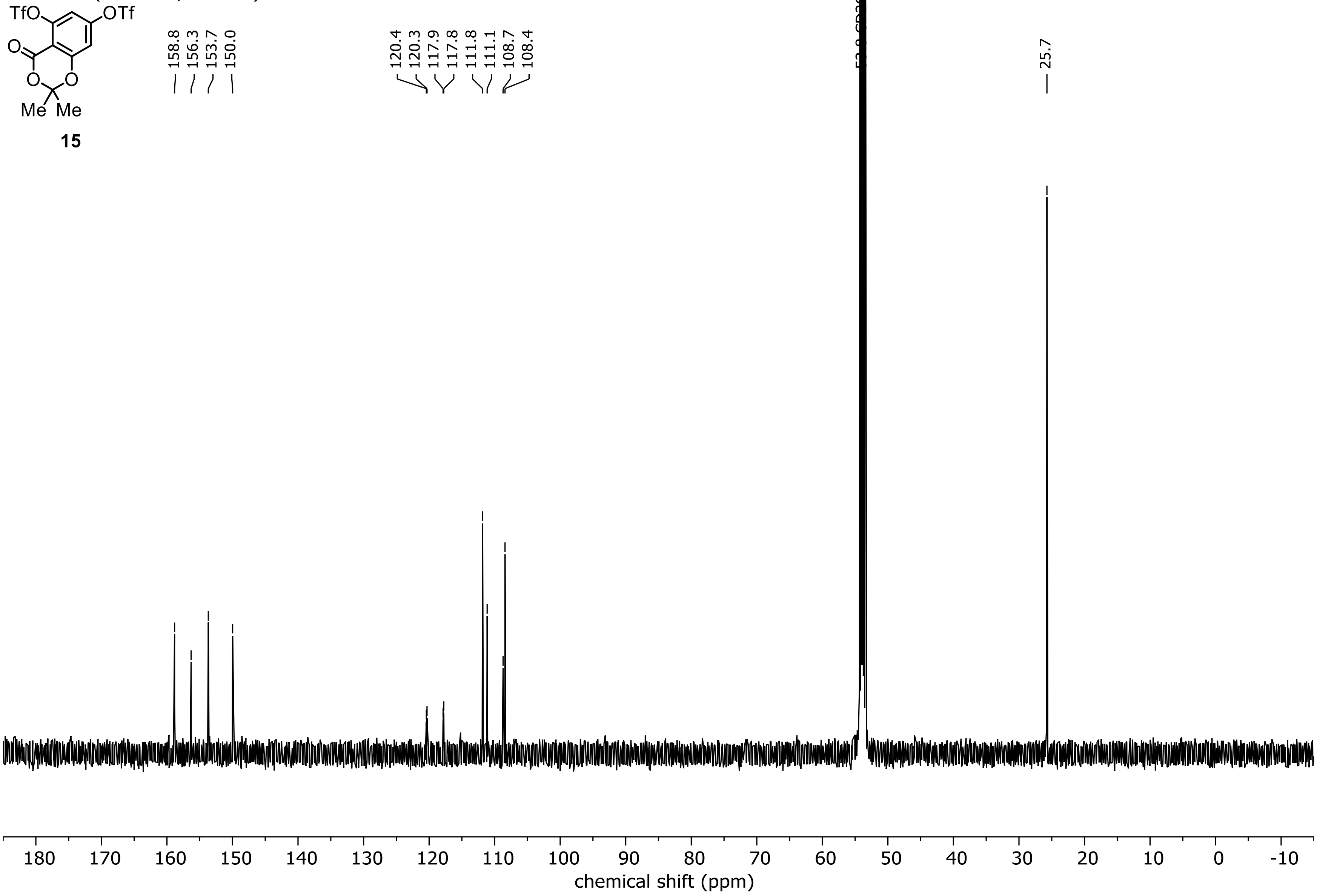

15

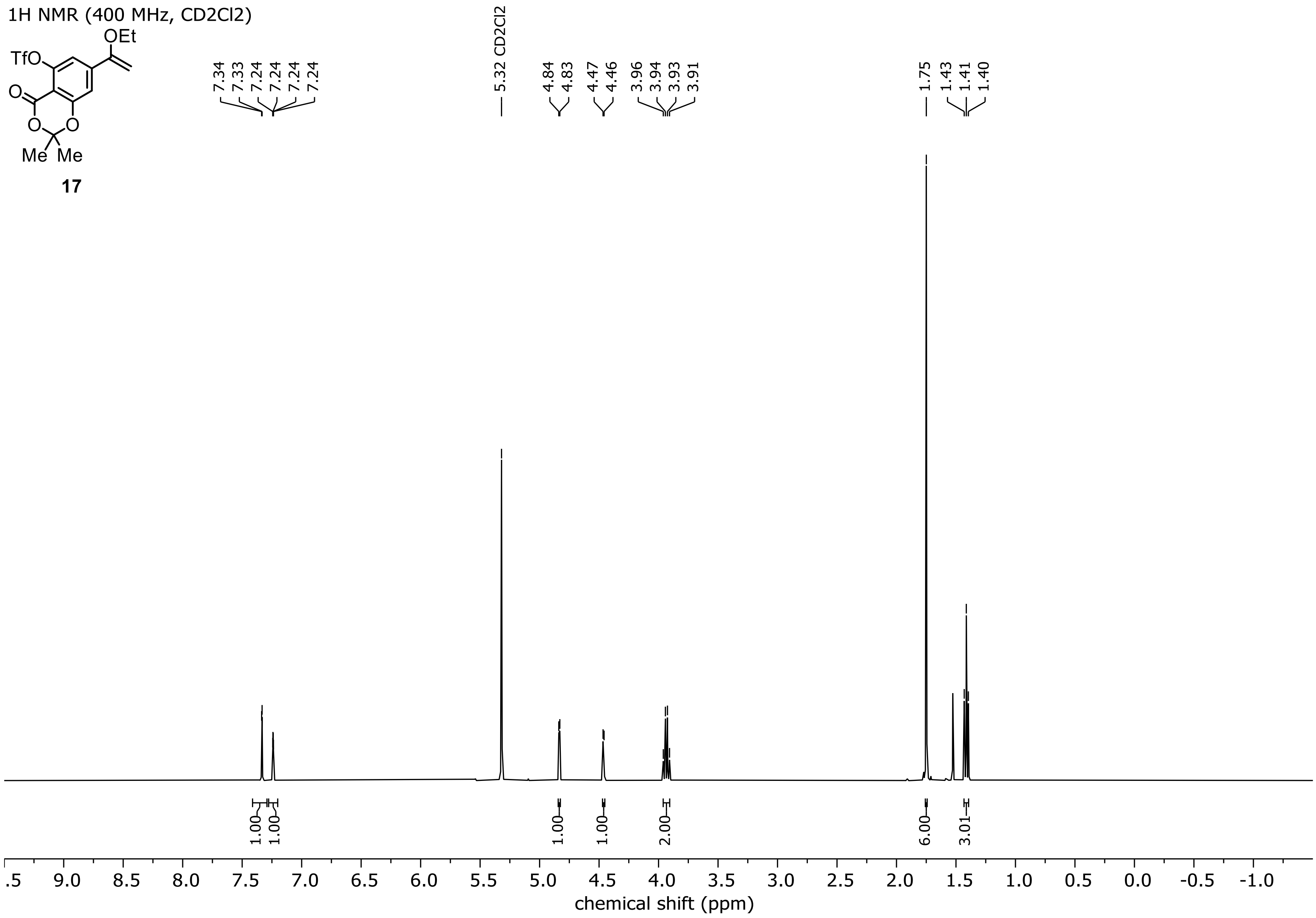
1H NMR (400 MHz, CD2Cl2)
OEt
TfO
O
O
O
Me Me
17
7.34
7.33
7.24
7.24
7.24
7.24
5.32 CD2Cl2
4.84
4.83
4.47
4.46
3.96
3.94
3.93
3.91
1.75
1.43
1.41
1.40
1.00
1.00
1.00
1.00
2.00
6.00
3.01
.5
9.0
8.5
8.0
7.5
7.0
6.5
6.0
5.5
5.0
4.5
4.0
3.5
3.0
2.5
2.0
1.5
1.0
0.5
0.0
-0.5
-1.0
chemical shift (ppm)

13C NMR (100 MHz, CD2Cl2)

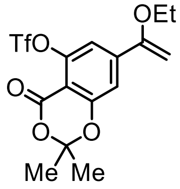

17

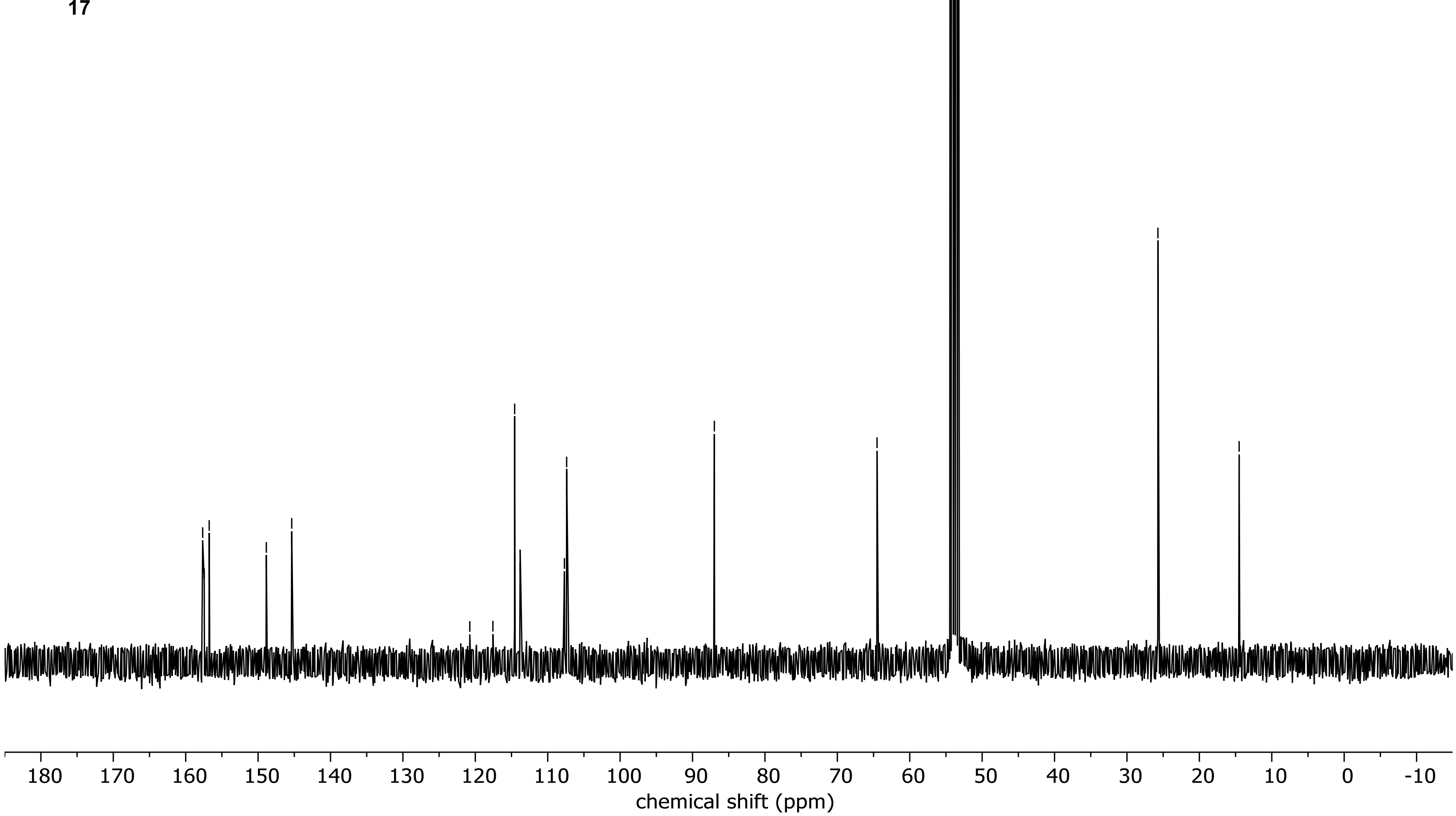

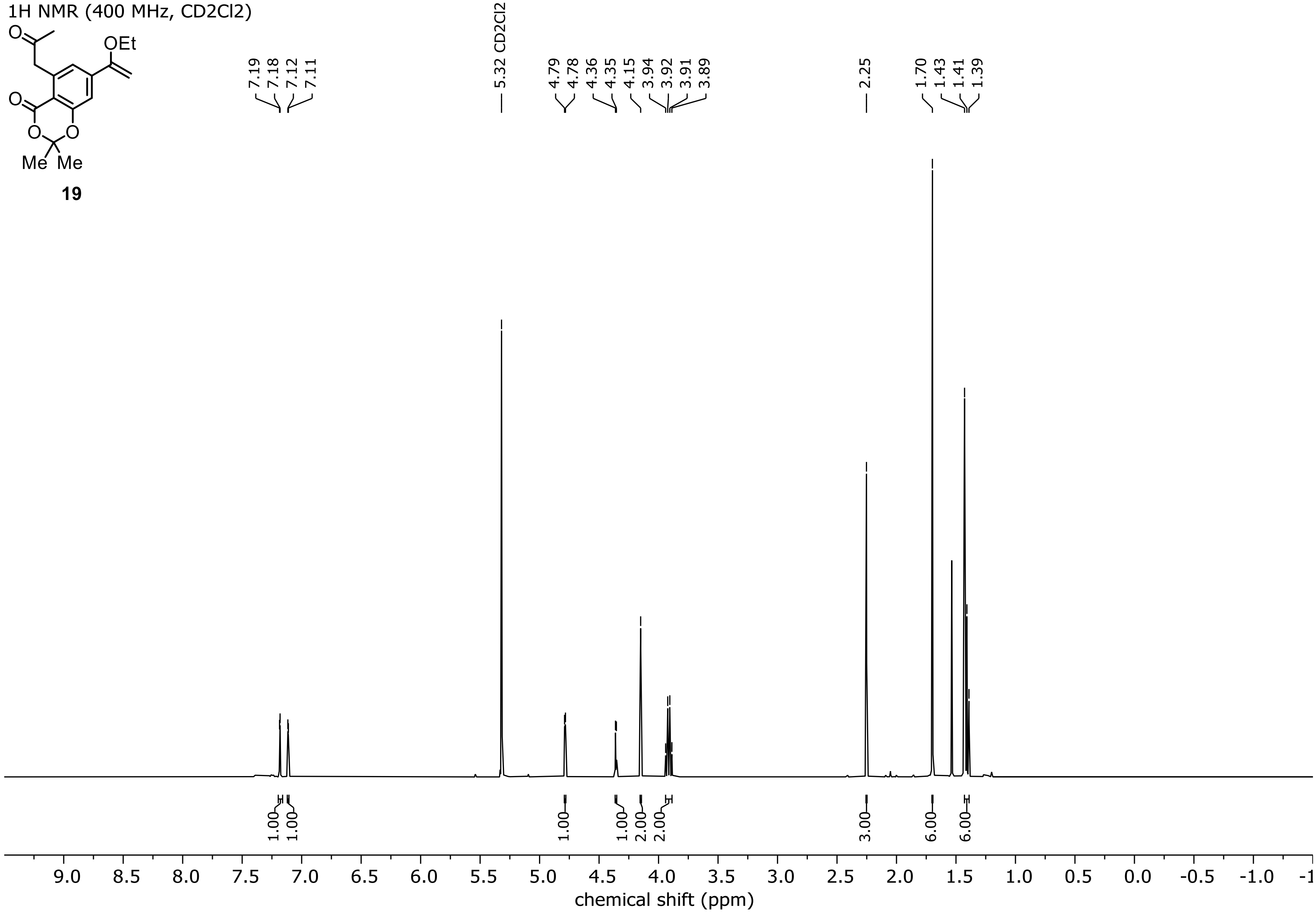
1H NMR (400 MHz, CD2Cl2)
OEt
Me Me
19
7.19
7.18
7.12
7.11
5.32 CD2Cl2
4.79
4.78
4.36
4.35
4.15
3.94
3.92
3.91
3.89
2.25
1.70
1.43
1.41
1.39
1.00
1.00
1.00
1.00
2.00
2.00
3.00
6.00
6.00
9.0 8.5 8.0 7.5 7.0 6.5 6.0 5.5 5.0 4.5 4.0 3.5 3.0 2.5 2.0 1.5 1.0 0.5 0.0 -0.5 -1.0
chemical shift (ppm)

13C NMR (176 MHz, CD2Cl2)

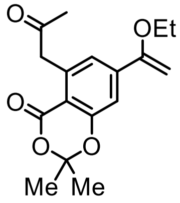

19

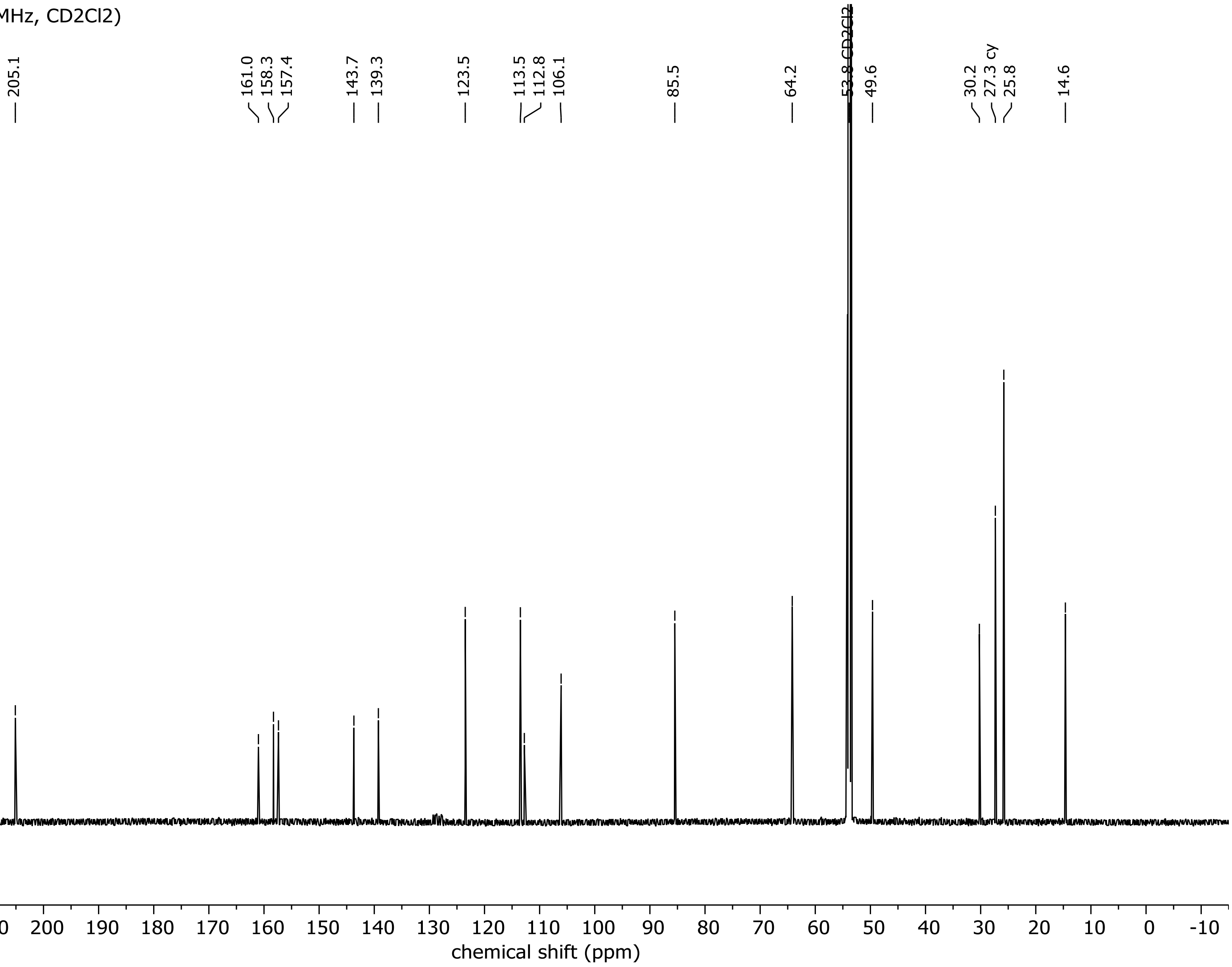

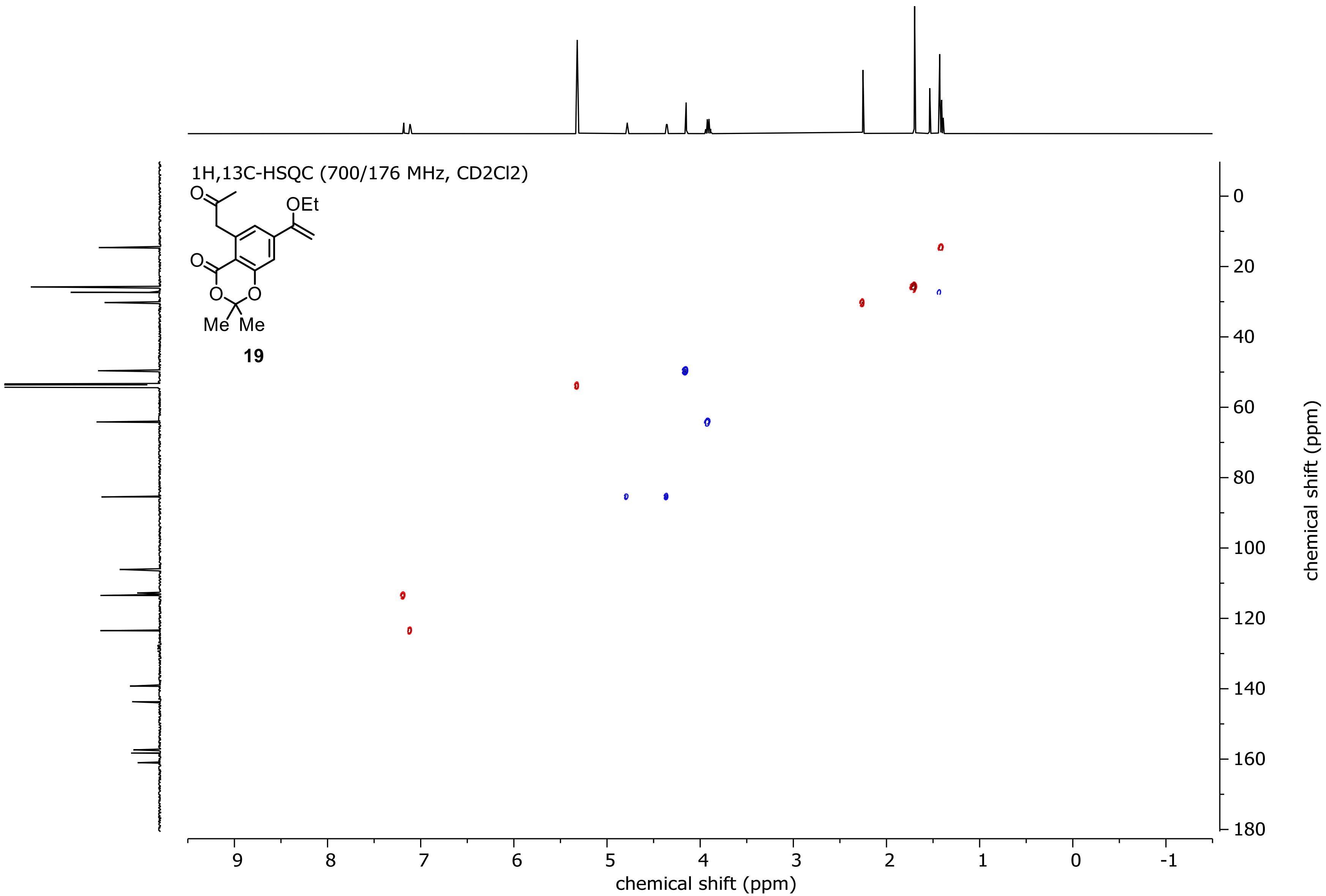
1H,13C-HSQC (700/176 MHz, CD2Cl2)
OEt
Me Me
19
chemical shift (ppm)
chemical shift (ppm)

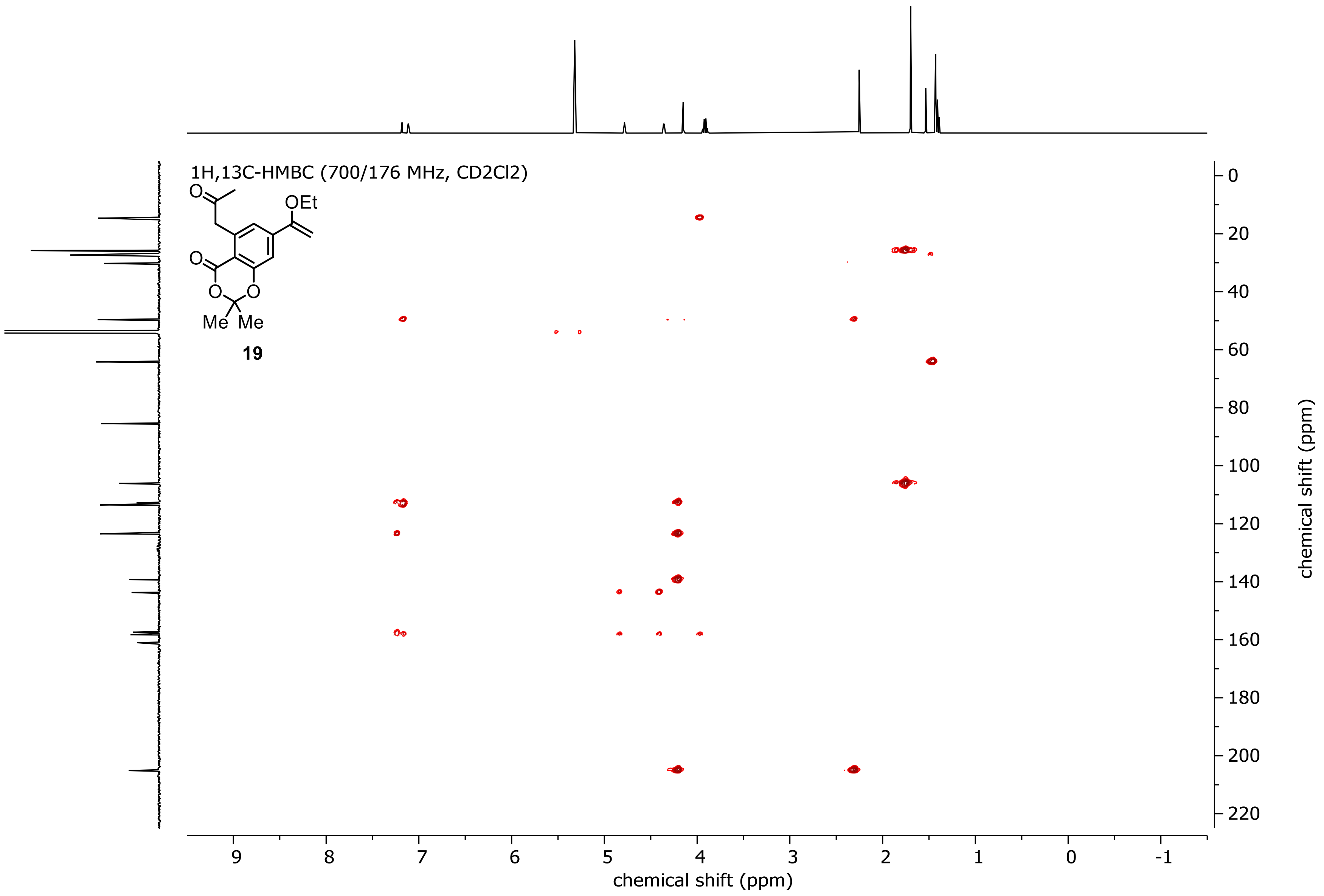

1H,13C-HMBC (700/176 MHz, CD2Cl2)
OEt
O
O
O
O
Me Me
19
chemical shift (ppm)
chemical shift (ppm)

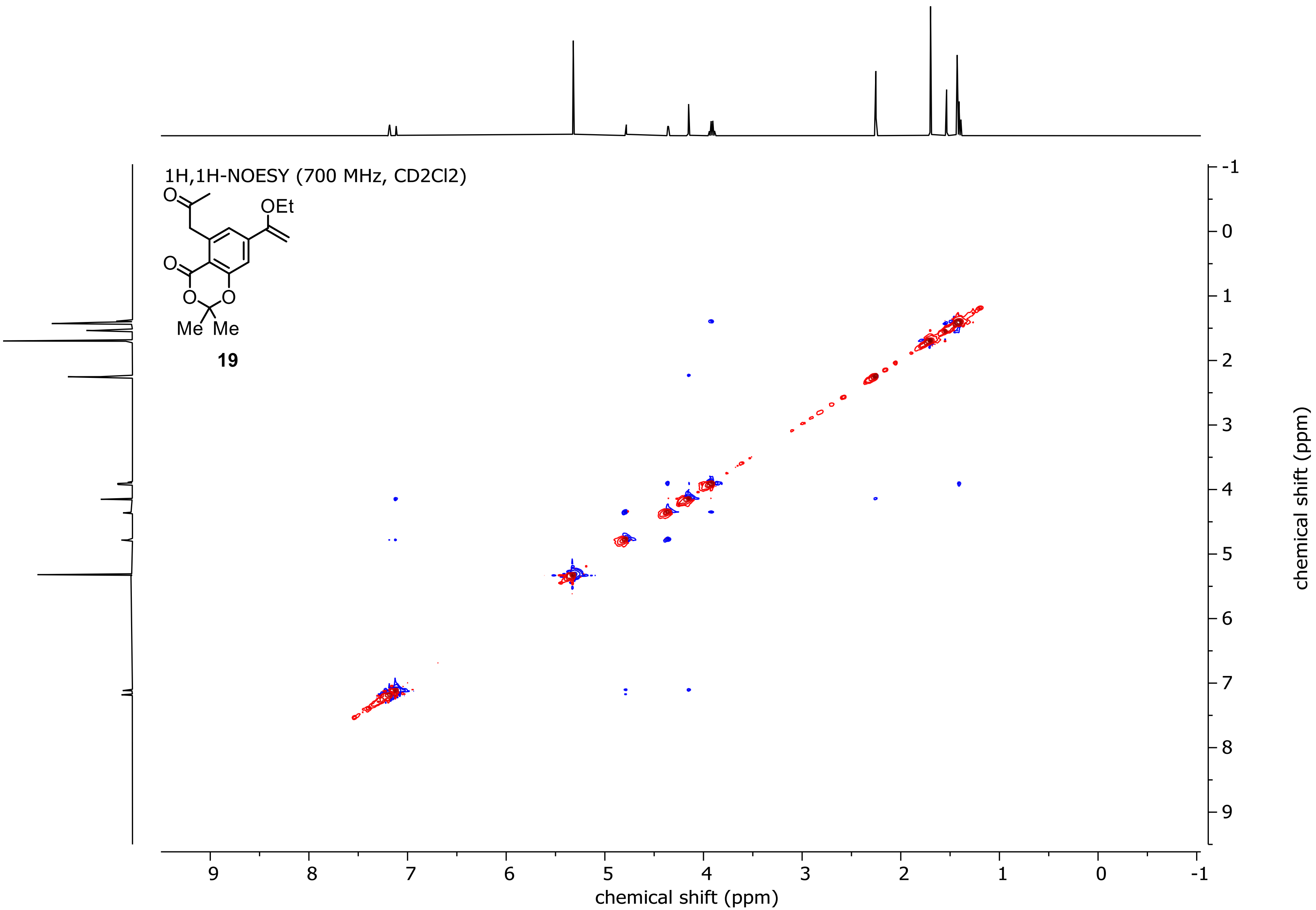
1H,1H-NOESY (700 MHz, CD2Cl2)
O
OEt
O
O
O
Me Me
19
chemical shift (ppm)
chemical shift (ppm)
9
8
7
6
5
4
3
2
1
0
-1

1H NMR (400 MHz, CD2Cl2)

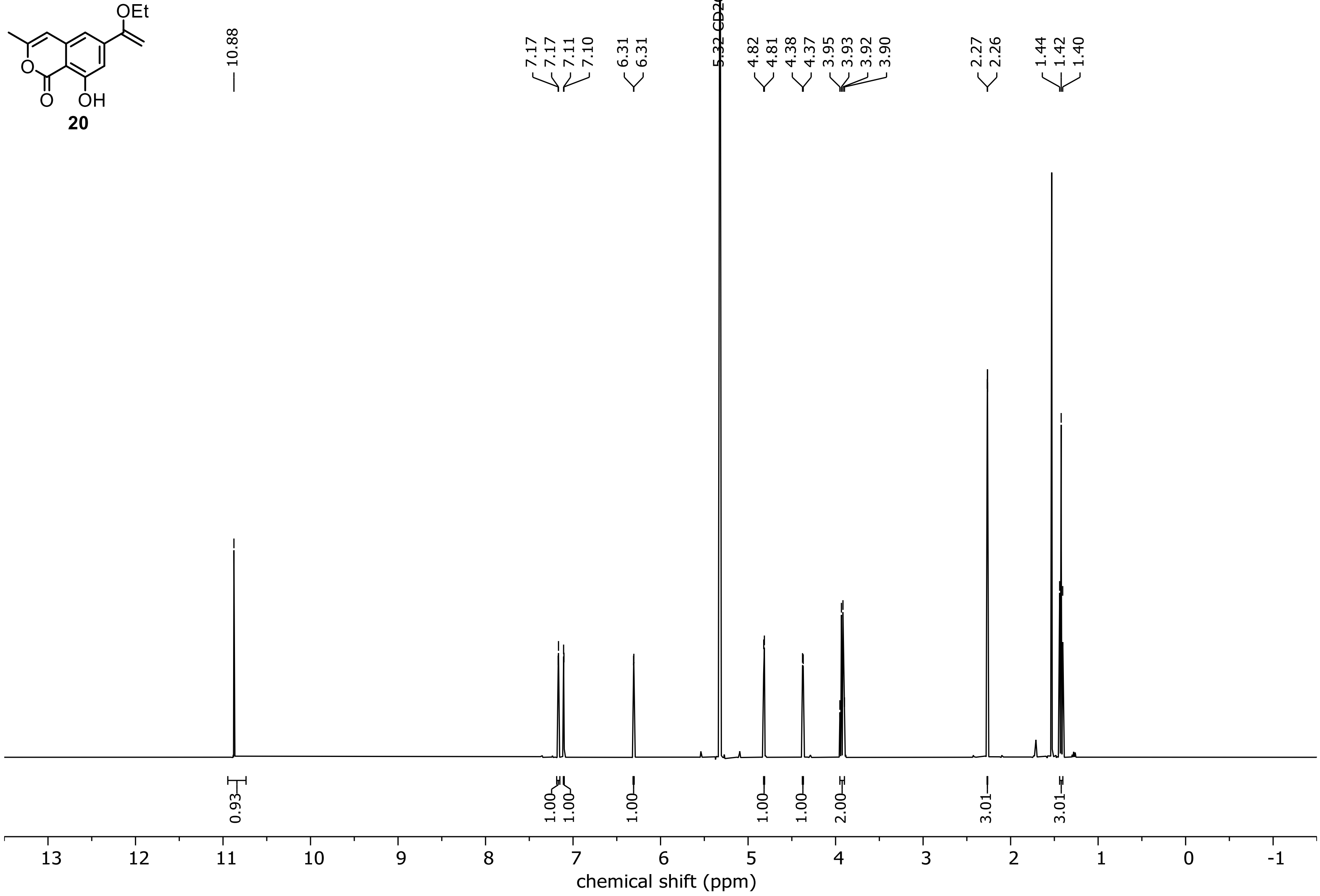

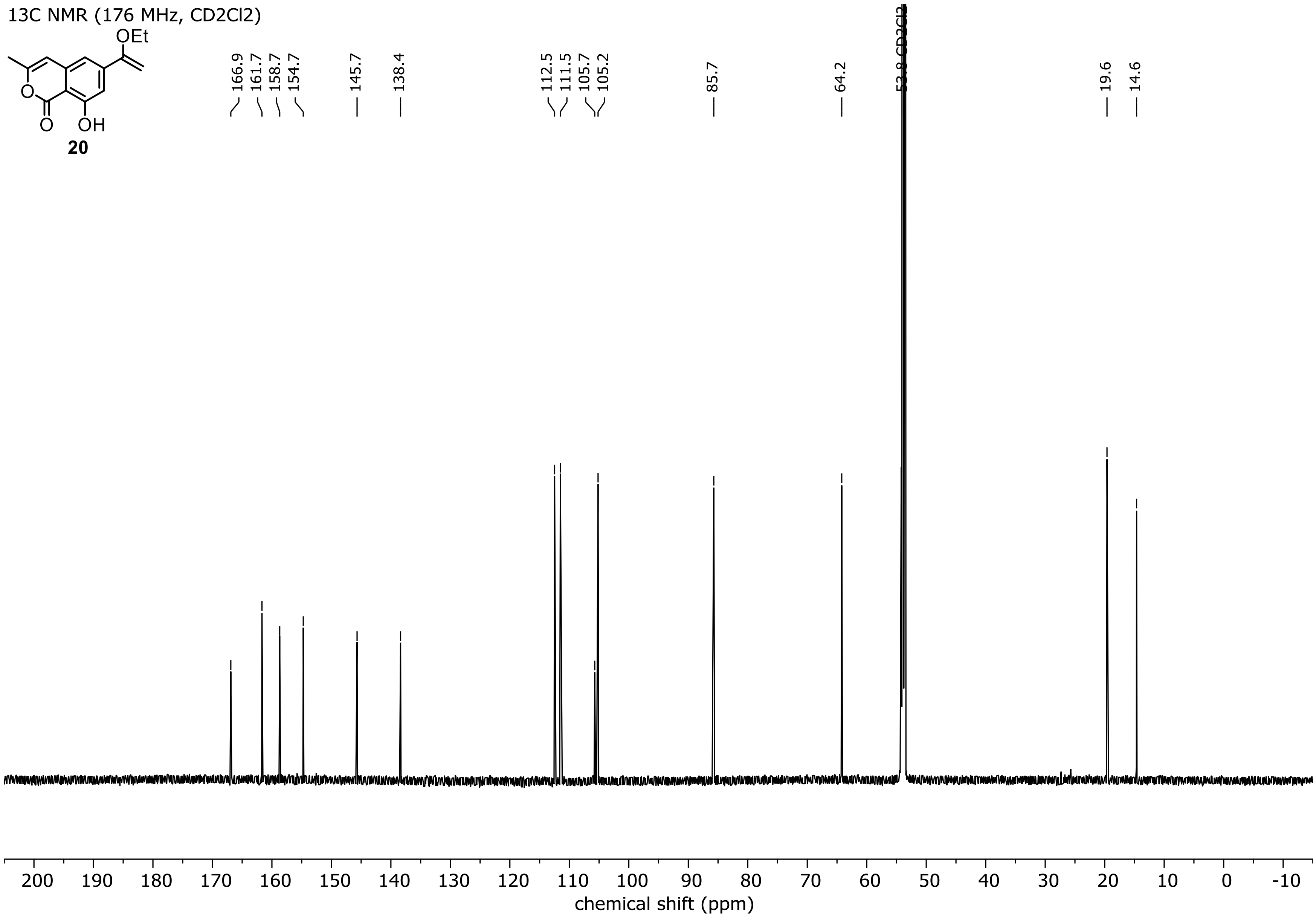
13C NMR (176 MHz, CD2Cl2)
OEt
O
O
OH
20
166.9
161.7
158.7
154.7
145.7
138.4
112.5
111.5
105.7
105.2
85.7
64.2
53.8 CD2Cl2
19.6
14.6
200 190 180 170 160 150 140 130 120 110 100 90 80 70 60 50 40 30 20 10 0 -10
chemical shift (ppm)

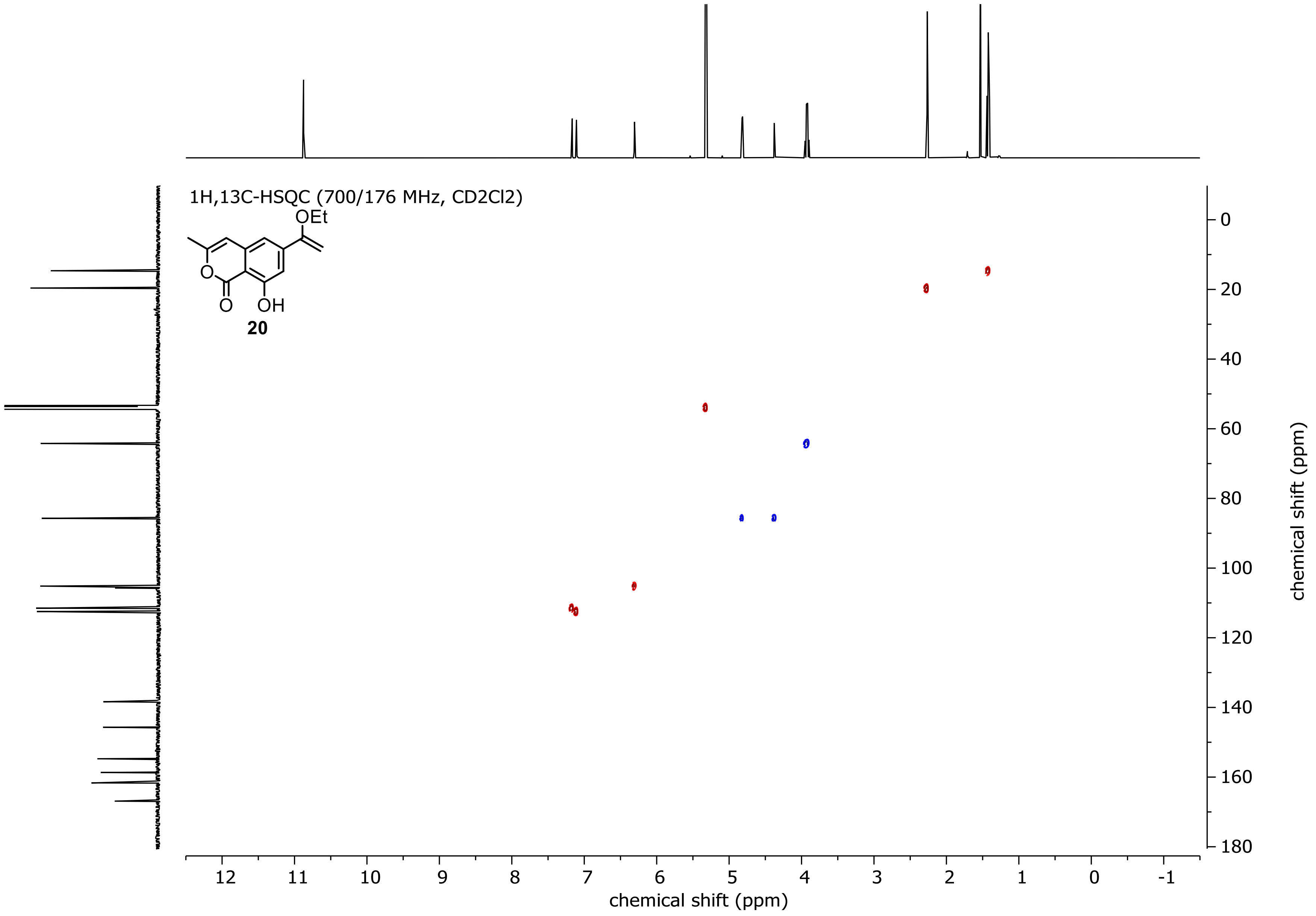

1H,13C-HSQC (700/176 MHz, CD2Cl2)
OEt
O
O
OH
20
0
20
40
60
80
100
120
140
160
180
chemical shift (ppm)
12
11
10
9
8
7
6
5
4
3
2
1
0
-1
chemical shift (ppm)

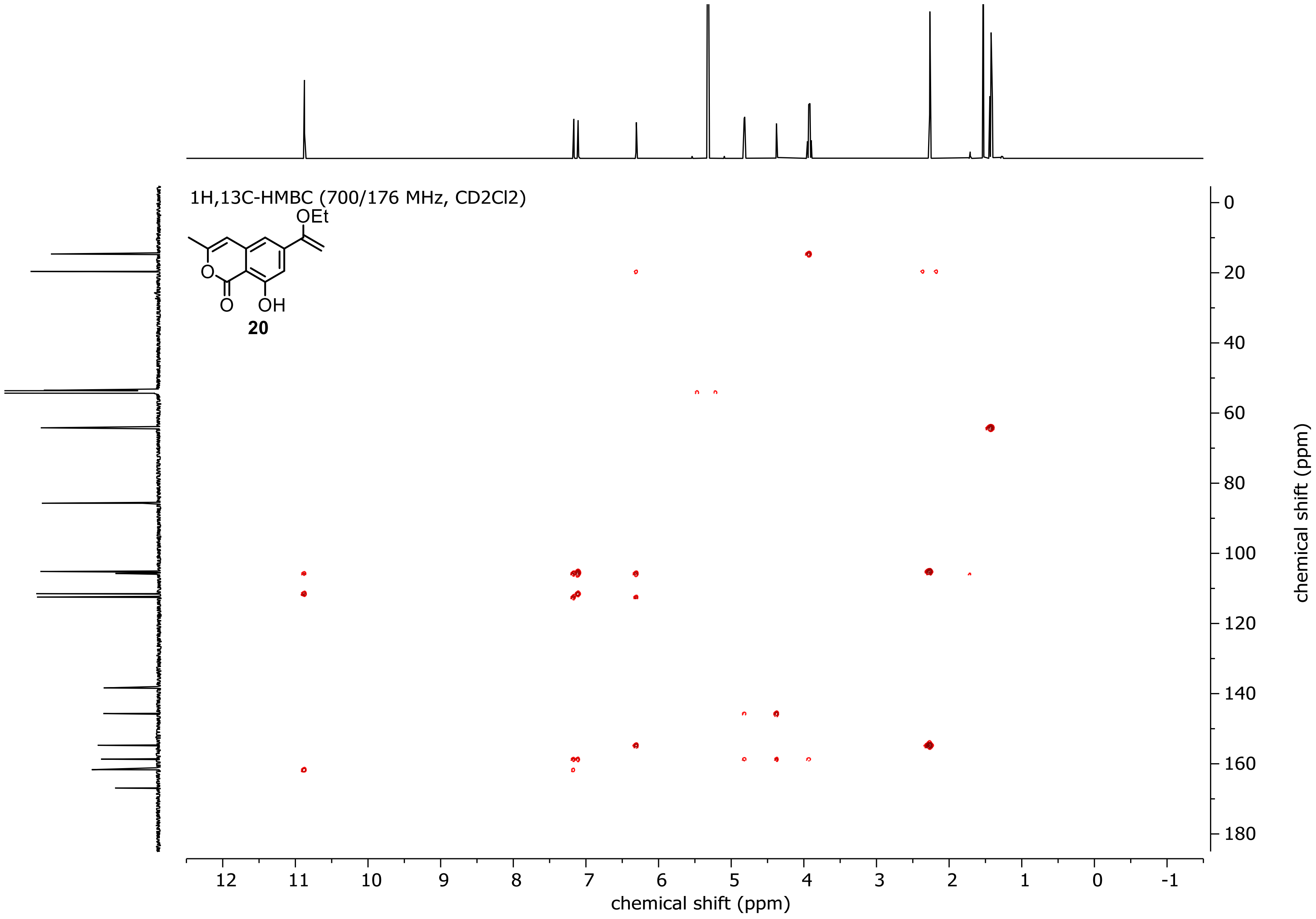
1H,13C-HMBC (700/176 MHz, CD2Cl2)
OEt
O
O
OH
20
0
20
40
60
80
100
120
140
160
180
chemical shift (ppm)
12
11
10
9
8
7
6
5
4
3
2
1
0
-1
chemical shift (ppm)

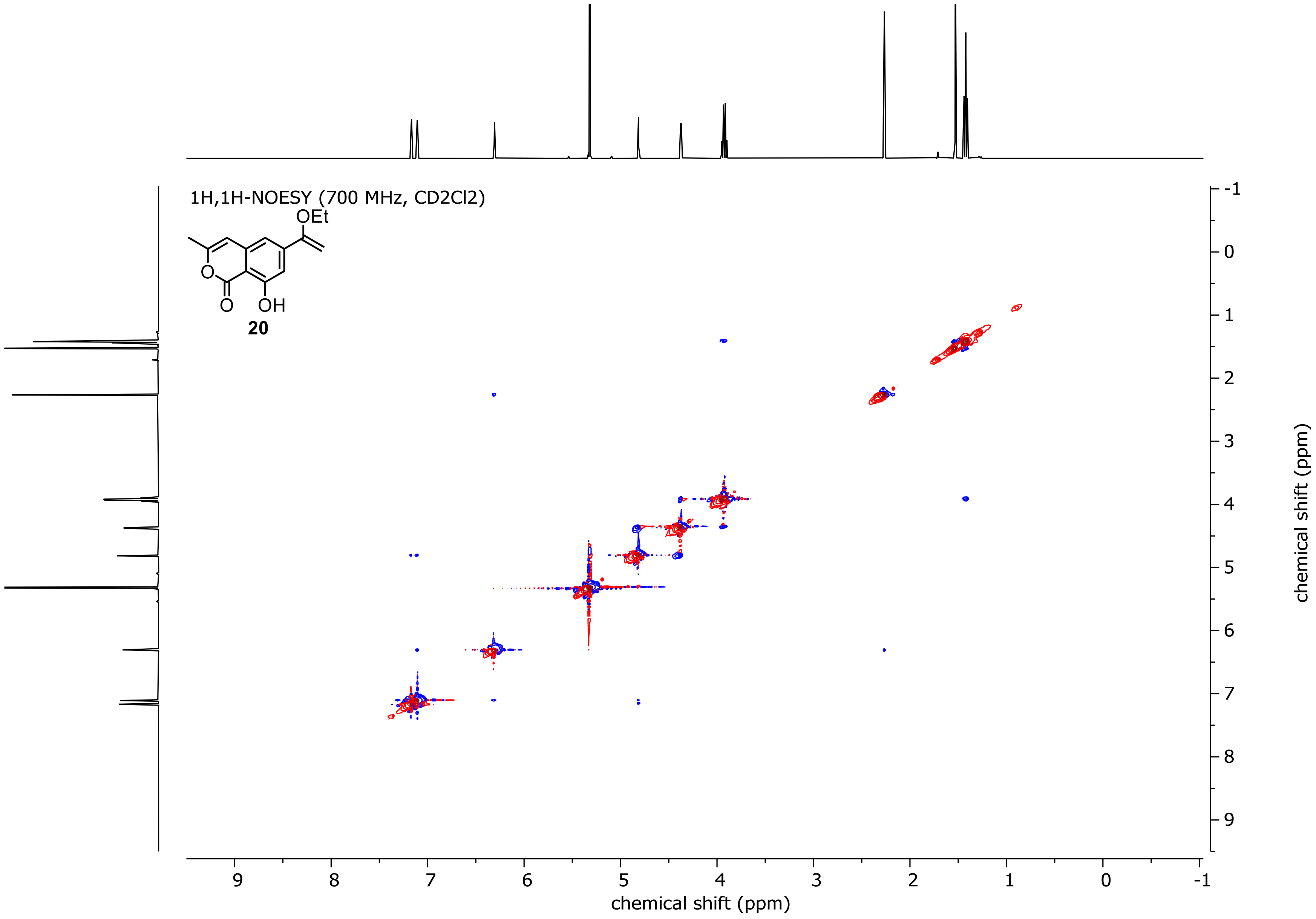
1H,1H-NOESY (700 MHz, CD2Cl2)
OEt
O
O
OH
20
chemical shift (ppm)
chemical shift (ppm)

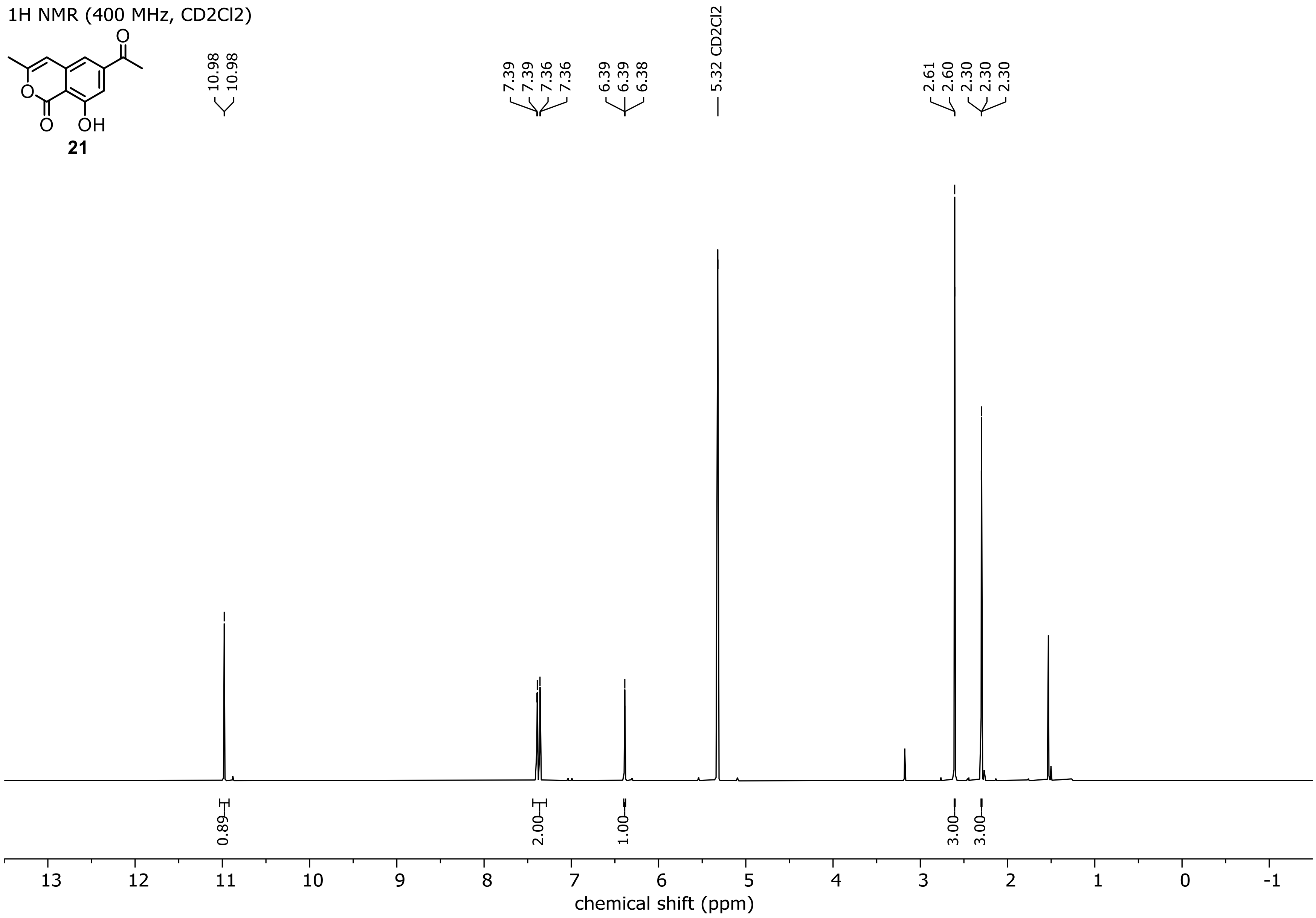
1H NMR (400 MHz, CD2Cl2)
21
10.98
10.98
7.39
7.39
7.36
7.36
6.39
6.39
6.38
5.32 CD2Cl2
2.61
2.60
2.30
2.30
2.30
0.89
2.00
1.00
3.00
3.00
13
12
11
10
9
8
7
6
5
4
3
2
1
0
-1
chemical shift (ppm)

13C NMR (125 MHz, CD2Cl2)

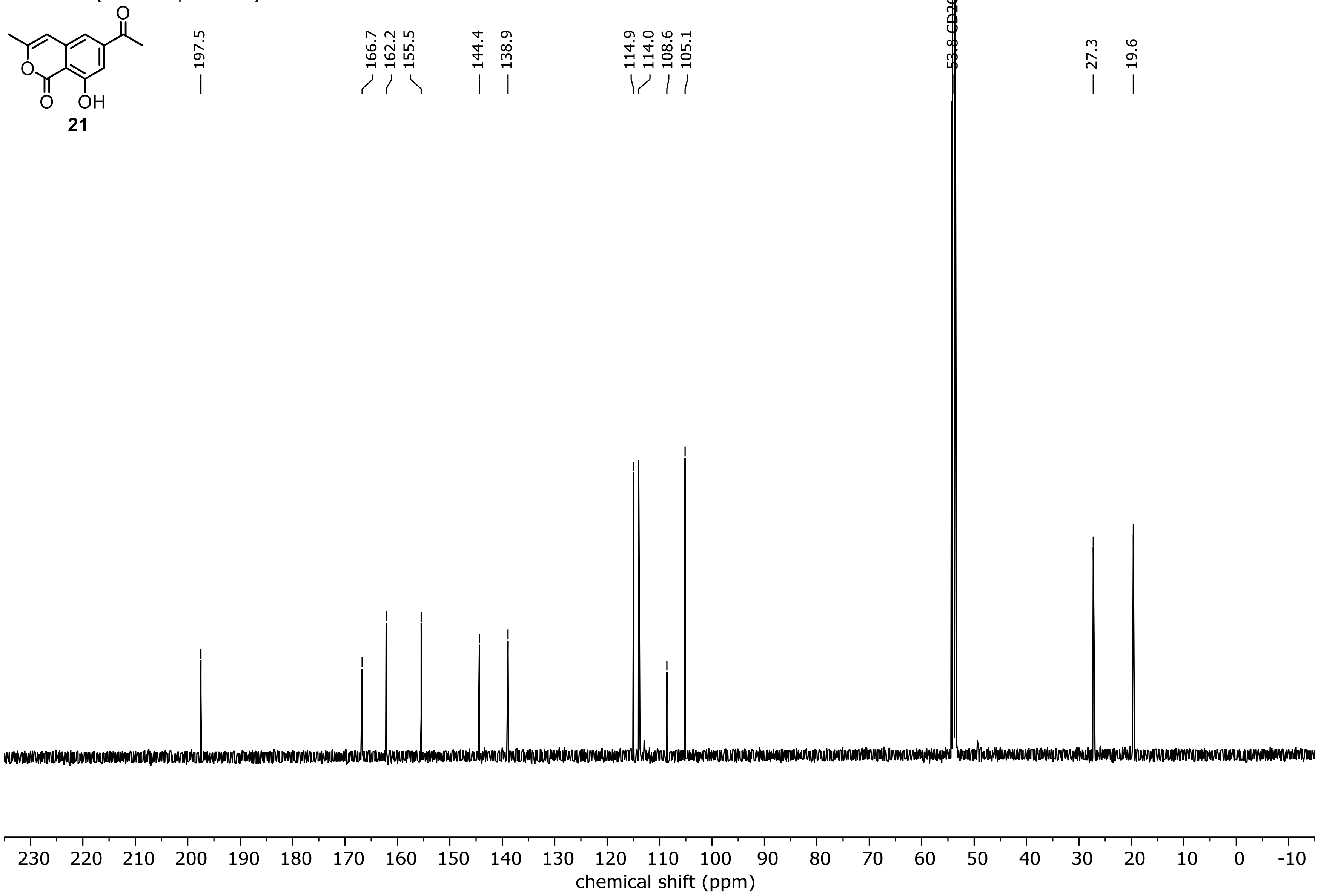

1H NMR (500 MHz, CD2Cl2)

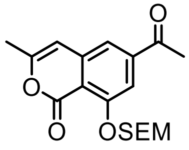

13a

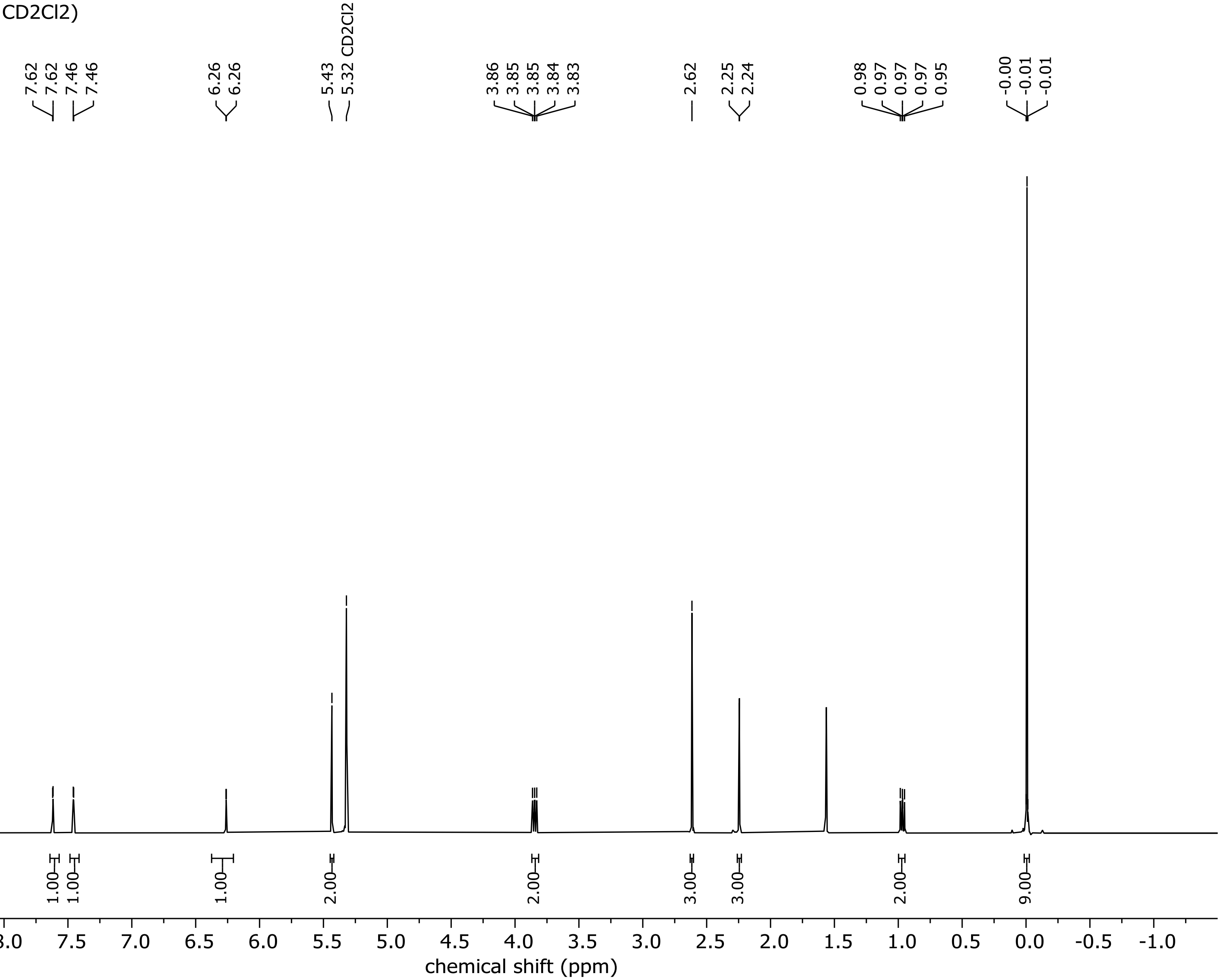

13C NMR (125 MHz, CD2Cl2)

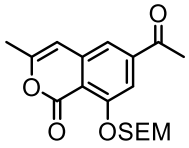

13a

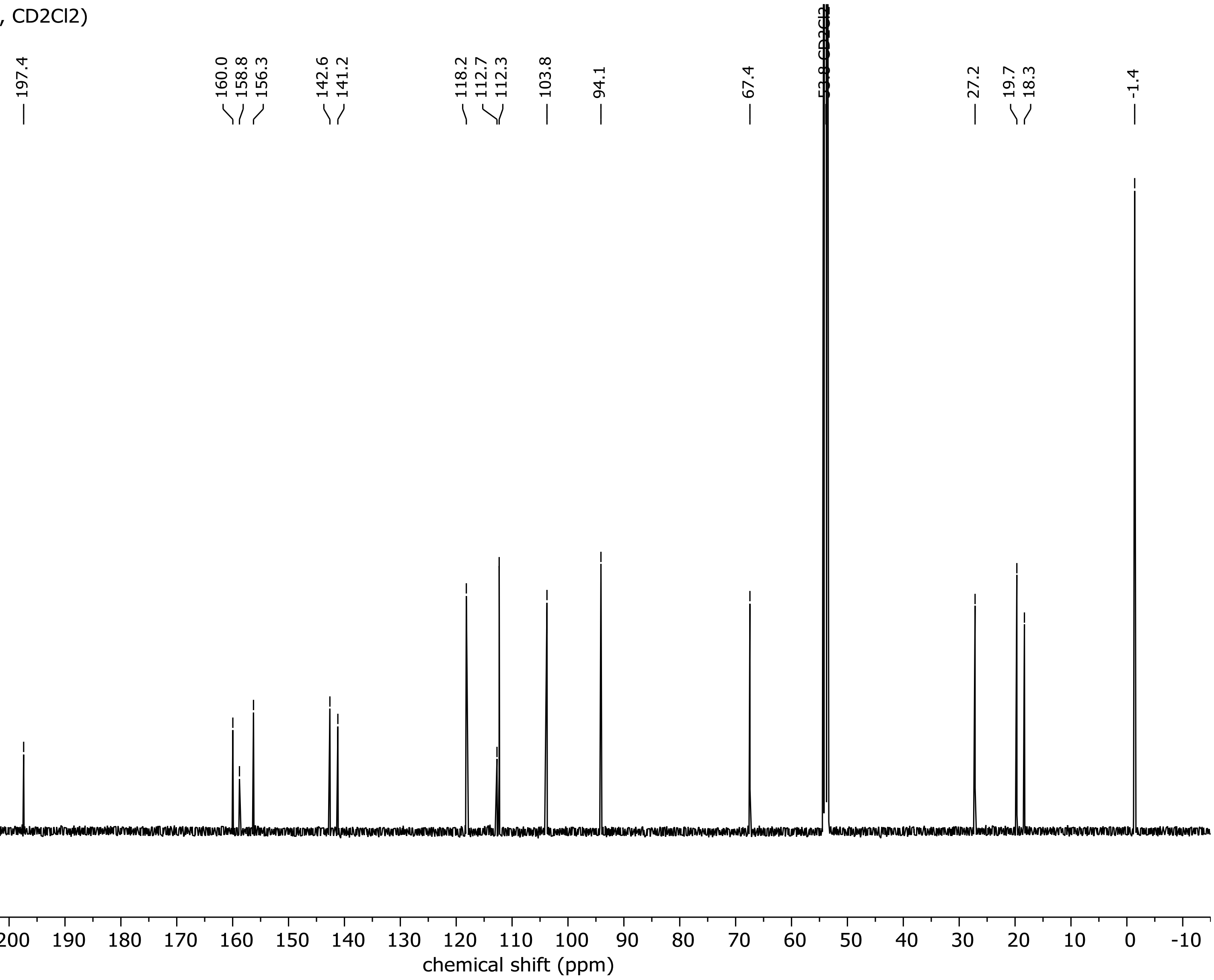

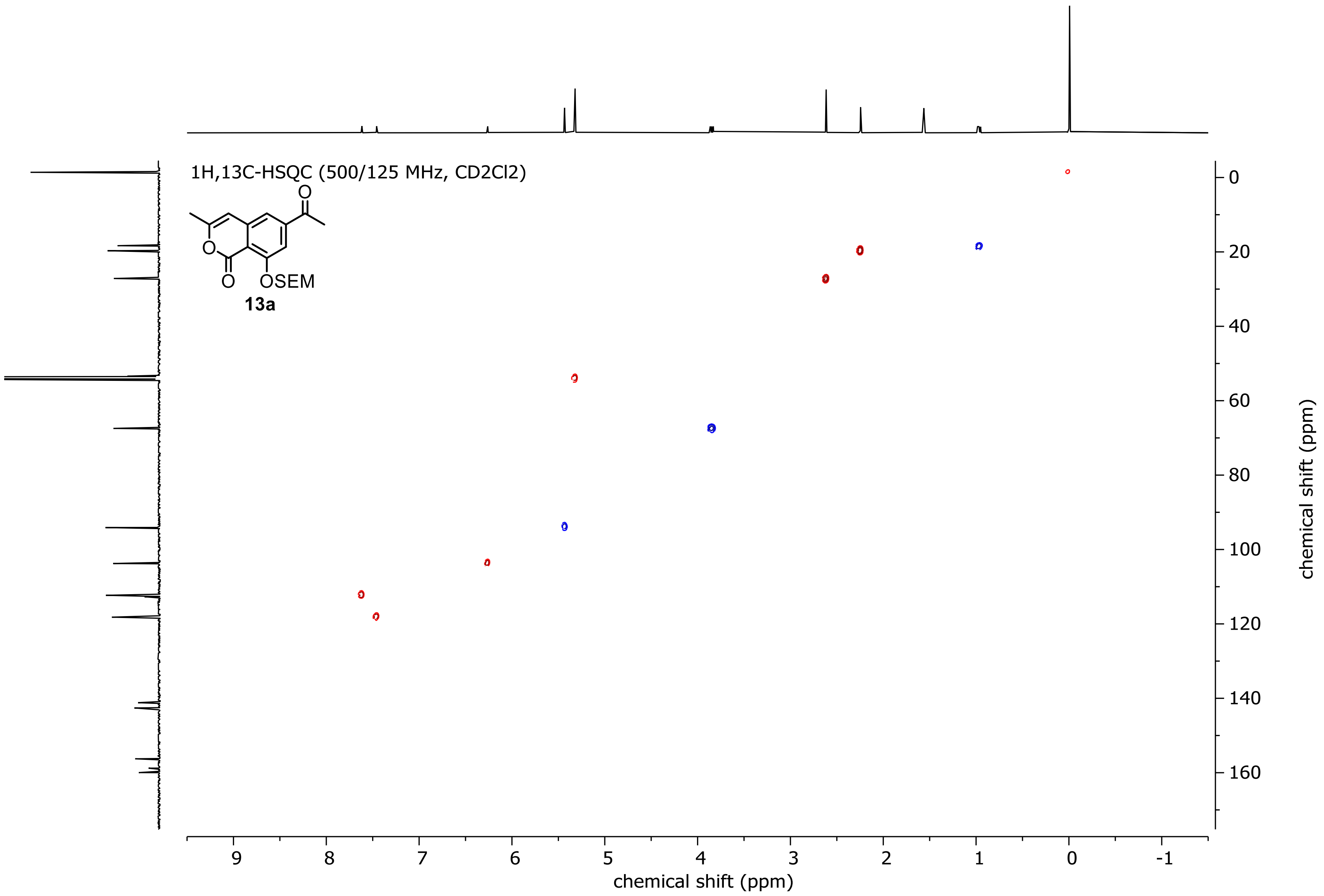

1H,13C-HSQC (500/125 MHz, CD2Cl2)
O
O
O
OSEM
13a
0
20
40
60
80
100
120
140
160
chemical shift (ppm)
9
8
7
6
5
4
3
2
1
0
-1
chemical shift (ppm)

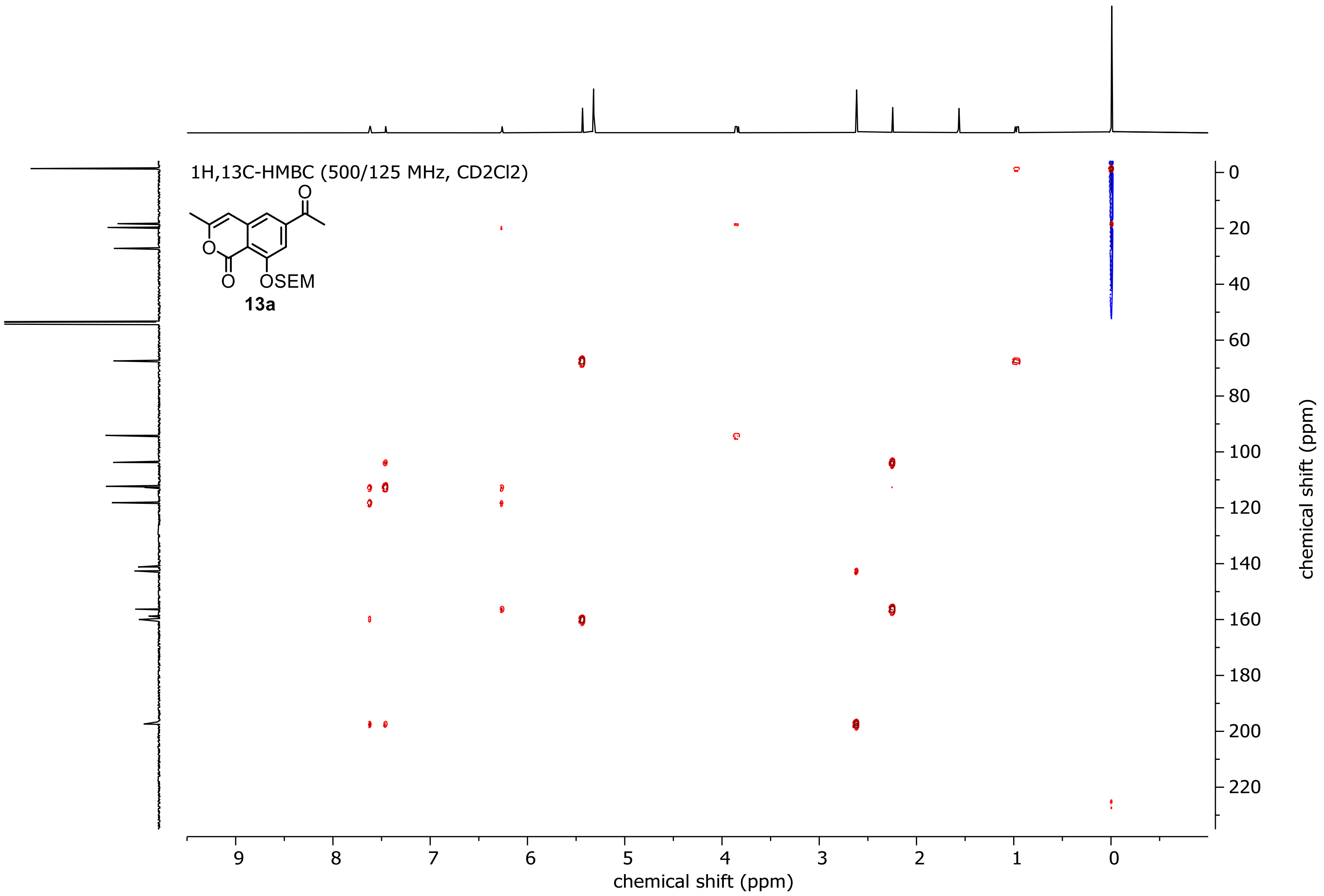
1H,13C-HMBC (500/125 MHz, CD2Cl2)
OSEM
13a
chemical shift (ppm)
chemical shift (ppm)

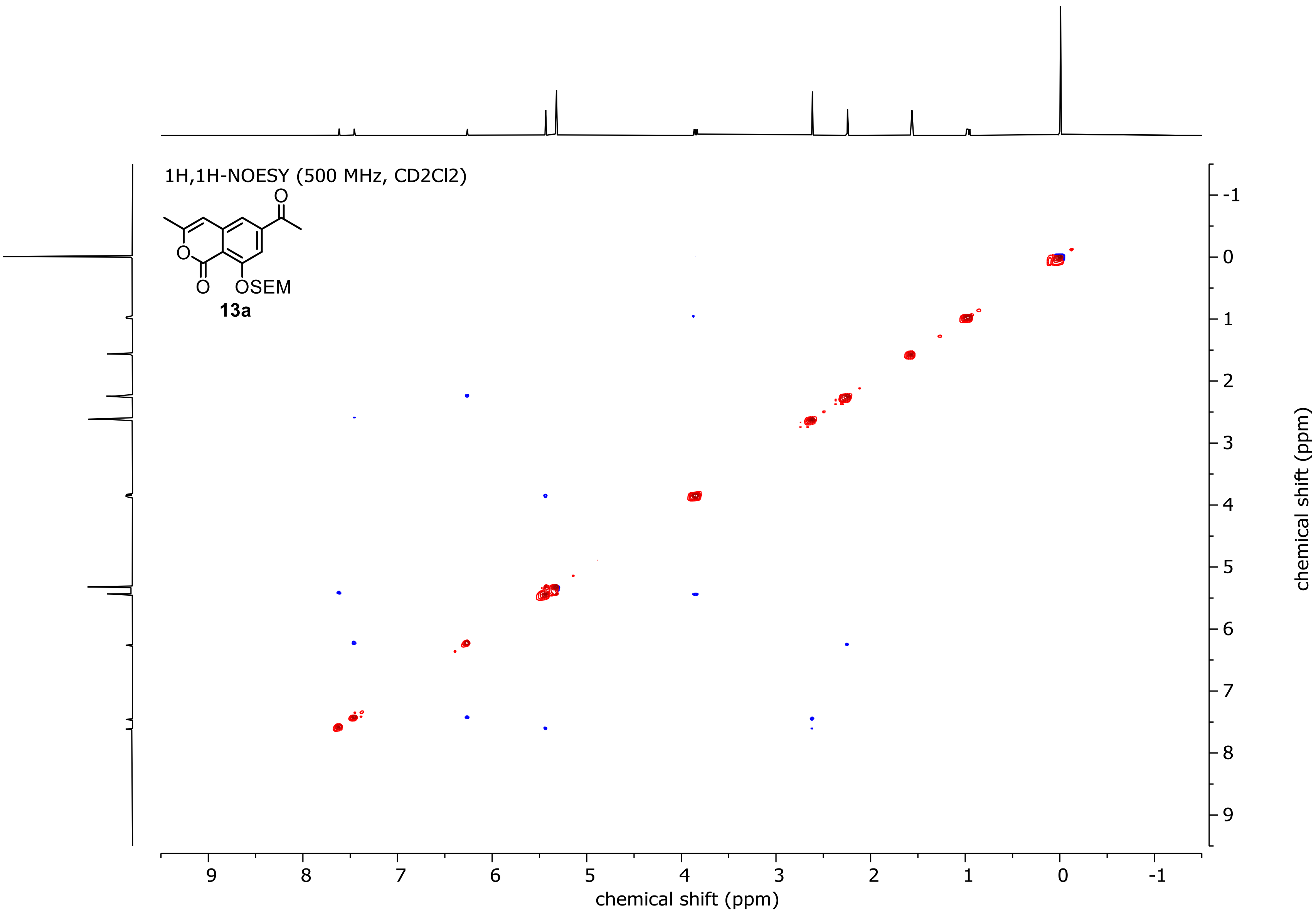
1H,1H-NOESY (500 MHz, CD2Cl2)
O
O
O
OSEM
13a
chemical shift (ppm)
chemical shift (ppm)

1H NMR (700 MHz, CD2Cl2)

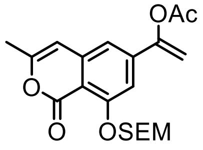

13b

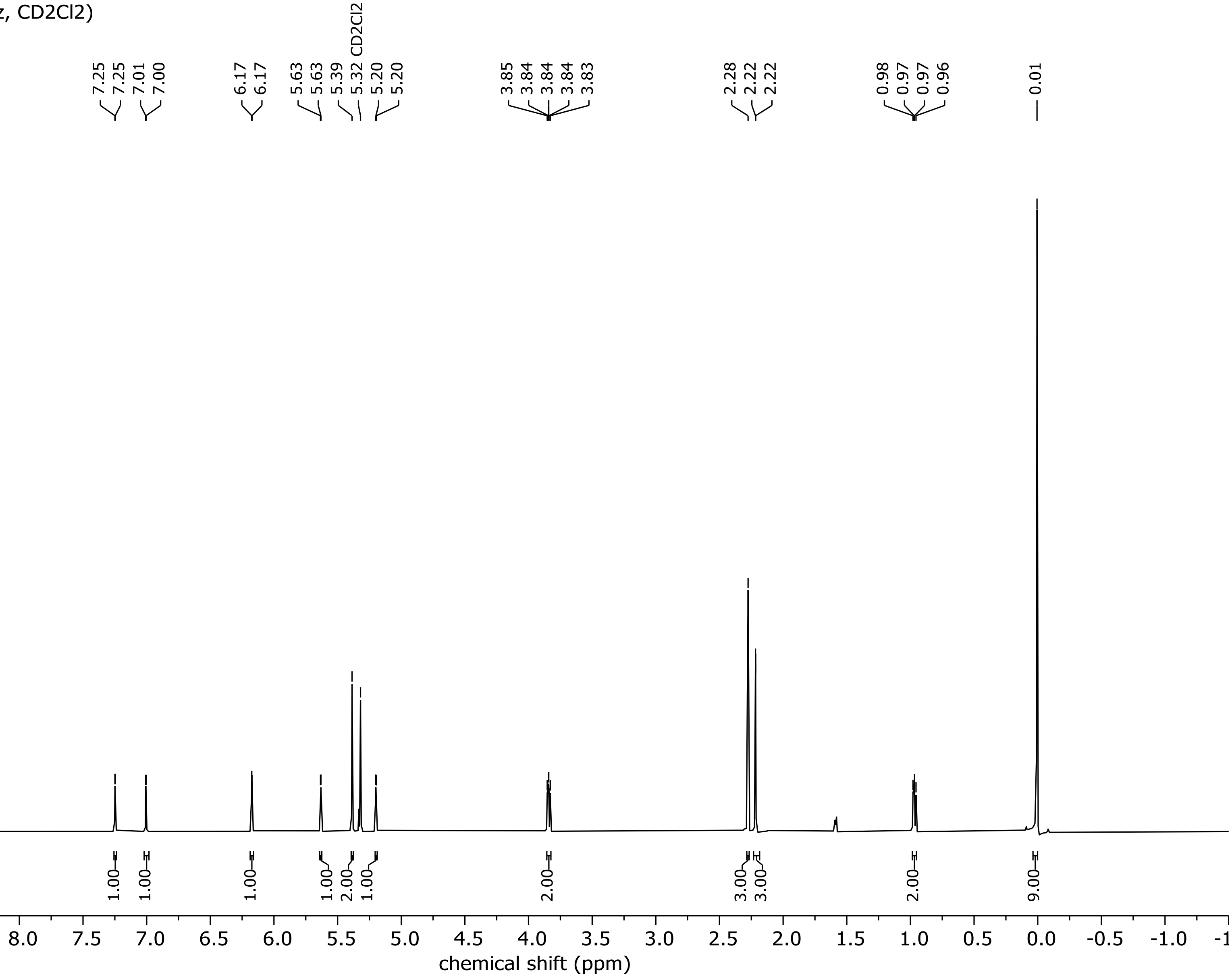

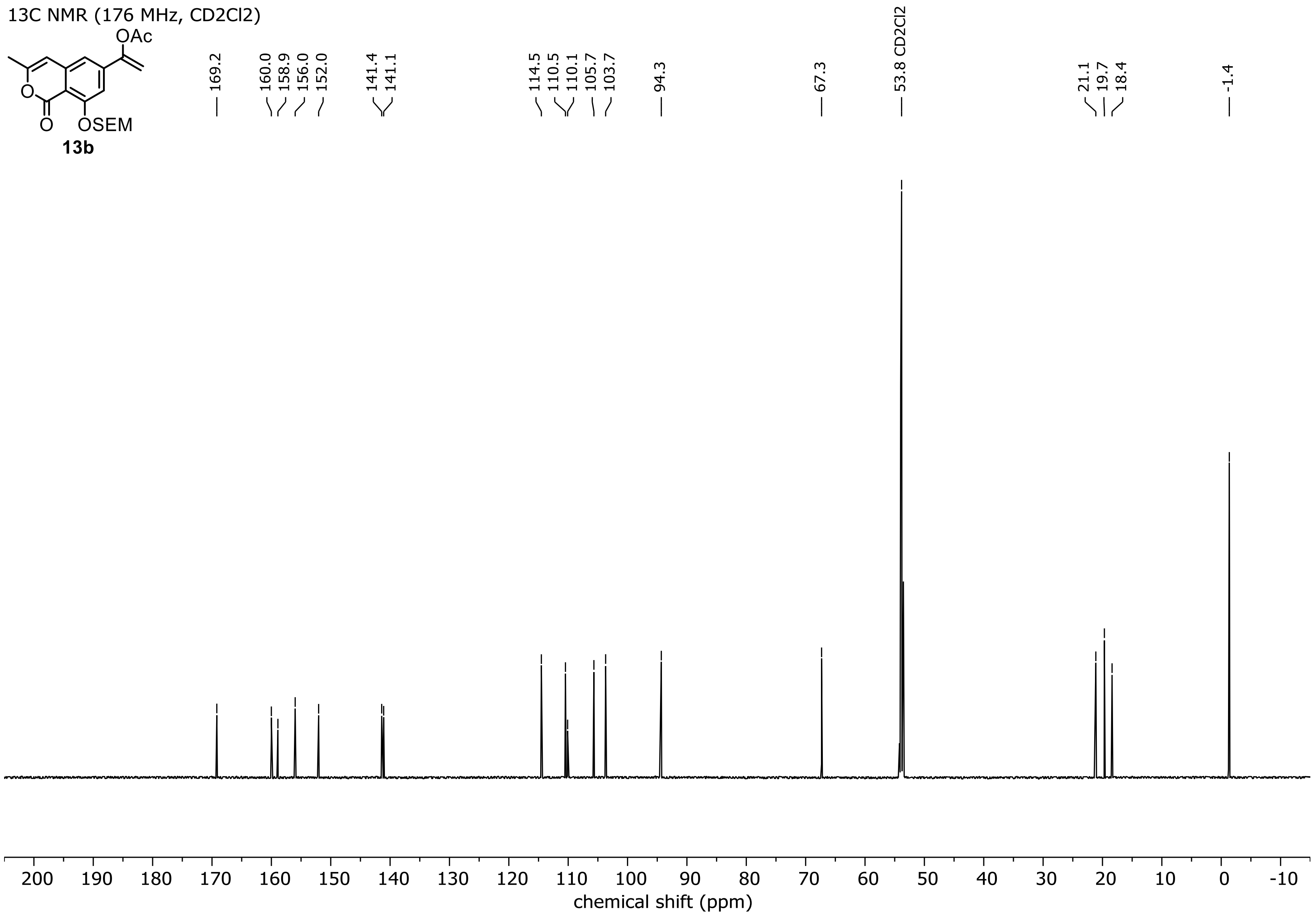
13C NMR (176 MHz, CD2Cl2)
OAc
O
O
OSEM
13b
169.2
160.0
158.9
156.0
152.0
141.4
141.1
114.5
110.5
110.1
105.7
103.7
94.3
67.3
53.8 CD2Cl2
21.1
19.7
18.4
-1.4
200 190 180 170 160 150 140 130 120 110 100 90 80 70 60 50 40 30 20 10 0 -10
chemical shift (ppm)

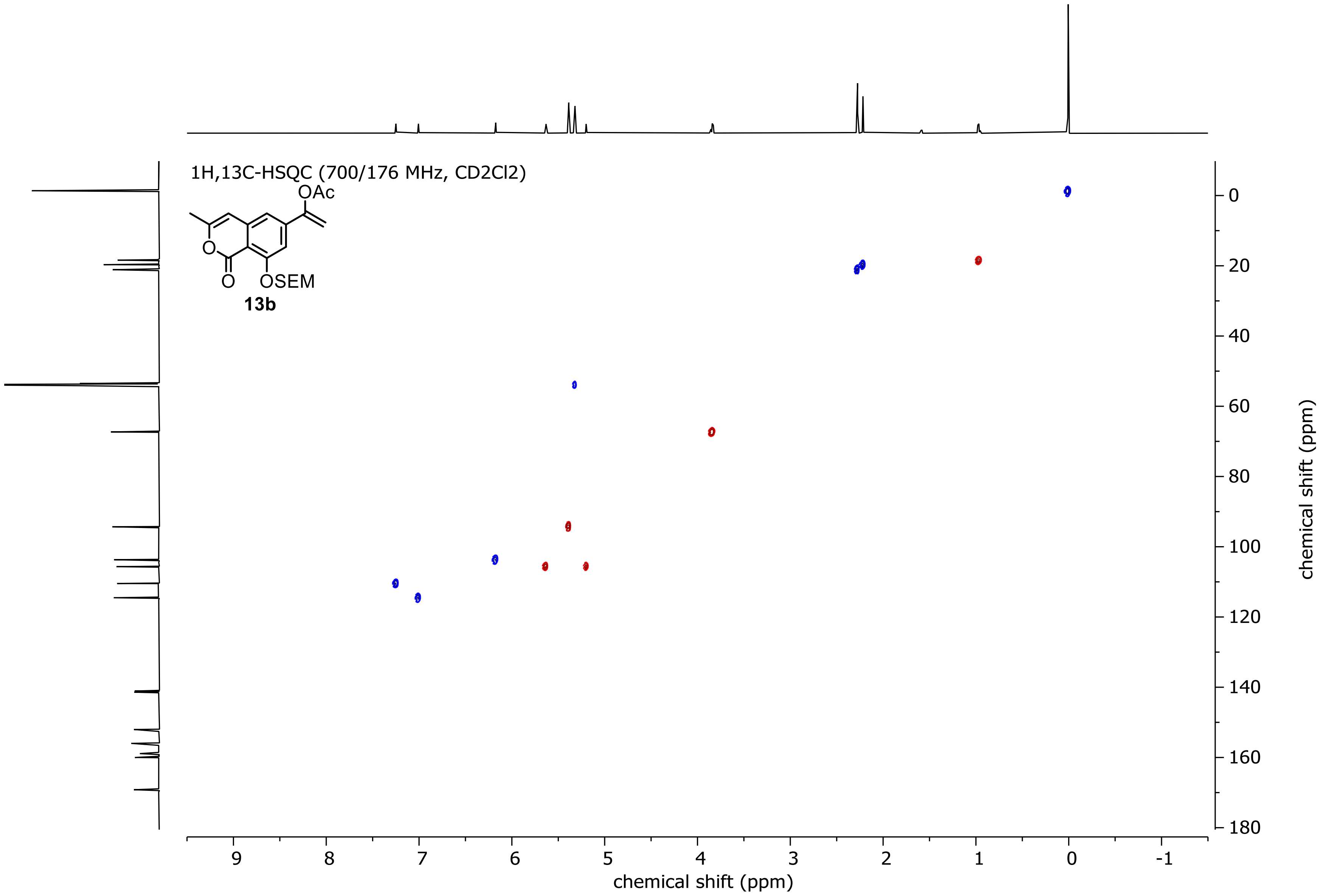
1H,13C-HSQC (700/176 MHz, CD2Cl2)
OAc
O
O
OSEM
13b
chemical shift (ppm)
chemical shift (ppm)

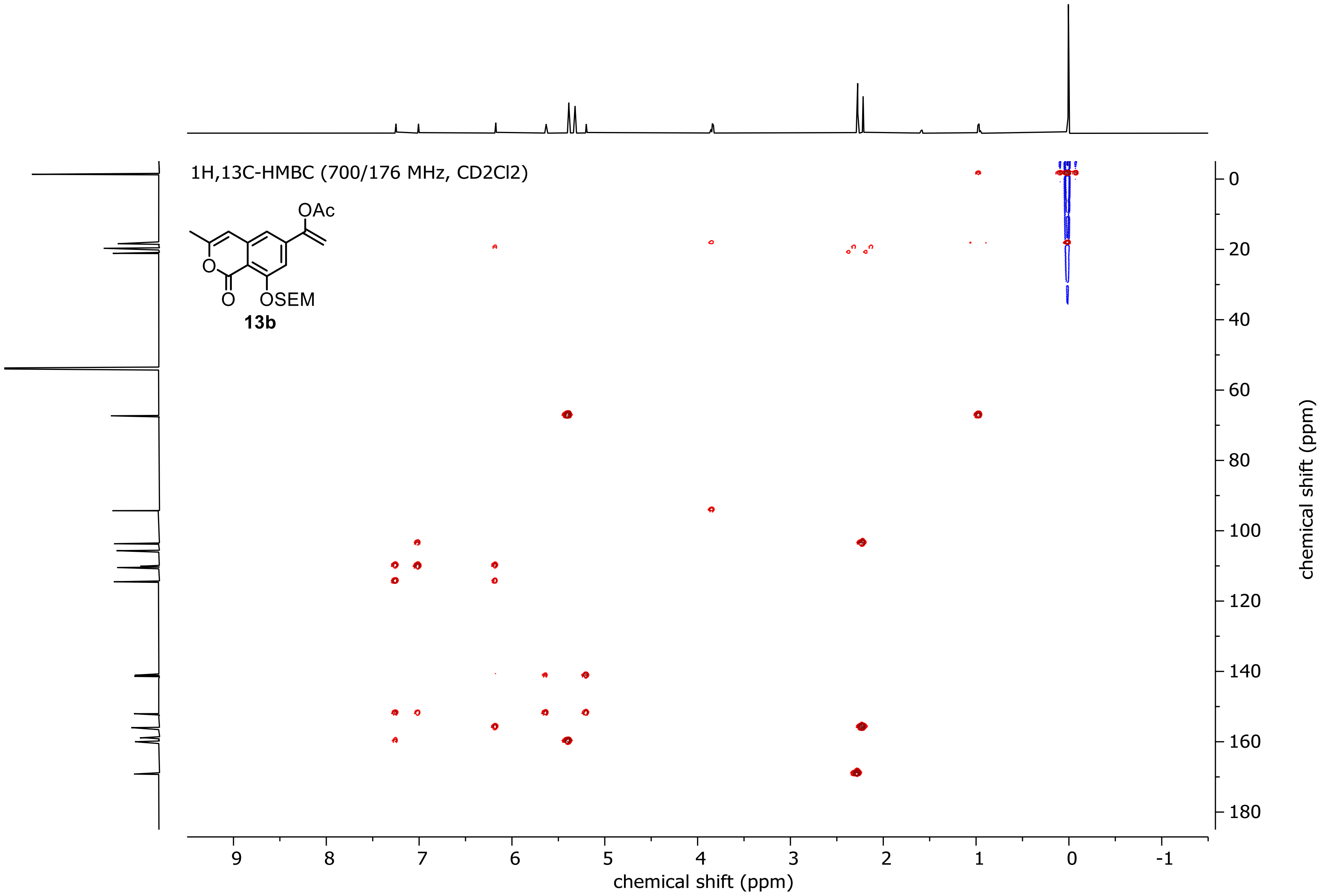
1H,13C-HMBC (700/176 MHz, CD2Cl2)
OAc
O
O
OSEM
13b
chemical shift (ppm)
chemical shift (ppm)

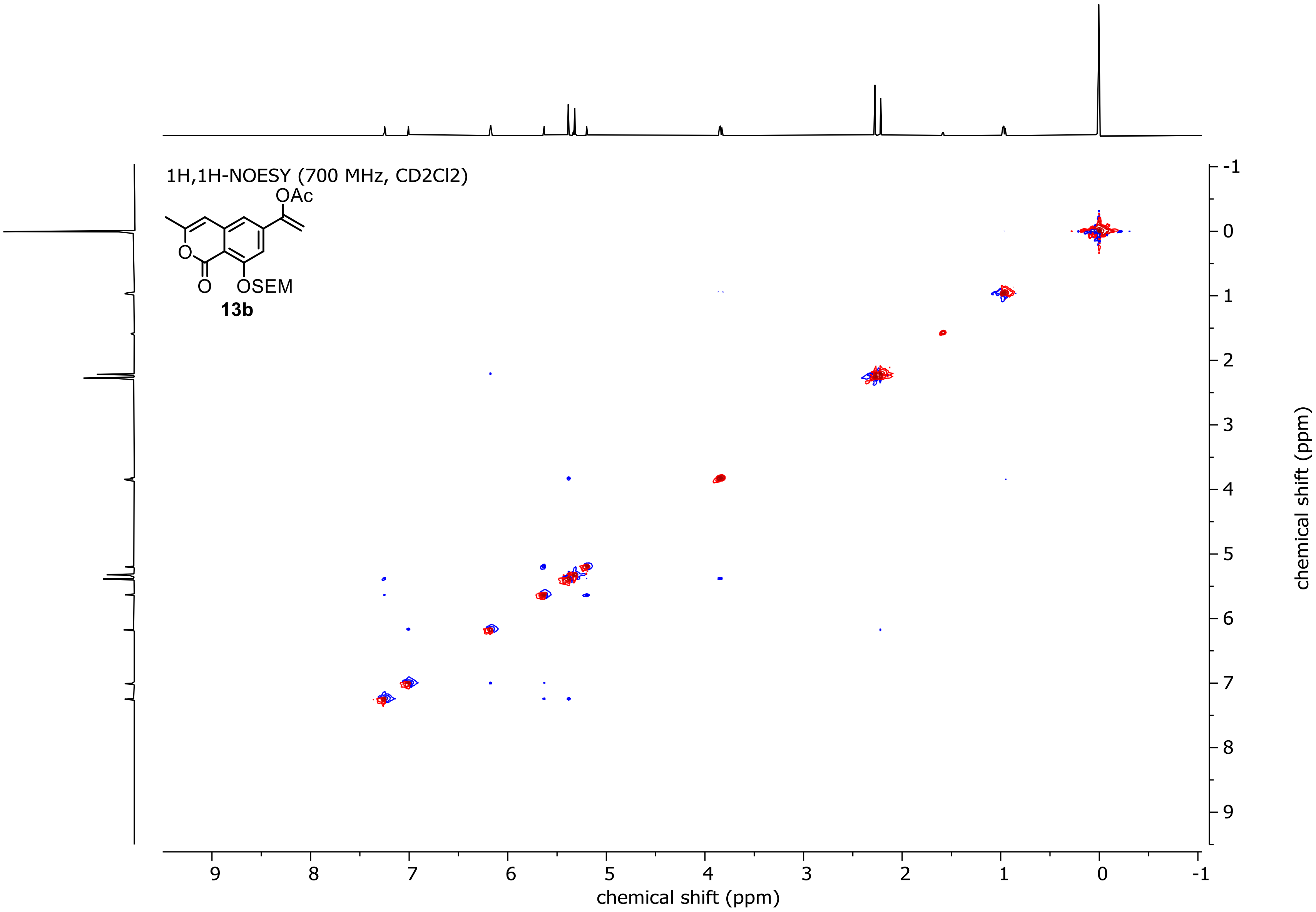
1H,1H-NOESY (700 MHz, CD2Cl2)
OAc
O
O
OSEM
13b
chemical shift (ppm)
chemical shift (ppm)
9
8
7
6
5
4
3
2
1
0
-1

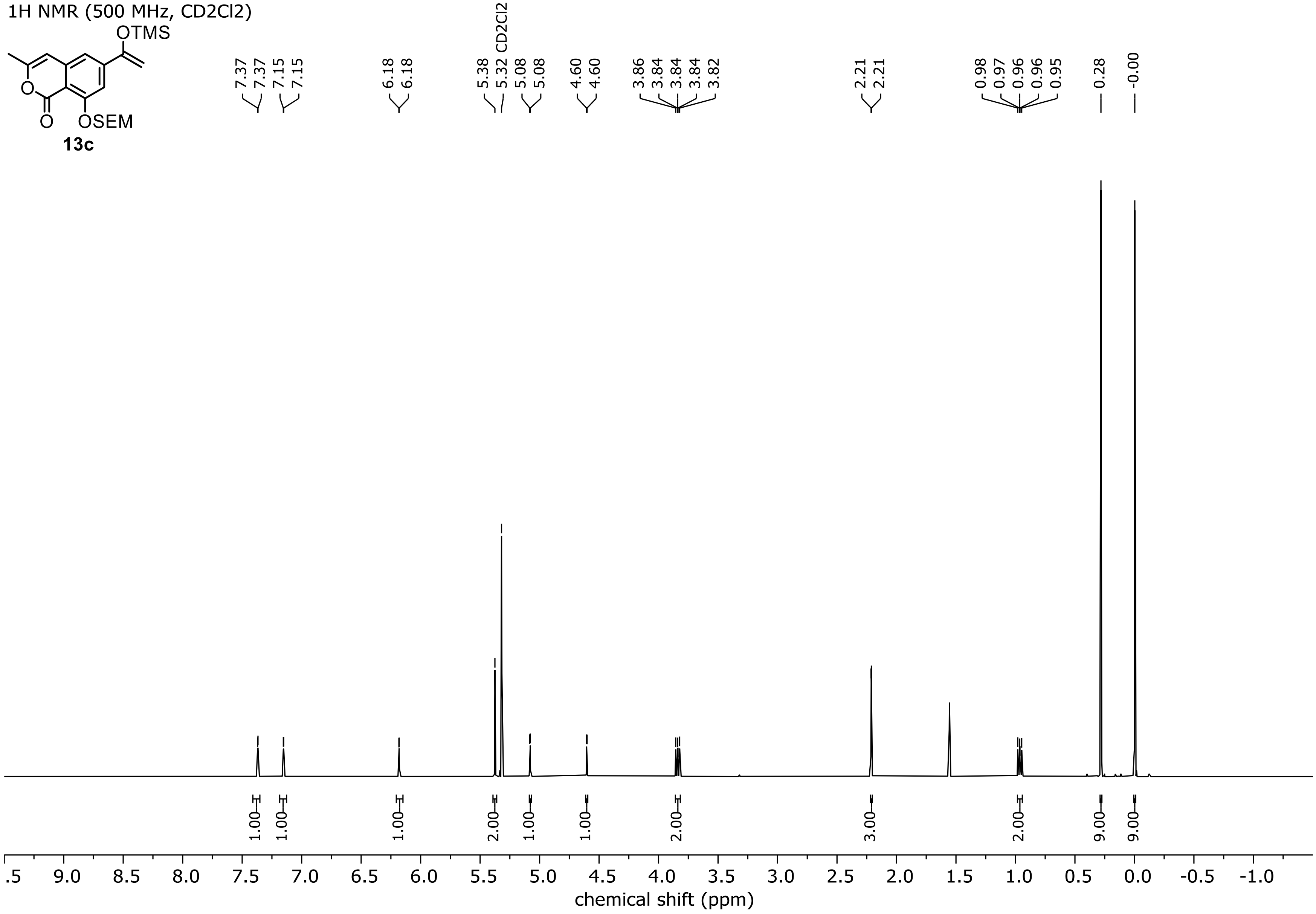
1H NMR (500 MHz, CD2Cl2)
OTMS
O
O
OSEM
13c
7.37
7.37
7.15
7.15
6.18
6.18
5.38
5.32 CD2Cl2
5.08
5.08
4.60
4.60
3.86
3.84
3.84
3.84
3.82
2.21
2.21
0.98
0.97
0.96
0.96
0.95
0.28
-0.00
1.00
1.00
1.00
2.00
1.00
1.00
2.00
3.00
2.00
9.00
9.00
.5
9.0
8.5
8.0
7.5
7.0
6.5
6.0
5.5
5.0
4.5
4.0
3.5
3.0
2.5
2.0
1.5
1.0
0.5
0.0
-0.5
-1.0
chemical shift (ppm)

13C NMR (125 MHz, CD2Cl2)

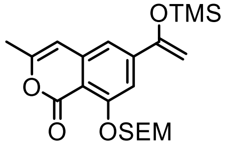

13c

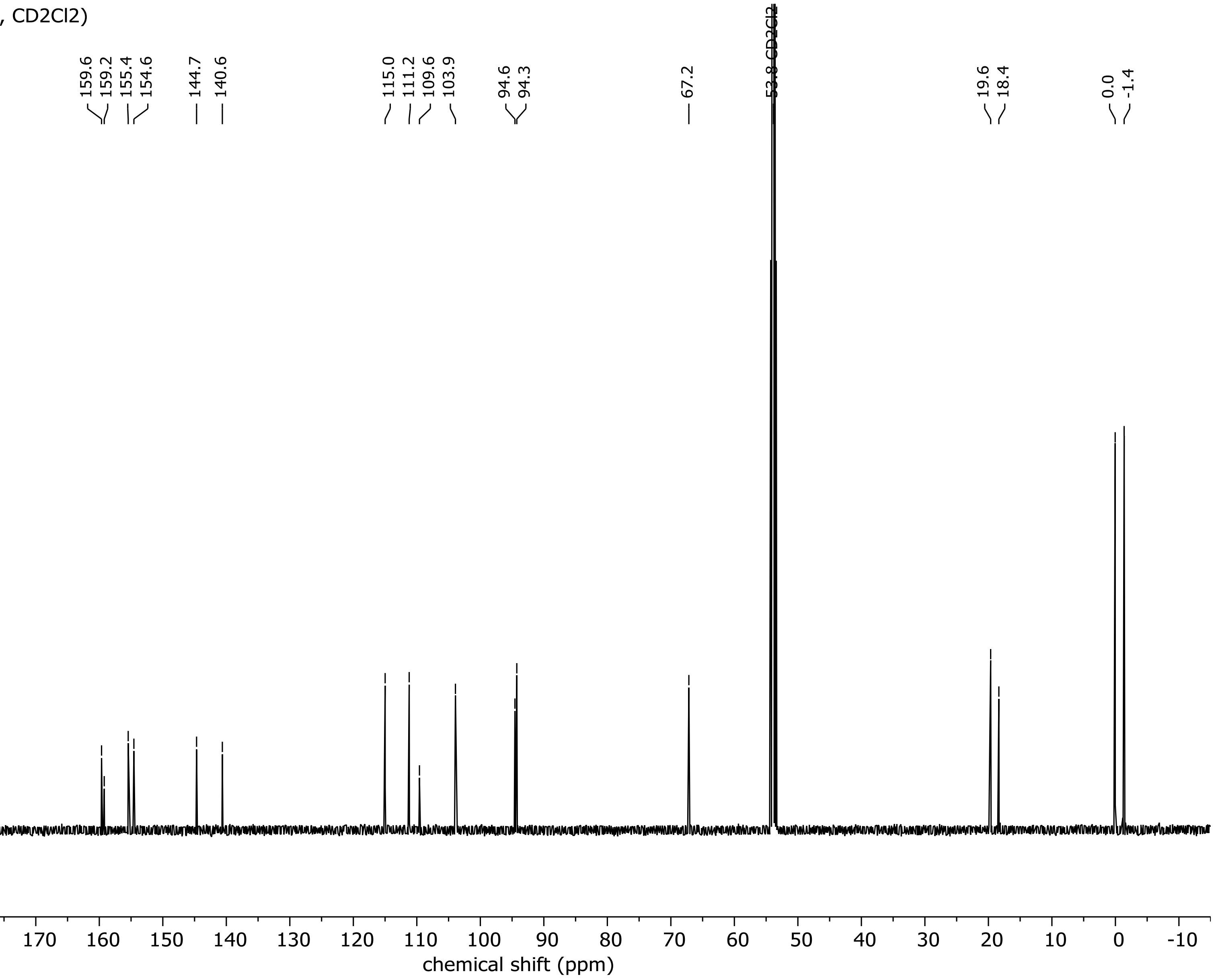

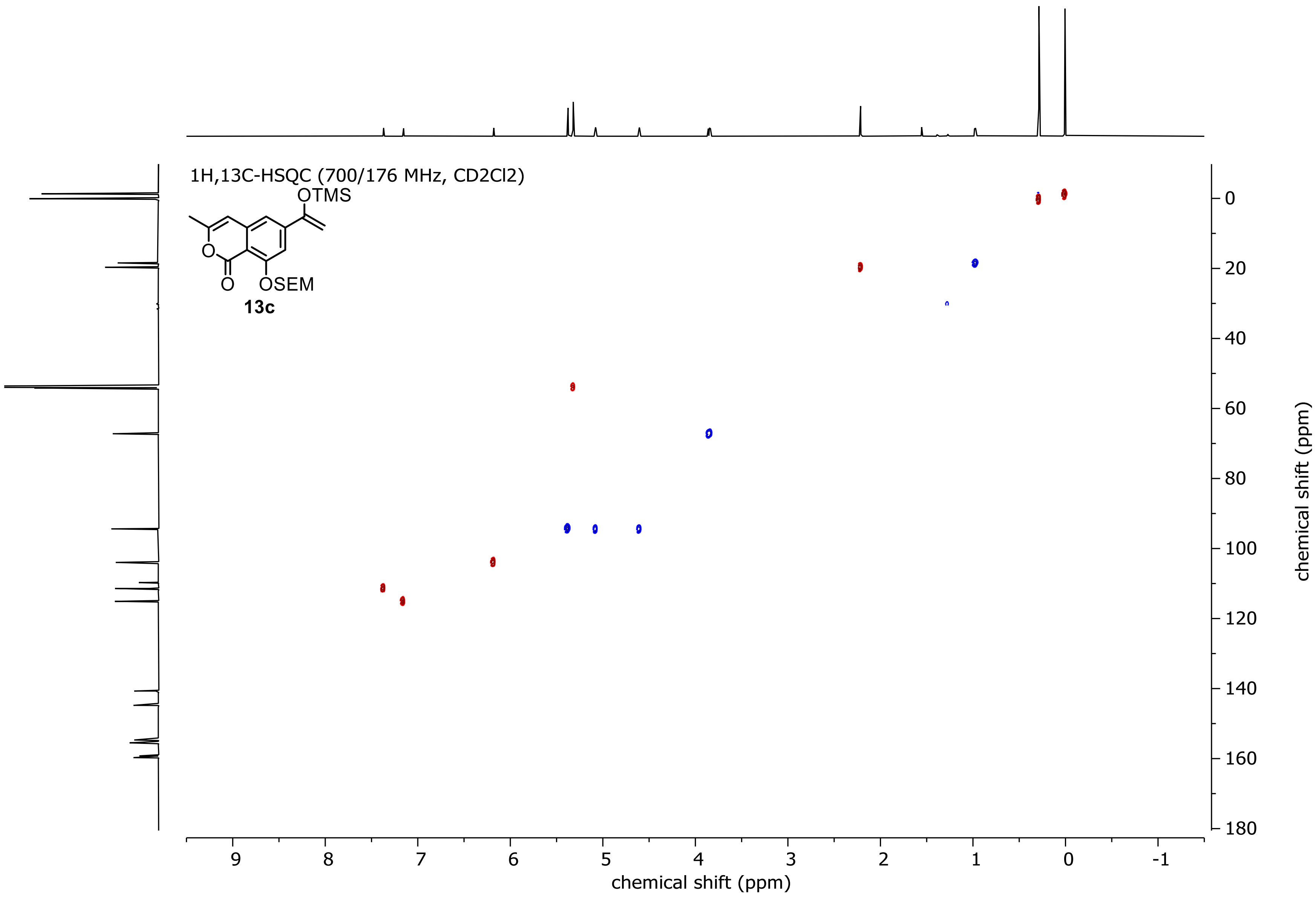
1H,13C-HSQC (700/176 MHz, CD2Cl2)
OTMS
O
O
OSEM
13c
9
8
7
6
5
4
3
2
1
0
-1
chemical shift (ppm)
0
20
40
60
80
100
120
140
160
180
chemical shift (ppm)

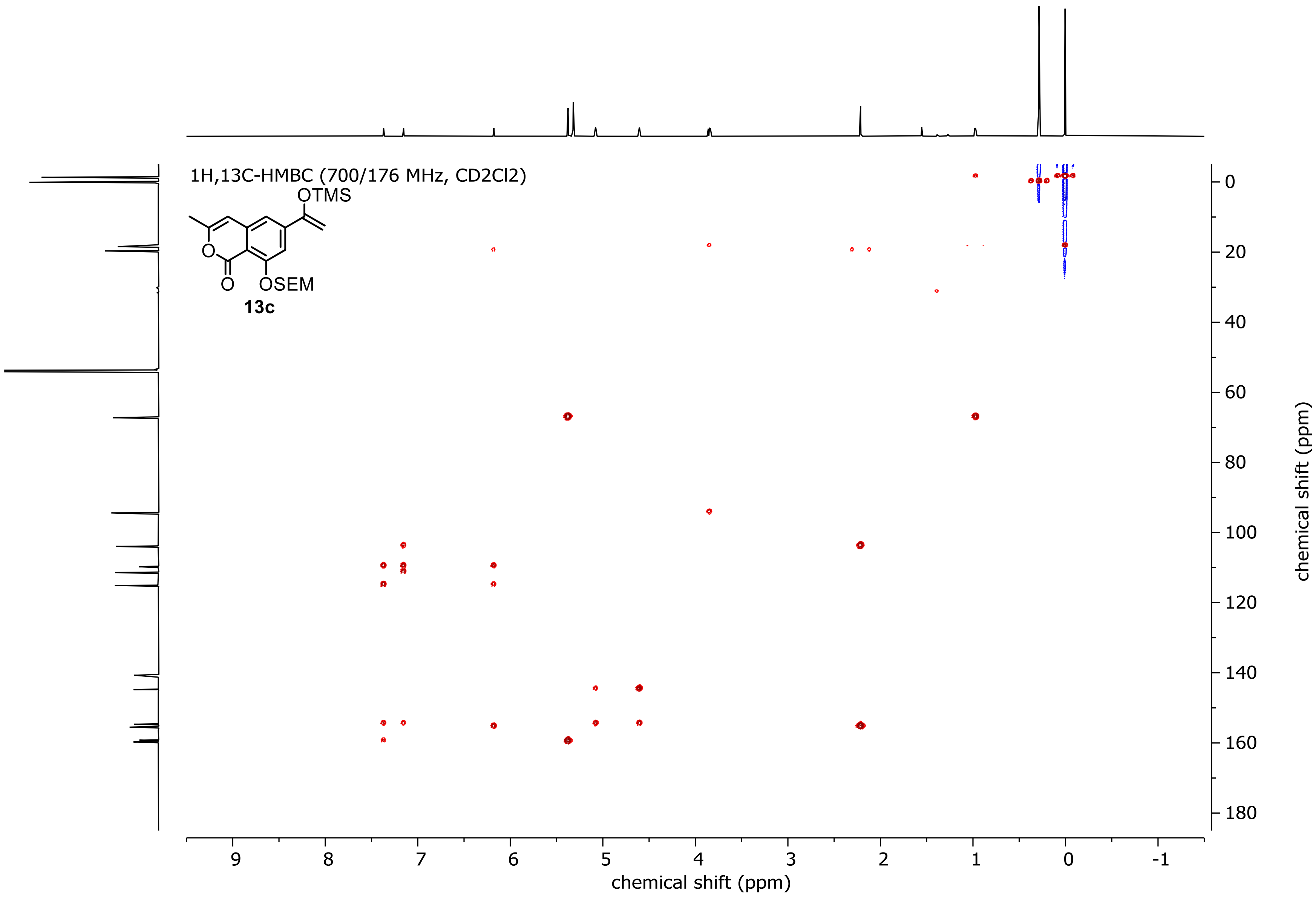
1H,13C-HMBC (700/176 MHz, CD2Cl2)
OTMS
O
O
OSEM
13c
chemical shift (ppm)
chemical shift (ppm)
0
20
40
60
80
100
120
140
160
180
9
8
7
6
5
4
3
2
1
0
-1

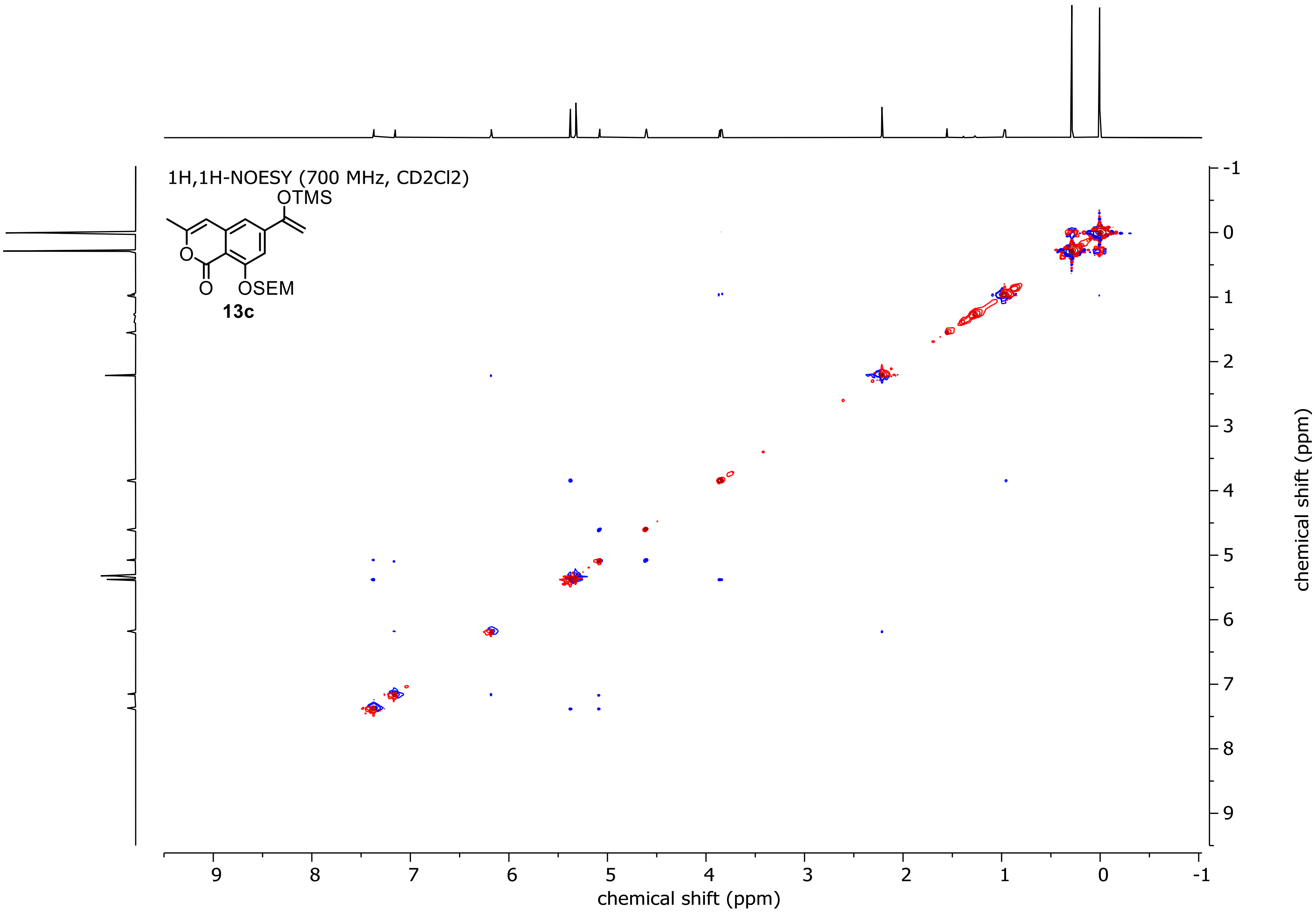
1H,1H-NOESY (700 MHz, CD2Cl2)
OTMS
O
O
OSEM
13c
chemical shift (ppm)
chemical shift (ppm)

7.2 Leading to Xanthone **14**

1H NMR (500 MHz, CD2Cl2)

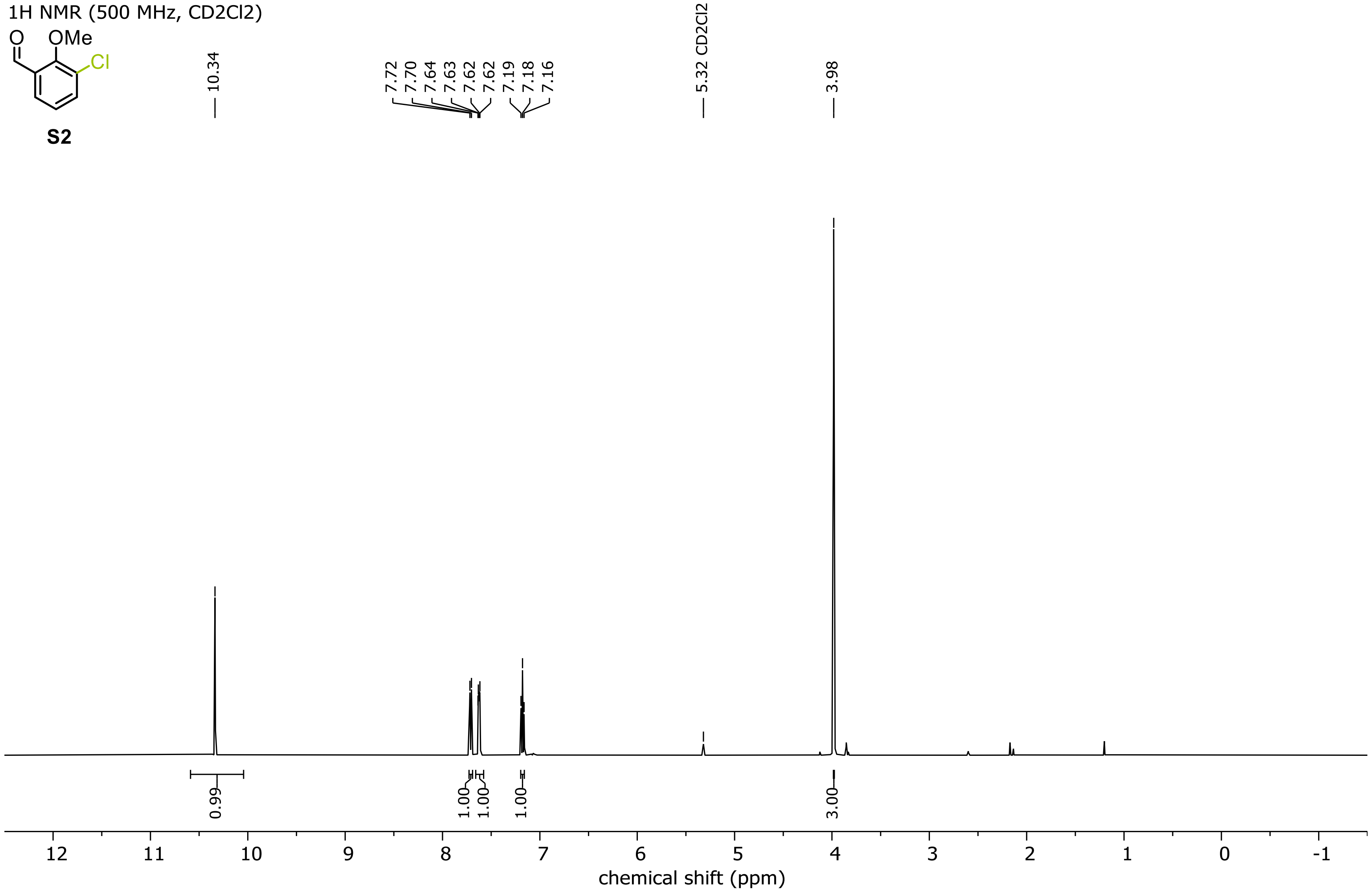

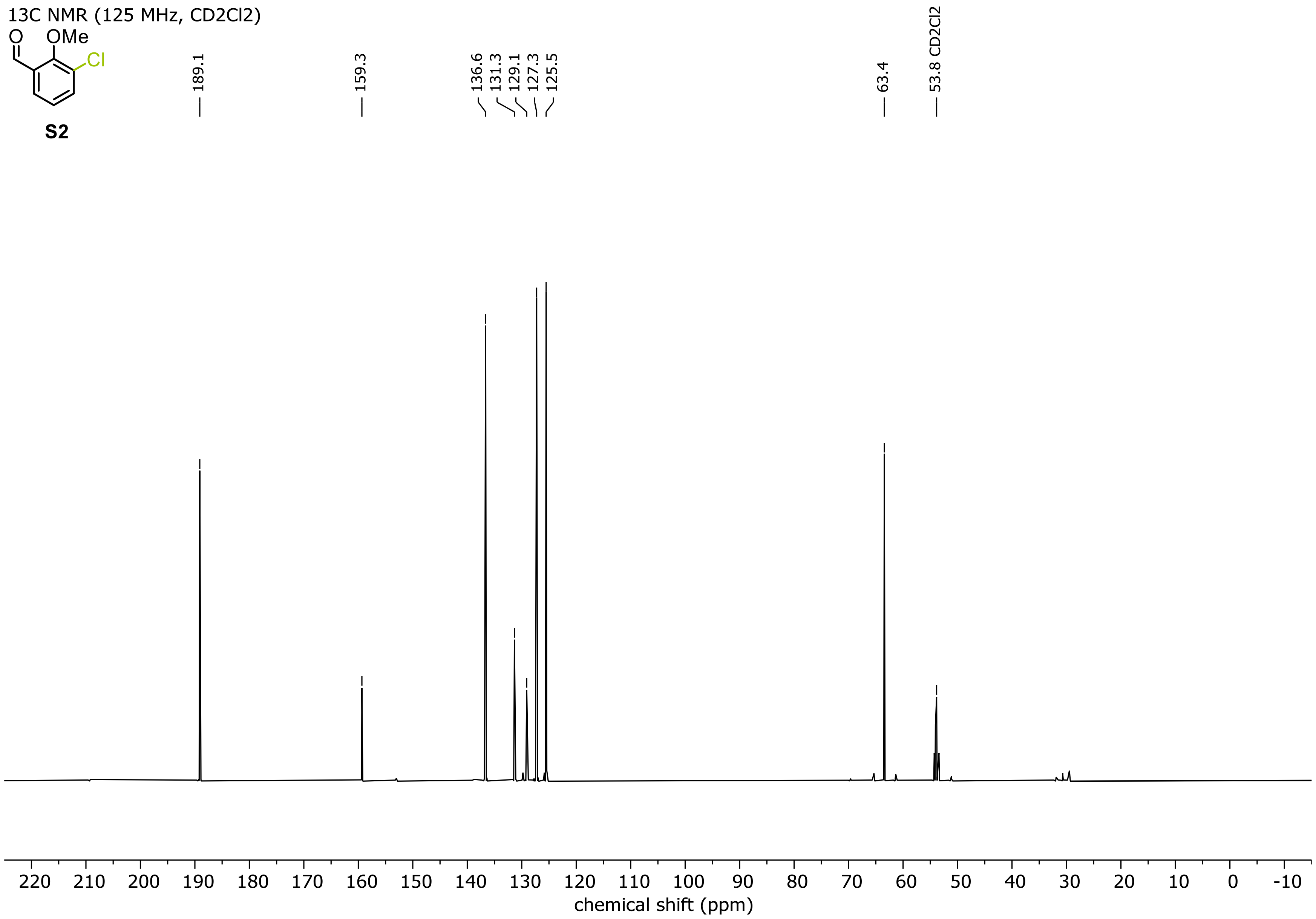
13C NMR (125 MHz, CD2Cl2)
O
OMe
Cl
S2
189.1
159.3
136.6
131.3
129.1
127.3
125.5
63.4
53.8 CD2Cl2
220 210 200 190 180 170 160 150 140 130 120 110 100 90 80 70 60 50 40 30 20 10 0 -10
chemical shift (ppm)

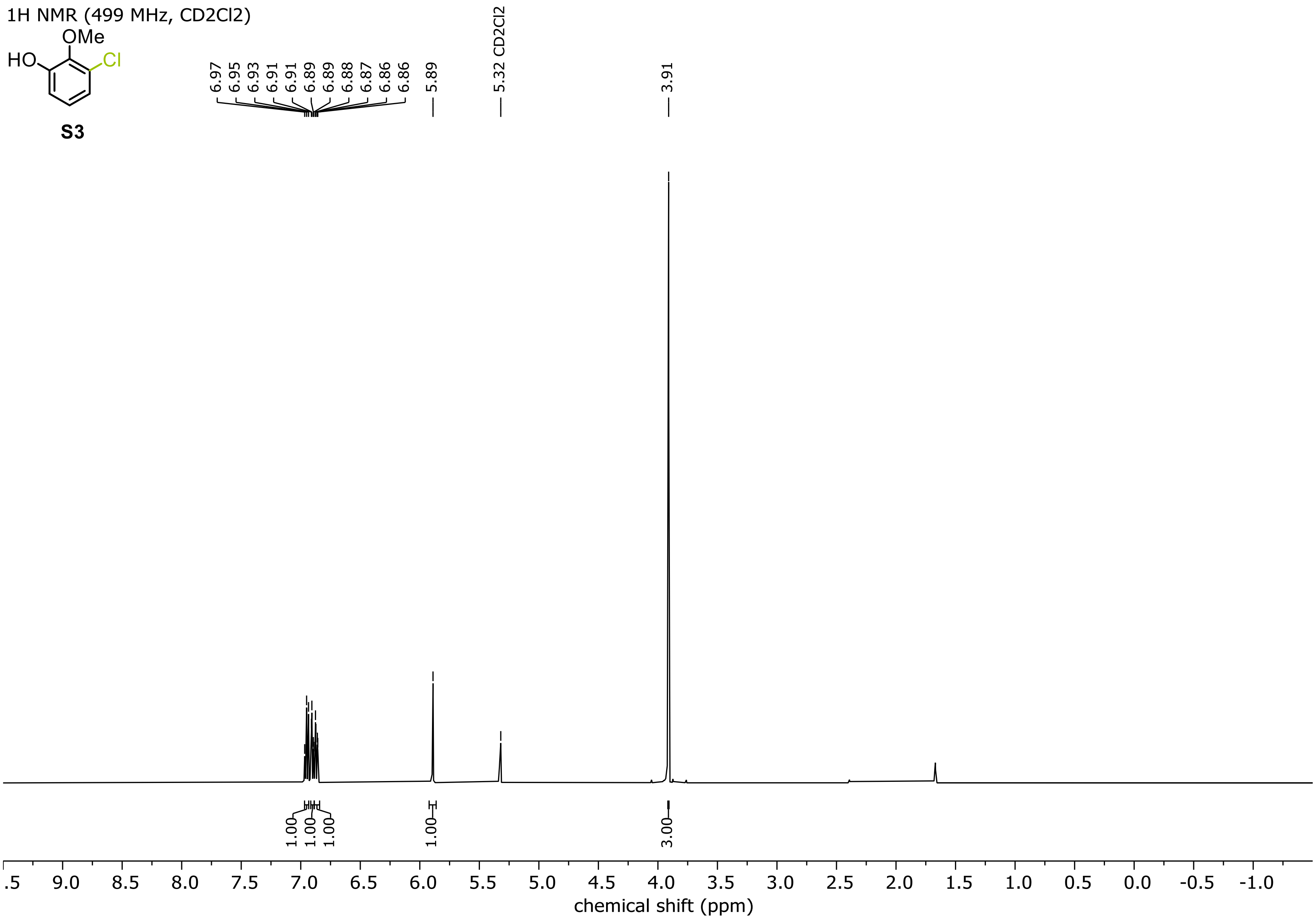
1H NMR (499 MHz, CD2Cl2)
OMe
HO
Cl
S3
6.97
6.95
6.93
6.91
6.91
6.89
6.89
6.88
6.87
6.86
6.86
5.89
5.32 CD2Cl2
3.91
1.00
1.00
1.00
1.00
3.00
.5
9.0
8.5
8.0
7.5
7.0
6.5
6.0
5.5
5.0
4.5
4.0
3.5
3.0
2.5
2.0
1.5
1.0
0.5
0.0
-0.5
-1.0
chemical shift (ppm)

13C NMR (125 MHz, CD2Cl2)

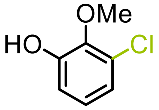

S3

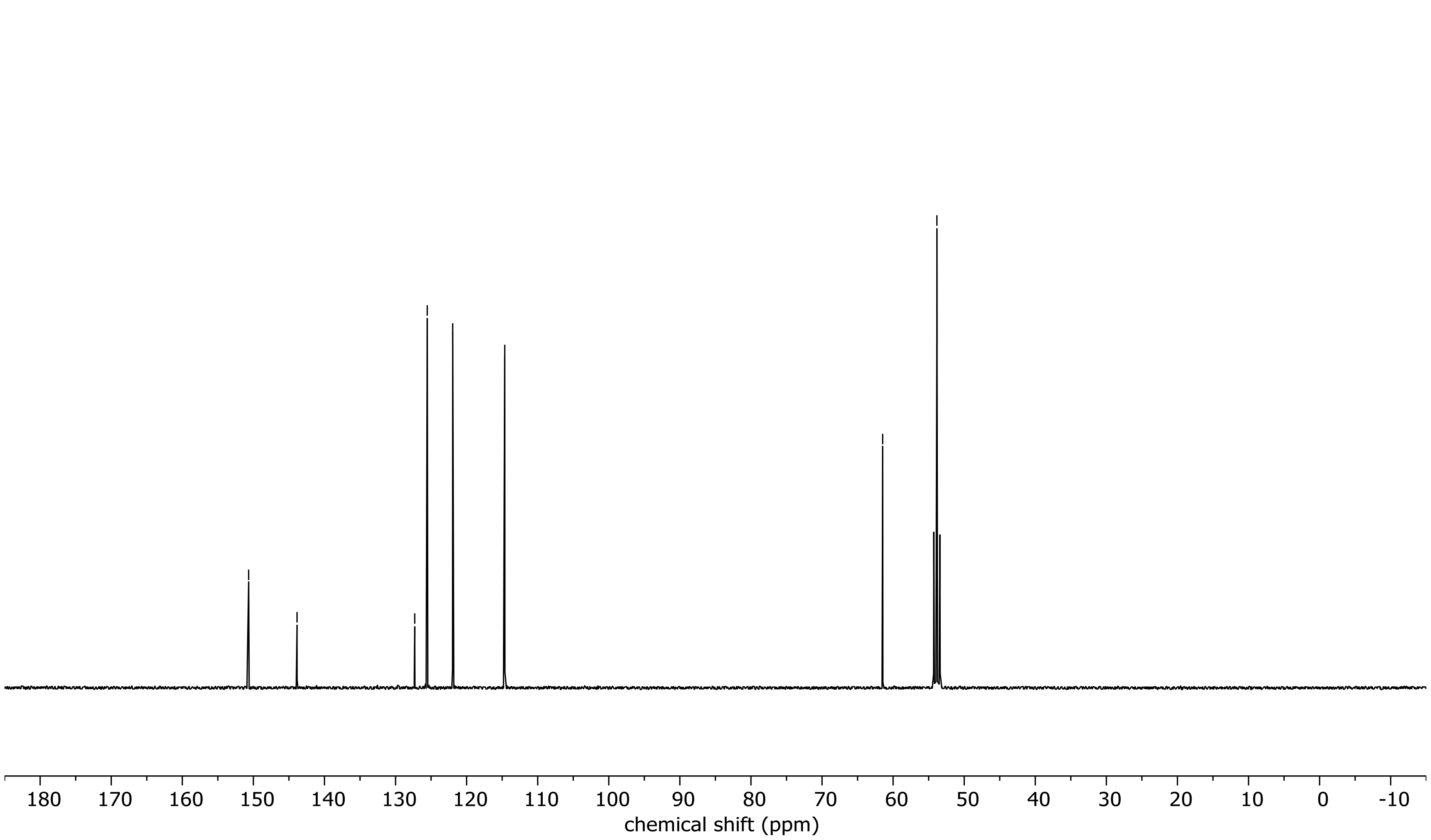

1H NMR (500 MHz, CD2Cl2)

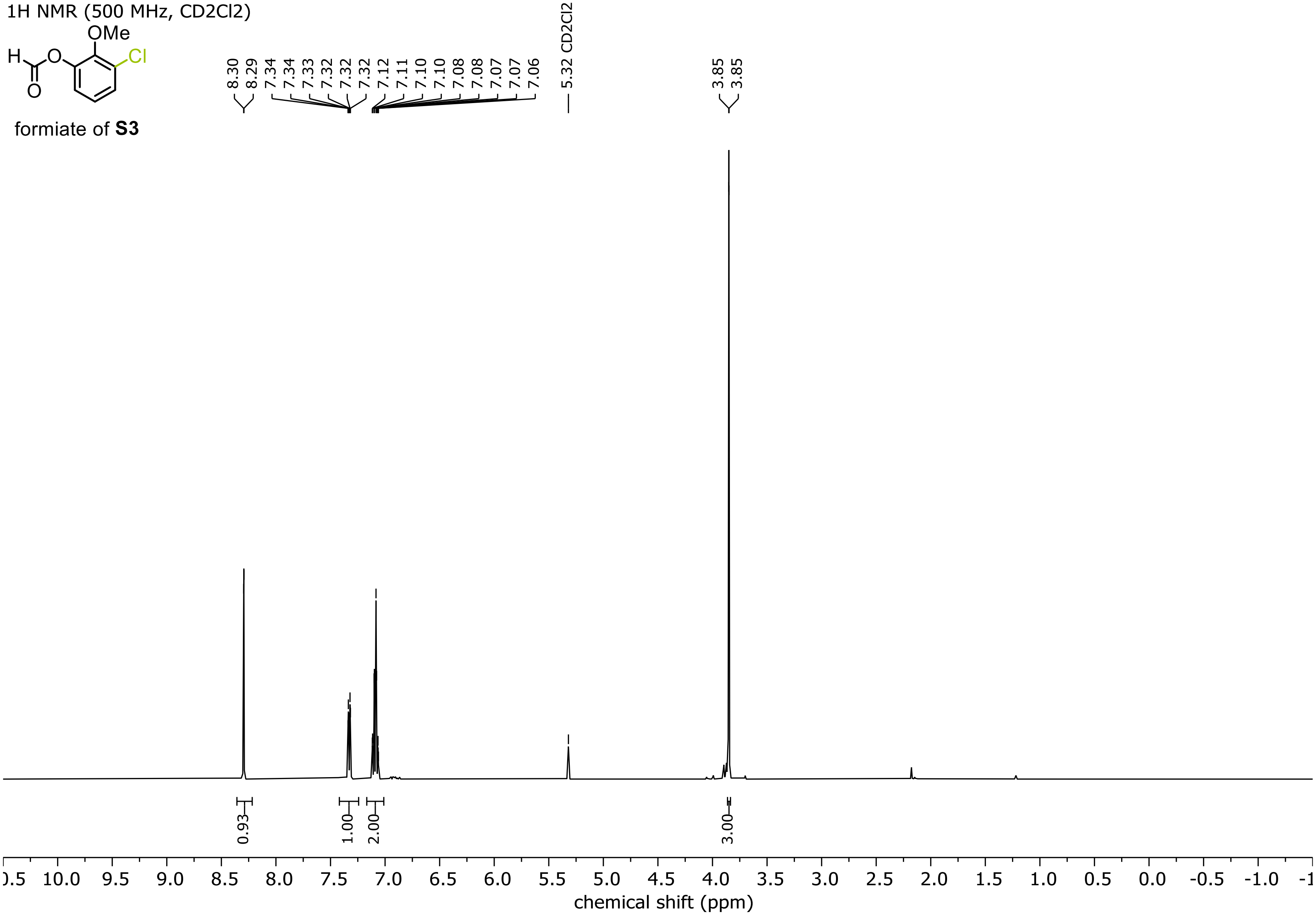

13C NMR (125 MHz, CD2Cl2)

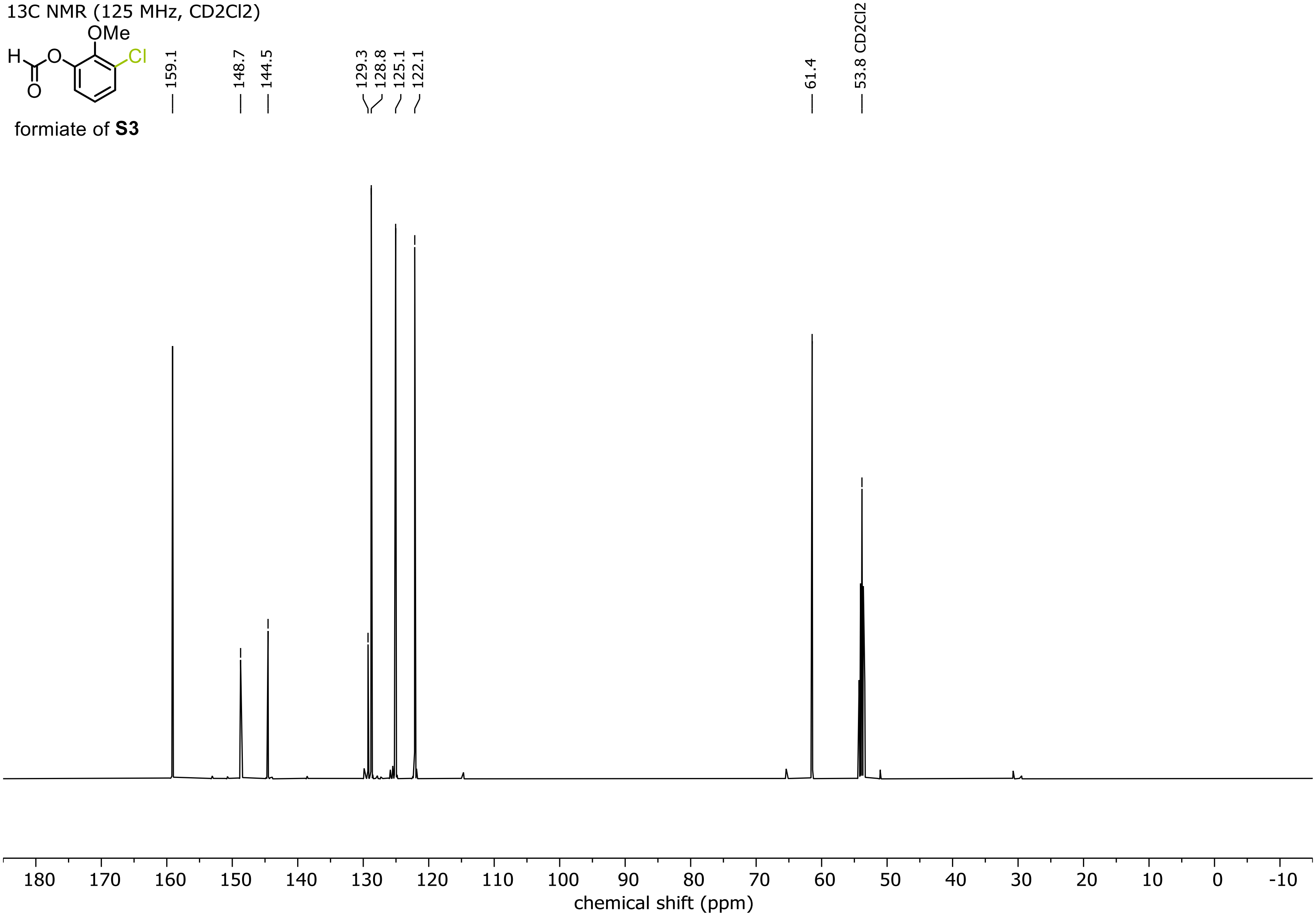

formiate of **S3**

1H NMR (500 MHz, CD2Cl2)

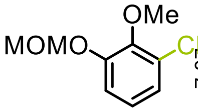

24

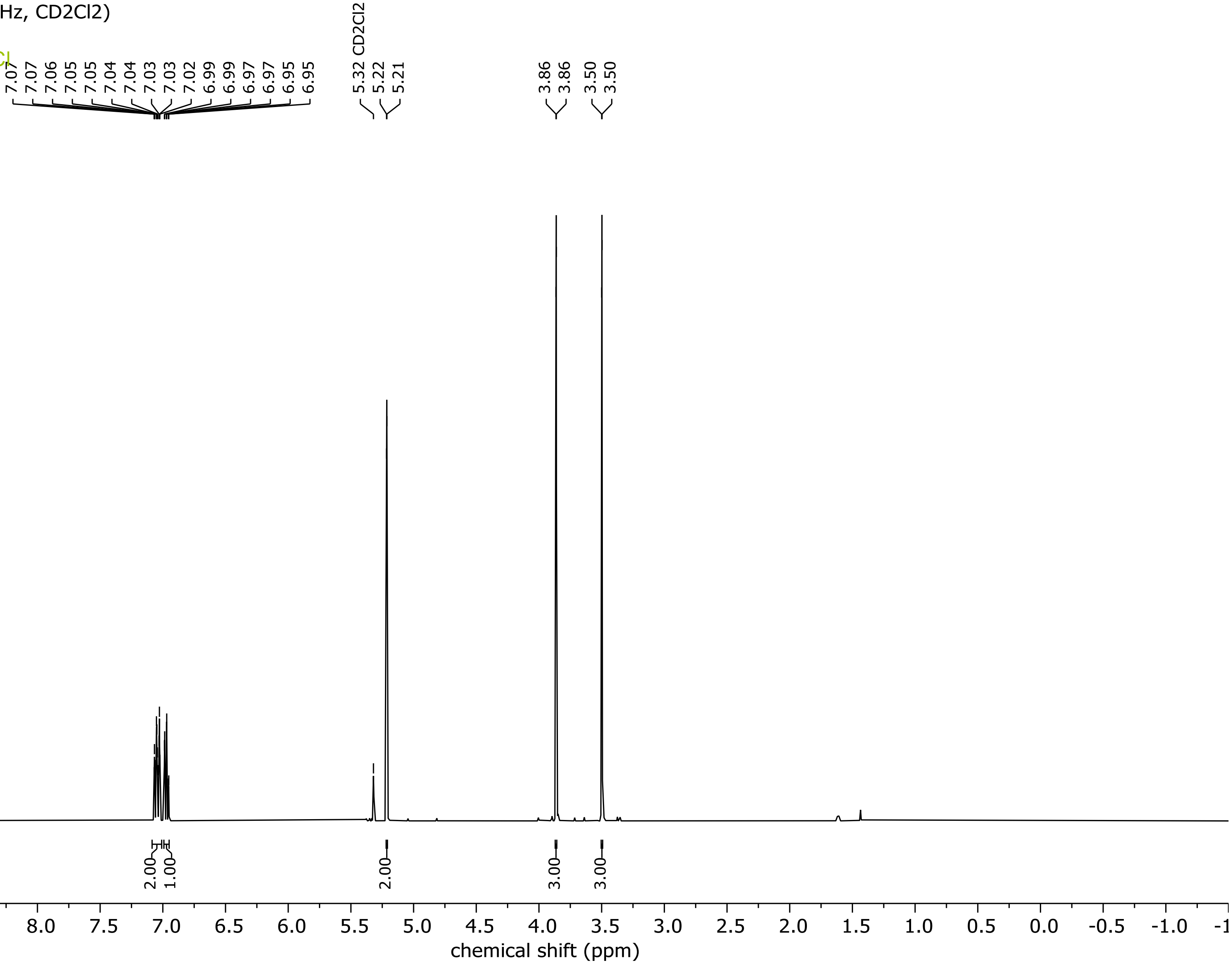

13C NMR (125 MHz, CD2Cl2)

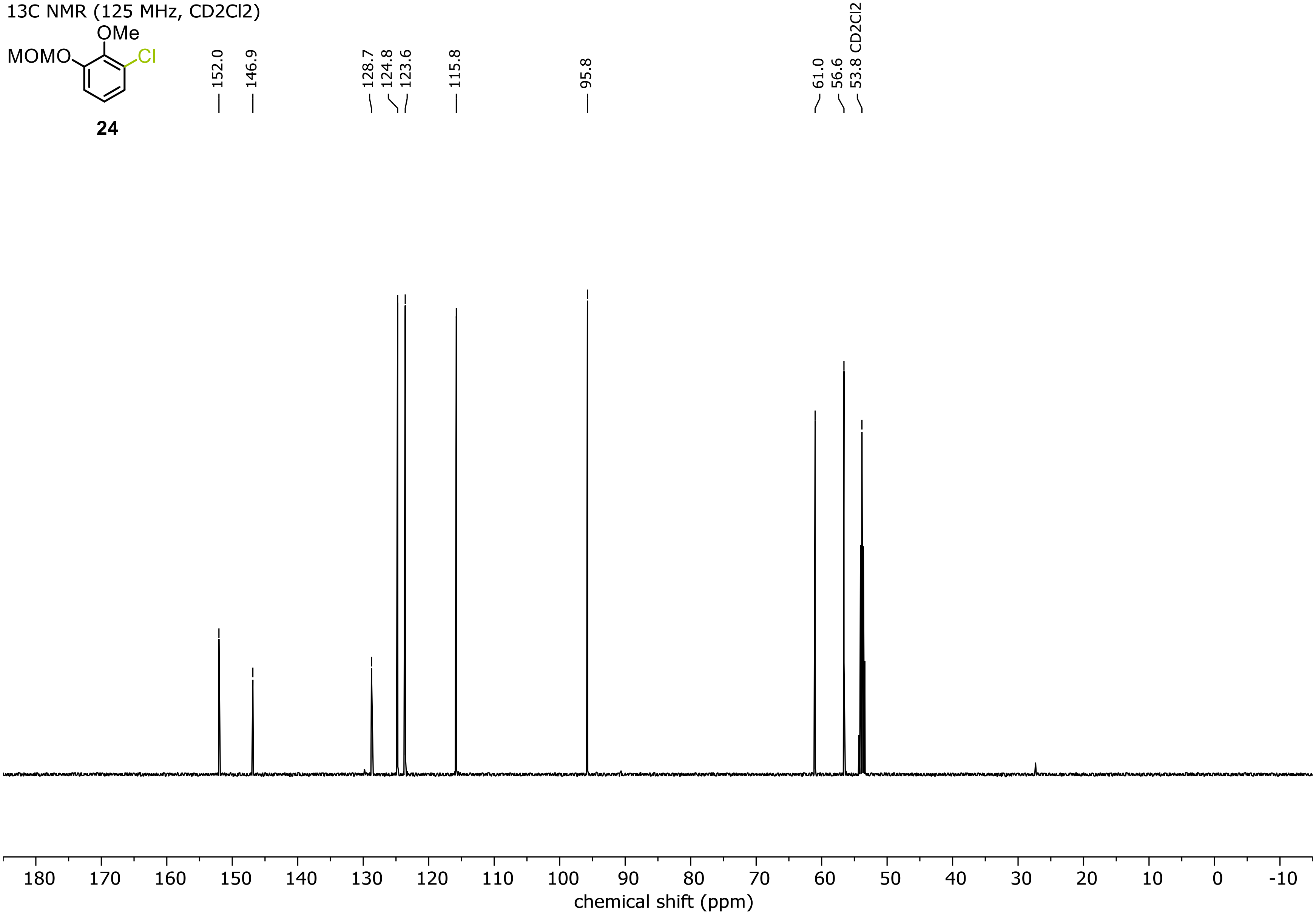

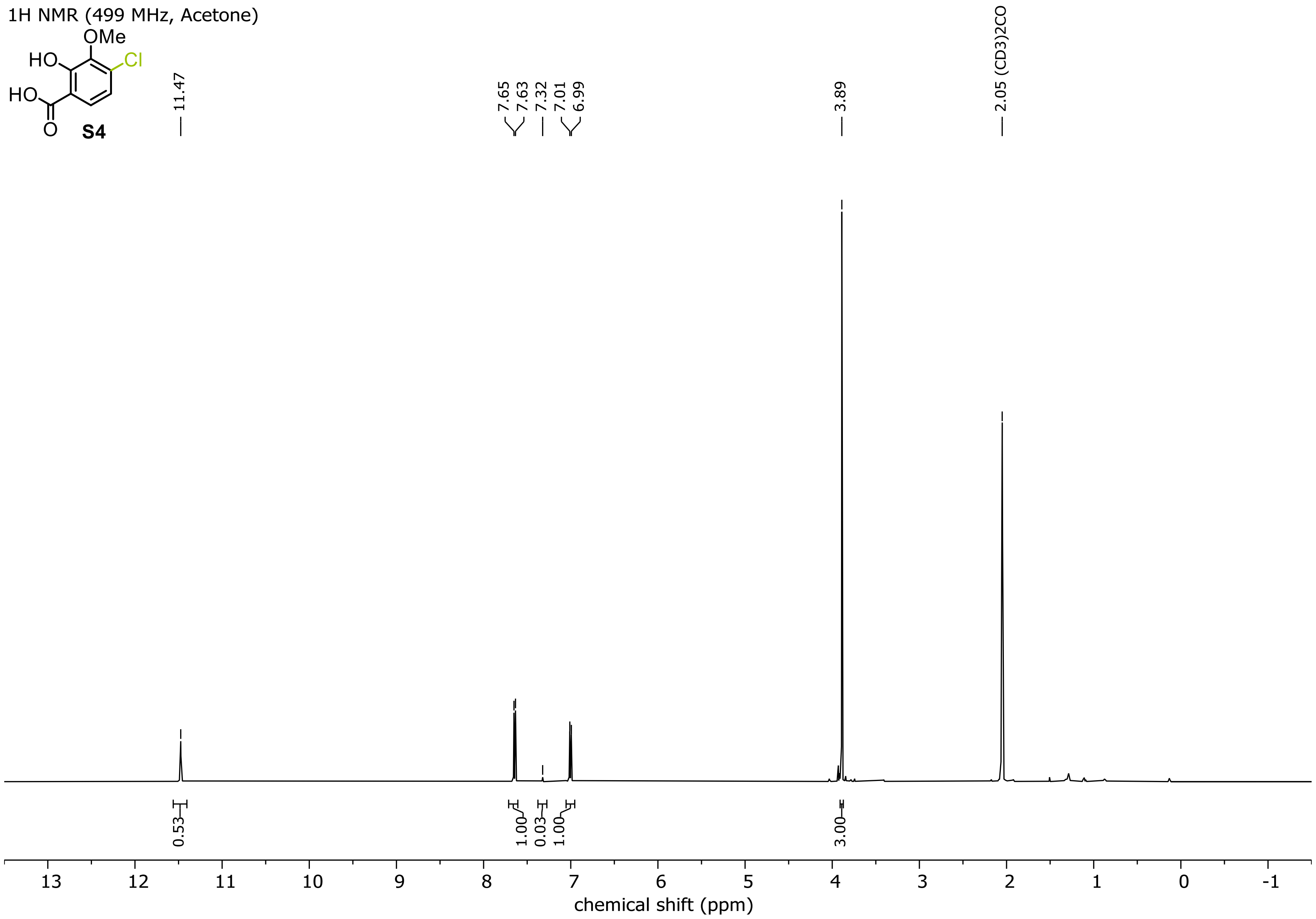
1H NMR (499 MHz, Acetone)
OMe
HO
Cl
HO
O
S4
11.47
7.65
7.63
7.32
7.01
6.99
3.89
2.05 (CD3)2CO
0.53
1.00
0.03
1.00
3.00
13
12
11
10
9
8
7
6
5
4
3
2
1
0
-1
chemical shift (ppm)

13C NMR (125 MHz, Acetone)

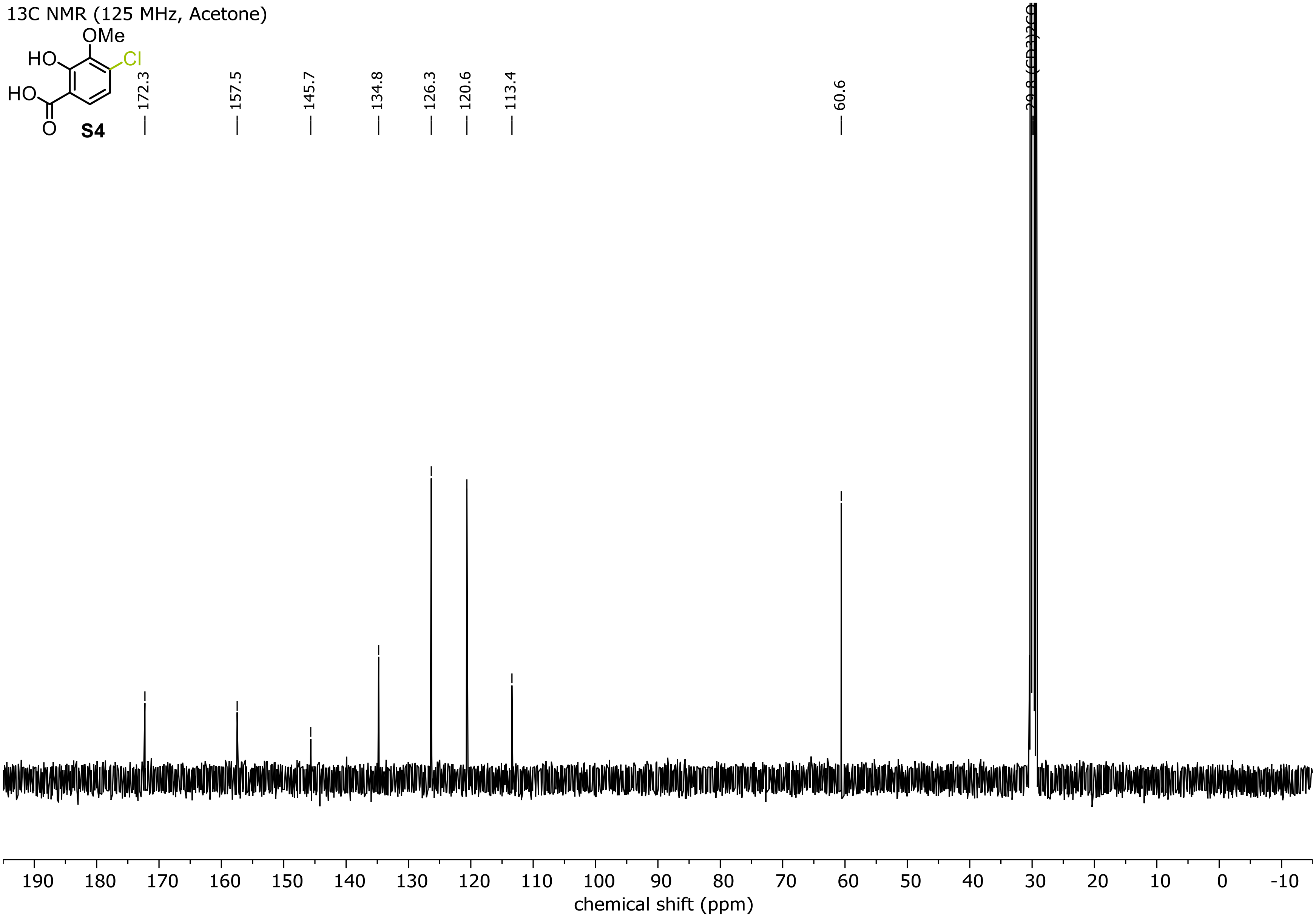

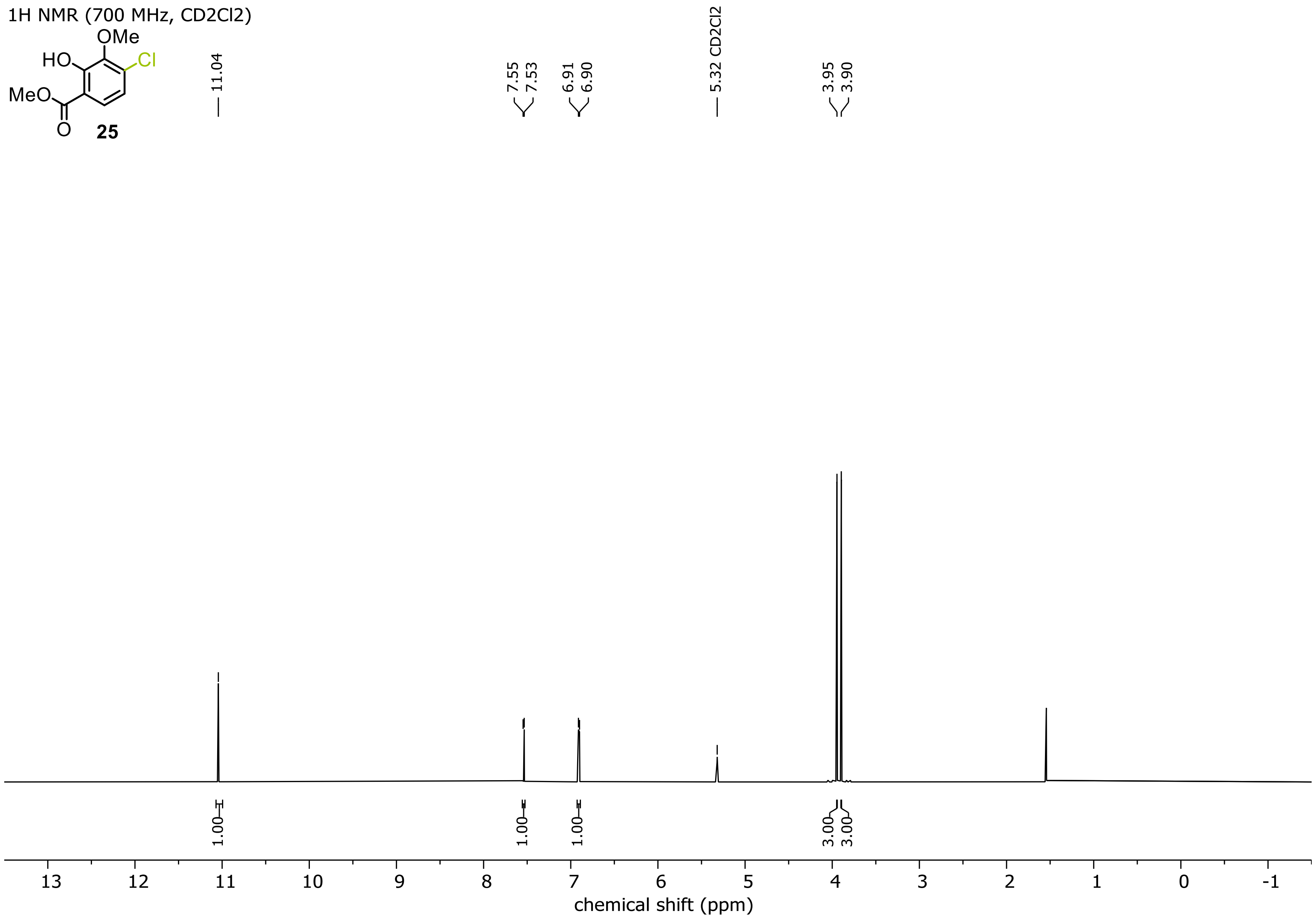
1H NMR (700 MHz, CD2Cl2)
OMe
HO
Cl
MeO
O
25
11.04
7.55
7.53
6.91
6.90
5.32 CD2Cl2
3.95
3.90
1.00
1.00
1.00
3.00
3.00
13
12
11
10
9
8
7
6
5
4
3
2
1
0
-1
chemical shift (ppm)

13C NMR (176 MHz, CD2Cl2)

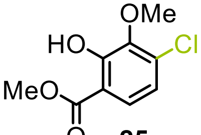

25

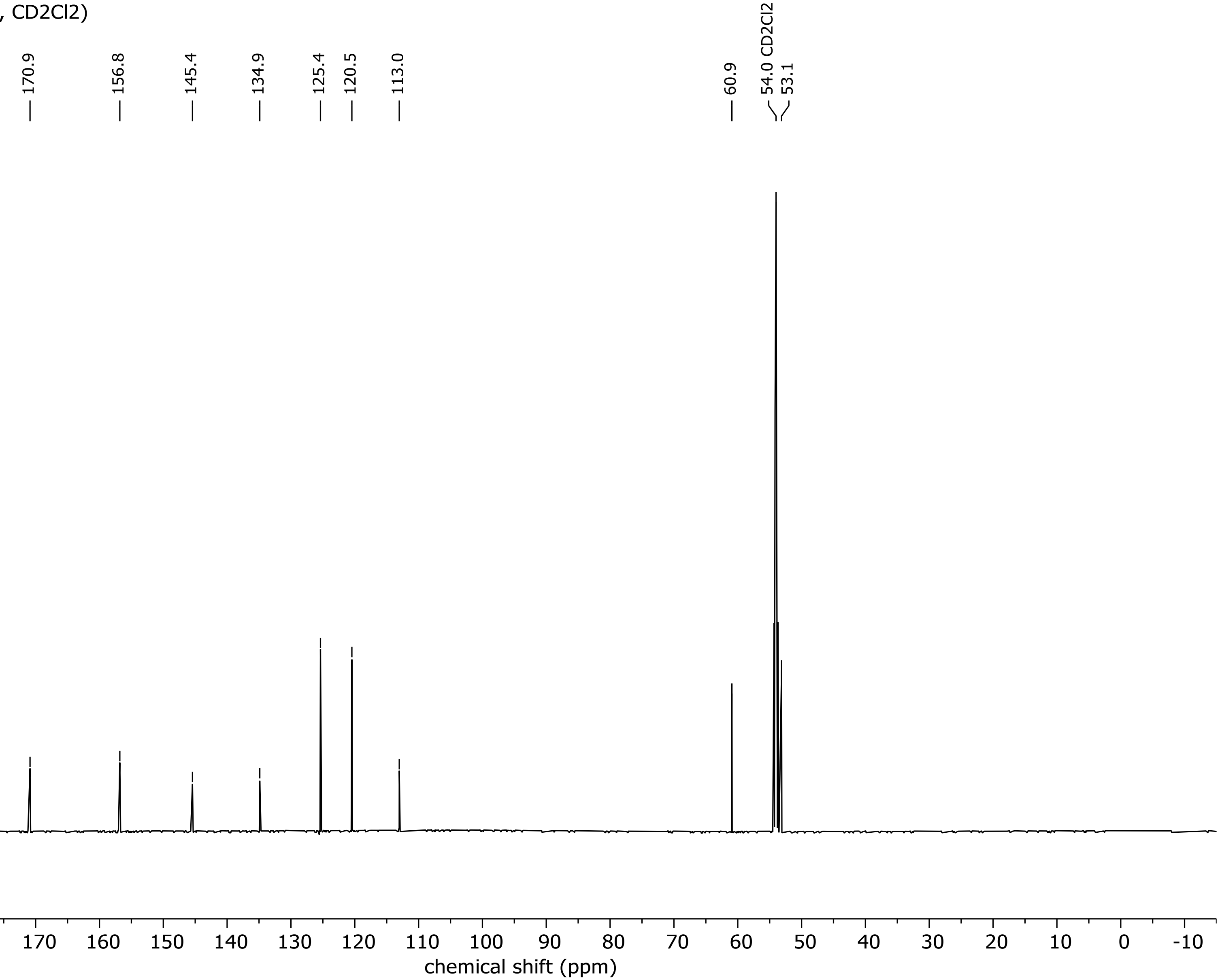

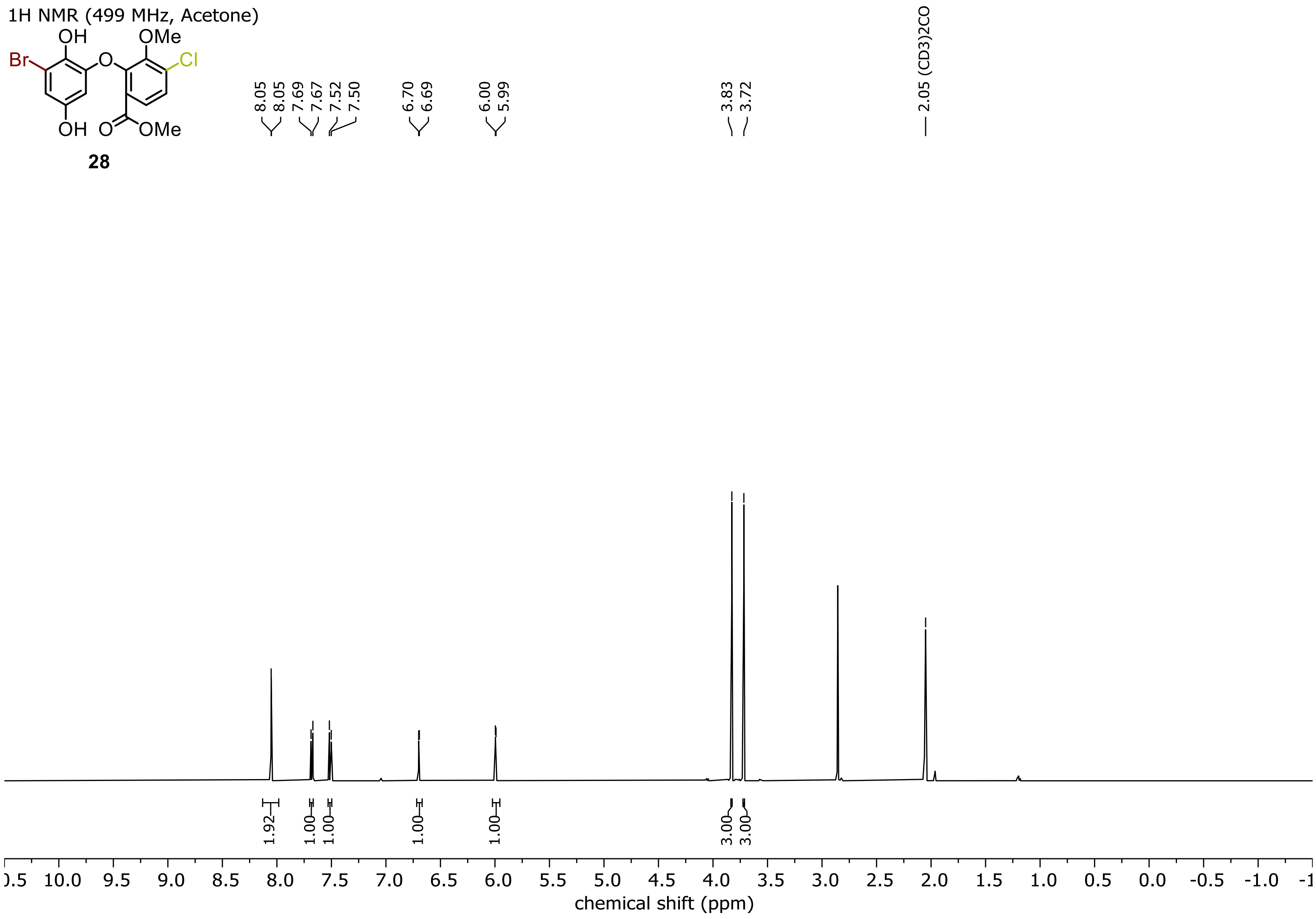
1H NMR (499 MHz, Acetone)
OH
OMe
Br
O
Cl
OH
O
OMe
28
8.05
8.05
7.69
7.67
7.52
7.50
6.70
6.69
6.00
5.99
3.83
3.72
2.05 (CD3)2CO
1.92
1.00
1.00
1.00
1.00
3.00
3.00
chemical shift (ppm)

13C NMR (125 MHz, Acetone)

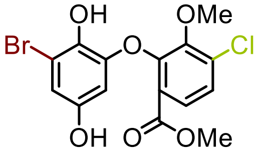

28

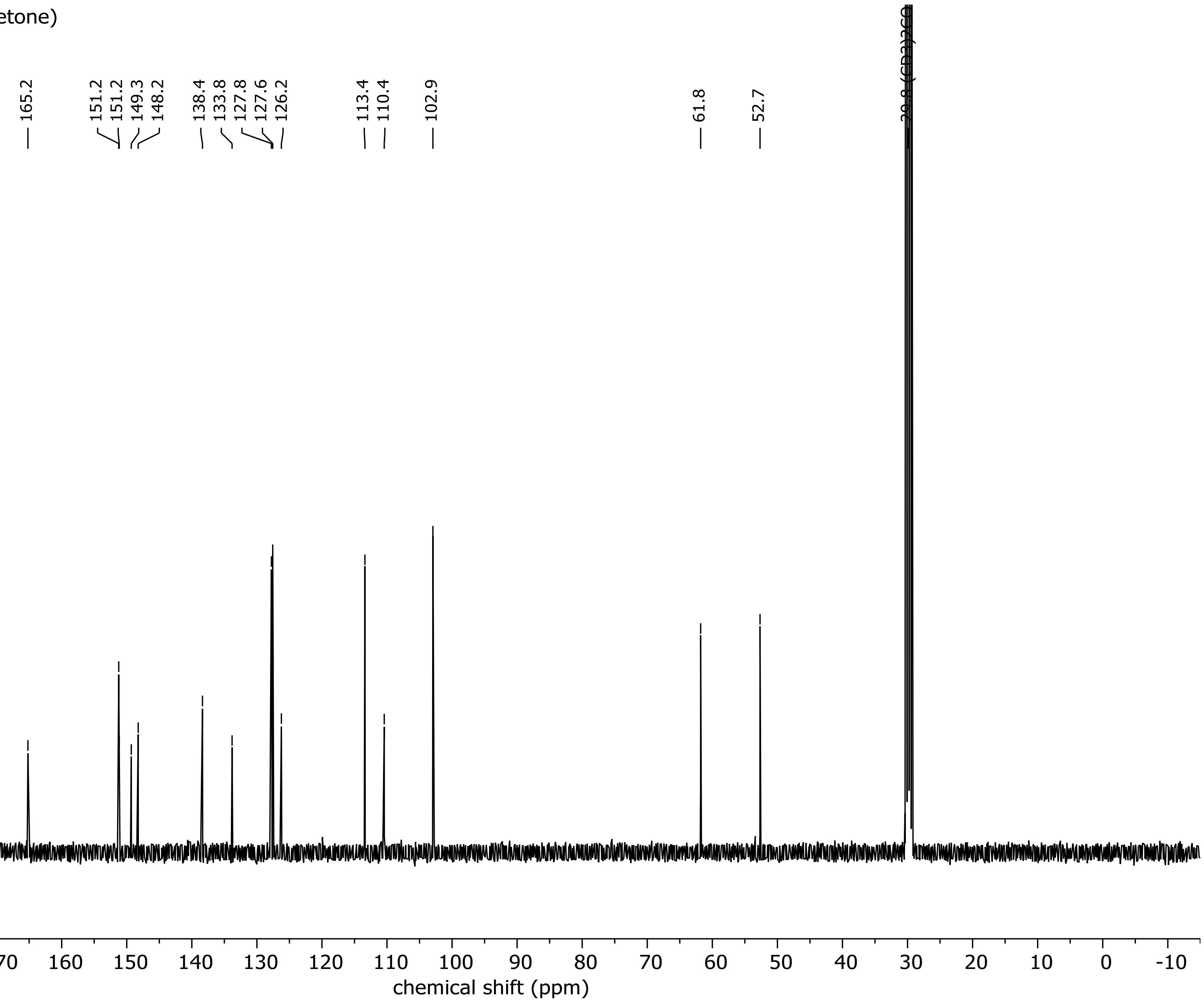

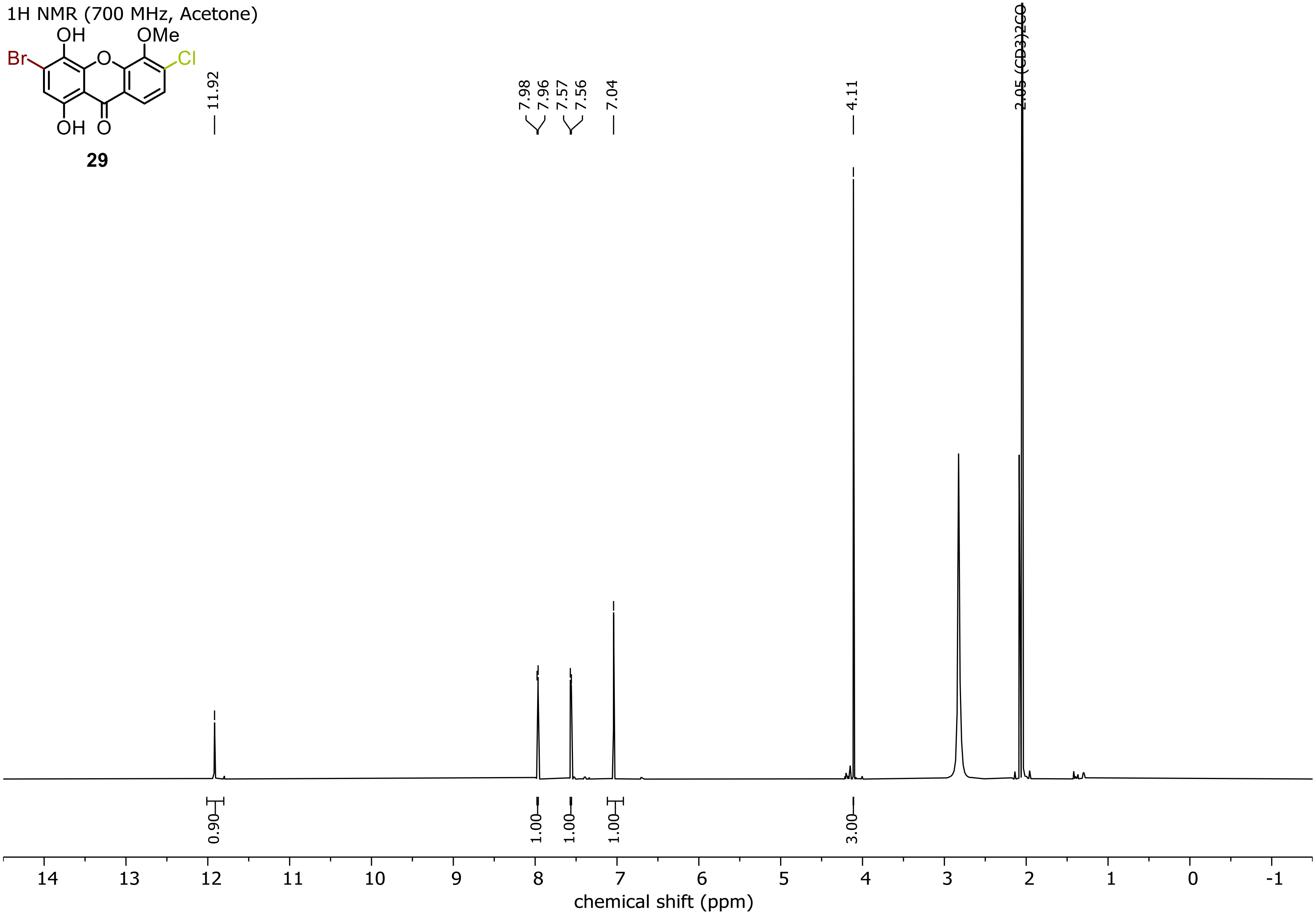

1H NMR (700 MHz, Acetone)
OH
OMe
Br
O
Cl
OH
O
11.92
7.98
7.96
7.57
7.56
7.04
4.11
2.05 (CD3)2CO
0.90
1.00
1.00
1.00
3.00
14 13 12 11 10 9 8 7 6 5 4 3 2 1 0 -1
chemical shift (ppm)

29

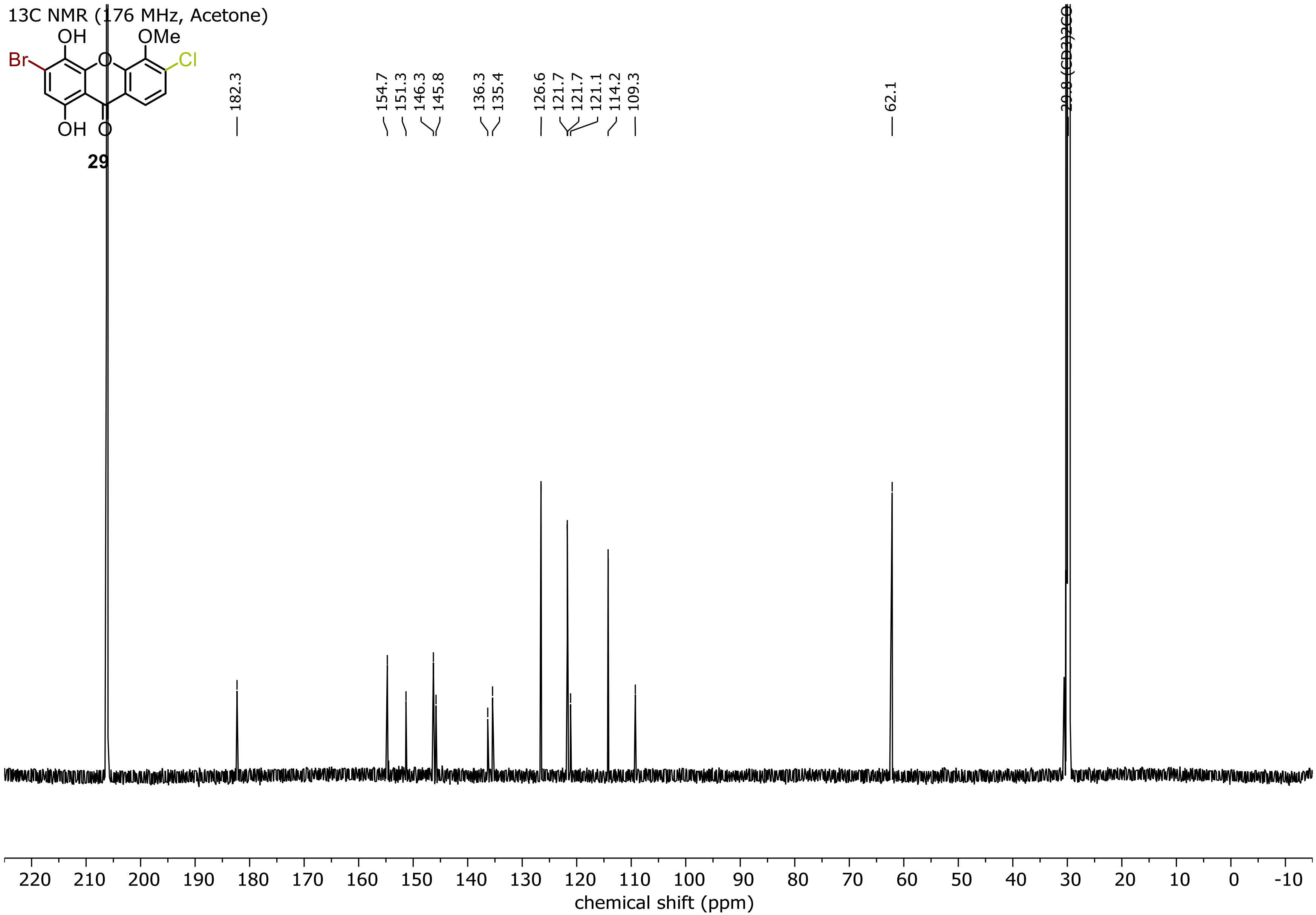
13C NMR (176 MHz, Acetone)
OH
OMe
Br
O
Cl
OH
O
29
182.3
154.7
151.3
146.3
145.8
136.3
135.4
126.6
121.7
121.7
121.1
114.2
109.3
62.1
29.8 (CD3)2CO
220 210 200 190 180 170 160 150 140 130 120 110 100 90 80 70 60 50 40 30 20 10 0 -10
chemical shift (ppm)

1H NMR (500 MHz, Acetone)

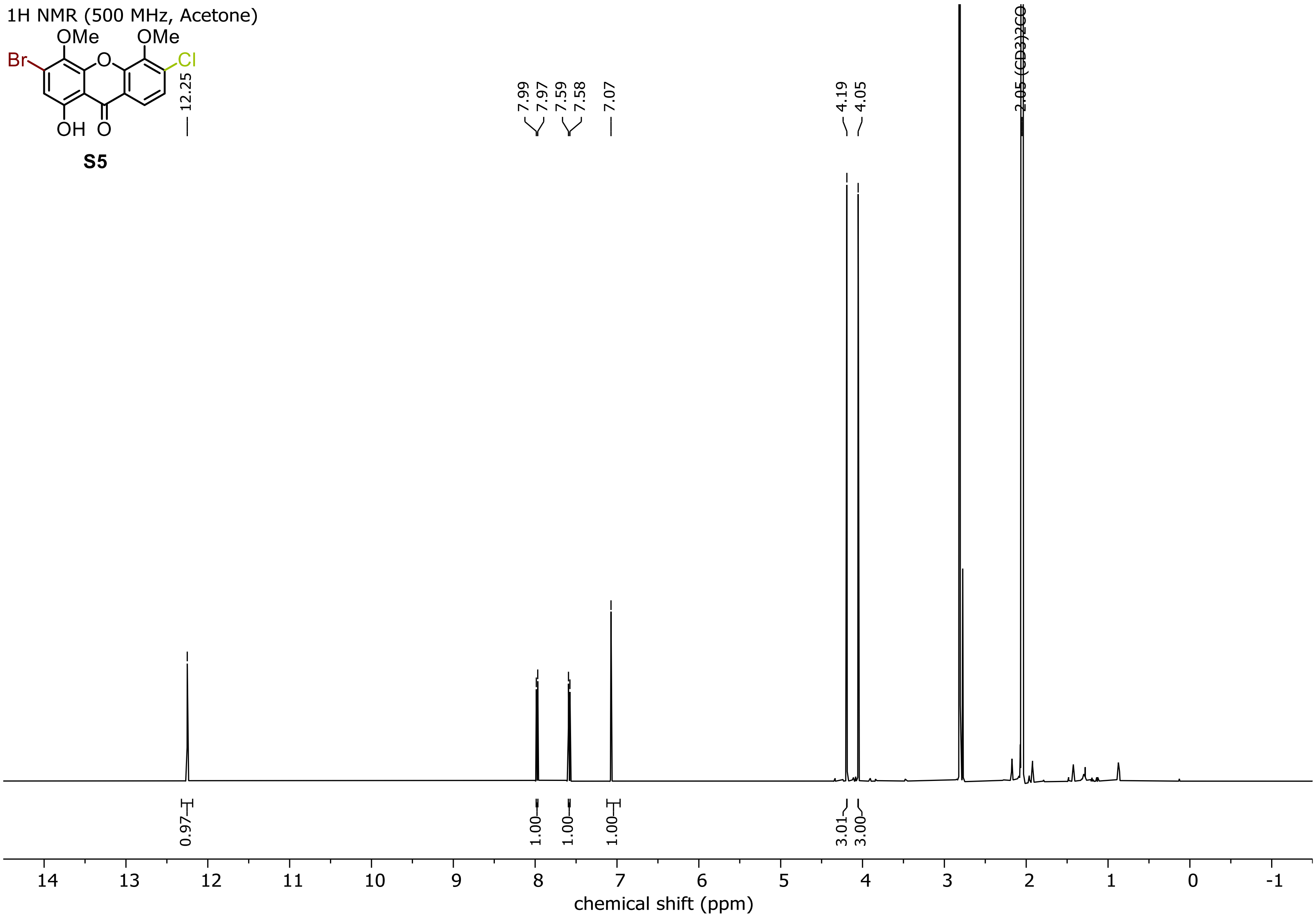

S5

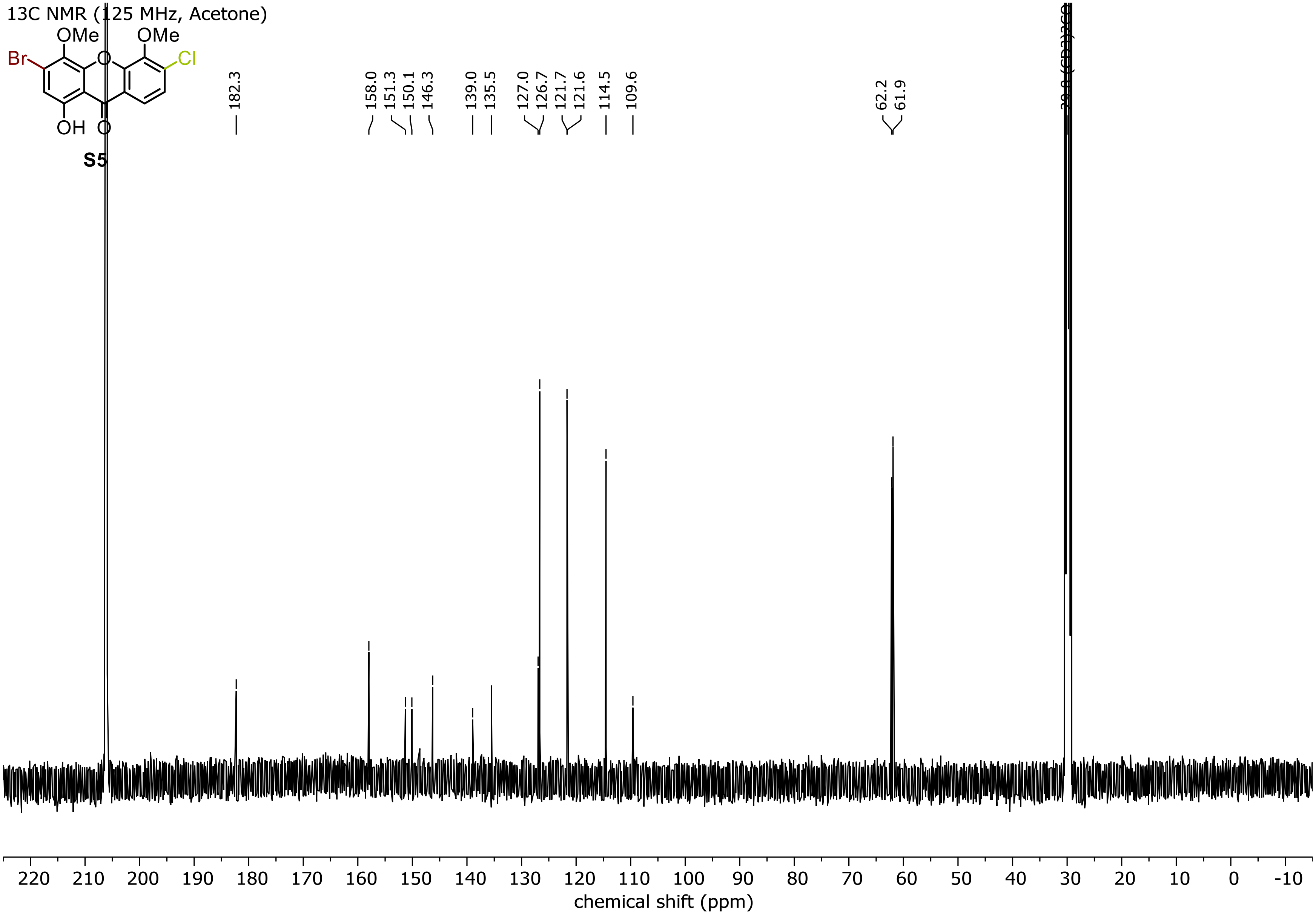
13C NMR (125 MHz, Acetone)
OMe
OMe
Br
O
Cl
OH
O
S5
182.3
158.0
151.3
150.1
146.3
139.0
135.5
127.0
126.7
121.7
121.6
114.5
109.6
62.2
61.9
220 210 200 190 180 170 160 150 140 130 120 110 100 90 80 70 60 50 40 30 20 10 0 -10
chemical shift (ppm)

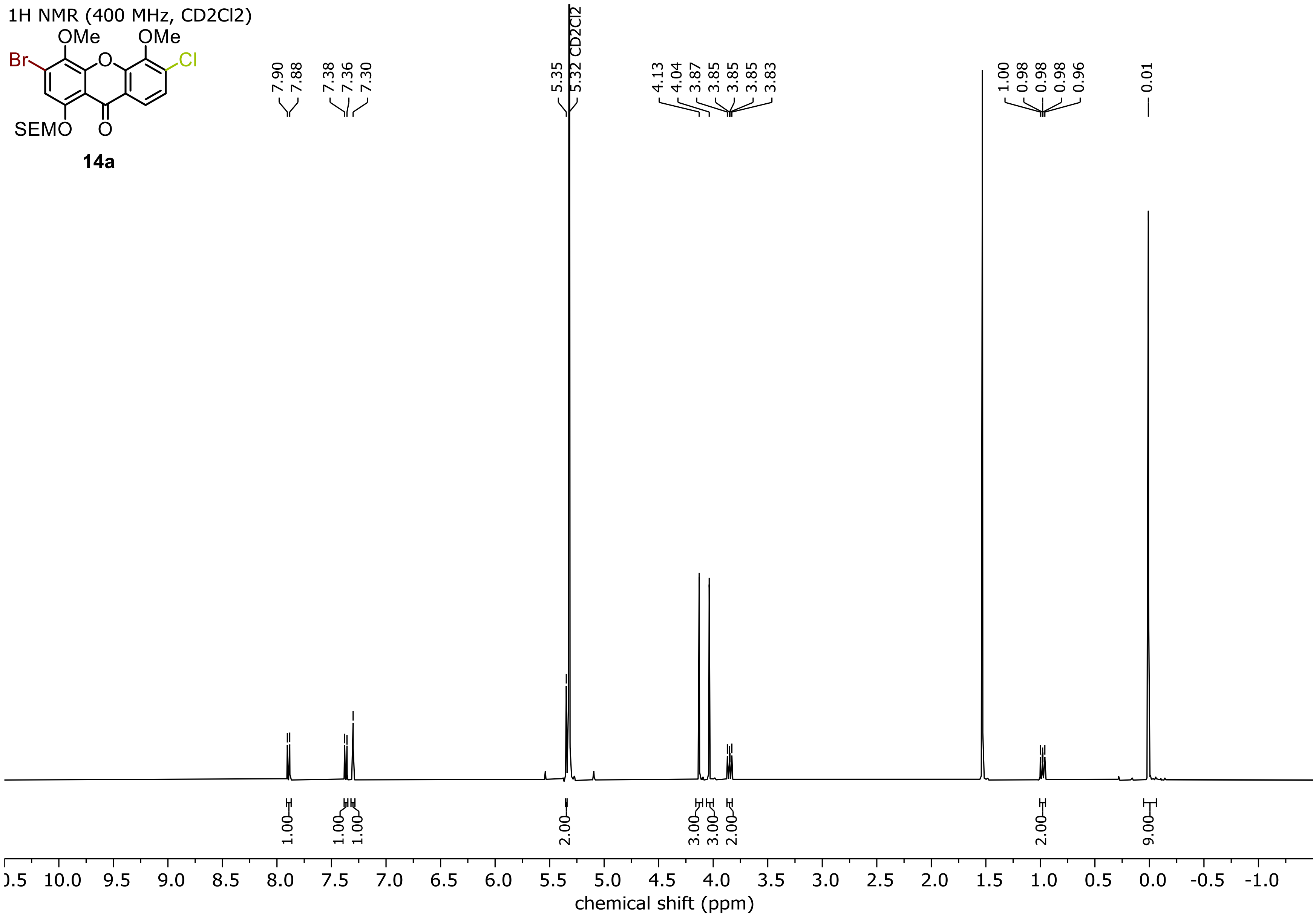
1H NMR (400 MHz, CD2Cl2)
OMe
OMe
Br
Cl
SEMO
O
14a
7.90
7.88
7.38
7.36
7.30
5.35
5.32 CD2Cl2
4.13
4.04
3.87
3.85
3.85
3.85
3.83
1.00
0.98
0.98
0.98
0.96
0.01
1.00
1.00
1.00
2.00
3.00
3.00
2.00
2.00
9.00
chemical shift (ppm)

13C NMR (176 MHz, CD2Cl2)

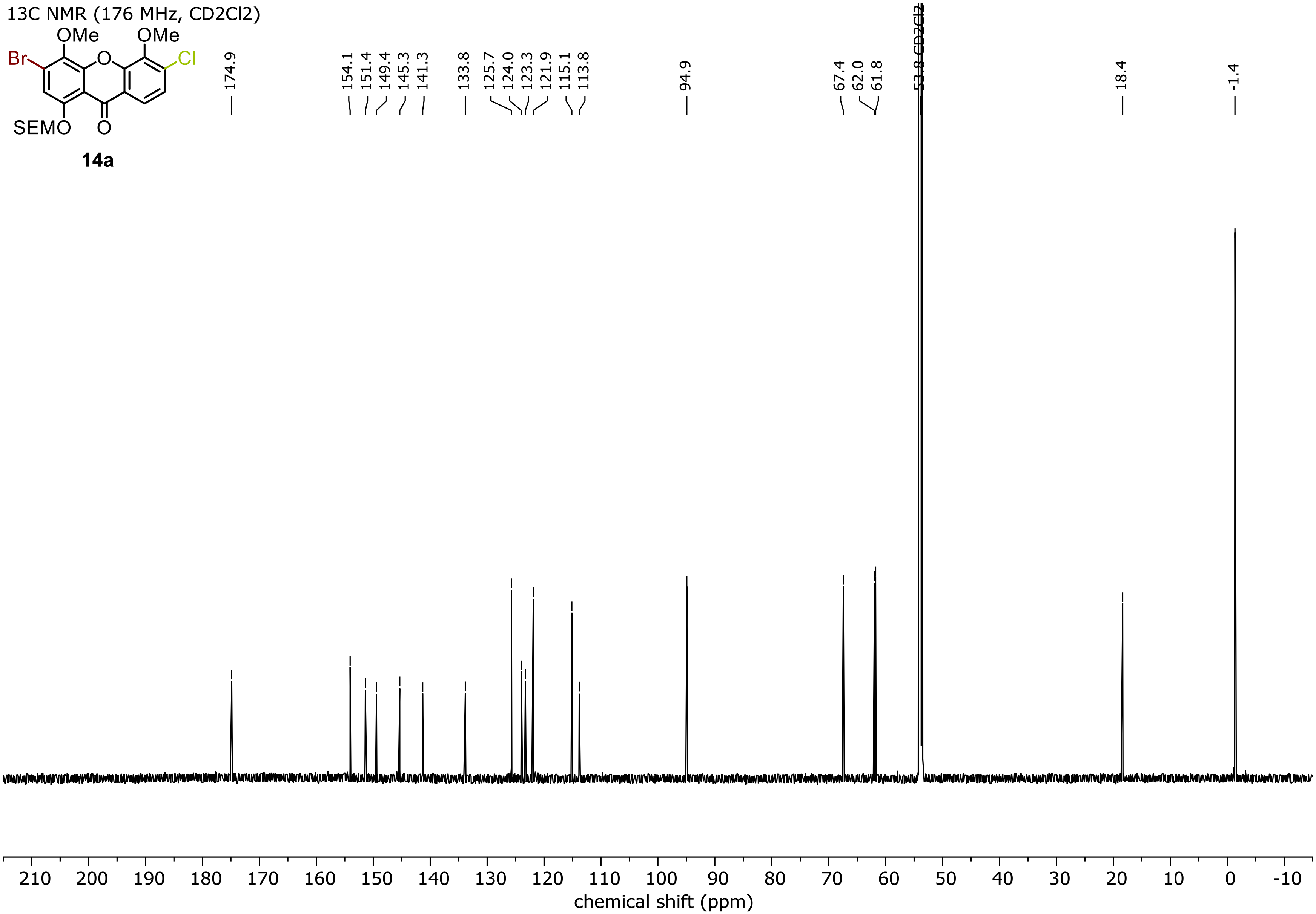

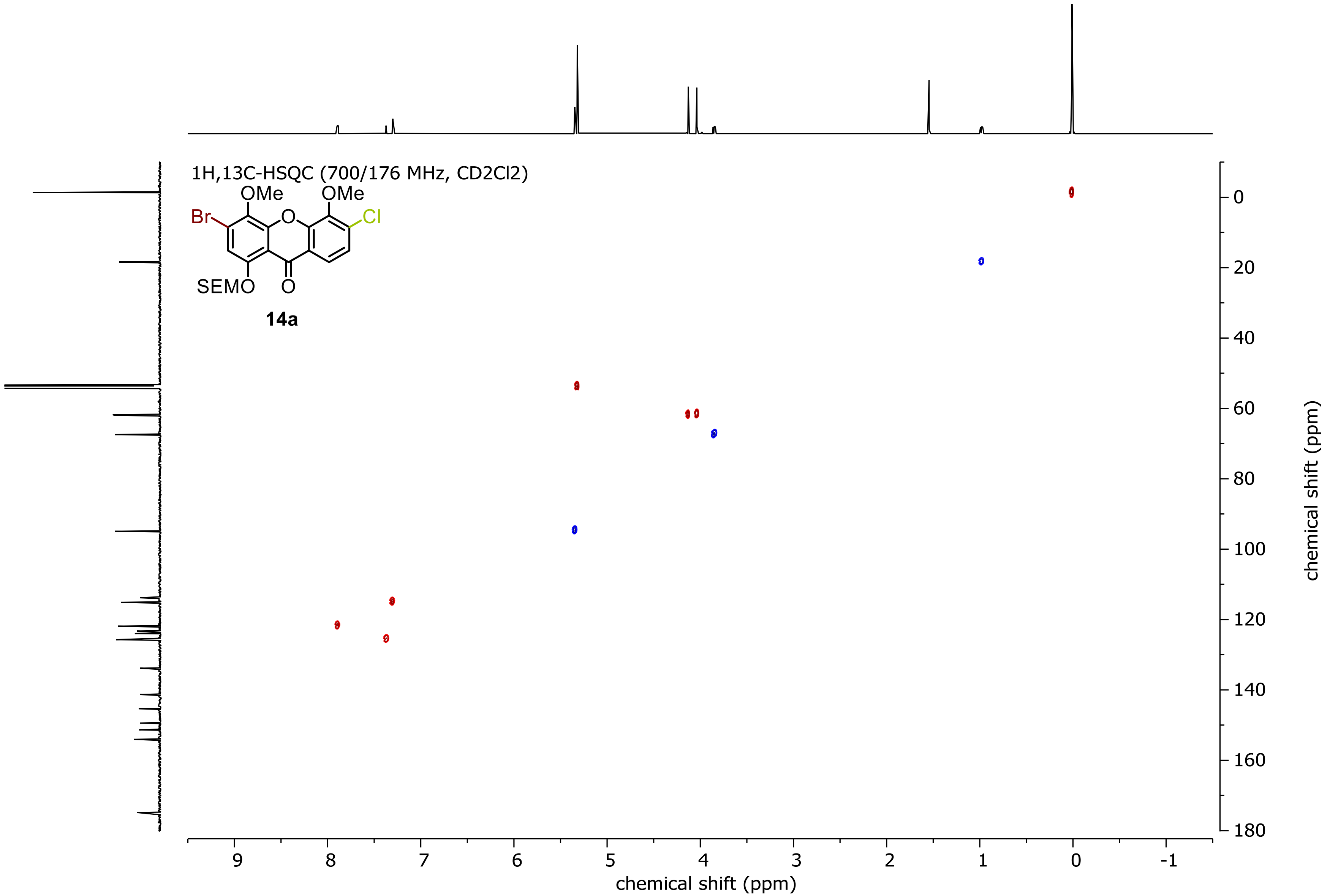
1H,13C-HSQC (700/176 MHz, CD2Cl2)
OMe
OMe
Br
Cl
SEMO
O
O
14a
chemical shift (ppm)
chemical shift (ppm)
9
8
7
6
5
4
3
2
1
0
-1
0
20
40
60
80
100
120
140
160
180

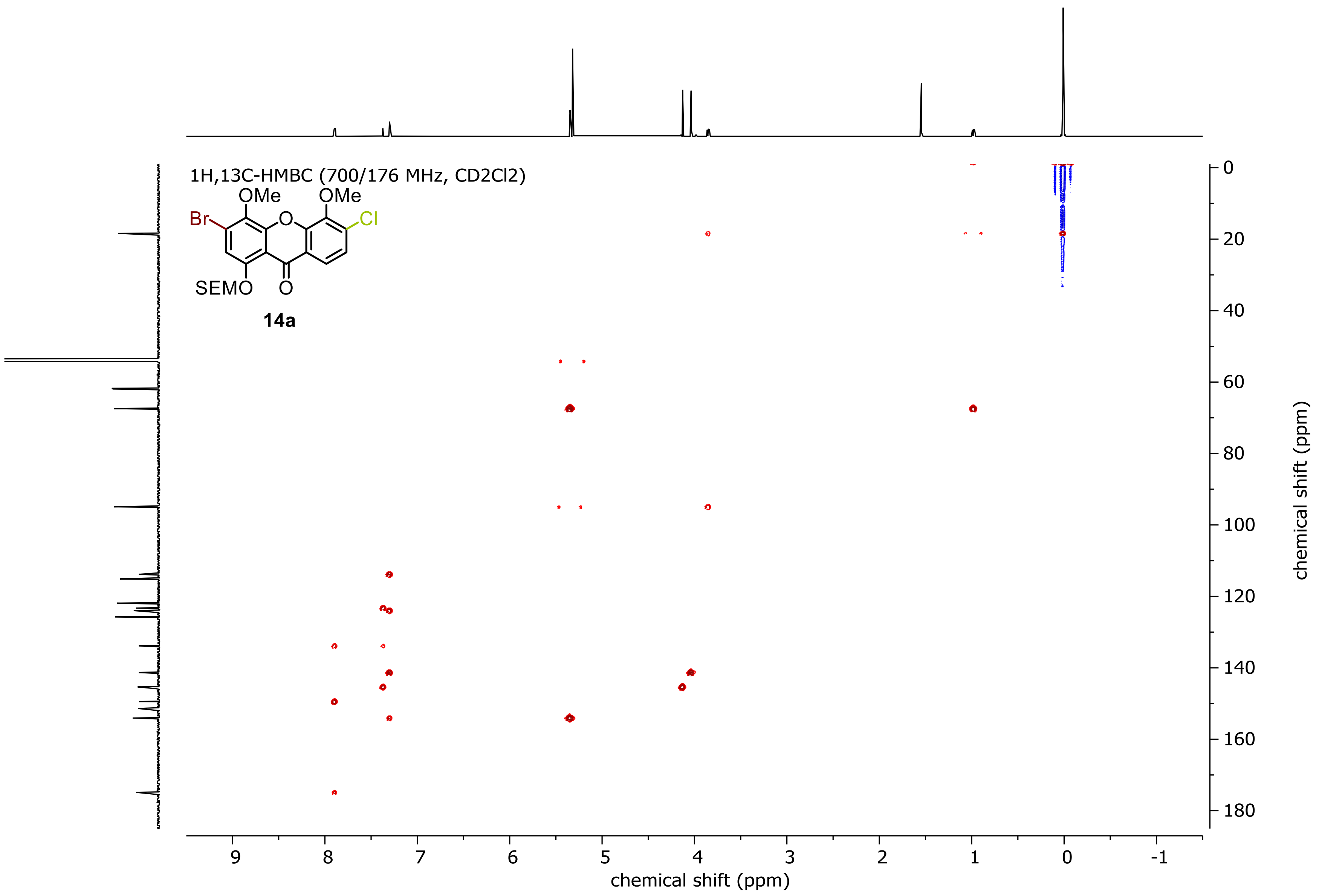
1H,13C-HMBC (700/176 MHz, CD2Cl2)
OMe
OMe
Br
Cl
SEMO
O
O
14a
chemical shift (ppm)
chemical shift (ppm)

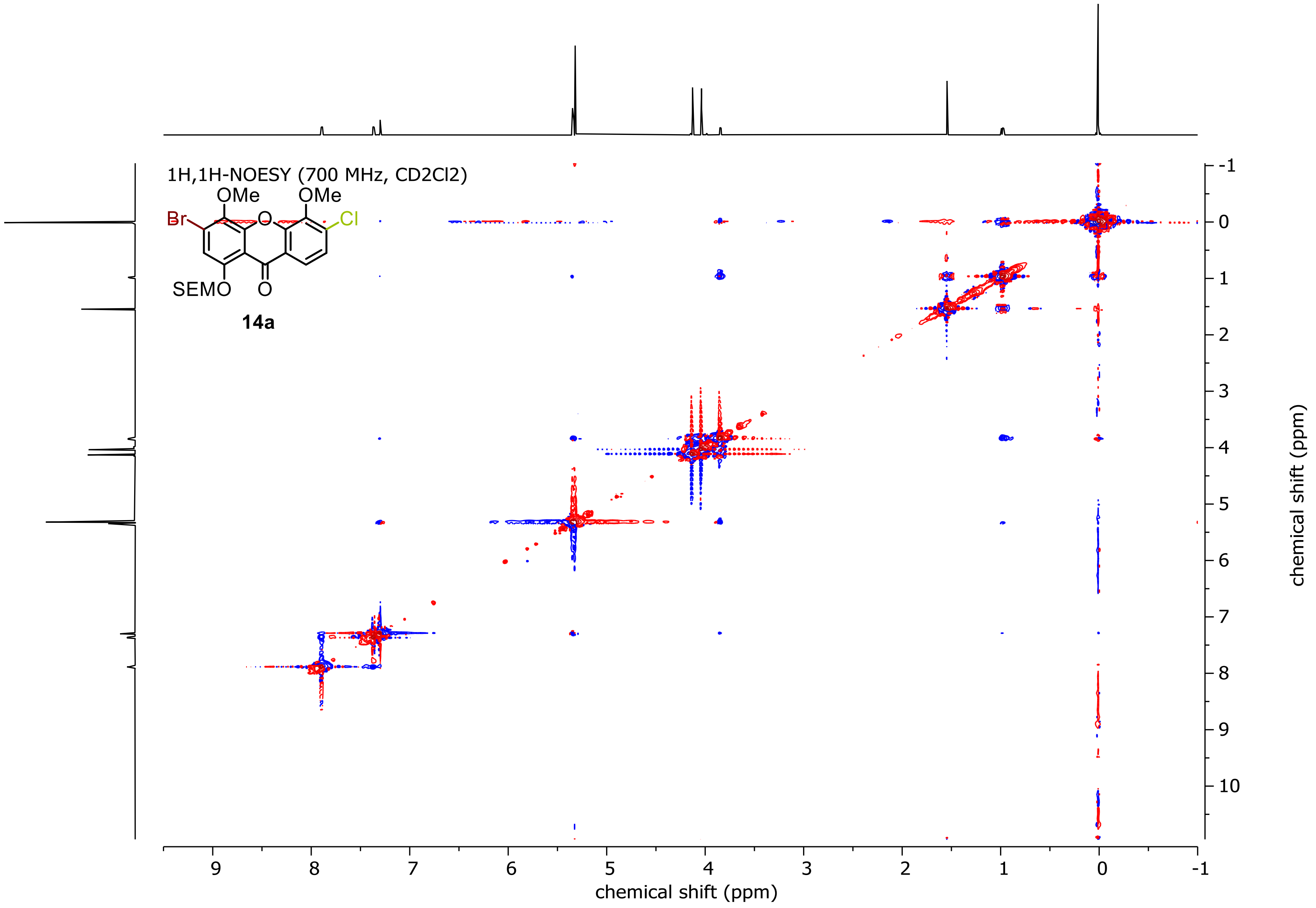
1H,1H-NOESY (700 MHz, CD2Cl2)
OMe
OMe
Br
O
Cl
SEMO
O
14a
chemical shift (ppm)
chemical shift (ppm)

7.3 Leading to CBS100 (**5**)

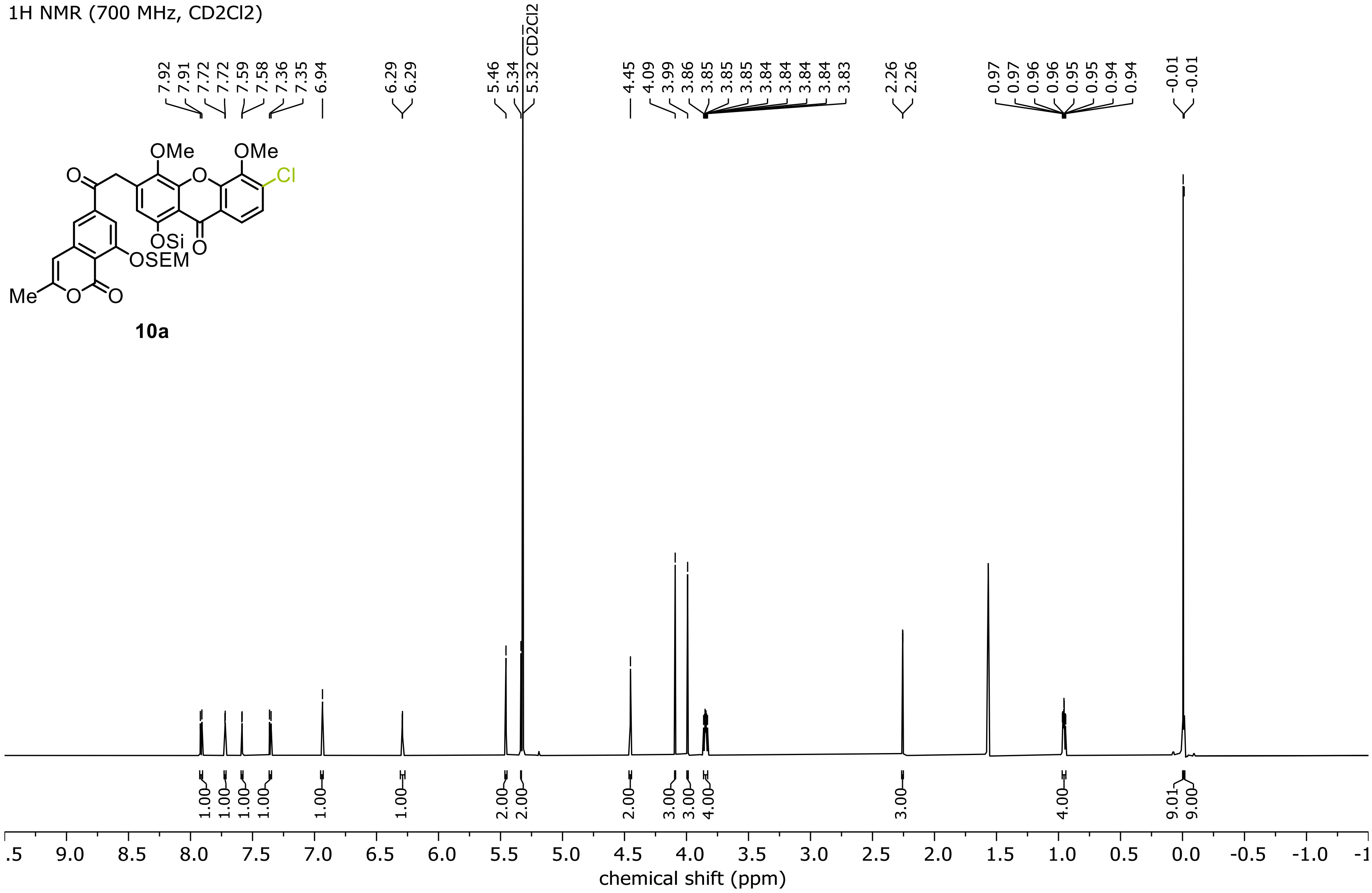

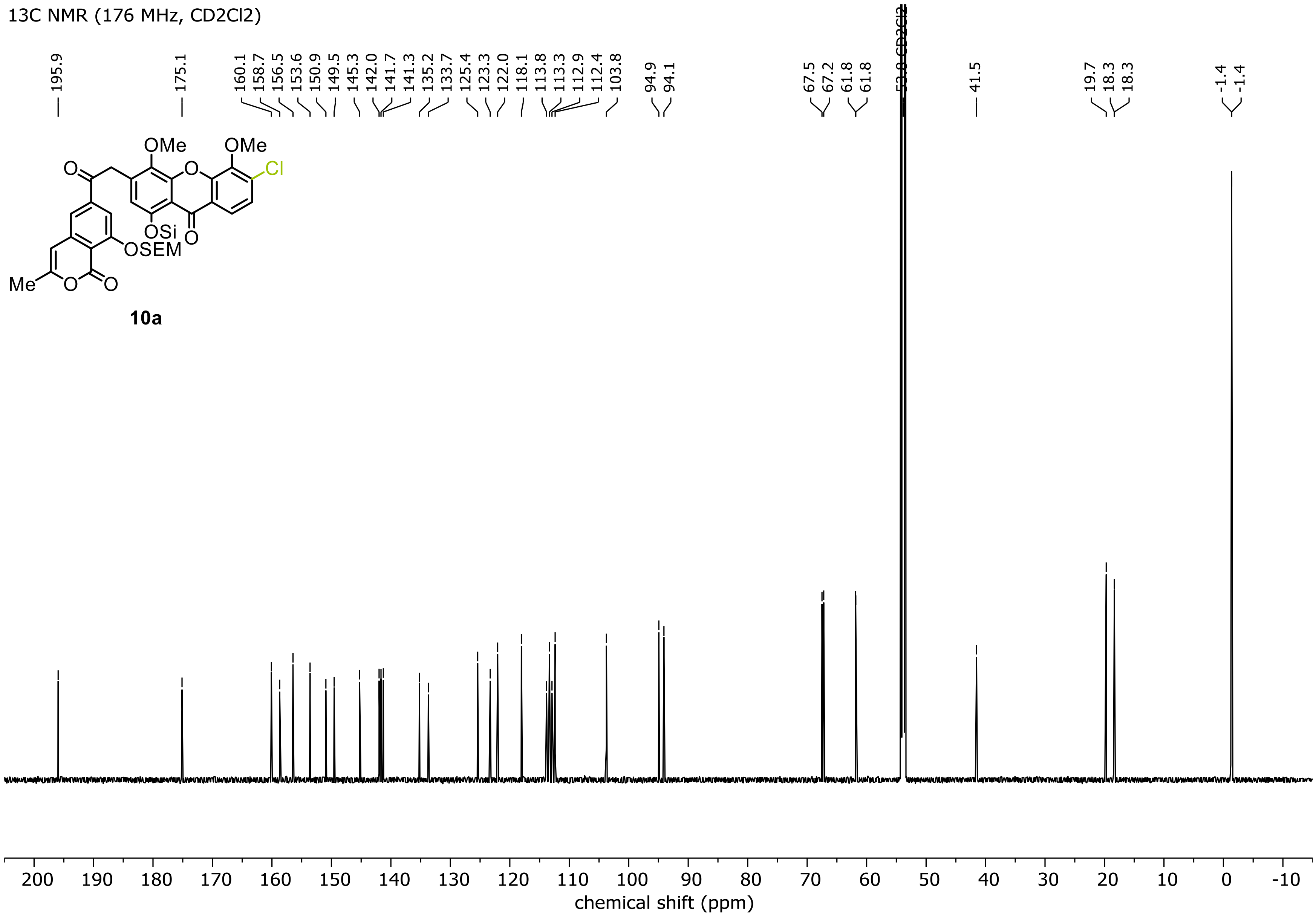
13C NMR (176 MHz, CD2Cl2)
195.9
175.1
160.1
158.7
156.5
153.6
150.9
149.5
145.3
142.0
141.7
141.3
135.2
133.7
125.4
123.3
122.0
118.1
113.8
113.3
112.9
112.4
103.8
94.9
94.1
67.5
67.2
61.8
61.8
CD2Cl2
41.5
19.7
18.3
18.3
-1.4
-1.4
OMe
OMe
Cl
OSi
OSEM
Me
10a
200 190 180 170 160 150 140 130 120 110 100 90 80 70 60 50 40 30 20 10 0 -10
chemical shift (ppm)

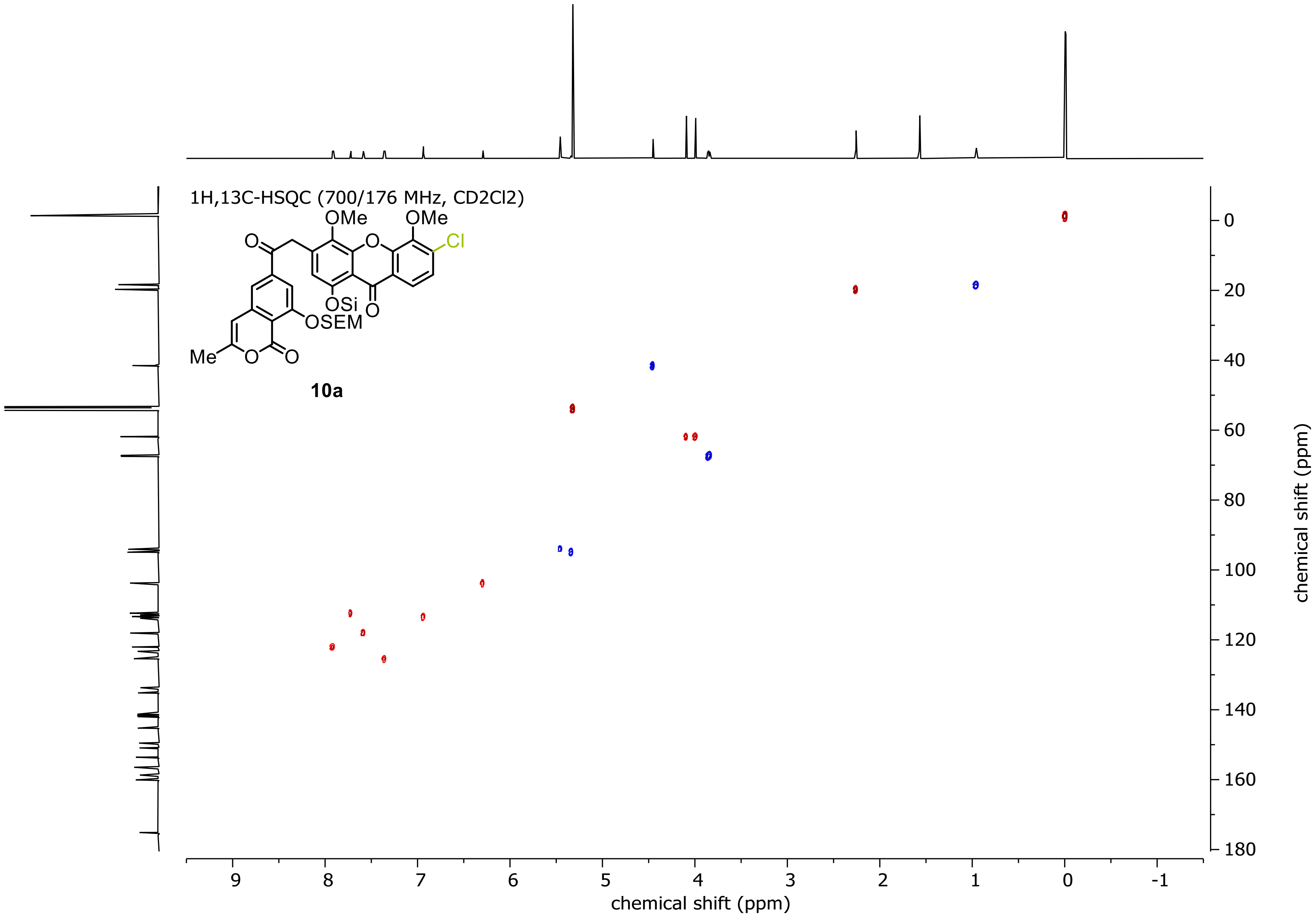
1H,13C-HSQC (700/176 MHz, CD2Cl2)
OMe
OMe
Cl
O
O
OSi
O
OSEM
Me
O
O
10a
9
8
7
6
5
4
3
2
1
0
-1
chemical shift (ppm)
0
20
40
60
80
100
120
140
160
180
chemical shift (ppm)

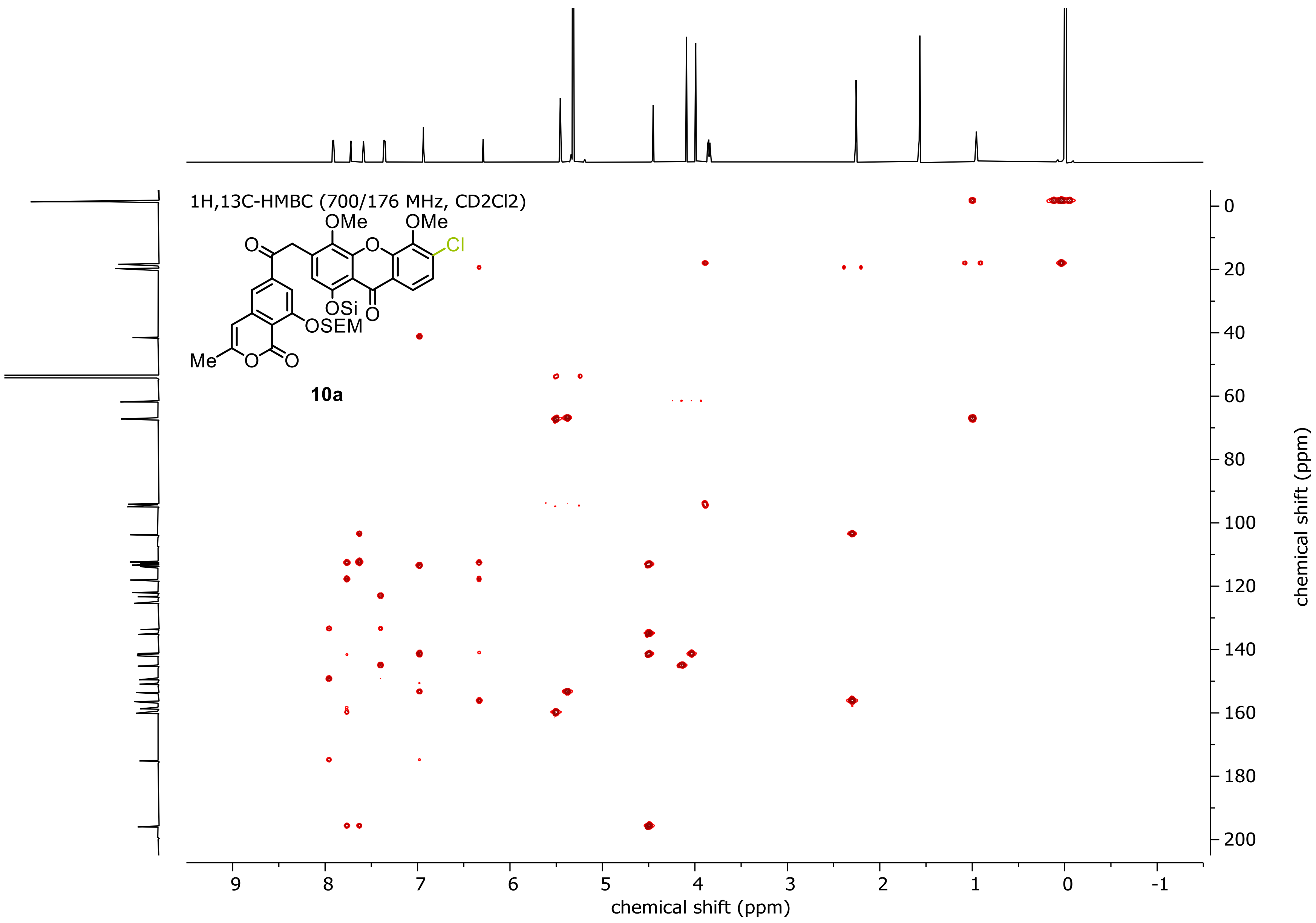
1H,13C-HMBC (700/176 MHz, CD2Cl2)
OMe
OMe
Cl
O
OSi
OSEM
Me
10a
chemical shift (ppm)
chemical shift (ppm)
9
8
7
6
5
4
3
2
1
0
-1
0
20
40
60
80
100
120
140
160
180
200

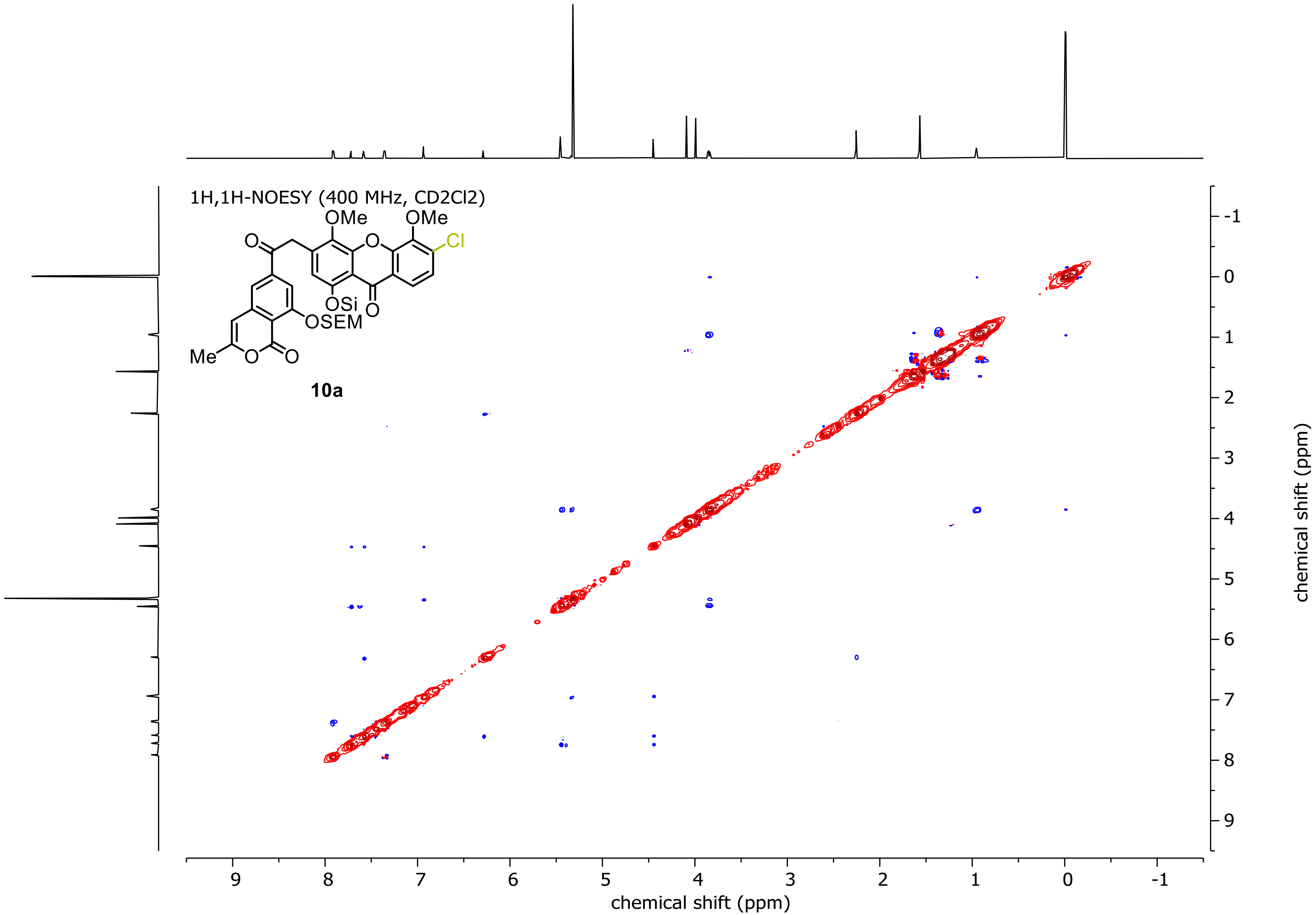
1H,1H-NOESY (400 MHz, CD2Cl2)
OMe
OMe
Cl
OSi
OSEM
Me
10a
chemical shift (ppm)
chemical shift (ppm)

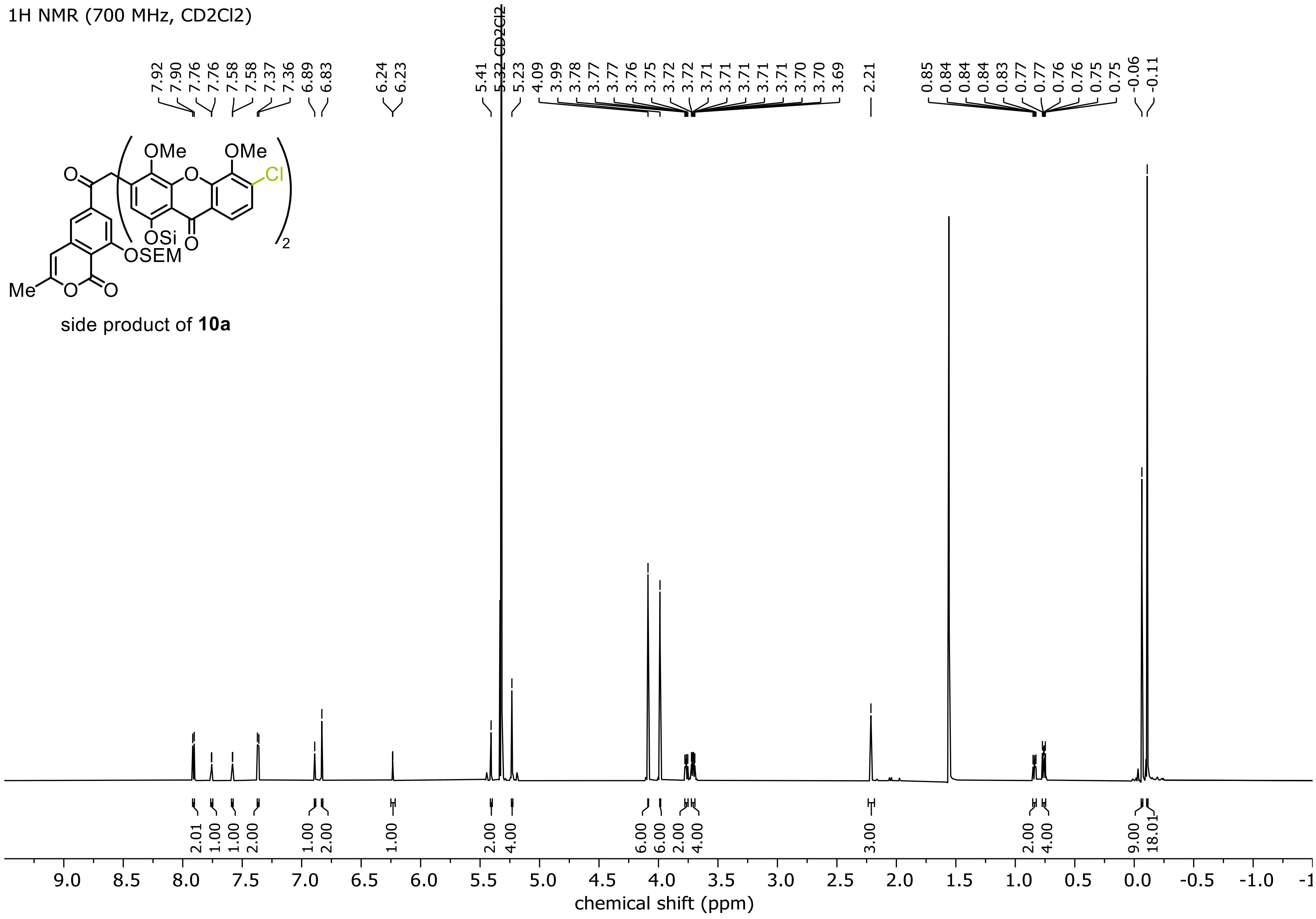

1H NMR (700 MHz, CD2Cl2)
OMe
OMe
O
O
Cl
OSi
O
OSEM
2
Me
O
O
side product of **10a**
7.92
7.90
7.76
7.76
7.58
7.58
7.37
7.36
6.89
6.83
6.24
6.23
5.41
5.32 CD2Cl2
5.23
4.09
3.99
3.78
3.77
3.77
3.76
3.75
3.72
3.72
3.71
3.71
3.71
3.71
3.71
3.70
3.70
3.69
2.21
0.85
0.84
0.84
0.84
0.83
0.77
0.77
0.76
0.76
0.75
0.75
-0.06
-0.11
2.01
1.00
1.00
2.00
1.00
2.00
1.00
2.00
4.00
6.00
6.00
2.00
4.00
3.00
2.00
4.00
9.00
18.01
9.0
8.5
8.0
7.5
7.0
6.5
6.0
5.5
5.0
4.5
4.0
3.5
3.0
2.5
2.0
1.5
1.0
0.5
0.0
-0.5
-1.0
chemical shift (ppm)

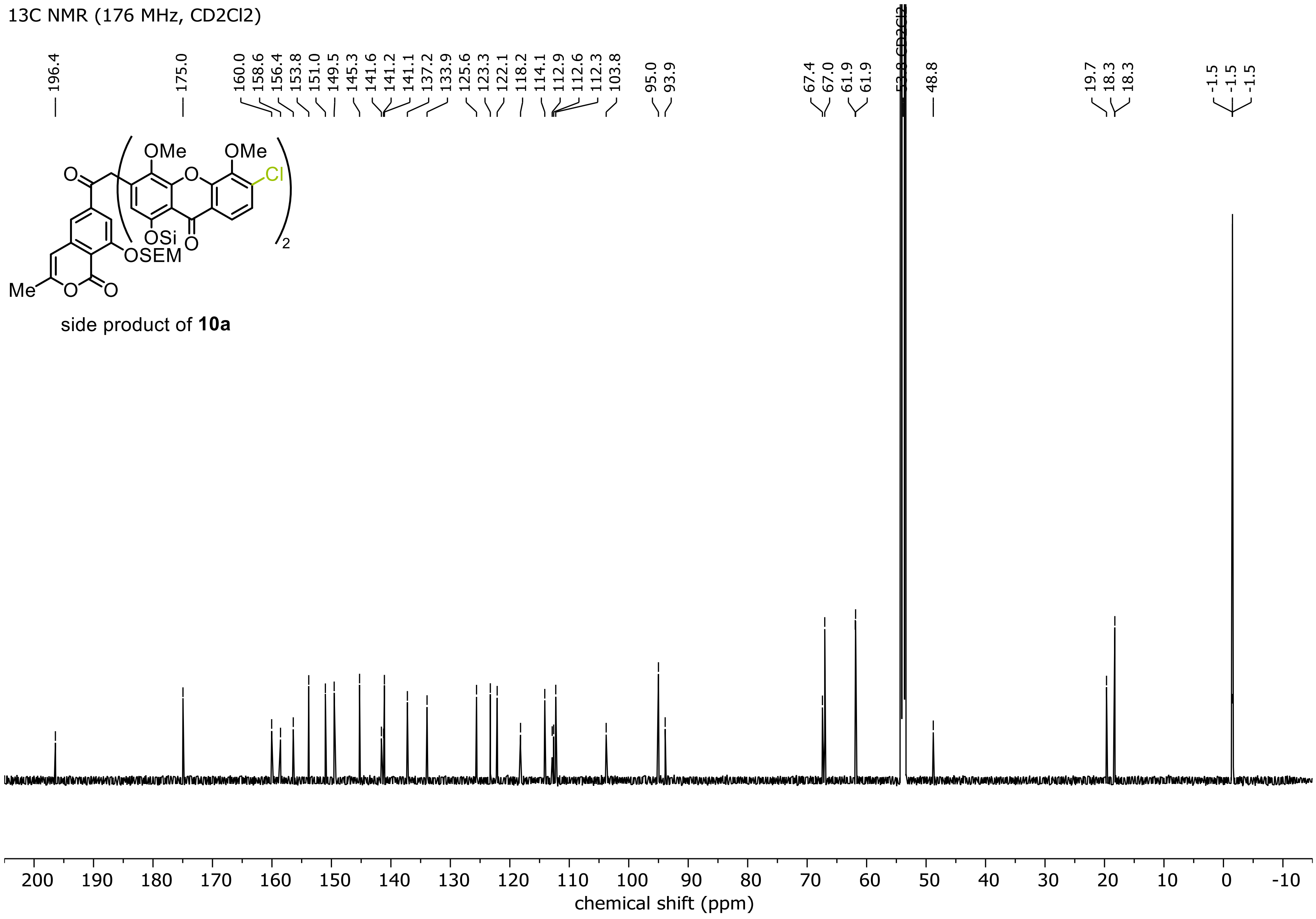
13C NMR (176 MHz, CD2Cl2)
196.4
175.0
160.0
158.6
156.4
153.8
151.0
149.5
145.3
141.6
141.2
141.1
137.2
133.9
125.6
123.3
122.1
118.2
114.1
112.9
112.6
112.3
103.8
95.0
93.9
67.4
67.0
61.9
61.9
CD2Cl2
48.8
19.7
18.3
18.3
-1.5
-1.5
-1.5
OMe
OMe
O
Cl
OSi
O
OSEM
2
Me
O
O
side product of **10a**
200 190 180 170 160 150 140 130 120 110 100 90 80 70 60 50 40 30 20 10 0 -10
chemical shift (ppm)

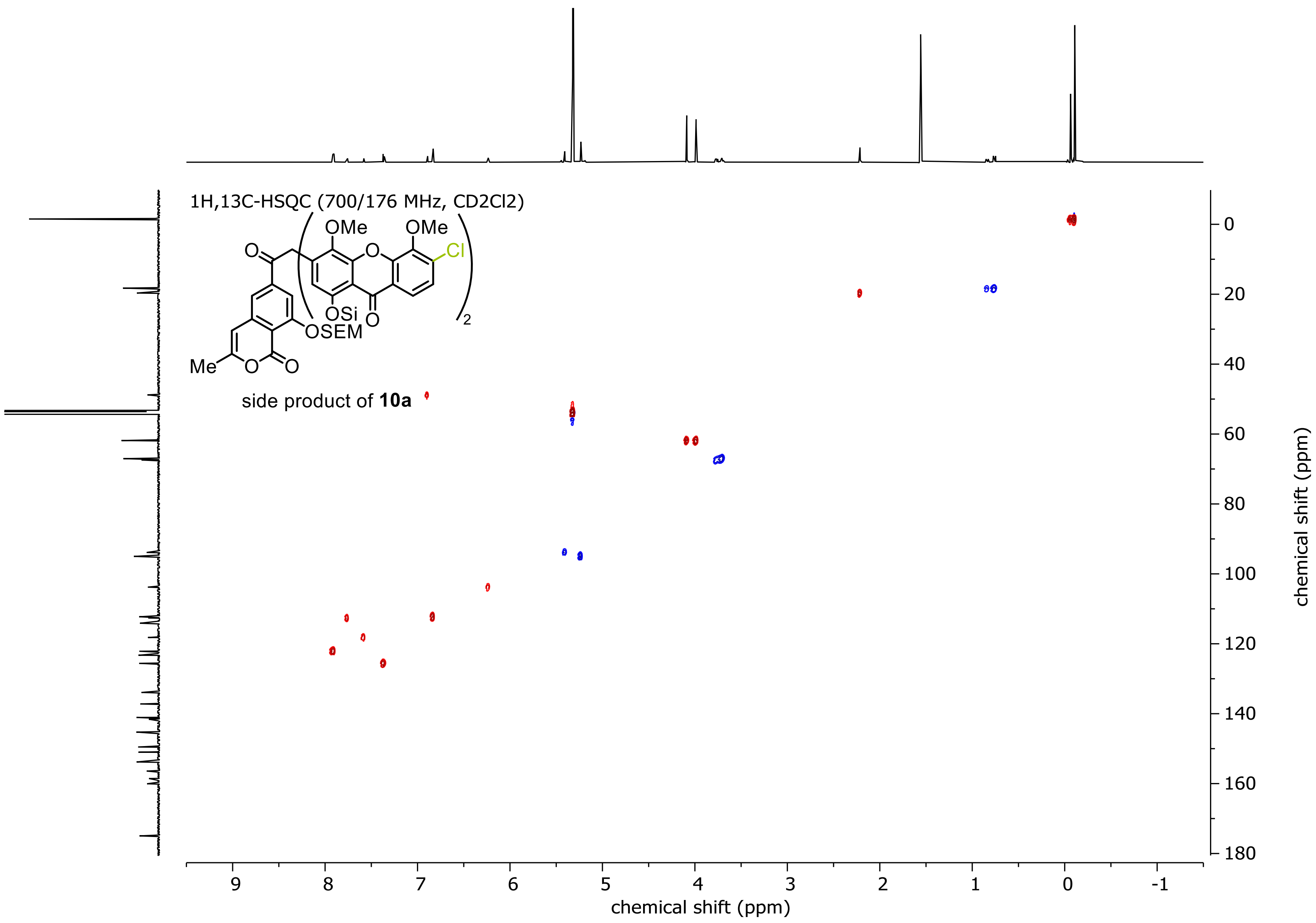
1H,13C-HSQC (700/176 MHz, CD2Cl2)
OMe
OMe
Cl
O
OSi
O
OSEM
Me
2
side product of **10a**
chemical shift (ppm)
chemical shift (ppm)

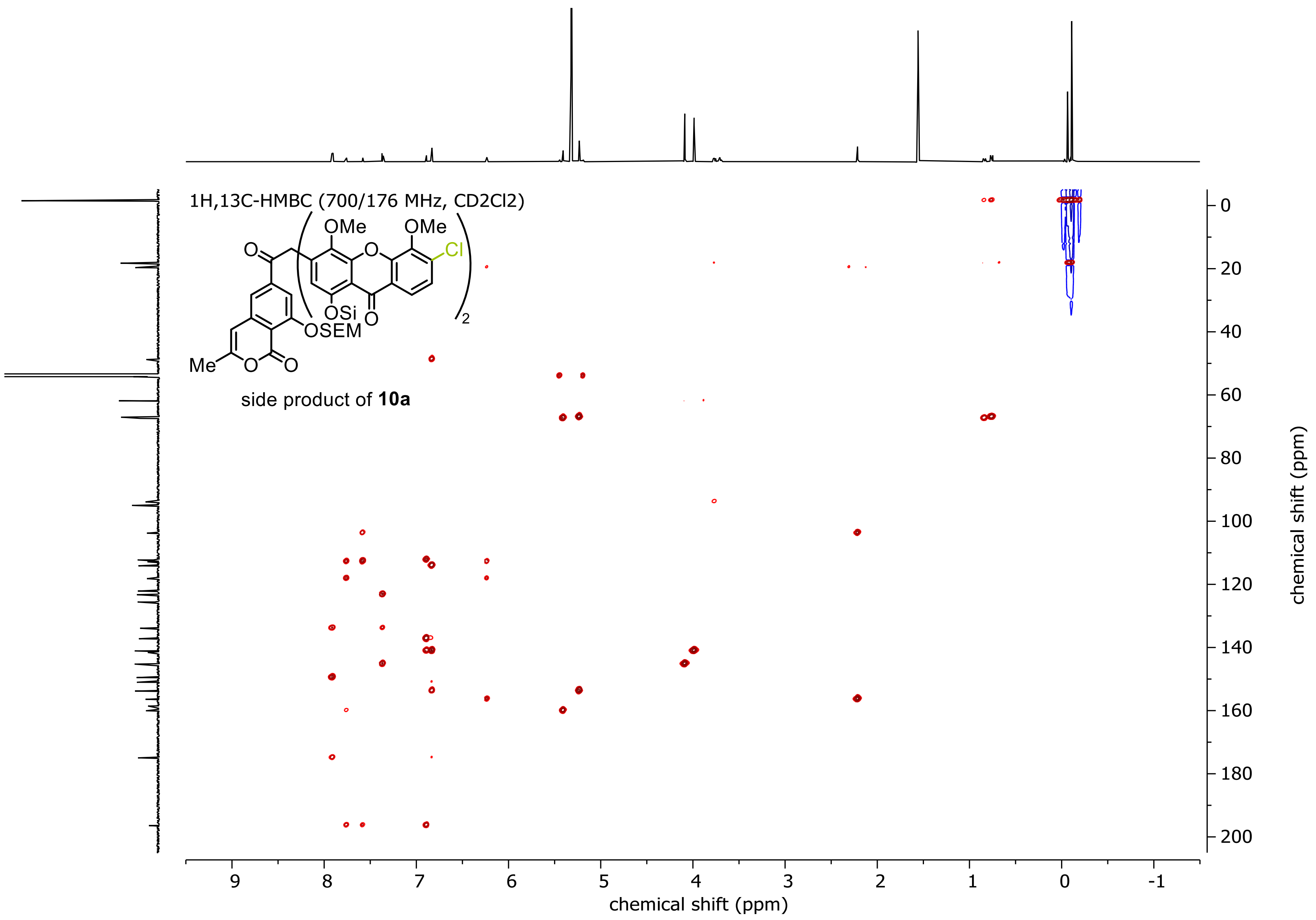
1H,13C-HMBC (700/176 MHz, CD2Cl2)
OMe
OMe
Cl
O
O
OSi
O
OSEM
2
Me
O
O
side product of **10a**
chemical shift (ppm)
chemical shift (ppm)
9
8
7
6
5
4
3
2
1
0
-1
0
20
40
60
80
100
120
140
160
180
200

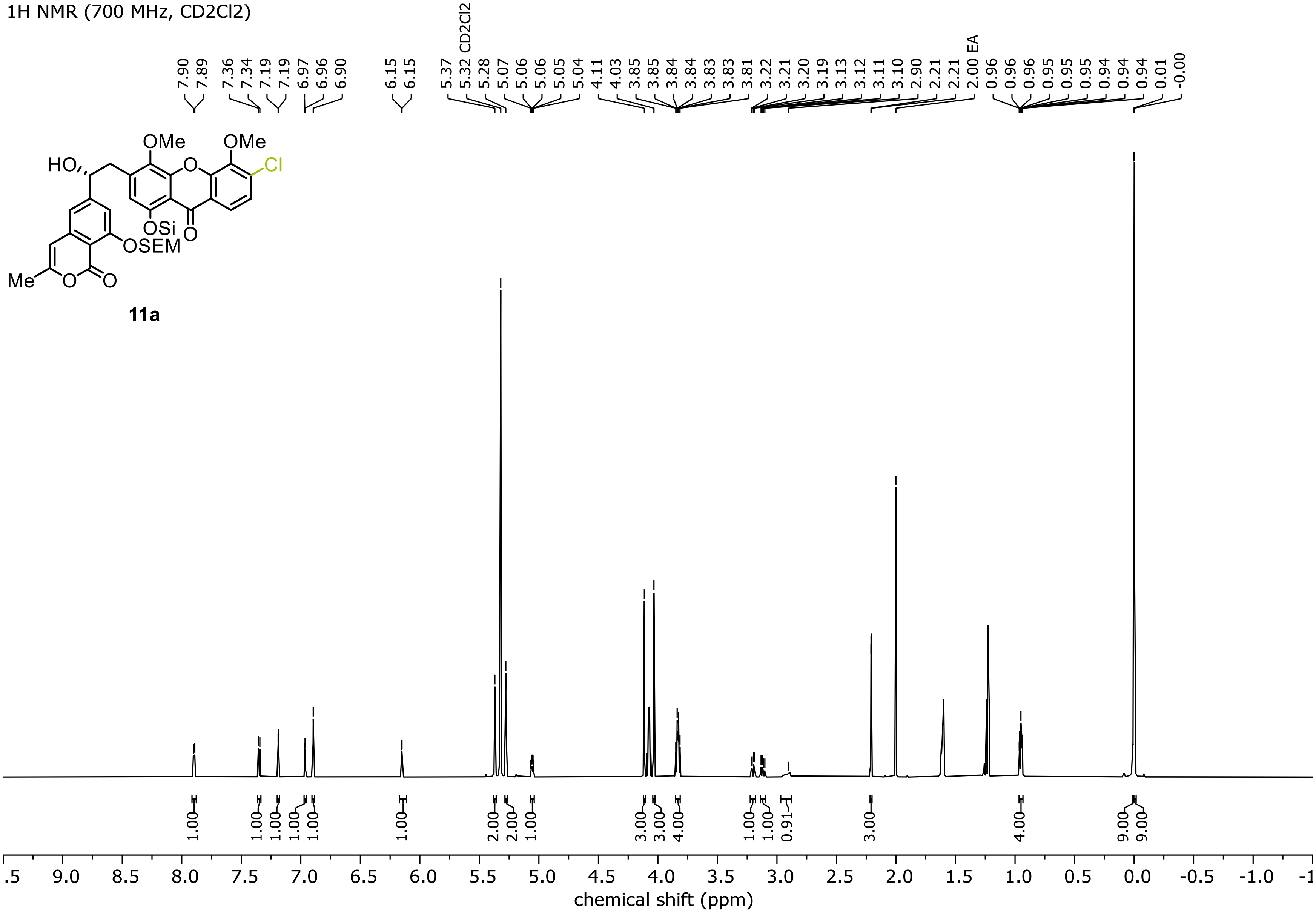
1H NMR (700 MHz, CD2Cl2)
CD2Cl2
EA
OMe
OMe
HO
Cl
O
OSi
O
OSEM
Me
O
O
11a
chemical shift (ppm)

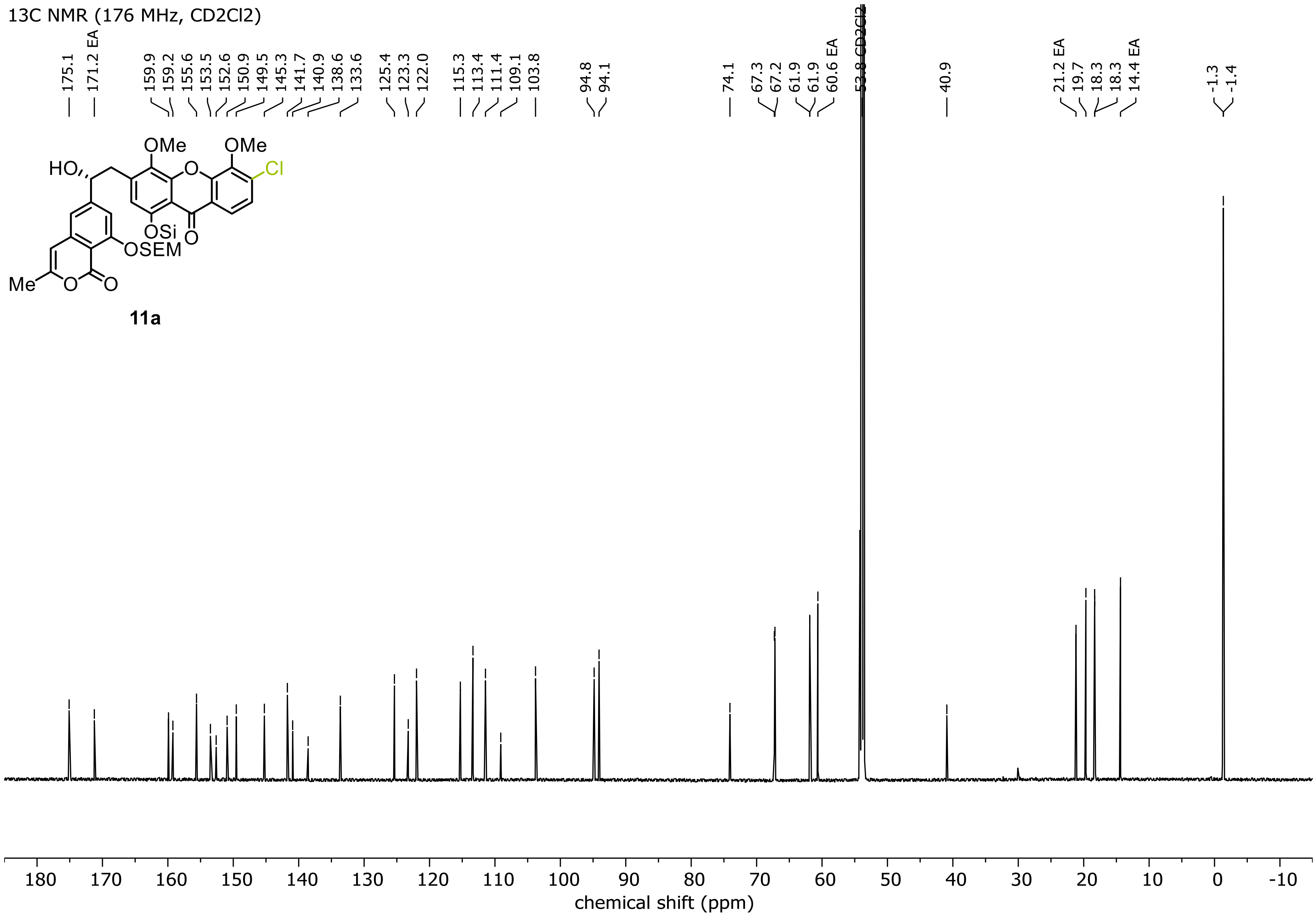
13C NMR (176 MHz, CD2Cl2)
175.1
171.2 EA
159.9
159.2
155.6
153.5
152.6
150.9
149.5
145.3
141.7
140.9
138.6
133.6
125.4
123.3
122.0
115.3
113.4
111.4
109.1
103.8
94.8
94.1
74.1
67.3
67.2
61.9
61.9
60.6 EA
53.8 CD2Cl2
40.9
21.2 EA
19.7
18.3
18.3
14.4 EA
-1.3
-1.4
OMe
OMe
HO
Cl
OSi
OSEM
Me
O
11a
chemical shift (ppm)

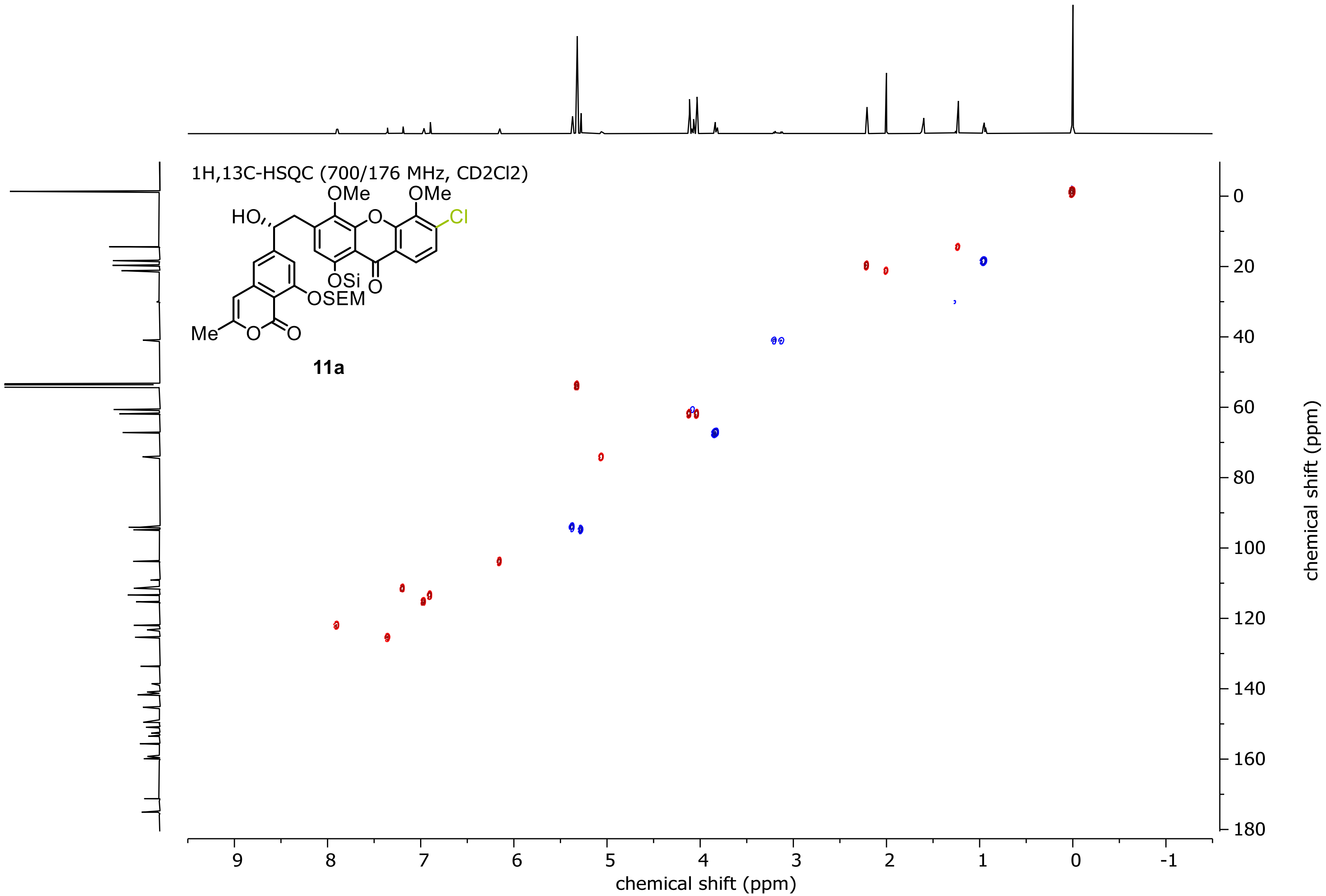

1H,13C-HSQC (700/176 MHz, CD2Cl2)
OMe
OMe
HO
O
Cl
OSi
O
OSEM
Me
O
O
11a
chemical shift (ppm)
chemical shift (ppm)

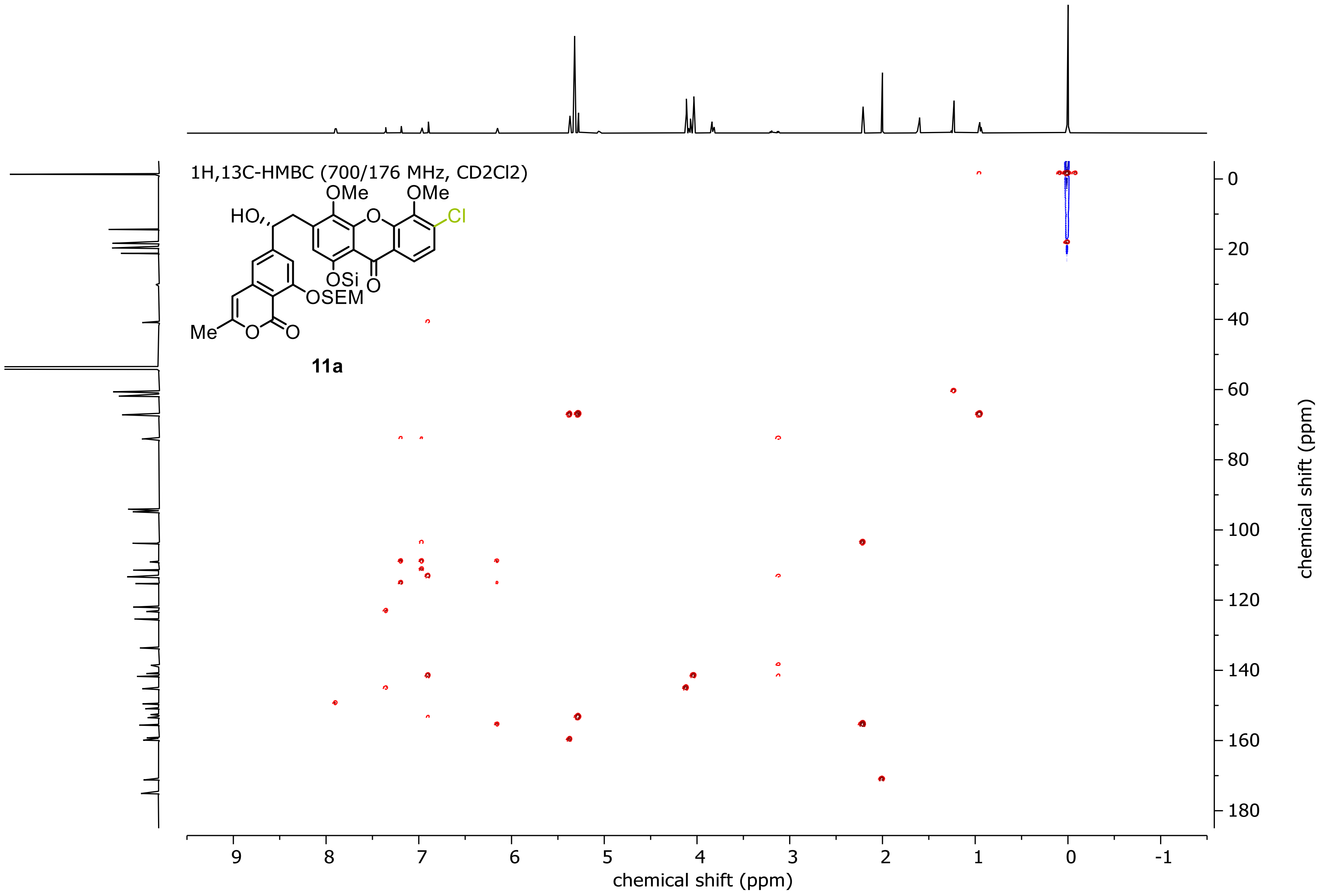
1H,13C-HMBC (700/176 MHz, CD2Cl2)
OMe
OMe
HO
O
Cl
OSi
O
OSEM
Me
O
O
11a
0
20
40
60
80
100
120
140
160
180
chemical shift (ppm)
9
8
7
6
5
4
3
2
1
0
-1
chemical shift (ppm)

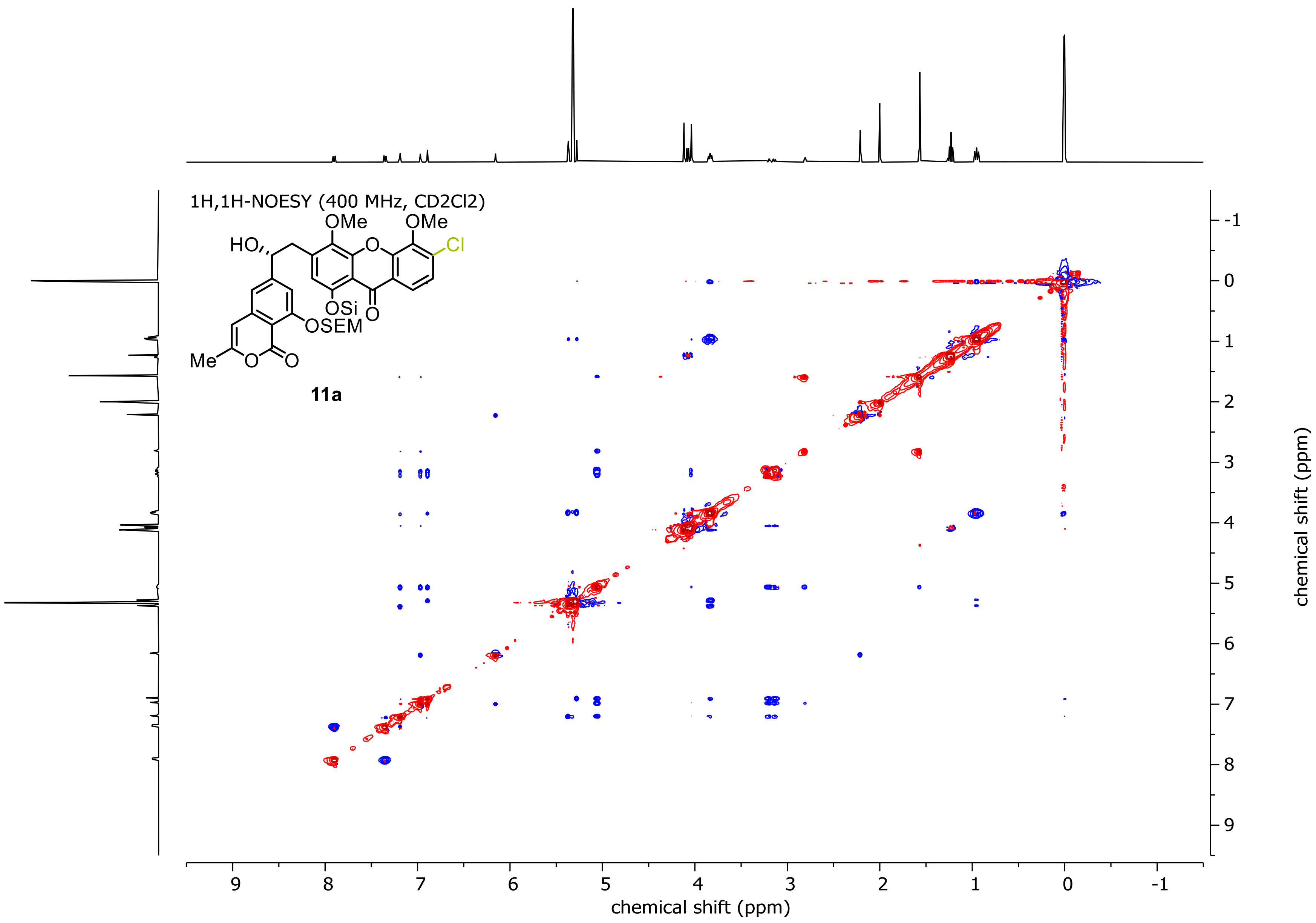

1H,1H-NOESY (400 MHz, CD2Cl2)
OMe
OMe
HO
Cl
O
OSi
O
OSEM
Me
O
O
11a
chemical shift (ppm)
chemical shift (ppm)

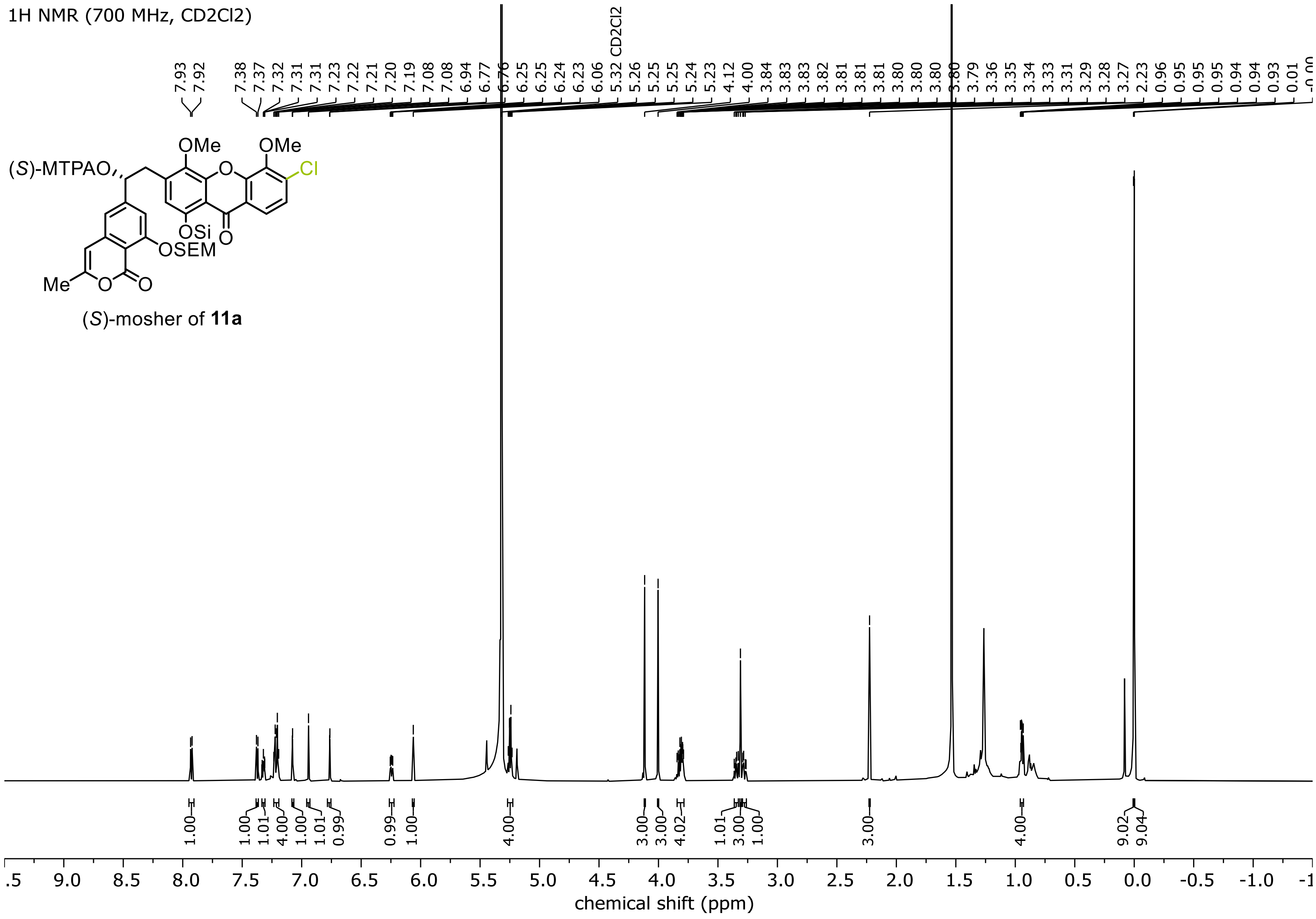

1H NMR (700 MHz, CD2Cl2)
CD2Cl2
(S)-MTPAO
OMe
OMe
Cl
OSi
OSEM
Me
O
O
(S)-mosher of 11a
chemical shift (ppm)

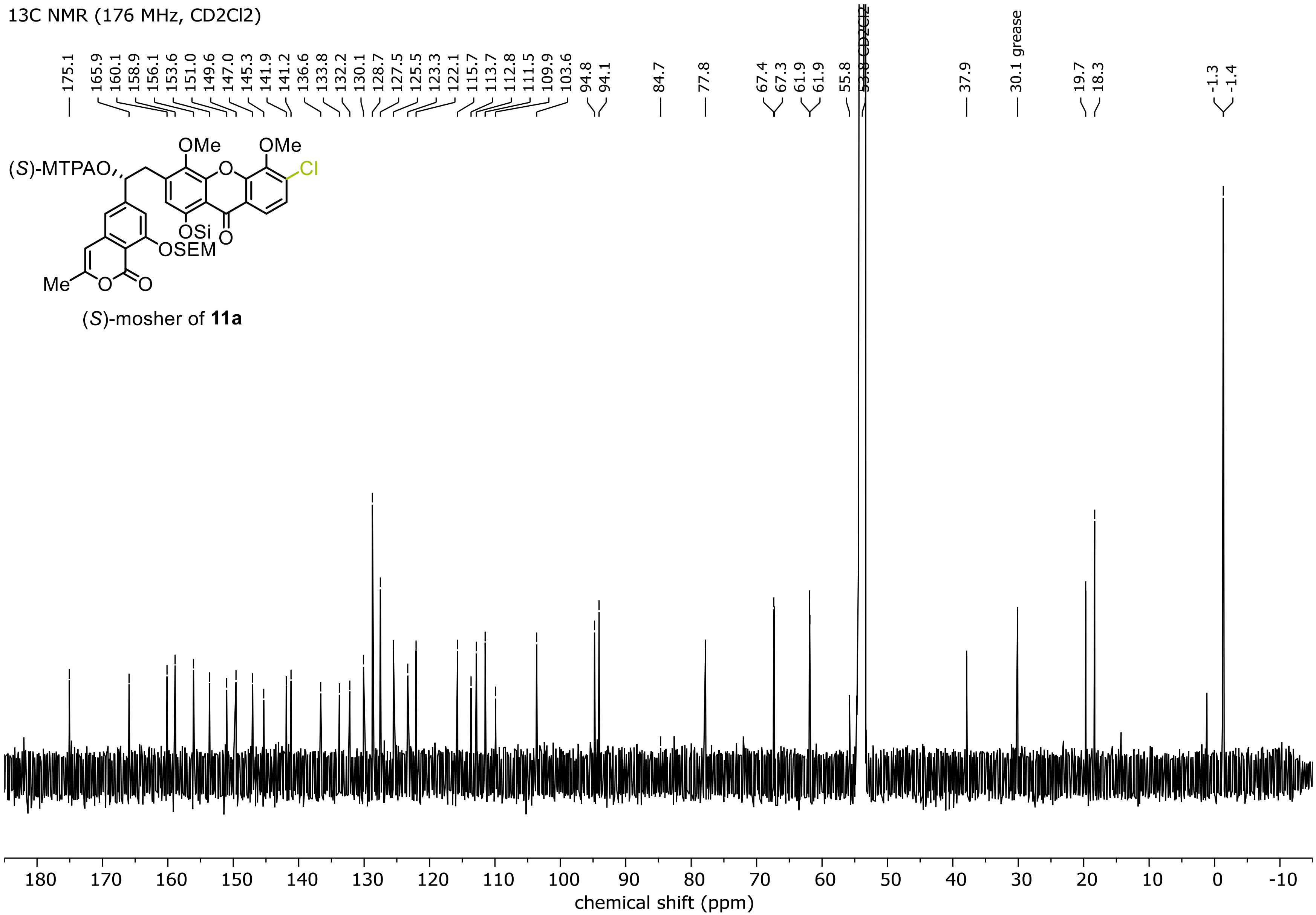

(*S*)-mosher of **11a**

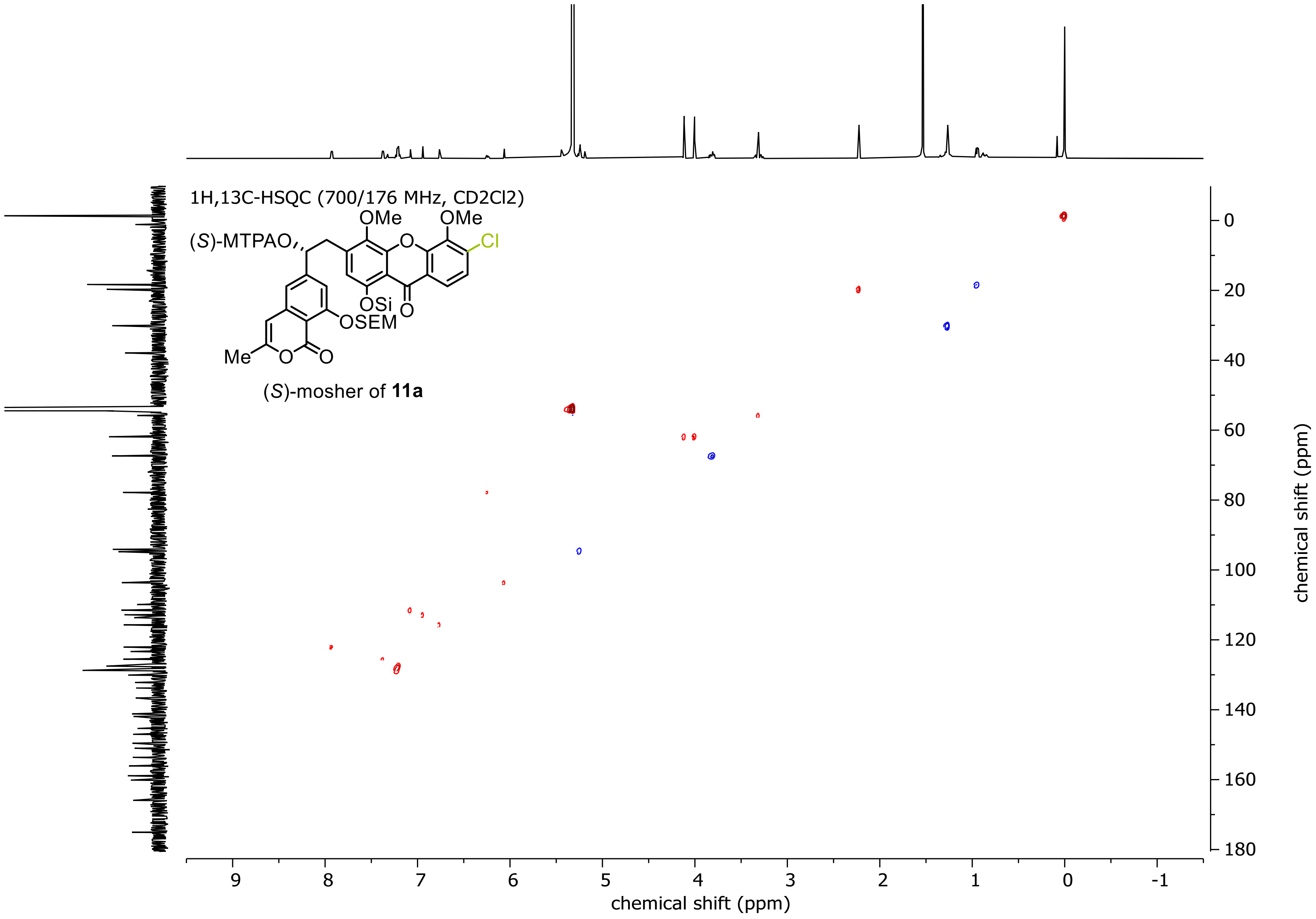
1H,13C-HSQC (700/176 MHz, CD2Cl2)
OMe
OMe
(S)-MTPAO
Cl
OSi
O
OSEM
Me
O
O
(S)-mosher of 11a
chemical shift (ppm)
chemical shift (ppm)

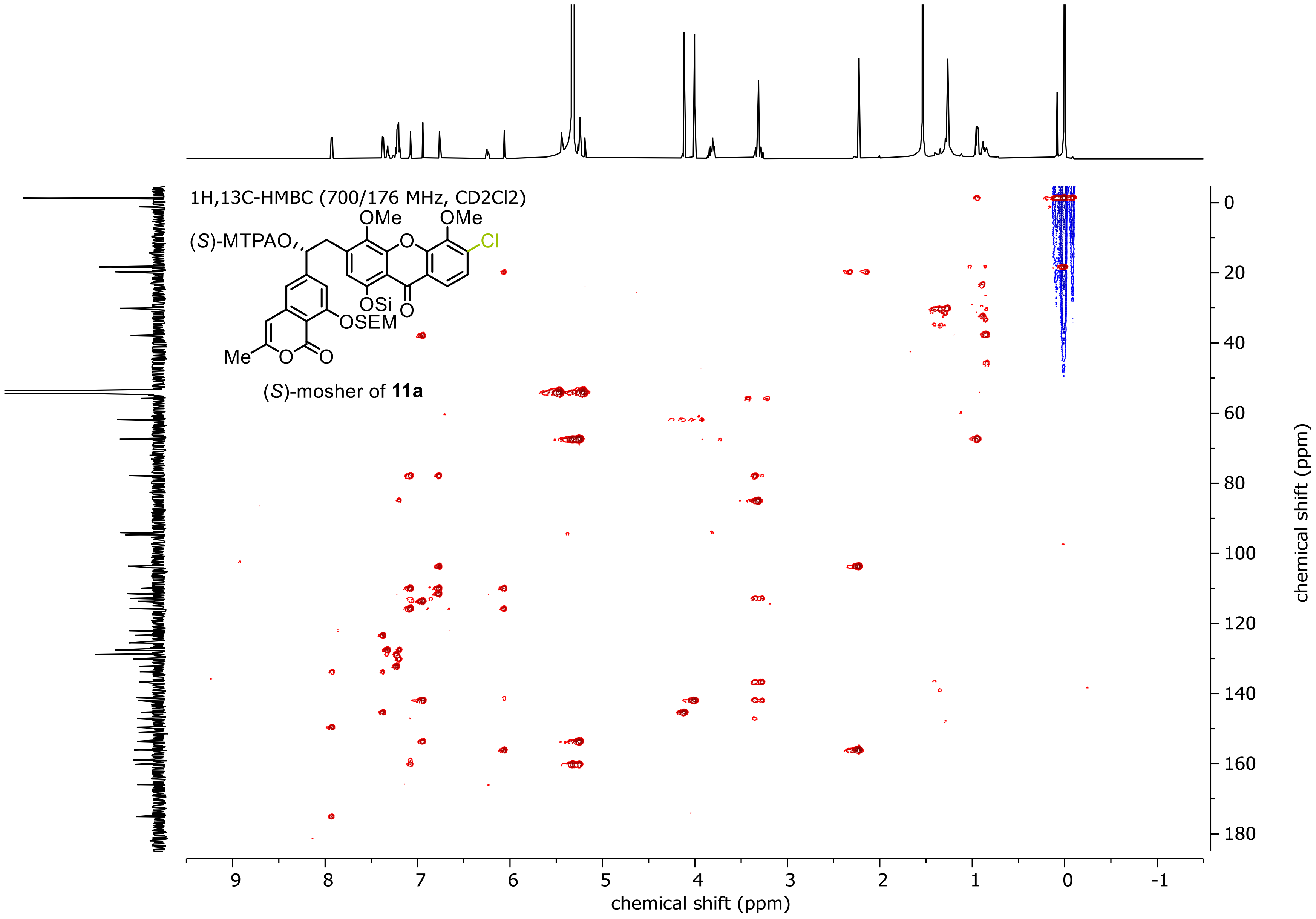
1H,13C-HMBC (700/176 MHz, CD2Cl2)
(S)-MTPAO
OMe
OMe
Cl
OSi
OSEM
Me
(S)-mosher of 11a
chemical shift (ppm)
chemical shift (ppm)

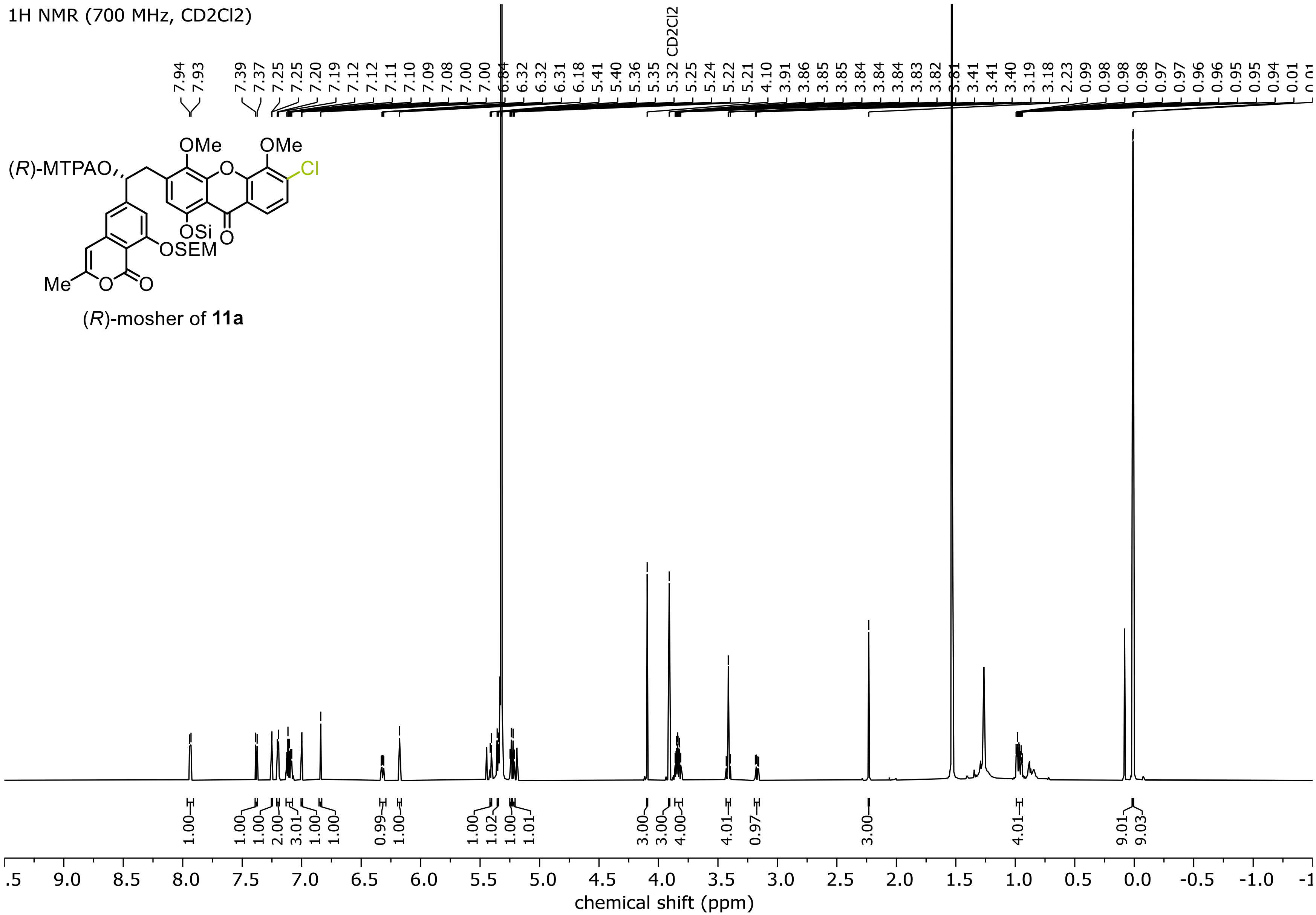
1H NMR (700 MHz, CD2Cl2)
CD2Cl2
7.94 7.93 7.39 7.37 7.25 7.25 7.20 7.19 7.12 7.12 7.11 7.10 7.09 7.08 7.00 7.00 6.84 6.32 6.32 6.31 6.18 5.41 5.40 5.36 5.35 5.32 5.25 5.24 5.22 5.21 4.10 3.91 3.86 3.85 3.85 3.84 3.84 3.84 3.83 3.82 3.81 3.41 3.41 3.40 3.19 3.18 2.23 0.99 0.98 0.98 0.98 0.97 0.97 0.96 0.96 0.95 0.95 0.94 0.01
OMe
OMe
(R)-MTPAO
Cl
OSi
O
OSEM
Me
O
O
(R)-mosher of **11a**
1.00 1.00 1.00 2.00 3.01 1.00 1.00 0.99 1.00 1.00 1.02 1.00 1.01 3.00 3.00 4.00 4.01 0.97 3.00 4.01 9.01 9.03
9.0 8.5 8.0 7.5 7.0 6.5 6.0 5.5 5.0 4.5 4.0 3.5 3.0 2.5 2.0 1.5 1.0 0.5 0.0 -0.5 -1.0
chemical shift (ppm)

13C NMR (176 MHz, CD2Cl2)

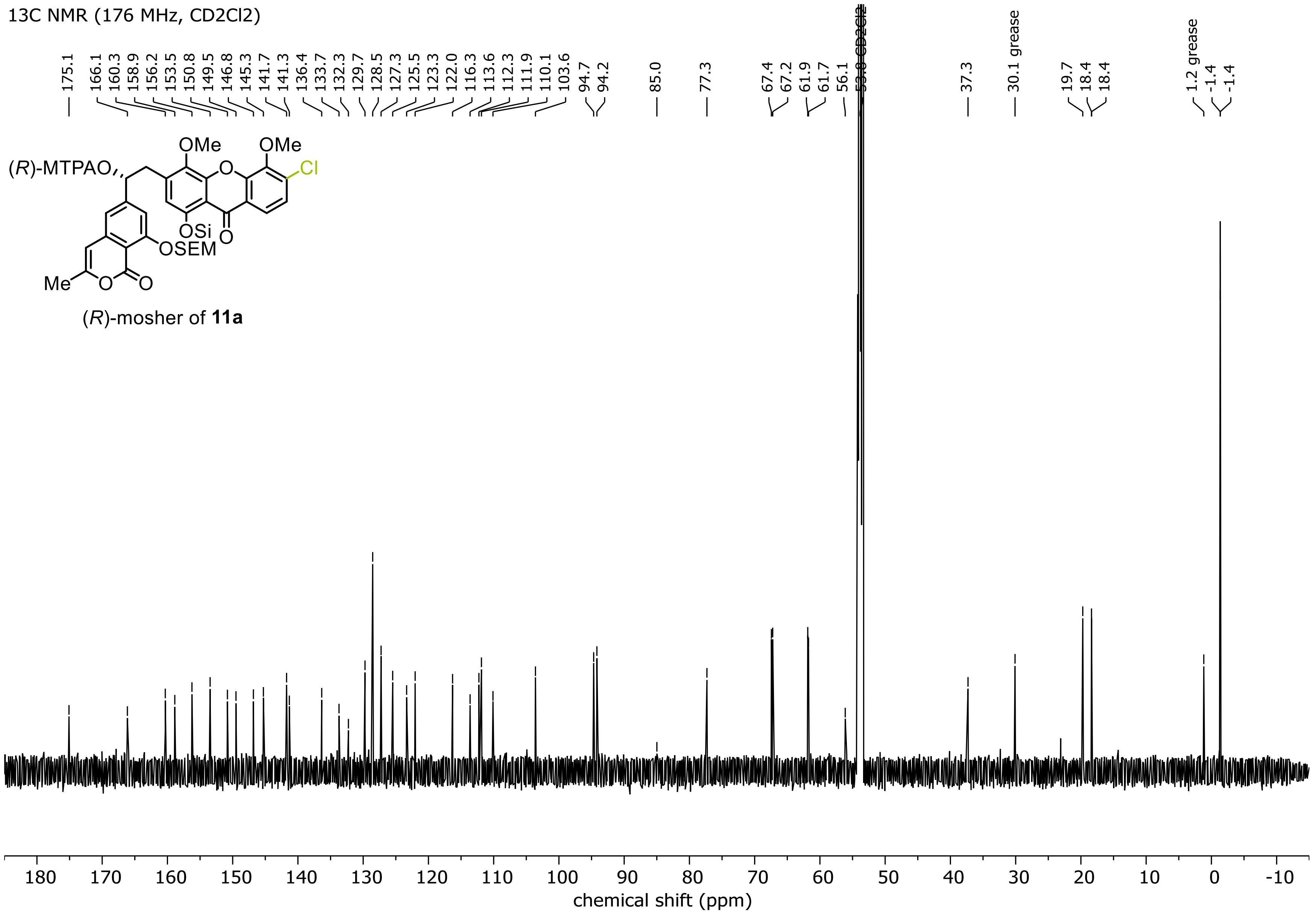

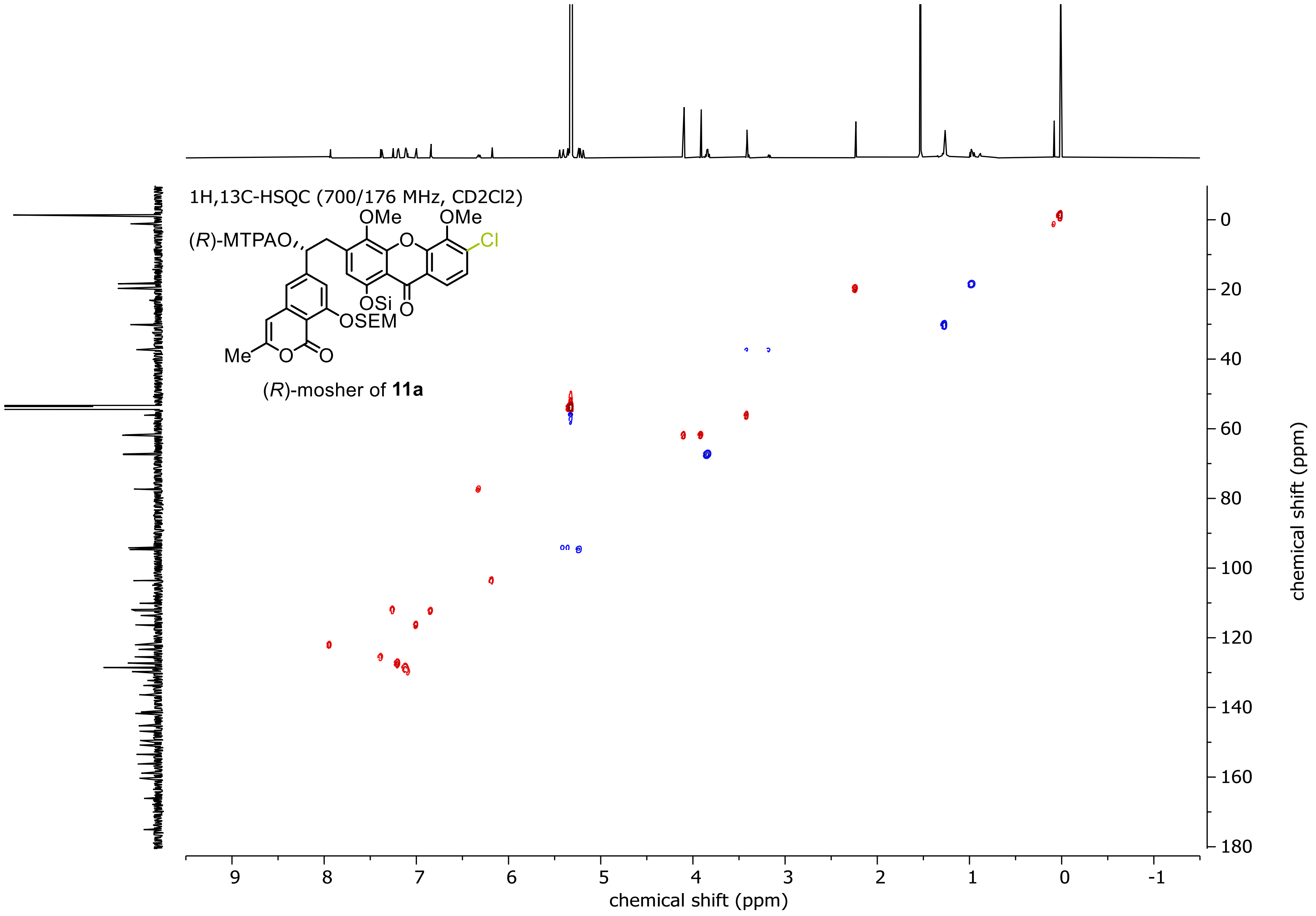

1H,13C-HSQC (700/176 MHz, CD2Cl2)
(R)-MTPAO
OMe
OMe
Cl
OSi
OSEM
Me
(R)-mosher of 11a
chemical shift (ppm)
chemical shift (ppm)

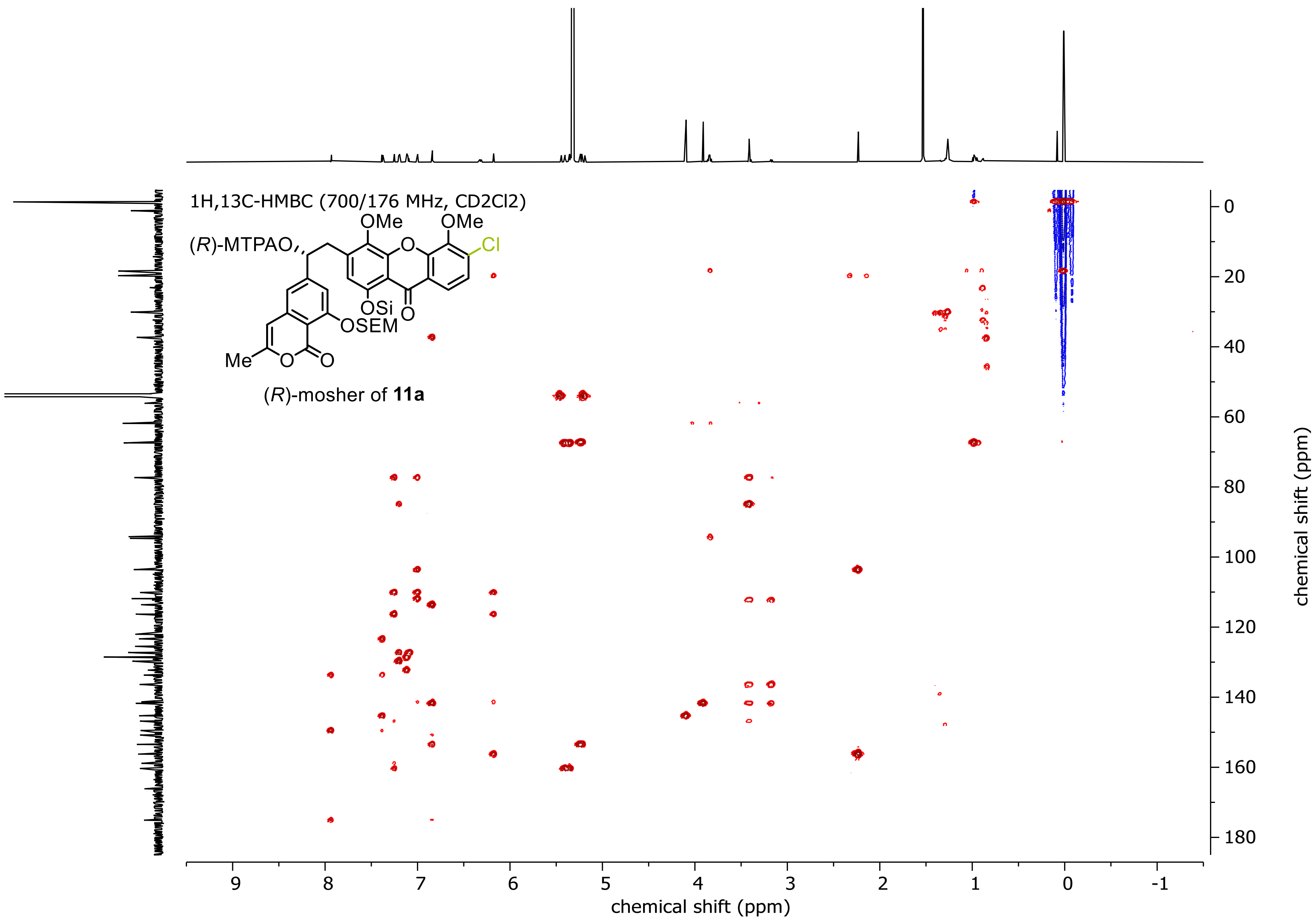

1H,13C-HMBC (700/176 MHz, CD2Cl2)
OMe
OMe
(R)-MTPAO
Cl
OSi
O
OSEM
Me
O
(R)-mosher of 11a
chemical shift (ppm)
chemical shift (ppm)

1H NMR (700 MHz, CD2Cl2)

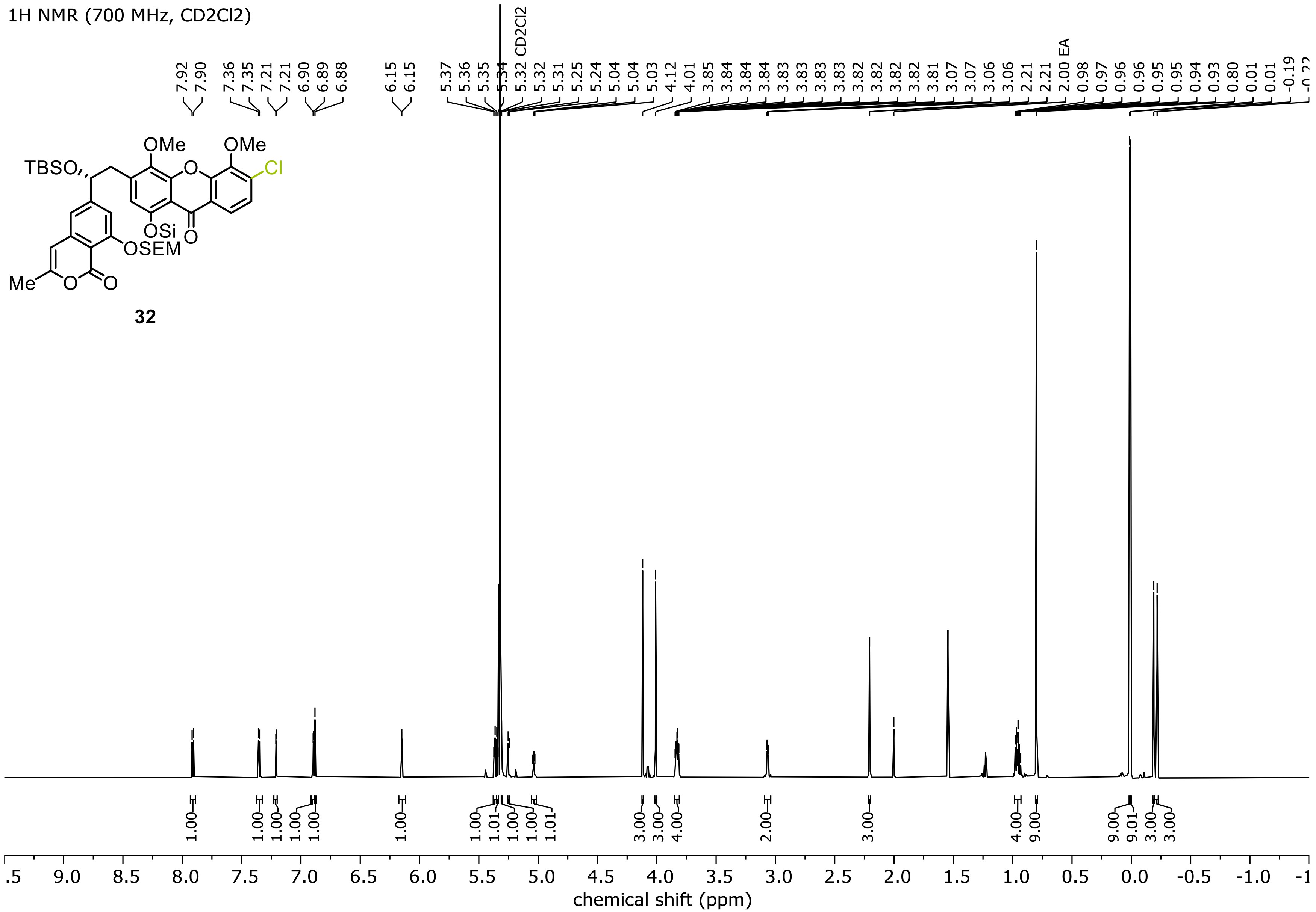

32

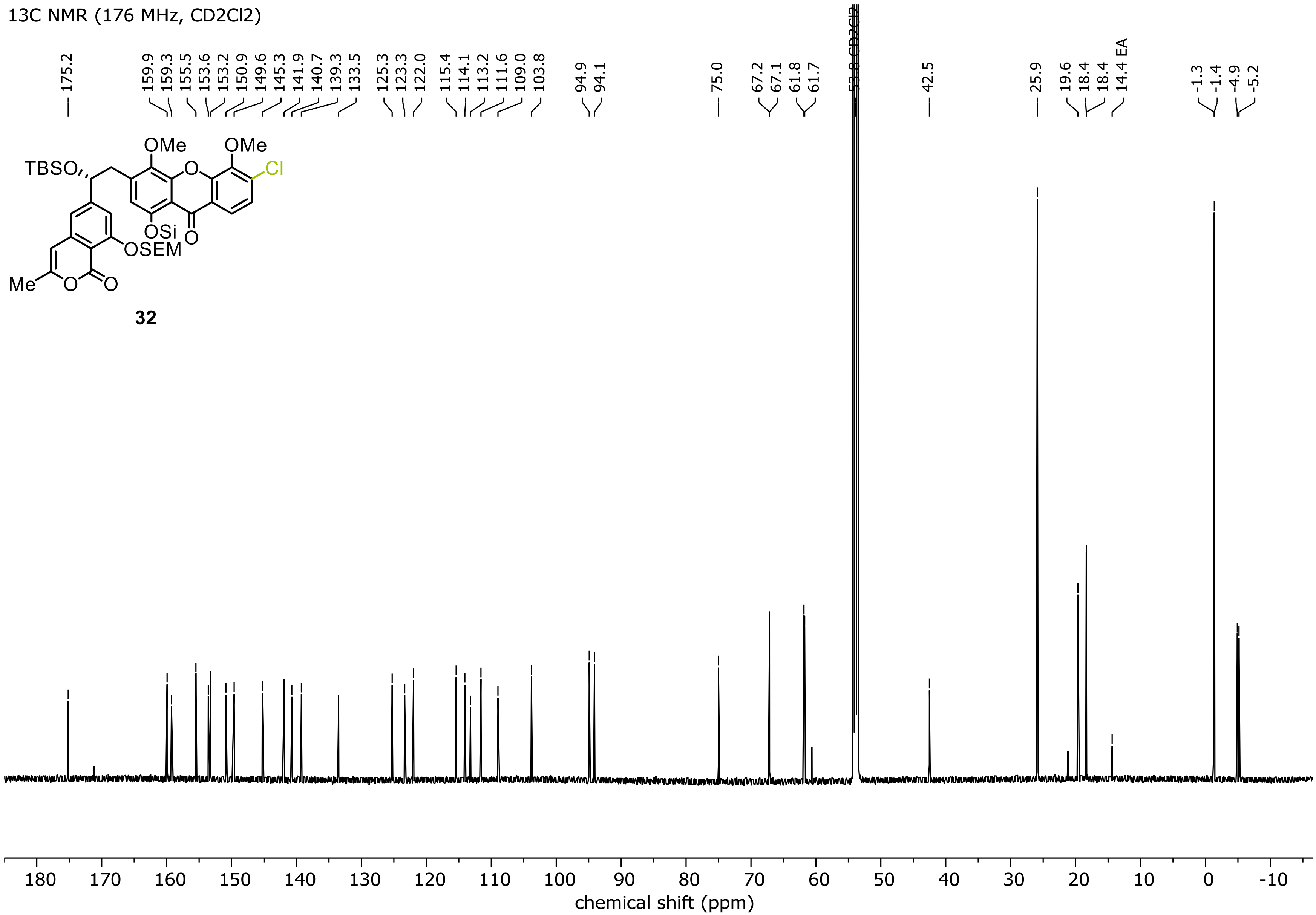

13C NMR (176 MHz, CD2Cl2)
175.2
159.9
159.3
155.5
153.6
153.2
150.9
149.6
145.3
141.9
140.7
139.3
133.5
125.3
123.3
122.0
115.4
114.1
113.2
111.6
109.0
103.8
94.9
94.1
75.0
67.2
67.1
61.8
61.7
53.8 CD2Cl2
42.5
25.9
19.6
18.4
18.4
14.4 EA
-1.3
-1.4
-4.9
-5.2
OMe
OMe
TBSO
Cl
OSi
O
OSEM
Me
O
O
32
chemical shift (ppm)

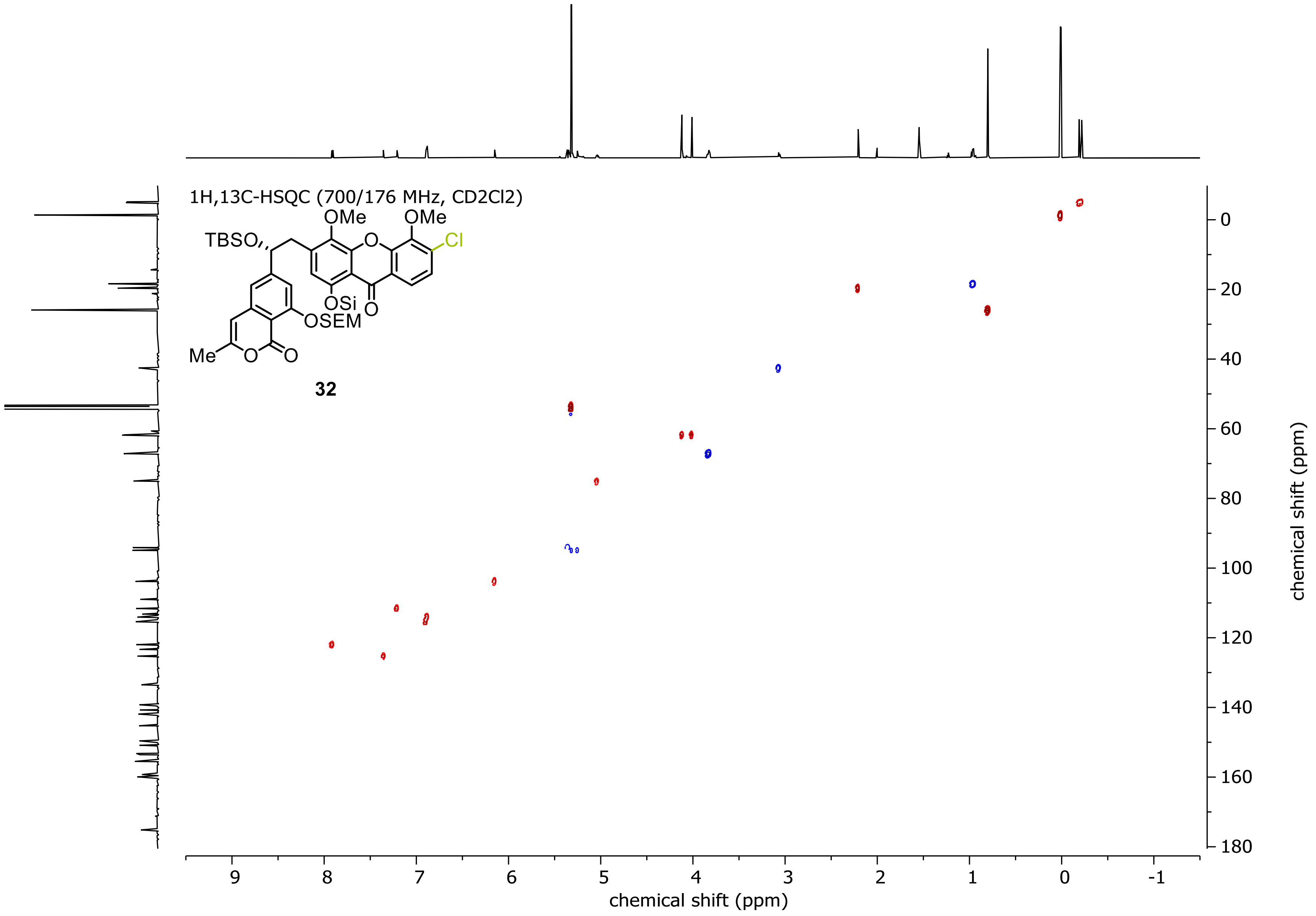

1H,13C-HSQC (700/176 MHz, CD2Cl2)
TBSO
OMe
OMe
Cl
OSi
O
OSEM
Me
O
O
32
9
8
7
6
5
4
3
2
1
0
-1
chemical shift (ppm)
0
20
40
60
80
100
120
140
160
180
chemical shift (ppm)

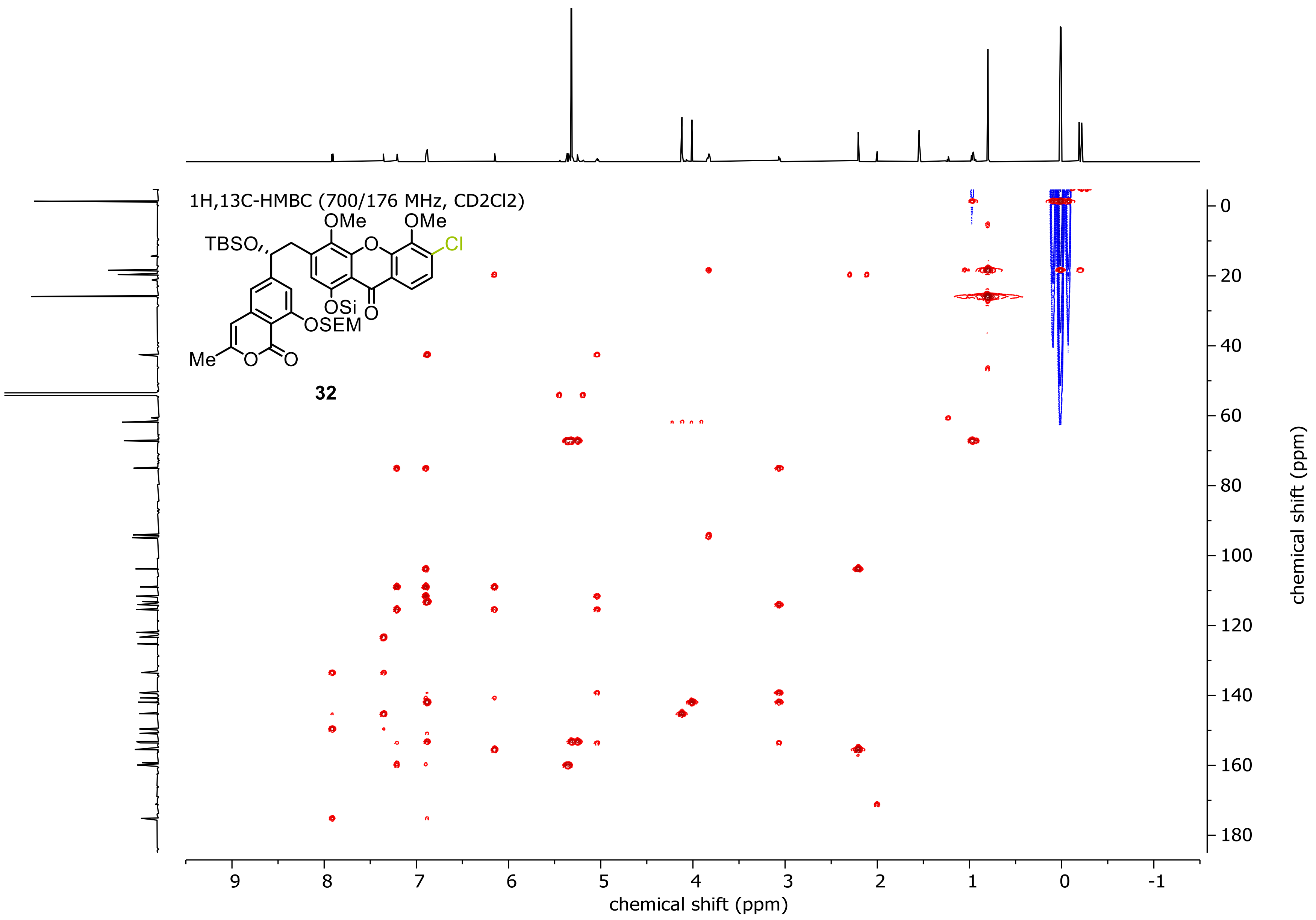
1H,13C-HMBC (700/176 MHz, CD2Cl2)
OMe
OMe
TBSO
Cl
OSi
O
OSEM
Me
O
O
32
chemical shift (ppm)
chemical shift (ppm)
9 8 7 6 5 4 3 2 1 0 -1
0 20 40 60 80 100 120 140 160 180

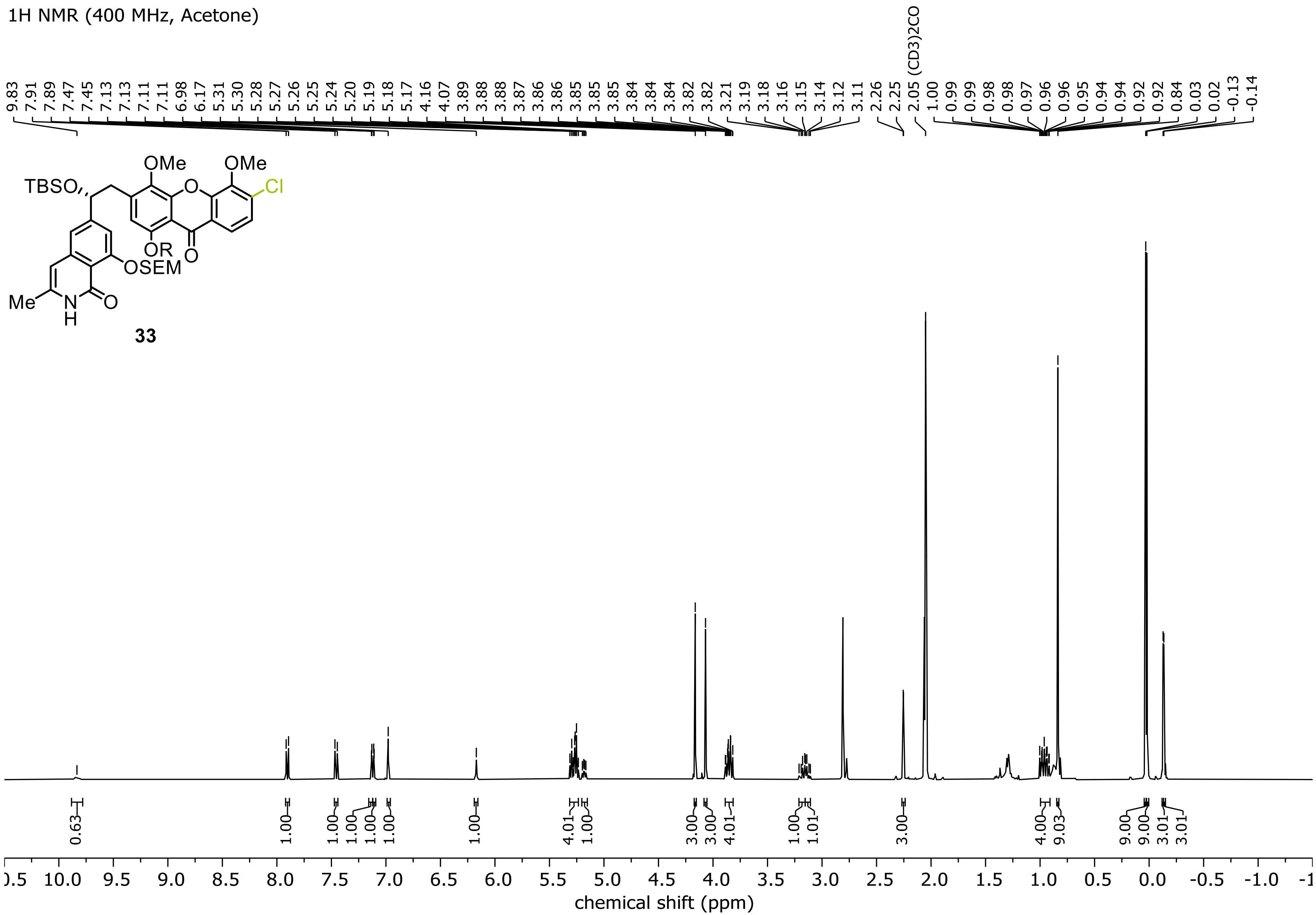
1H NMR (400 MHz, Acetone)
9.83 7.91 7.89 7.47 7.45 7.13 7.13 7.11 7.11 6.98 6.17 5.31 5.30 5.28 5.27 5.26 5.25 5.24 5.20 5.19 5.18 5.17 4.16 4.07 3.89 3.88 3.88 3.87 3.86 3.86 3.85 3.85 3.85 3.84 3.84 3.84 3.82 3.82 3.21 3.19 3.18 3.16 3.15 3.14 3.12 3.11 2.26 2.25 2.05 (CD3)2CO 1.00 0.99 0.99 0.98 0.98 0.97 0.96 0.96 0.95 0.94 0.94 0.92 0.92 0.84 0.03 0.02 -0.13 -0.14
OMe
OMe
TBSO
Cl
OR
OSEM
O
Me
N
H
33
0.63 1.00 1.00 1.00 1.00 1.00 1.00 4.01 1.00 3.00 3.00 4.01 1.00 1.01 3.00 4.00 9.03 9.00 9.00 3.01 3.01
chemical shift (ppm)

1H NMR (700 MHz, Acetone)

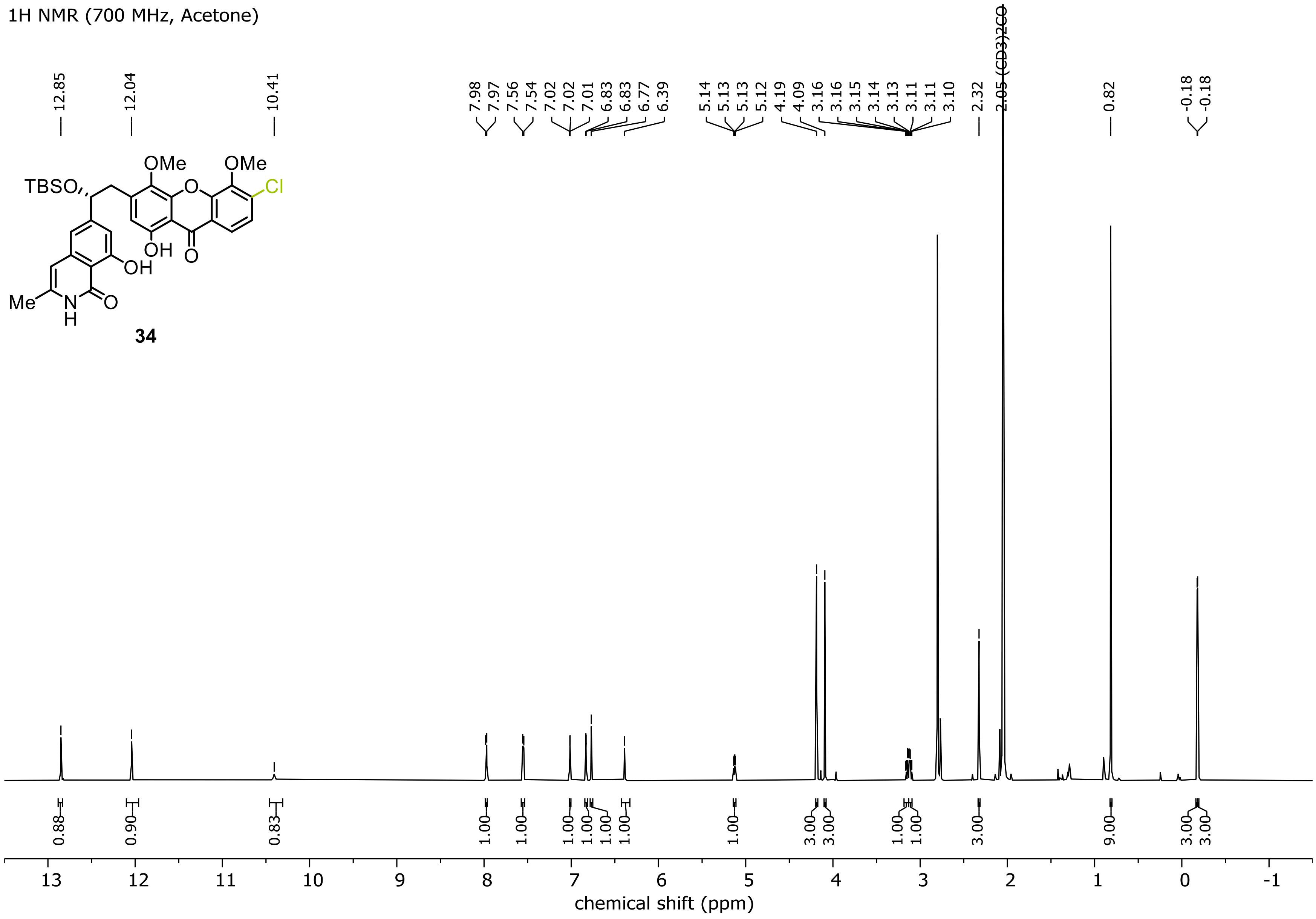

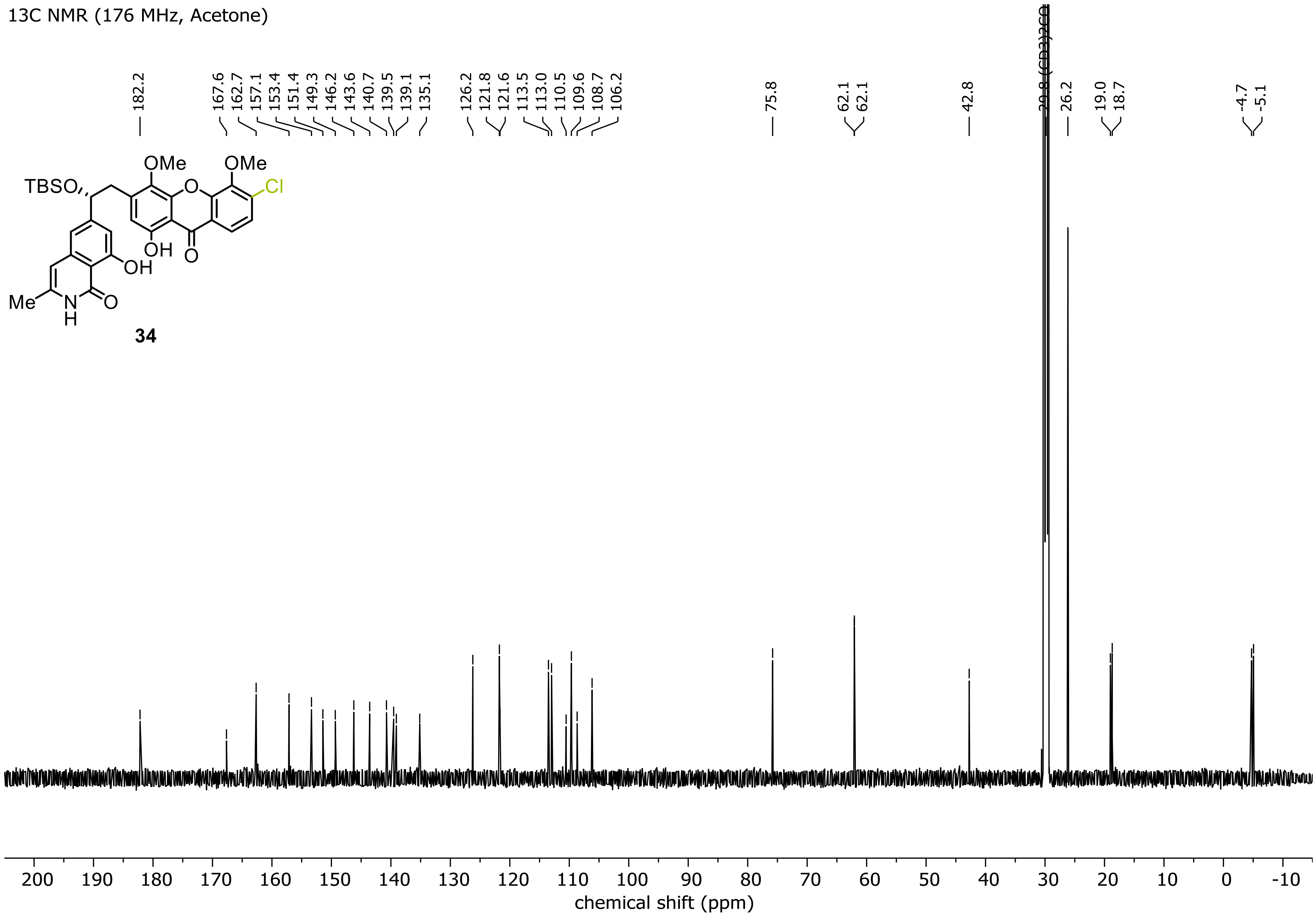
13C NMR (176 MHz, Acetone)
182.2
167.6
162.7
157.1
153.4
151.4
149.3
146.2
143.6
140.7
139.5
139.1
135.1
126.2
121.8
121.6
113.5
113.0
110.5
109.6
108.7
106.2
75.8
62.1
62.1
42.8
26.2
19.0
18.7
-4.7
-5.1
OMe
OMe
TBSO
O
Cl
OH
O
OH
Me
N
H
O
34
200 190 180 170 160 150 140 130 120 110 100 90 80 70 60 50 40 30 20 10 0 -10
chemical shift (ppm)

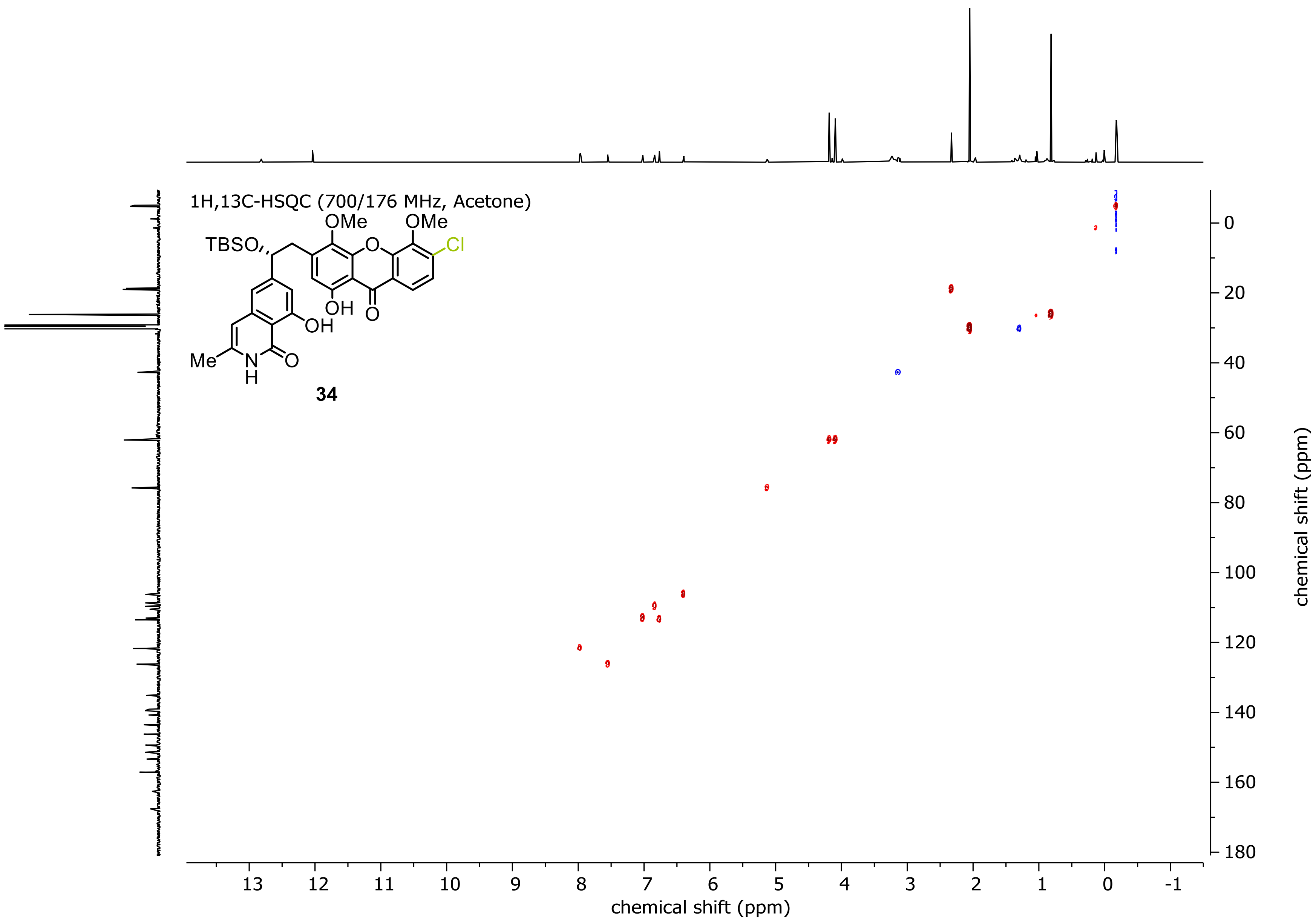

1H,13C-HSQC (700/176 MHz, Acetone)
OMe
OMe
TBSO
O
Cl
OH
O
OH
Me
N
H
O
34
chemical shift (ppm)
chemical shift (ppm)

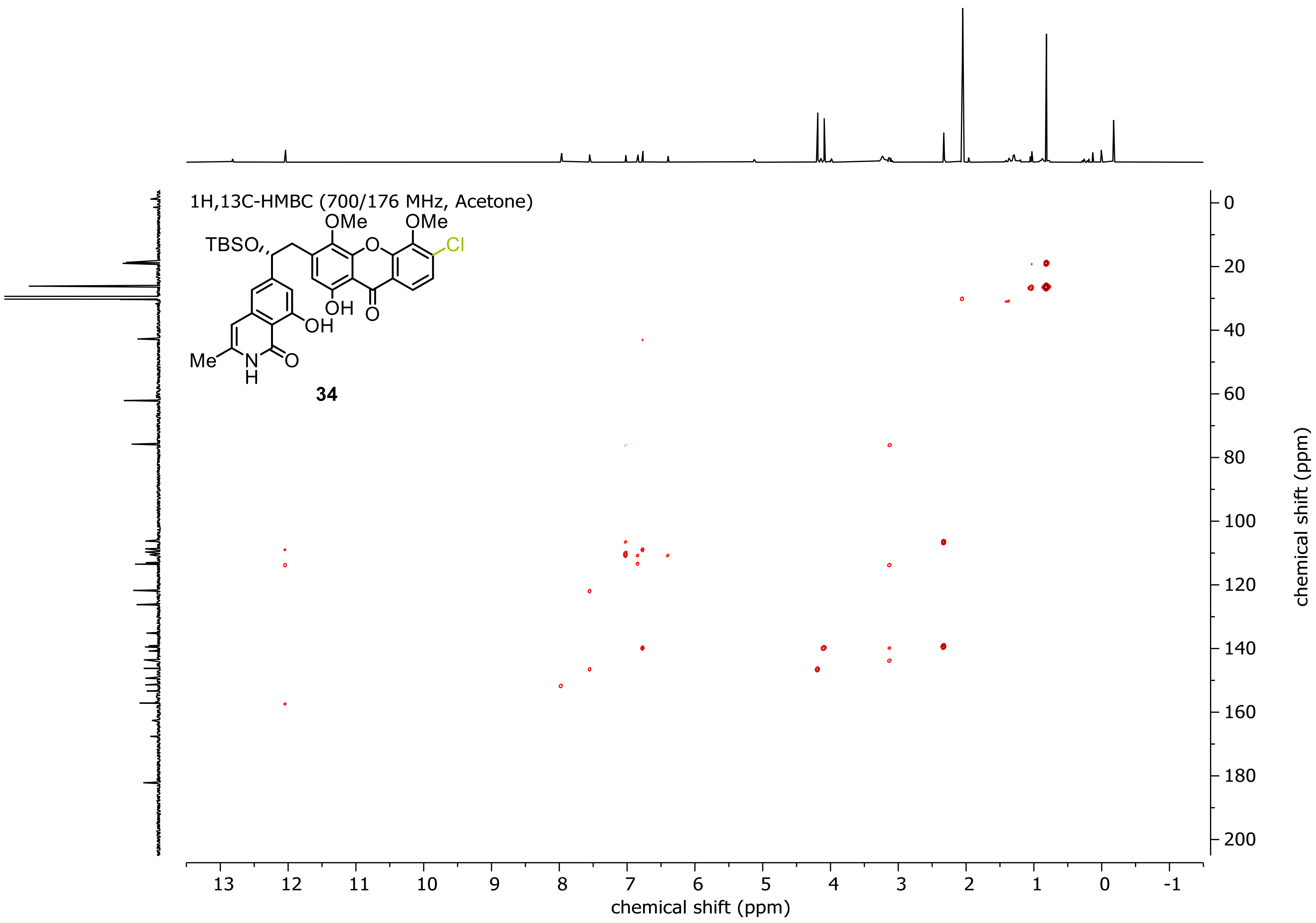

1H,13C-HMBC (700/176 MHz, Acetone)
34
chemical shift (ppm)
chemical shift (ppm)

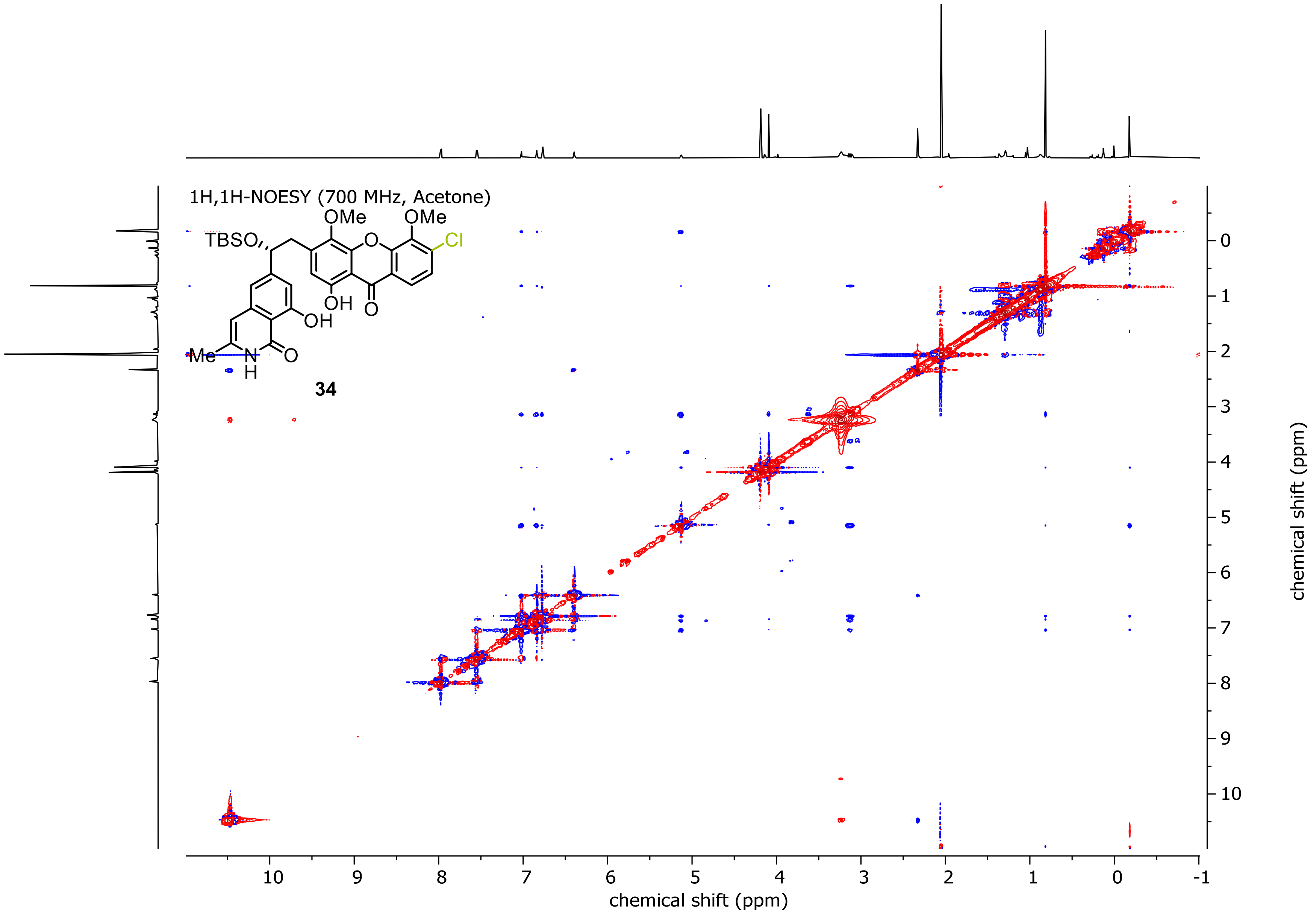
1H,1H-NOESY (700 MHz, Acetone)
TBSO
OMe
OMe
O
Cl
OH
O
OH
Me
N
H
O
34
chemical shift (ppm)
chemical shift (ppm)

1H NMR (700 MHz, Acetone)

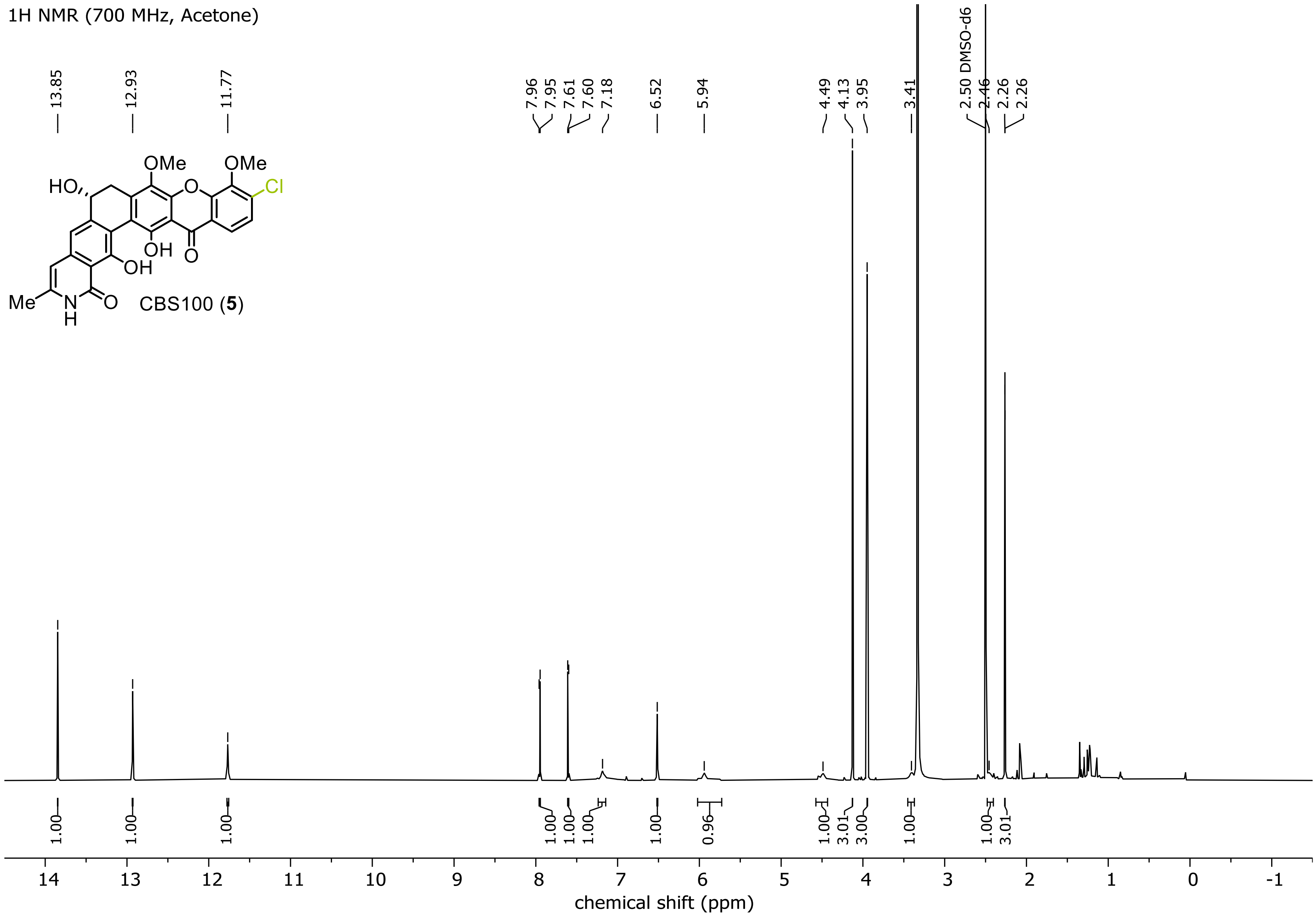

13C NMR (176 MHz, Acetone)

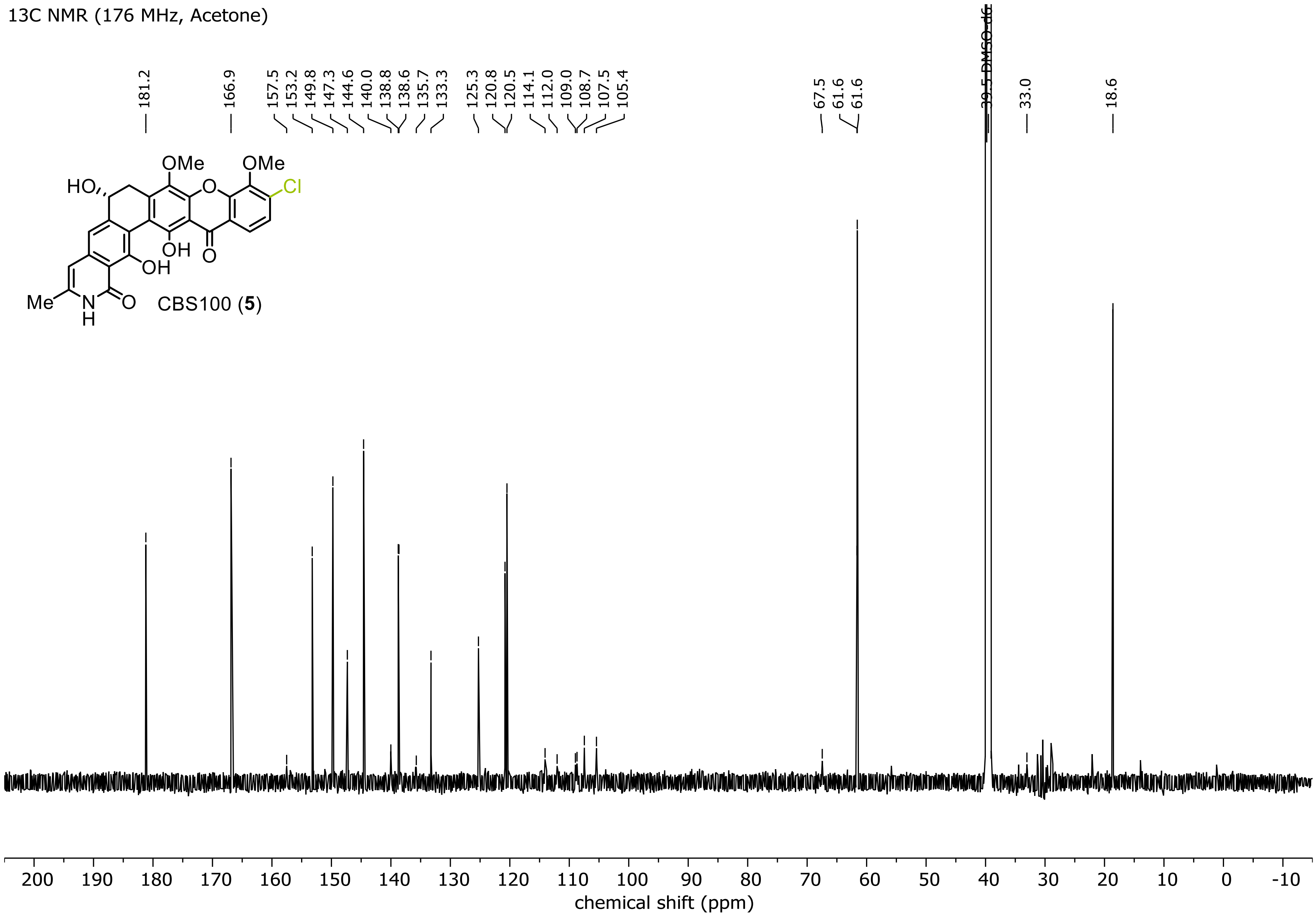

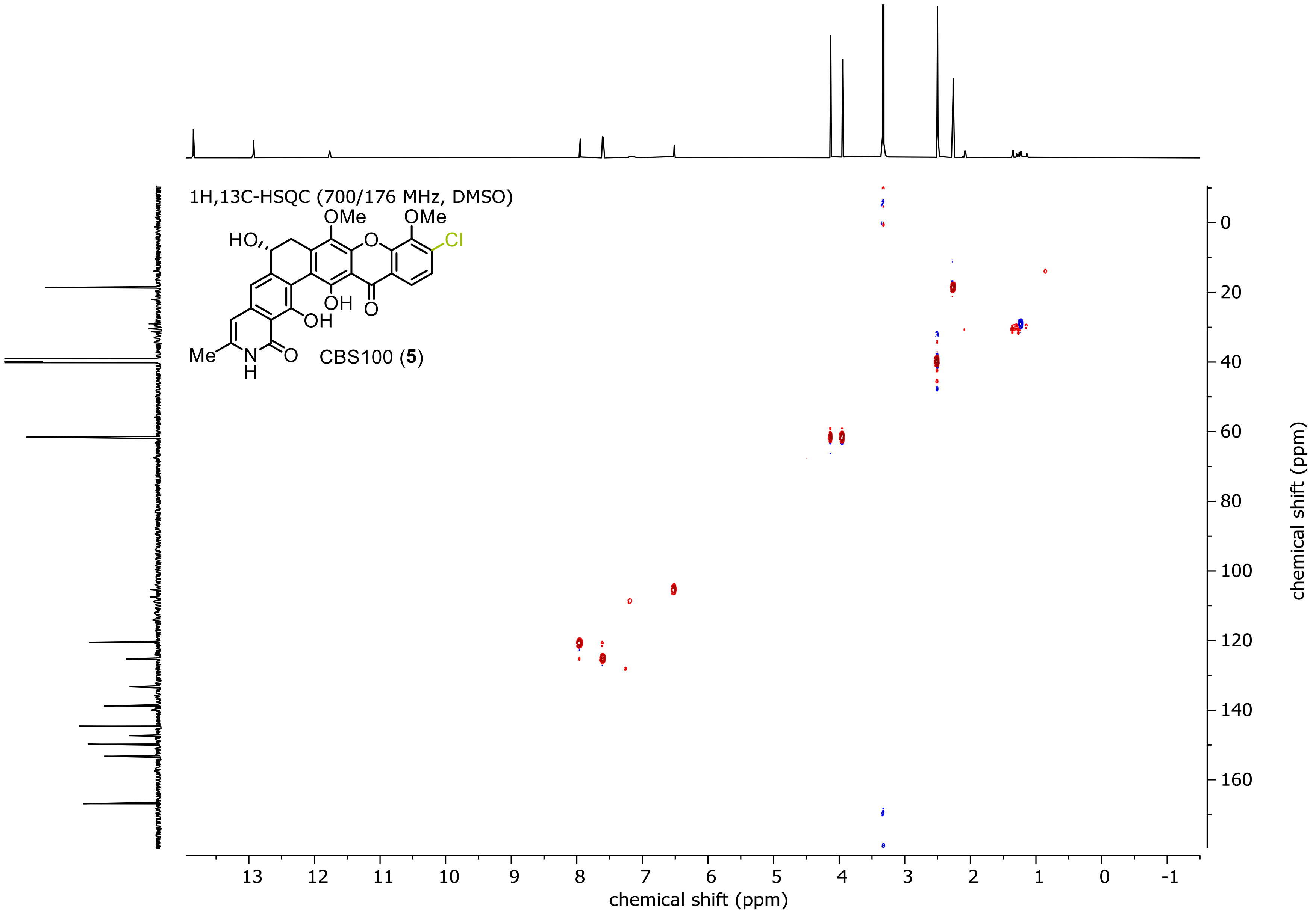

1H,13C-HSQC (700/176 MHz, DMSO)
OMe
OMe
HO
O
Cl
OH
O
OH
Me
N
H
O
CBS100 (**5**)
chemical shift (ppm)
chemical shift (ppm)

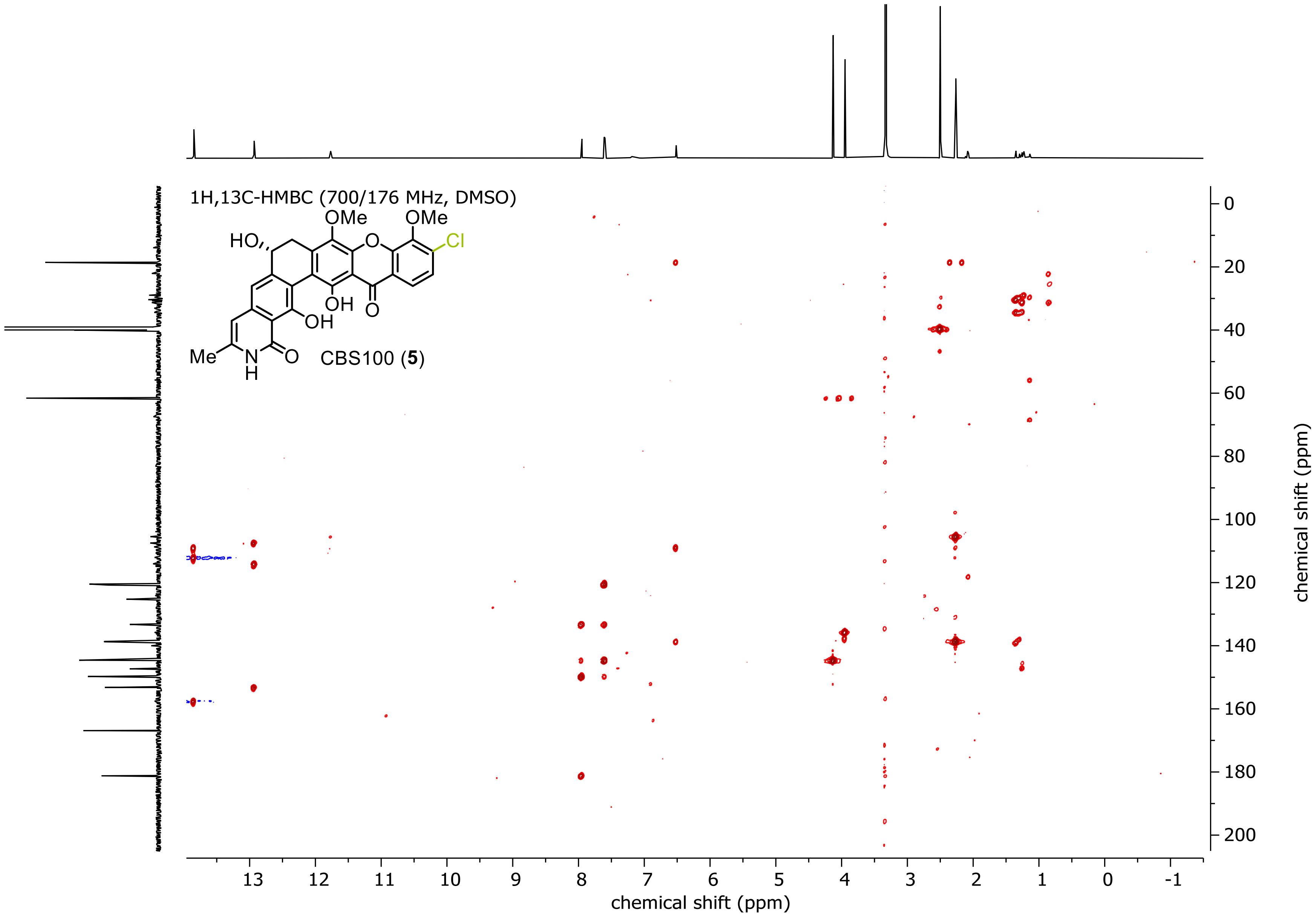

1H,13C-HMBC (700/176 MHz, DMSO)
OMe
OMe
HO
Cl
OH
O
OH
Me
N
H
O
CBS100 (5)
chemical shift (ppm)
chemical shift (ppm)

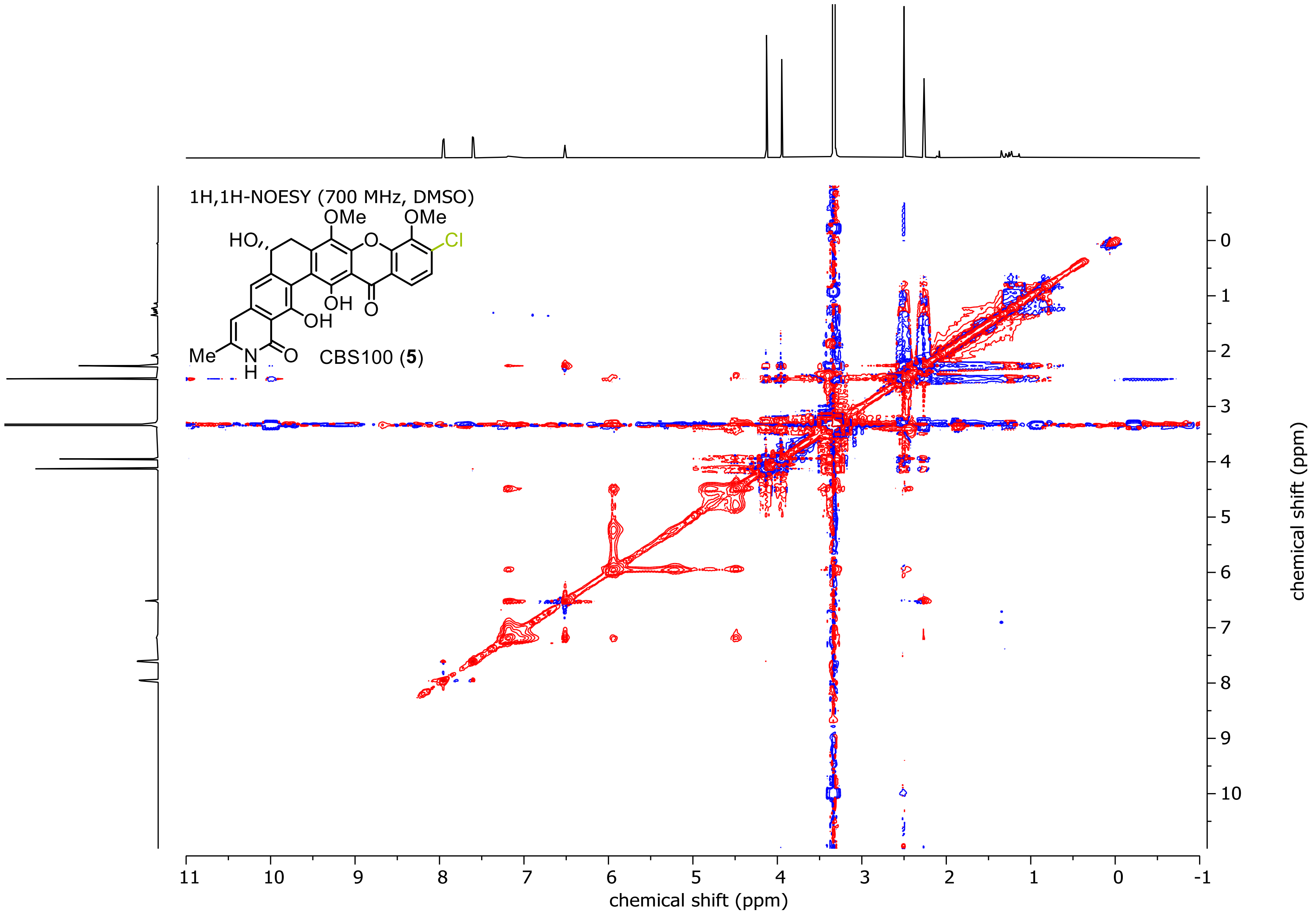
1H,1H-NOESY (700 MHz, DMSO)
OMe
OMe
HO
Cl
OH
OH
O
Me
N
H
O
CBS100 (5)
chemical shift (ppm)
chemical shift (ppm)

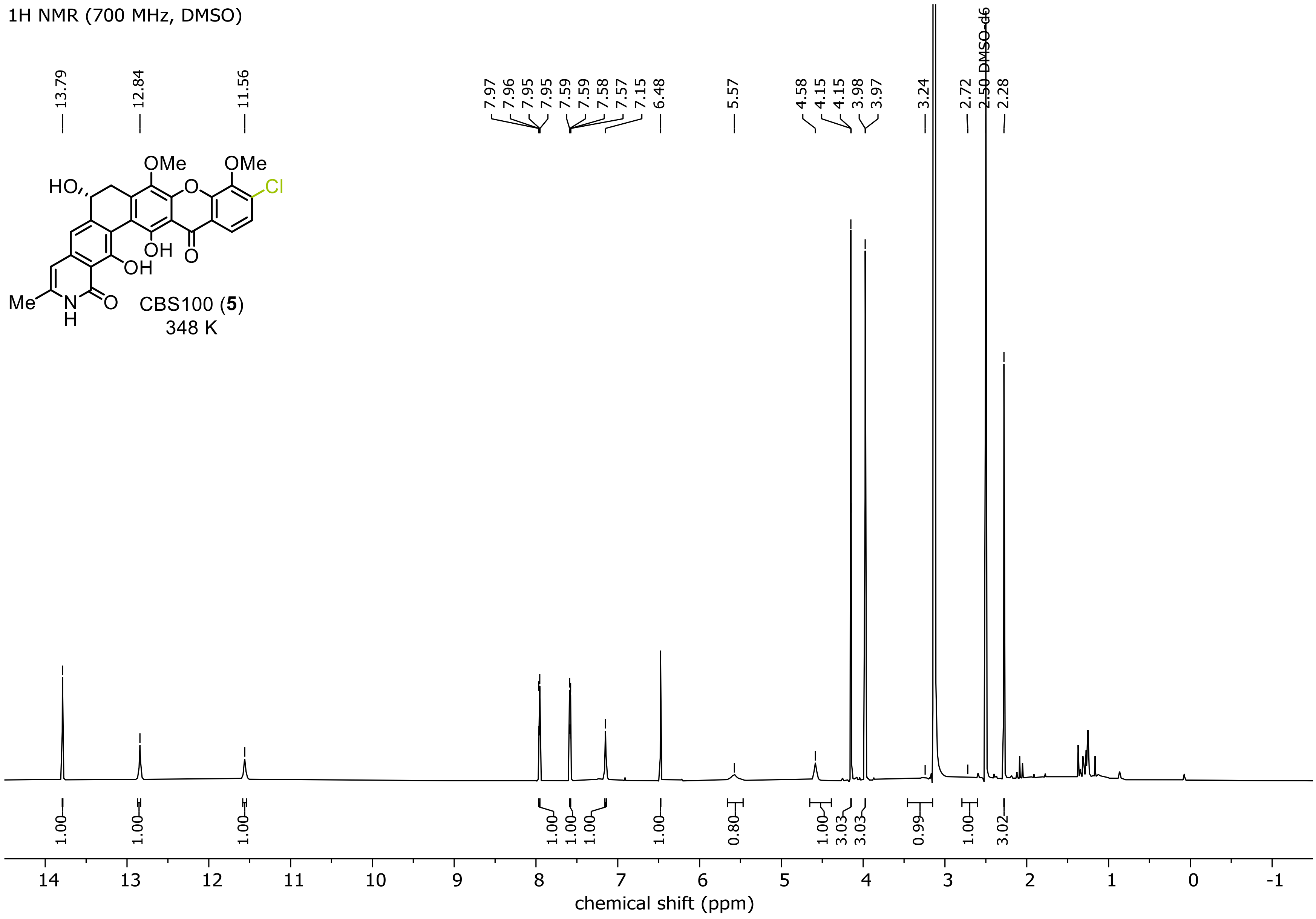
1H NMR (700 MHz, DMSO)
13.79
12.84
11.56
7.97
7.96
7.95
7.95
7.59
7.59
7.58
7.57
7.15
6.48
5.57
4.58
4.15
4.15
3.98
3.97
3.24
2.72
2.50 DMSO-d6
2.28
OMe
OMe
HO
O
Cl
OH
OH
O
Me
N
H
O
CBS100 (5)
348 K
1.00
1.00
1.00
1.00
1.00
1.00
1.00
0.80
1.00
3.03
3.03
0.99
1.00
3.02
14
13
12
11
10
9
8
7
6
5
4
3
2
1
0
-1
chemical shift (ppm)

13C NMR (176 MHz, DMSO)

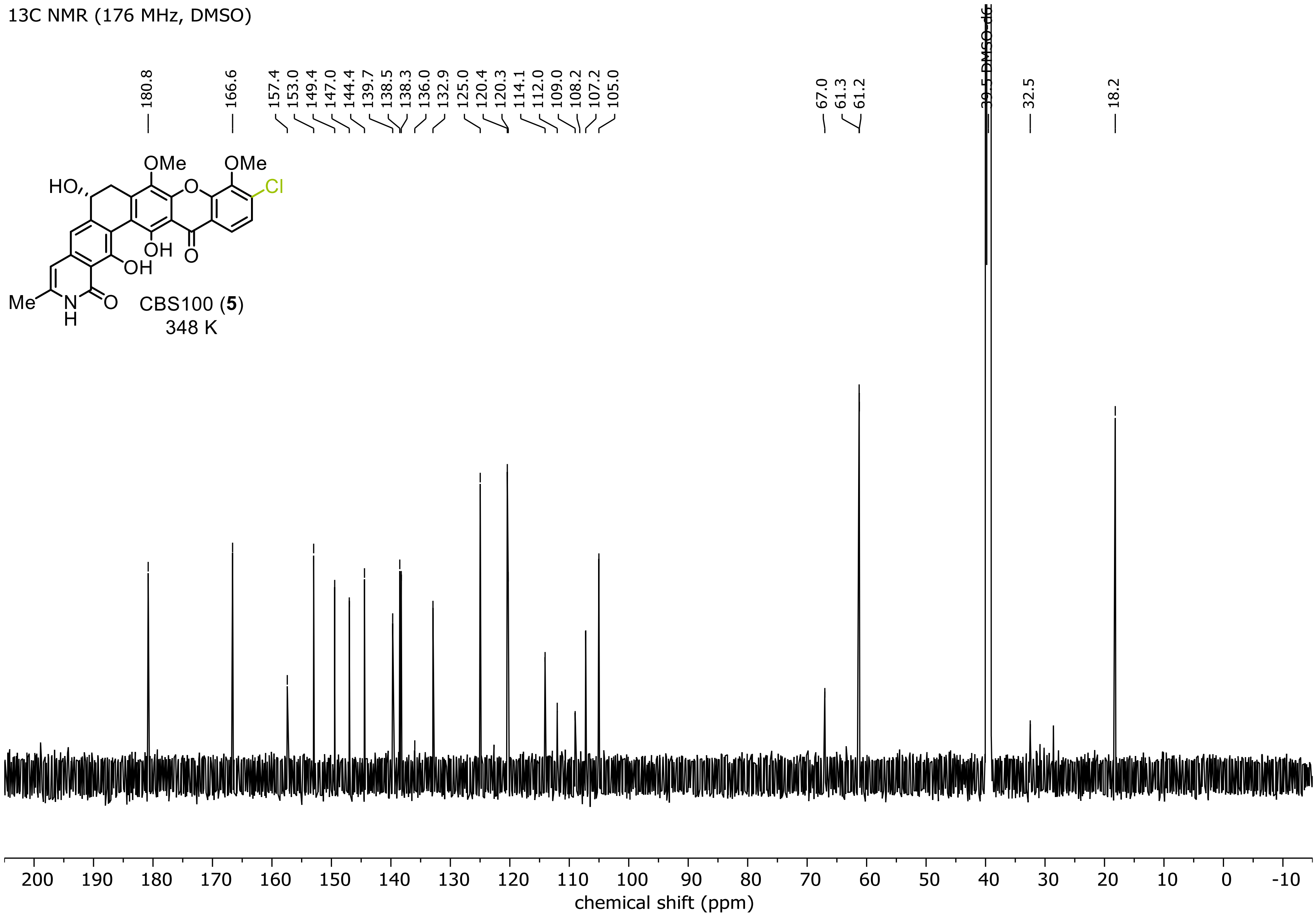

1H NMR (700 MHz, DMSO)

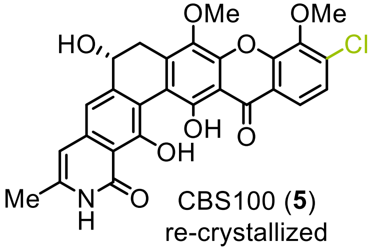

CBS100 (**5**)
re-crystallized

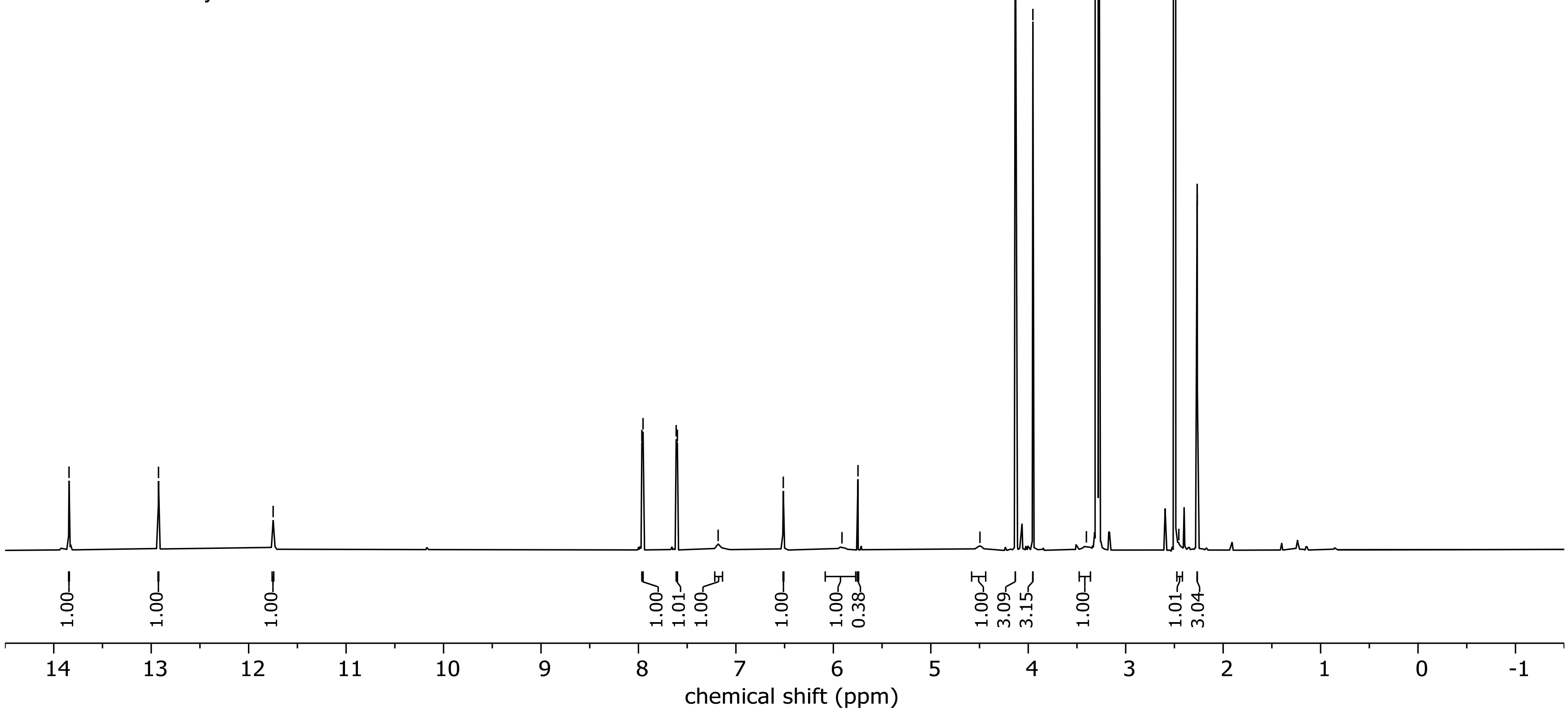

7.4 Leading to CBS87 (**4**)

1H NMR (500 MHz, Acetone)

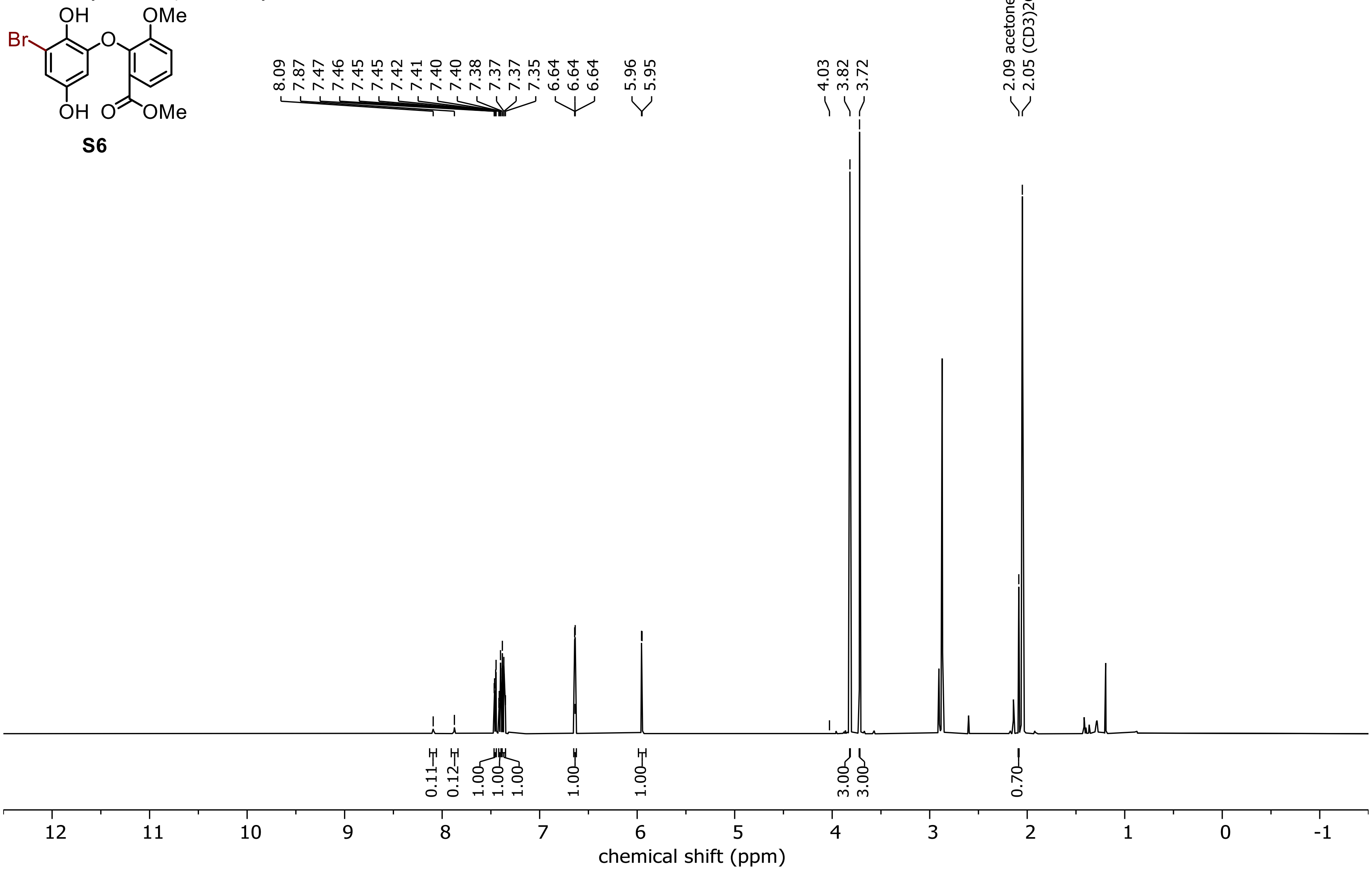

S6

13C NMR (125 MHz, Acetone)

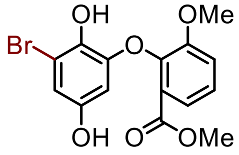

S6

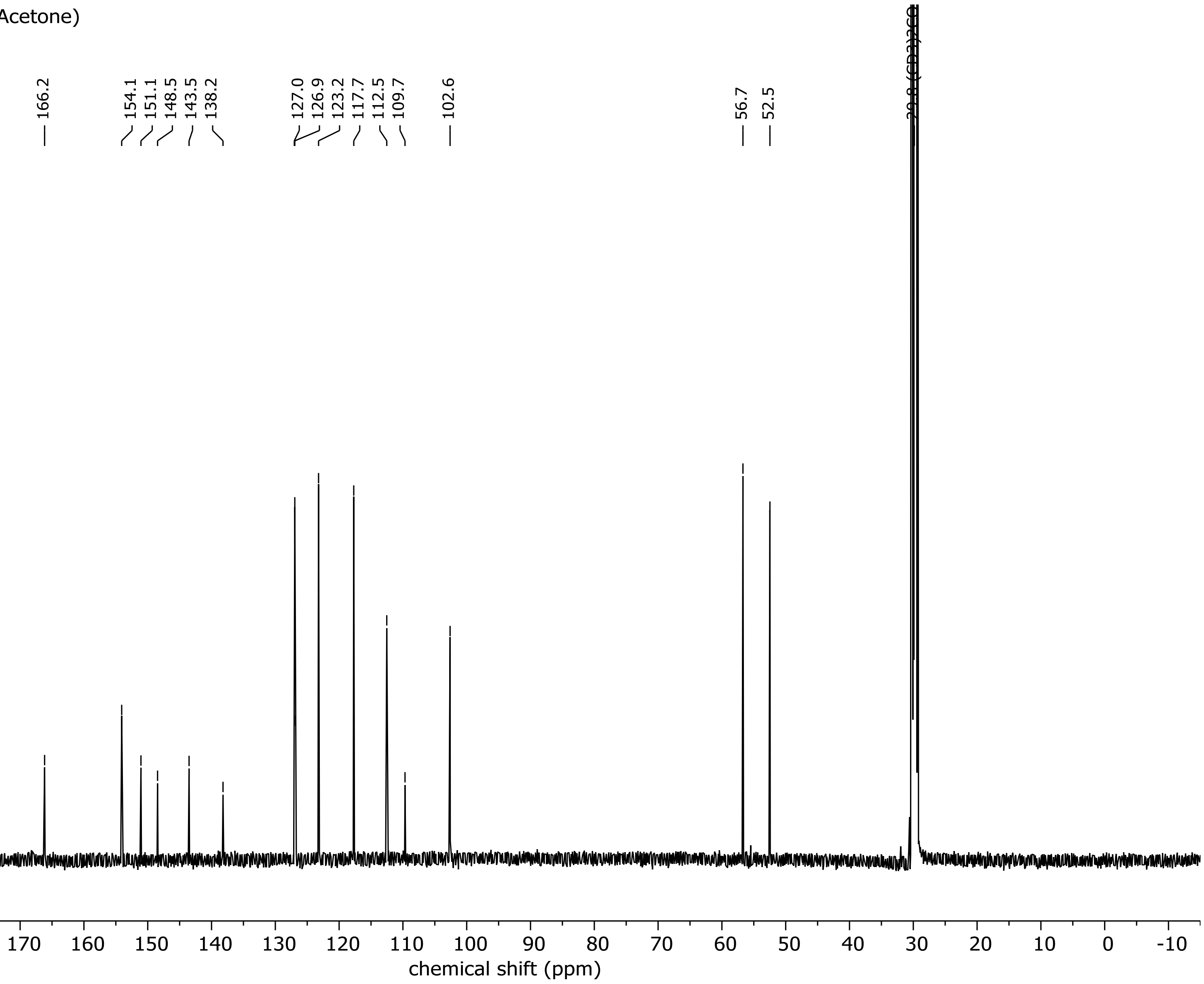

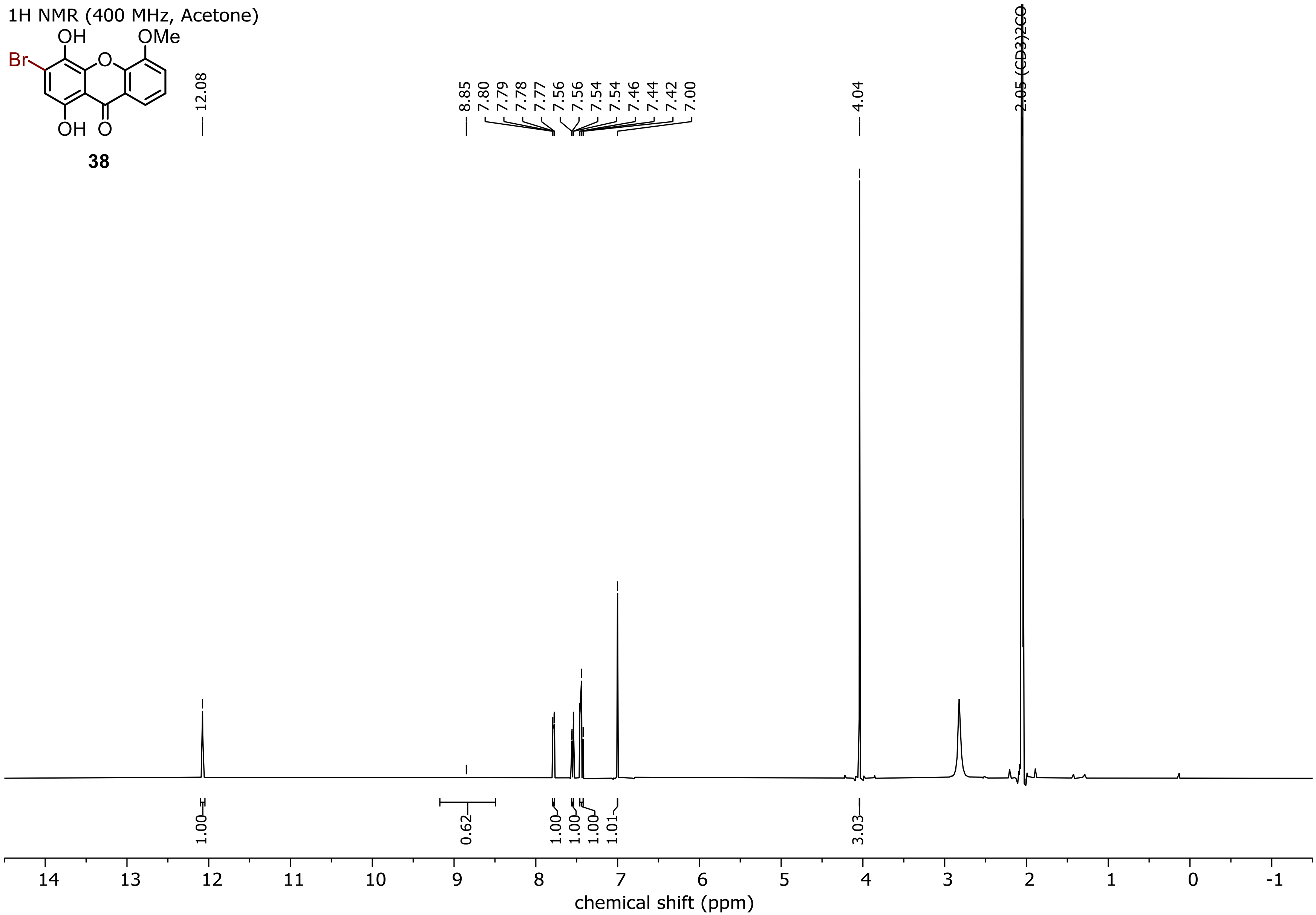
1H NMR (400 MHz, Acetone)
OH
OMe
Br
O
OH
O
38
12.08
8.85
7.80
7.79
7.78
7.77
7.56
7.56
7.54
7.54
7.46
7.44
7.42
7.00
4.04
2.05 (CD3)2CO
1.00
0.62
1.00
1.00
1.00
1.01
3.03
14
13
12
11
10
9
8
7
6
5
4
3
2
1
0
-1
chemical shift (ppm)

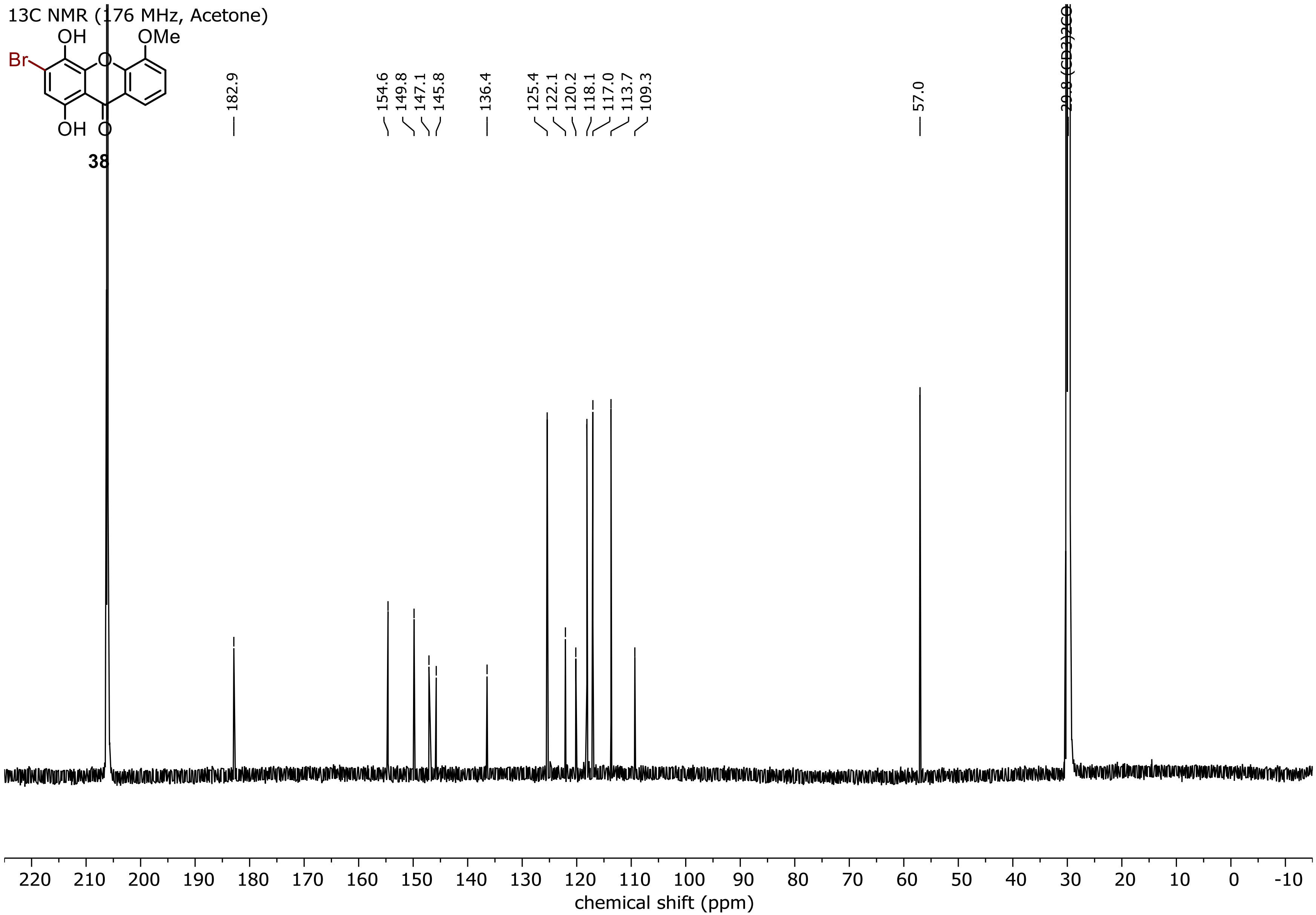
13C NMR (176 MHz, Acetone)
OH
OMe
Br
O
OH
O
38
182.9
154.6
149.8
147.1
145.8
136.4
125.4
122.1
120.2
118.1
117.0
113.7
109.3
57.0
29.8 (CD3)2CO
220 210 200 190 180 170 160 150 140 130 120 110 100 90 80 70 60 50 40 30 20 10 0 -10
chemical shift (ppm)

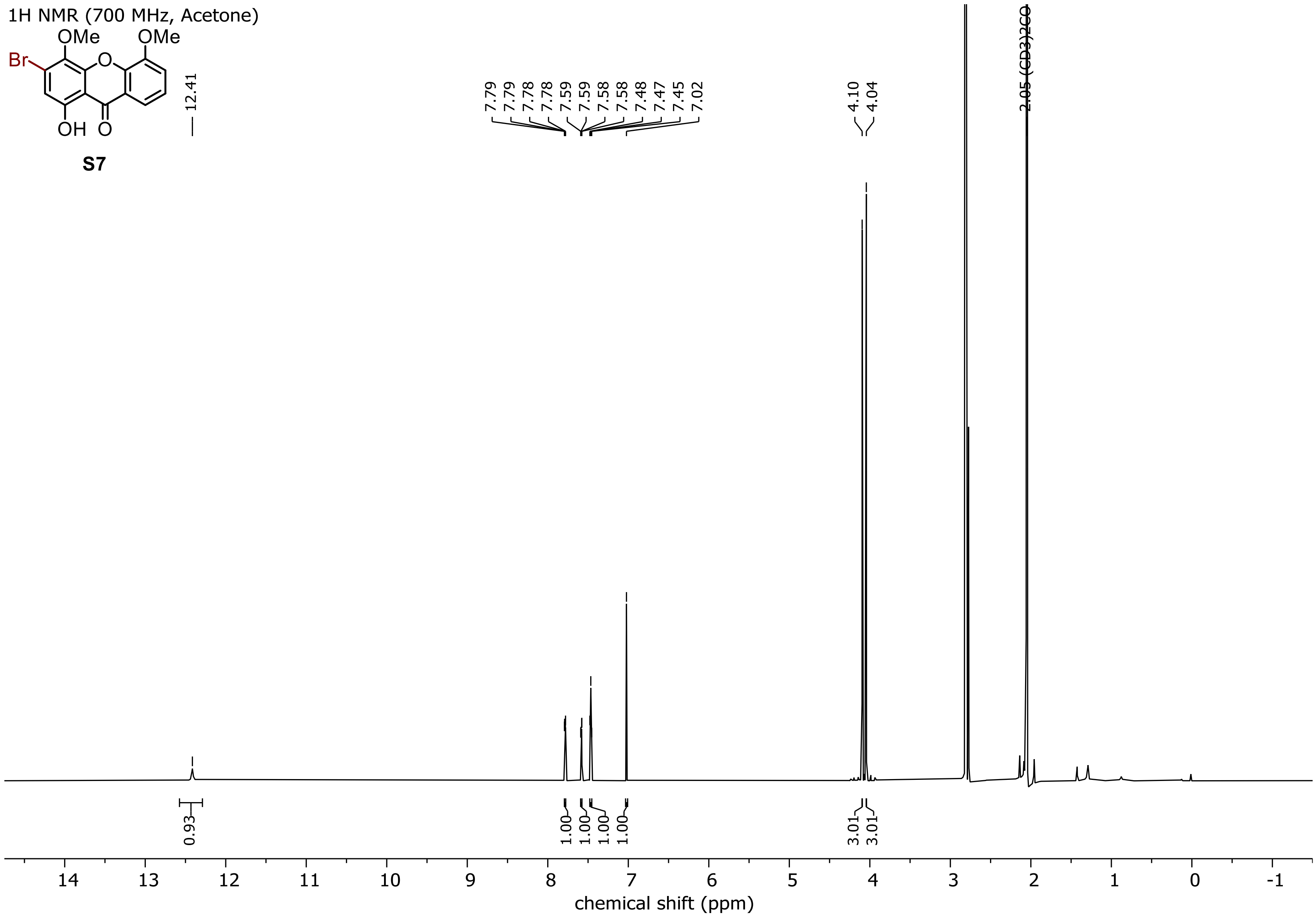
1H NMR (700 MHz, Acetone)
OMe
OMe
Br
O
OH
O
S7
12.41
7.79
7.79
7.78
7.78
7.59
7.59
7.58
7.58
7.48
7.47
7.45
7.02
4.10
4.04
2.05 (CD3)2CO
0.93
1.00
1.00
1.00
1.00
3.01
3.01
14
13
12
11
10
9
8
7
6
5
4
3
2
1
0
-1
chemical shift (ppm)

13C NMR (176 MHz, Acetone)

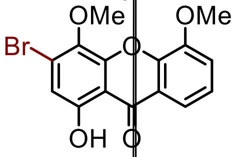

S7

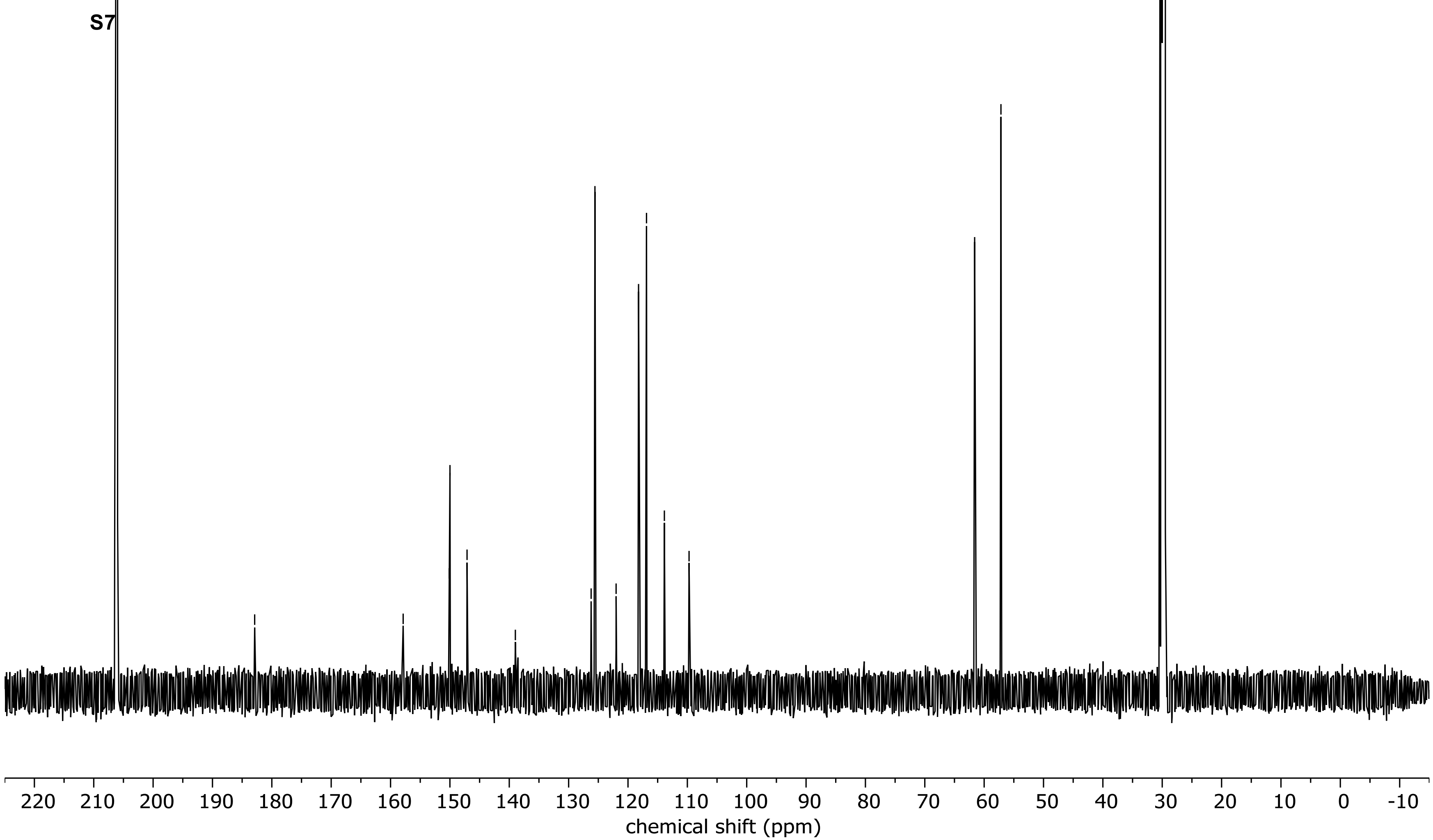

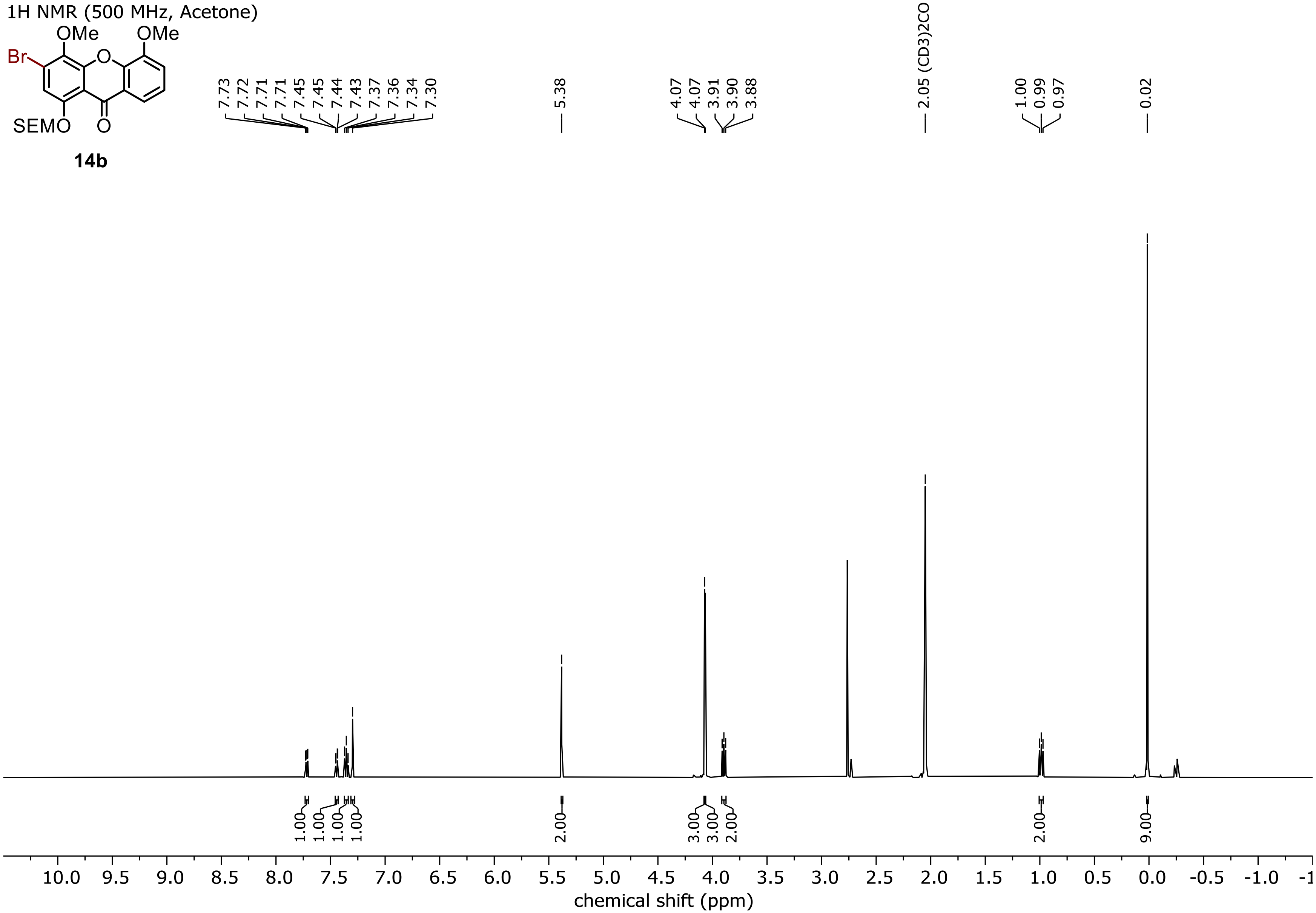

1H NMR (500 MHz, Acetone)
OMe
OMe
Br
O
SEMO
O
14b
7.73
7.72
7.71
7.71
7.45
7.45
7.44
7.43
7.37
7.36
7.34
7.30
5.38
4.07
4.07
3.91
3.90
3.88
2.05 (CD3)2CO
1.00
0.99
0.97
0.02
1.00
1.00
1.00
1.00
2.00
3.00
3.00
2.00
2.00
9.00
10.0 9.5 9.0 8.5 8.0 7.5 7.0 6.5 6.0 5.5 5.0 4.5 4.0 3.5 3.0 2.5 2.0 1.5 1.0 0.5 0.0 -0.5 -1.0
chemical shift (ppm)

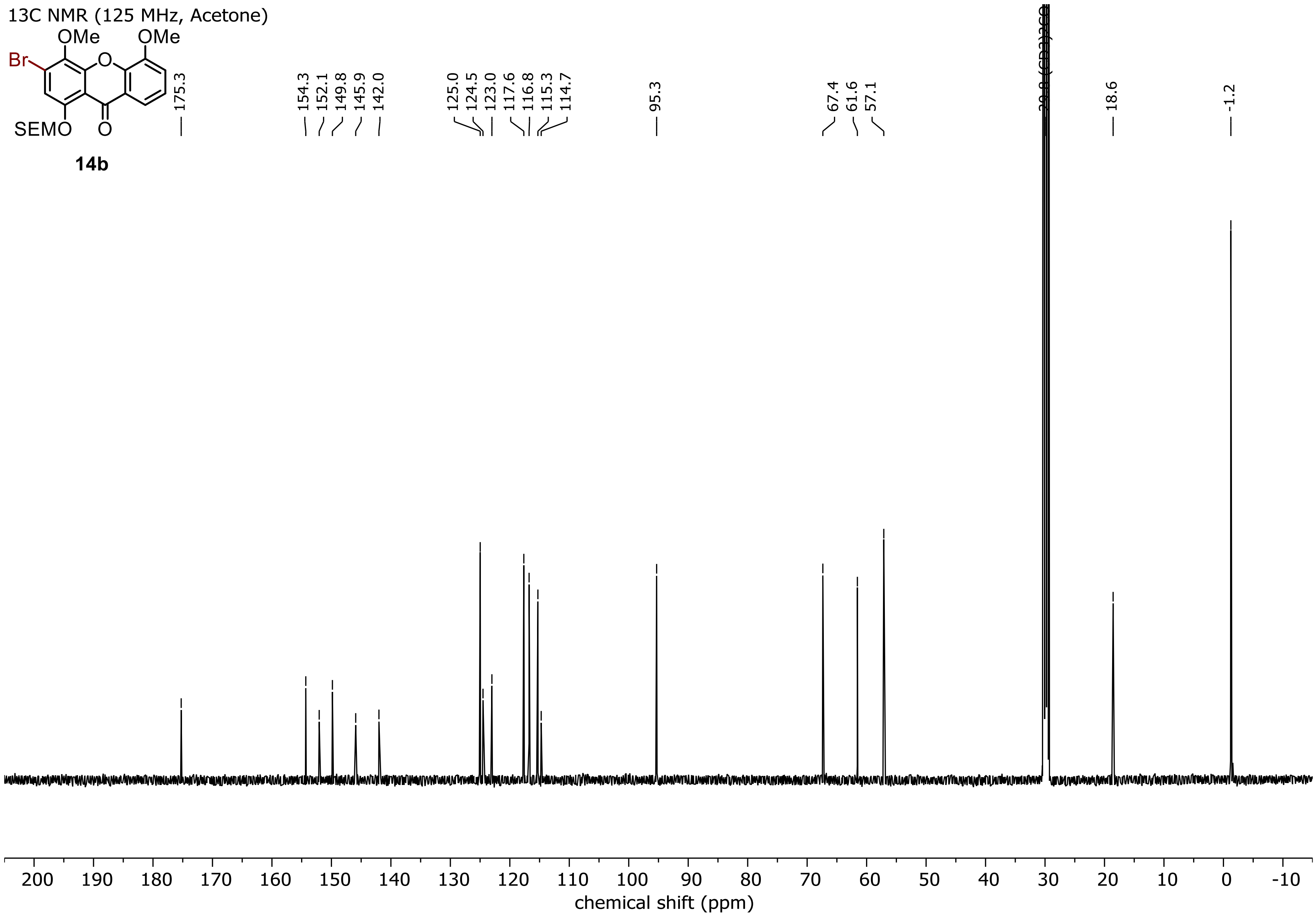

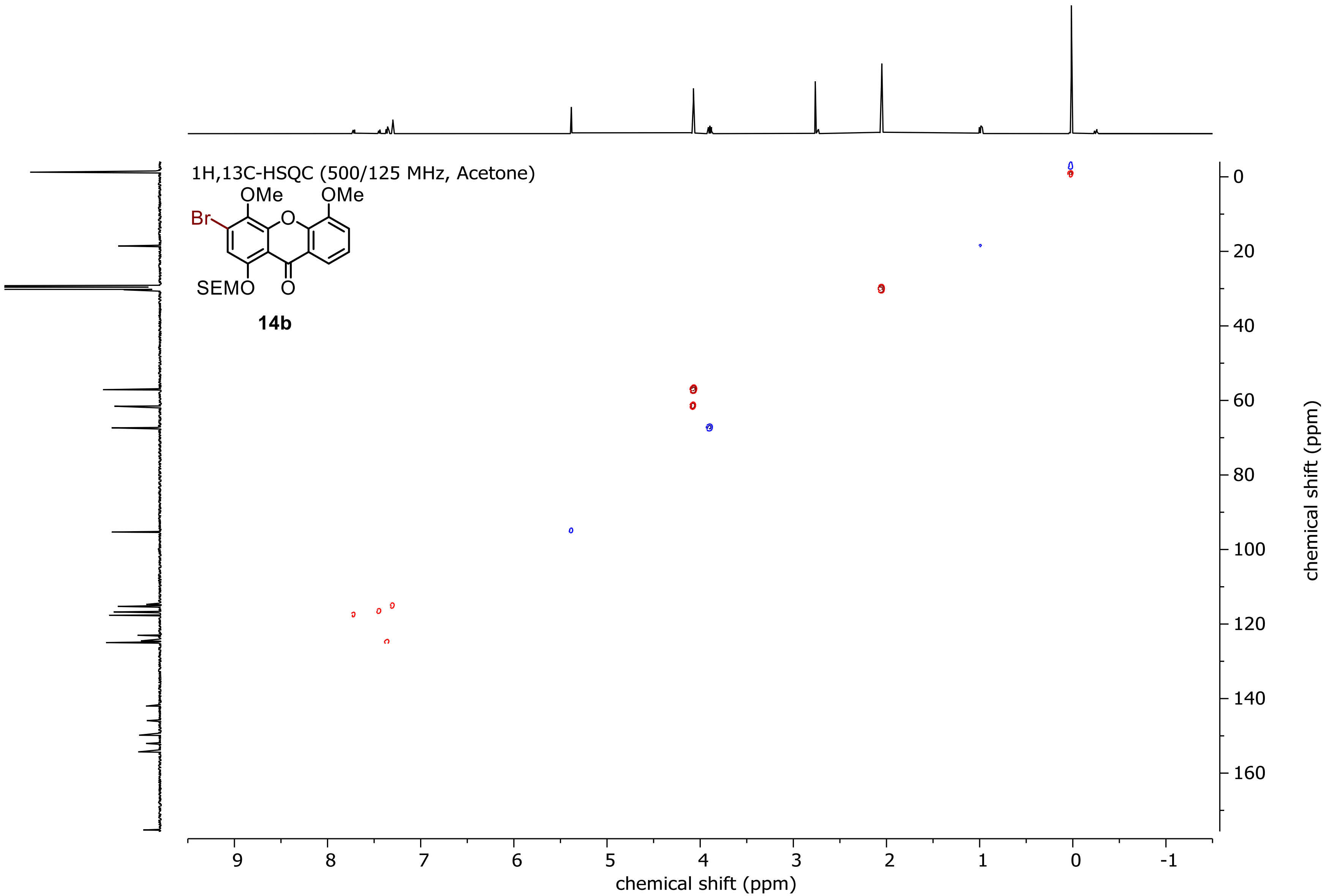
1H,13C-HSQC (500/125 MHz, Acetone)
OMe
OMe
Br
O
SEMO
O
14b
chemical shift (ppm)
chemical shift (ppm)

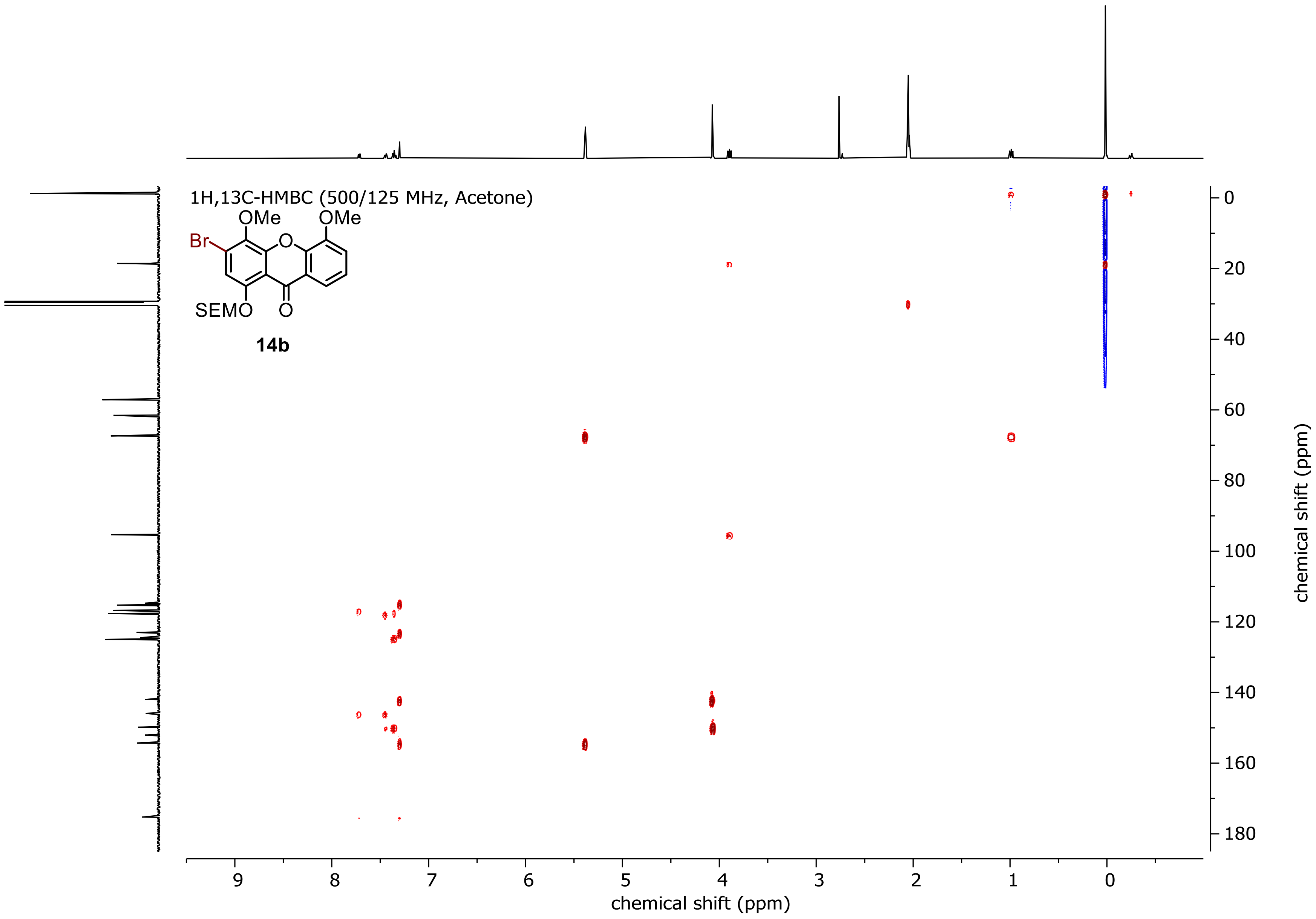

1H,13C-HMBC (500/125 MHz, Acetone)
OMe
OMe
Br
O
SEMO
O
14b
chemical shift (ppm)
chemical shift (ppm)

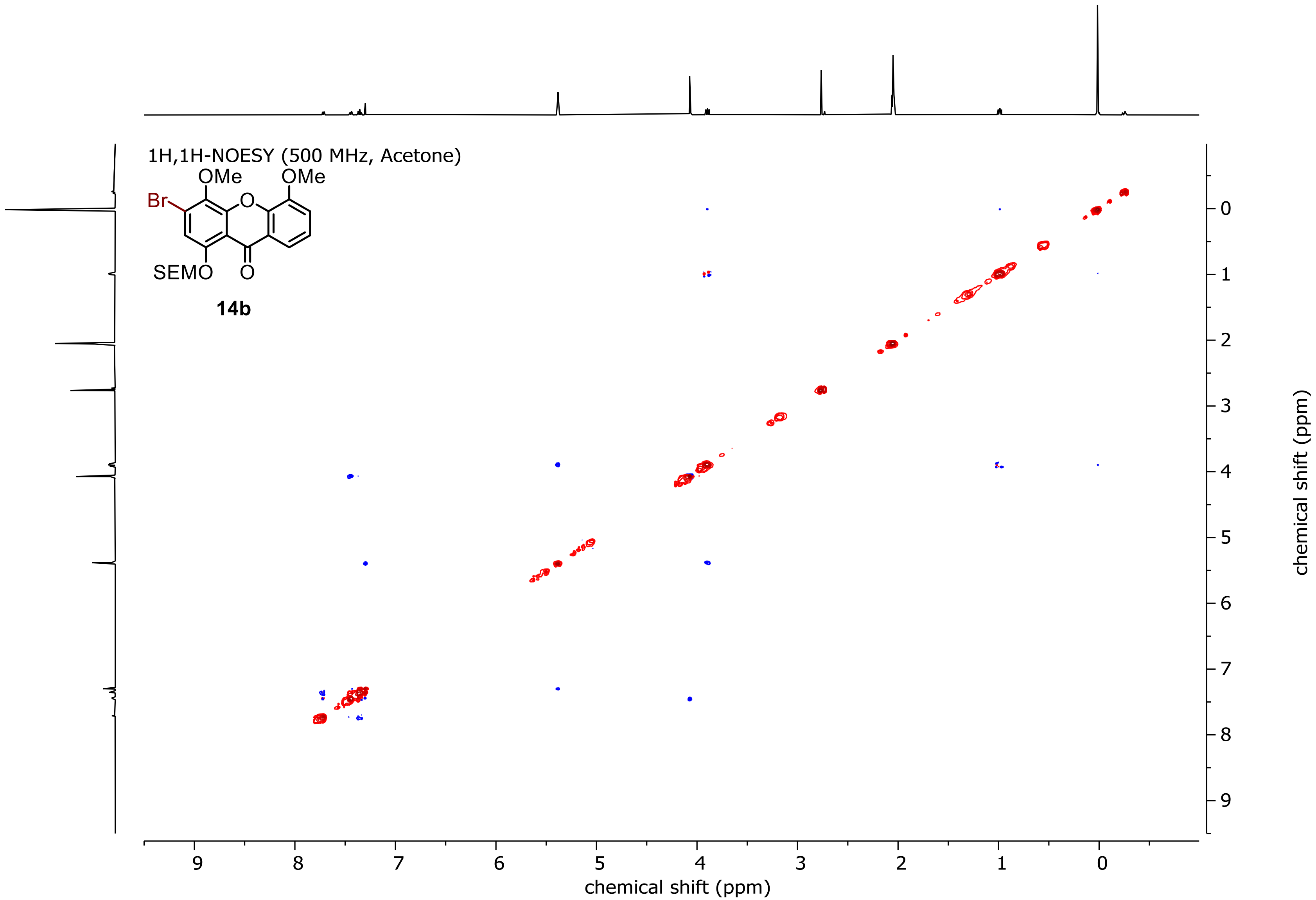

1H,1H-NOESY (500 MHz, Acetone)
OMe
OMe
Br
O
SEMO
O
14b
9
8
7
6
5
4
3
2
1
0
chemical shift (ppm)
chemical shift (ppm)

1H NMR (500 MHz, Acetone)

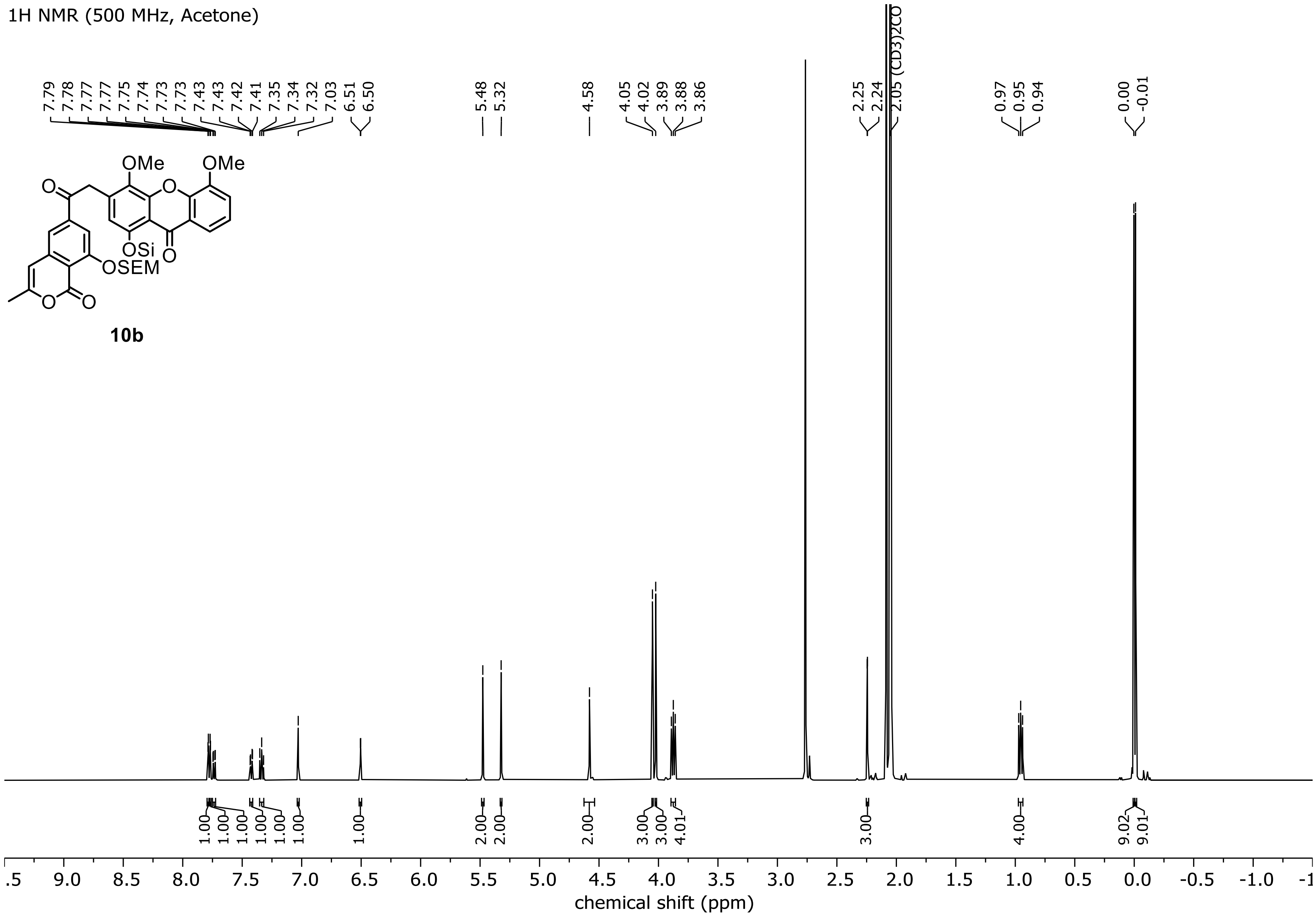

13C NMR (125 MHz, Acetone)

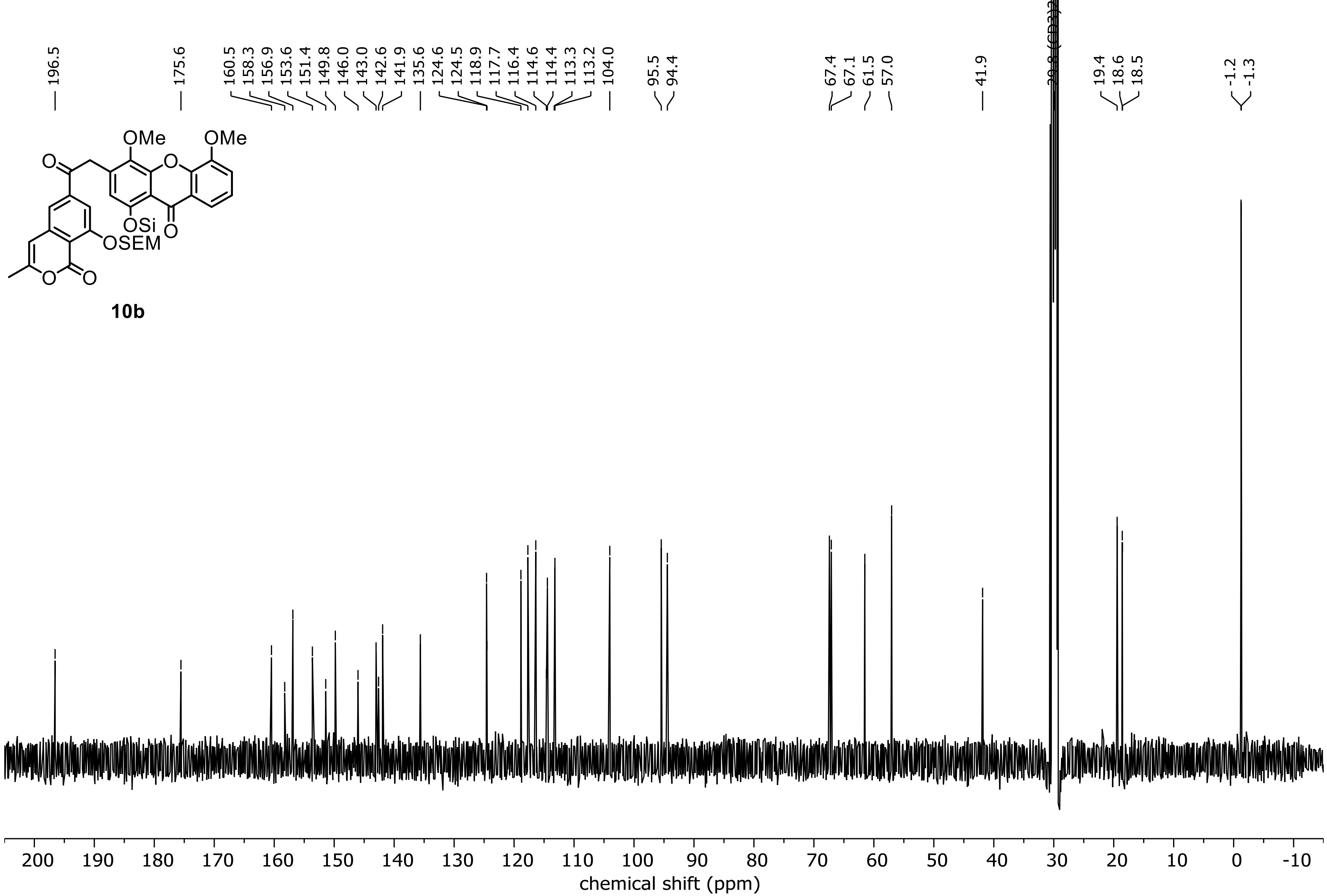

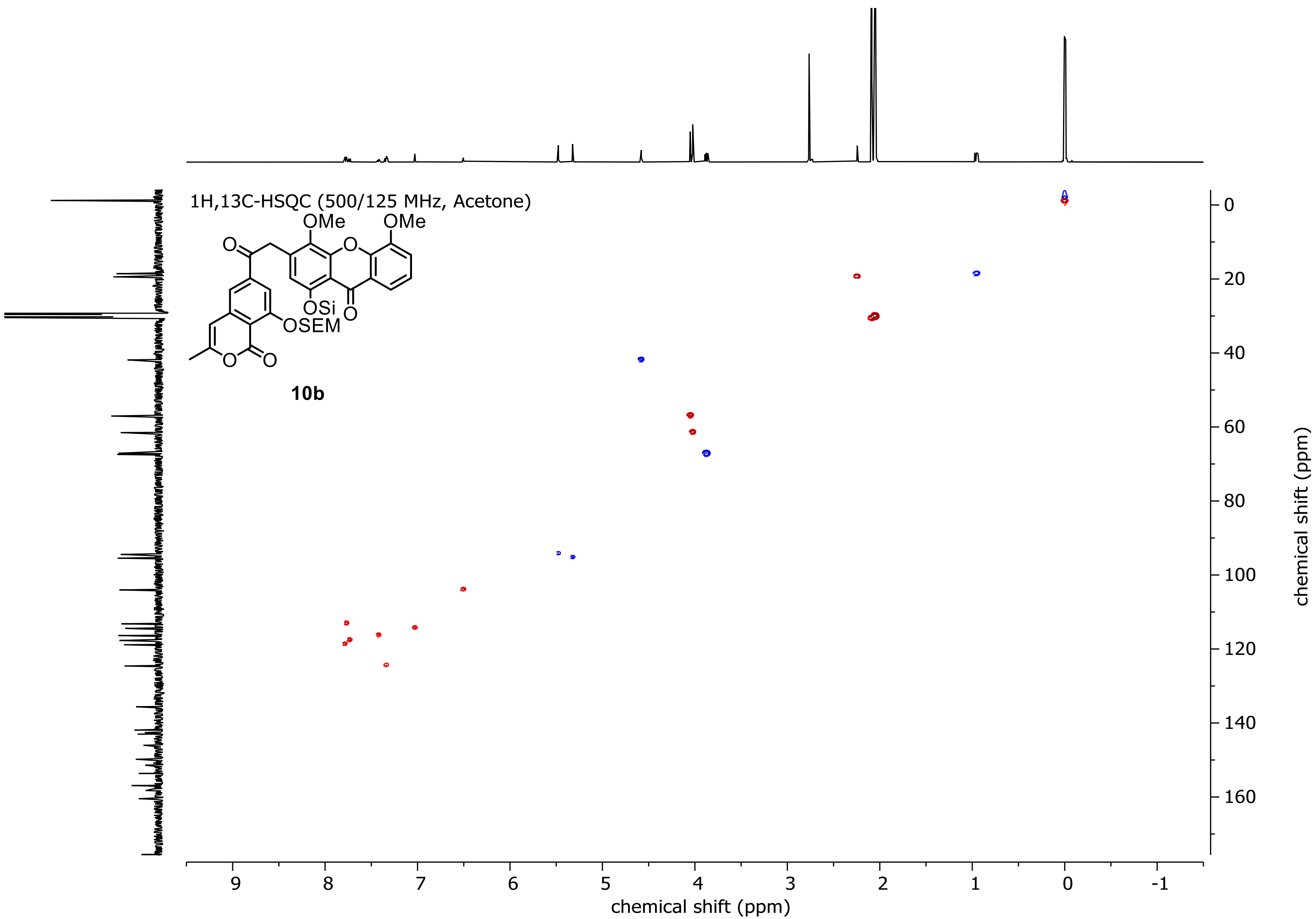
1H,13C-HSQC (500/125 MHz, Acetone)
OMe
OMe
O
O
OSi
O
OSEM
O
O
10b
chemical shift (ppm)
chemical shift (ppm)

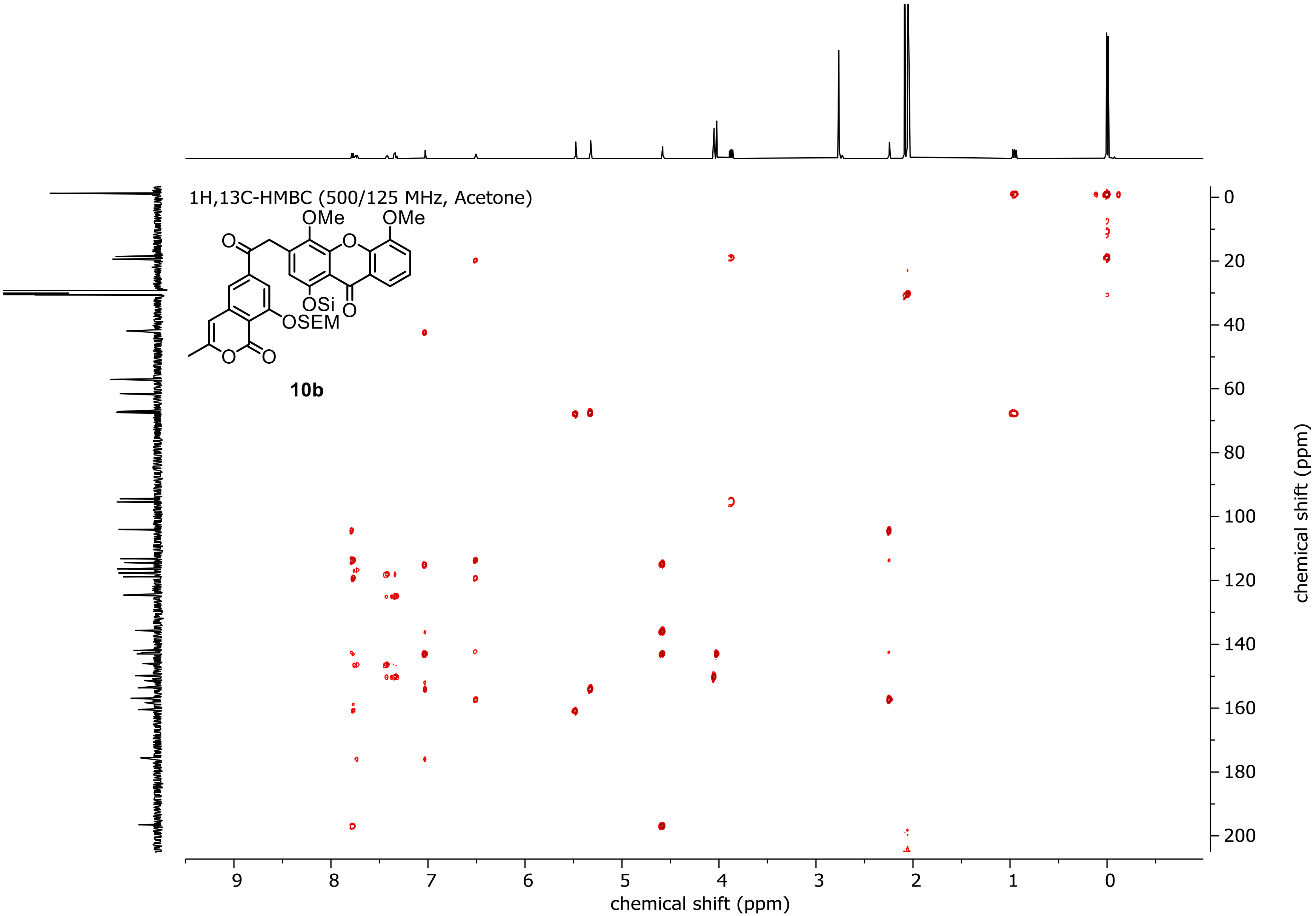
1H,13C-HMBC (500/125 MHz, Acetone)
OMe
OMe
O
OSi
OSEM
O
O
O
10b
0
20
40
60
80
100
120
140
160
180
200
chemical shift (ppm)
9
8
7
6
5
4
3
2
1
0
chemical shift (ppm)

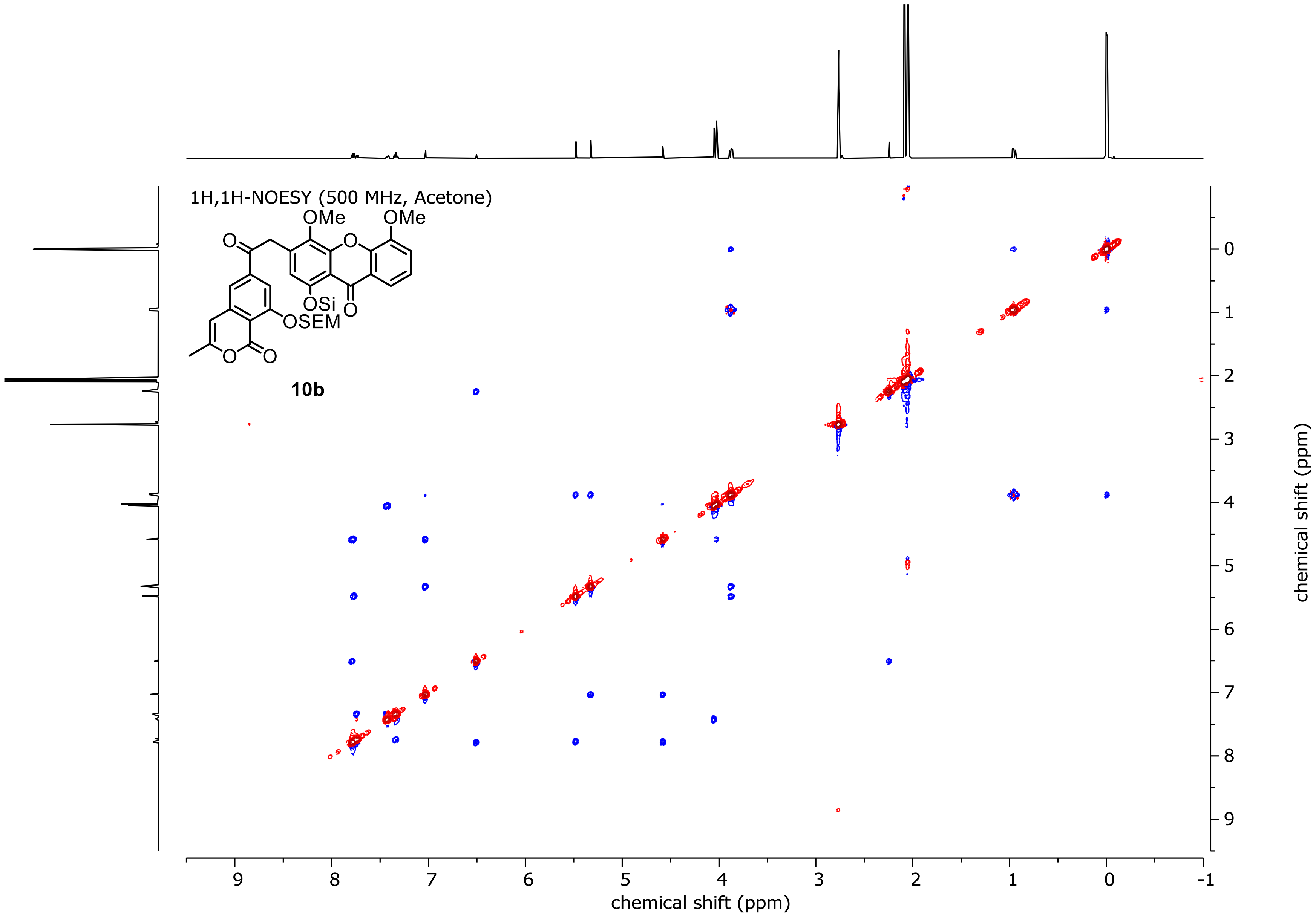
1H,1H-NOESY (500 MHz, Acetone)
OMe
OMe
O
O
OSi
O
OSEM
O
O
10b
chemical shift (ppm)
chemical shift (ppm)
9
8
7
6
5
4
3
2
1
0
-1

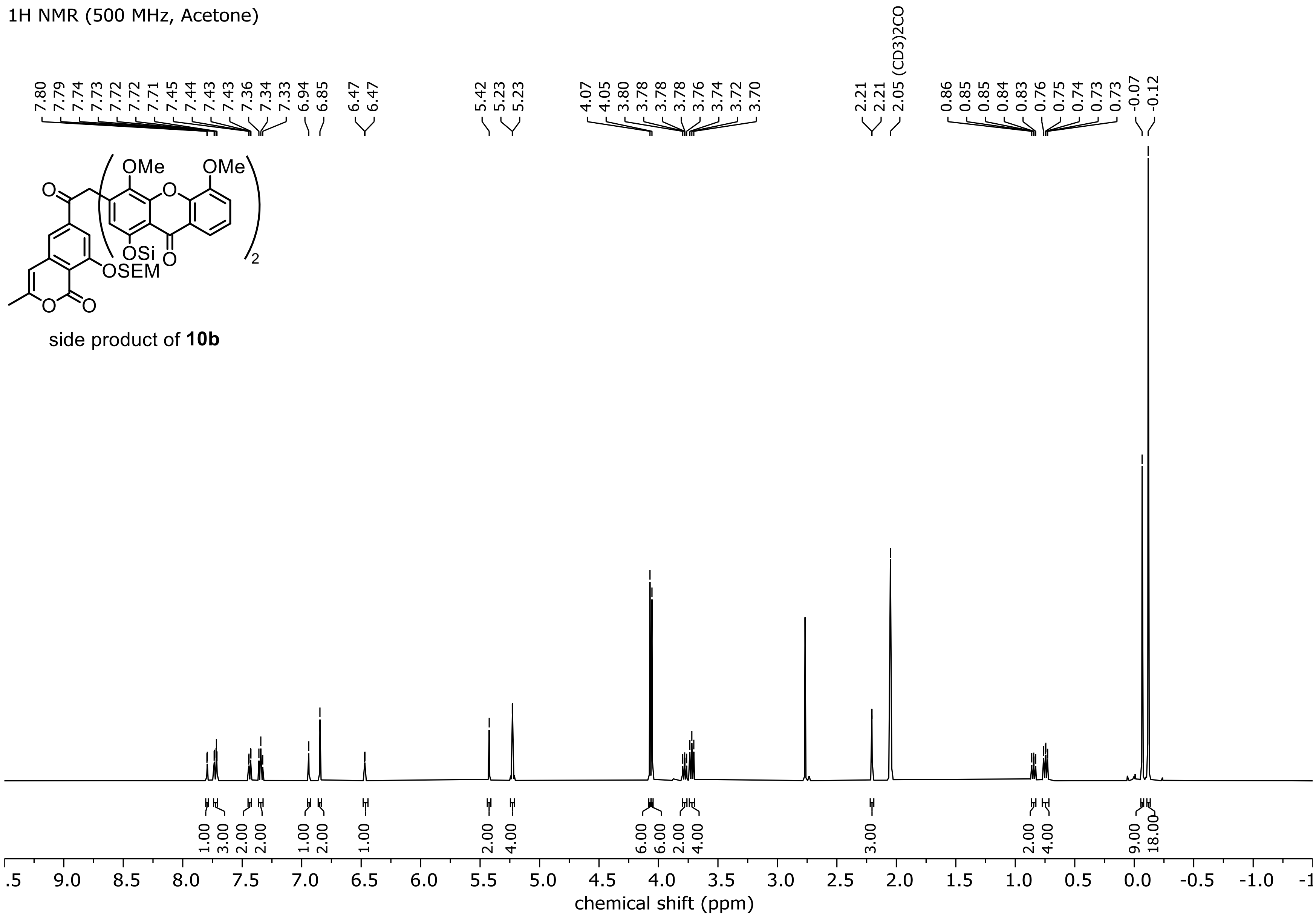
1H NMR (500 MHz, Acetone)
OMe
OMe
OSi
OSEM
2
side product of 10b
(CD3)2CO
chemical shift (ppm)

13C NMR (125 MHz, Acetone)

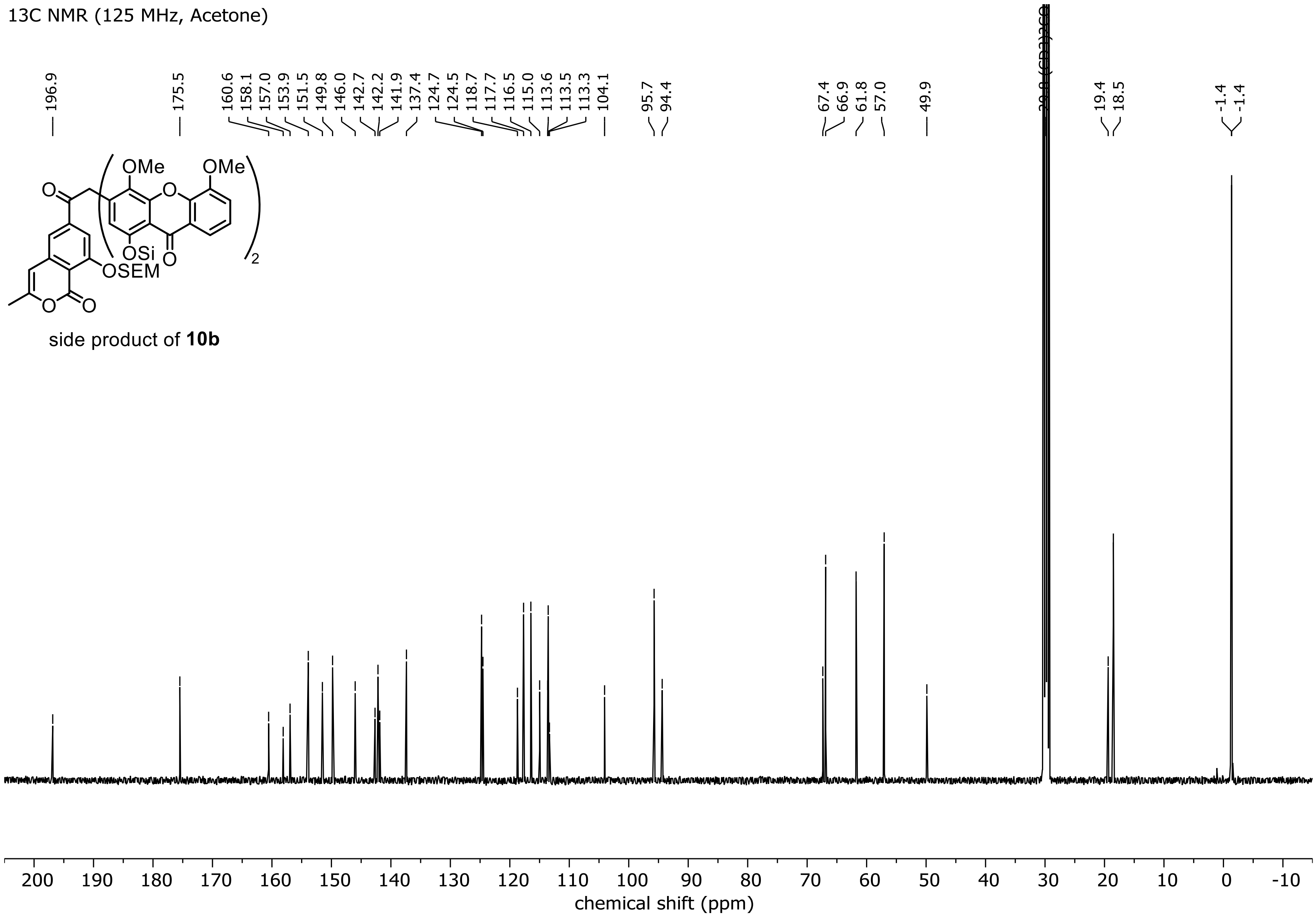

side product of **10b**

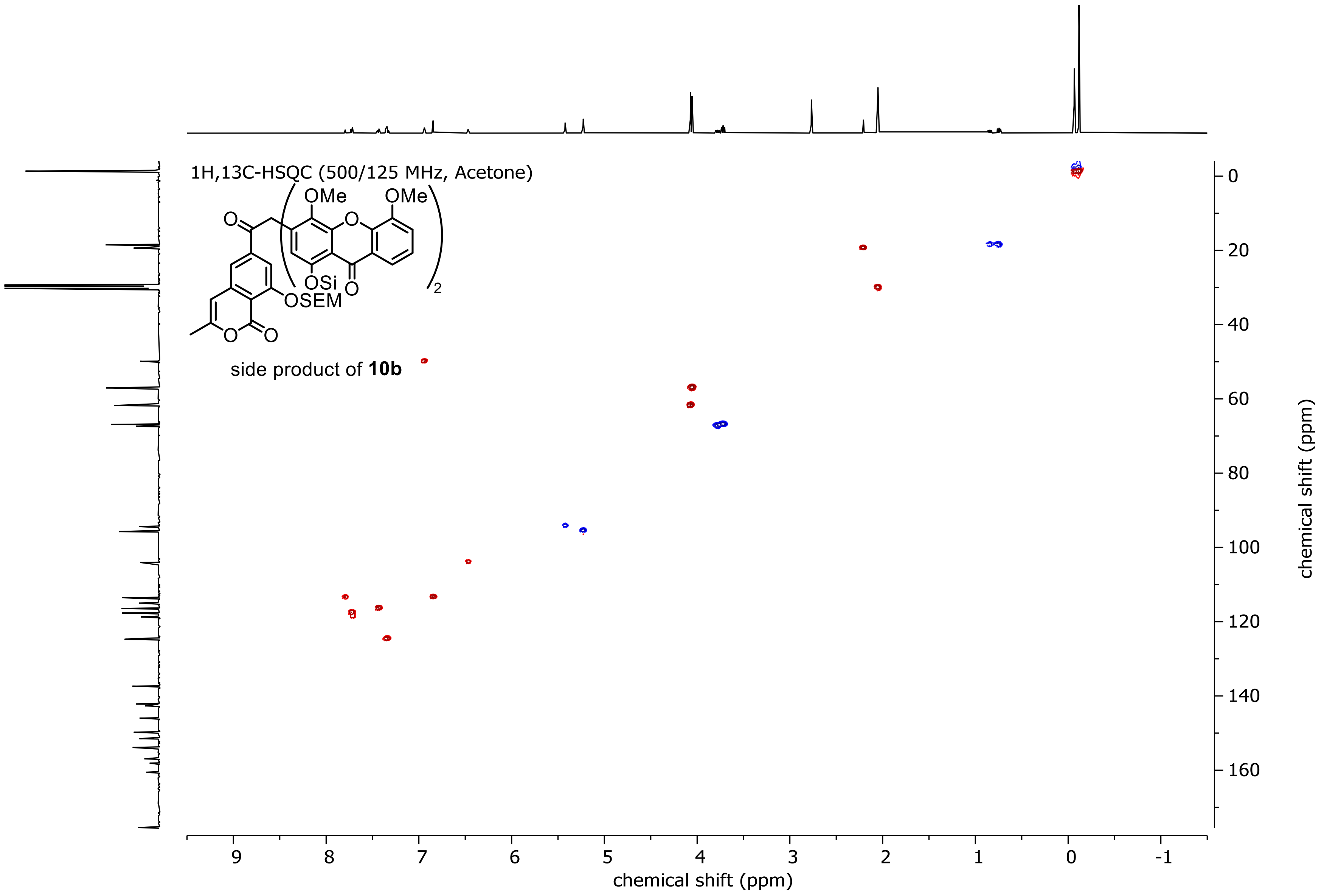
1H,13C-HSQC (500/125 MHz, Acetone)
OMe
OMe
O
O
OSi
O
OSEM
O
O
2
side product of **10b**
chemical shift (ppm)
chemical shift (ppm)

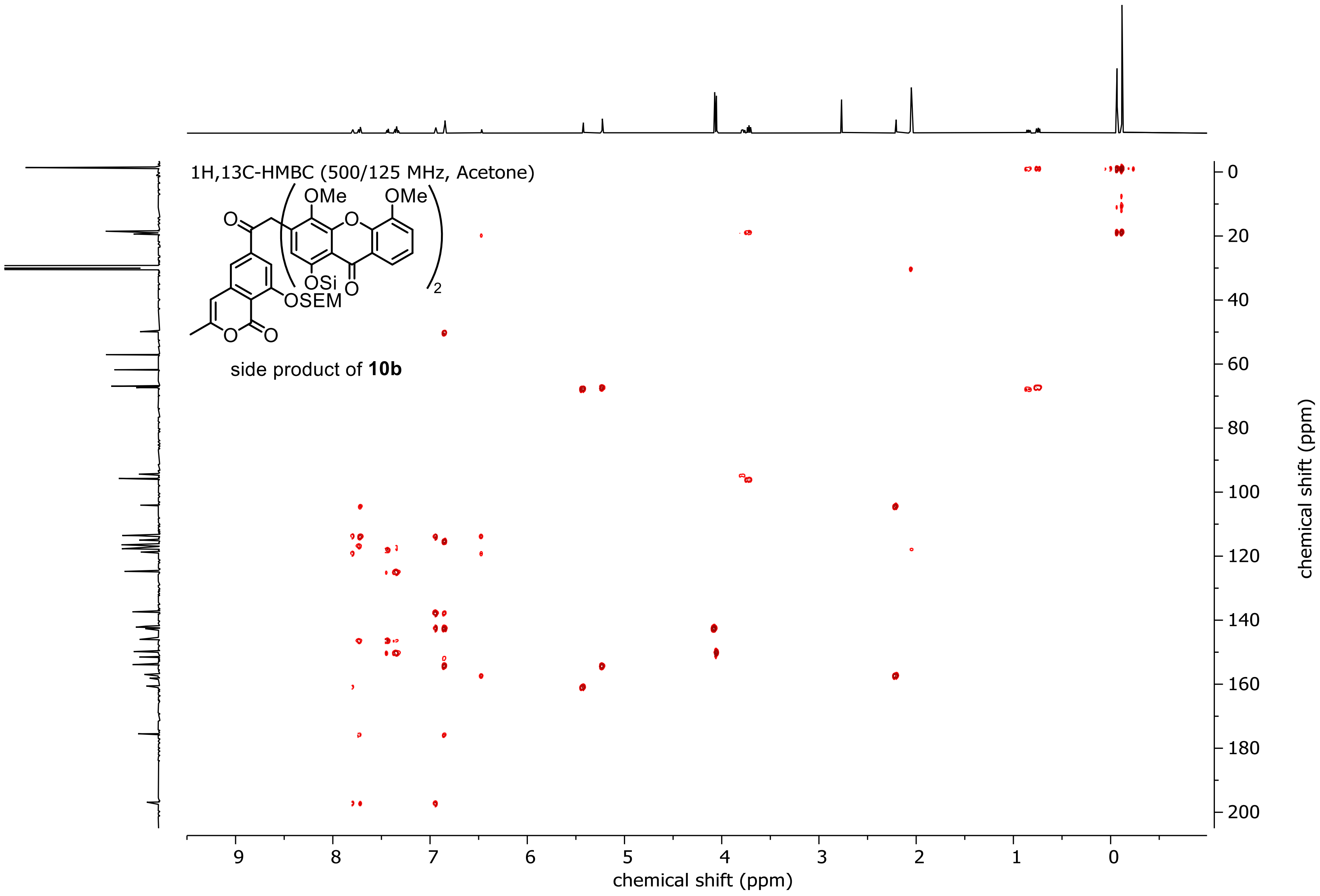
1H,13C-HMBC (500/125 MHz, Acetone)
OMe
OMe
O
O
OSi
O
OSEM
O
O
2
side product of **10b**
9
8
7
6
5
4
3
2
1
0
chemical shift (ppm)
0
20
40
60
80
100
120
140
160
180
200
chemical shift (ppm)

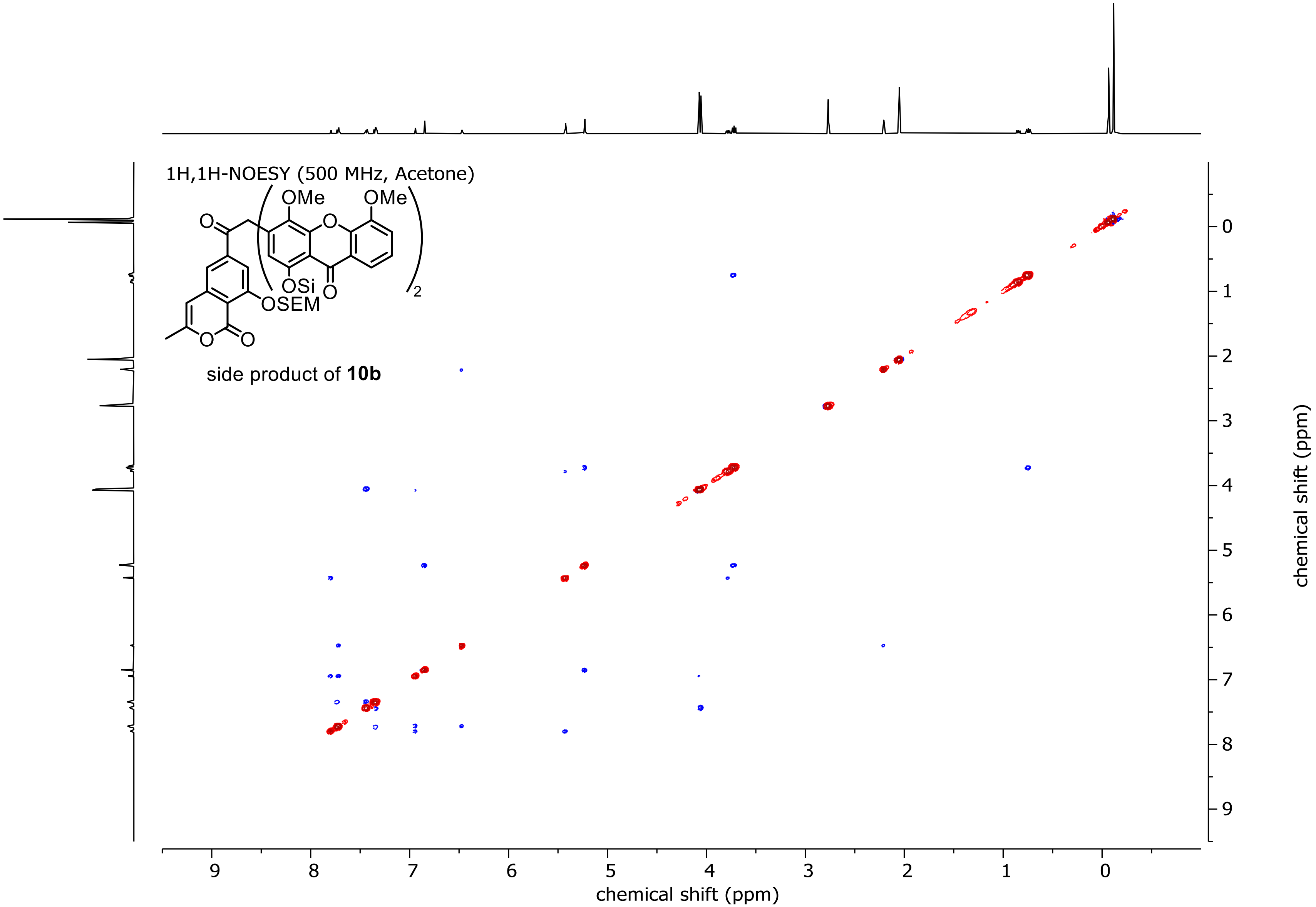
1H,1H-NOESY (500 MHz, Acetone)
OMe
OMe
O
O
OSi
O
OSEM
O
O
2
side product of **10b**
chemical shift (ppm)
chemical shift (ppm)

1H NMR (700 MHz, Acetone)

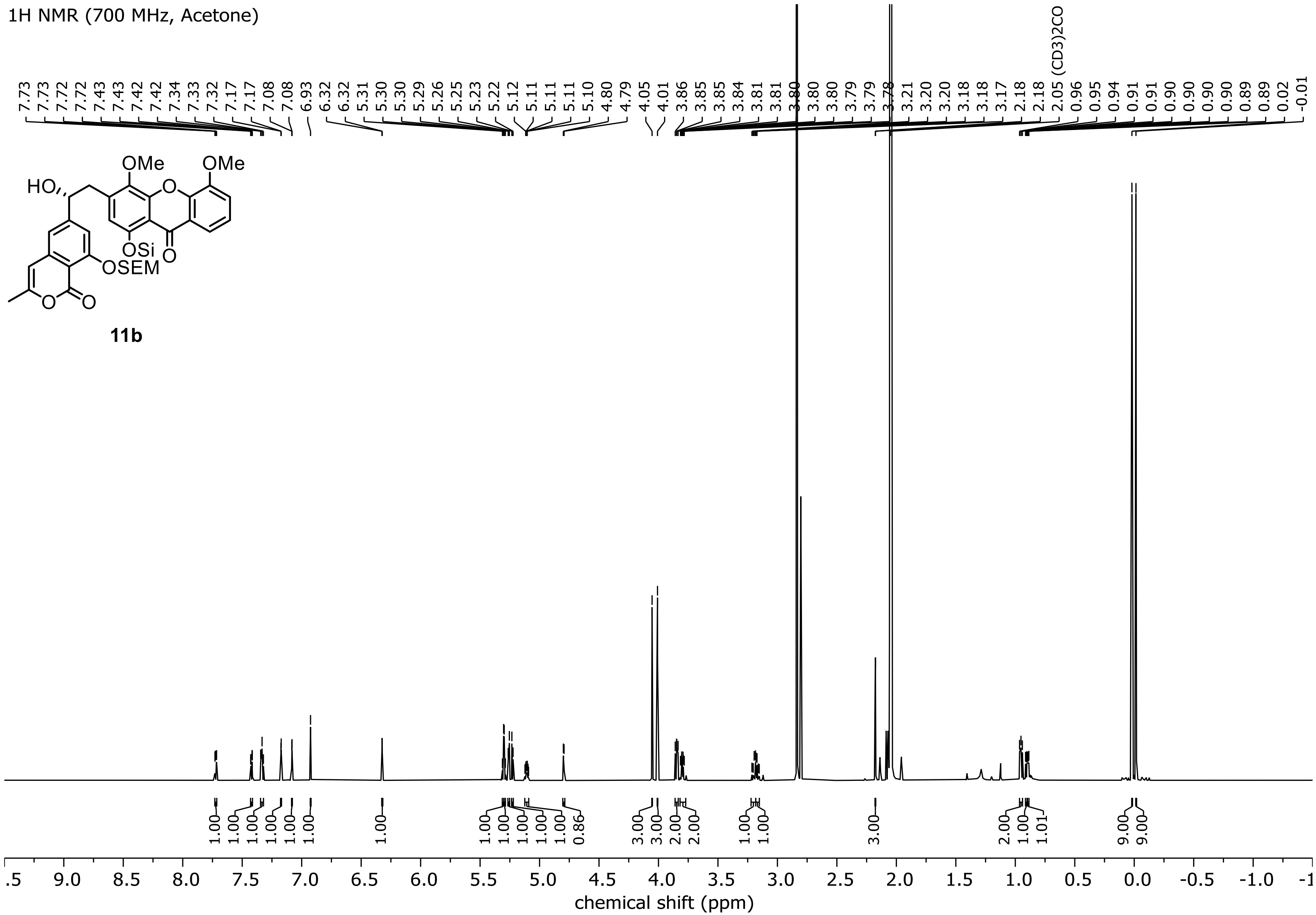

11b

13C NMR (176 MHz, Acetone)

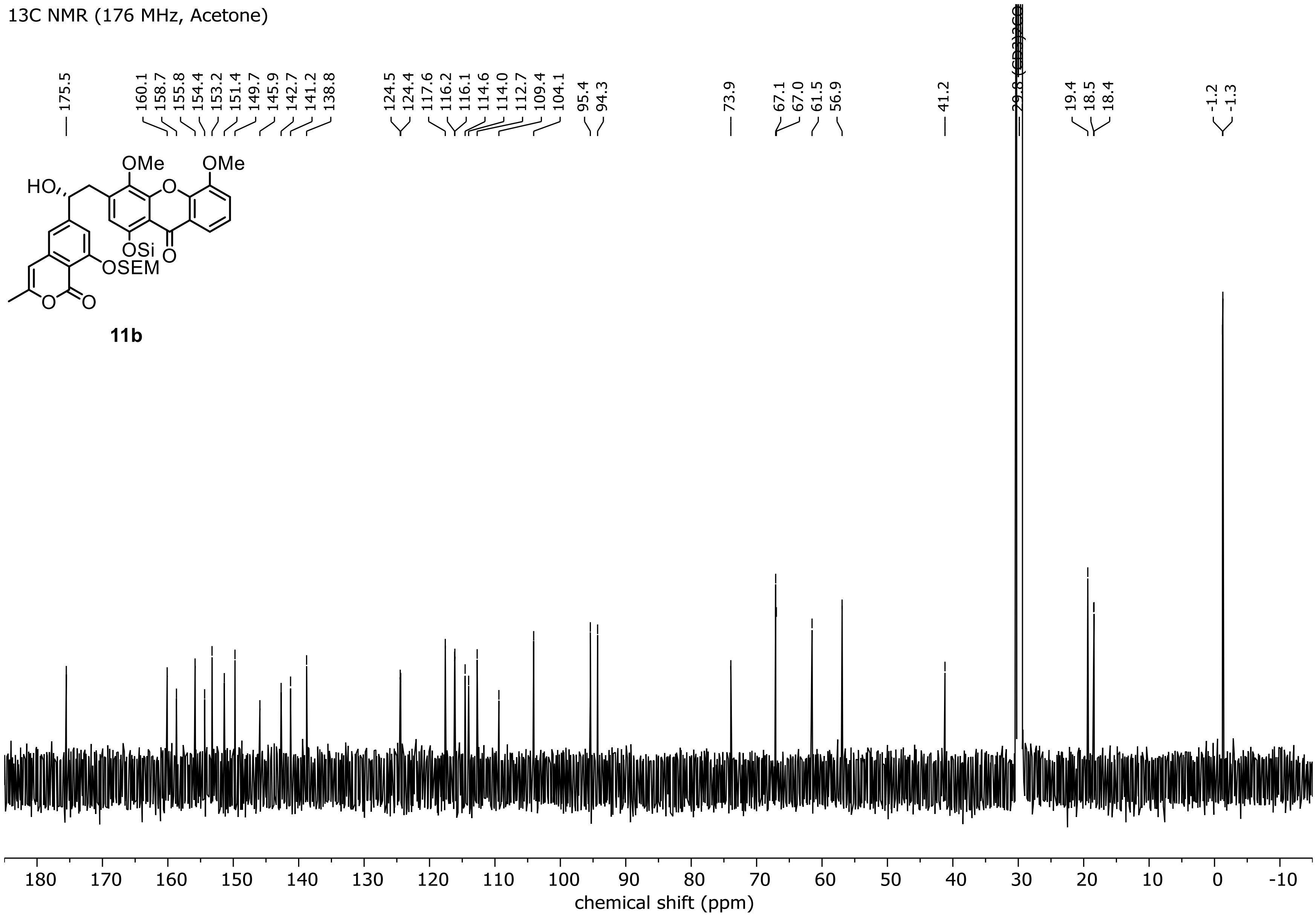

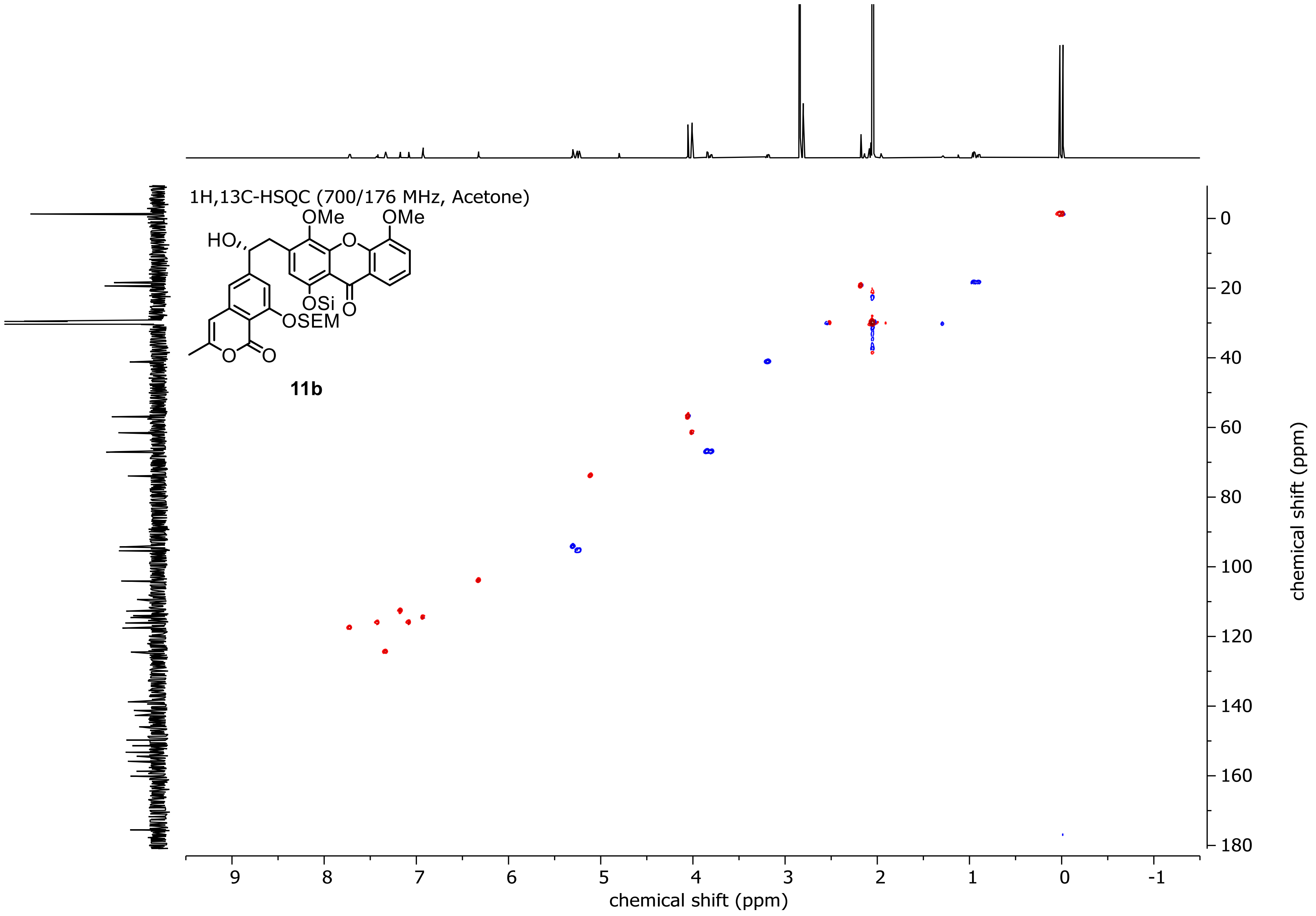

1H,13C-HSQC (700/176 MHz, Acetone)
OMe
OMe
HO
O
OSi
O
OSEM
O
O
11b
chemical shift (ppm)
chemical shift (ppm)
9
8
7
6
5
4
3
2
1
0
-1
0
20
40
60
80
100
120
140
160
180

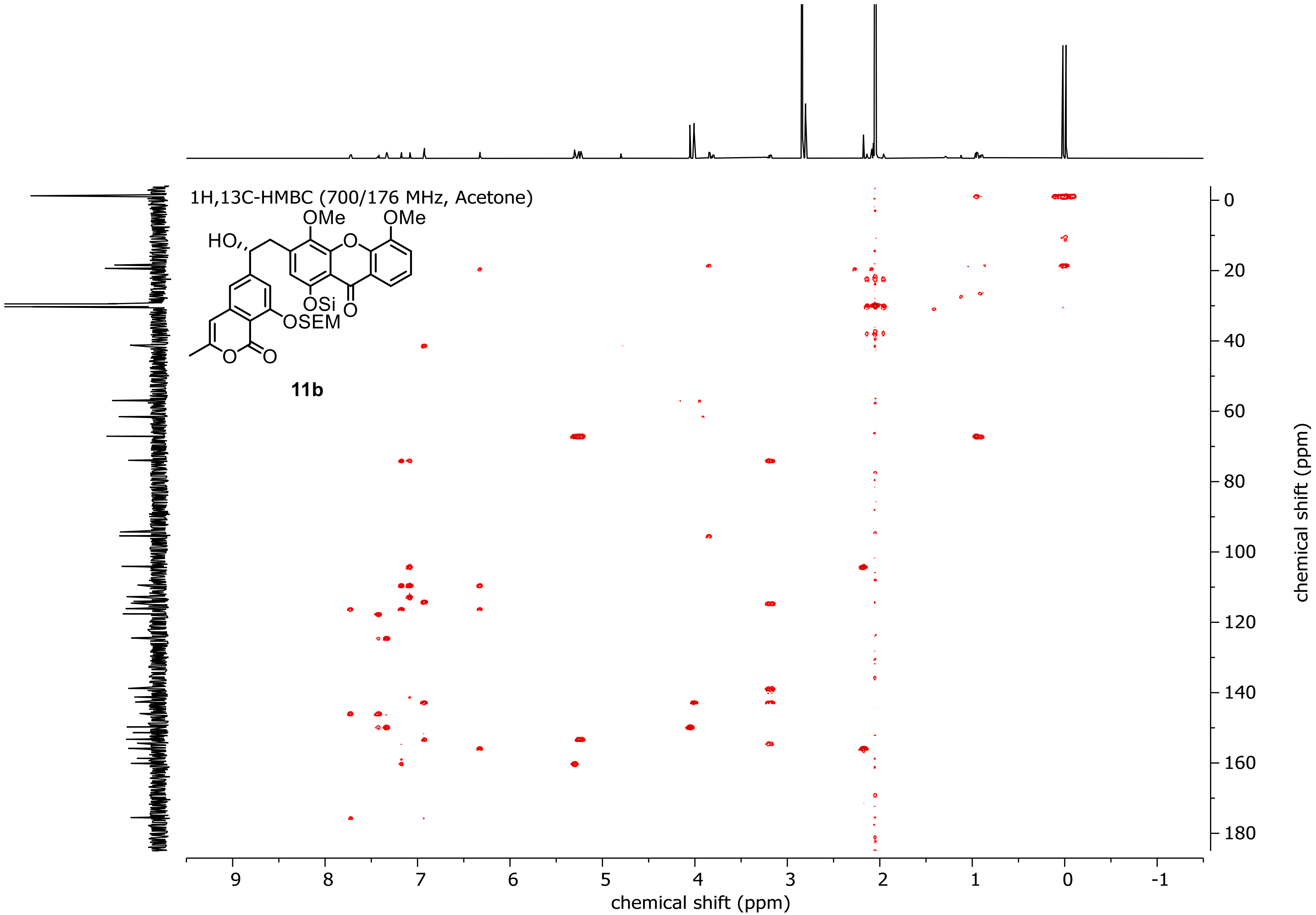

1H,13C-HMBC (700/176 MHz, Acetone)
OMe
OMe
HO
O
OSi
O
OSEM
O
O
11b
chemical shift (ppm)
chemical shift (ppm)

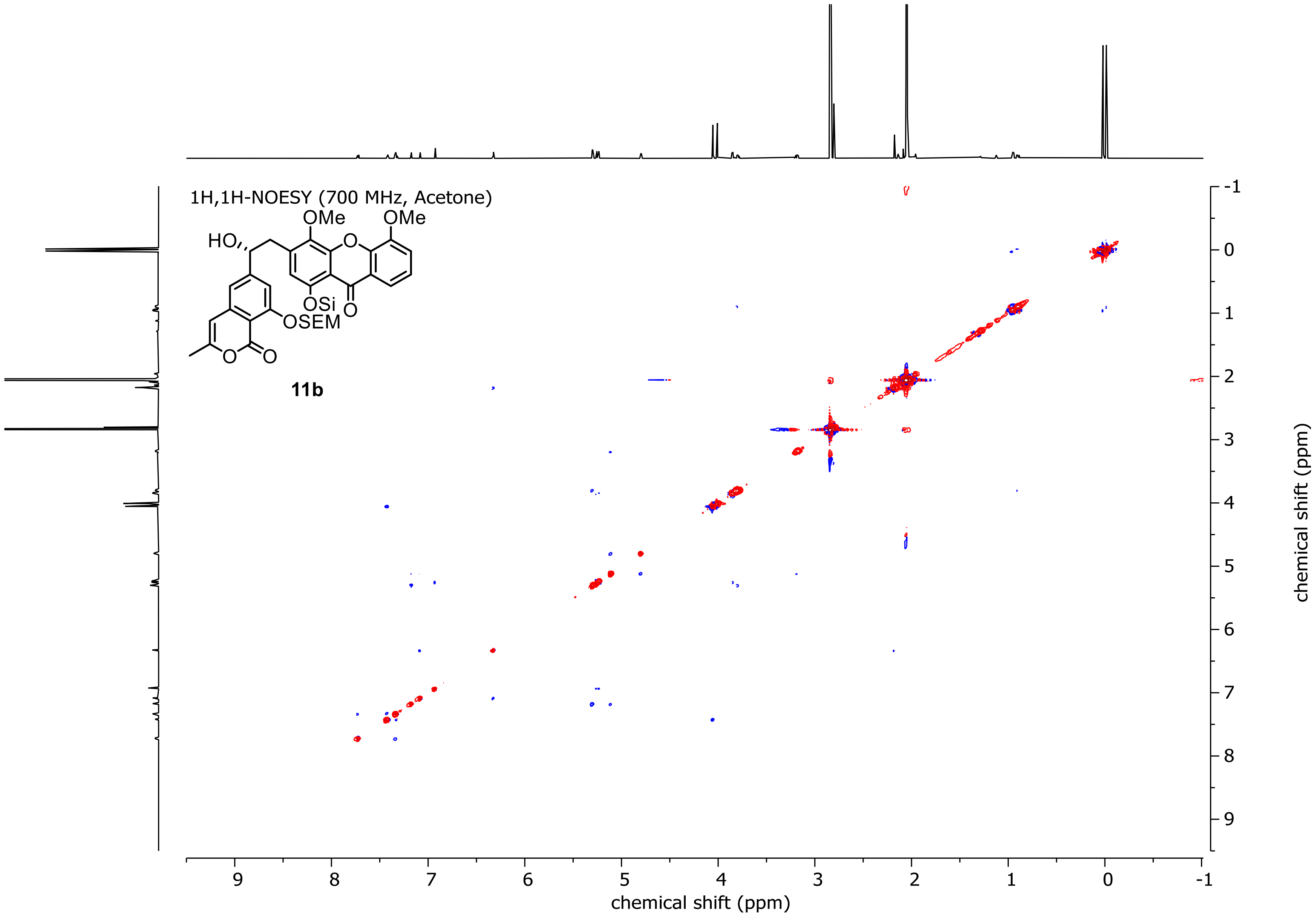
1H,1H-NOESY (700 MHz, Acetone)
OMe
OMe
HO
O
OSi
O
OSEM
O
O
11b
chemical shift (ppm)
chemical shift (ppm)

1H NMR (500 MHz, Acetone)

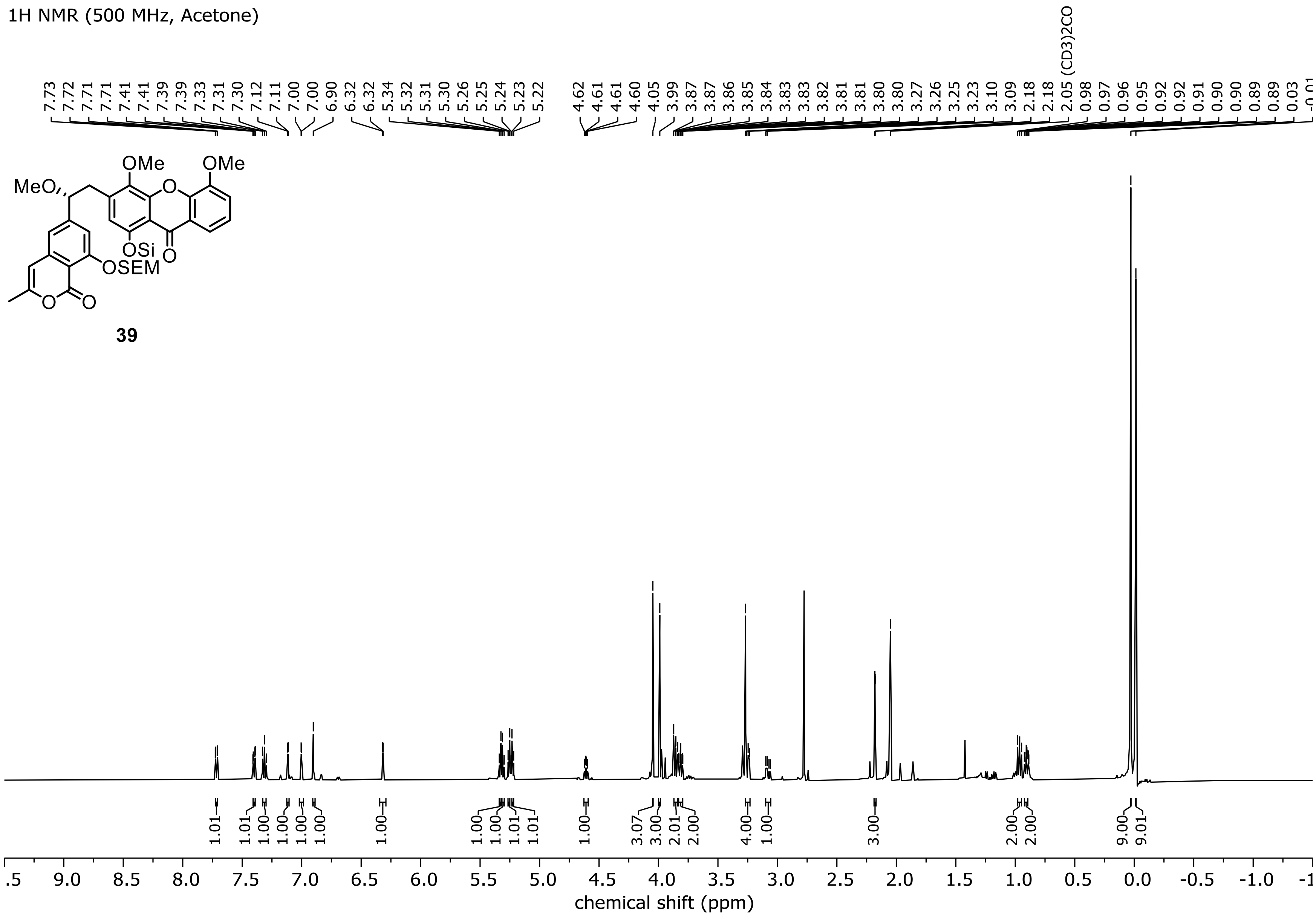

39

13C NMR (125 MHz, Acetone)

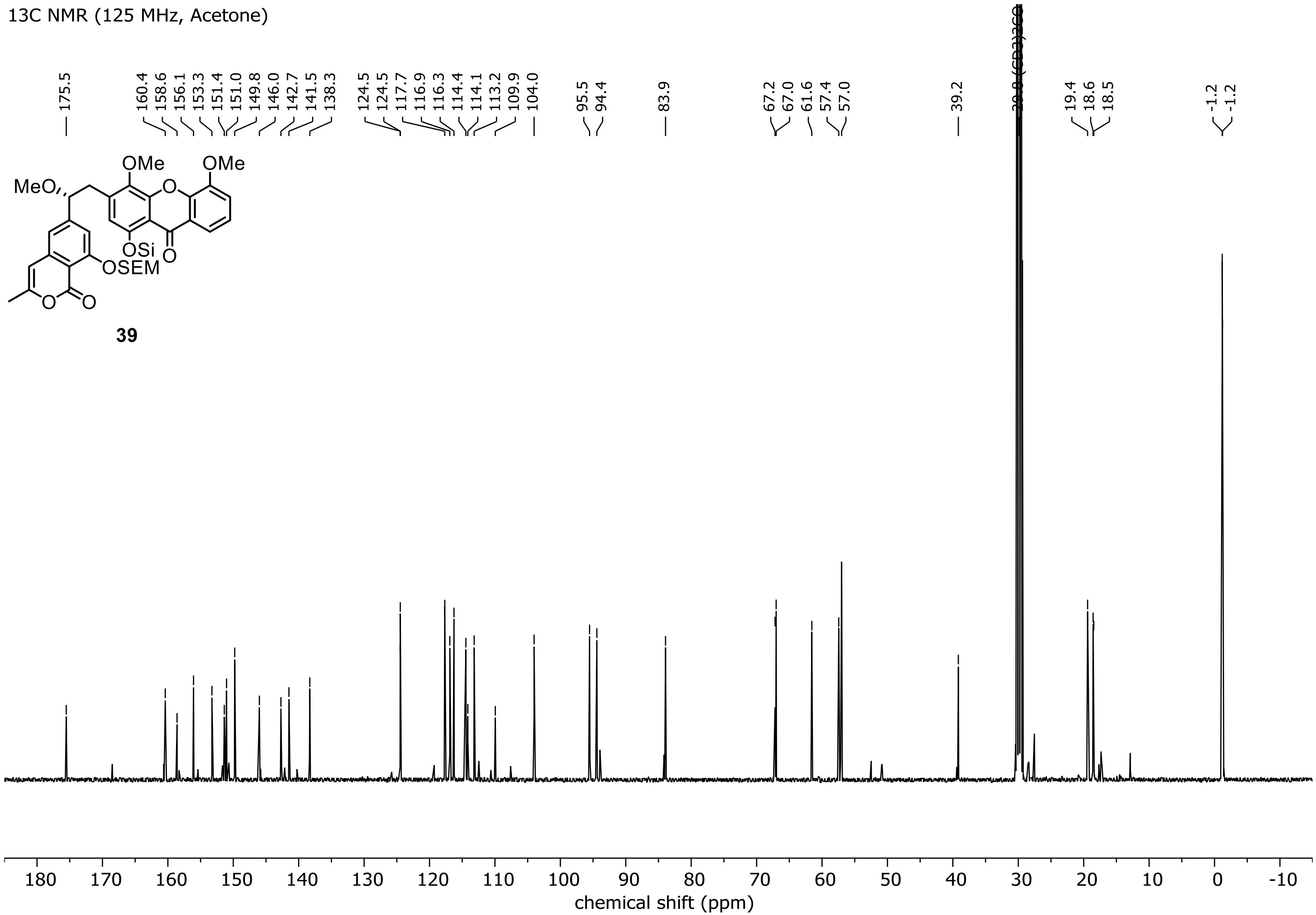

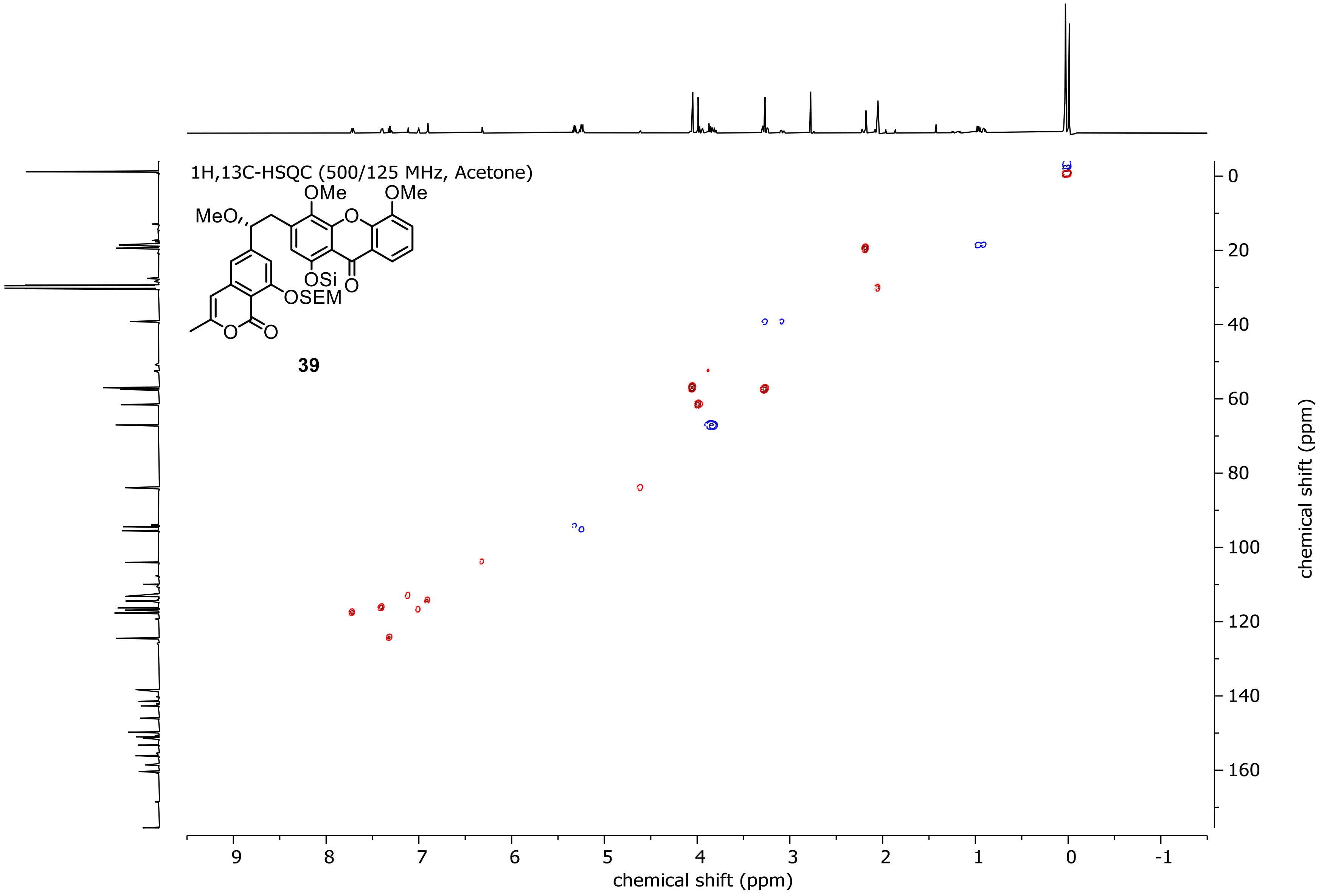
1H,13C-HSQC (500/125 MHz, Acetone)
OMe
OMe
MeO
O
OSi
O
OSEM
O
O
39
chemical shift (ppm)
chemical shift (ppm)

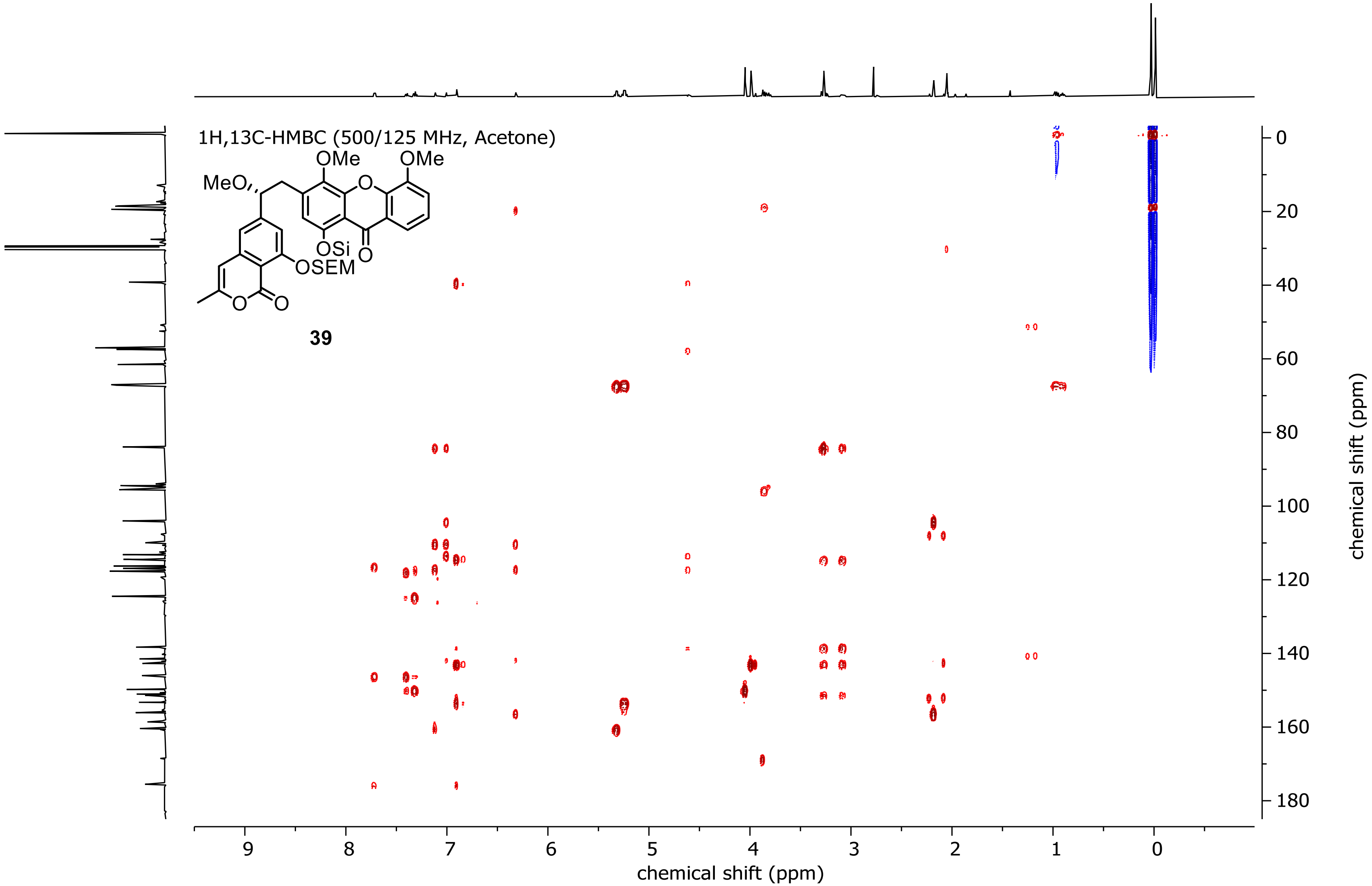
1H,13C-HMBC (500/125 MHz, Acetone)
OMe
OMe
MeO
OSi
OSEM
O
O
O
O
39
9
8
7
6
5
4
3
2
1
0
chemical shift (ppm)
0
20
40
60
80
100
120
140
160
180
chemical shift (ppm)

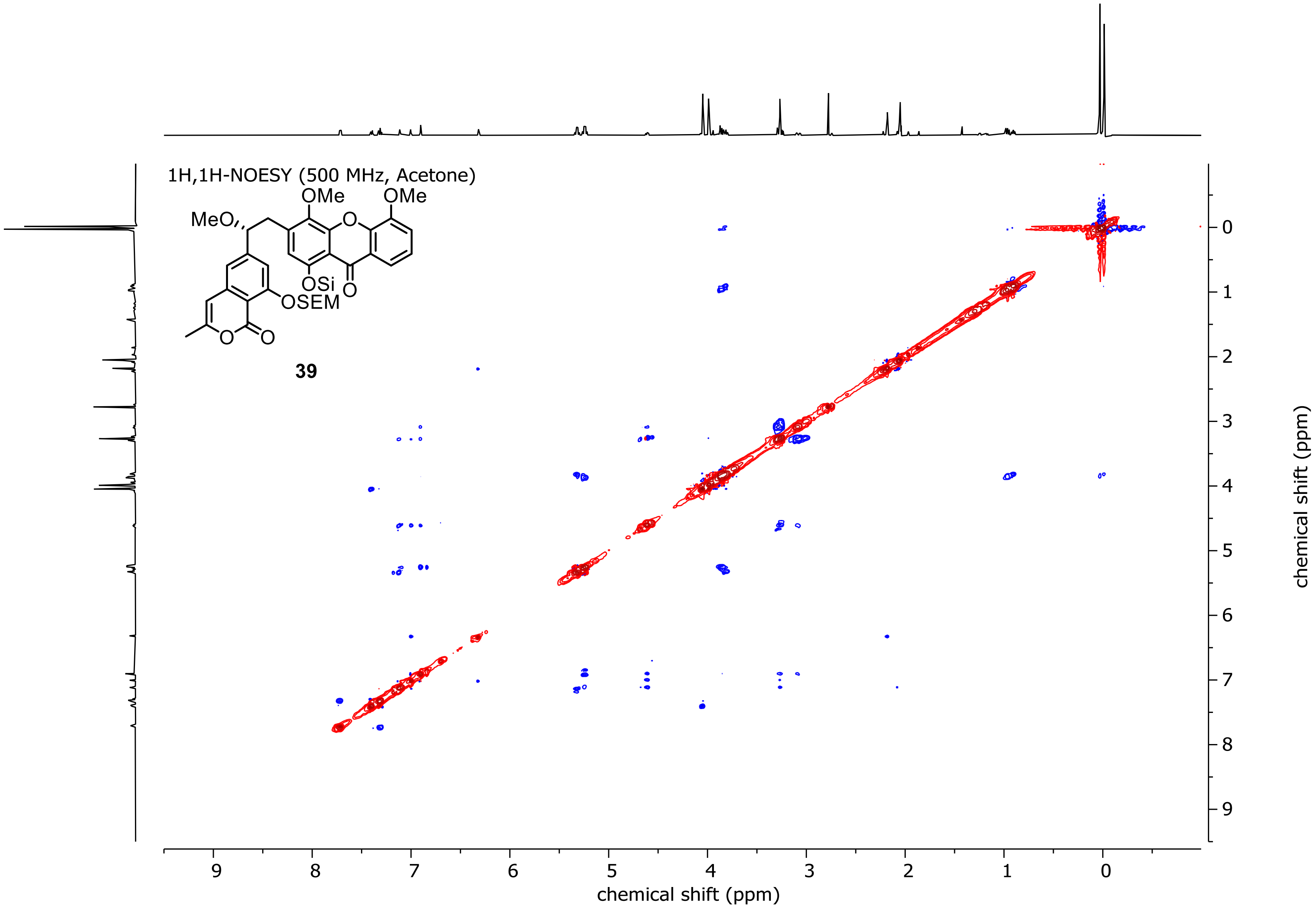

1H,1H-NOESY (500 MHz, Acetone)
OMe
OMe
MeO
O
OSi
O
OSEM
O
O
39
chemical shift (ppm)
chemical shift (ppm)

1H NMR (700 MHz, Acetone)

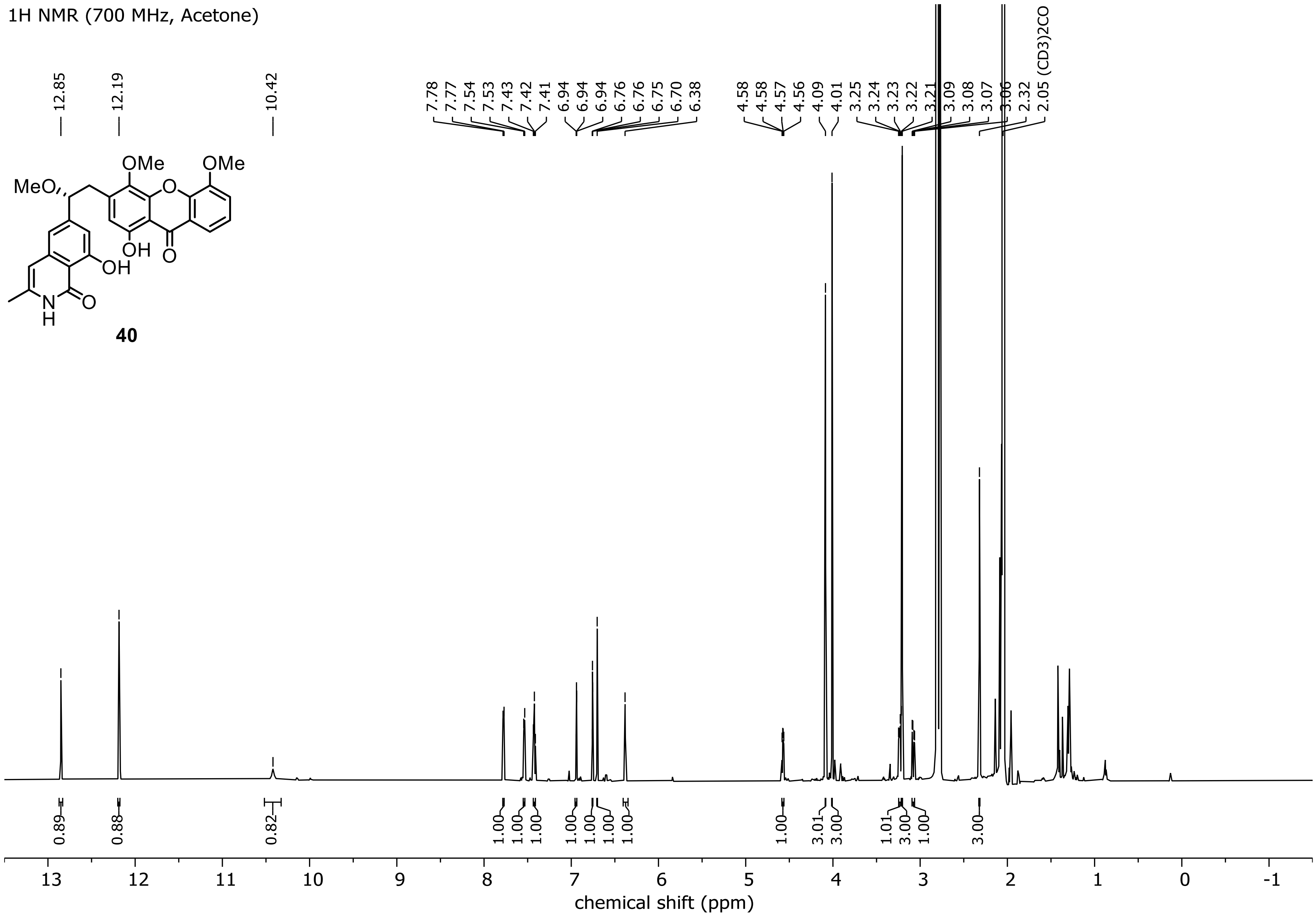

40

13C NMR (176 MHz, Acetone)

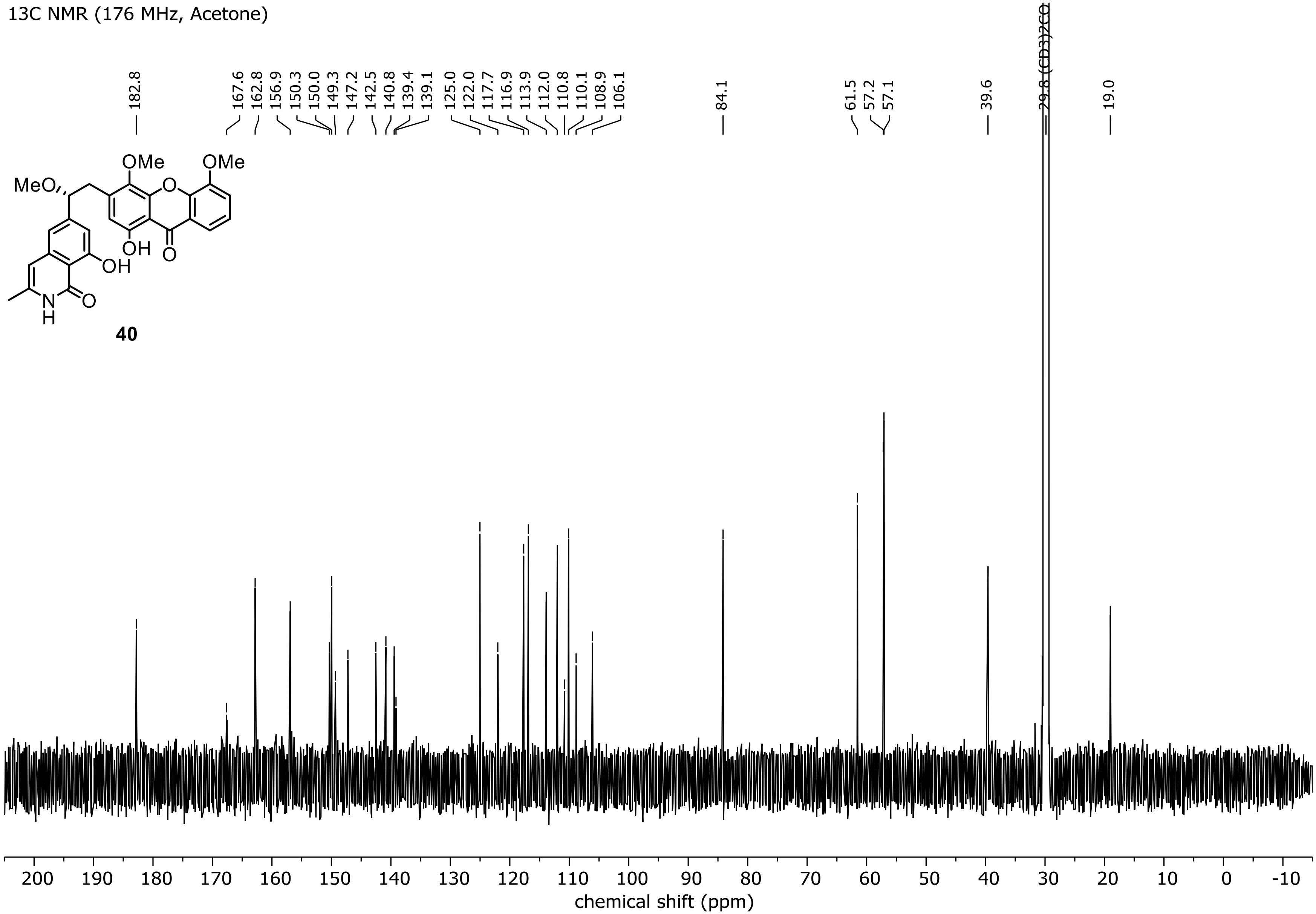

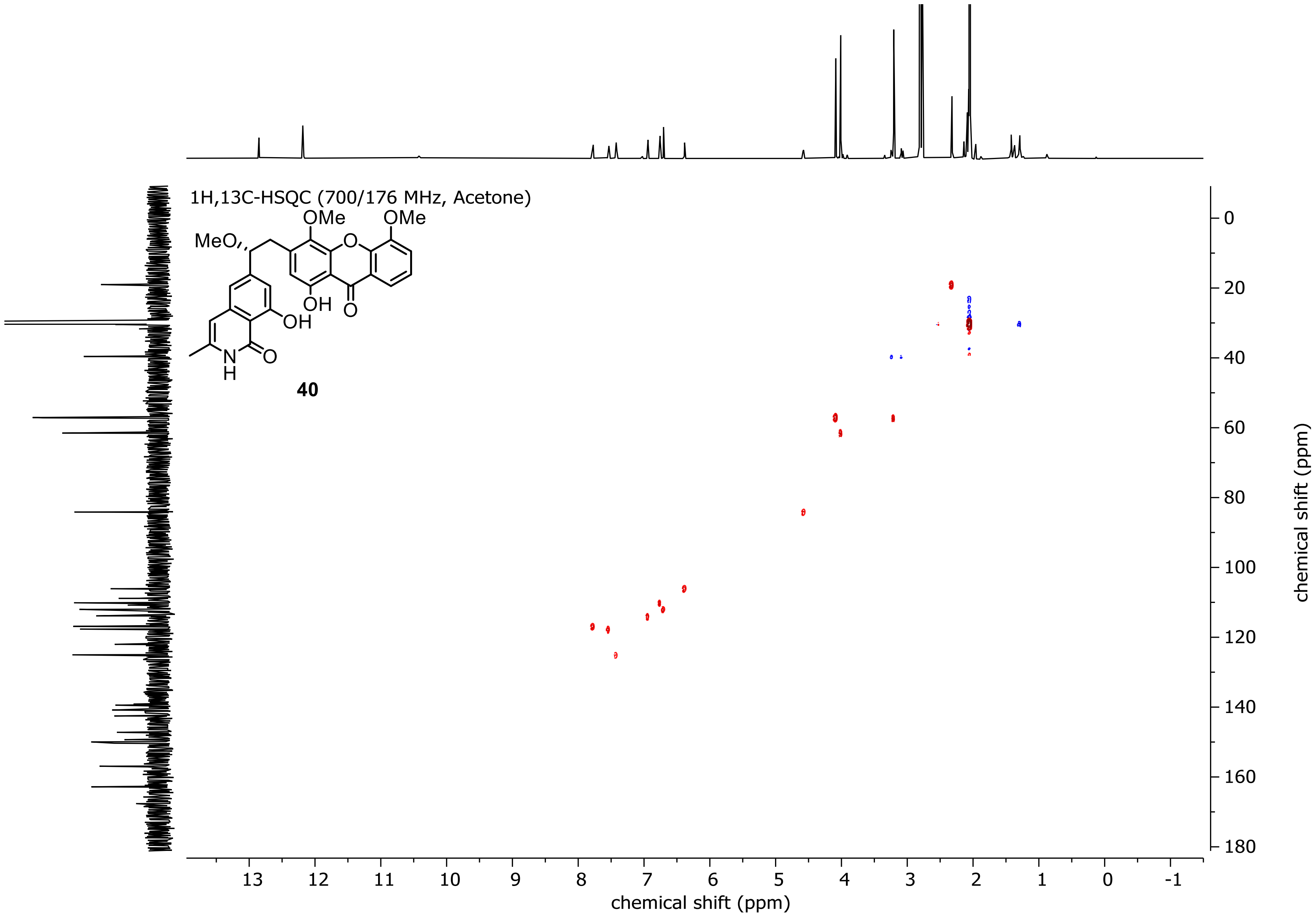
1H,13C-HSQC (700/176 MHz, Acetone)
OMe
OMe
MeO
O
OH
O
OH
N
H
O
40
chemical shift (ppm)
chemical shift (ppm)

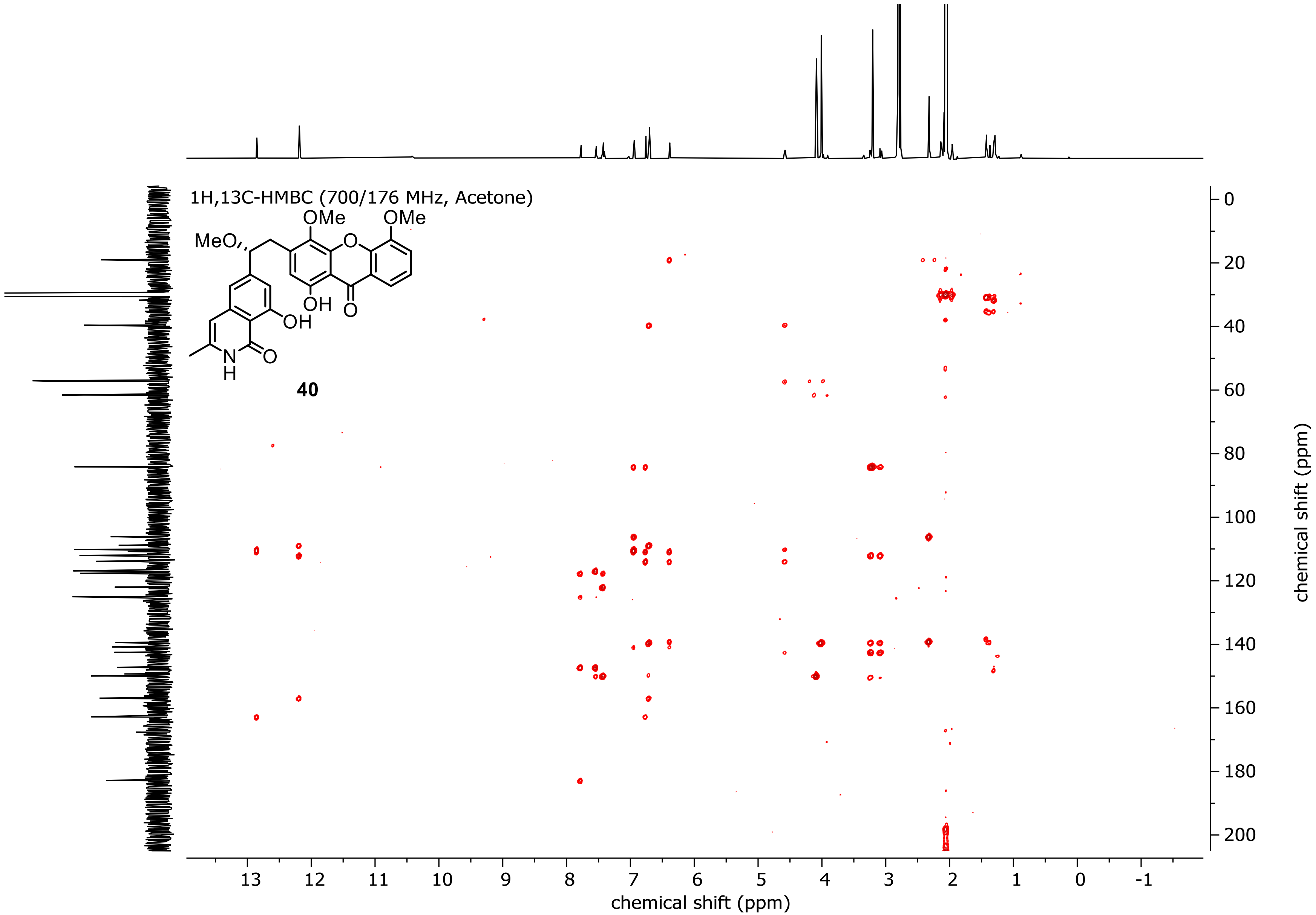
1H,13C-HMBC (700/176 MHz, Acetone)
OMe
OMe
MeO
O
OH
O
OH
N
H
O
40
0
20
40
60
80
100
120
140
160
180
200
chemical shift (ppm)
13
12
11
10
9
8
7
6
5
4
3
2
1
0
-1
chemical shift (ppm)

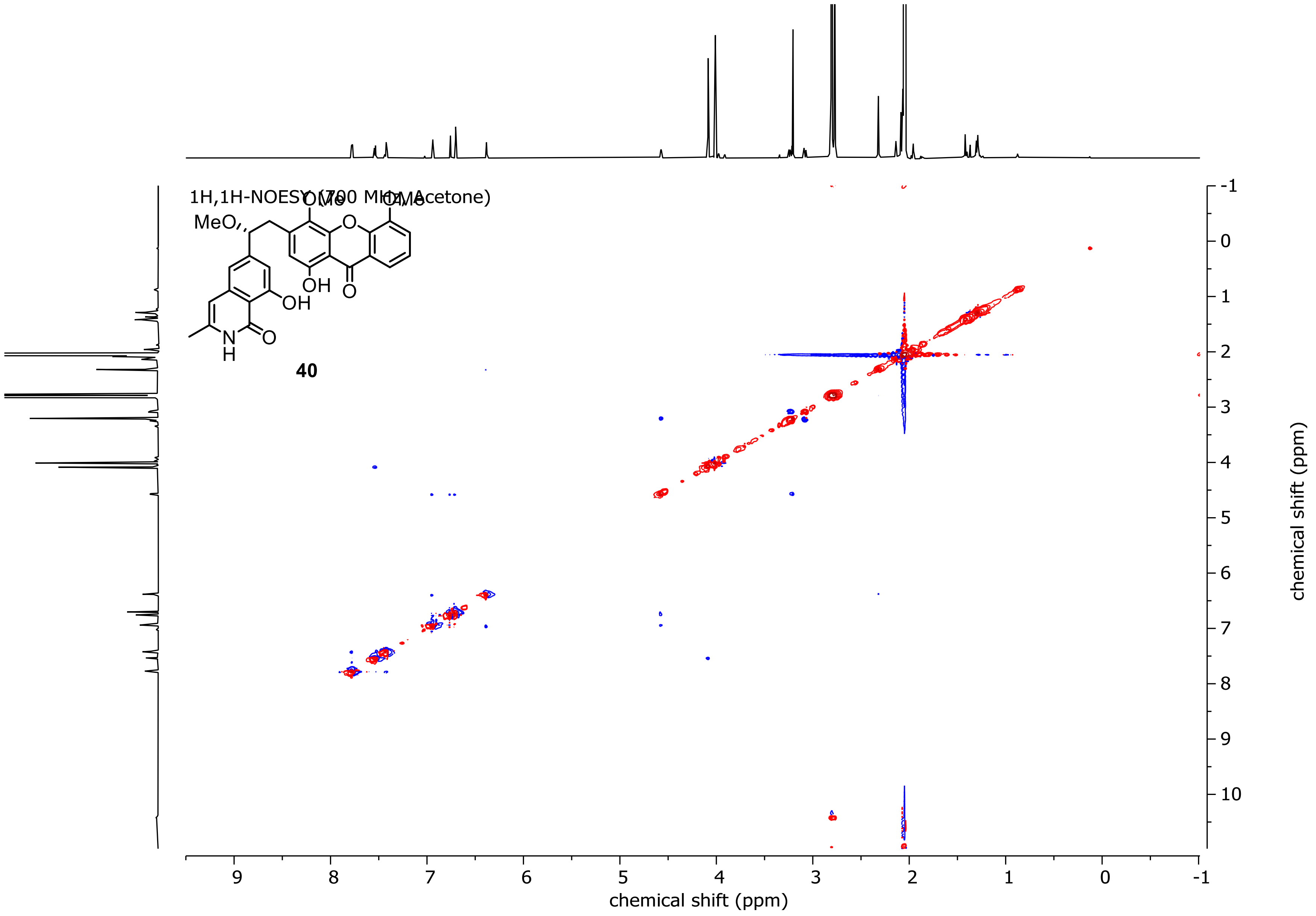
1H,1H-NOESY (700 MHz, Acetone)
MeO
OMe
O
OMe
OH
OH
O
N
H
O
40
chemical shift (ppm)
chemical shift (ppm)

1H NMR (700 MHz, DMSO)

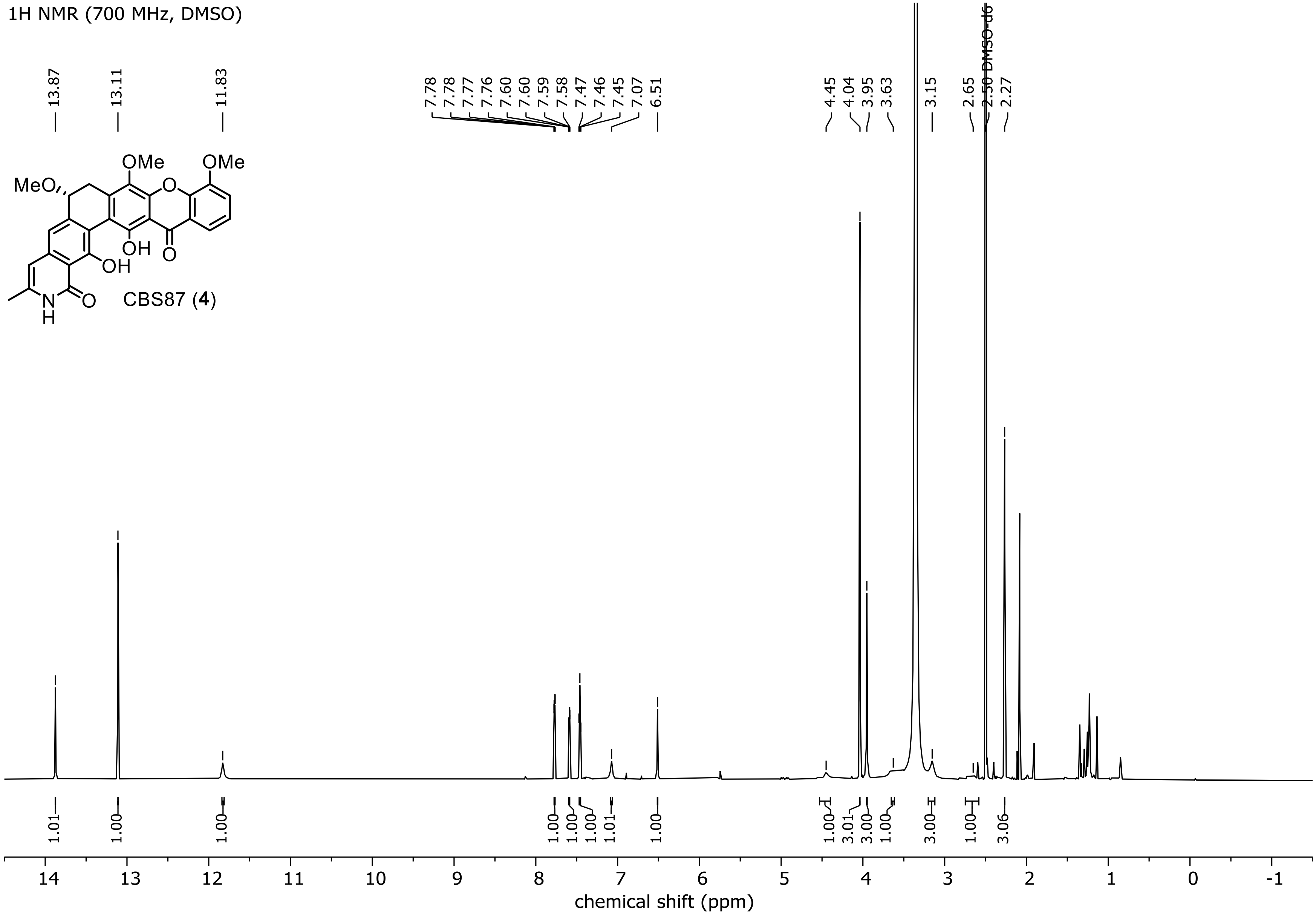

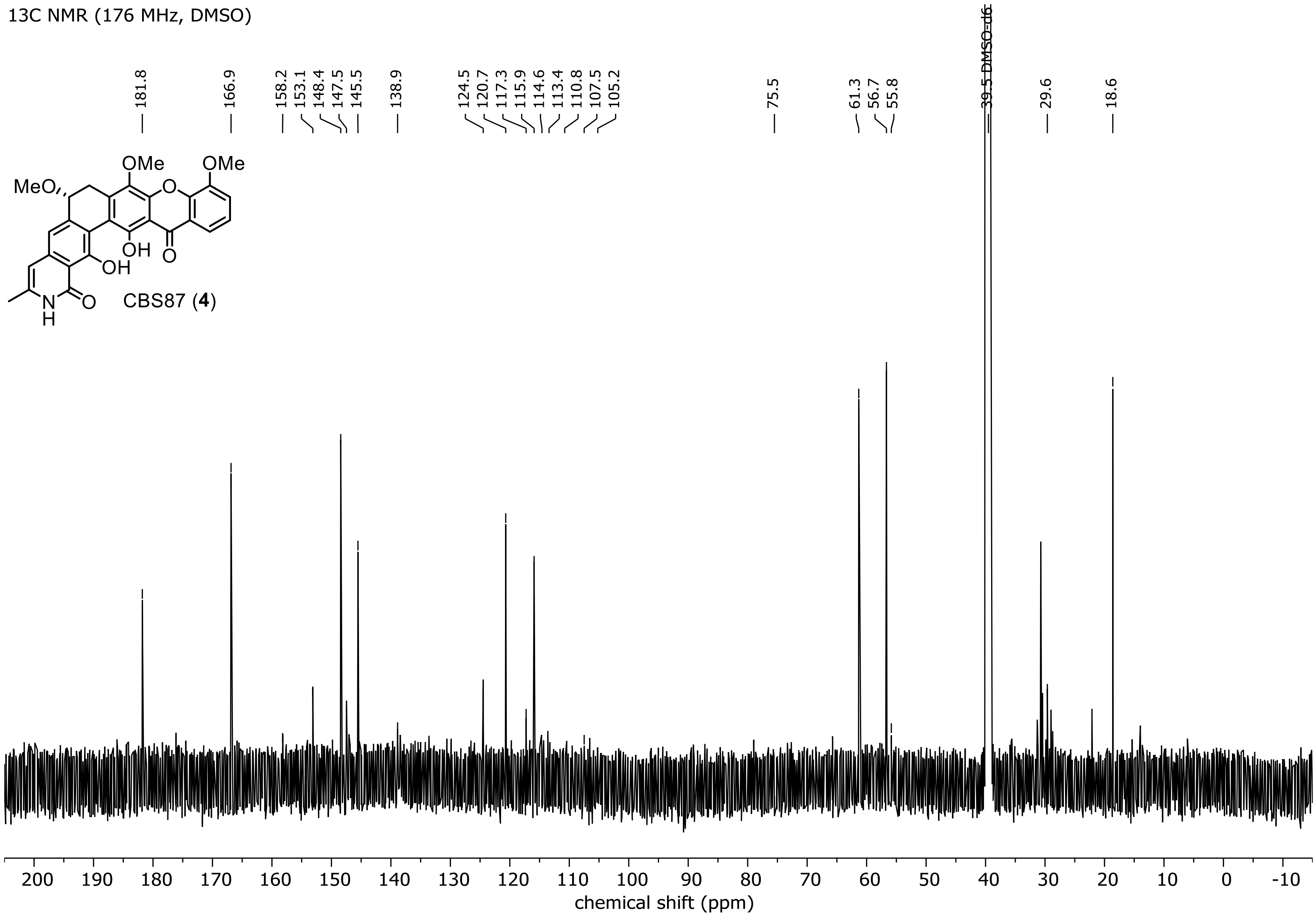
13C NMR (176 MHz, DMSO)
181.8
166.9
158.2
153.1
148.4
147.5
145.5
138.9
124.5
120.7
117.3
115.9
114.6
113.4
110.8
107.5
105.2
75.5
61.3
56.7
55.8
39.5 DMSO-d6
29.6
18.6
OMe
OMe
MeO
O
OH
OH
O
N
H
O
CBS87 (4)
200
190
180
170
160
150
140
130
120
110
100
90
80
70
60
50
40
30
20
10
0
-10
chemical shift (ppm)

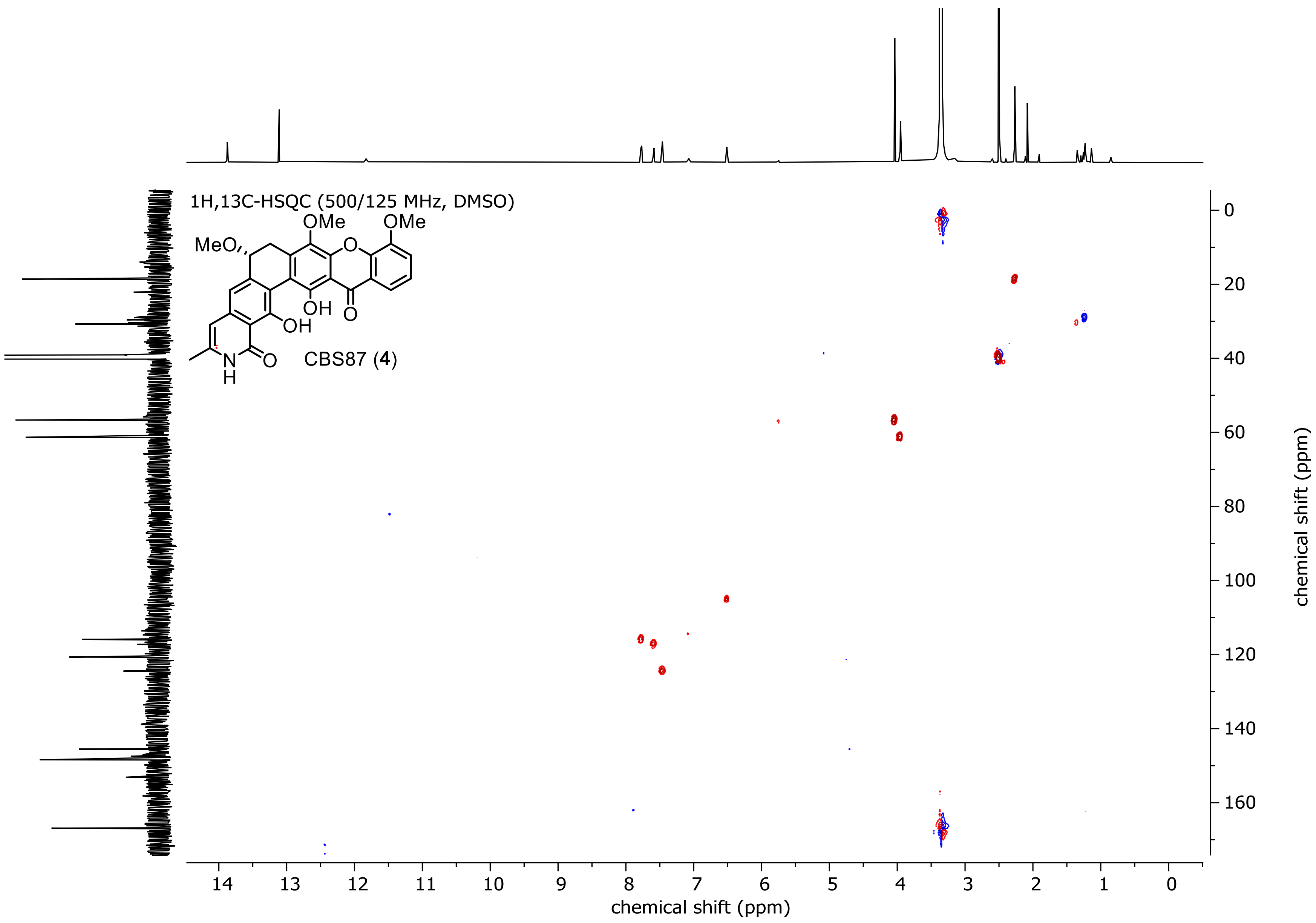
1H,13C-HSQC (500/125 MHz, DMSO)
OMe
OMe
MeO
O
OH
O
OH
N
H
O
CBS87 (4)
0
20
40
60
80
100
120
140
160
chemical shift (ppm)
14
13
12
11
10
9
8
7
6
5
4
3
2
1
0
chemical shift (ppm)

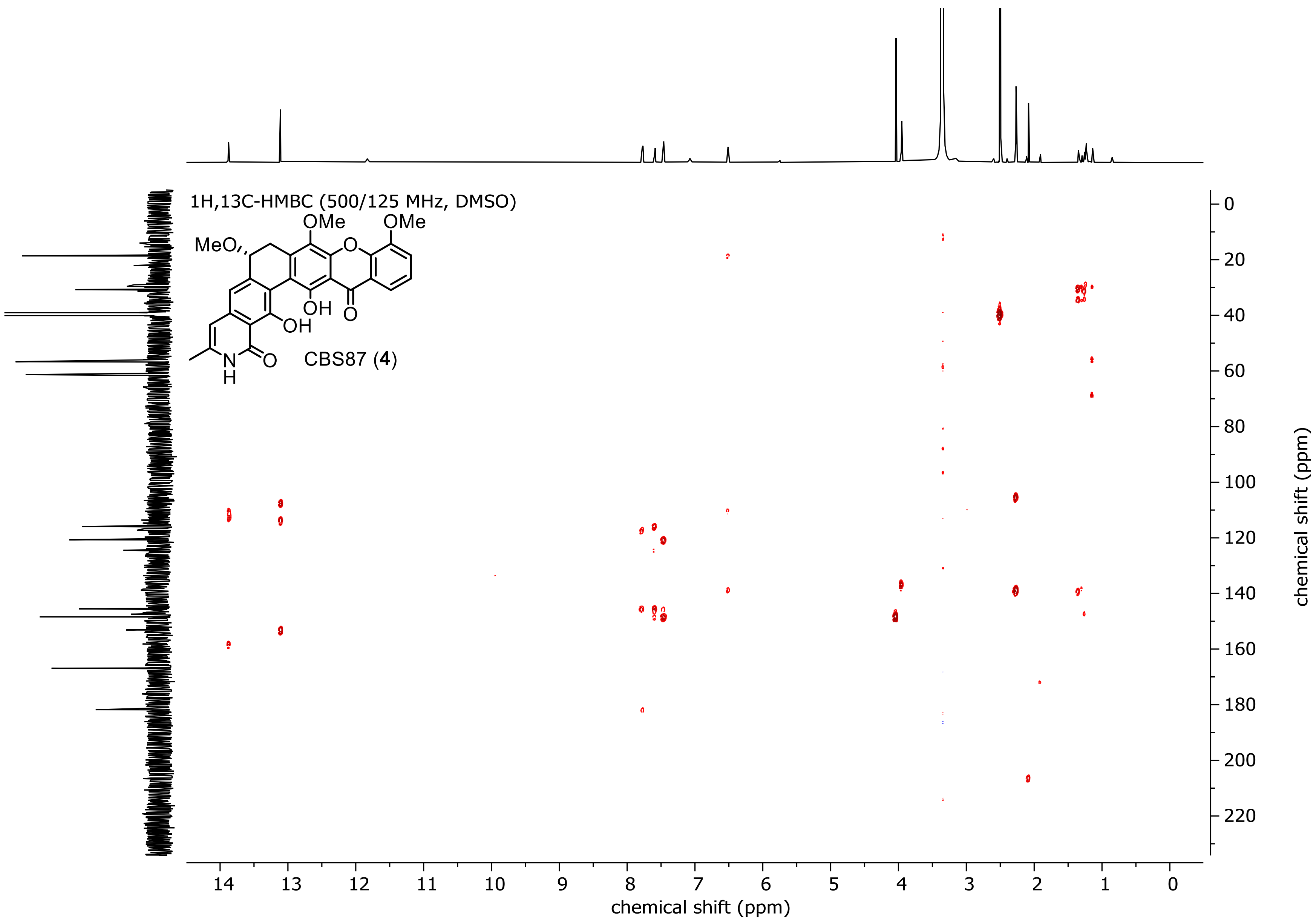
1H,13C-HMBC (500/125 MHz, DMSO)
MeO
OMe
OMe
O
OH
OH
O
N
H
O
CBS87 (4)
chemical shift (ppm)
chemical shift (ppm)
14 13 12 11 10 9 8 7 6 5 4 3 2 1 0
0 20 40 60 80 100 120 140 160 180 200 220

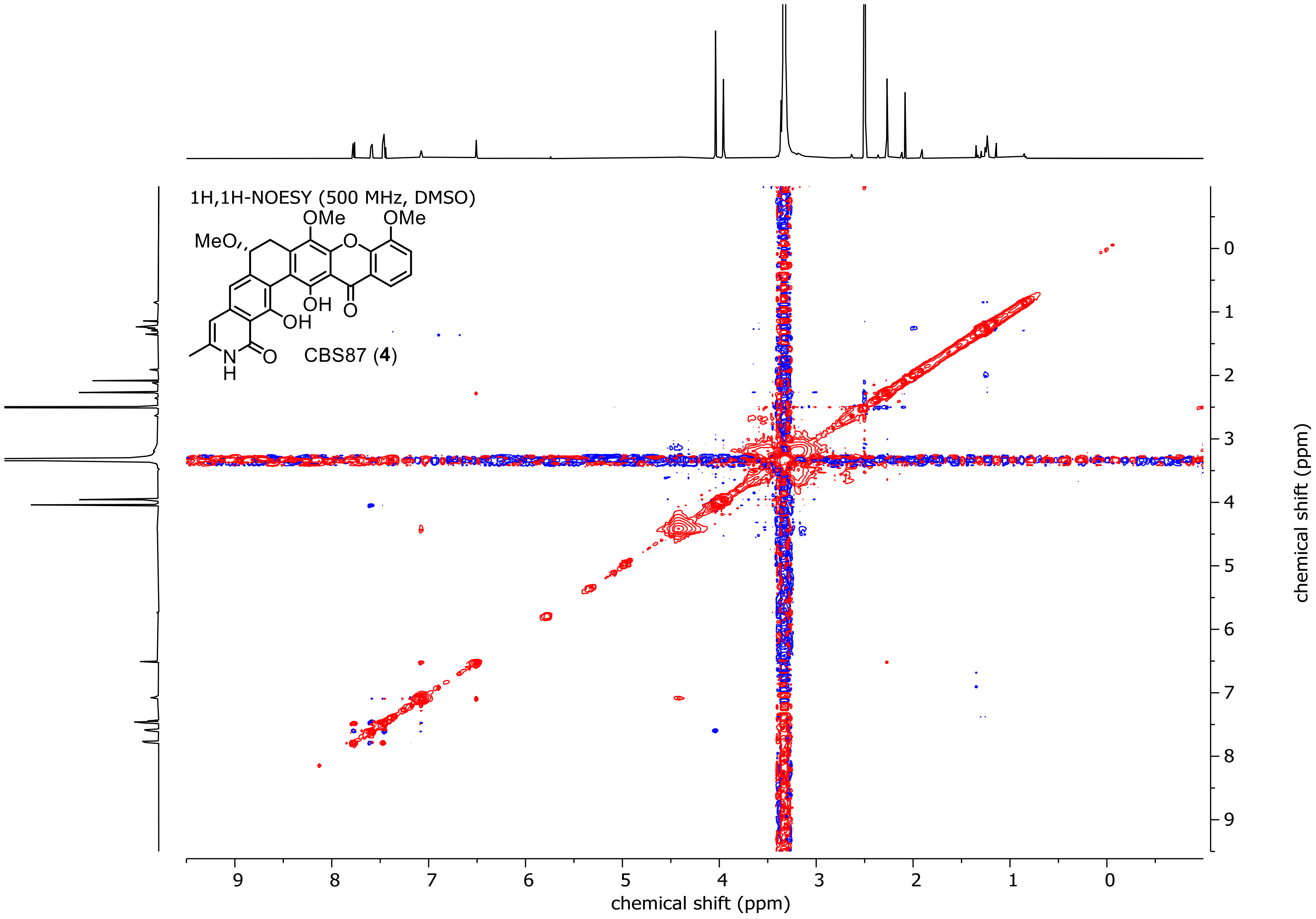

1H,1H-NOESY (500 MHz, DMSO)
OMe
OMe
MeO
O
OH
O
OH
N
H
O
CBS87 (4)
chemical shift (ppm)
chemical shift (ppm)

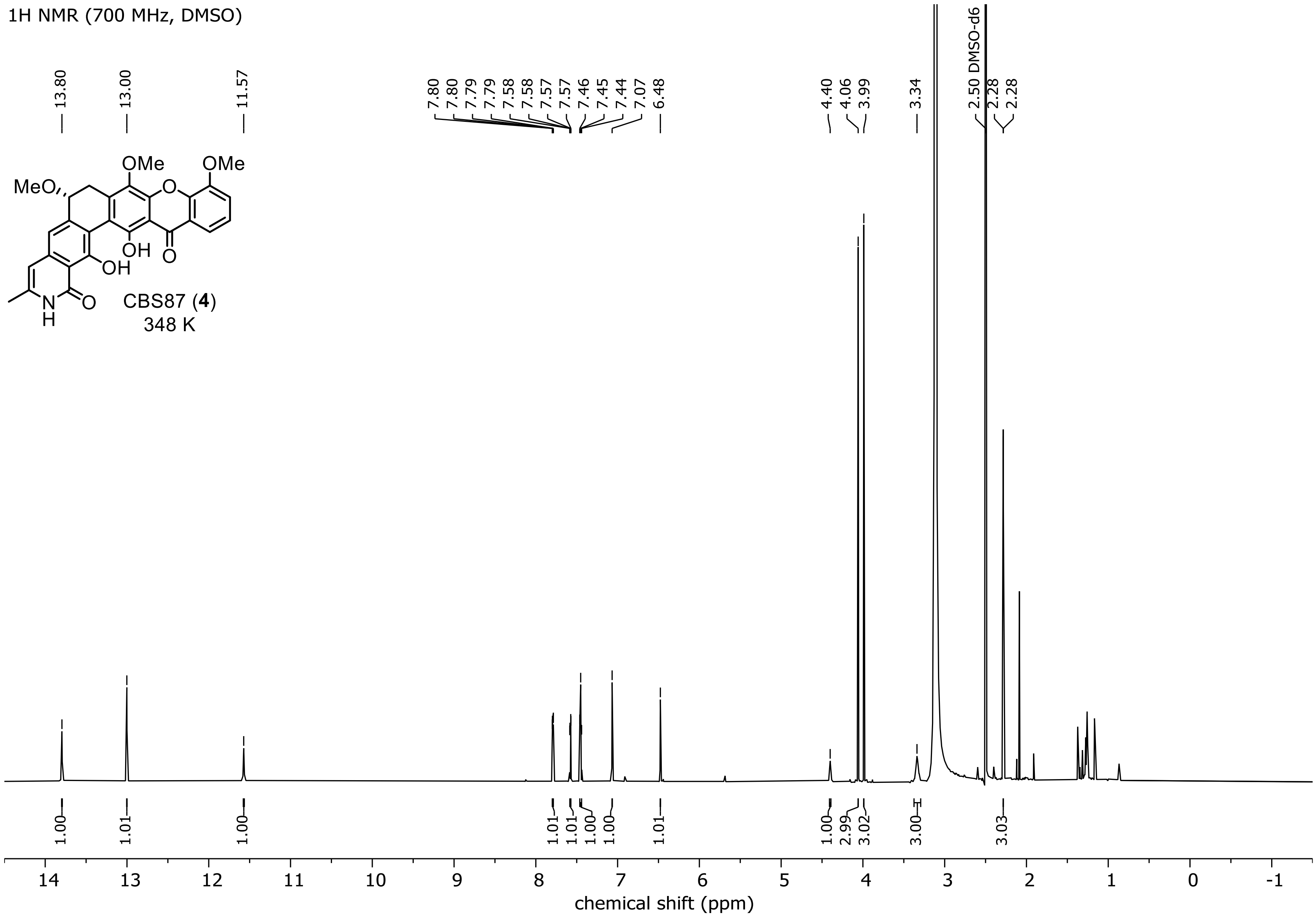
1H NMR (700 MHz, DMSO)
13.80
13.00
11.57
7.80
7.80
7.79
7.79
7.58
7.58
7.57
7.57
7.46
7.45
7.44
7.07
6.48
4.40
4.06
3.99
3.34
2.50 DMSO-d6
2.28
2.28
OMe
OMe
MeO
OH
OH
O
N
H
O
CBS87 (4)
348 K
1.00
1.01
1.00
1.01
1.01
1.00
1.00
1.01
1.00
2.99
3.02
3.00
3.03
14
13
12
11
10
9
8
7
6
5
4
3
2
1
0
-1
chemical shift (ppm)

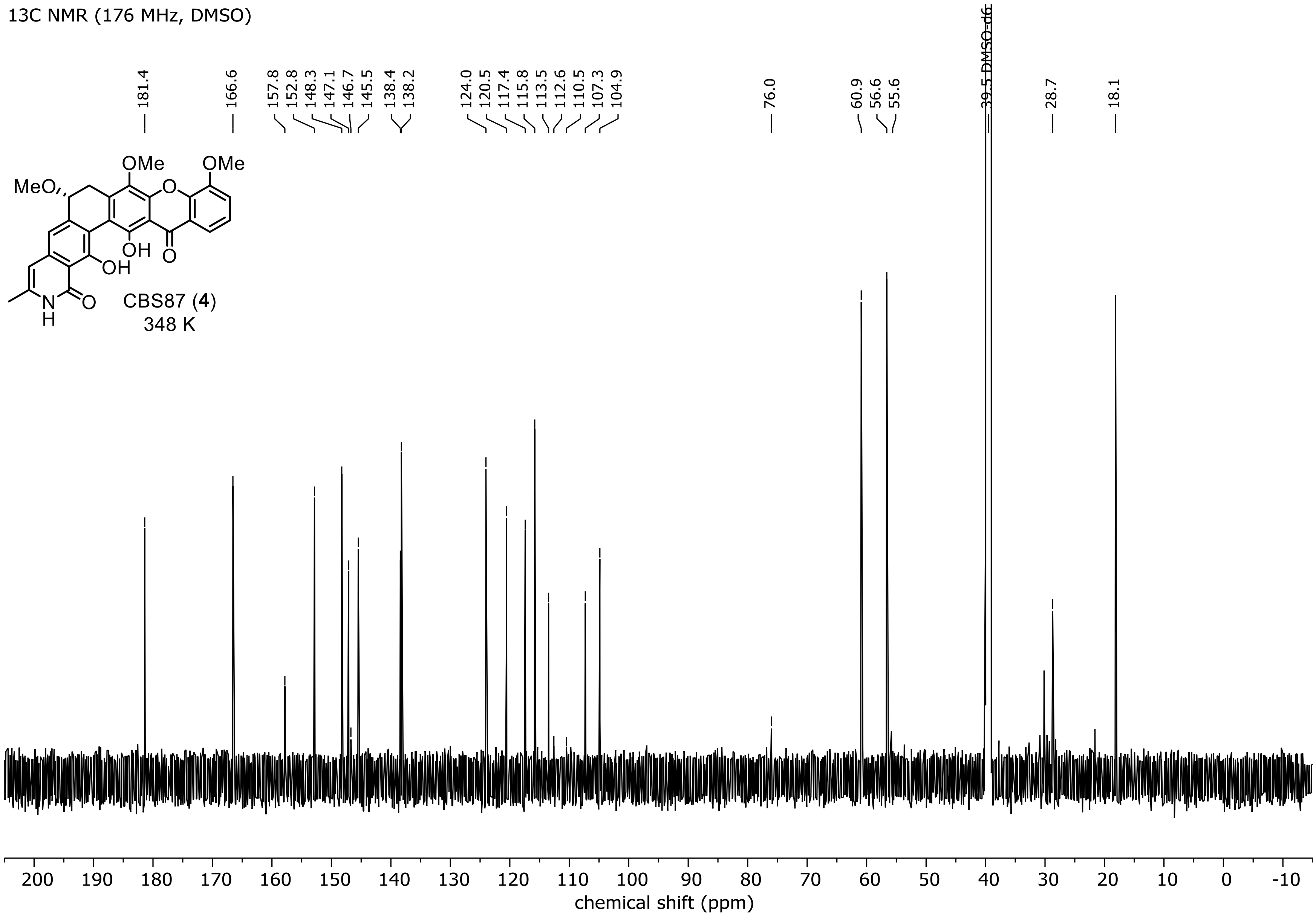
13C NMR (176 MHz, DMSO)
181.4
166.6
157.8
152.8
148.3
147.1
146.7
145.5
138.4
138.2
124.0
120.5
117.4
115.8
113.5
112.6
110.5
107.3
104.9
76.0
60.9
56.6
55.6
39.5 DMSO-d6
28.7
18.1
OMe
OMe
MeO
OH
OH
O
O
N
H
O
CBS87 (**4**)
348 K
chemical shift (ppm)

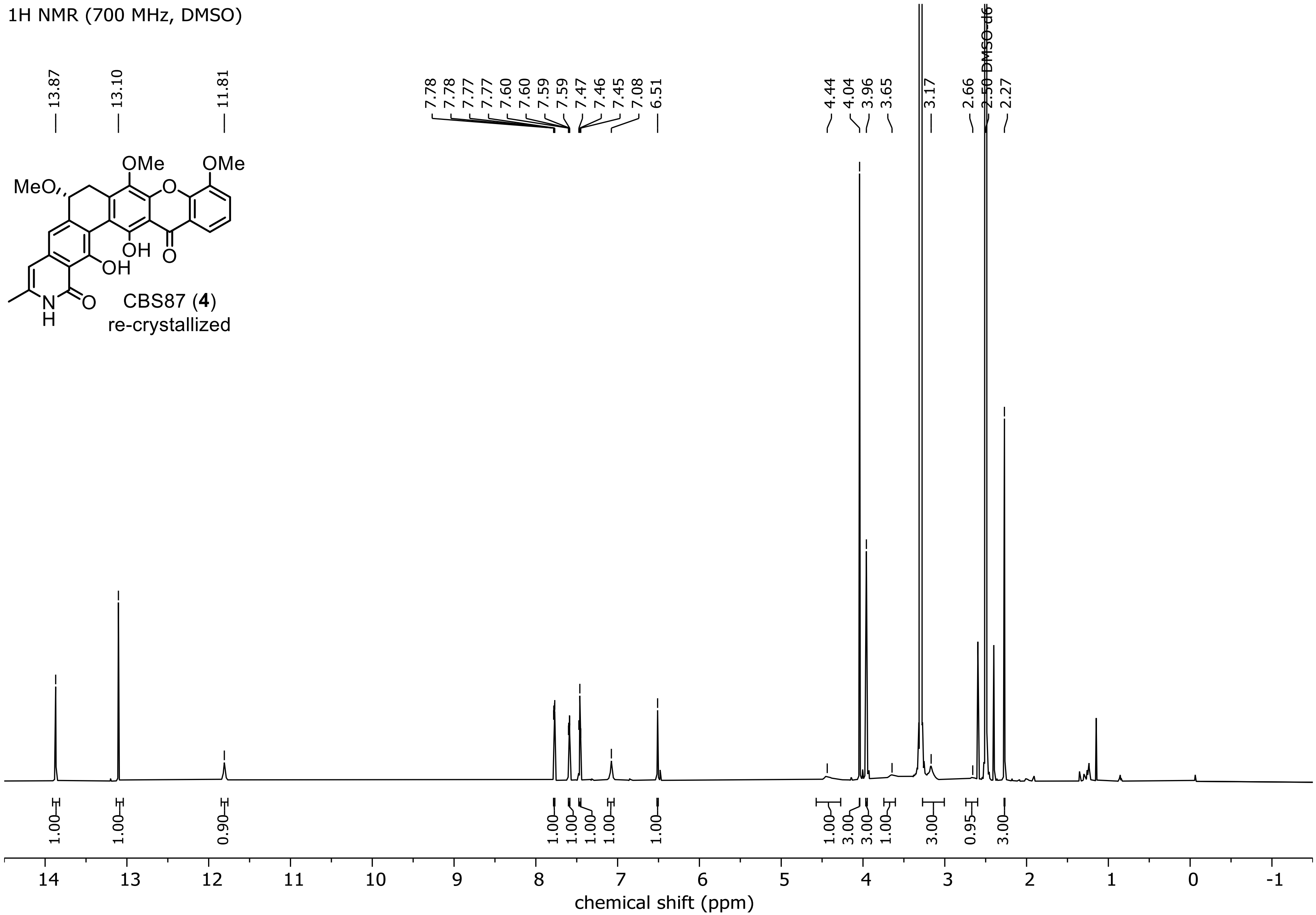
1H NMR (700 MHz, DMSO)
13.87
13.10
11.81
7.78
7.78
7.77
7.77
7.60
7.60
7.59
7.59
7.47
7.46
7.45
7.08
6.51
4.44
4.04
3.96
3.65
3.17
2.66
2.50 DMSO-d6
2.27
OMe
OMe
MeO
O
OH
O
OH
N
H
O
CBS87 (**4**)
re-crystallized
1.00
1.00
0.90
1.00
1.00
1.00
1.00
1.00
1.00
3.00
3.00
1.00
3.00
0.95
3.00
14
13
12
11
10
9
8
7
6
5
4
3
2
1
0
-1
chemical shift (ppm)

7.5 Leading to SCB72 (**3**)

1H NMR (700 MHz, Acetone)

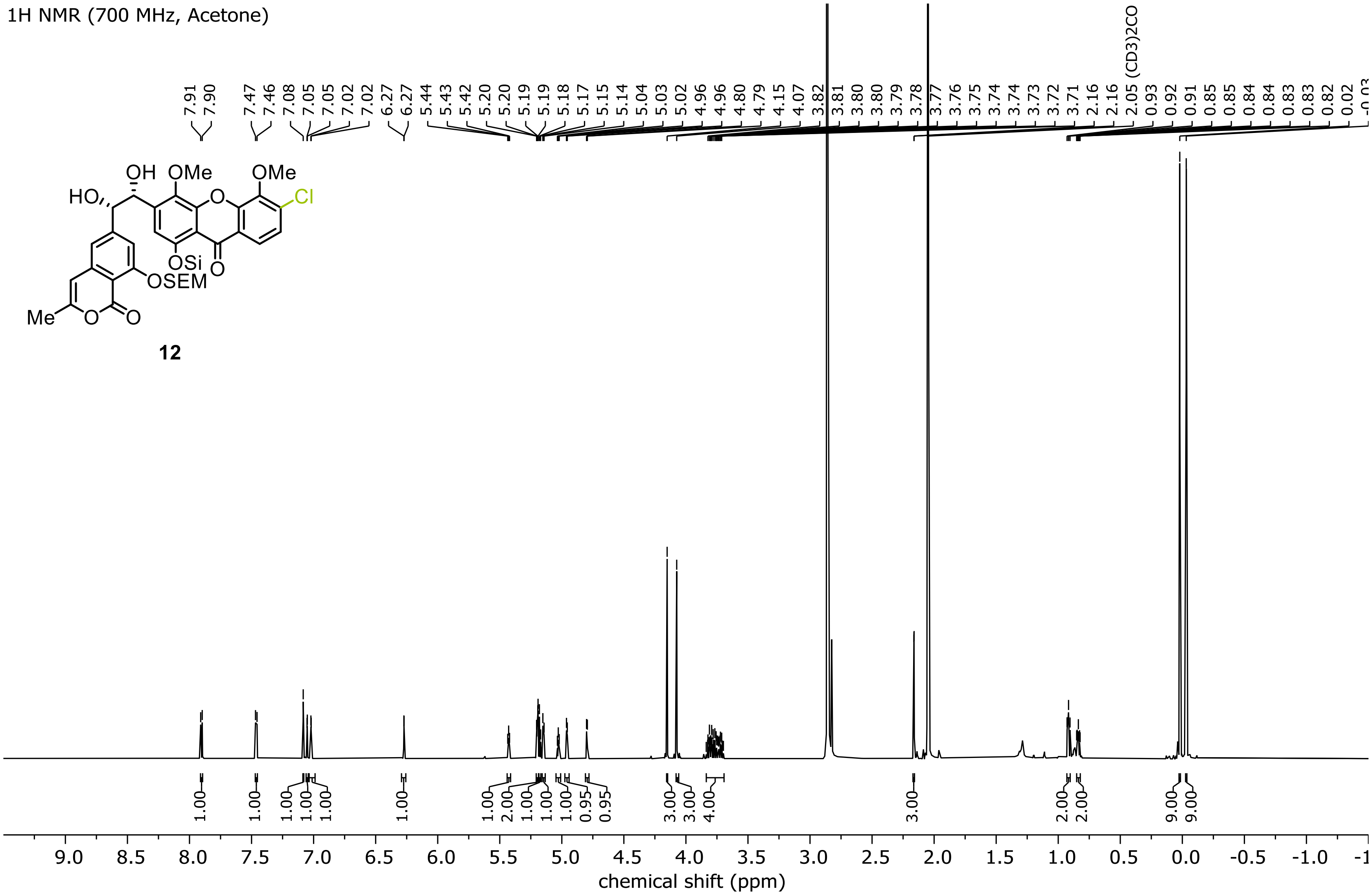

13C NMR (176 MHz, Acetone)

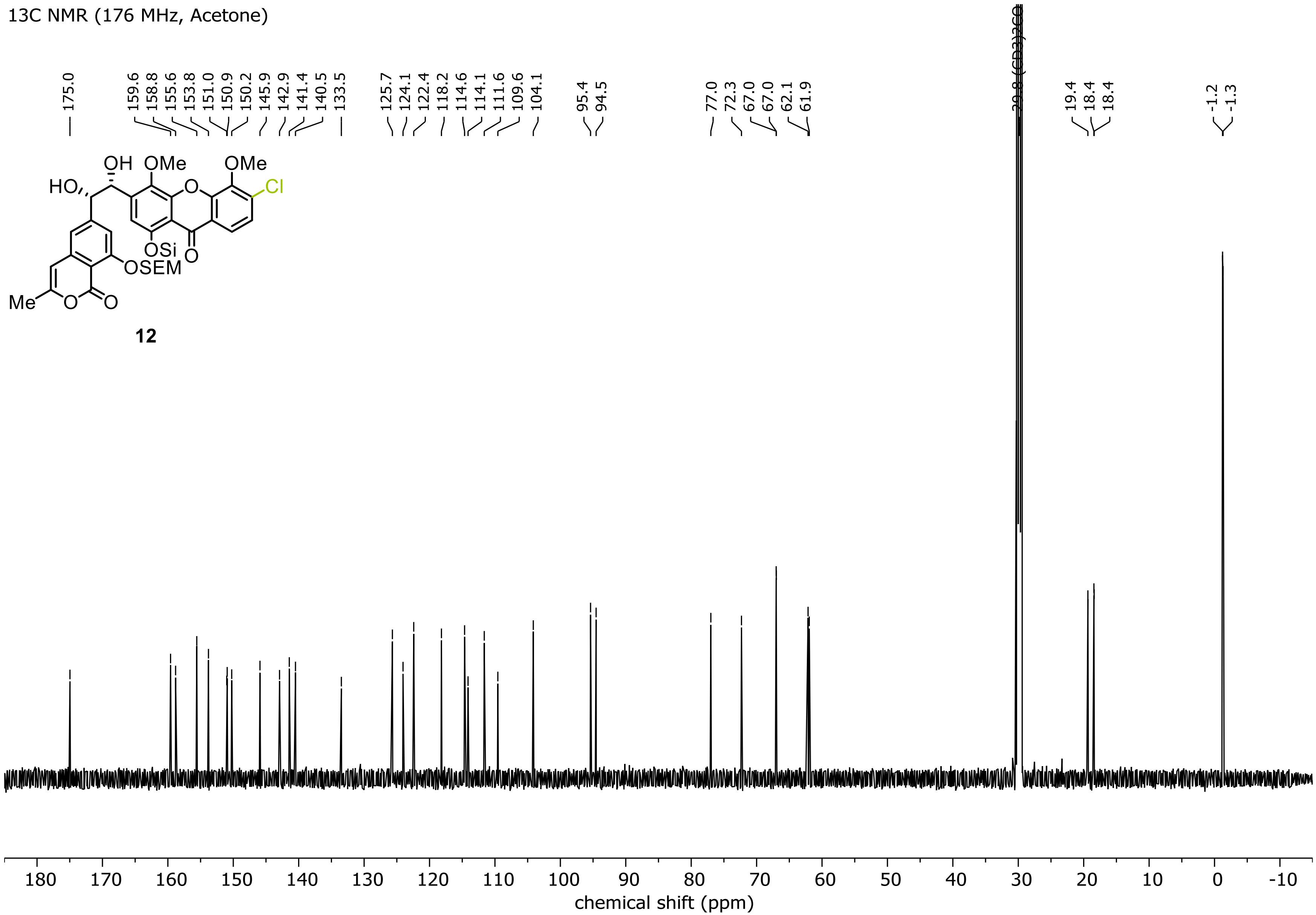

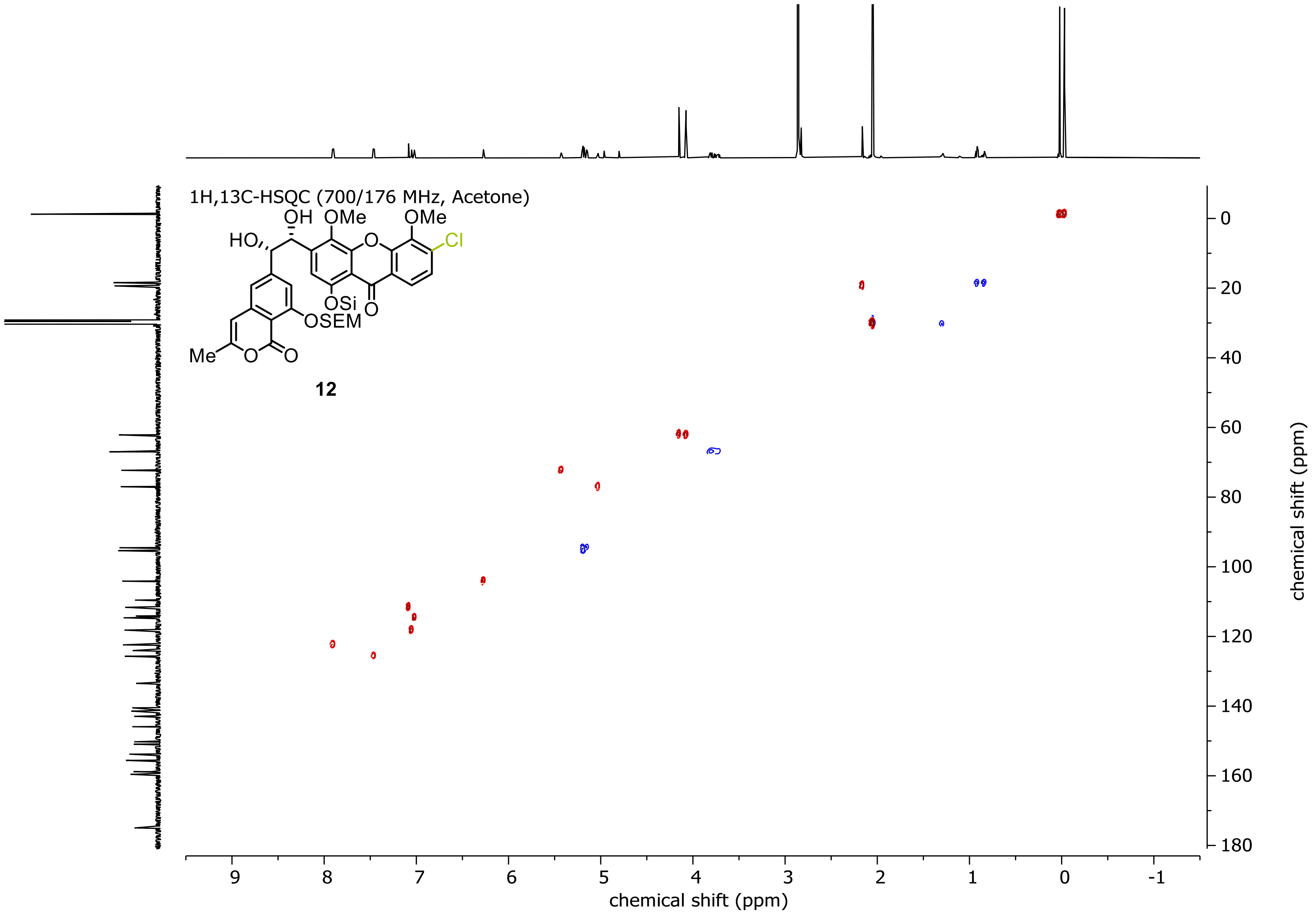
1H,13C-HSQC (700/176 MHz, Acetone)
OH
OMe
OMe
HO
O
Cl
OSi
O
OSEM
Me
O
O
12
chemical shift (ppm)
chemical shift (ppm)

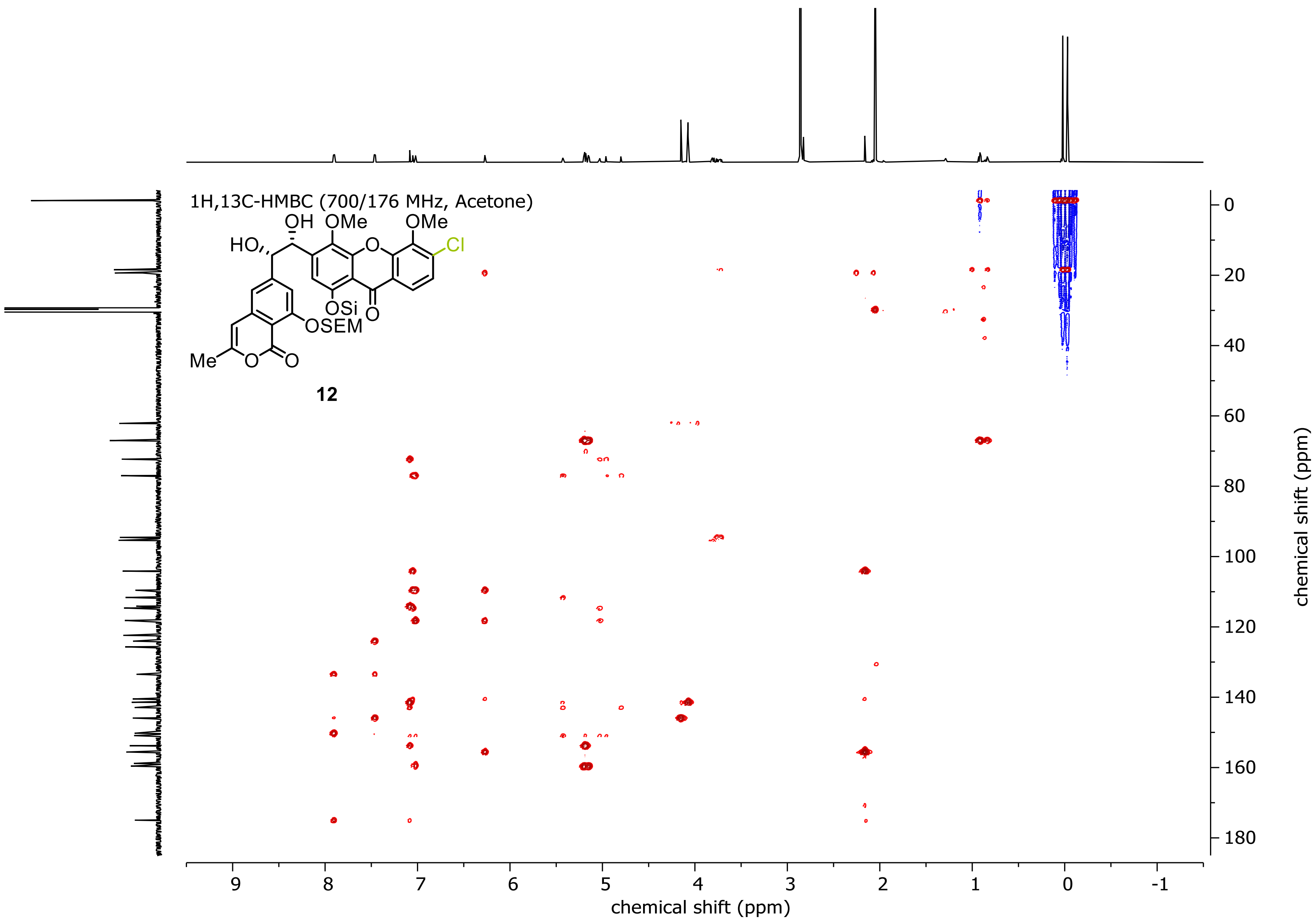
1H,13C-HMBC (700/176 MHz, Acetone)
OH OMe OMe
HO
O
Cl
OSi
O
OSEM
Me
O
O
12
chemical shift (ppm)
chemical shift (ppm)

1H NMR (400 MHz, Acetone)

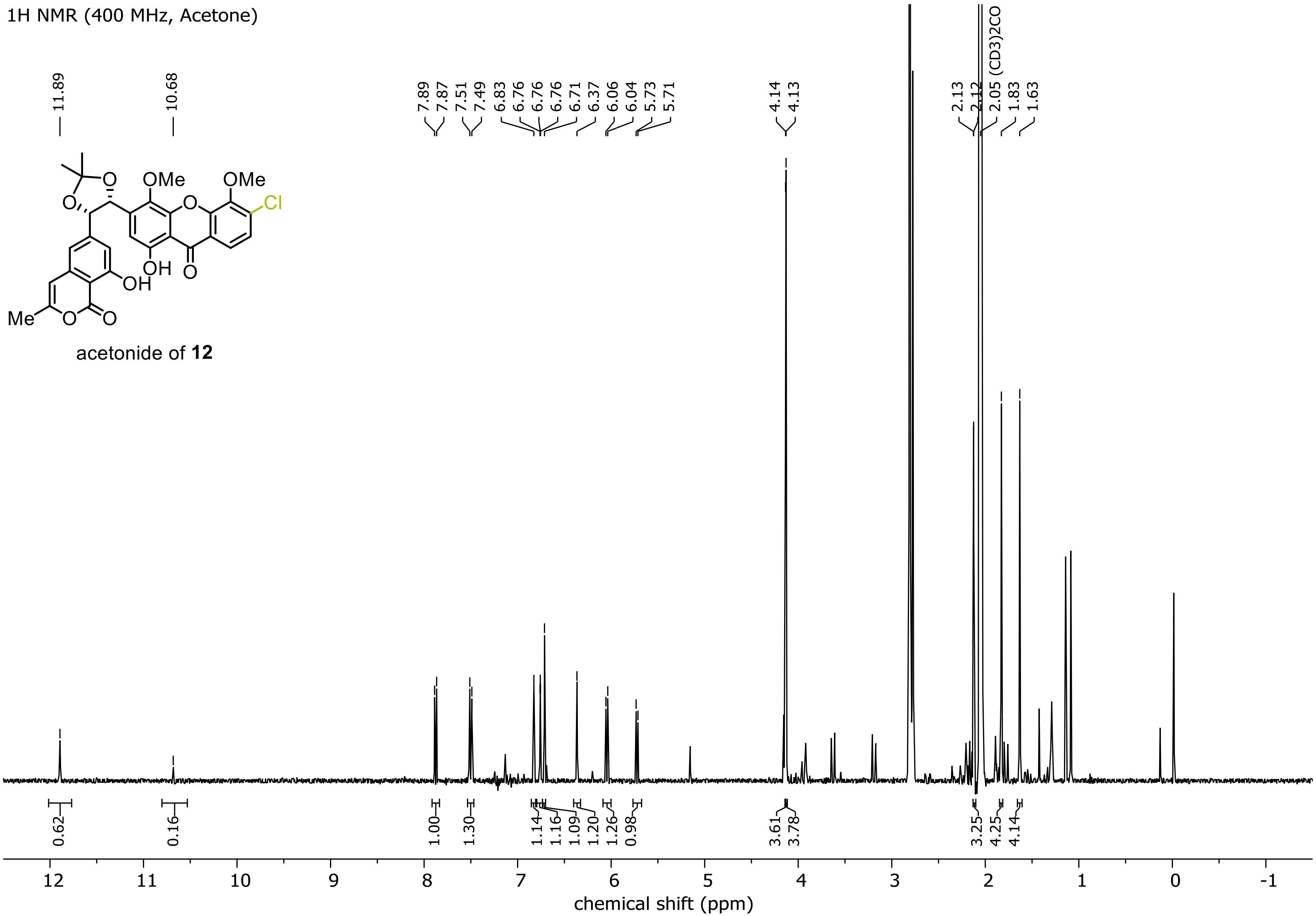

acetonide of **12**

13C NMR (176 MHz, Acetone)

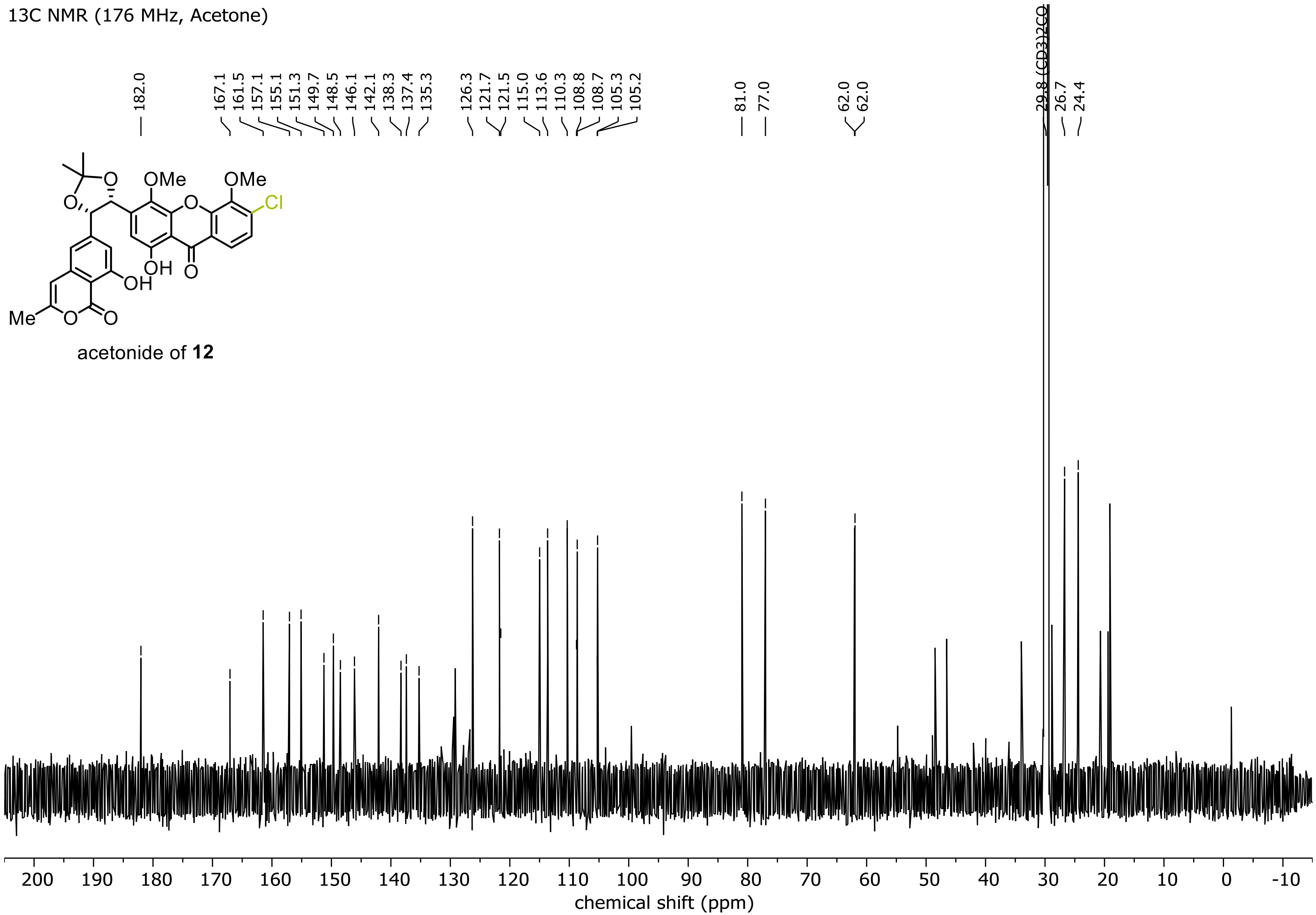

acetonide of **12**

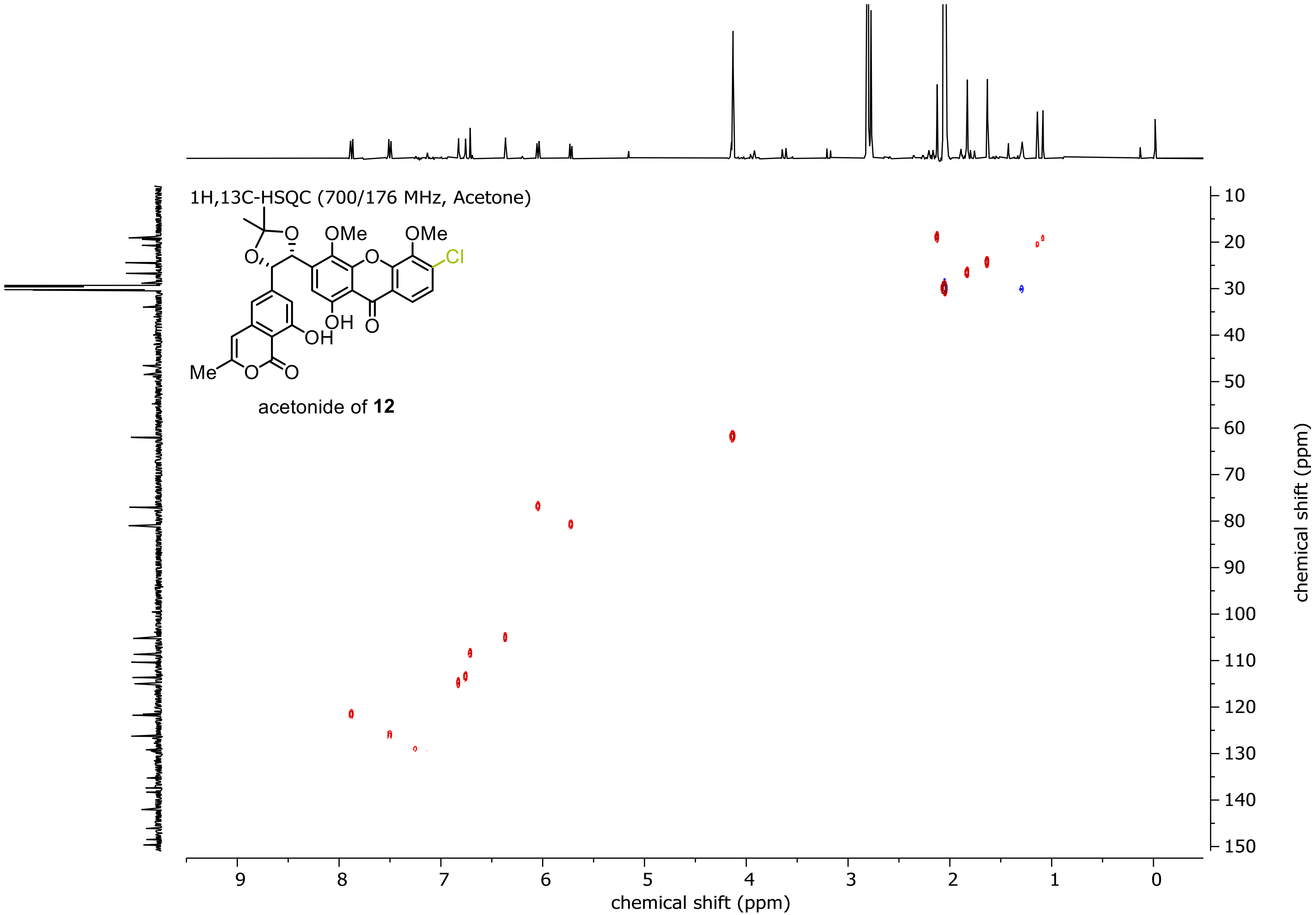
1H,13C-HSQC (700/176 MHz, Acetone)
OMe
OMe
Cl
OH
OH
Me
acetonide of 12
chemical shift (ppm)
chemical shift (ppm)

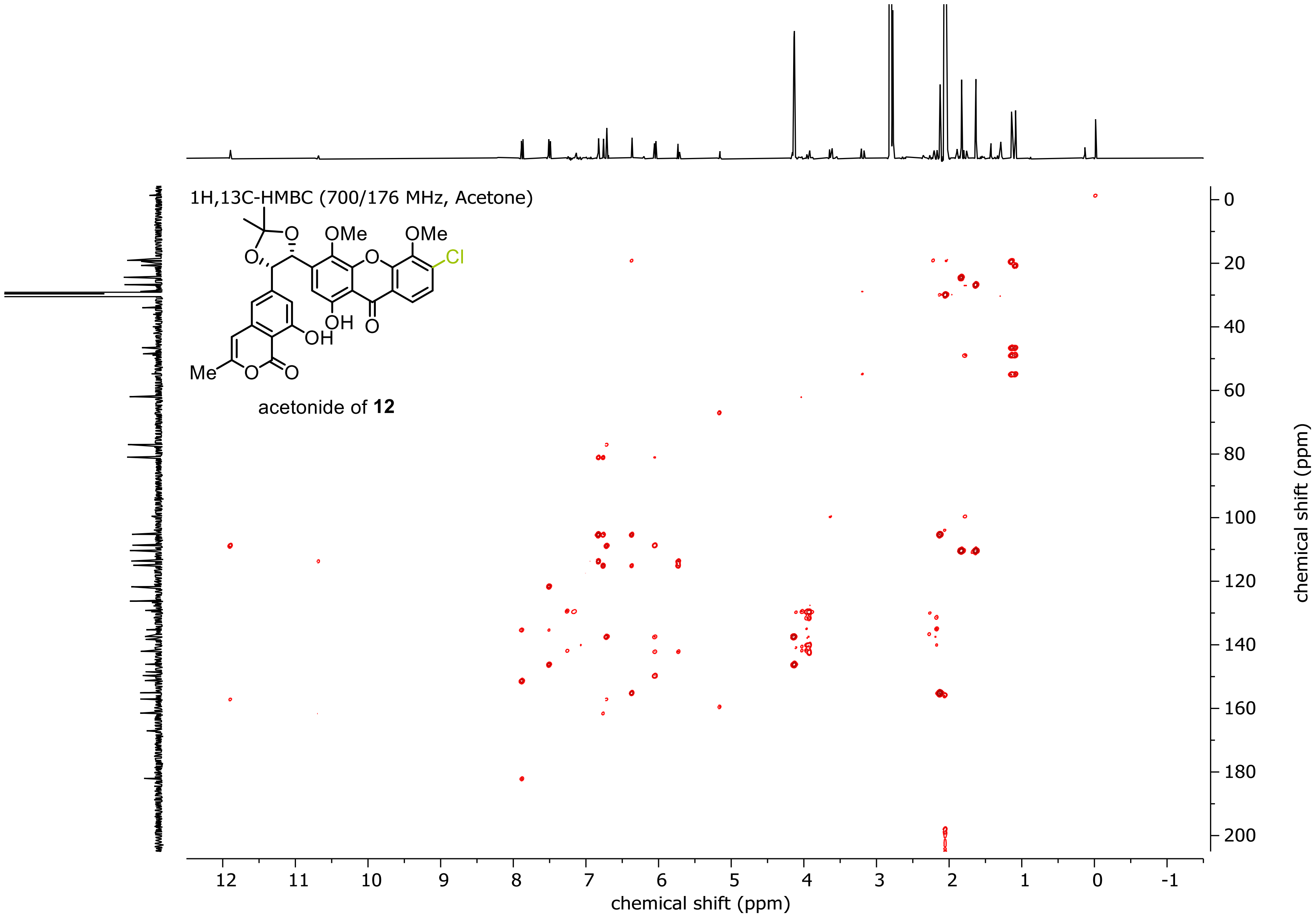
1H,13C-HMBC (700/176 MHz, Acetone)
OMe
OMe
Cl
OH
OH
O
Me
acetonide of 12
chemical shift (ppm)
chemical shift (ppm)

1H NMR (499 MHz, Acetone)

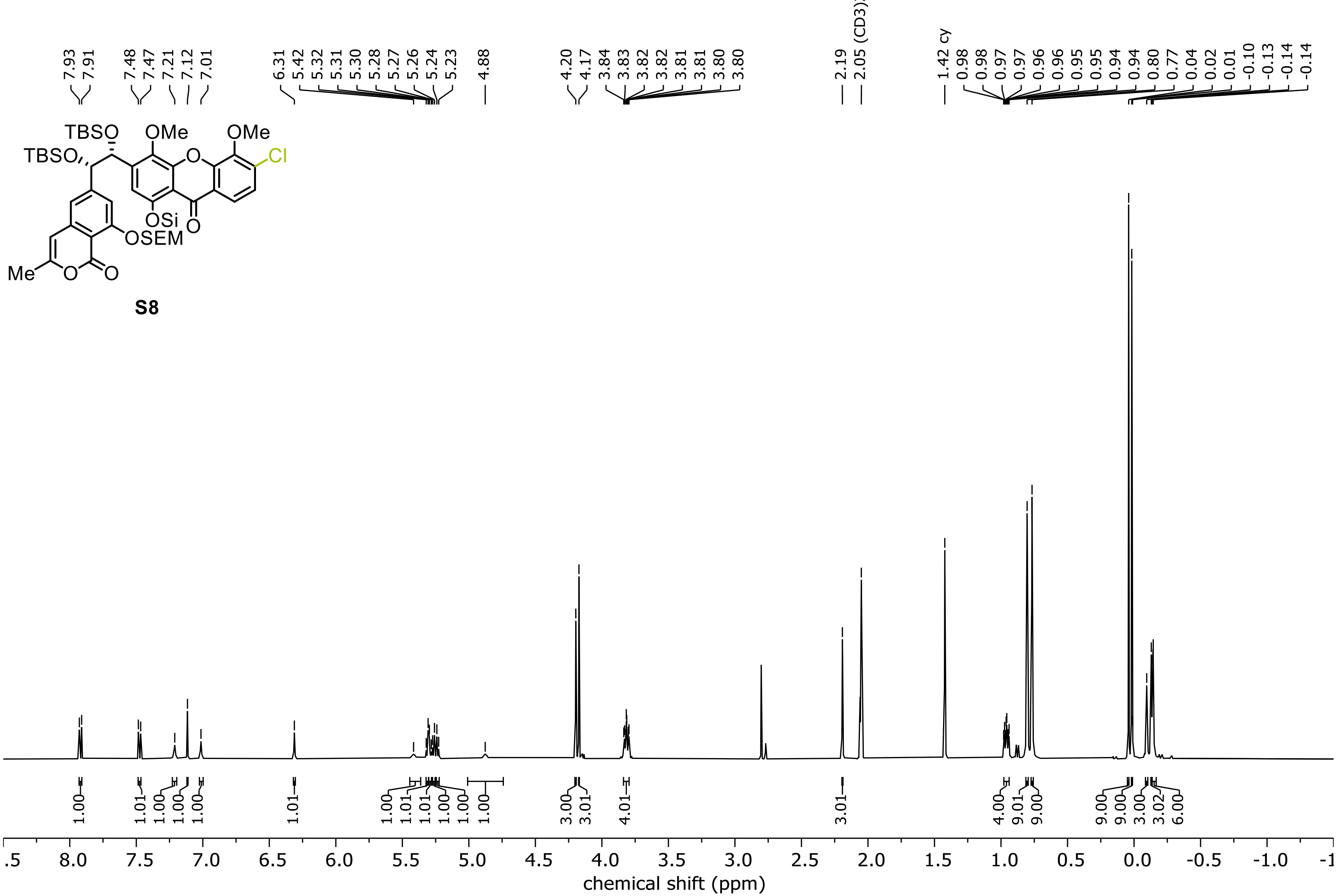

S8

13C NMR (125 MHz, Acetone)

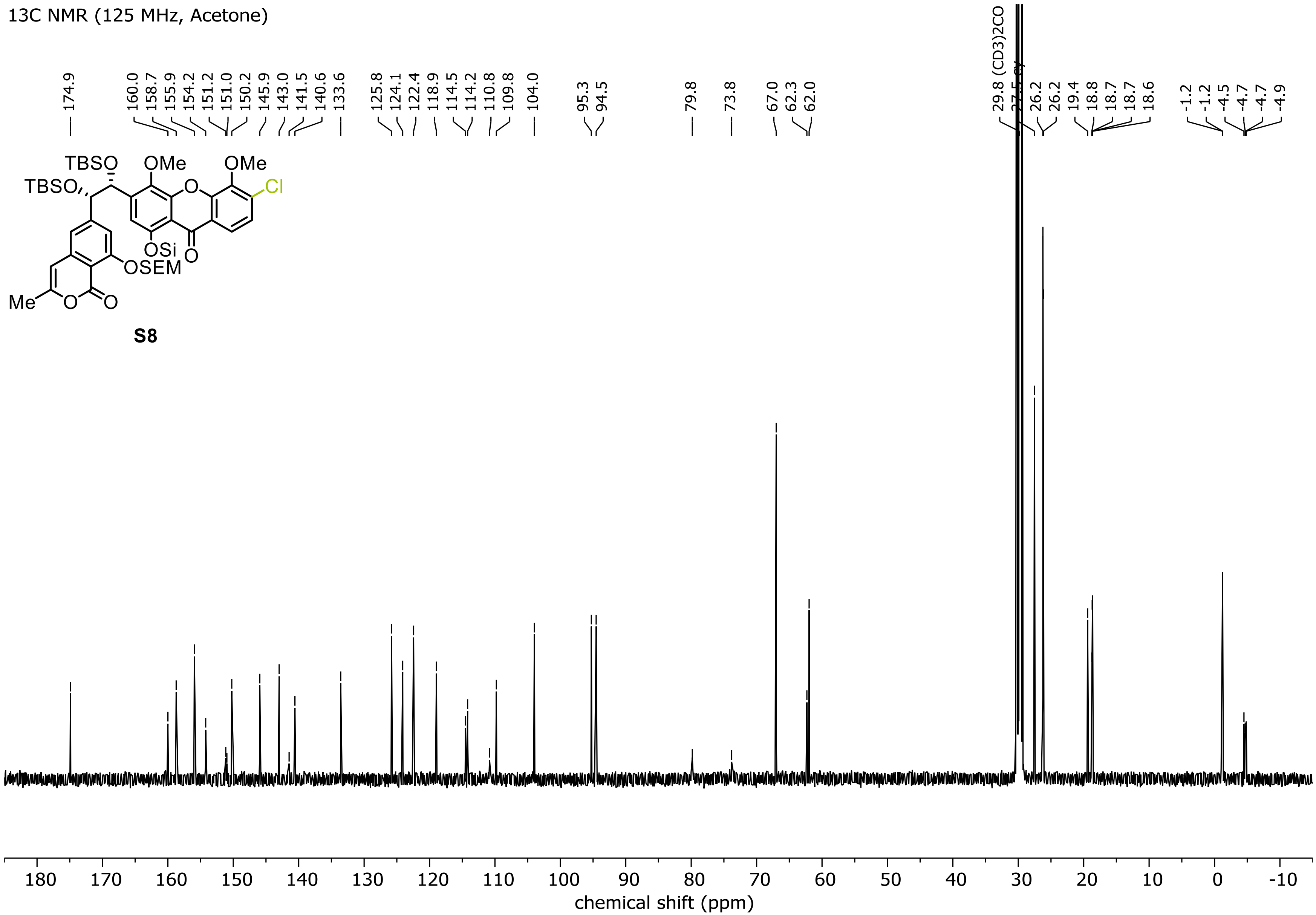

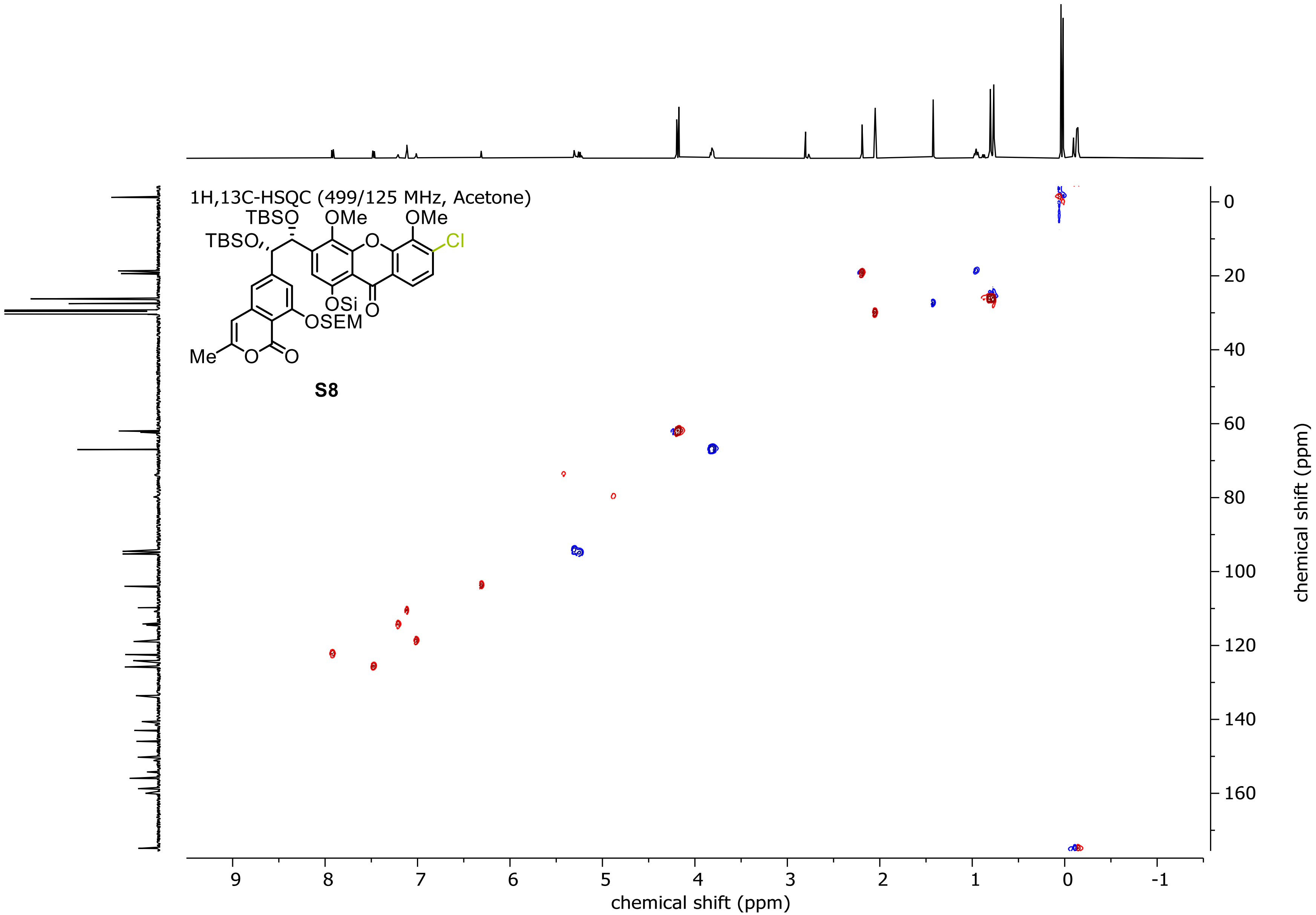
1H,13C-HSQC (499/125 MHz, Acetone)
TBSO
TBSO
OMe
OMe
O
Cl
OSi
O
OSEM
Me
O
O
S8
chemical shift (ppm)
chemical shift (ppm)

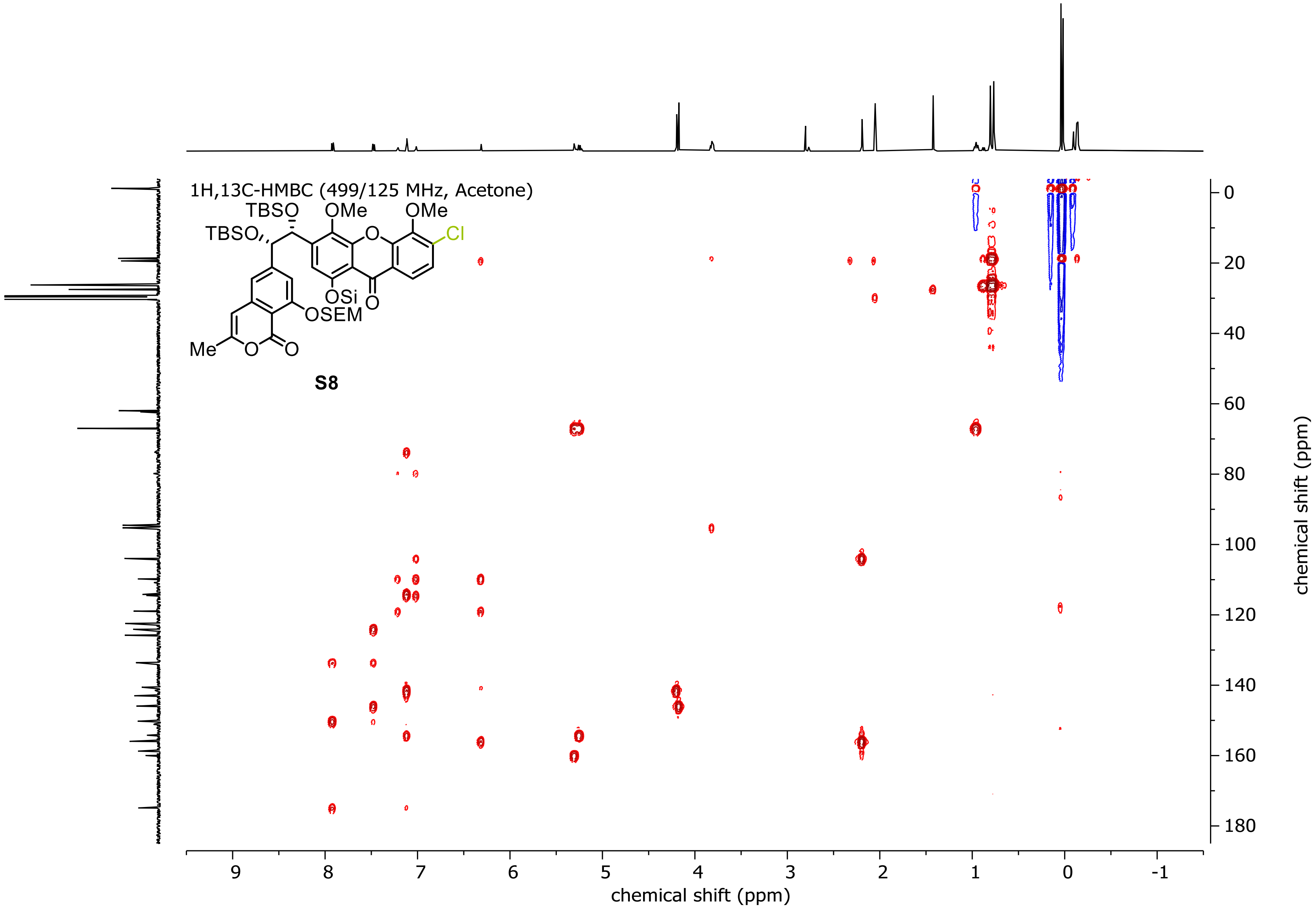
1H,13C-HMBC (499/125 MHz, Acetone)
TBSO
TBSO
OMe
OMe
O
Cl
OSi
O
OSEM
Me
O
O
S8
chemical shift (ppm)
chemical shift (ppm)

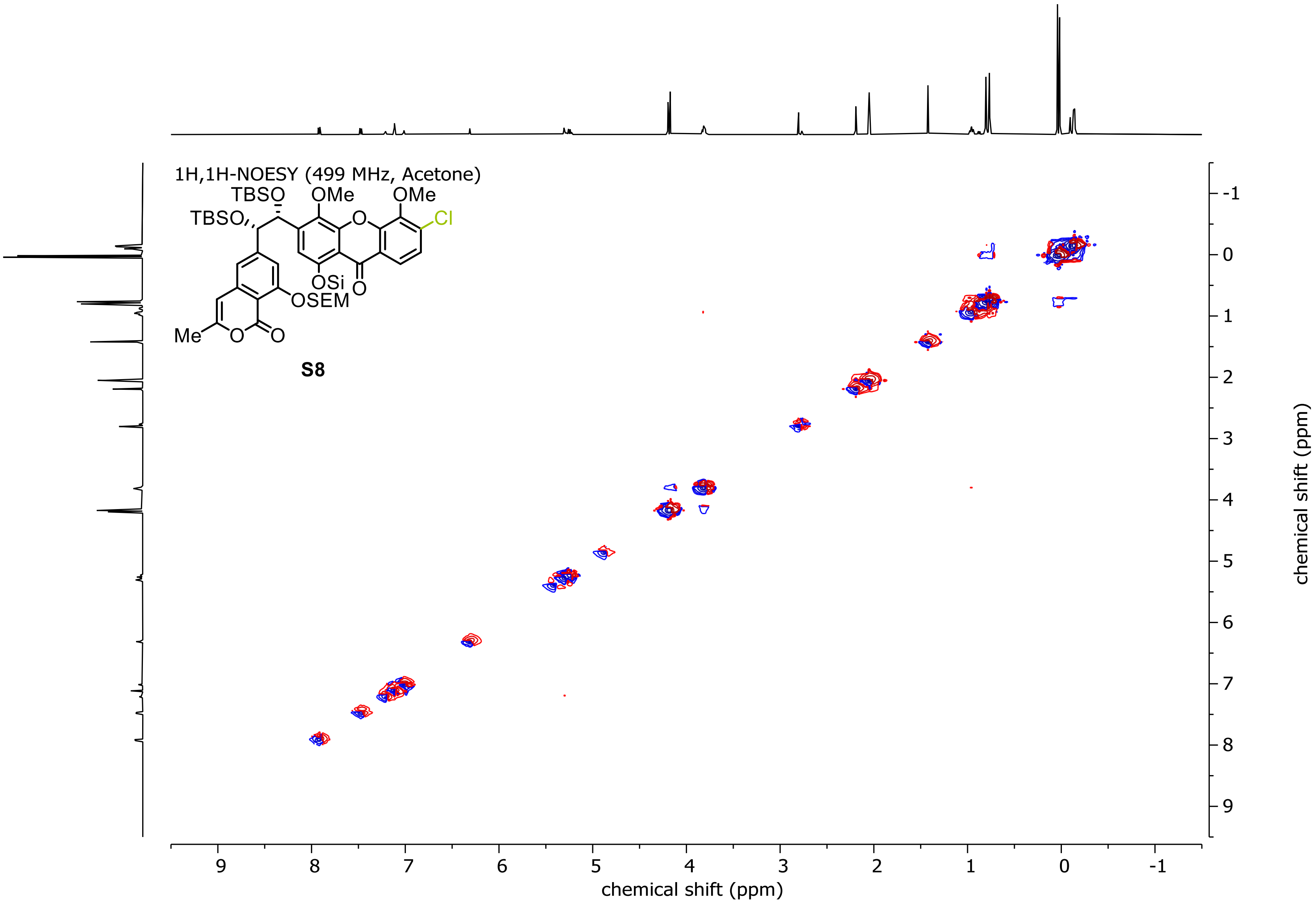
1H,1H-NOESY (499 MHz, Acetone)
TBSO
TBSO
OMe
OMe
Cl
OSi
O
OSEM
Me
O
O
S8
chemical shift (ppm)
chemical shift (ppm)

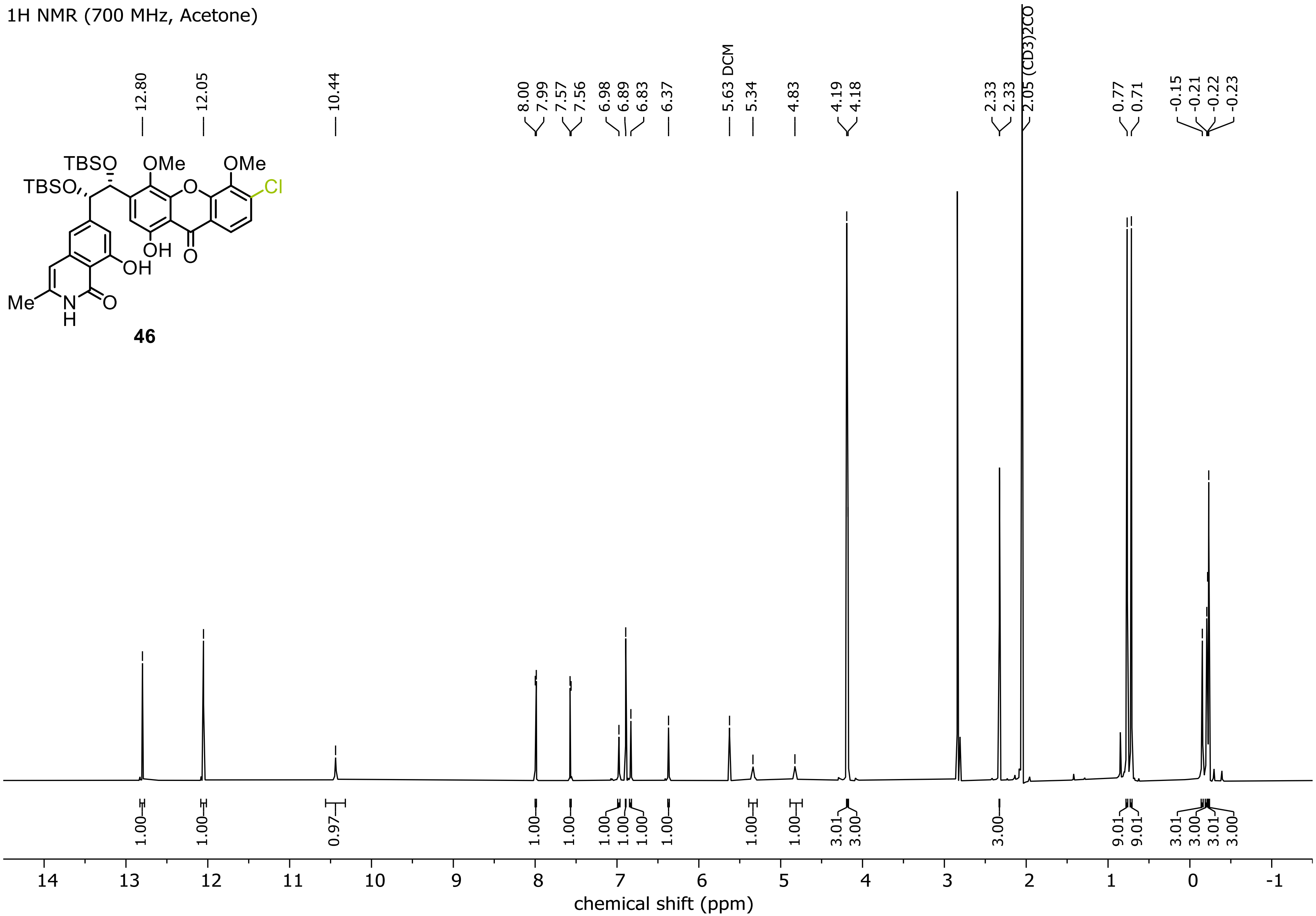
1H NMR (700 MHz, Acetone)
12.80
12.05
10.44
8.00
7.99
7.57
7.56
6.98
6.89
6.83
6.37
5.63 DCM
5.34
4.83
4.19
4.18
2.33
2.33
2.05 (CD3)2CO
0.77
0.71
-0.15
-0.21
-0.22
-0.23
TBSO
TBSO
OMe
OMe
O
Cl
OH
O
OH
Me
N
H
O
46
1.00
1.00
0.97
1.00
1.00
1.00
1.00
1.00
1.00
1.00
1.00
3.01
3.00
3.00
9.01
9.01
3.01
3.00
3.01
3.00
14
13
12
11
10
9
8
7
6
5
4
3
2
1
0
-1
chemical shift (ppm)

13C NMR (176 MHz, Acetone)

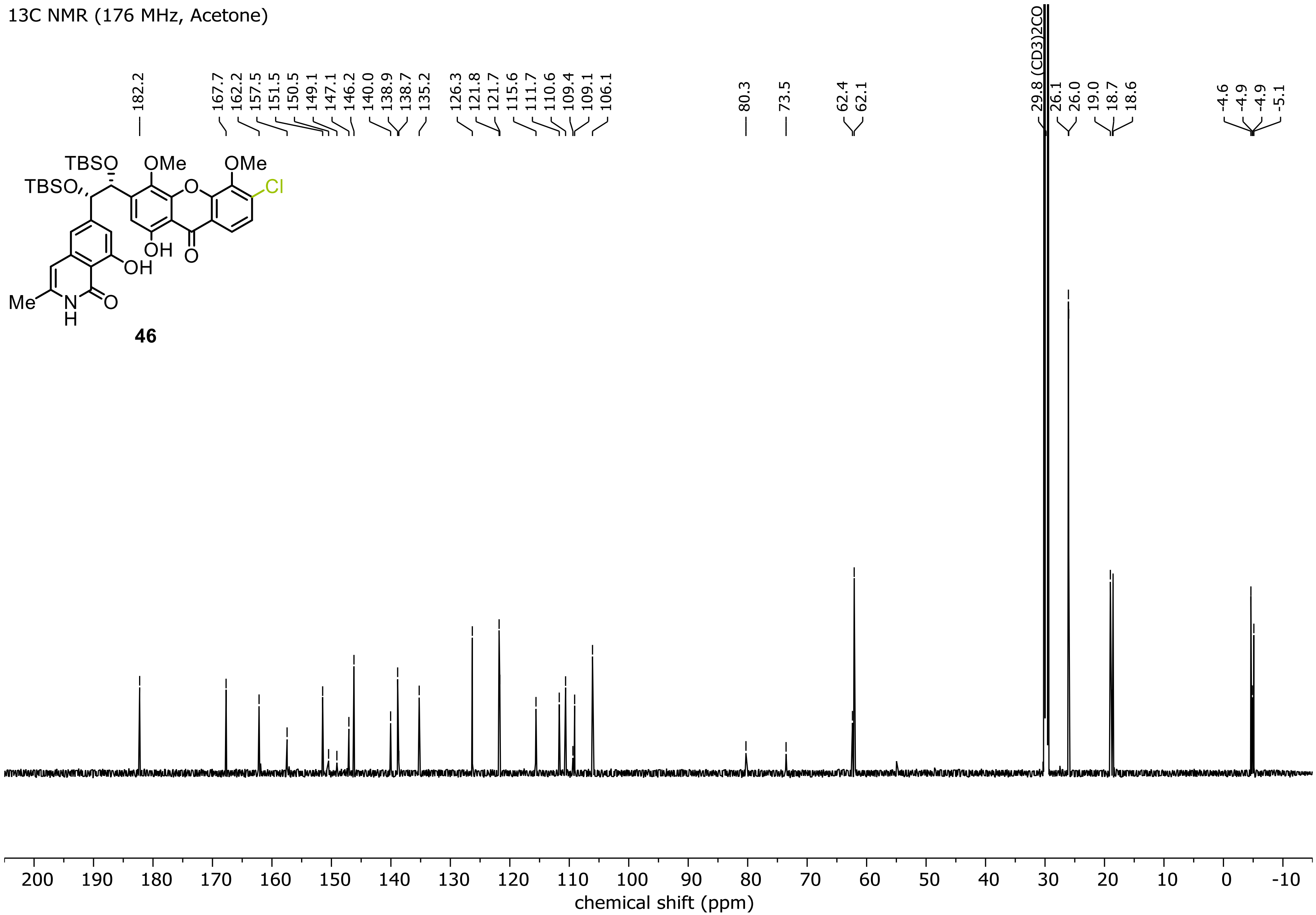

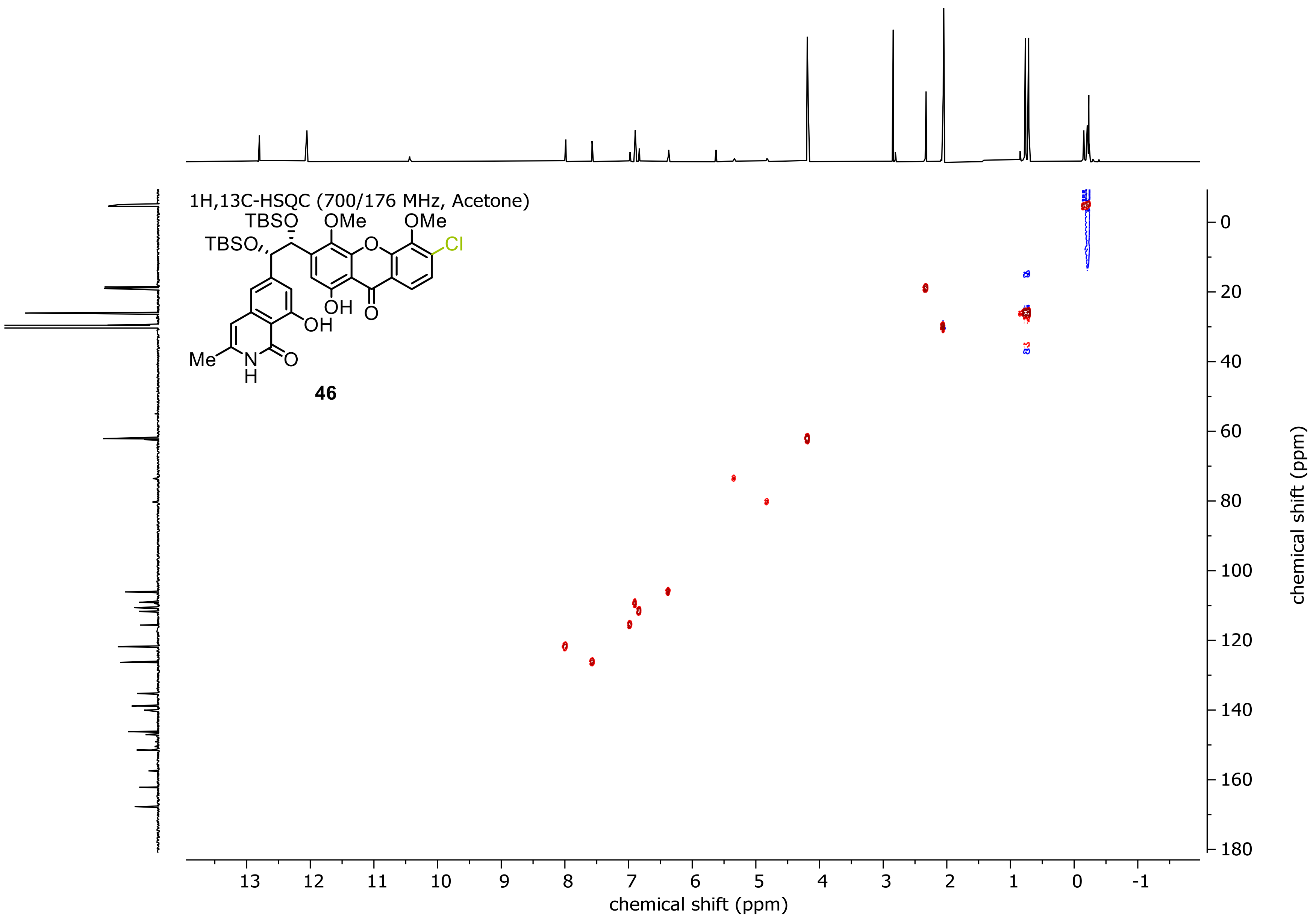
1H,13C-HSQC (700/176 MHz, Acetone)
TBSO
TBSO
OMe
OMe
O
Cl
OH
OH
O
Me
N
H
O
46
chemical shift (ppm)
chemical shift (ppm)

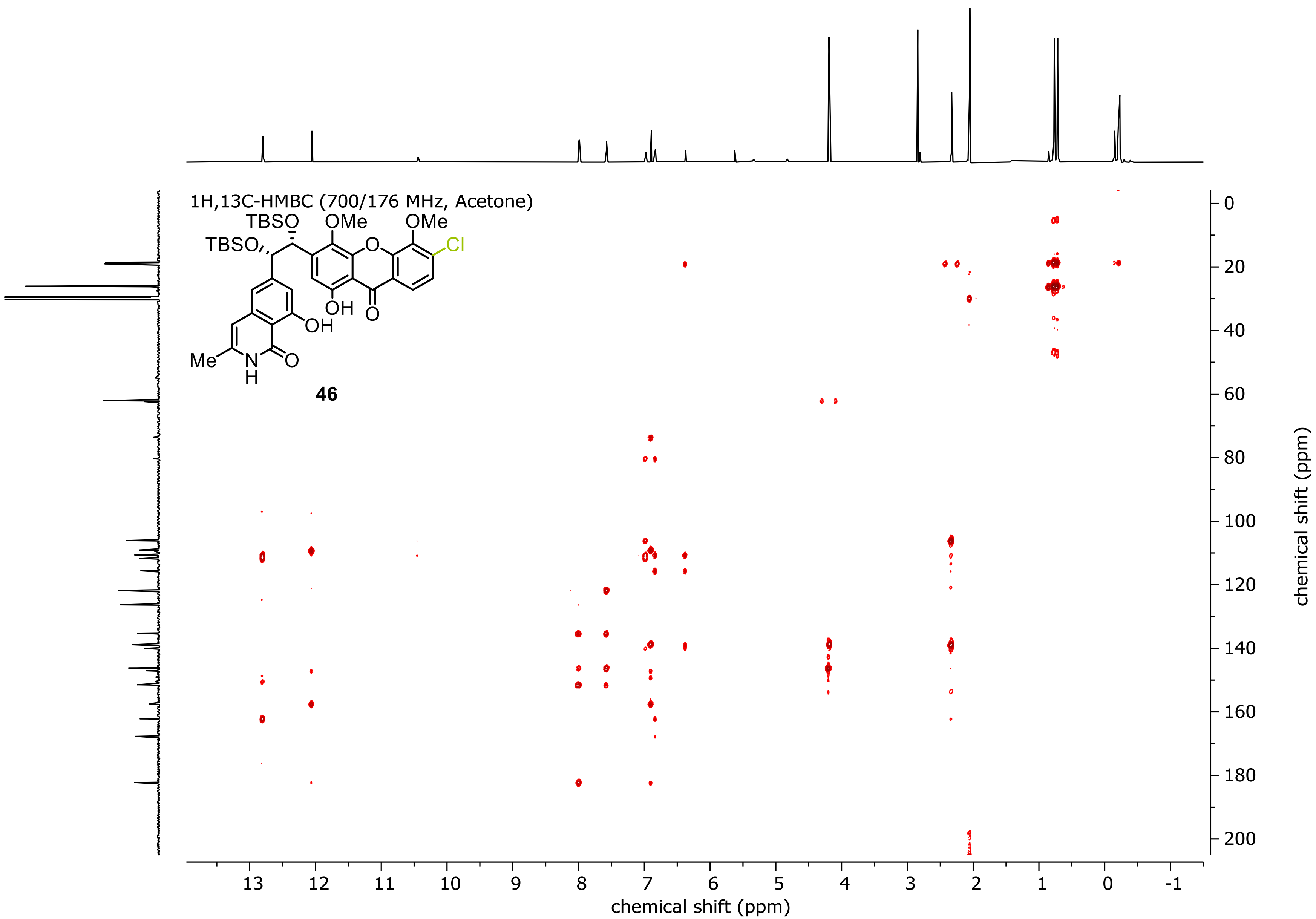
1H,13C-HMBC (700/176 MHz, Acetone)
TBSO
OMe
OMe
TBSO
O
Cl
OH
O
OH
Me
N
H
O
46
chemical shift (ppm)
chemical shift (ppm)

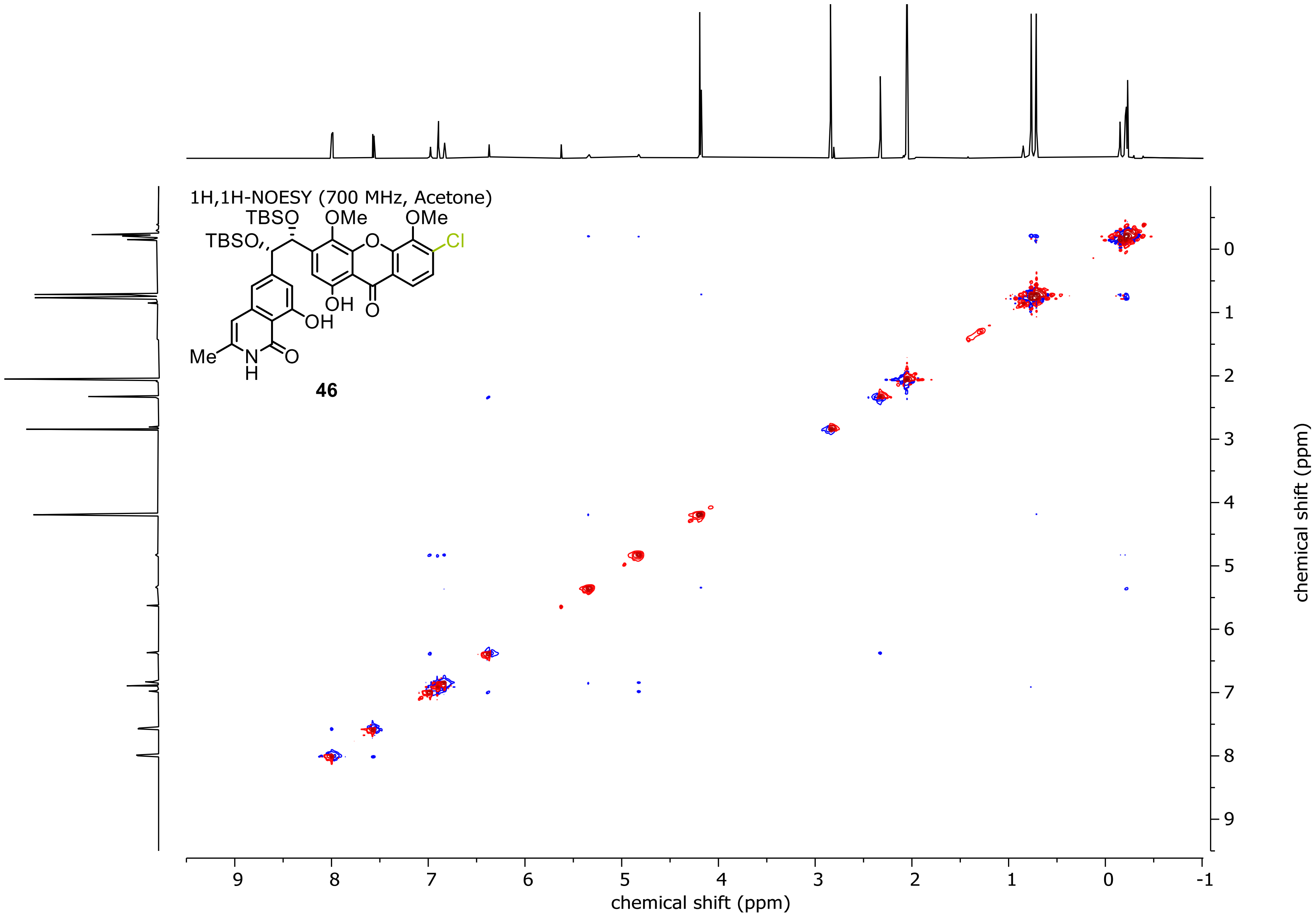
1H,1H-NOESY (700 MHz, Acetone)
TBSO
TBSO
OMe
OMe
O
Cl
OH
OH
O
Me
N
H
O
46
chemical shift (ppm)
chemical shift (ppm)

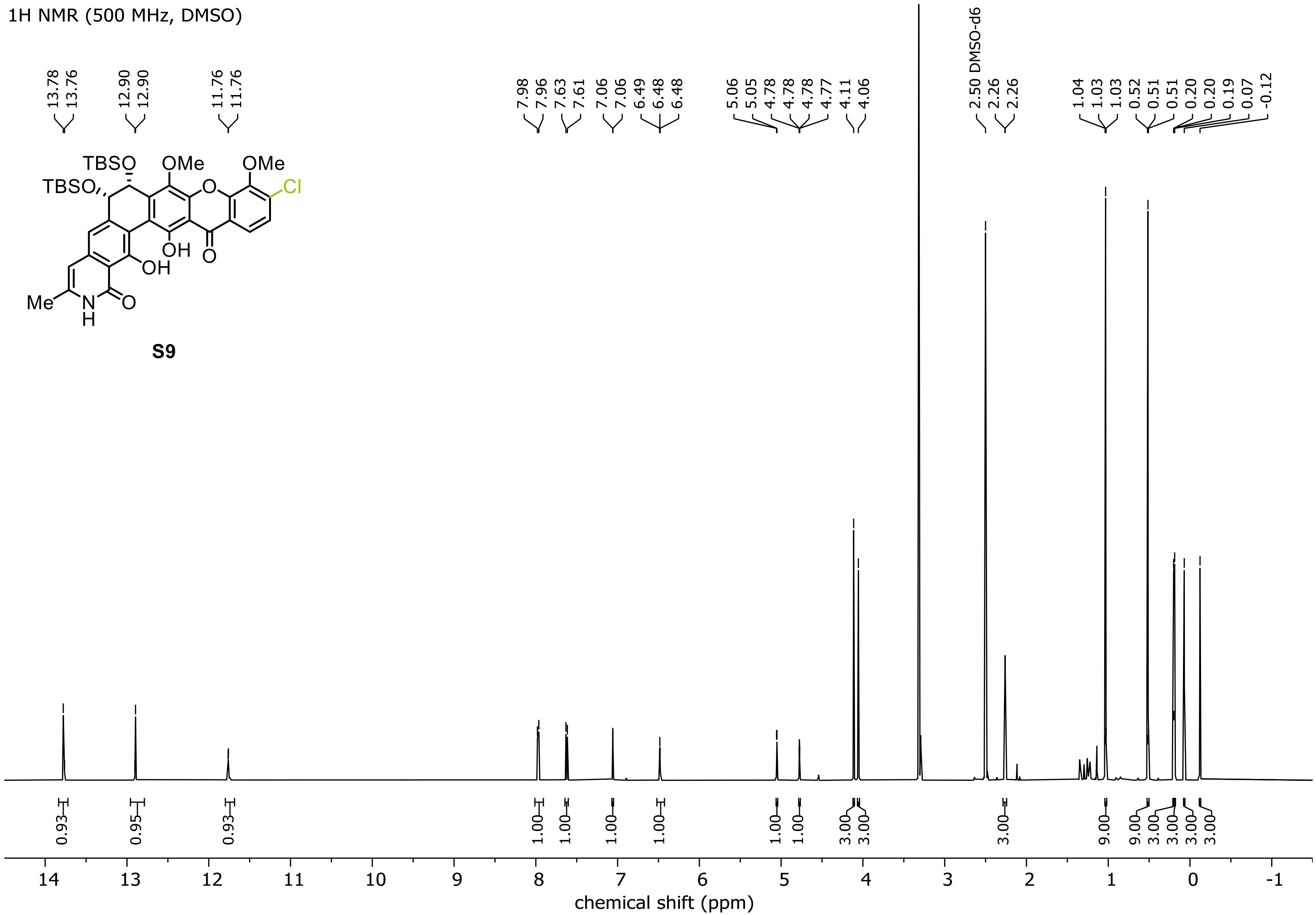
1H NMR (500 MHz, DMSO)
13.78 13.76 12.90 12.90 11.76 11.76 7.98 7.96 7.63 7.61 7.06 7.06 6.49 6.48 6.48 5.06 5.05 4.78 4.78 4.78 4.77 4.11 4.06 2.50 DMSO-d6 2.26 2.26 1.04 1.03 1.03 0.52 0.51 0.51 0.20 0.20 0.19 0.07 -0.12
TBSO
OMe
OMe
TBSO
Cl
OH
OH
O
O
Me
N
H
O
S9
0.93 0.95 0.93 1.00 1.00 1.00 1.00 1.00 1.00 3.00 3.00 3.00 9.00 9.00 3.00 3.00 3.00 3.00
14 13 12 11 10 9 8 7 6 5 4 3 2 1 0 -1
chemical shift (ppm)

13C NMR (125 MHz, DMSO)

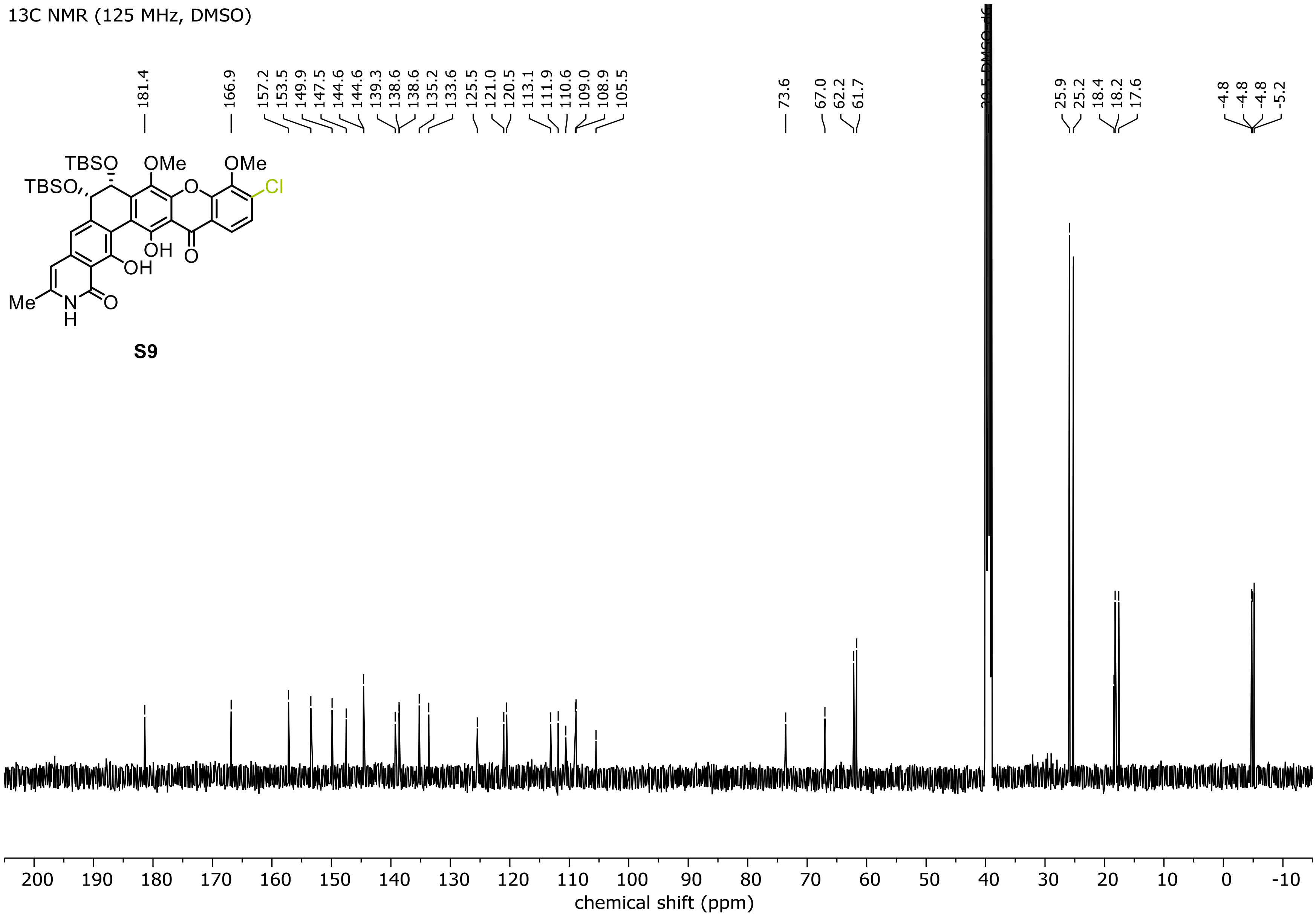

S9

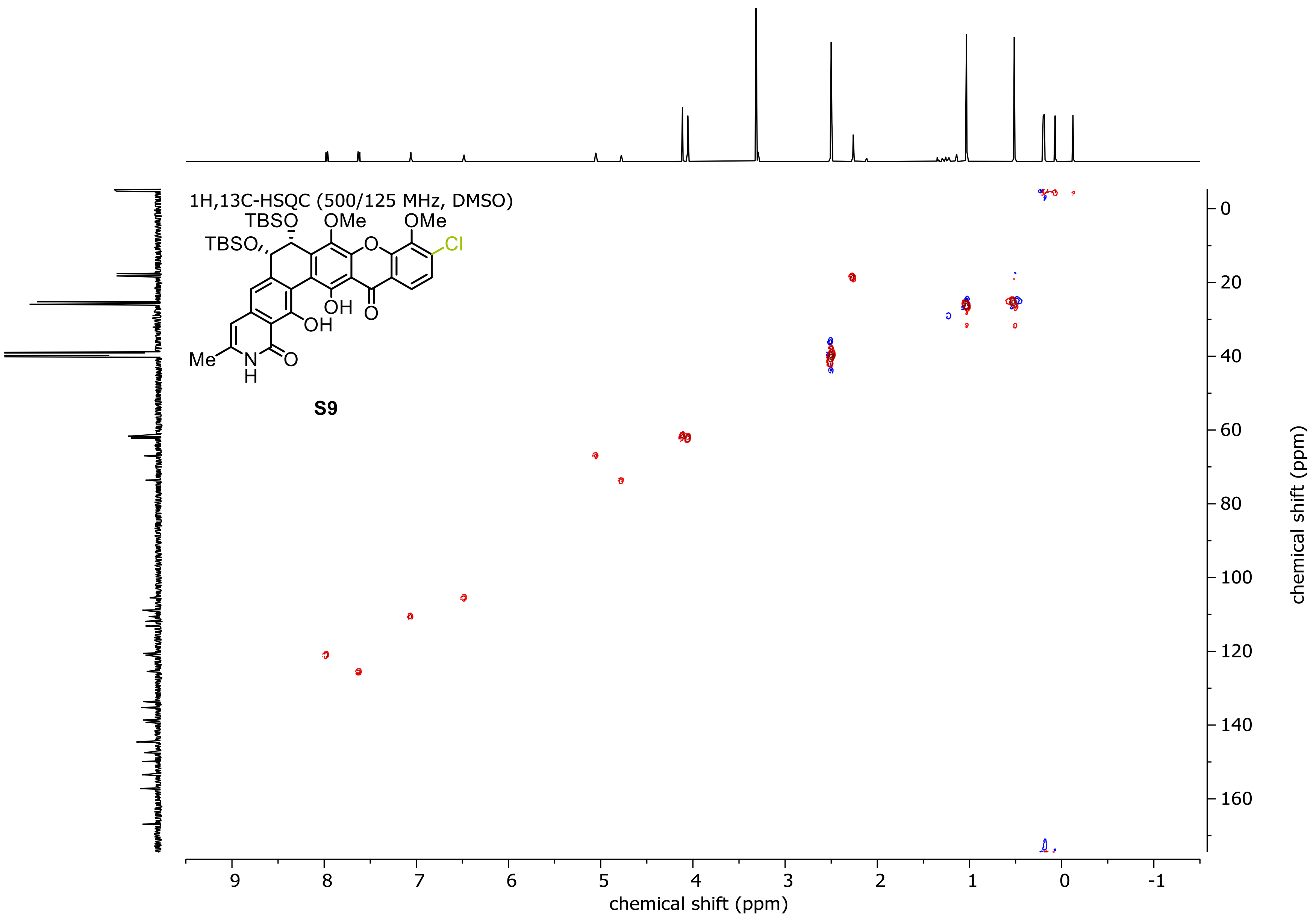
1H,13C-HSQC (500/125 MHz, DMSO)
TBSO
OMe
OMe
TBSO
O
Cl
OH
O
OH
Me
N
H
O
S9
0
20
40
60
80
100
120
140
160
chemical shift (ppm)
9
8
7
6
5
4
3
2
1
0
-1
chemical shift (ppm)

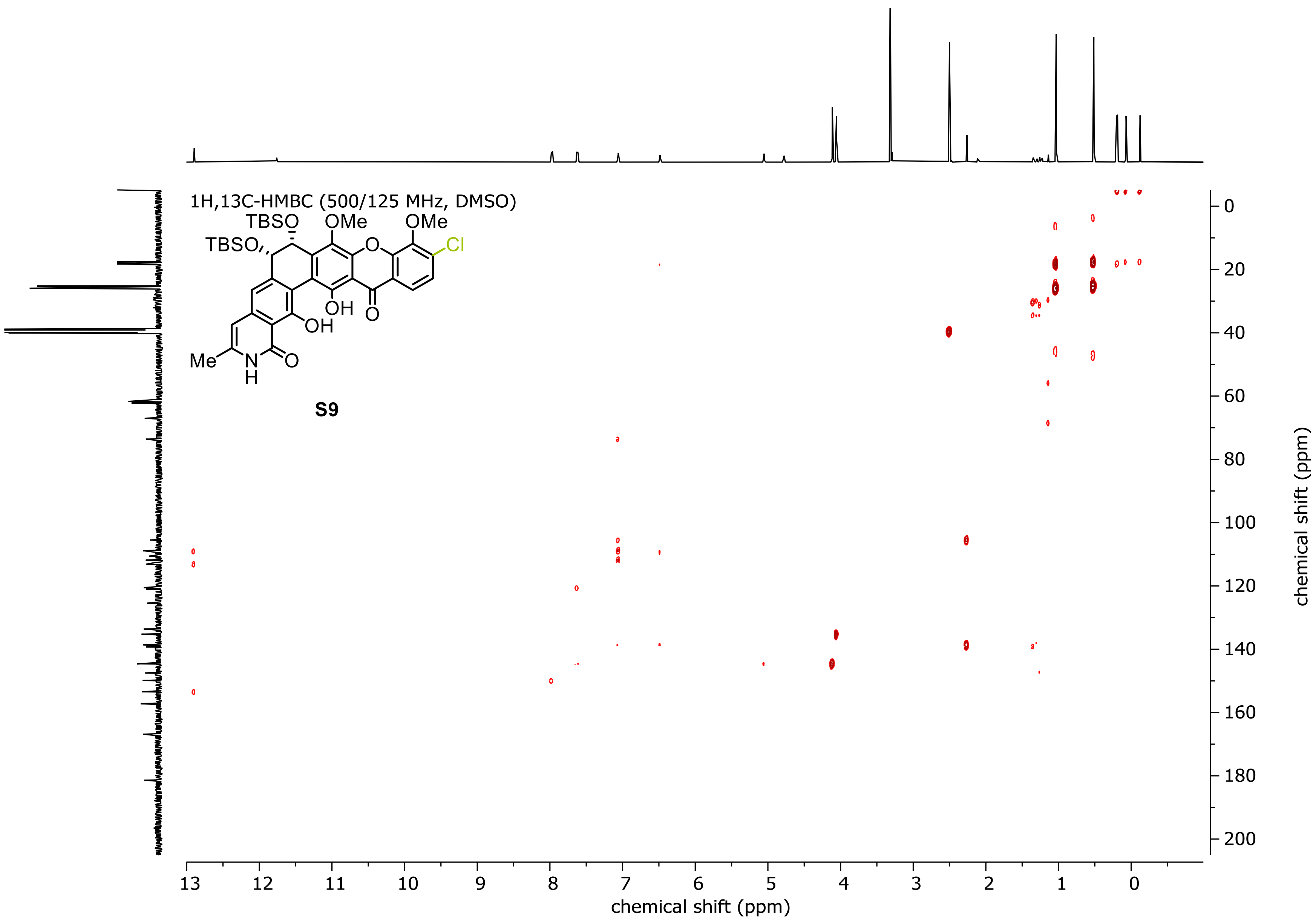
1H,13C-HMBC (500/125 MHz, DMSO)
TBSO
TBSO
OMe
OMe
Cl
OH
OH
O
O
Me
N
H
S9
chemical shift (ppm)
chemical shift (ppm)

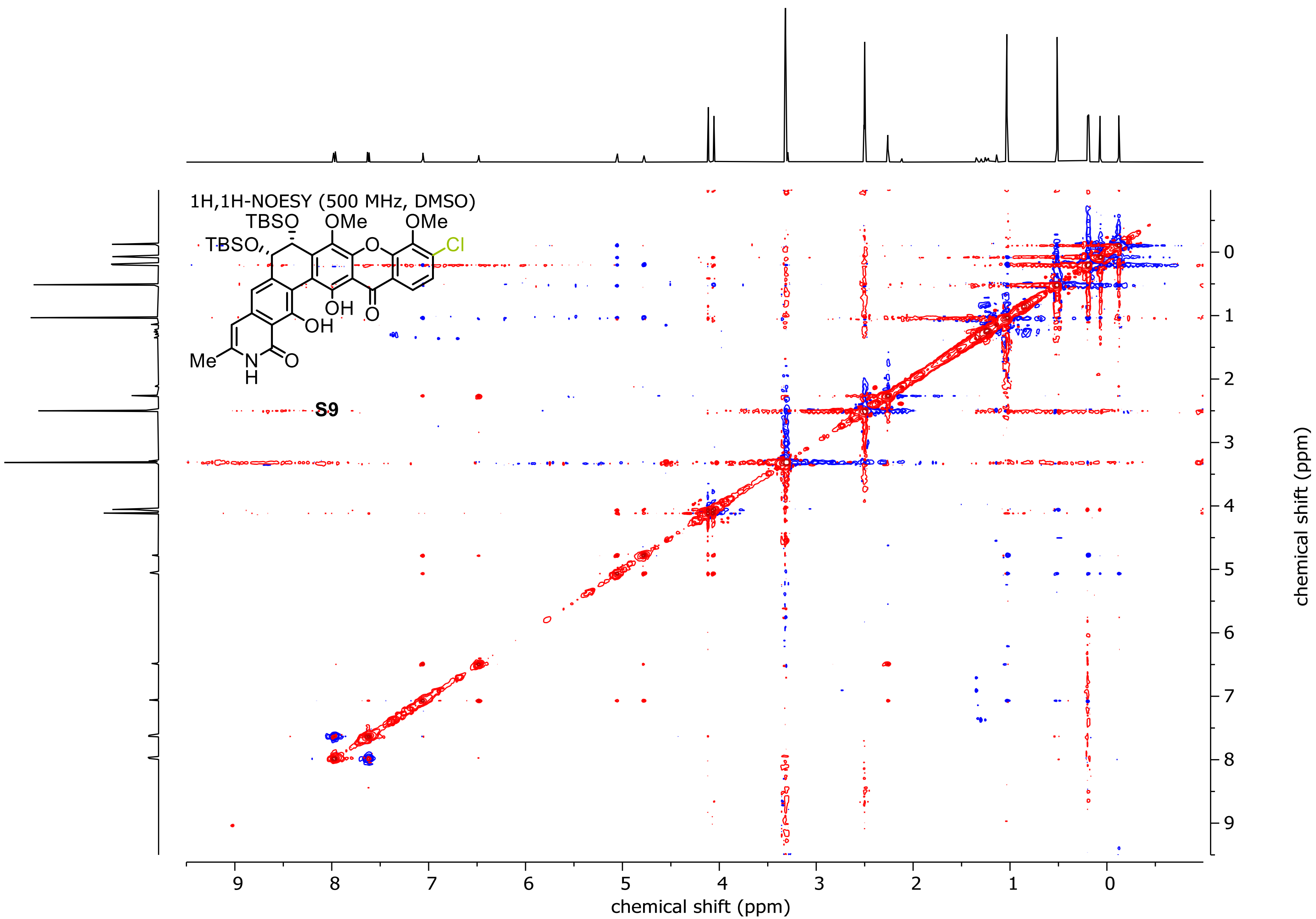
1H,1H-NOESY (500 MHz, DMSO)
TBSO
TBSO
OMe
OMe
O
Cl
OH
OH
O
Me
N
H
O
S9
chemical shift (ppm)
chemical shift (ppm)

1H NMR (700 MHz, DMSO)

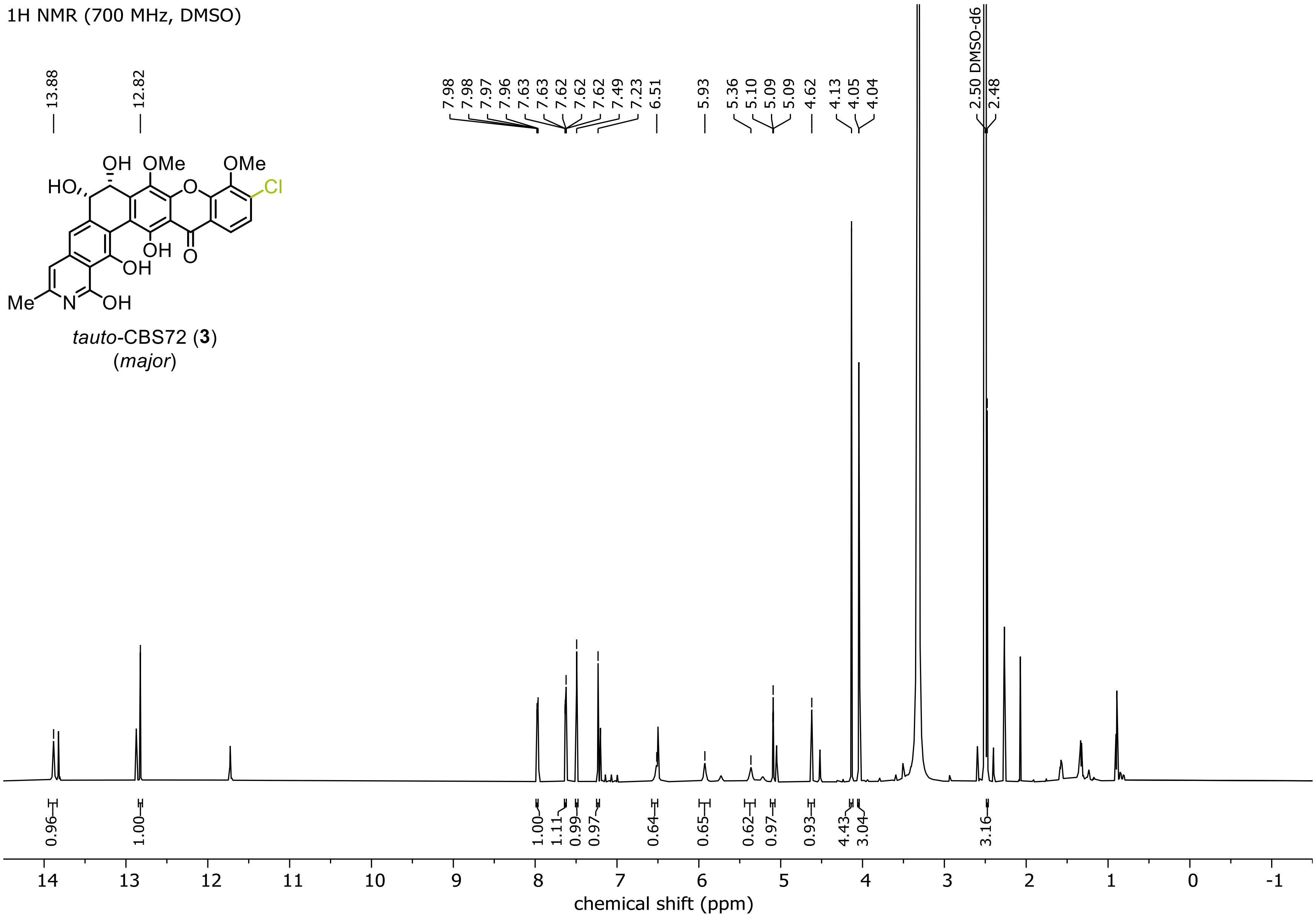

tauto-CBS72 (**3**)
(*major*)

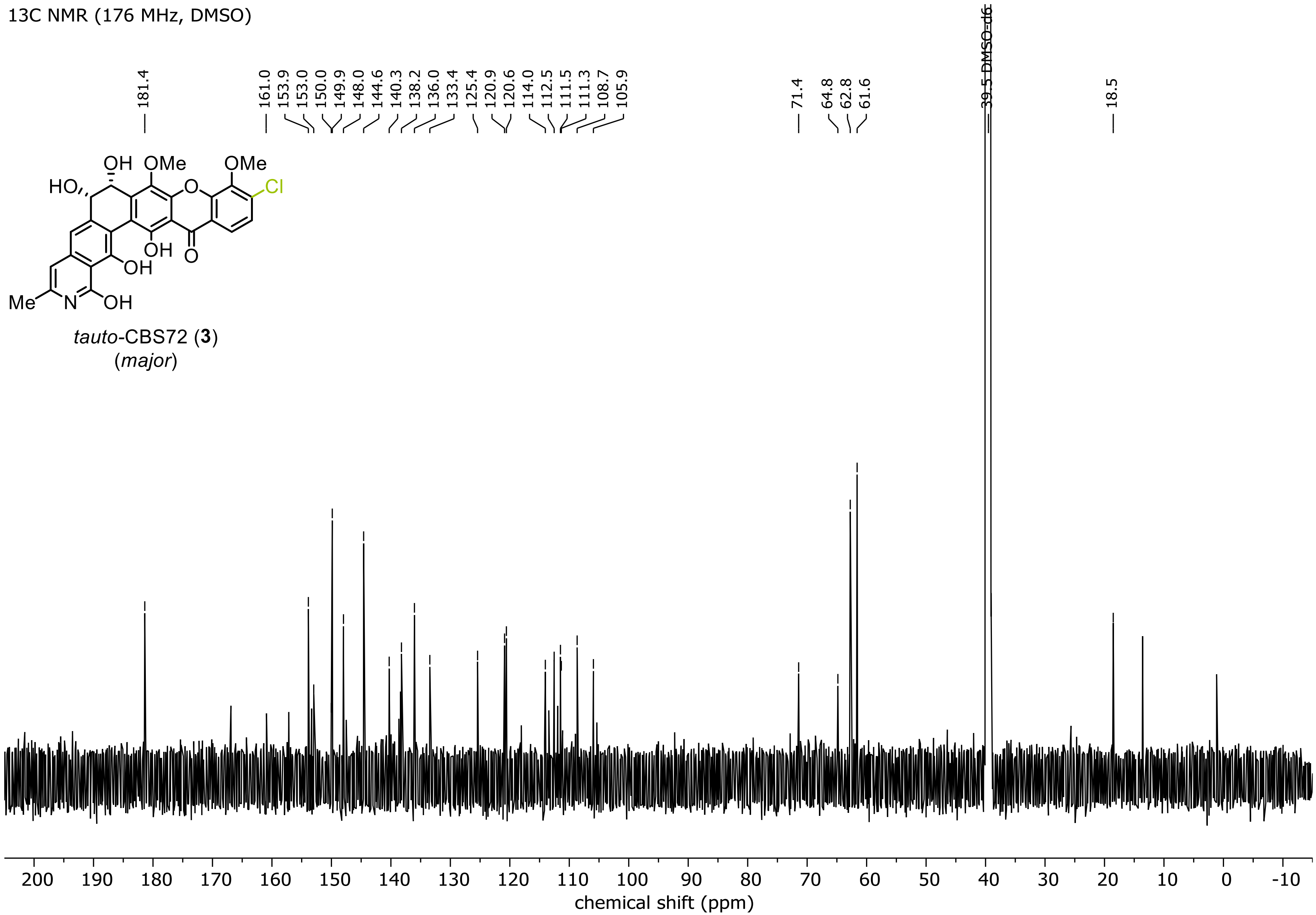
13C NMR (176 MHz, DMSO)
181.4
161.0
153.9
153.0
150.0
149.9
148.0
144.6
140.3
138.2
136.0
133.4
125.4
120.9
120.6
114.0
112.5
111.5
111.3
108.7
105.9
71.4
64.8
62.8
61.6
39.5 DMSO-d6
18.5
OH
OMe
OMe
HO
O
Cl
OH
O
OH
Me
N
OH
tauto-CBS72 (3)
(major)
chemical shift (ppm)

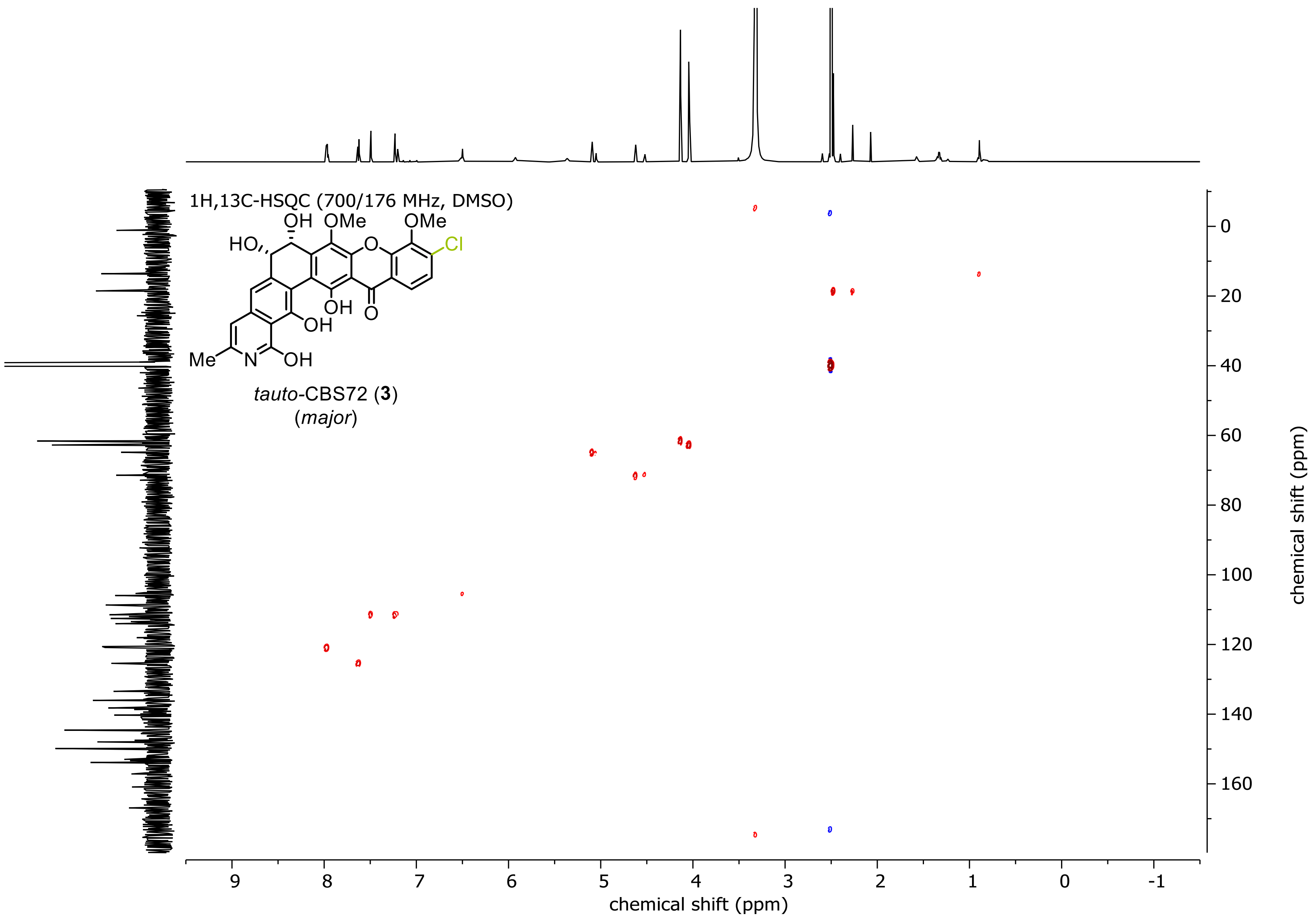
1H,13C-HSQC (700/176 MHz, DMSO)
OH OMe OMe
HO
Cl
OH
O
OH
Me N OH
tauto-CBS72 (3)
(major)
chemical shift (ppm)
chemical shift (ppm)

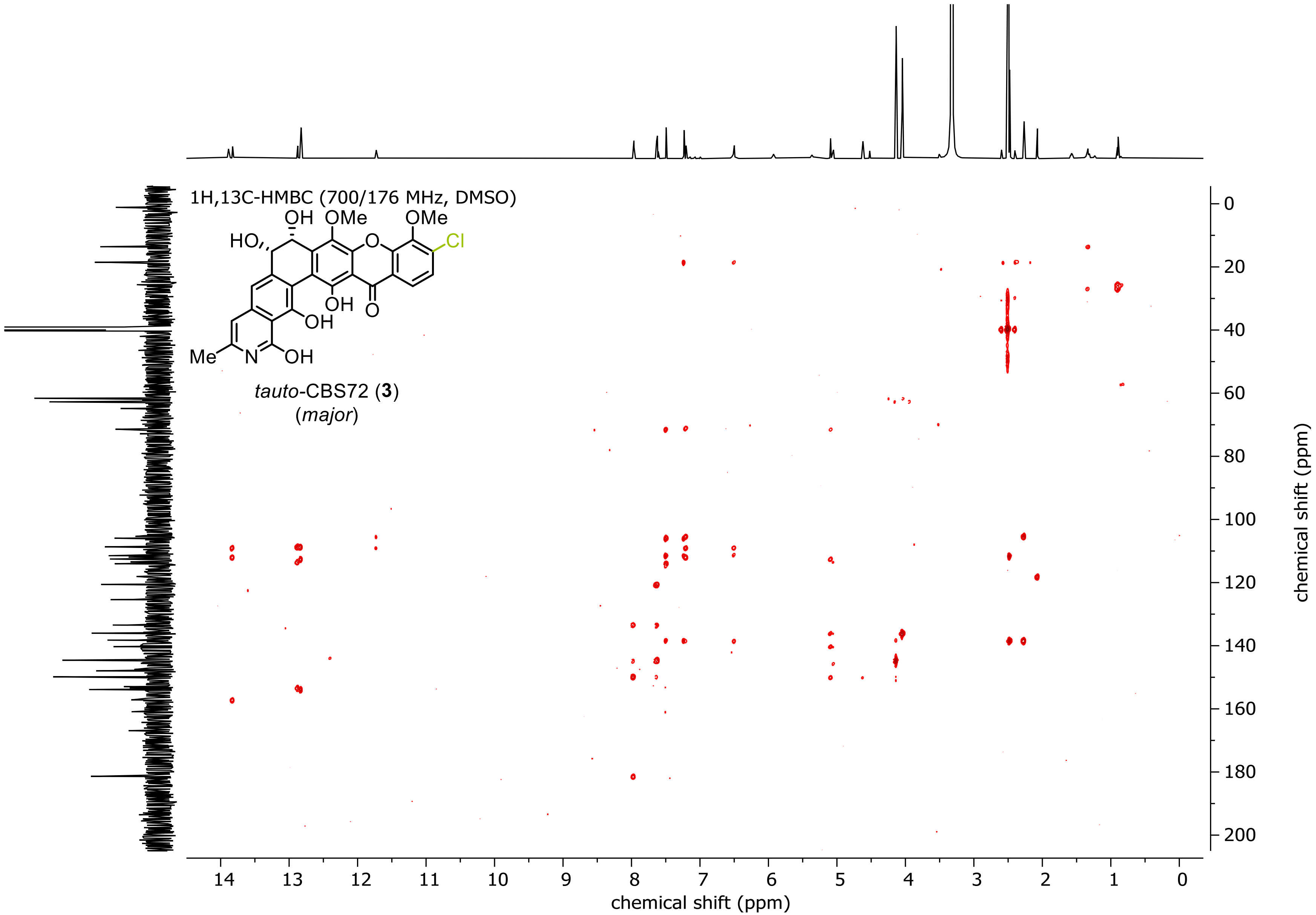
1H,13C-HMBC (700/176 MHz, DMSO)
OH
OMe
OMe
HO
O
Cl
OH
OH
O
Me
N
OH
tauto-CBS72 (**3**)
(*major*)
chemical shift (ppm)
chemical shift (ppm)

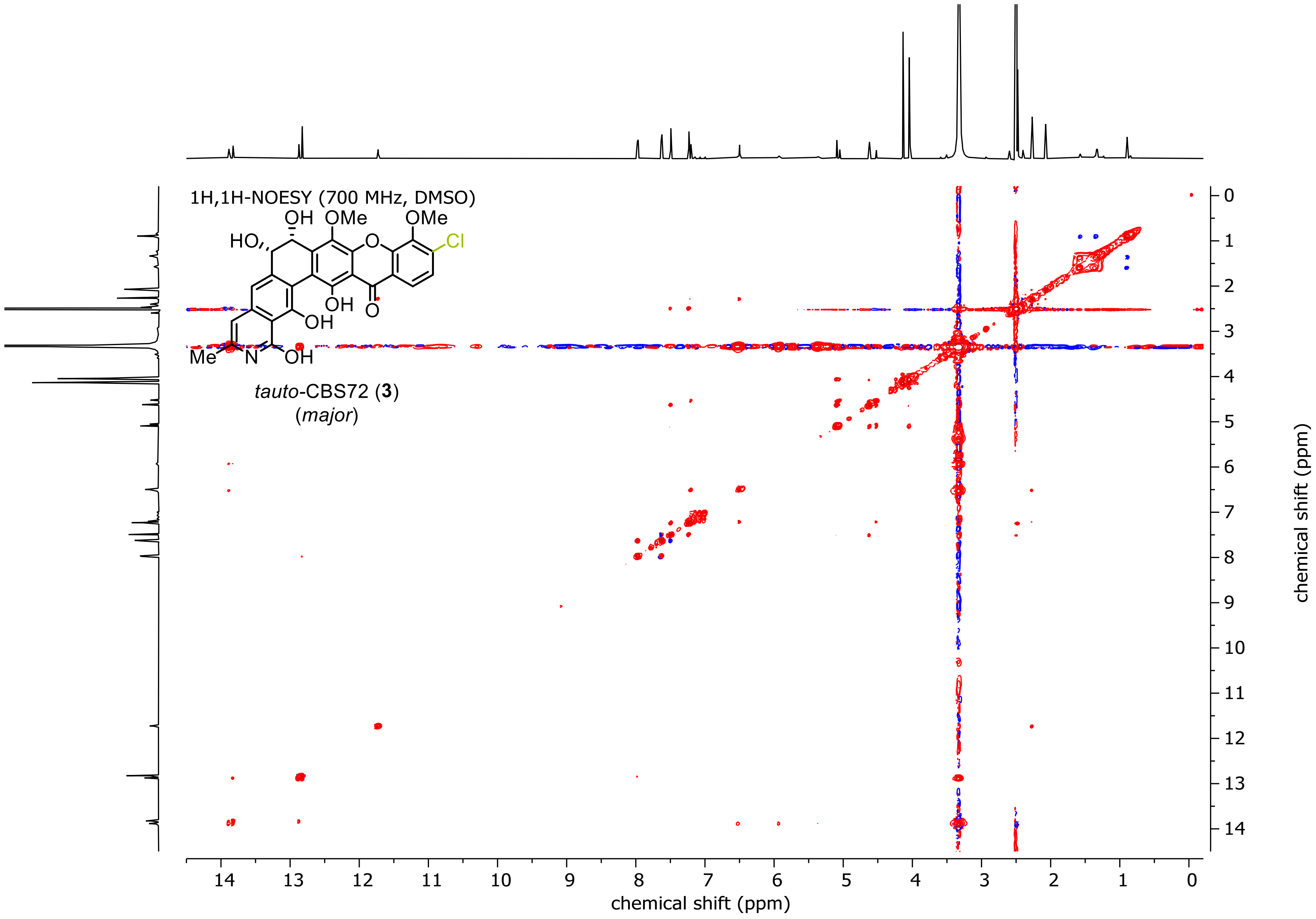
1H,1H-NOESY (700 MHz, DMSO)
OH OMe OMe
HO Cl
OH O
OH
Me N OH
tauto-CBS72 (**3**)
(*major*)
chemical shift (ppm)
chemical shift (ppm)
14 13 12 11 10 9 8 7 6 5 4 3 2 1 0

1H NMR (700 MHz, DMSO)

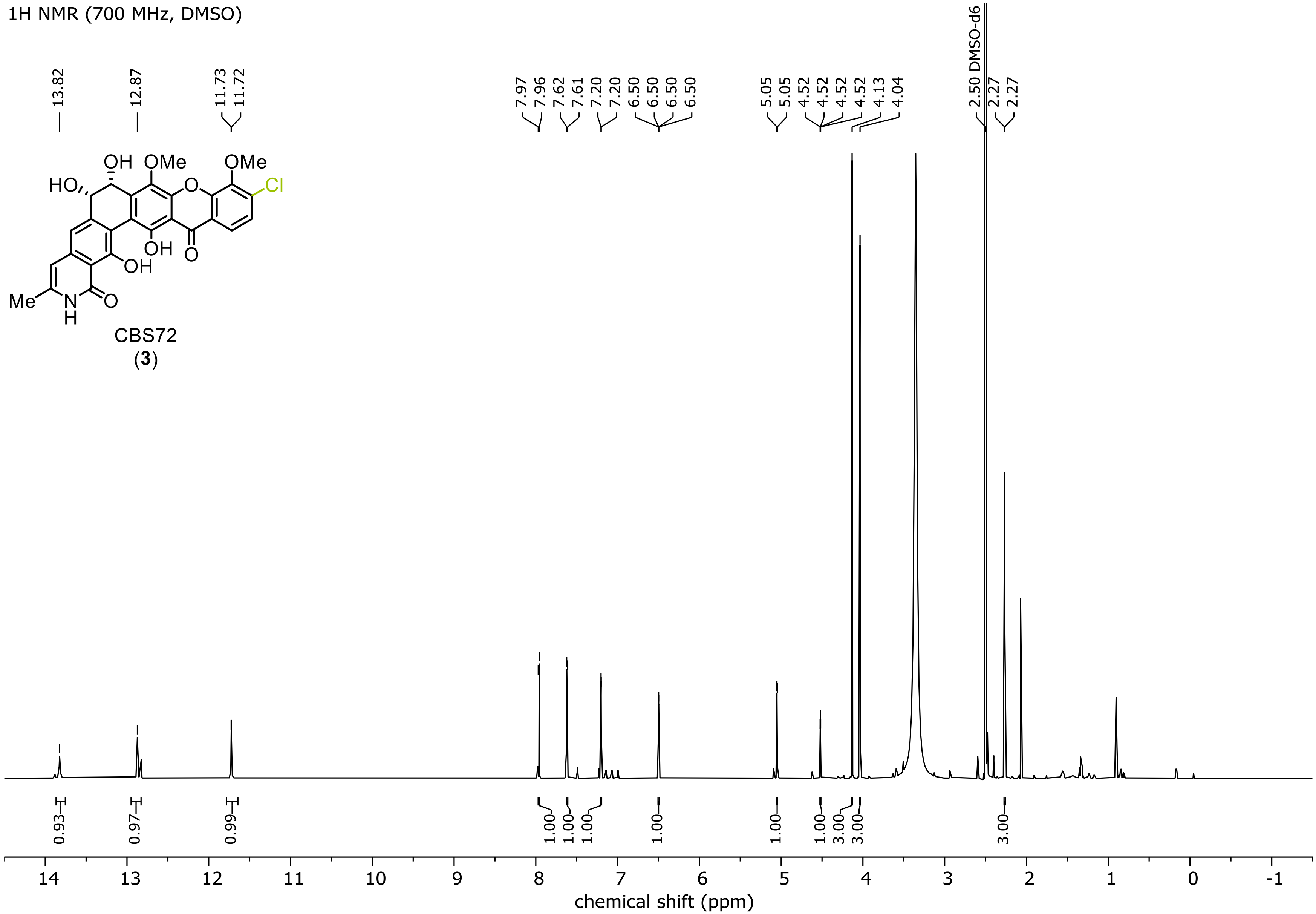

13C NMR (176 MHz, DMSO)

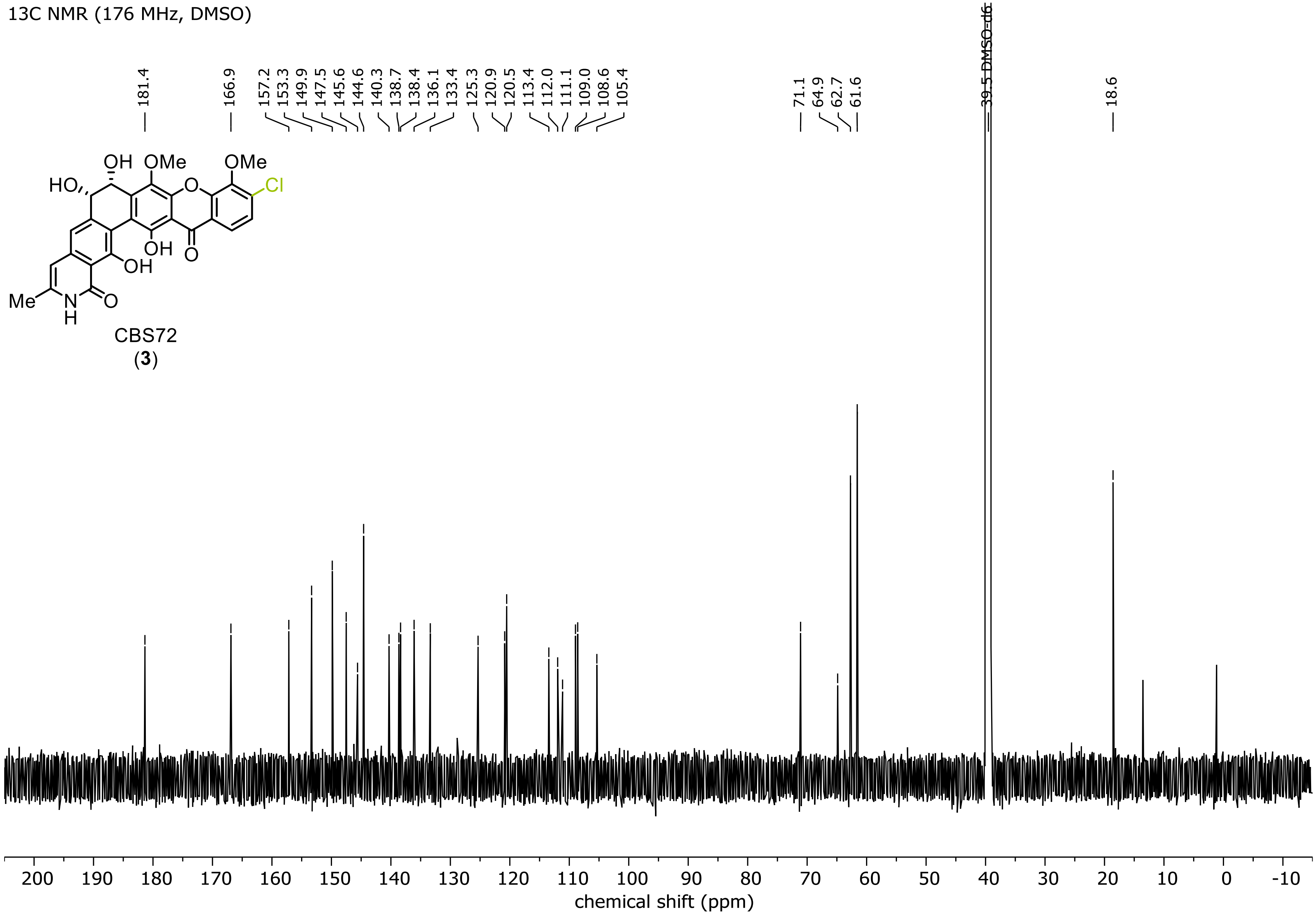

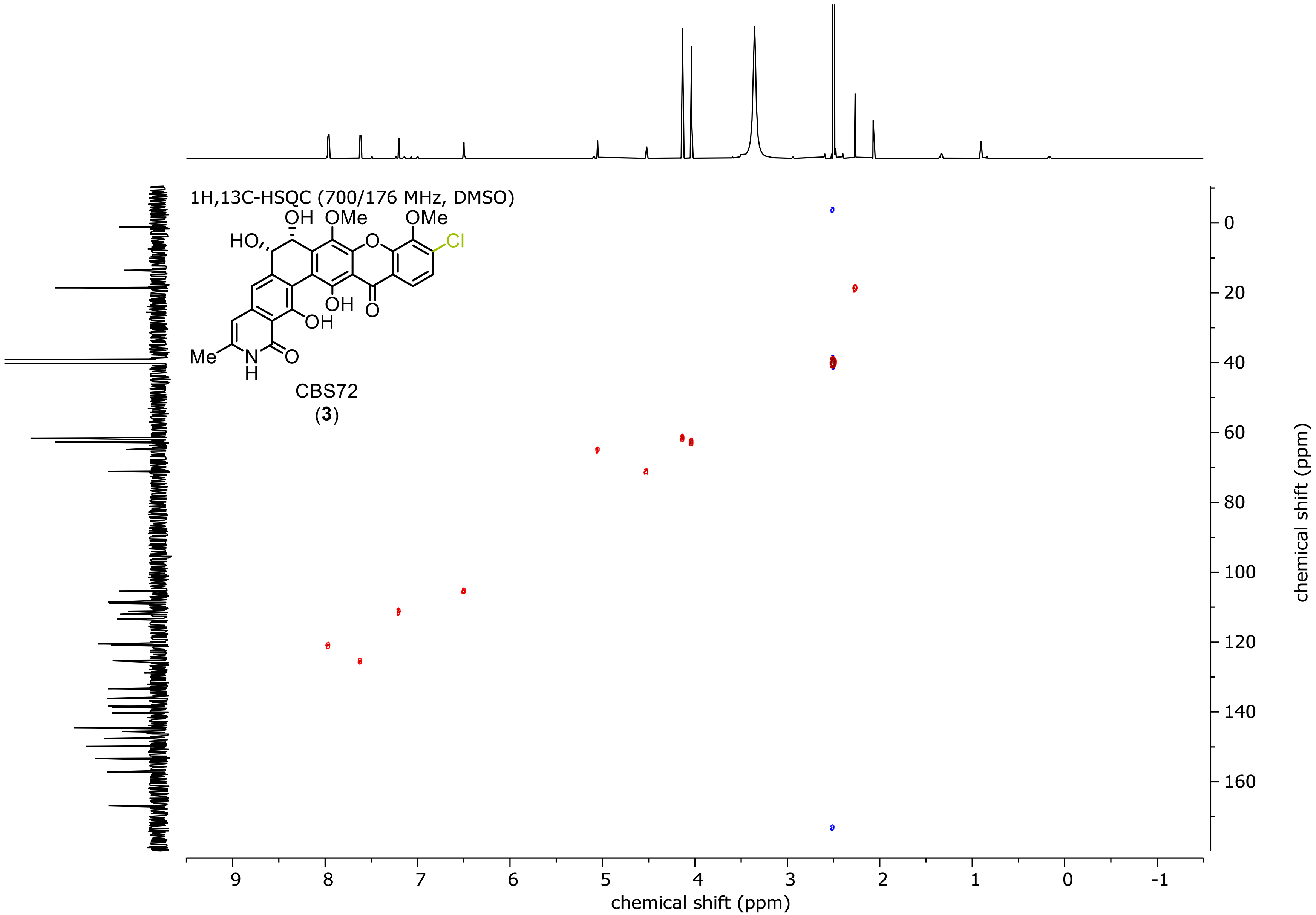
1H,13C-HSQC (700/176 MHz, DMSO)
OH
OMe
OMe
HO
O
Cl
OH
O
OH
Me
N
H
O
CBS72
(3)
chemical shift (ppm)
chemical shift (ppm)

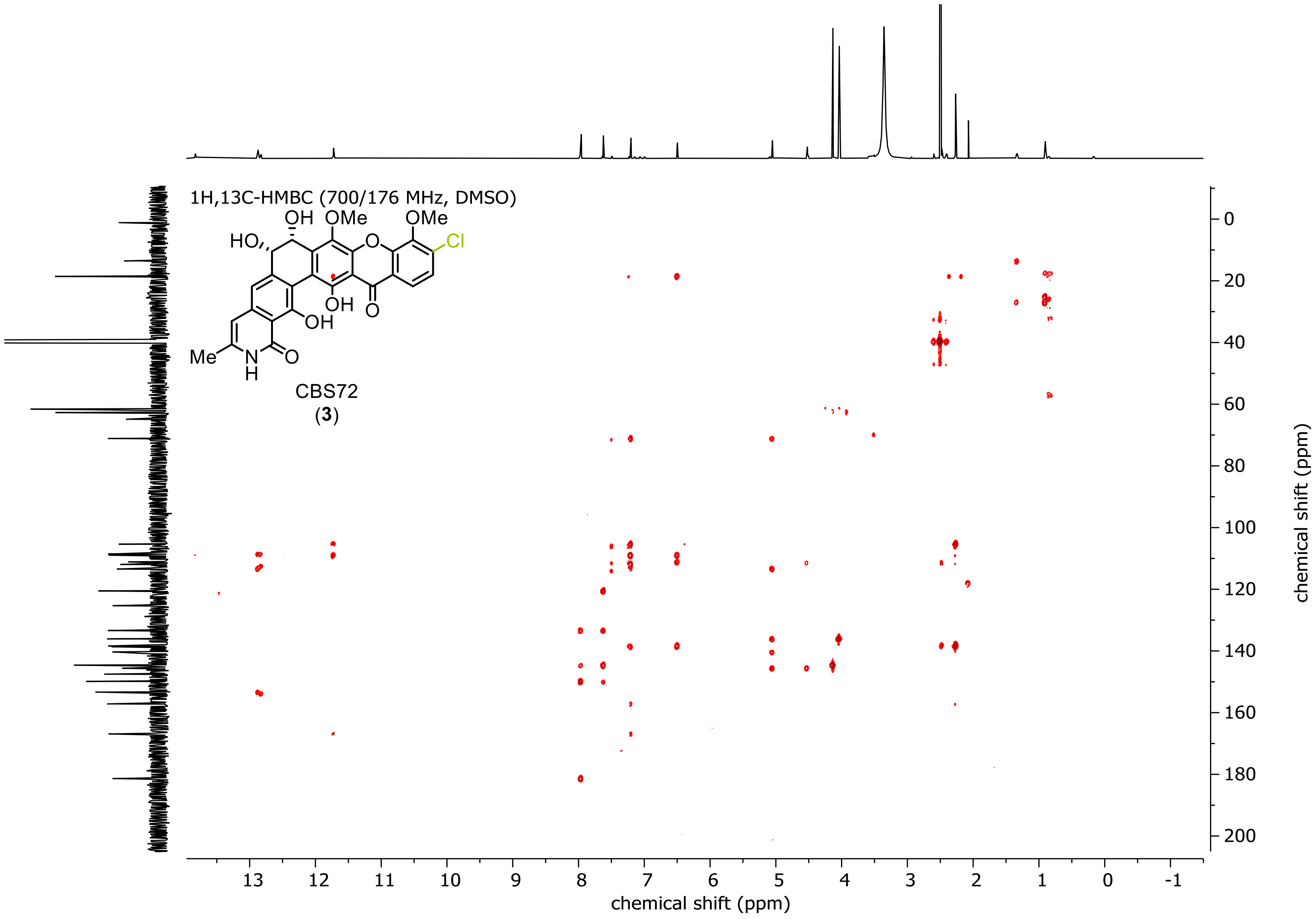
1H,13C-HMBC (700/176 MHz, DMSO)
OH
OMe
OMe
HO
Cl
O
OH
O
OH
Me
N
H
O
CBS72
(3)
chemical shift (ppm)
chemical shift (ppm)

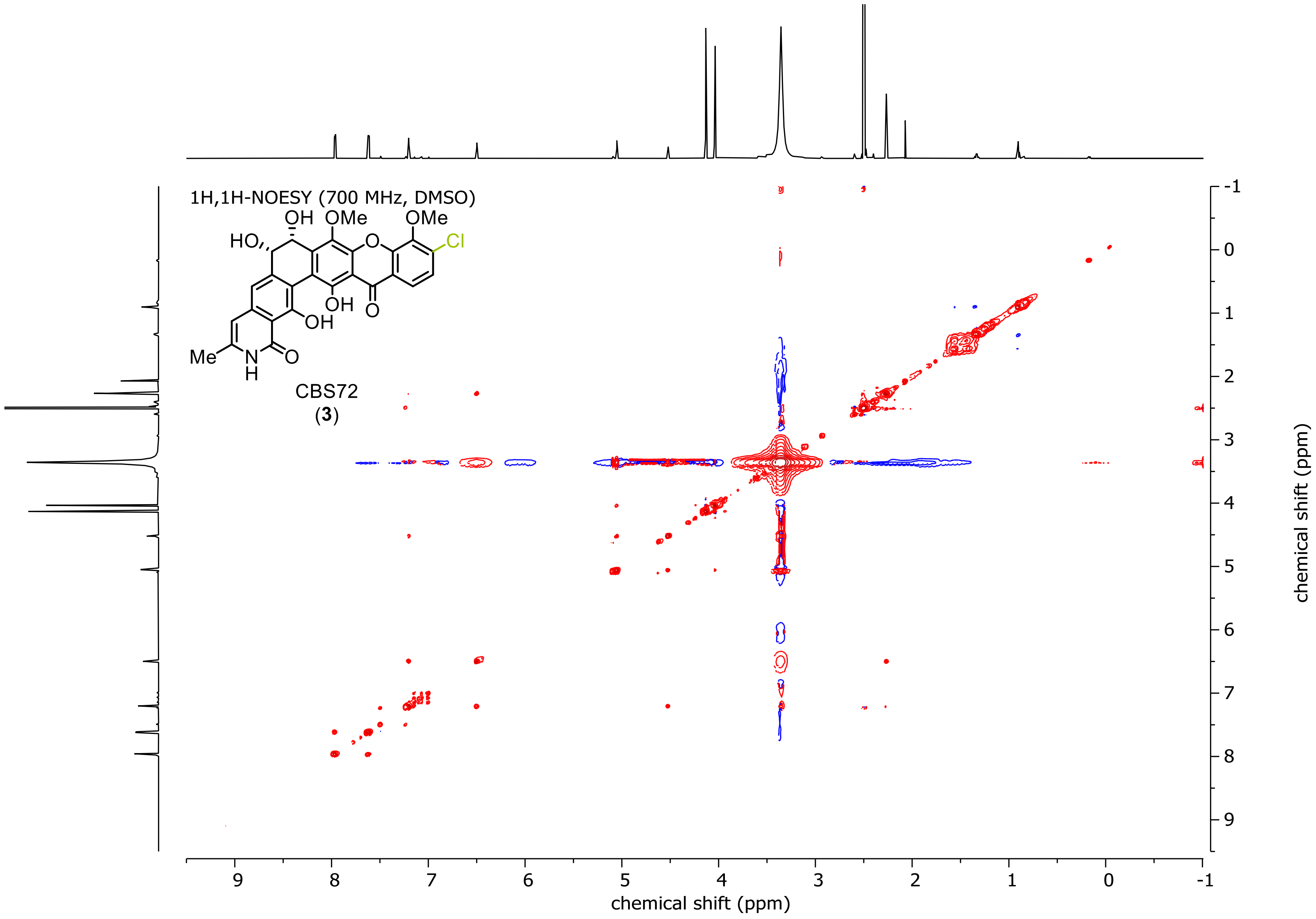

1H,1H-NOESY (700 MHz, DMSO)
OH
OMe
OMe
HO
O
Cl
OH
O
OH
Me
N
H
O
CBS72
(3)
chemical shift (ppm)
chemical shift (ppm)

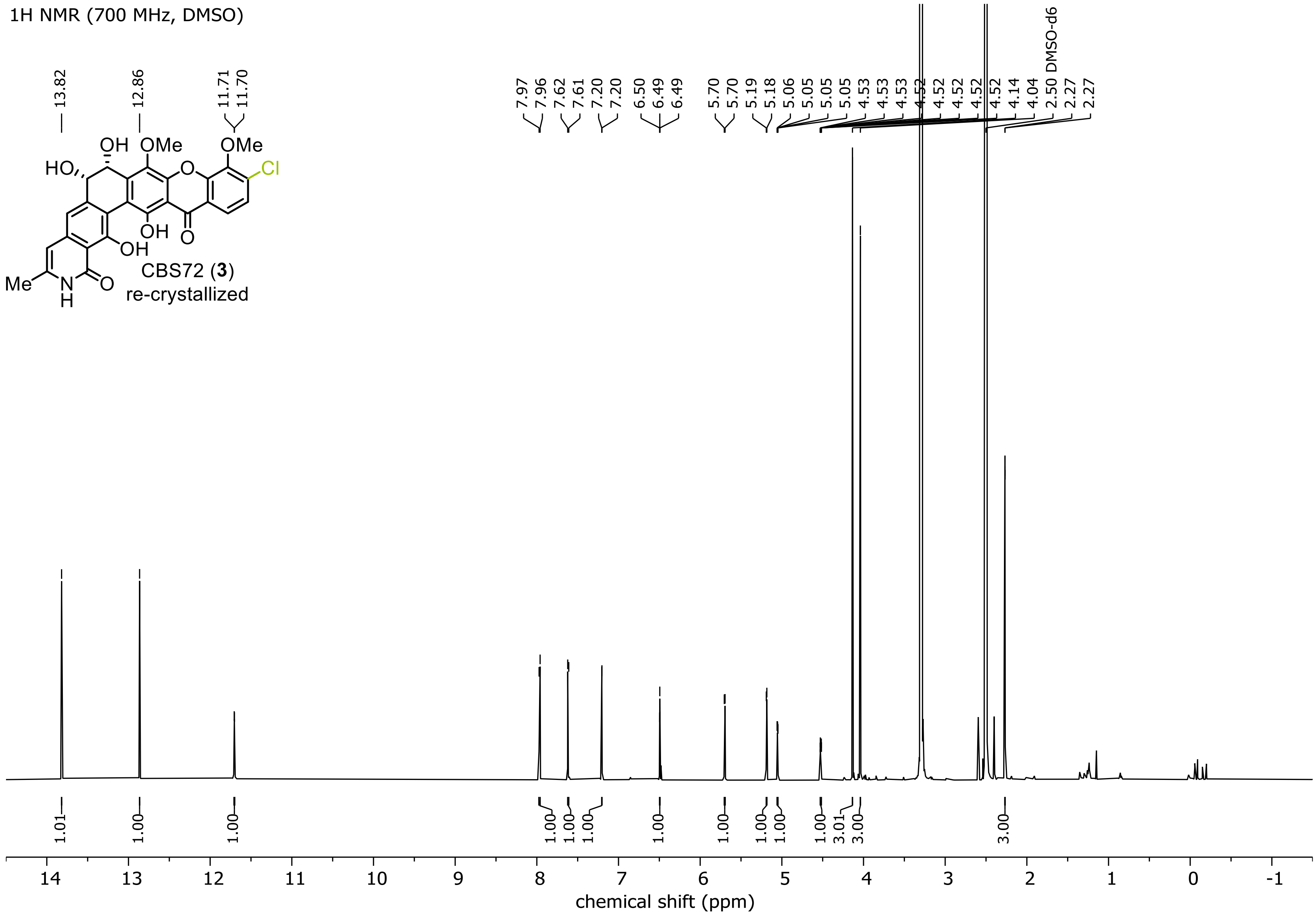
1H NMR (700 MHz, DMSO)
CBS72 (3)
re-crystallized
13.82
12.86
11.71
11.70
7.97
7.96
7.62
7.61
7.20
7.20
6.50
6.49
6.49
5.70
5.70
5.19
5.18
5.06
5.05
5.05
5.05
4.53
4.53
4.53
4.52
4.52
4.52
4.52
4.52
4.14
4.04
2.50 DMSO-d6
2.27
2.27
1.01
1.00
1.00
1.00
1.00
1.00
1.00
1.00
1.00
1.00
1.00
3.01
3.00
3.00
chemical shift (ppm)

7.6 Additional Spectra

1H NMR (400 MHz, CD2Cl2)

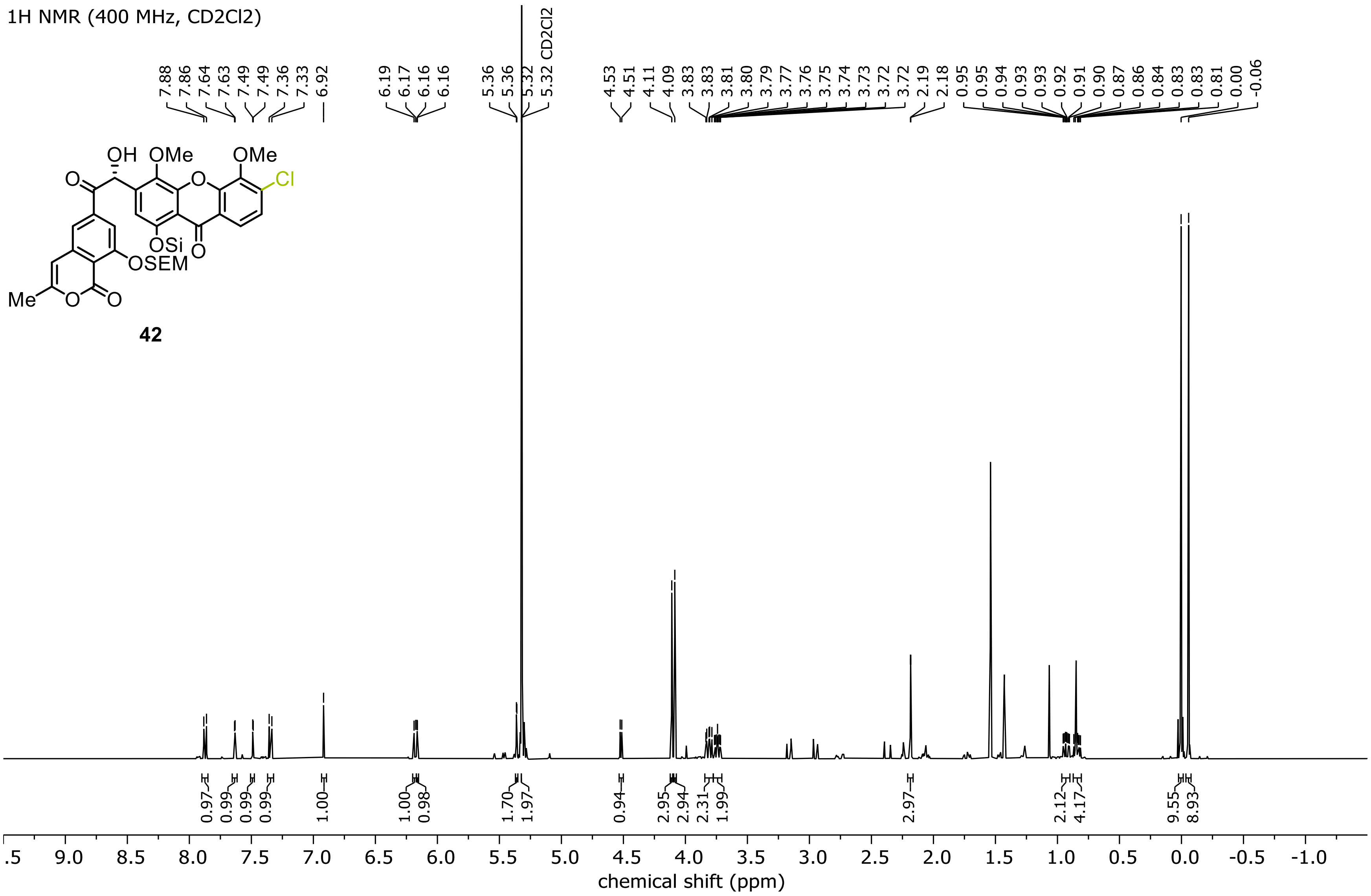

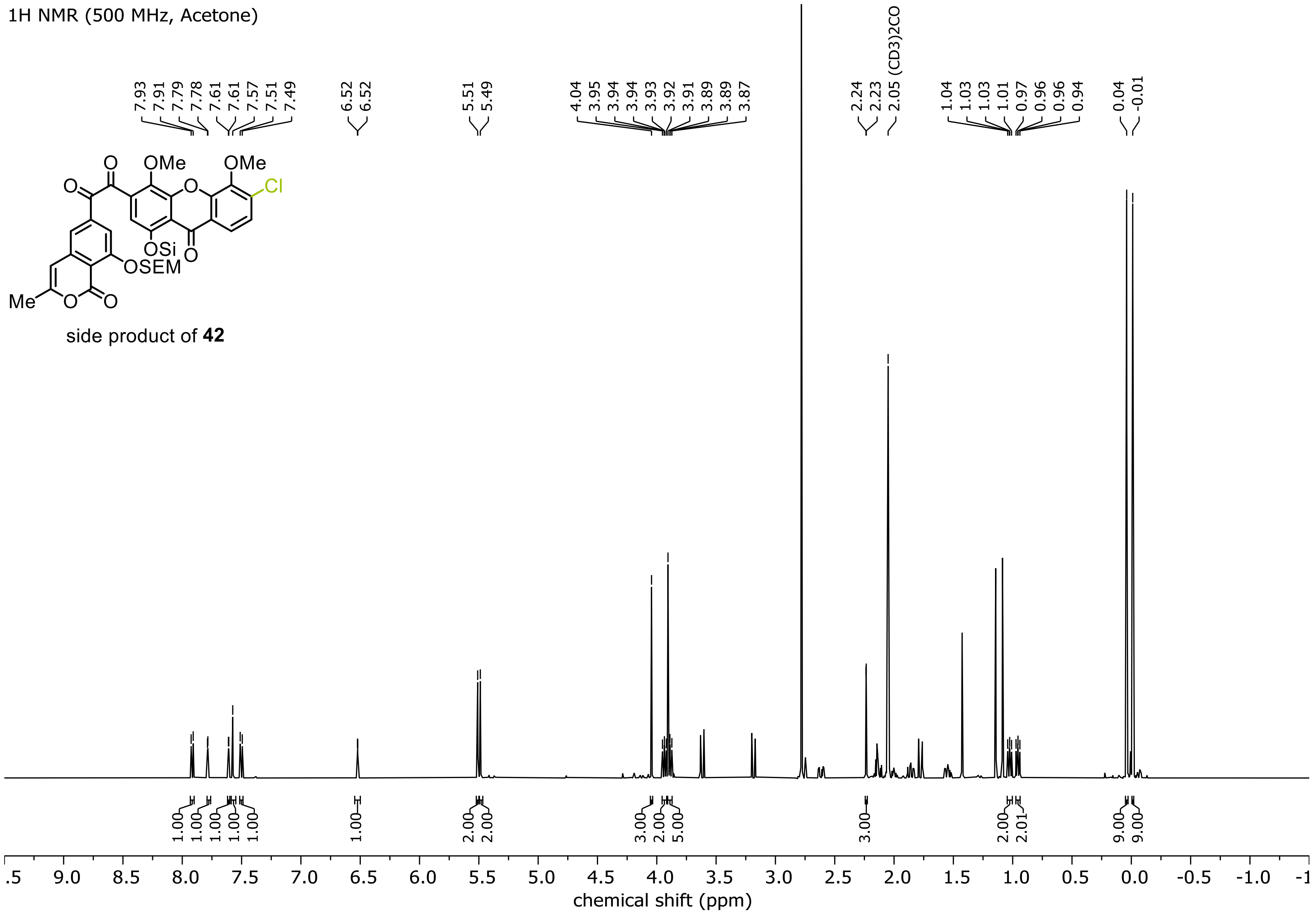

1H NMR (500 MHz, Acetone)
7.93
7.91
7.79
7.78
7.61
7.61
7.57
7.51
7.49
6.52
6.52
5.51
5.49
4.04
3.95
3.94
3.94
3.93
3.92
3.91
3.89
3.89
3.87
2.24
2.23
2.05 (CD3)2CO
1.04
1.03
1.03
1.01
0.97
0.96
0.96
0.94
0.04
-0.01
OMe
OMe
Cl
OSi
OSEM
Me
side product of **42**
1.00
1.00
1.00
1.00
1.00
1.00
2.00
2.00
3.00
2.00
5.00
3.00
2.00
2.01
9.00
9.00
chemical shift (ppm)

13C NMR (125 MHz, Acetone)

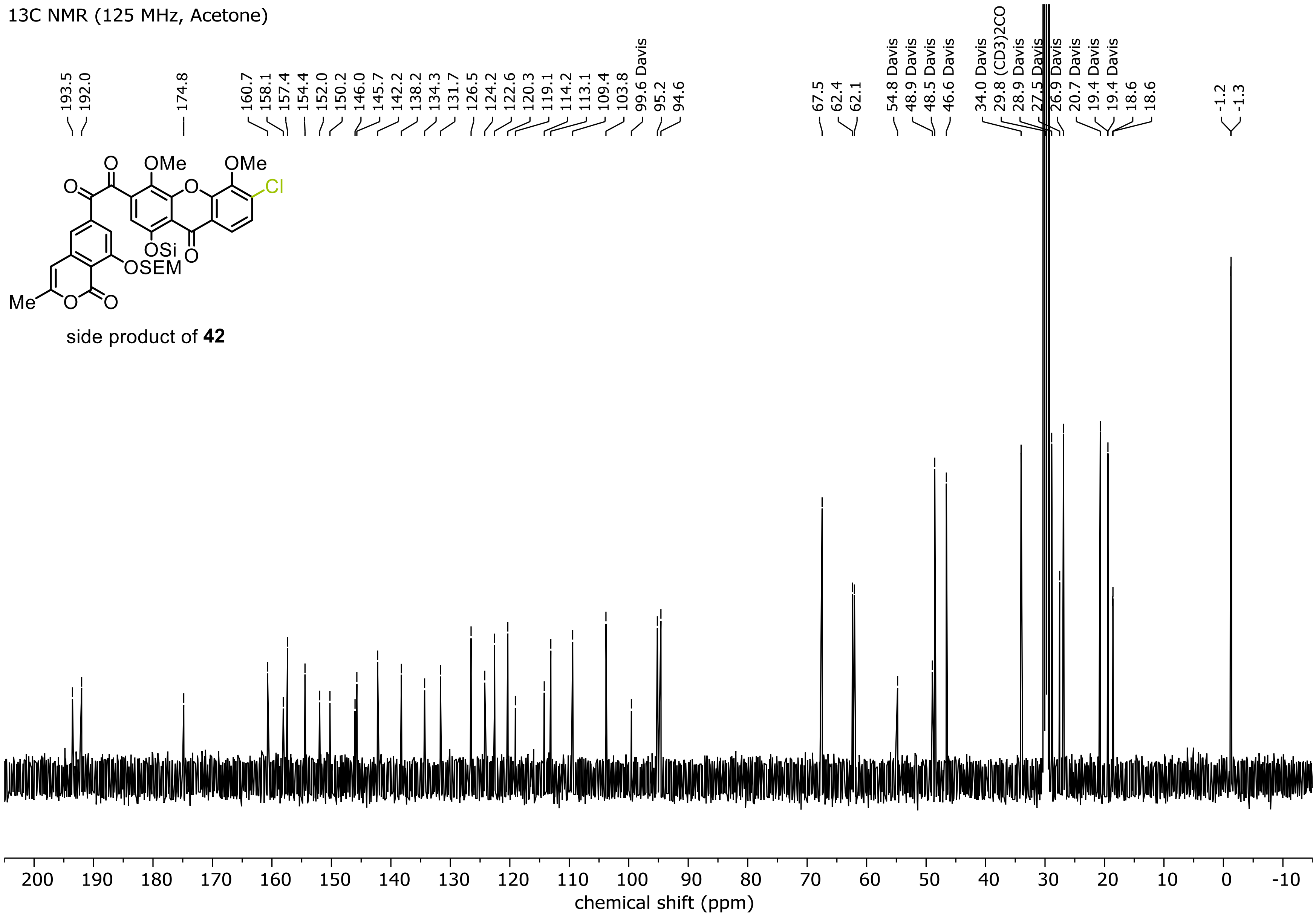

side product of **42**

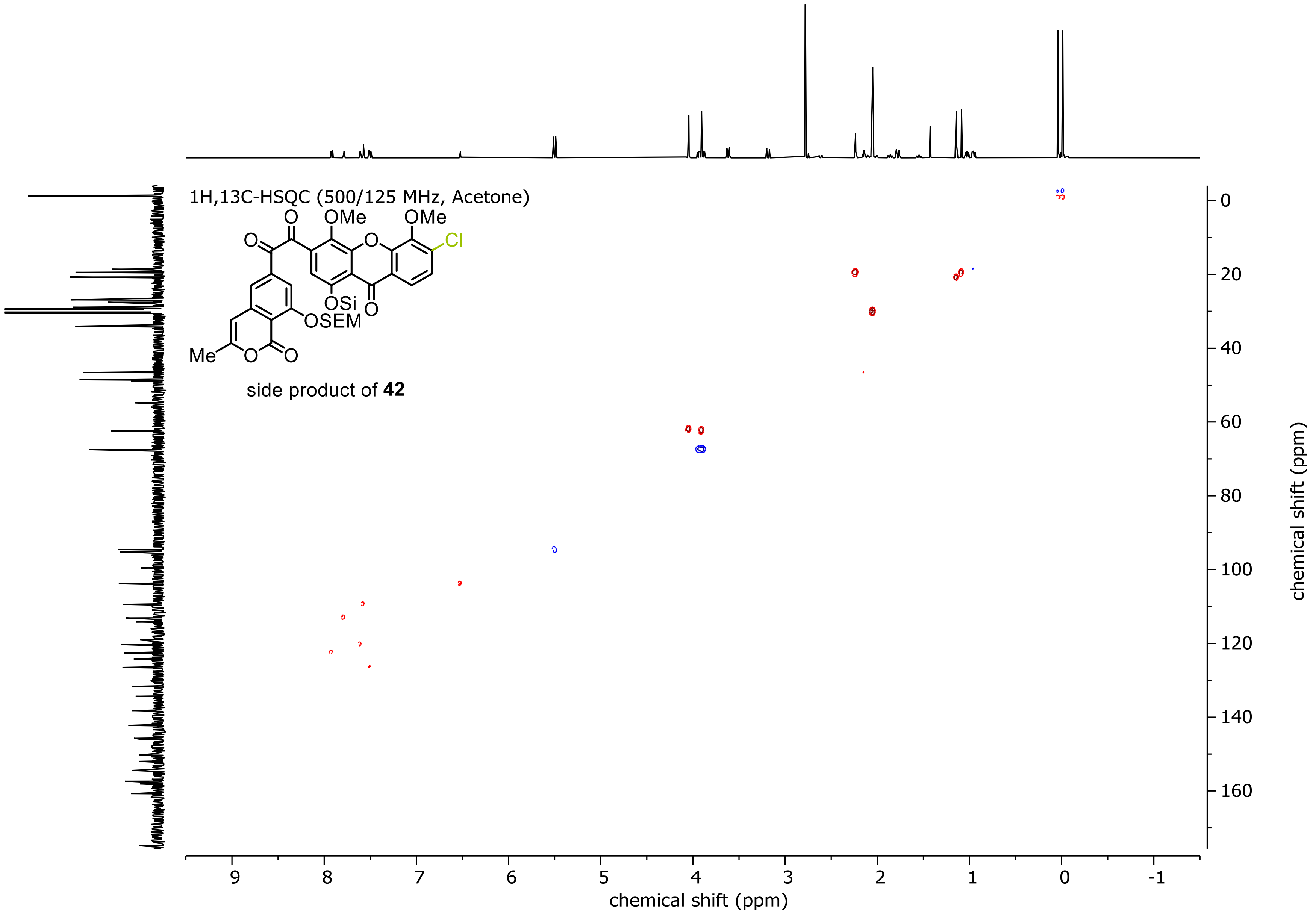
1H,13C-HSQC (500/125 MHz, Acetone)
O
OMe
OMe
O
O
Cl
OSi
O
OSEM
Me
O
O
side product of **42**
chemical shift (ppm)
chemical shift (ppm)

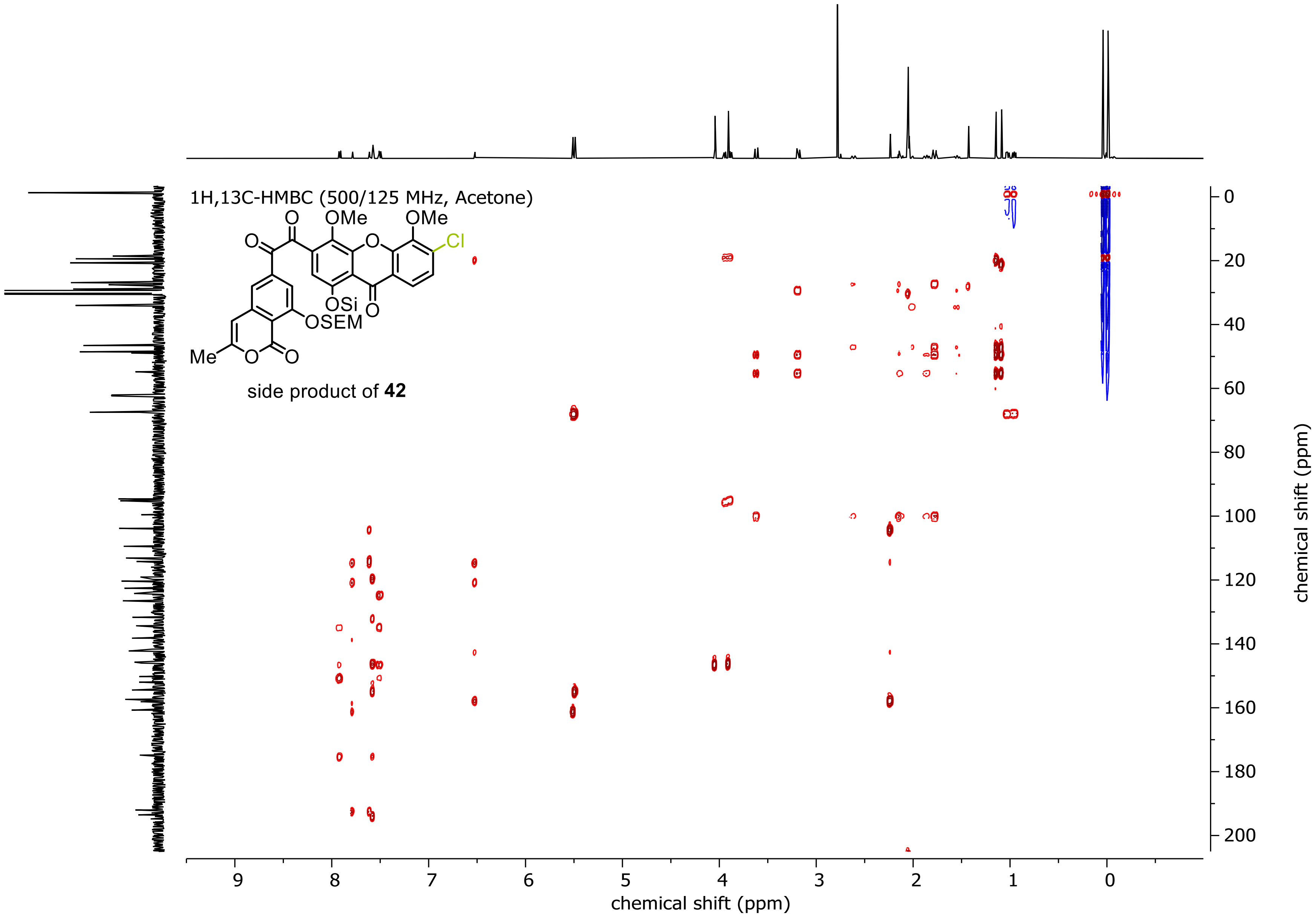
1H,13C-HMBC (500/125 MHz, Acetone)
O
OMe
OMe
O
Cl
O
OSi
OSEM
O
Me
O
O
side product of **42**
chemical shift (ppm)
chemical shift (ppm)

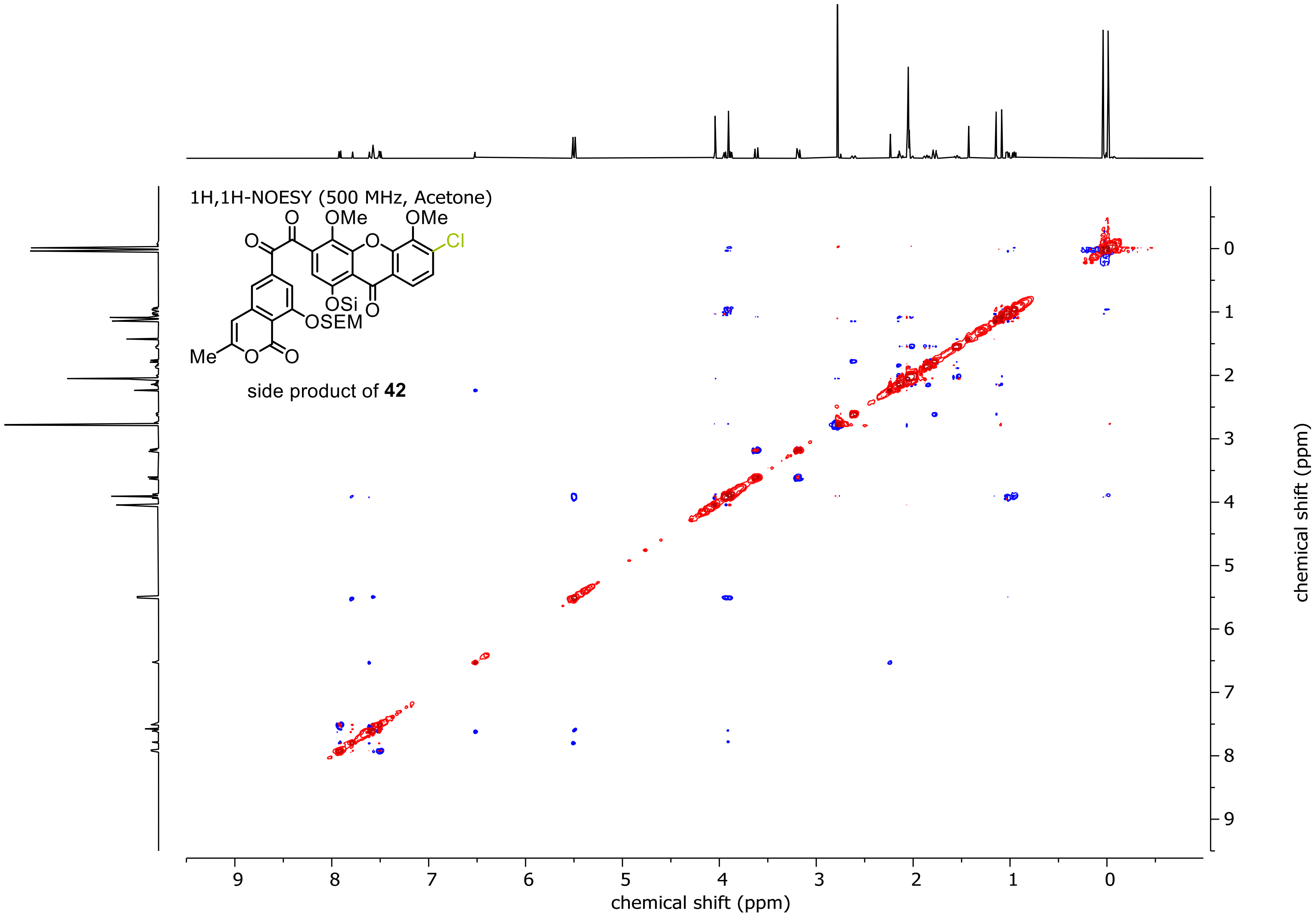

1H,1H-NOESY (500 MHz, Acetone)
OMe
OMe
Cl
OSi
OSEM
Me
side product of **42**
chemical shift (ppm)
chemical shift (ppm)

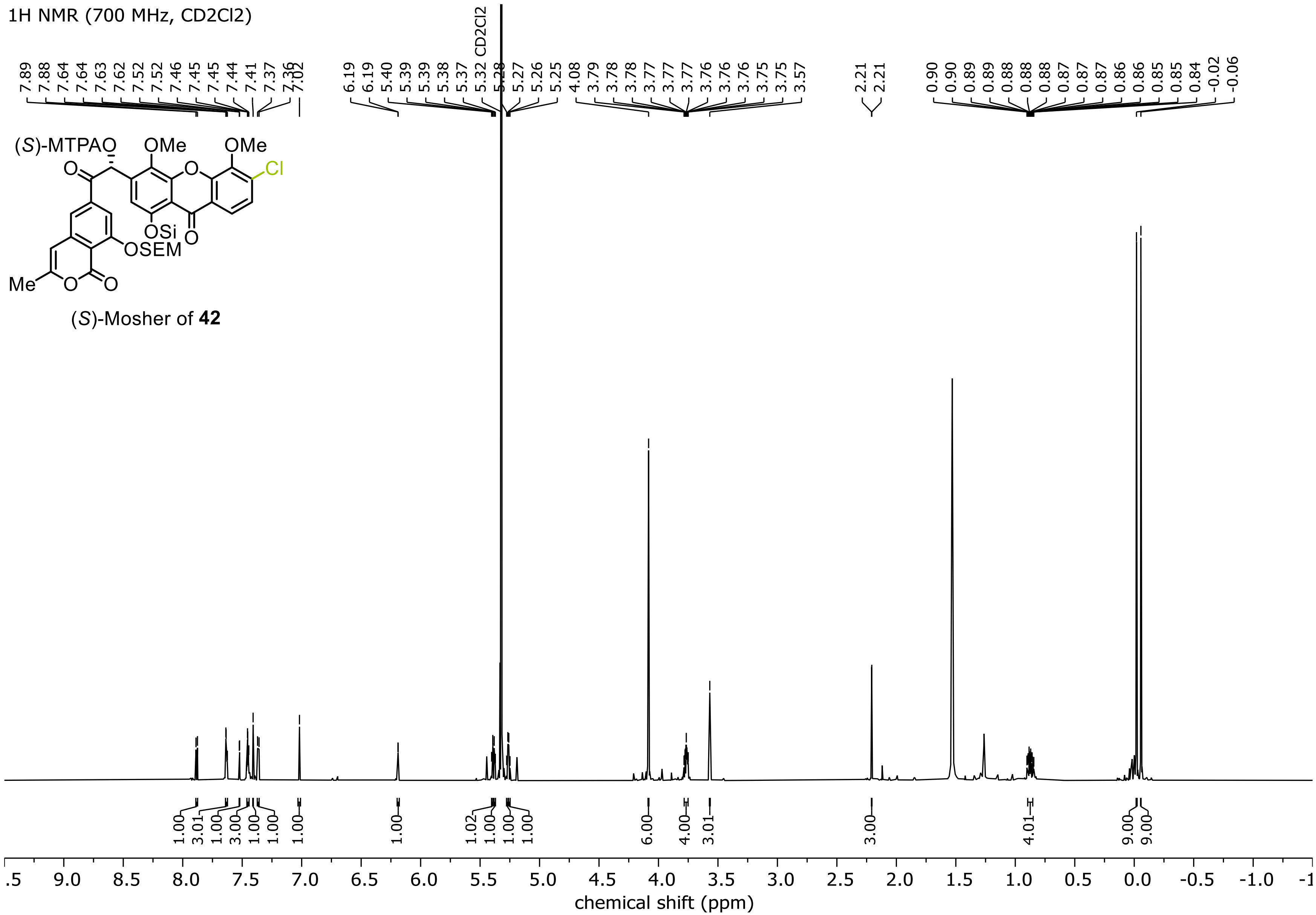
1H NMR (700 MHz, CD2Cl2)
7.89
7.88
7.64
7.64
7.63
7.62
7.52
7.52
7.46
7.45
7.45
7.44
7.41
7.37
7.36
7.02
6.19
6.19
5.40
5.39
5.39
5.38
5.37
5.32 CD2Cl2
5.28
5.27
5.26
5.25
4.08
3.79
3.78
3.78
3.77
3.77
3.77
3.76
3.76
3.76
3.75
3.75
3.57
2.21
2.21
0.90
0.90
0.89
0.89
0.88
0.88
0.88
0.87
0.87
0.87
0.86
0.86
0.85
0.85
0.84
-0.02
-0.06
(S)-MTPAO
OMe
OMe
O
O
Cl
OSi
O
OSEM
Me
O
O
(S)-Mosher of 42
1.00
3.01
1.00
3.00
1.00
1.00
1.00
1.00
1.02
1.00
1.00
1.00
6.00
4.00
3.01
3.00
4.01
9.00
9.00
.5
9.0
8.5
8.0
7.5
7.0
6.5
6.0
5.5
5.0
4.5
4.0
3.5
3.0
2.5
2.0
1.5
1.0
0.5
0.0
-0.5
-1.0
-1
chemical shift (ppm)

13C NMR (176 MHz, CD2Cl2)

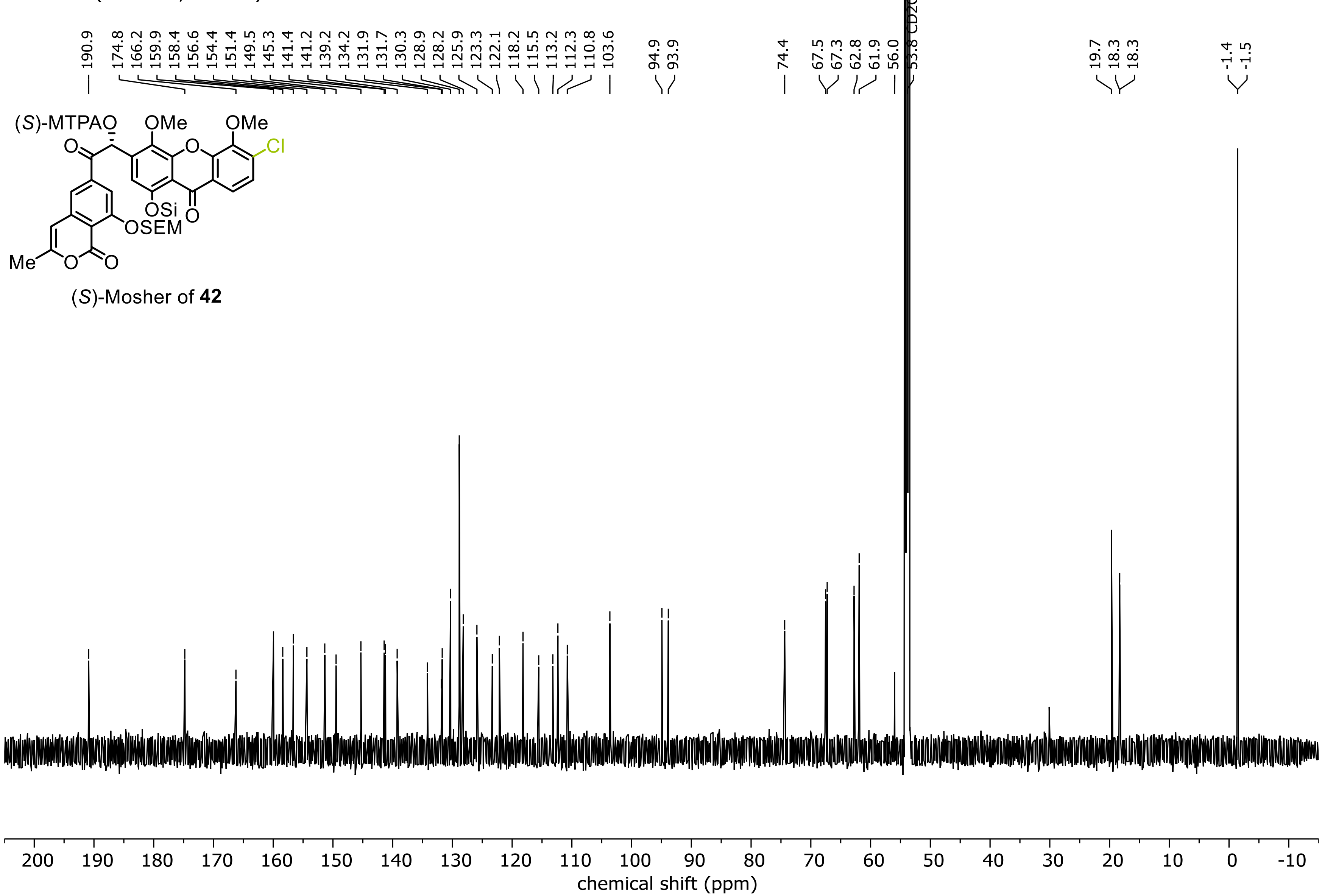

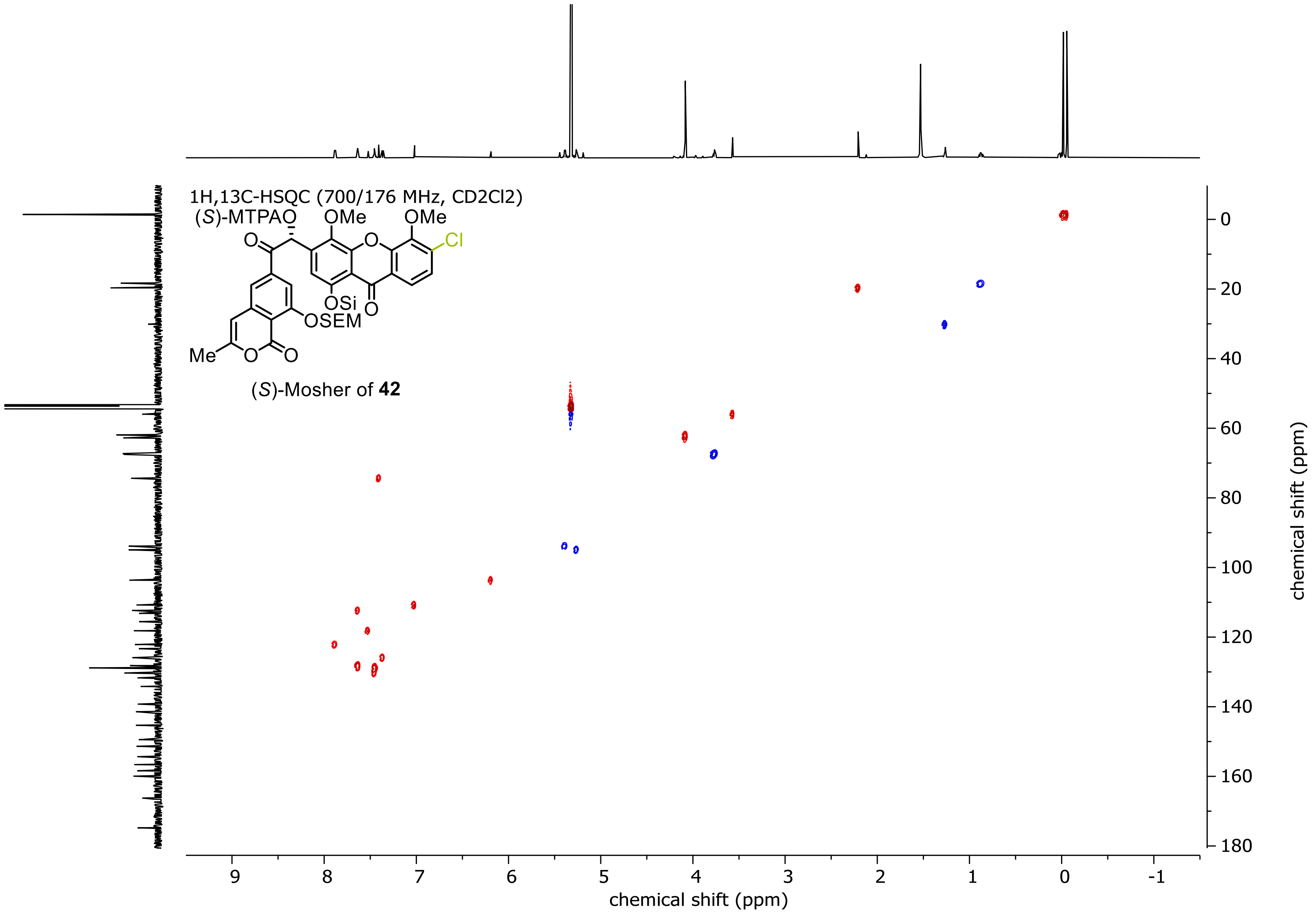
1H,13C-HSQC (700/176 MHz, CD2Cl2)
(S)-MTPAO
OMe
OMe
Cl
O
OSi
OSEM
Me
(S)-Mosher of 42
chemical shift (ppm)
chemical shift (ppm)

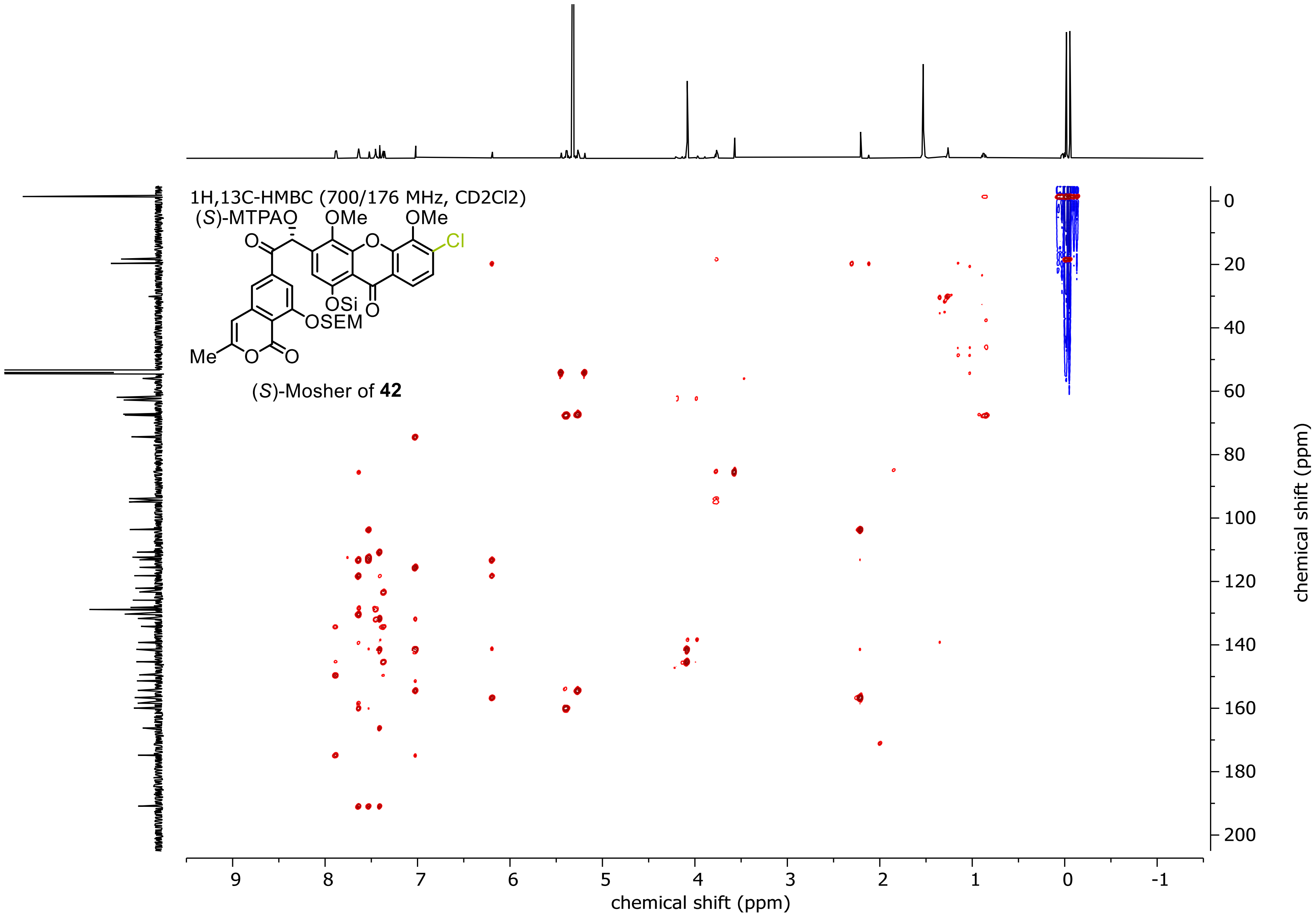
1H,13C-HMBC (700/176 MHz, CD2Cl2)
(S)-MTPAO
OMe
OMe
O
Cl
O
OSi
O
OSEM
Me
O
O
(S)-Mosher of 42
chemical shift (ppm)
chemical shift (ppm)

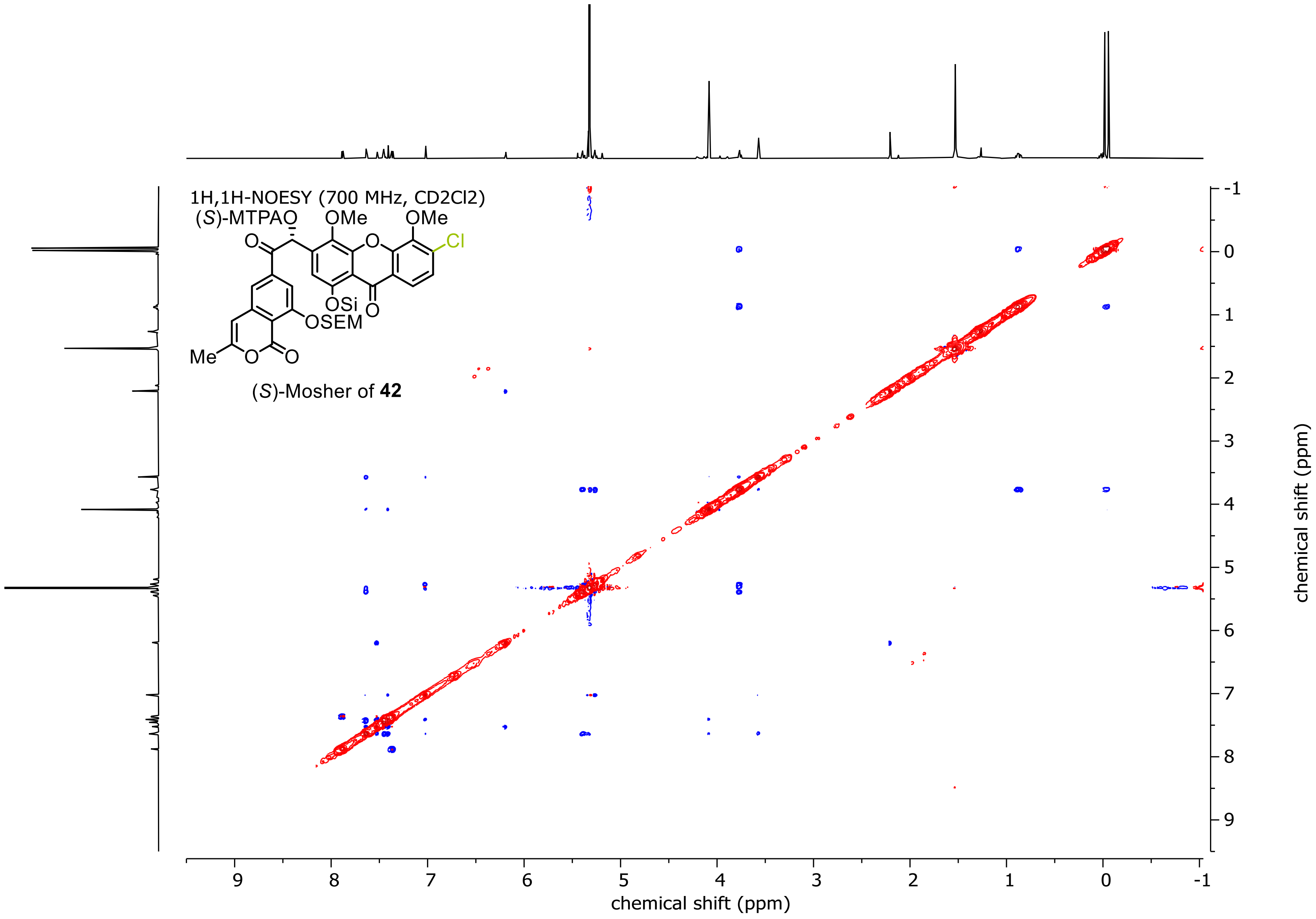

1H,1H-NOESY (700 MHz, CD2Cl2)
(S)-MTPAO
OMe
OMe
Cl
OSi
OSEM
Me
(S)-Mosher of 42
chemical shift (ppm)
chemical shift (ppm)

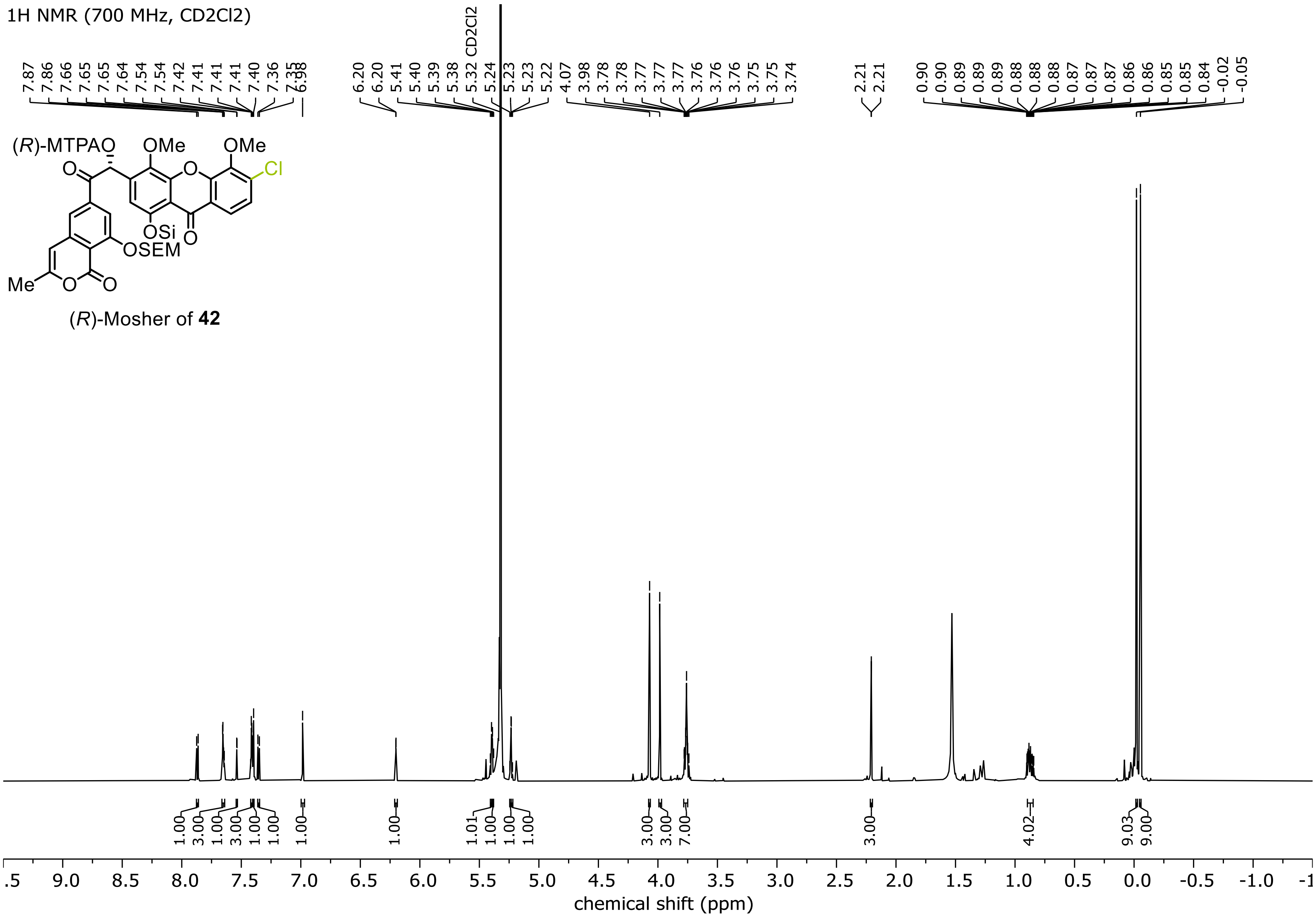

(R)-Mosher of **42**

13C NMR (176 MHz, CD2Cl2)

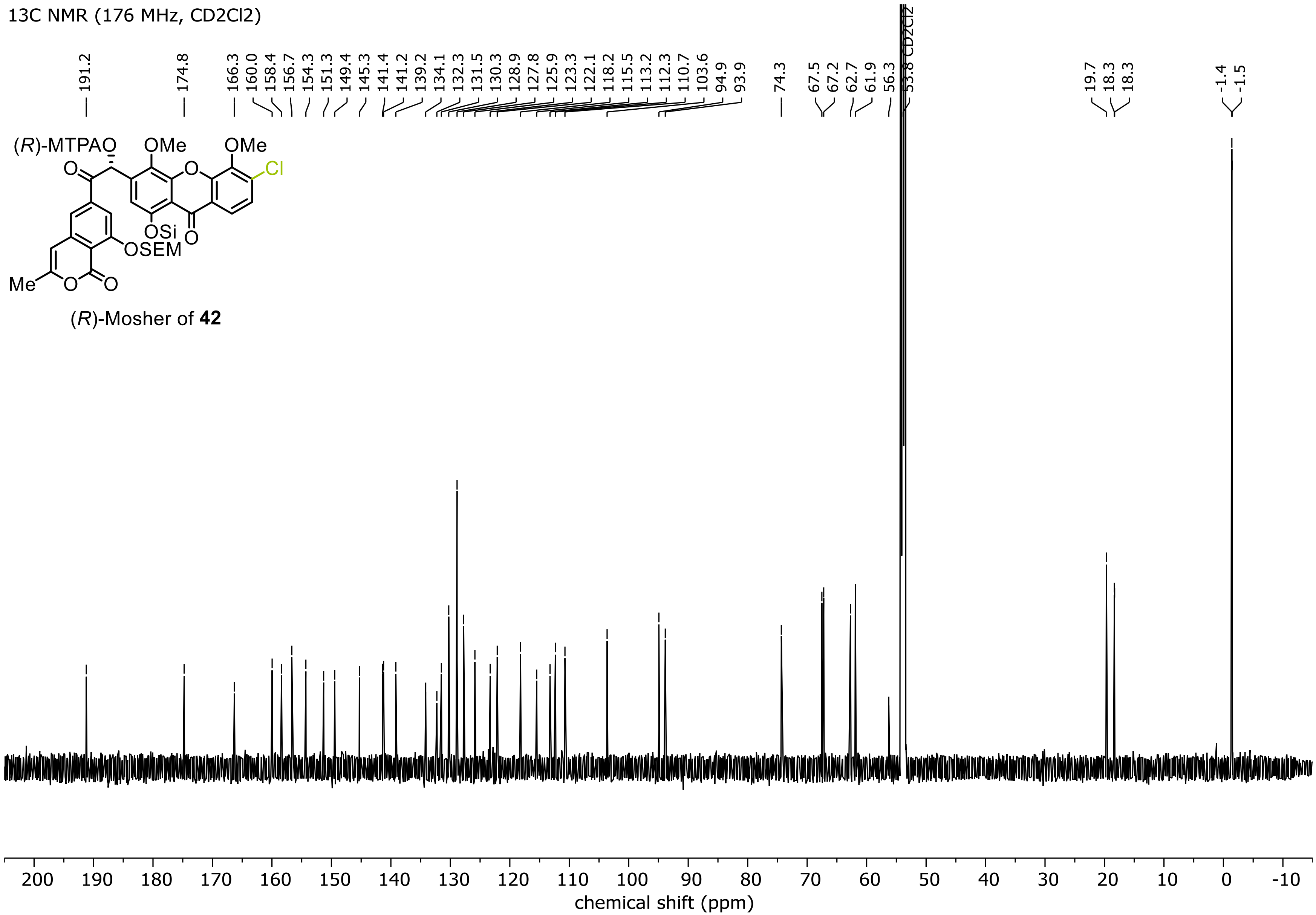

(*R*)-Mosher of **42**

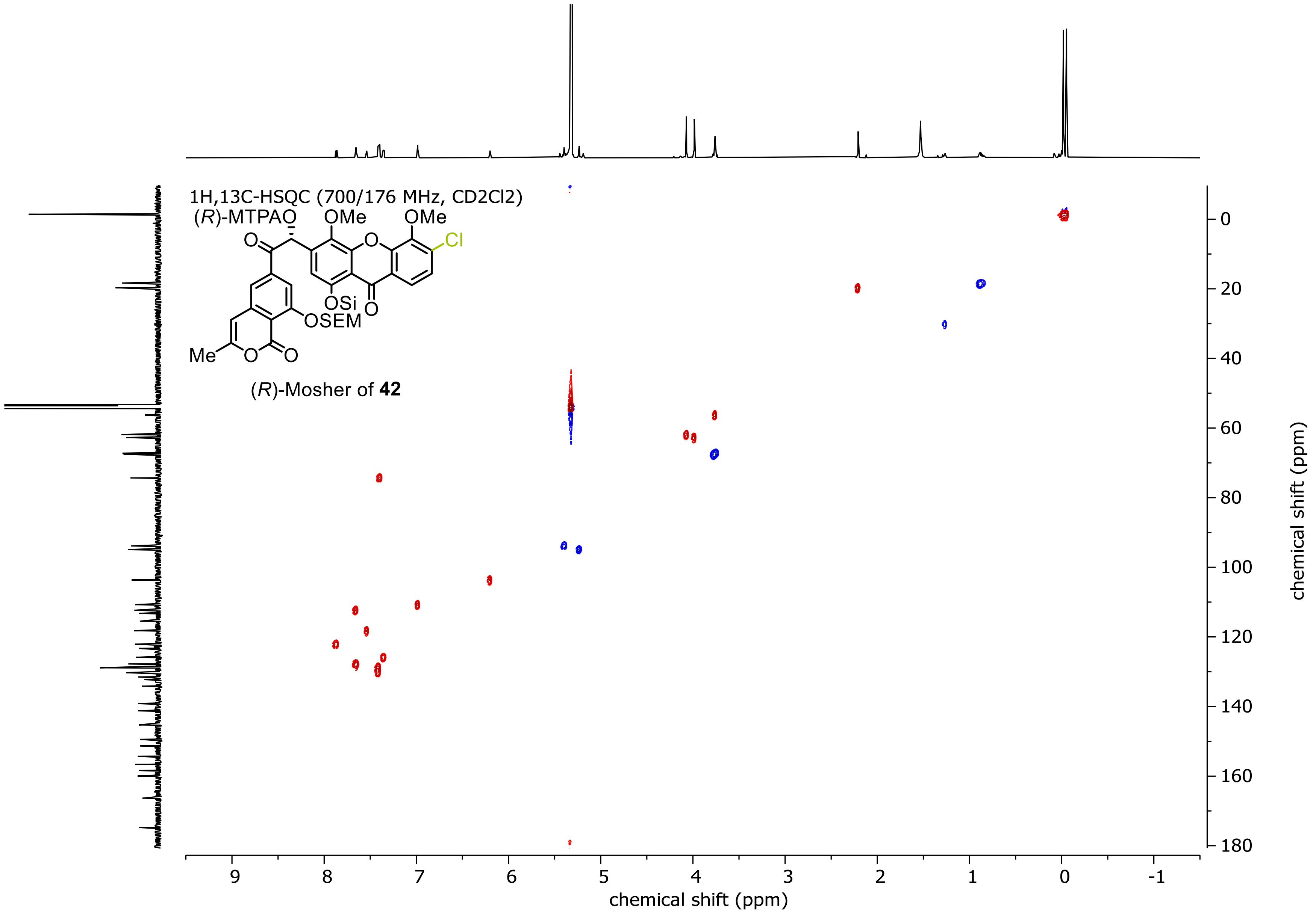
1H,13C-HSQC (700/176 MHz, CD2Cl2)
(R)-MTPAO
OMe
OMe
Cl
OSi
OSEM
Me
(R)-Mosher of 42
chemical shift (ppm)
chemical shift (ppm)

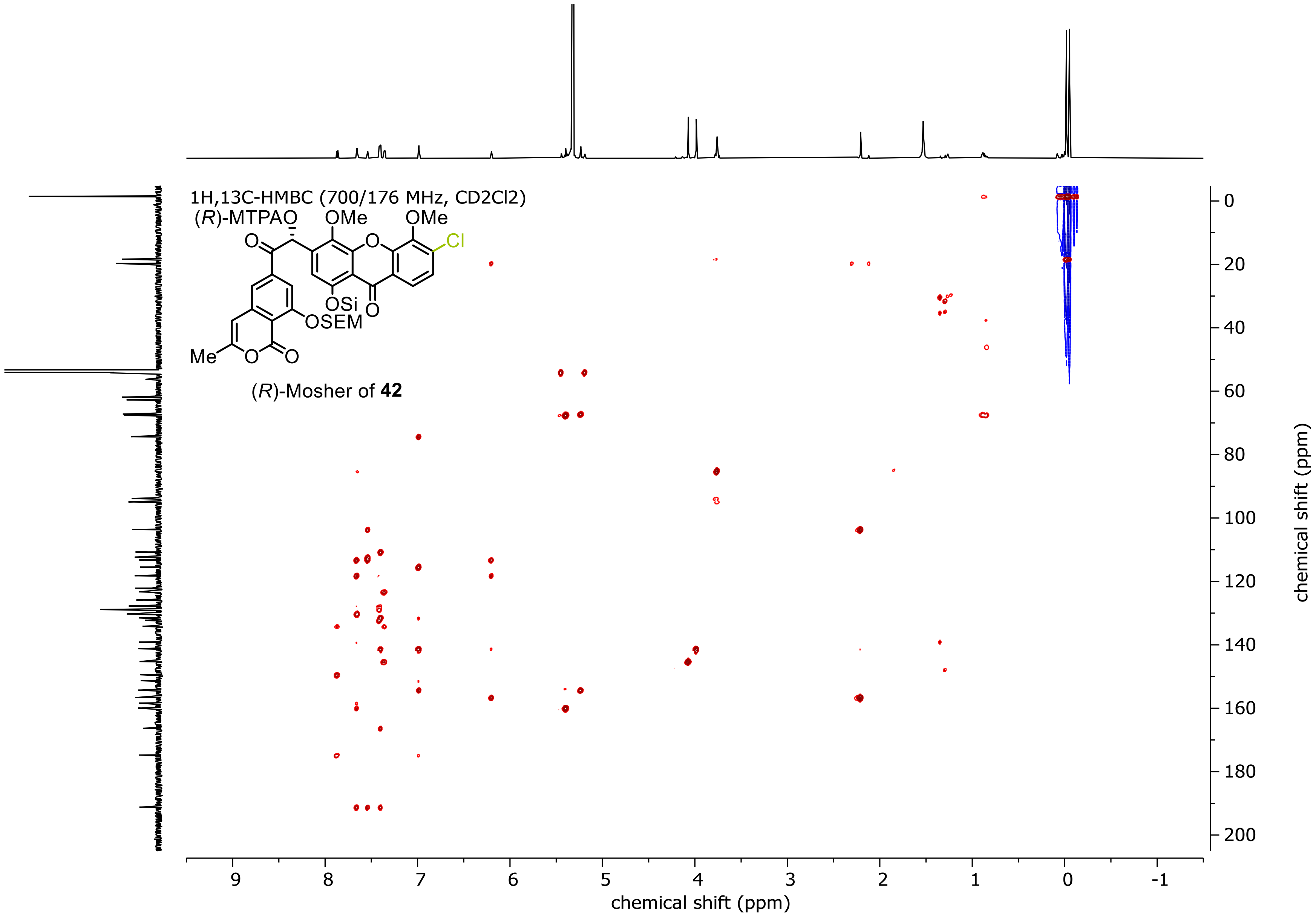
1H,13C-HMBC (700/176 MHz, CD2Cl2)
(R)-MTPAO
OMe
OMe
Cl
O
OSi
OSEM
Me
(R)-Mosher of 42
chemical shift (ppm)
chemical shift (ppm)

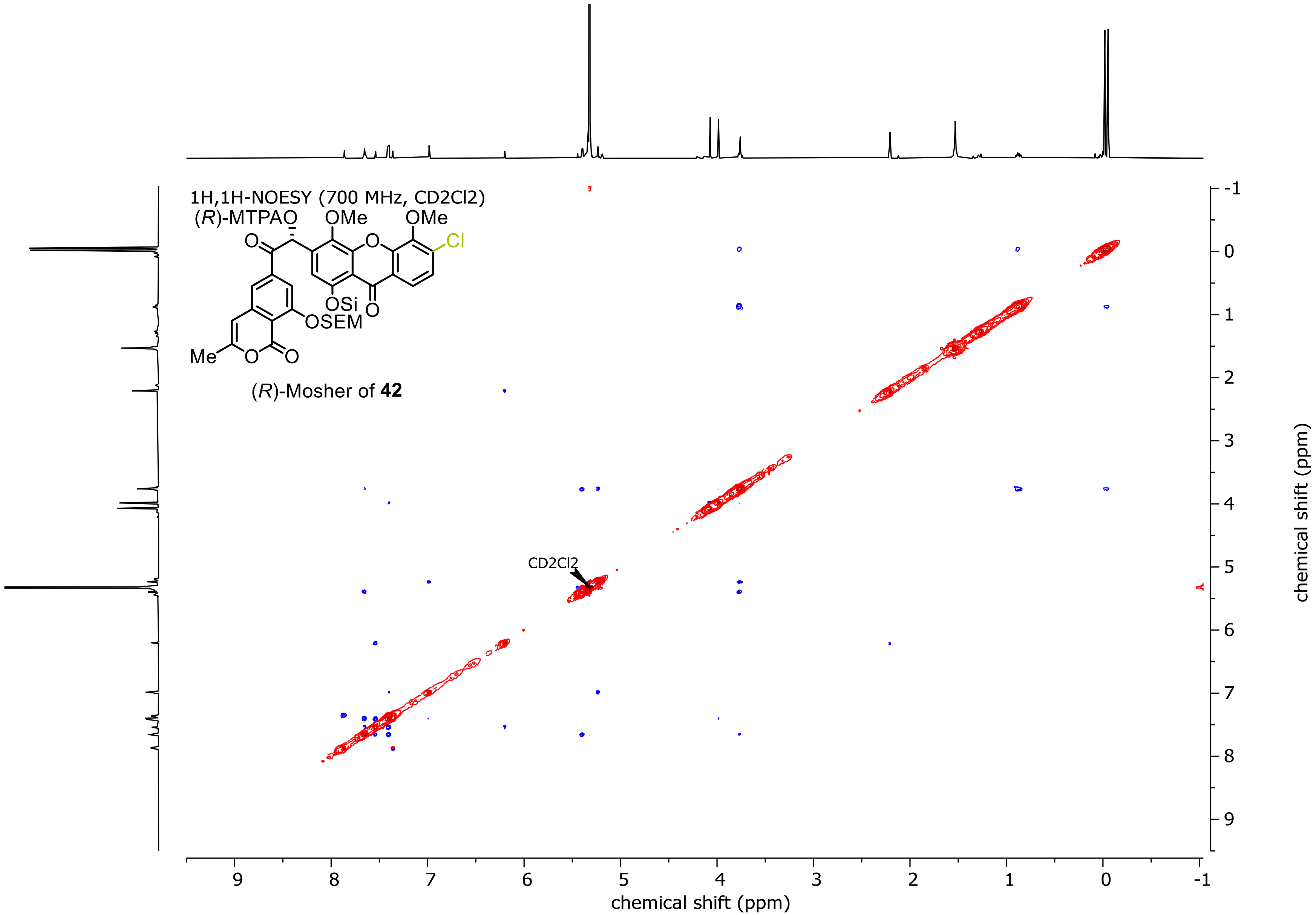
1H,1H-NOESY (700 MHz, CD2Cl2)
(R)-MTPAO
OMe
OMe
Cl
O
OSi
OSEM
Me
(R)-Mosher of 42
CD2Cl2
chemical shift (ppm)
chemical shift (ppm)

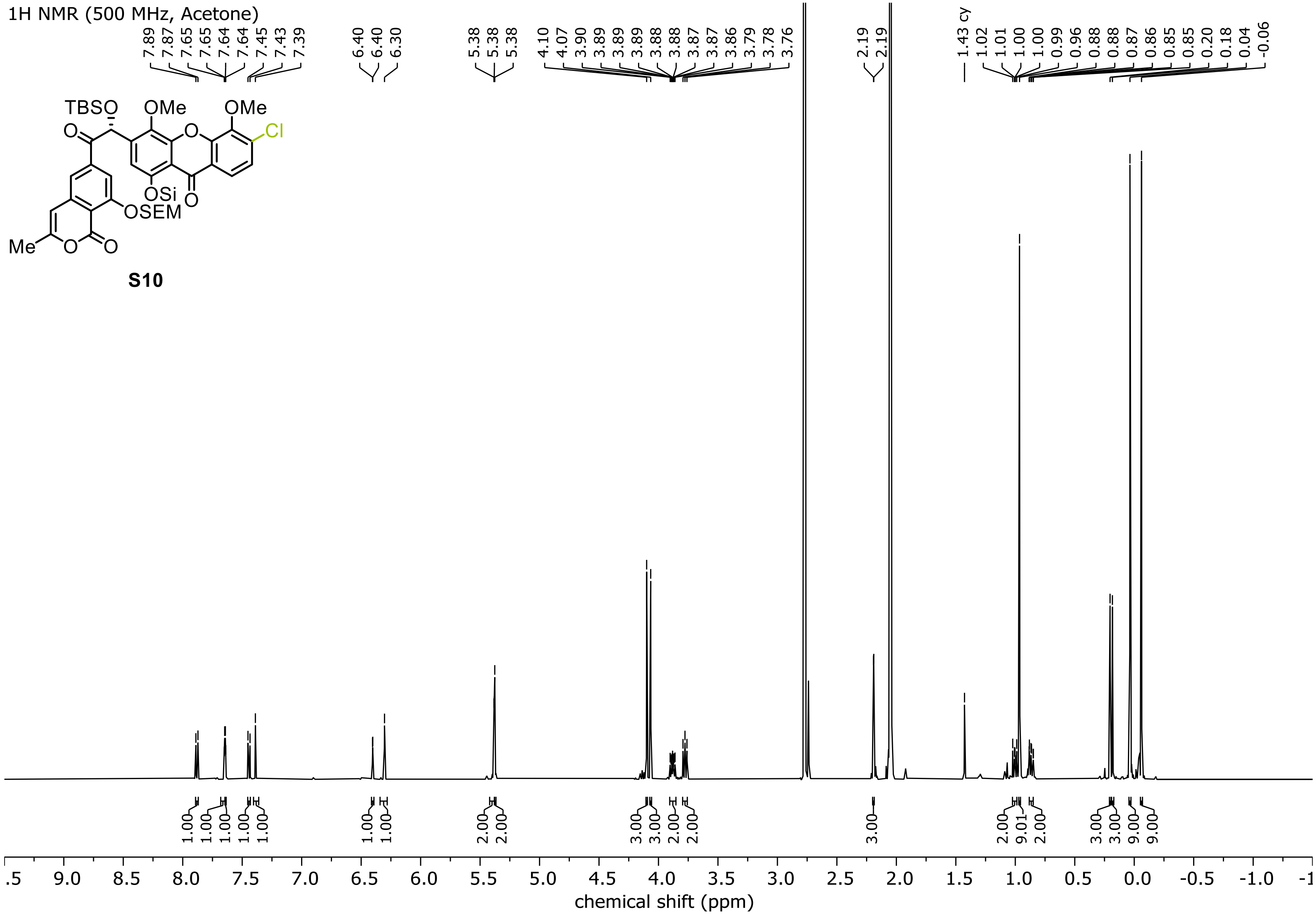

1H NMR (500 MHz, Acetone)
7.89 7.87 7.65 7.65 7.64 7.64 7.45 7.43 7.39
6.40 6.40 6.30
5.38 5.38 5.38
4.10 4.07 3.90 3.89 3.89 3.89 3.88 3.88 3.87 3.87 3.86 3.79 3.78 3.76
2.19 2.19
1.43 cy
1.02 1.01 1.00 1.00 0.99 0.96 0.88 0.88 0.87 0.86 0.85 0.85 0.20 0.18 0.04 -0.06
TBSO
OMe
OMe
Cl
O
OSi
OSEM
Me
S10
1.00 1.00 1.00 1.00 1.00
1.00 1.00
2.00 2.00
3.00 3.00 2.00 2.00
3.00
2.00 9.01 2.00
3.00 3.00 9.00 9.00
.5 9.0 8.5 8.0 7.5 7.0 6.5 6.0 5.5 5.0 4.5 4.0 3.5 3.0 2.5 2.0 1.5 1.0 0.5 0.0 -0.5 -1.0 -1
chemical shift (ppm)

13C NMR (125 MHz, Acetone)

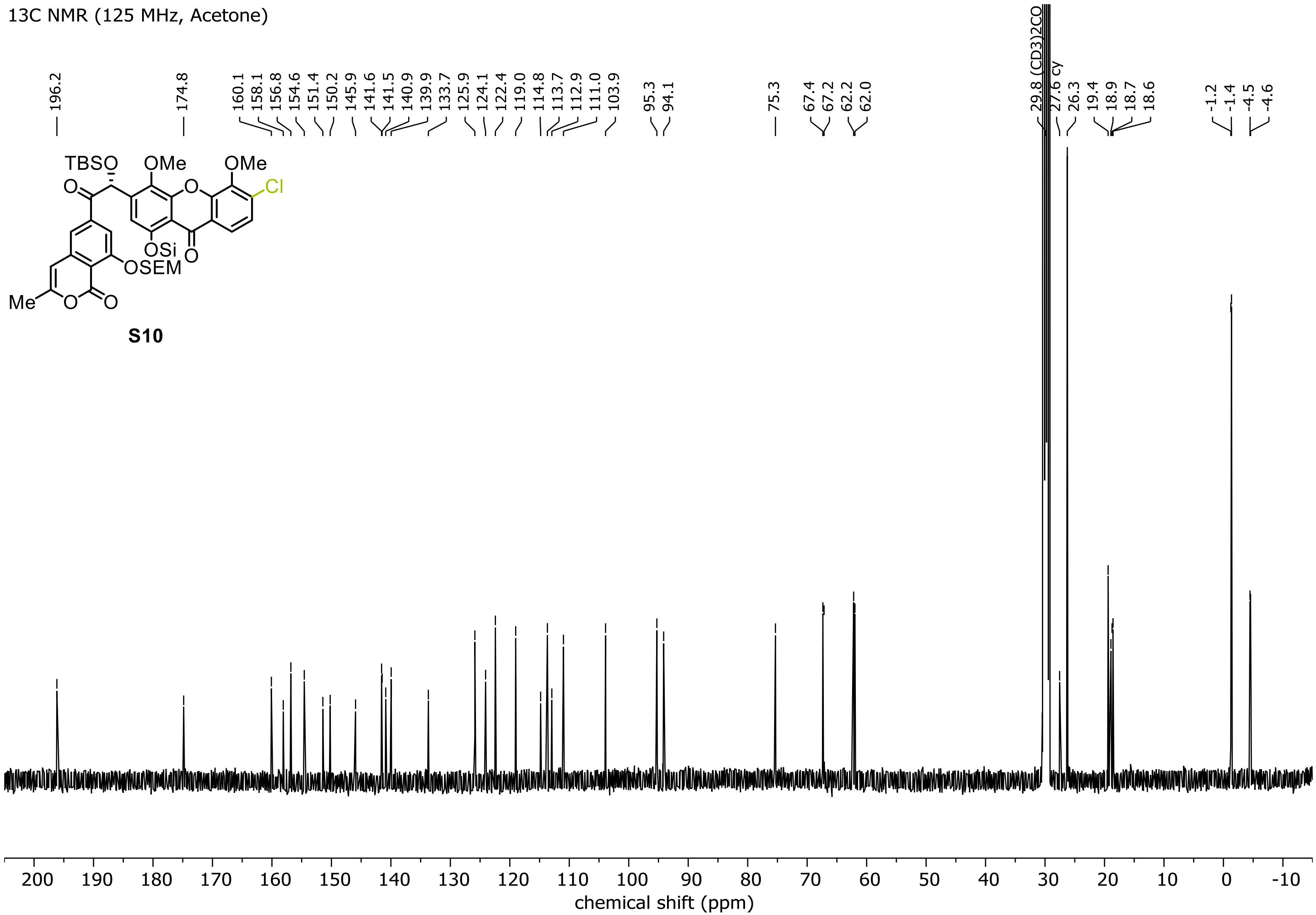

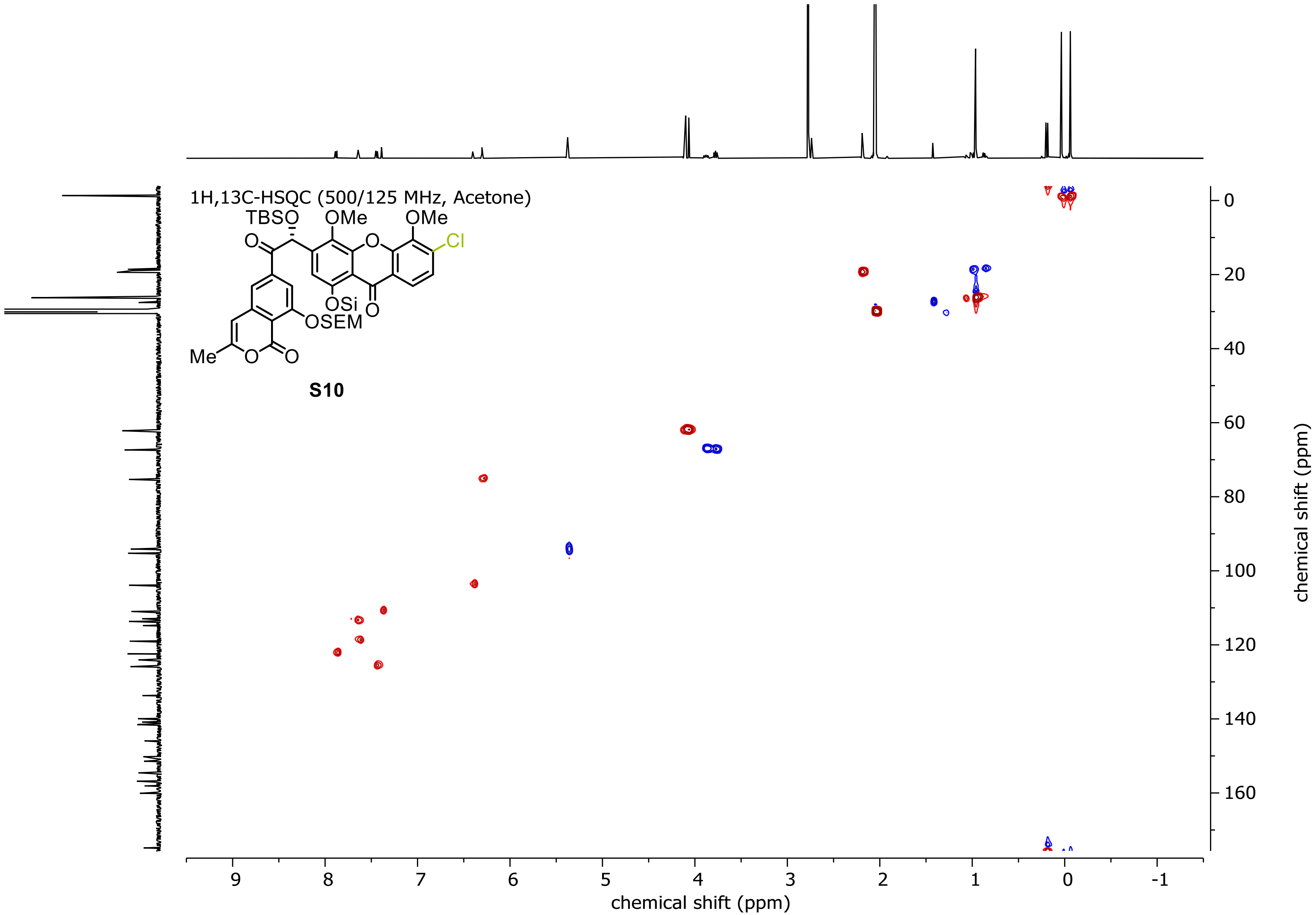
1H,13C-HSQC (500/125 MHz, Acetone)
TBSO
OMe
OMe
O
O
Cl
OSi
O
OSEM
Me
O
O
S10
0
20
40
60
80
100
120
140
160
chemical shift (ppm)
9
8
7
6
5
4
3
2
1
0
-1
chemical shift (ppm)

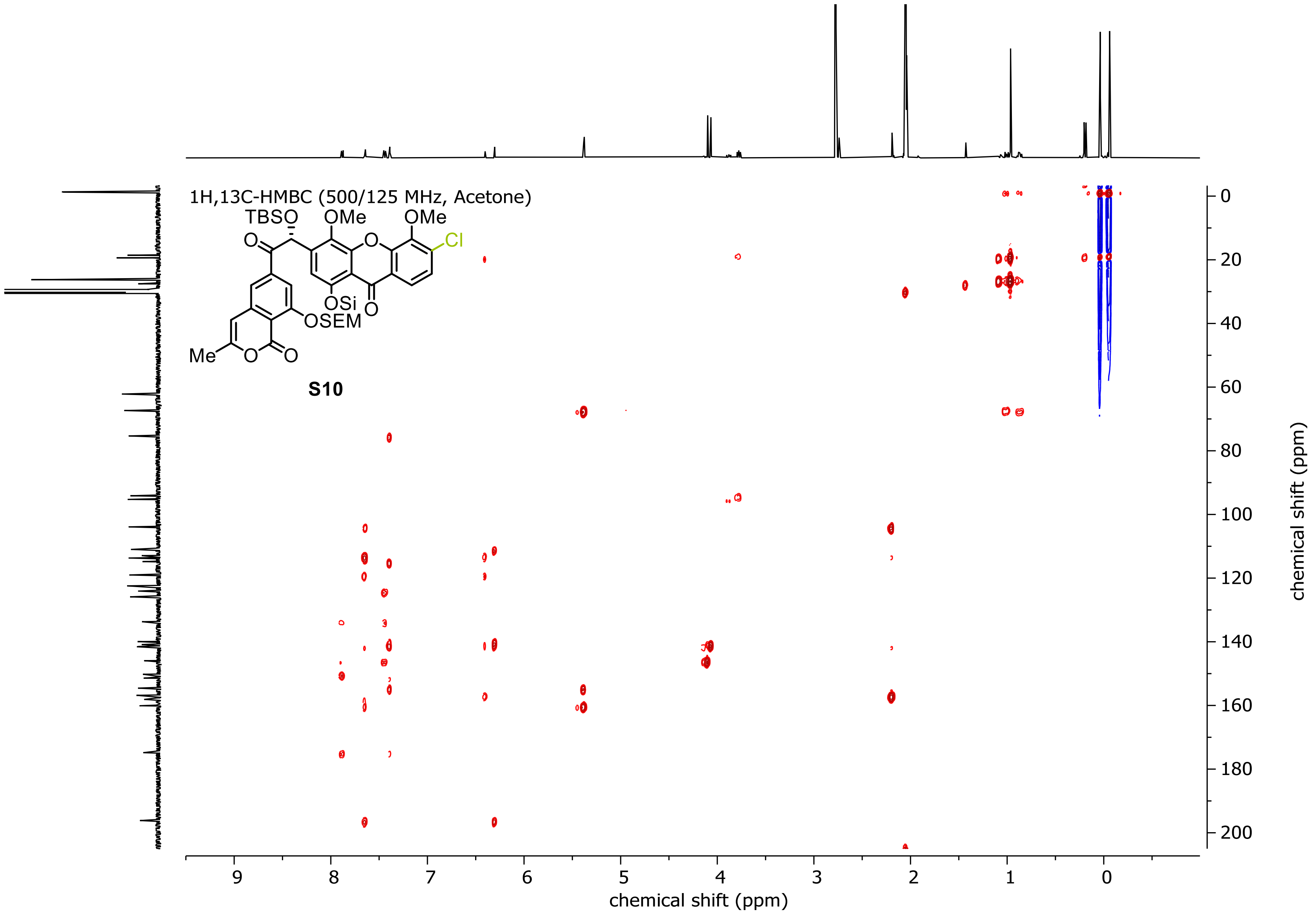
1H,13C-HMBC (500/125 MHz, Acetone)
TBSO
OMe
OMe
Cl
O
OSi
OSEM
Me
S10
chemical shift (ppm)
chemical shift (ppm)

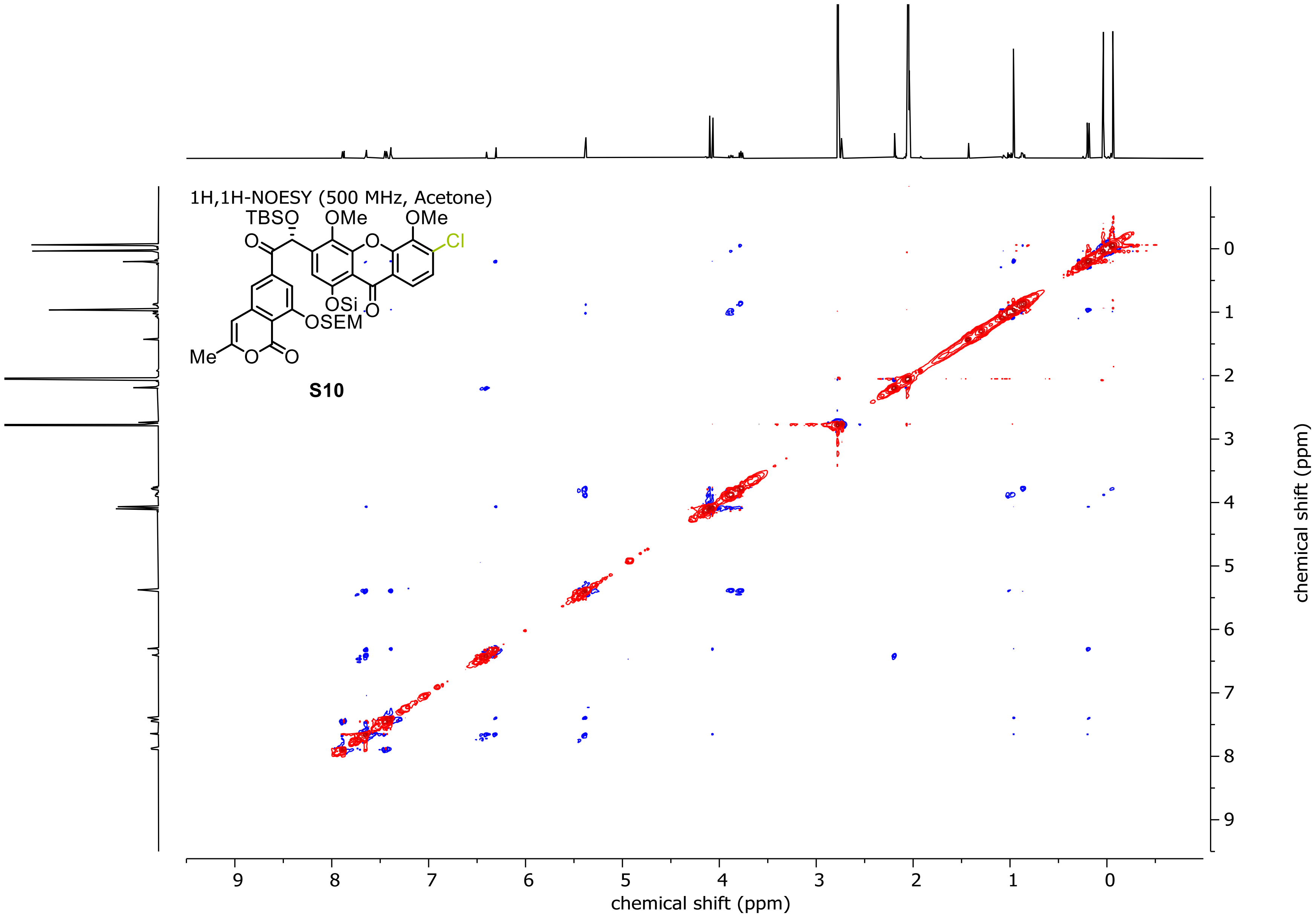
1H,1H-NOESY (500 MHz, Acetone)
TBSO
OMe
OMe
Cl
O
OSi
OSEM
Me
S10
chemical shift (ppm)
chemical shift (ppm)

1H NMR (500 MHz, Acetone)

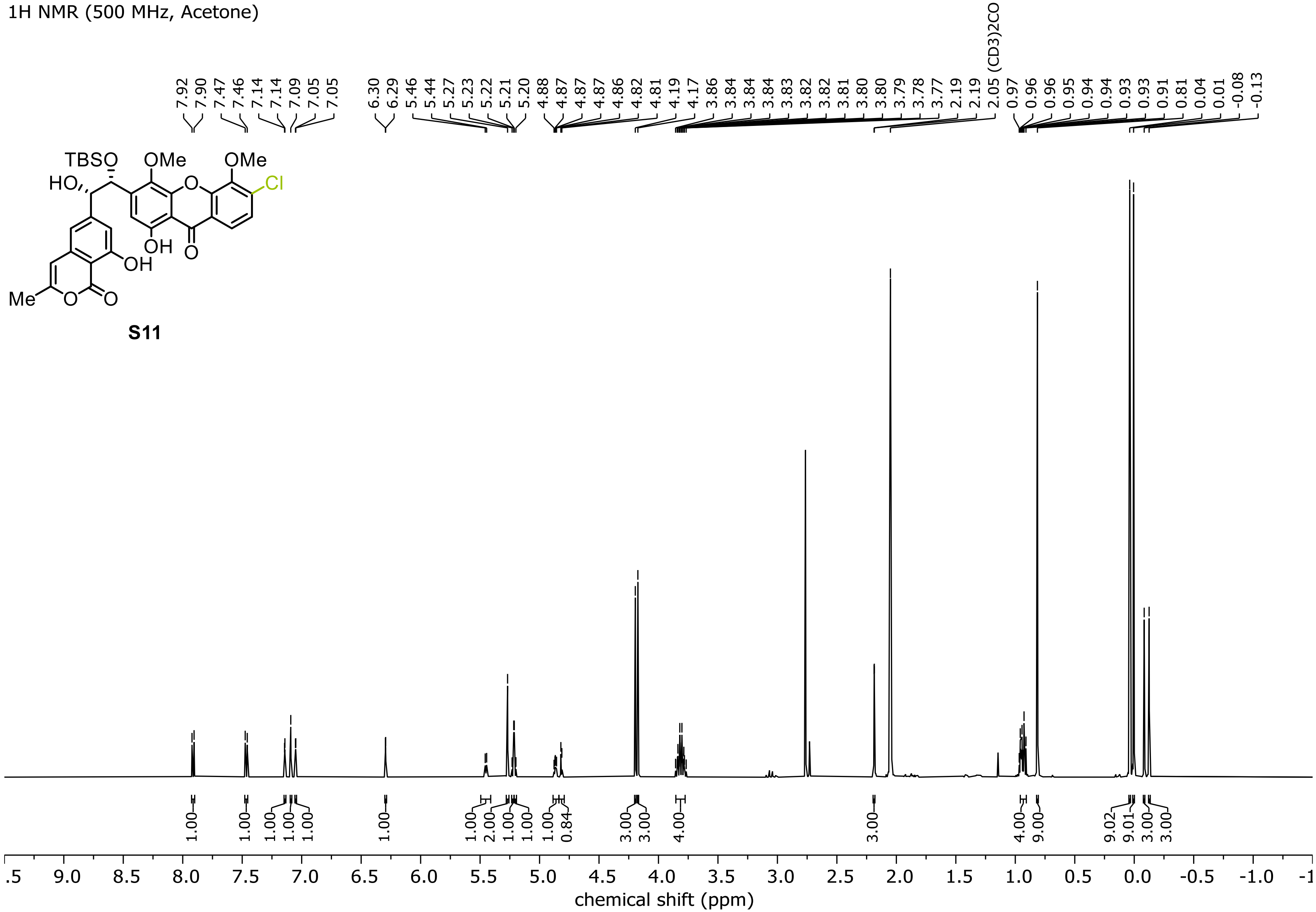

13C NMR (125 MHz, Acetone)

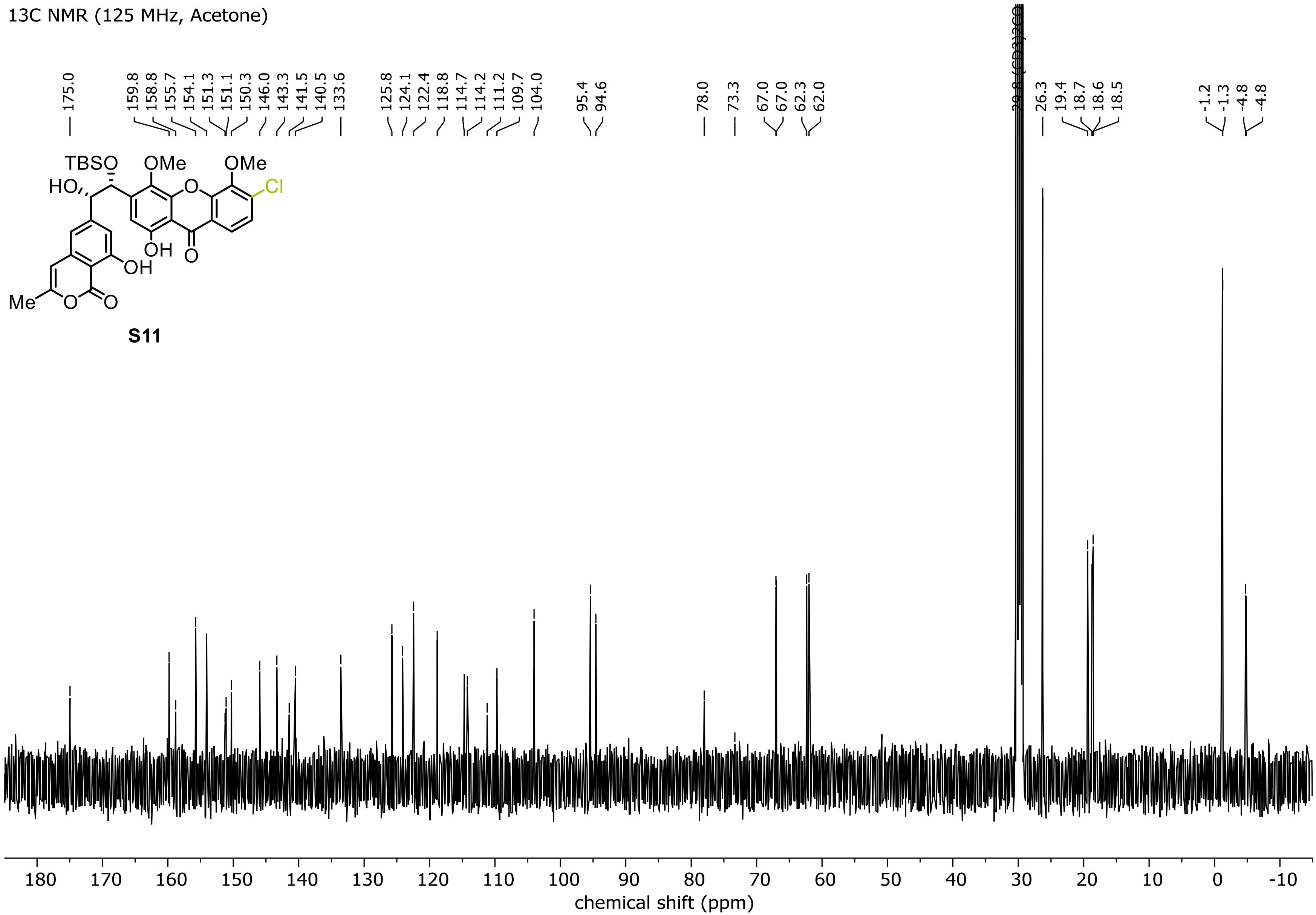

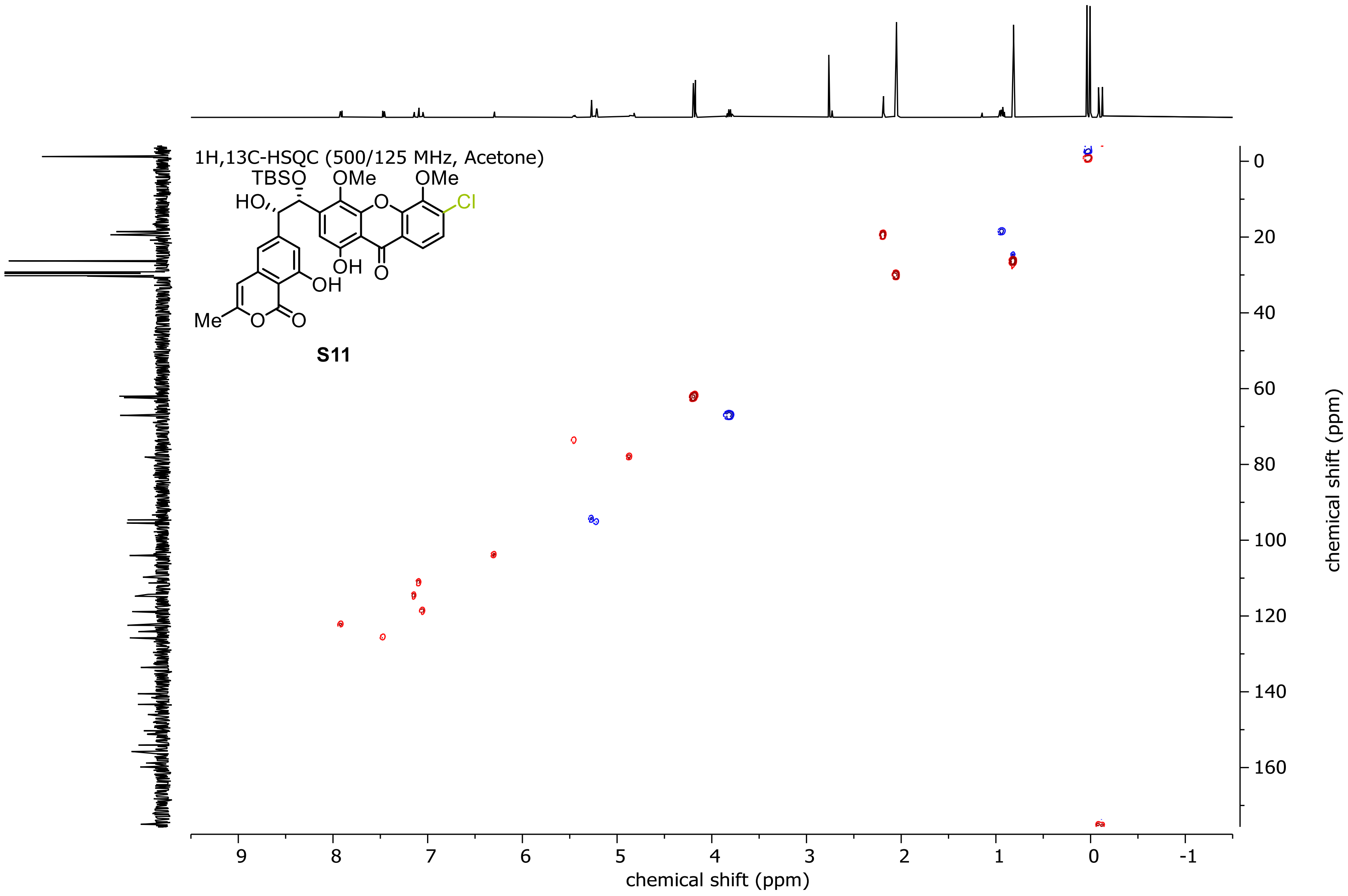
1H,13C-HSQC (500/125 MHz, Acetone)
TBSO
OMe
OMe
HO
O
Cl
OH
O
OH
Me
O
O
S11
0
20
40
60
80
100
120
140
160
chemical shift (ppm)
9
8
7
6
5
4
3
2
1
0
-1
chemical shift (ppm)

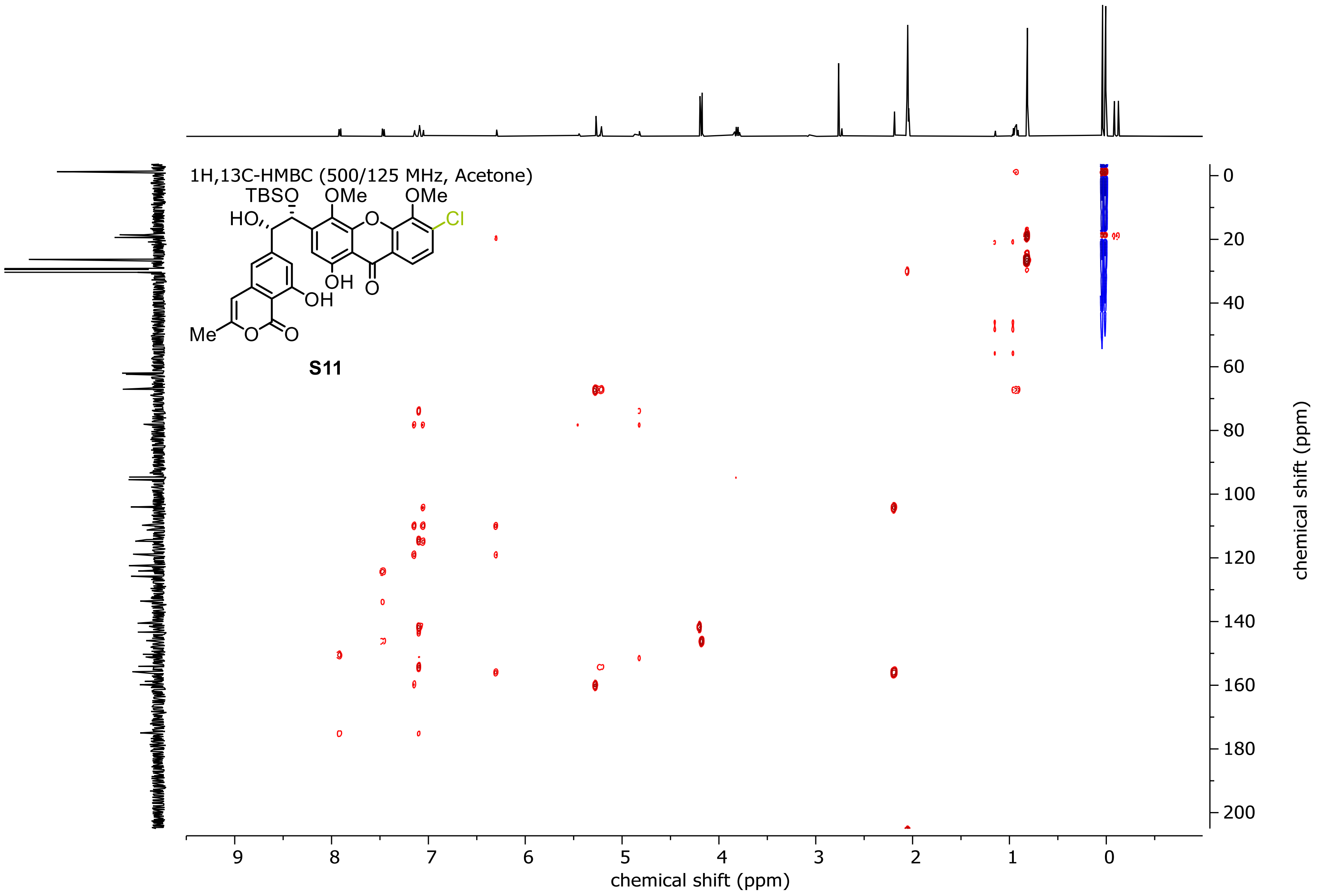

1H,13C-HMBC (500/125 MHz, Acetone)
TBSO
OMe
OMe
HO
O
Cl
OH
O
OH
Me
O
O
S11
chemical shift (ppm)
chemical shift (ppm)

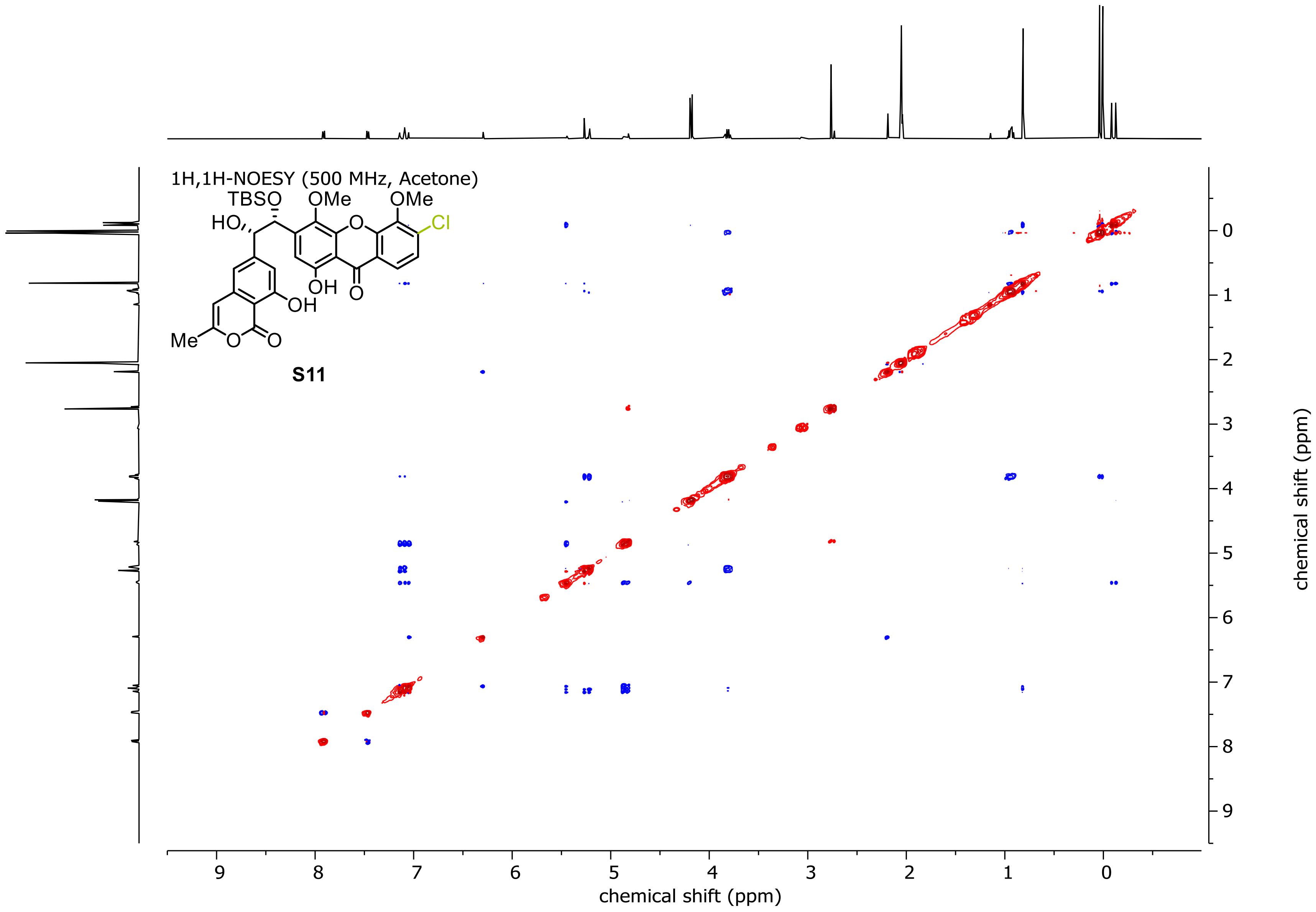
1H,1H-NOESY (500 MHz, Acetone)
TBSO
OMe
OMe
HO
Cl
OH
O
OH
Me
O
O
S11
chemical shift (ppm)
chemical shift (ppm)
9
8
7
6
5
4
3
2
1
0

1H NMR (700 MHz, Acetone)

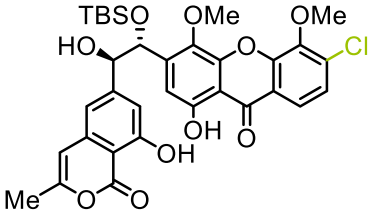

dia-**S11**

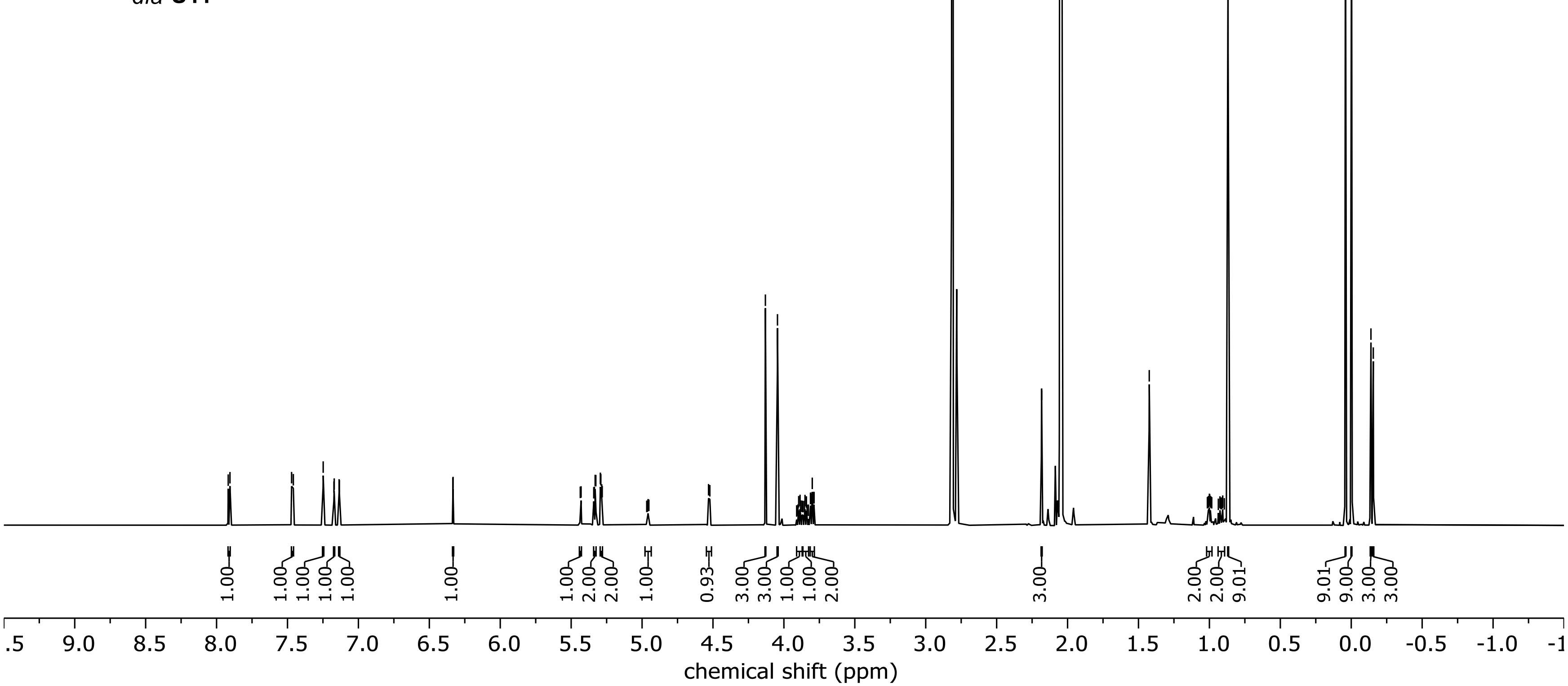

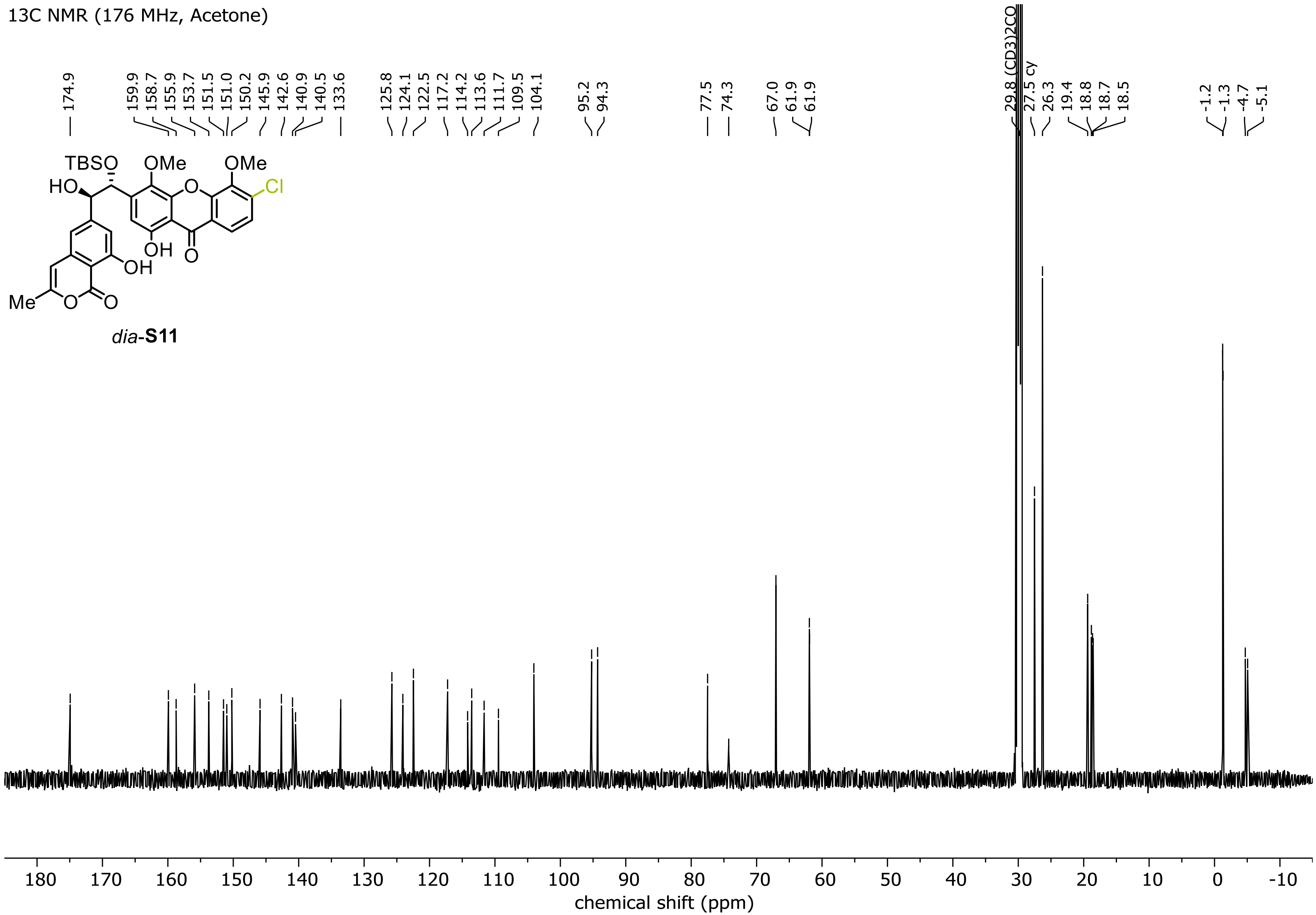
13C NMR (176 MHz, Acetone)
174.9
159.9
158.7
155.9
153.7
151.5
151.0
150.2
145.9
142.6
140.9
140.5
133.6
125.8
124.1
122.5
117.2
114.2
113.6
111.7
109.5
104.1
95.2
94.3
77.5
74.3
67.0
61.9
61.9
29.8 (CD3)2CO
27.5 cy
26.3
19.4
18.8
18.7
18.5
-1.2
-1.3
-4.7
-5.1
TBSO
HO
OMe
OMe
Cl
OH
OH
O
O
Me
O
O
dia-S11
180
170
160
150
140
130
120
110
100
90
80
70
60
50
40
30
20
10
0
-10
chemical shift (ppm)

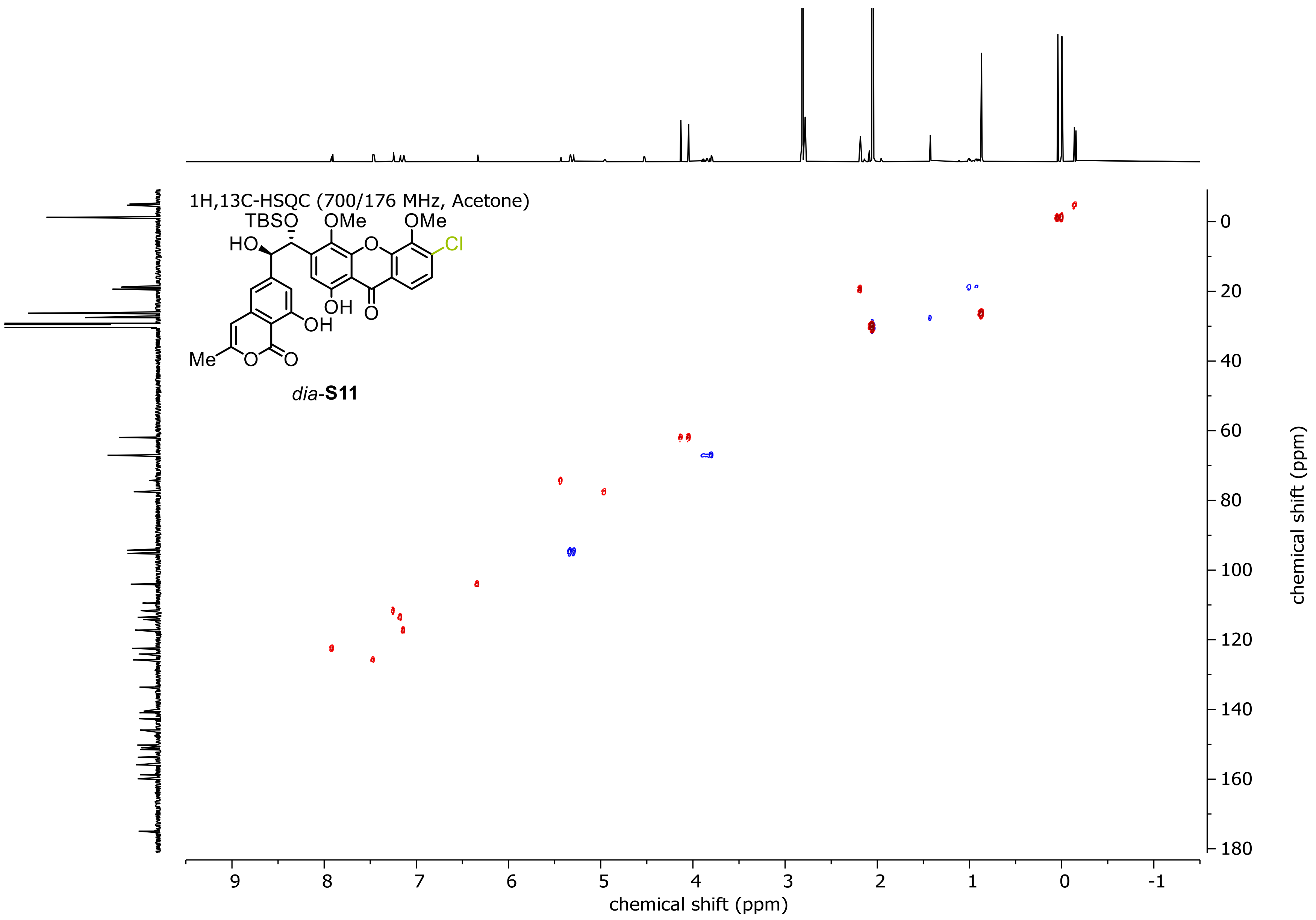
1H,13C-HSQC (700/176 MHz, Acetone)
TBSO
OMe
OMe
HO
O
Cl
OH
O
OH
Me
O
O
dia-**S11**
chemical shift (ppm)
chemical shift (ppm)

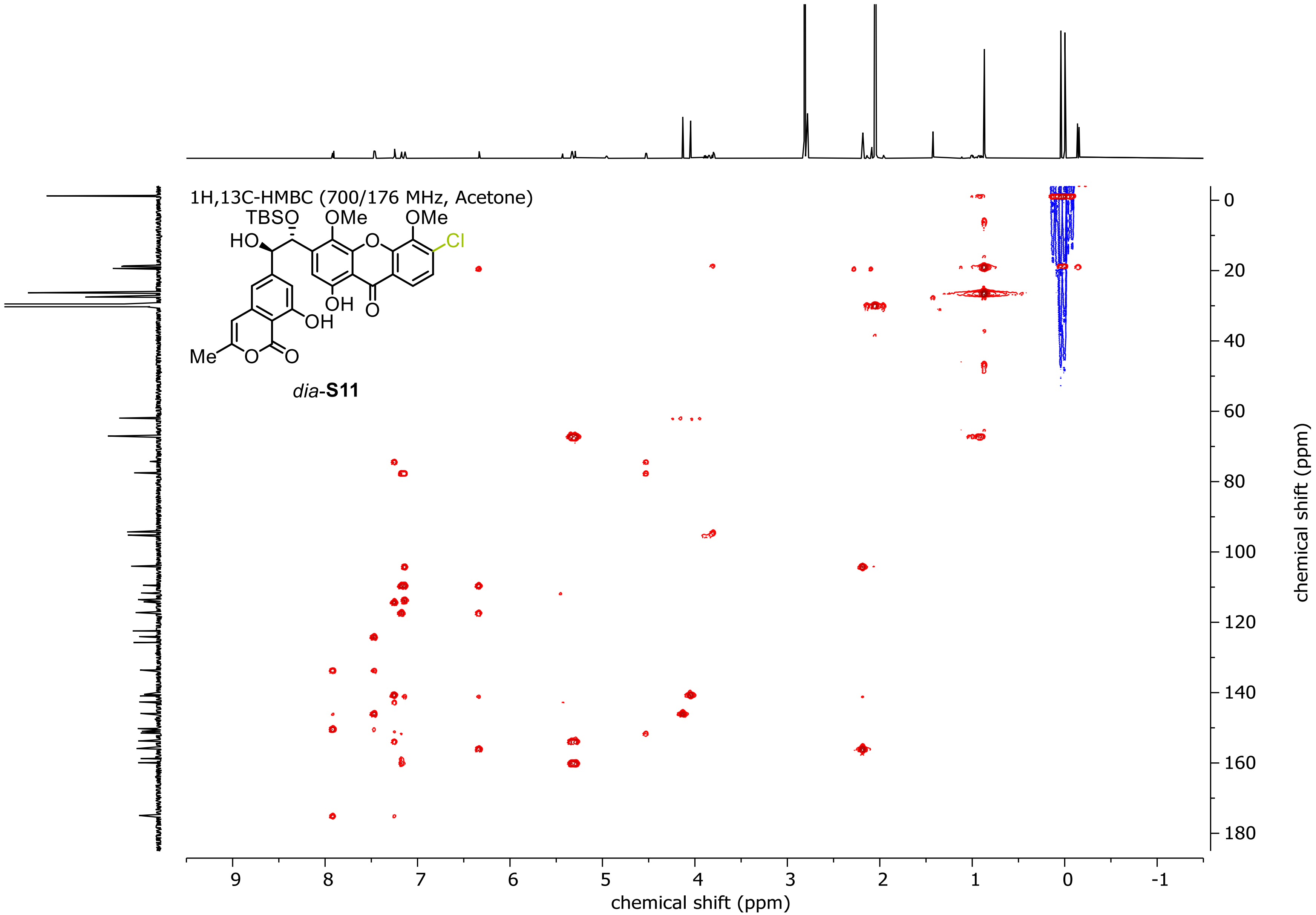
1H,13C-HMBC (700/176 MHz, Acetone)
TBSO
OMe
OMe
HO
Cl
OH
O
OH
Me
O
O
dia-S11
chemical shift (ppm)
chemical shift (ppm)

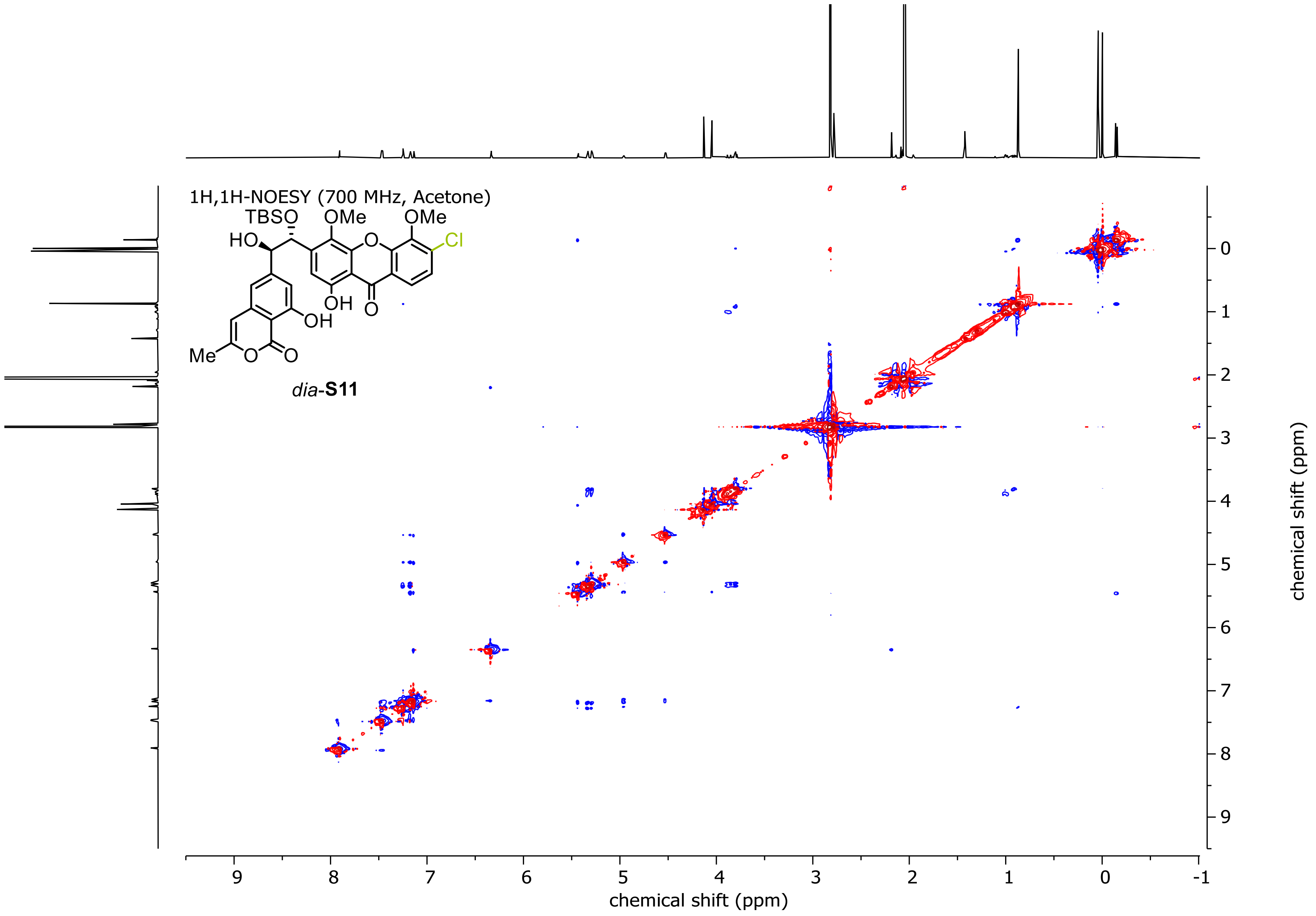
1H,1H-NOESY (700 MHz, Acetone)
TBSO
OMe
OMe
HO
Cl
OH
O
OH
Me
O
O
dia-S11
chemical shift (ppm)
chemical shift (ppm)

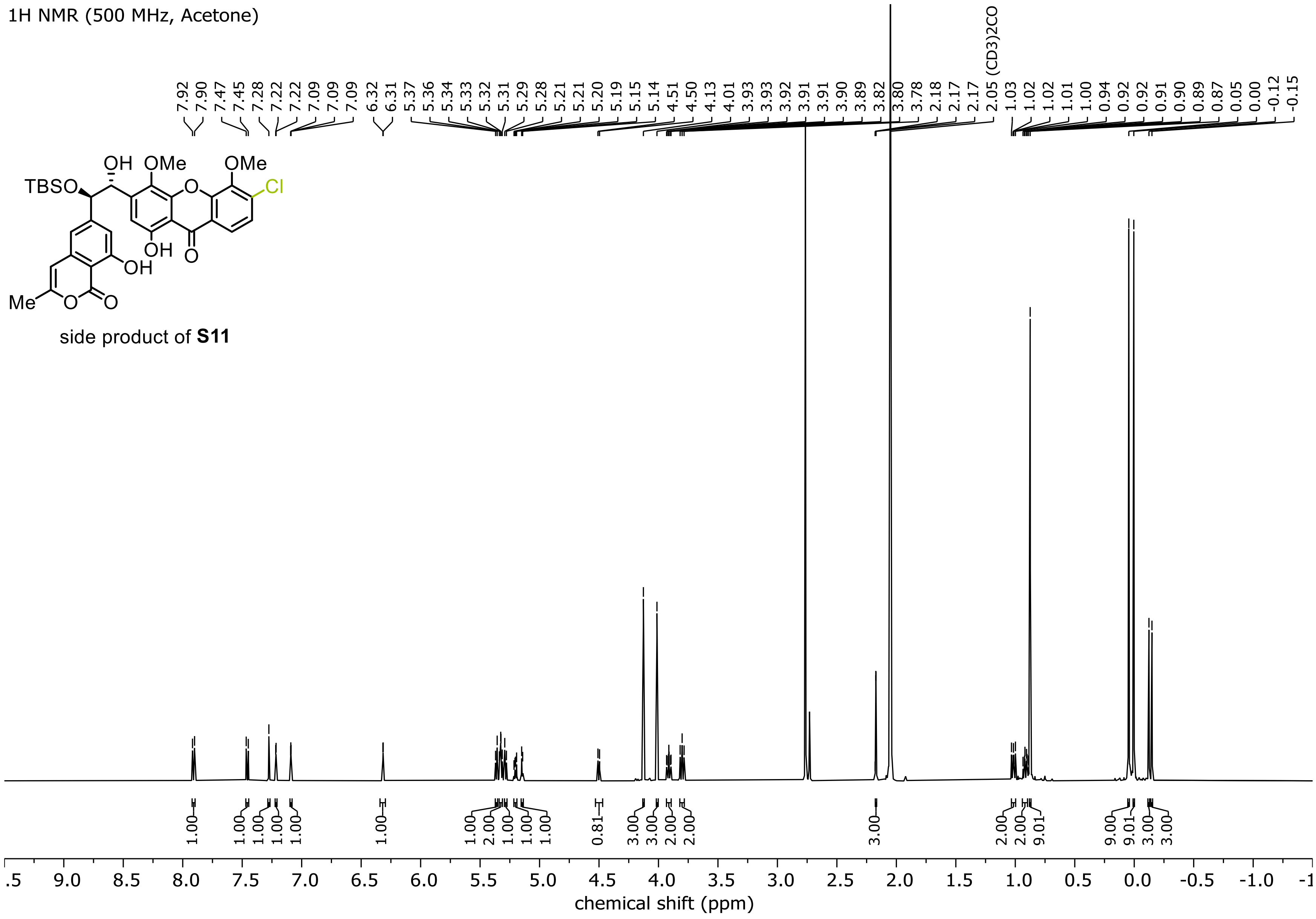
1H NMR (500 MHz, Acetone)
TBSO
OH
OMe
OMe
Cl
OH
OH
O
Me
side product of S11
(CD3)2CO
chemical shift (ppm)

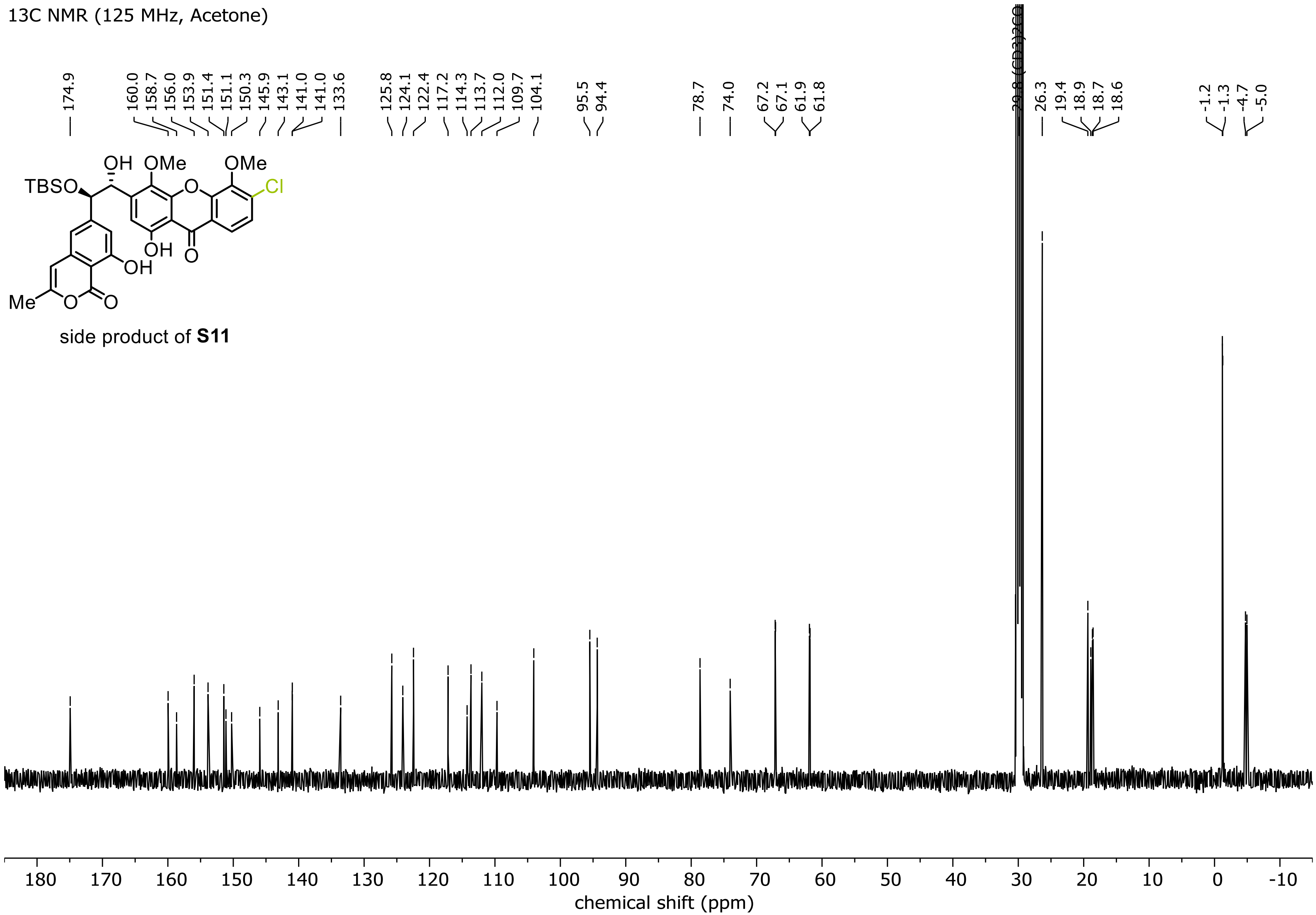
13C NMR (125 MHz, Acetone)
174.9
160.0
158.7
156.0
153.9
151.4
151.1
150.3
145.9
143.1
141.0
141.0
133.6
125.8
124.1
122.4
117.2
114.3
113.7
112.0
109.7
104.1
95.5
94.4
78.7
74.0
67.2
67.1
61.9
61.8
29.8 (CD3)2CO
26.3
19.4
18.9
18.7
18.6
-1.2
-1.3
-4.7
-5.0
OH
OMe
OMe
TBSO
O
Cl
OH
O
OH
Me
O
O
side product of **S11**
chemical shift (ppm)

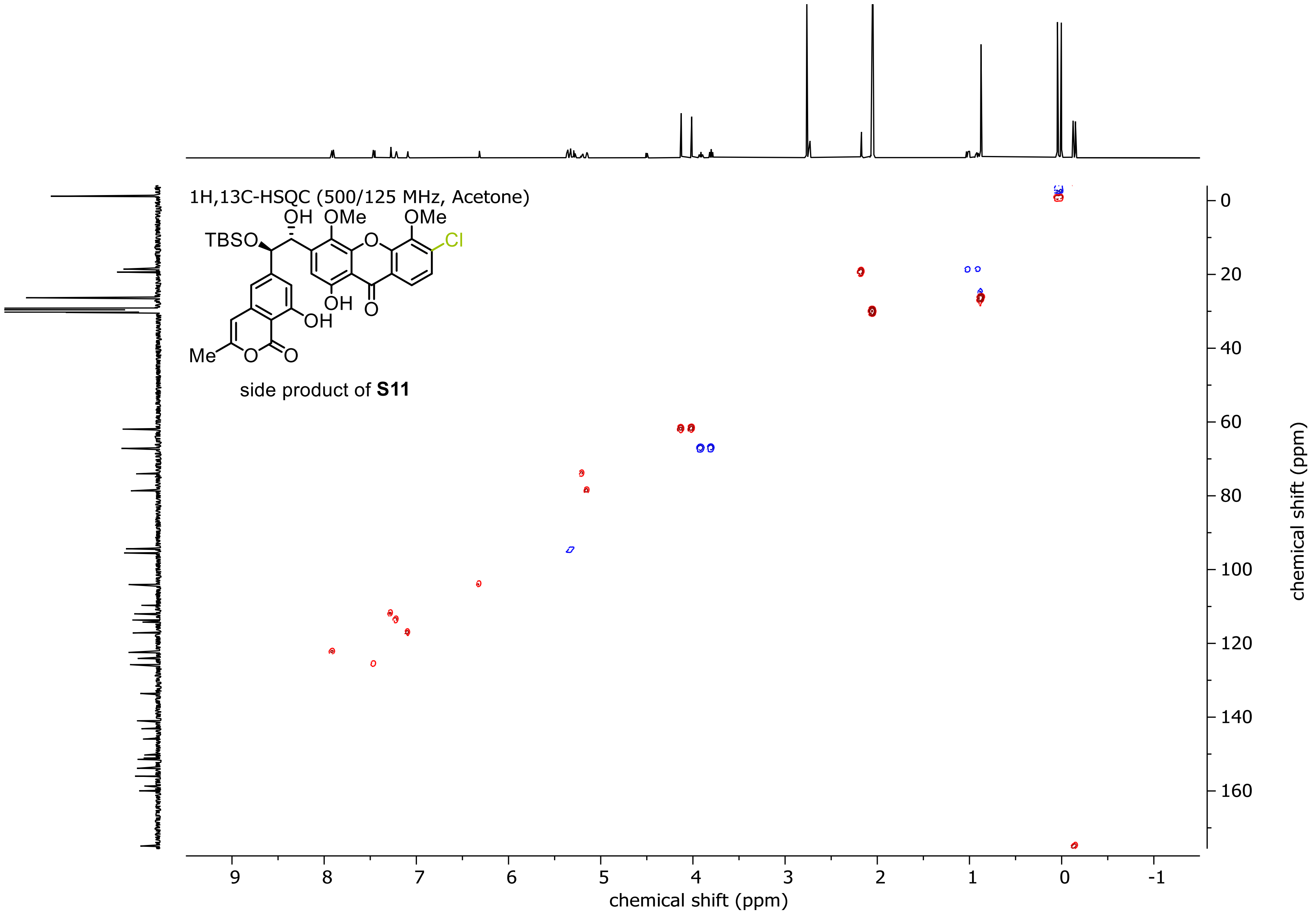
1H,13C-HSQC (500/125 MHz, Acetone)
OH OMe OMe
TBSO
Cl
OH O
OH
Me O O
side product of **S11**
chemical shift (ppm)
chemical shift (ppm)

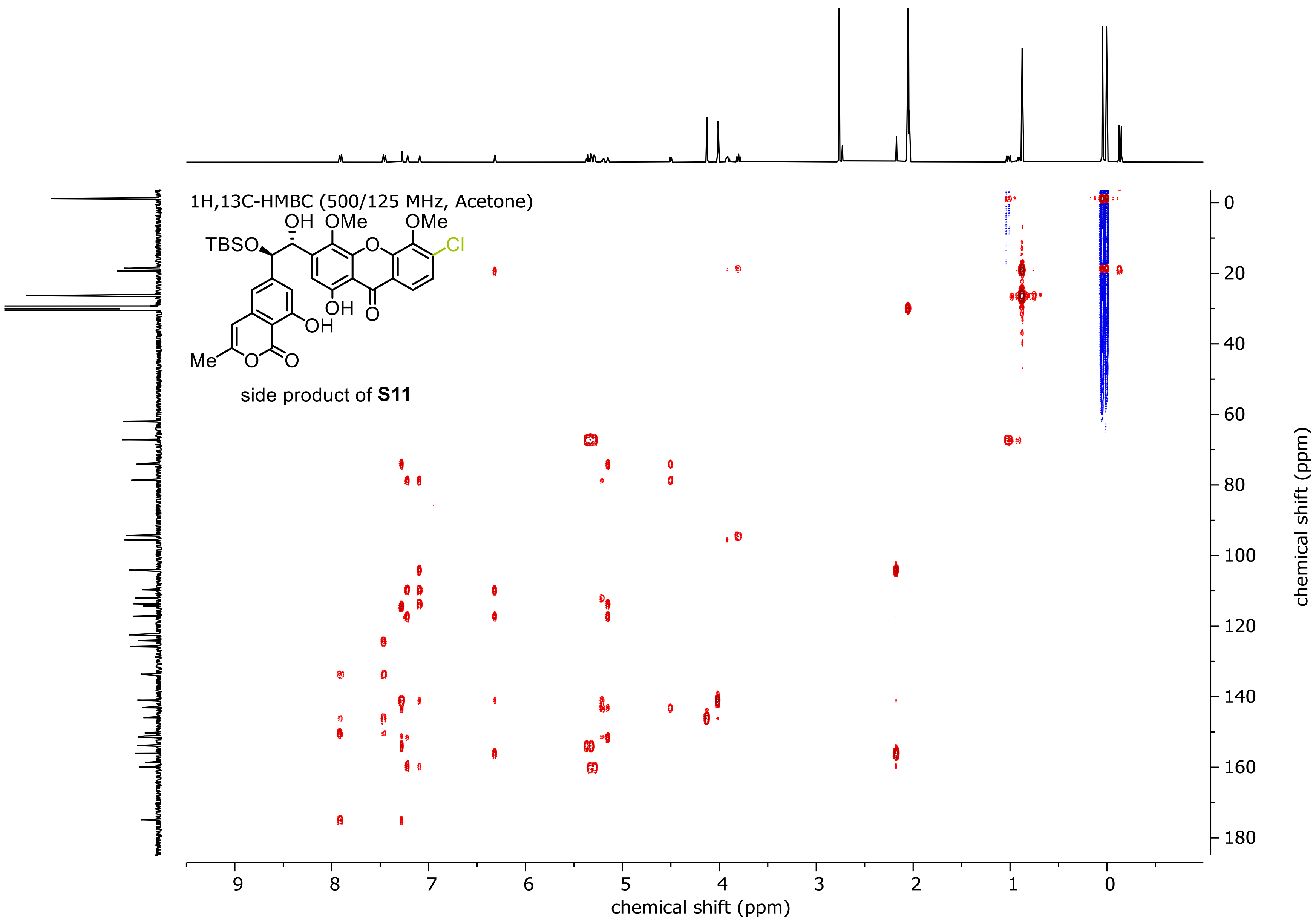
1H,13C-HMBC (500/125 MHz, Acetone)
TBSO
OH
OMe
OMe
O
Cl
OH
O
OH
Me
O
O
side product of **S11**
9
8
7
6
5
4
3
2
1
0
chemical shift (ppm)
0
20
40
60
80
100
120
140
160
180
chemical shift (ppm)

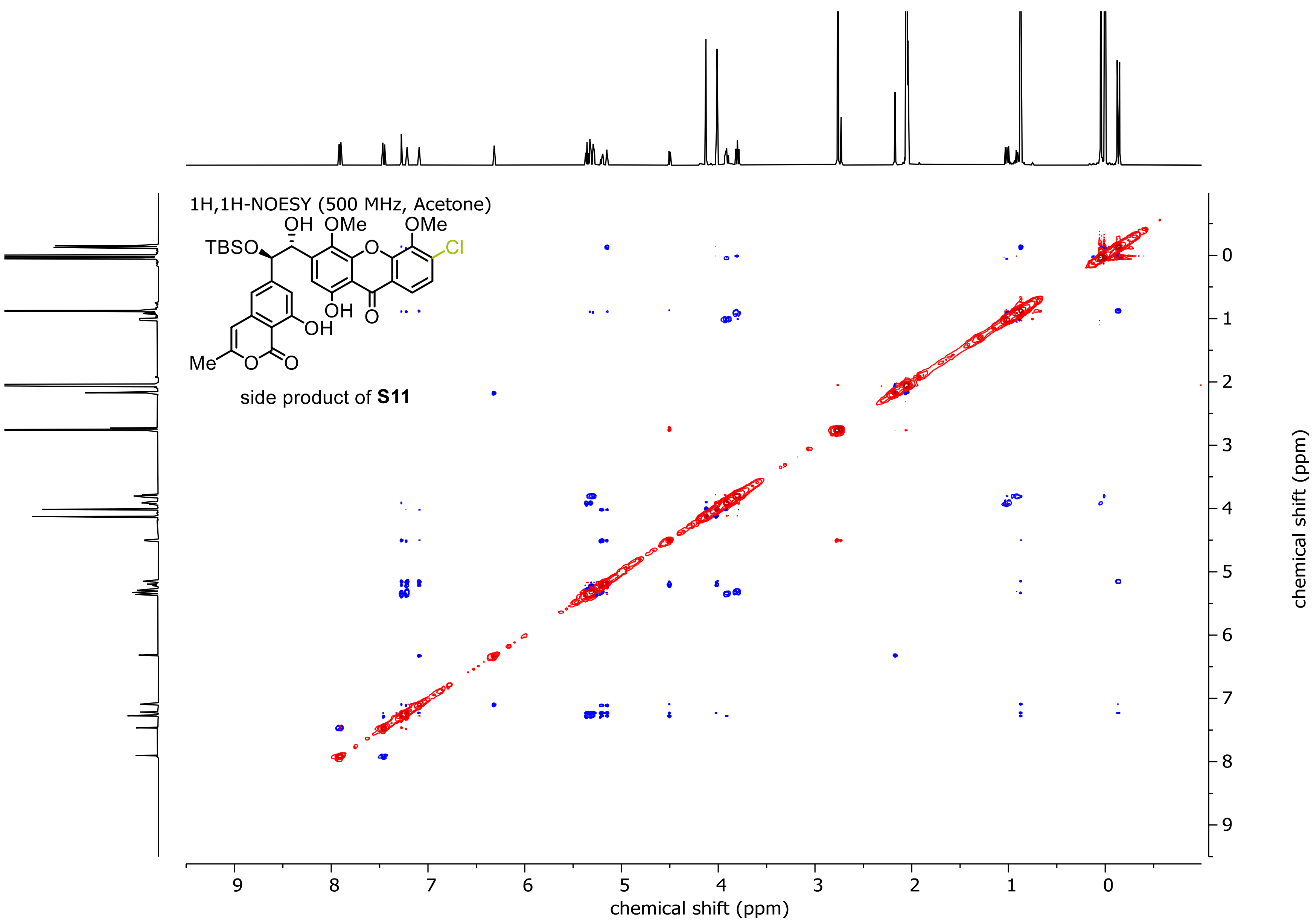

1H,1H-NOESY (500 MHz, Acetone)
OH
OMe
OMe
TBSO
O
Cl
OH
O
OH
Me
O
O
side product of **S11**
0
1
2
3
4
5
6
7
8
9
chemical shift (ppm)
9
8
7
6
5
4
3
2
1
0
chemical shift (ppm)

1H NMR (700 MHz, Acetone)

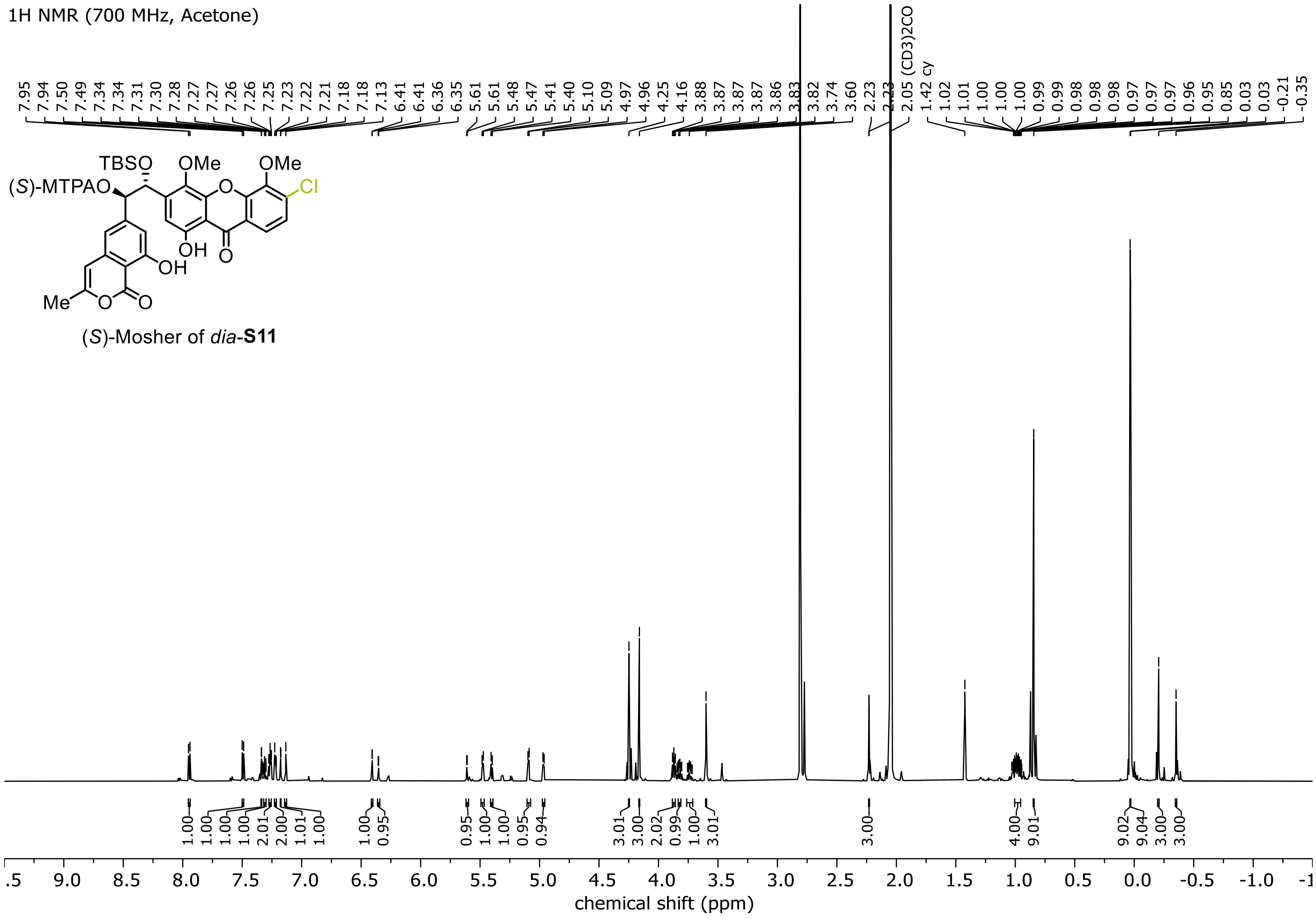

(*S*)-Mosher of *dia*-**S11**

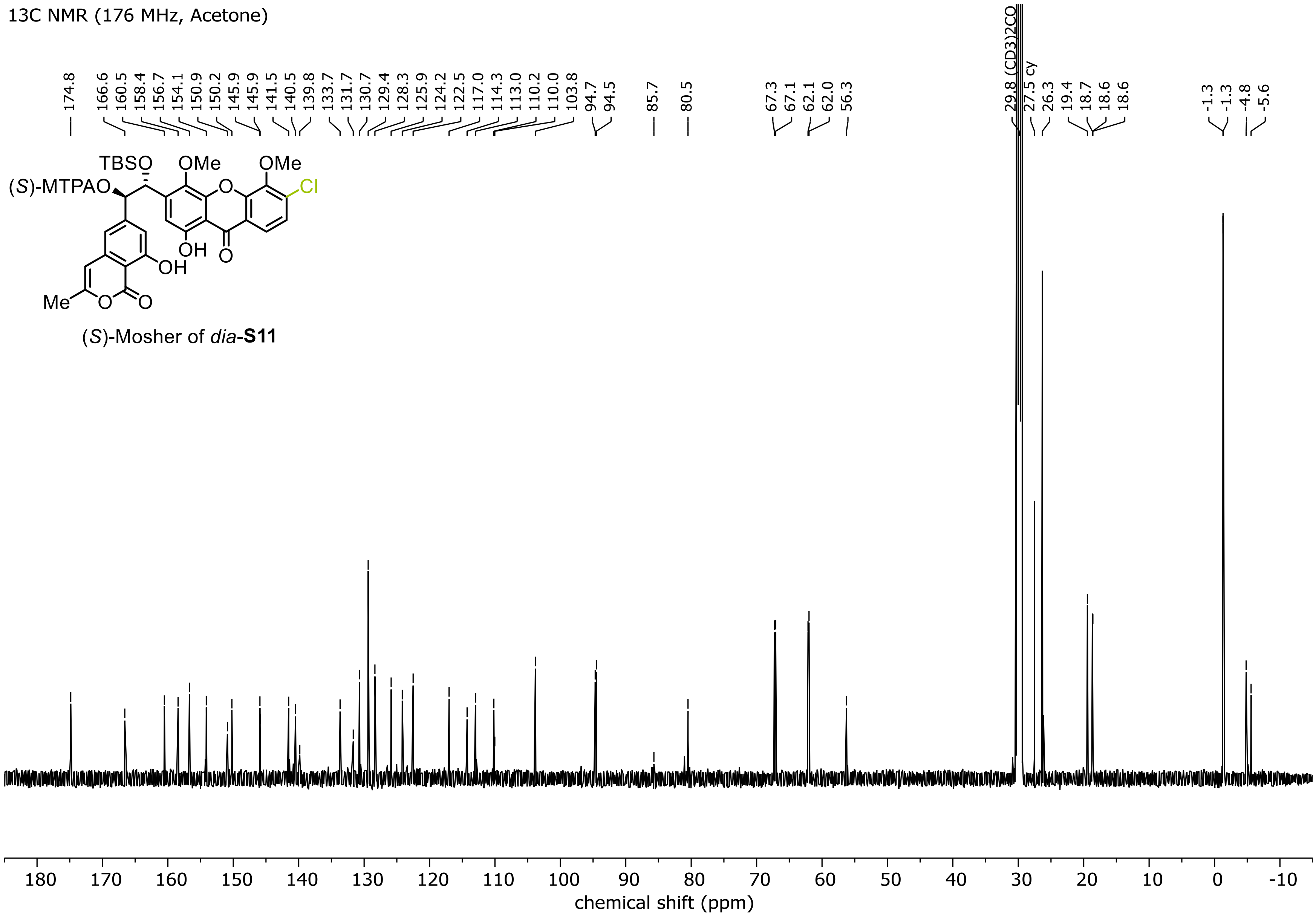
13C NMR (176 MHz, Acetone)
174.8
166.6
160.5
158.4
156.7
154.1
150.9
150.2
145.9
145.9
141.5
140.5
139.8
133.7
131.7
130.7
129.4
128.3
125.9
124.2
122.5
117.0
114.3
113.0
110.2
110.0
103.8
94.7
94.5
85.7
80.5
67.3
67.1
62.1
62.0
56.3
29.8 (CD3)2CO
27.5 cy
26.3
19.4
18.7
18.6
18.6
-1.3
-1.3
-4.8
-5.6
TBSO
OMe
OMe
(S)-MTPAO
O
Cl
OH
O
OH
Me
O
O
(S)-Mosher of dia-S11
180
170
160
150
140
130
120
110
100
90
80
70
60
50
40
30
20
10
0
-10
chemical shift (ppm)

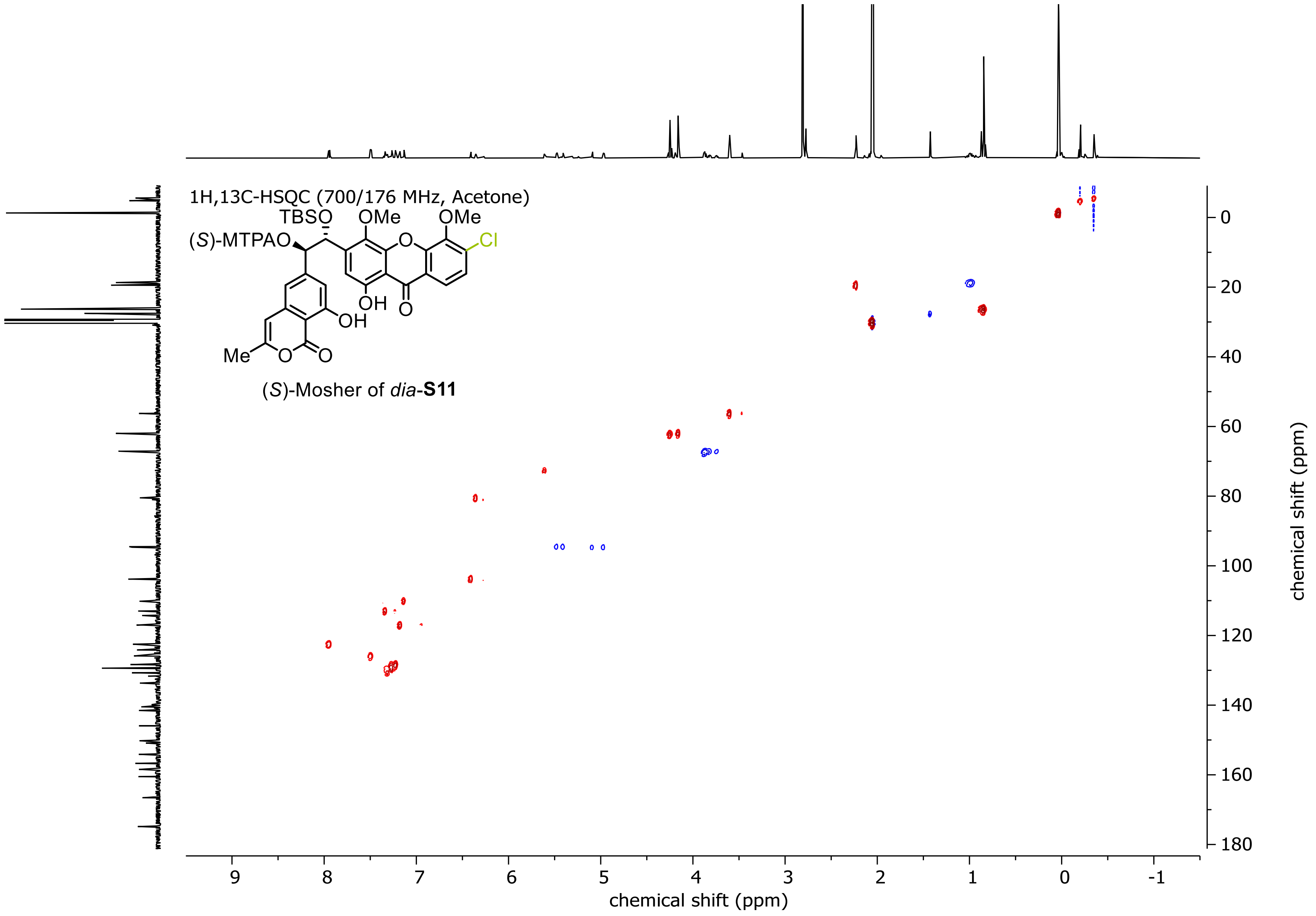
1H,13C-HSQC (700/176 MHz, Acetone)
TBSO
OMe
OMe
(S)-MTPAO
Cl
OH
O
OH
Me
O
O
(S)-Mosher of dia-S11
chemical shift (ppm)
chemical shift (ppm)

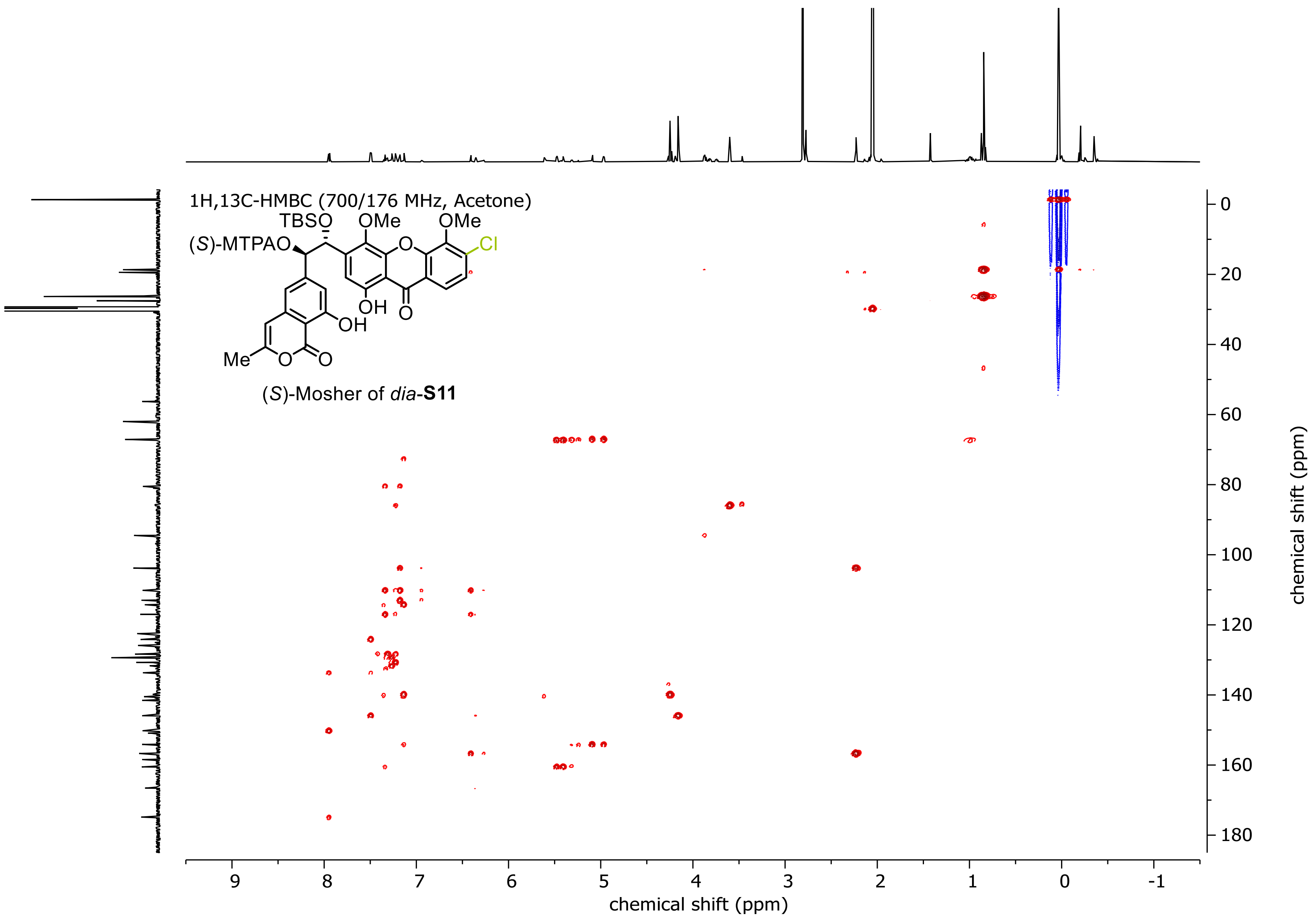
1H,13C-HMBC (700/176 MHz, Acetone)
TBSO
OMe
OMe
(S)-MTPAO
Cl
OH
O
OH
Me
O
O
(S)-Mosher of dia-S11
chemical shift (ppm)
chemical shift (ppm)

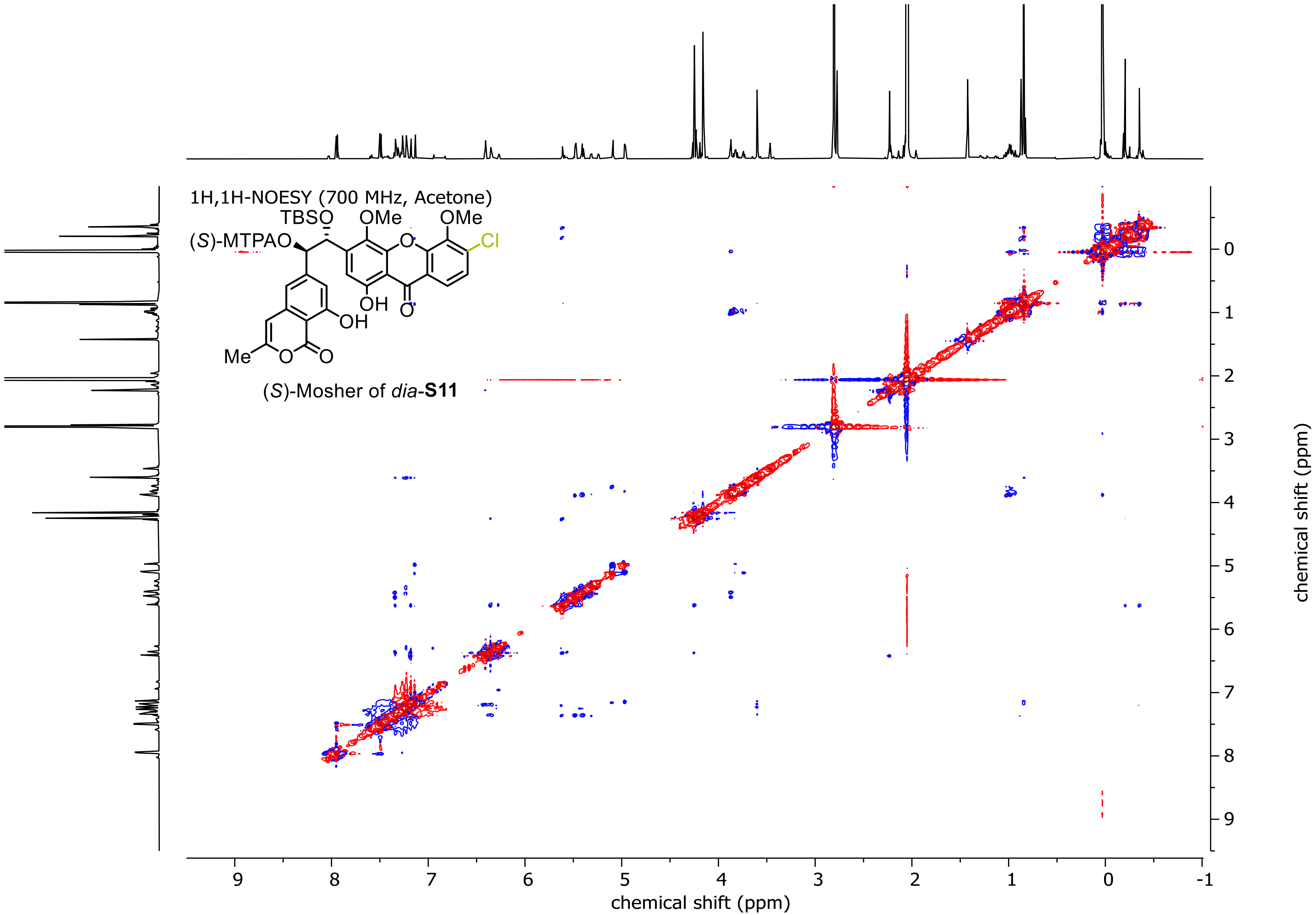

1H,1H-NOESY (700 MHz, Acetone)
TBSO
OMe
OMe
(S)-MTPAO
Cl
OH
OH
O
Me
O
O
(S)-Mosher of dia-S11
chemical shift (ppm)
chemical shift (ppm)

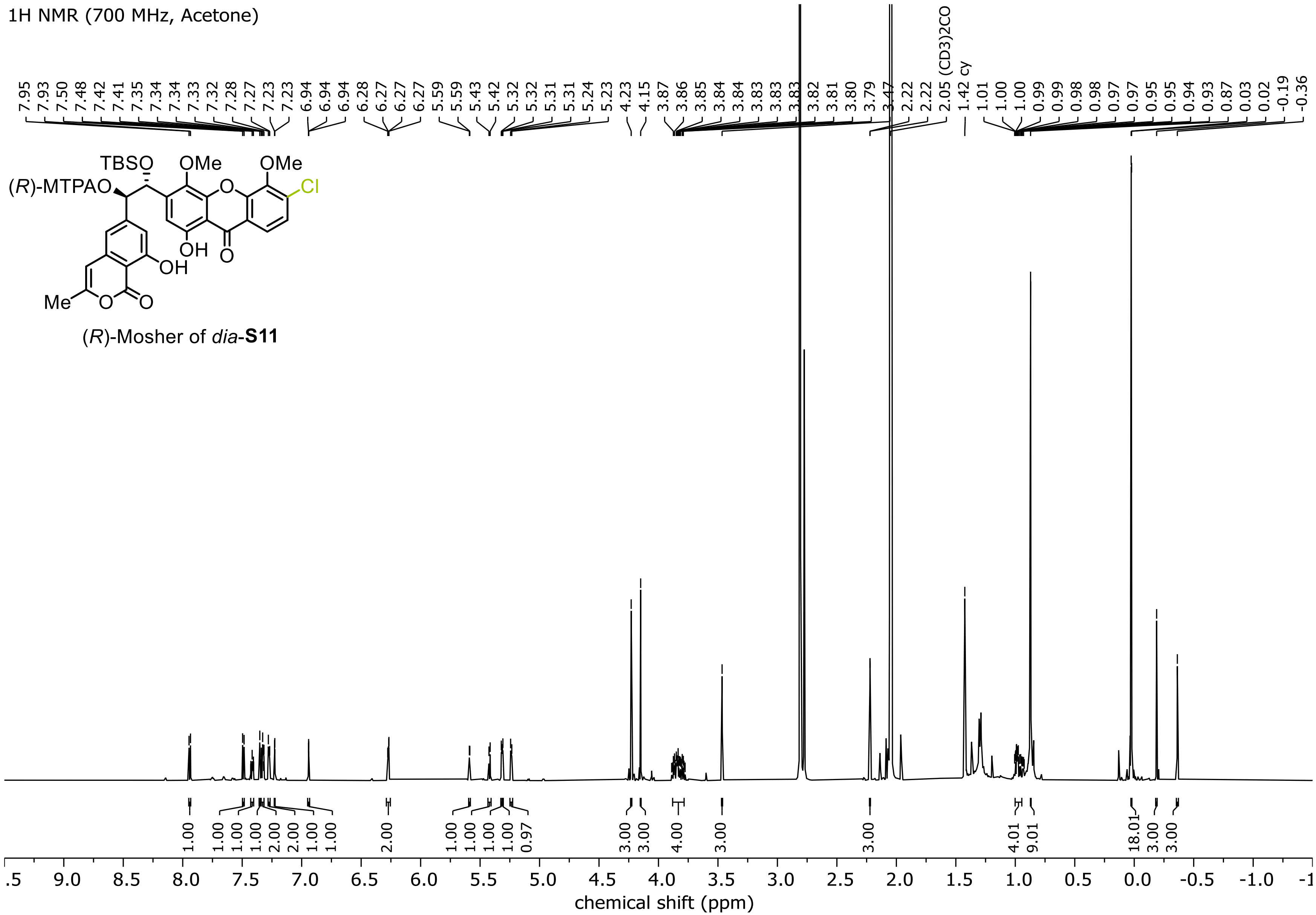
1H NMR (700 MHz, Acetone)
7.95 7.93 7.50 7.48 7.42 7.41 7.35 7.34 7.34 7.33 7.32 7.28 7.27 7.23 7.23 6.94 6.94 6.94 6.28 6.27 6.27 6.27 5.59 5.59 5.43 5.42 5.32 5.32 5.31 5.31 5.24 5.23 4.23 4.15 3.87 3.86 3.85 3.84 3.84 3.83 3.83 3.83 3.82 3.81 3.80 3.79 3.47 2.22 2.22 2.05 (CD3)2CO 1.42 cy 1.01 1.00 1.00 0.99 0.99 0.98 0.98 0.97 0.97 0.95 0.95 0.94 0.93 0.87 0.03 0.02 -0.19 -0.36
TBSO
OMe
OMe
(R)-MTPAO
Cl
OH
O
OH
Me
O
O
(R)-Mosher of dia-S11
1.00 1.00 1.00 1.00 2.00 2.00 1.00 1.00 2.00 1.00 1.00 1.00 1.00 0.97 3.00 3.00 4.00 3.00 3.00 4.01 9.01 18.01 3.00 3.00
chemical shift (ppm)

13C NMR (176 MHz, Acetone)

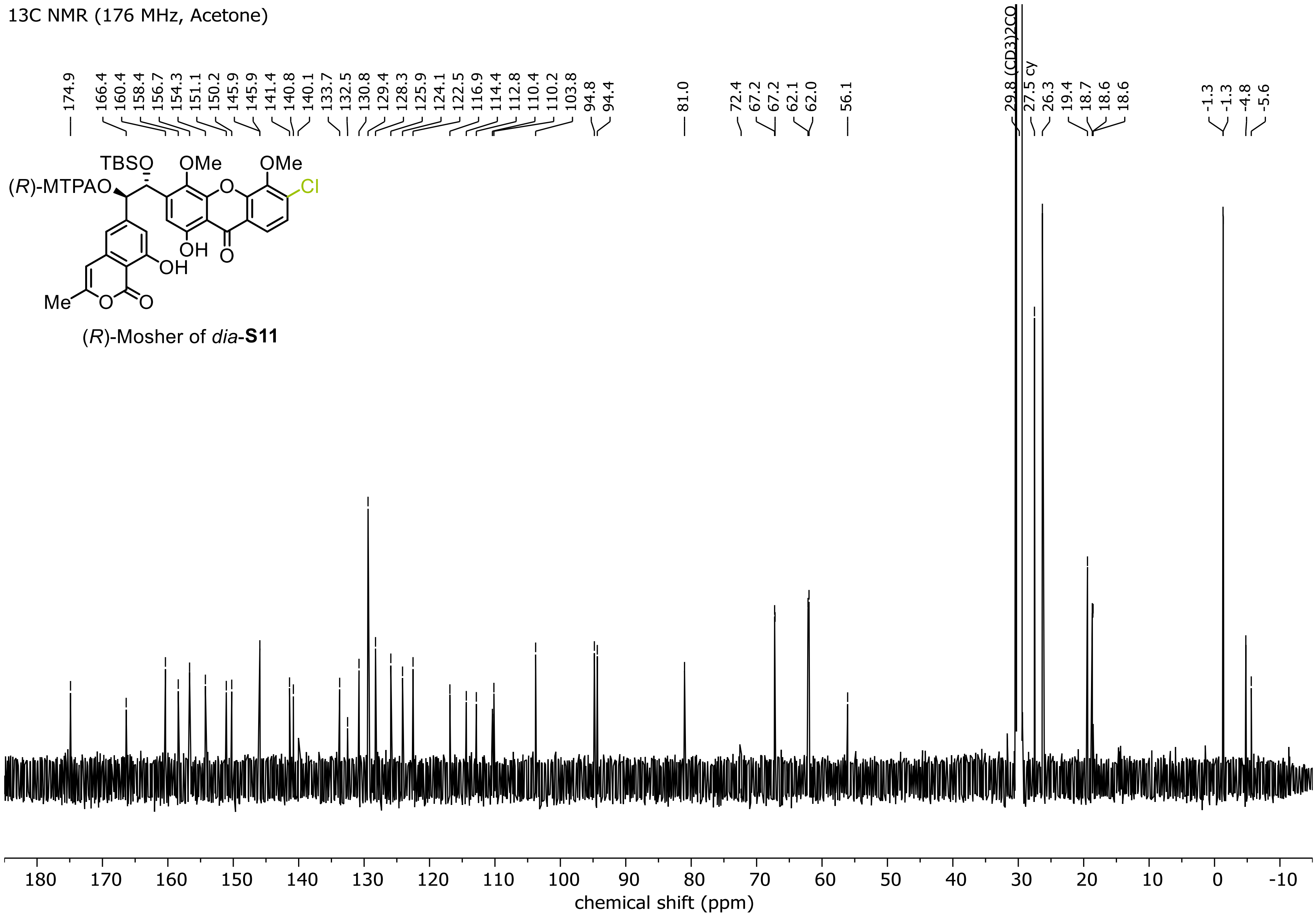

(*R*)-Mosher of *dia*-**S11**

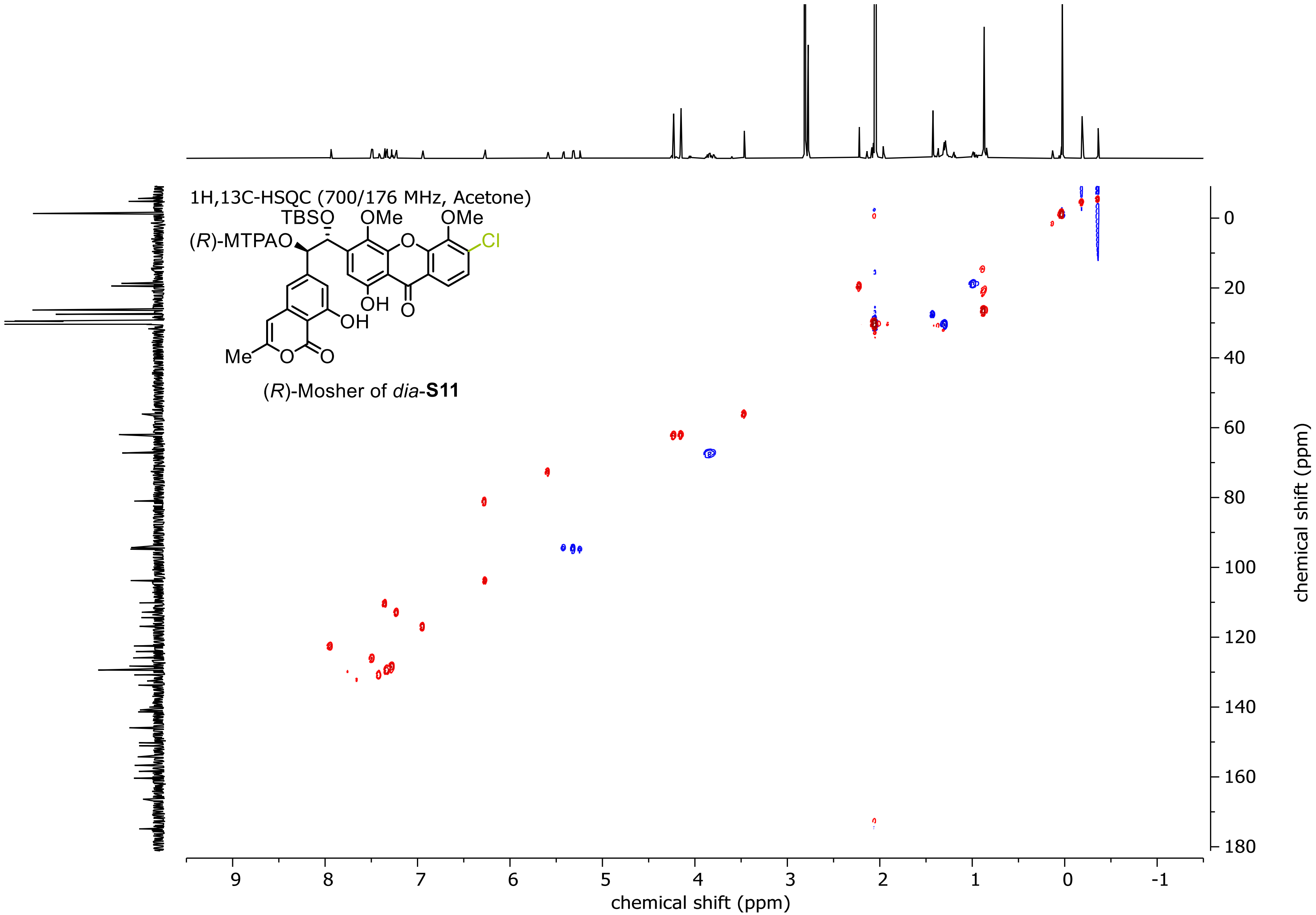
1H,13C-HSQC (700/176 MHz, Acetone)
TBSO
OMe
OMe
(R)-MTPAO
O
Cl
OH
O
OH
Me
O
O
(R)-Mosher of dia-S11
chemical shift (ppm)
chemical shift (ppm)

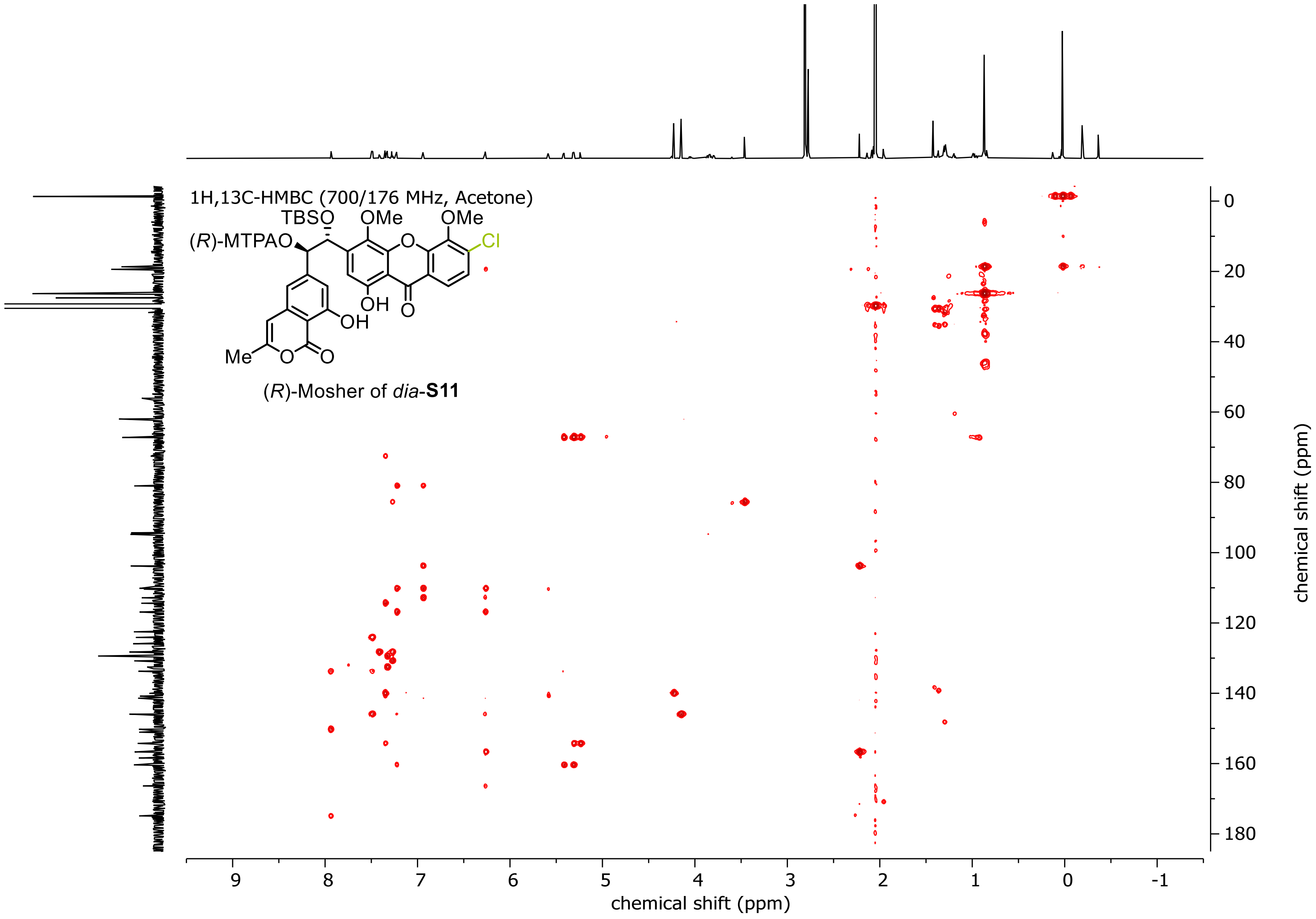
1H,13C-HMBC (700/176 MHz, Acetone)
TBSO
OMe
OMe
(R)-MTPAO
Cl
OH
O
OH
Me
O
O
(R)-Mosher of dia-S11
chemical shift (ppm)
chemical shift (ppm)

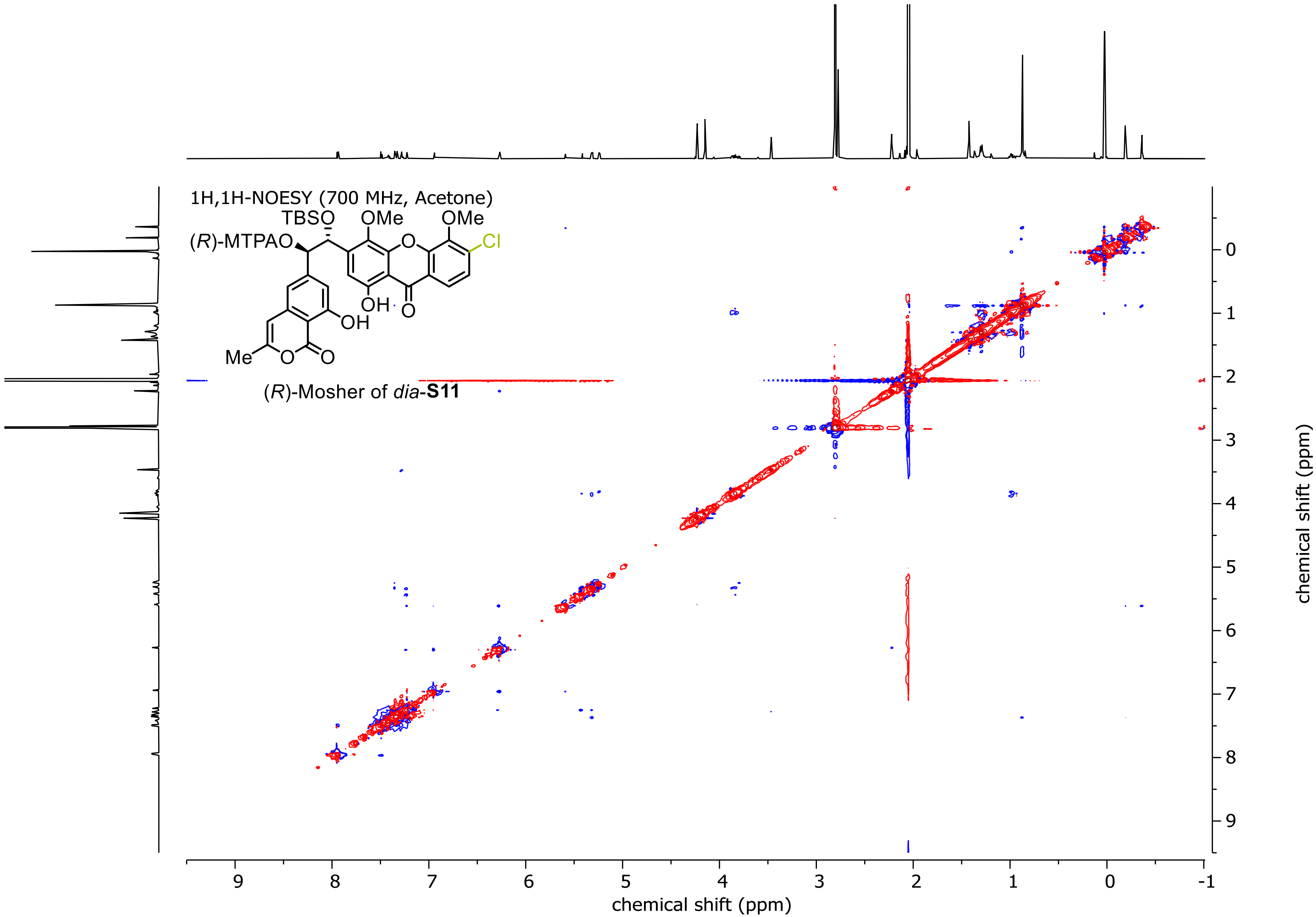
1H,1H-NOESY (700 MHz, Acetone)
TBSO
OMe
OMe
(R)-MTPAO
Cl
OH
O
OH
Me
O
O
(R)-Mosher of dia-S11
chemical shift (ppm)
chemical shift (ppm)
